# 33rd Annual Meeting & Pre-Conference Programs of the Society for Immunotherapy of Cancer (SITC 2018): Washington, D.C., USA. 7-11 November 2018

**DOI:** 10.1186/s40425-018-0423-x

**Published:** 2018-11-06

## Poster Presentations

### Co-Stimulatory Ligand-Receptor Interaction

#### P397 Ectopic Tim-3 expression on T regulatory cells leads to lymphoproliferation and T cell activation

##### Hridesh Banerjee, Héctor Nieves-Rosado, Lawrence P. Kane, Ph.D.

###### University of Pittsburgh, Pittsburgh, PA, USA

####### **Correspondence:** Lawrence P. Kane (lkane@pitt.edu)

**Background**

T cell (or transmembrane) immunoglobulin and mucin domain 3 (Tim-3) is a transmembrane protein that has been associated with both inhibitory and co-stimulatory function in T cells. In tumor-infiltrating (TI) T cells and during chronic infection, Tim-3 has been seen to be expressed in terminally exhausted T cells and a significant proportion of regulatory T cells (Treg). However, what role Tim-3 plays in Treg is still unclear. Another factor complicating the role of Tim-3 is that along with Tim-3, other checkpoint receptors such as PD-1 are also upregulated in TI-Treg and very little is known about crosstalk between various checkpoint receptors in effector T cells and Treg.

**Methods**

To investigate the role of Tim-3 in Treg, we used two mouse models, a constitutive Tim-3/Treg model (Foxp3-YFP-Cre x flox-stop-flox Tim-3) and a tamoxifen-inducible Treg/Tim-3 model (Foxp3-CreERT2 x flox-stop-flox Tim- 3).Basic characterisation of the immune system specifically the lyymphoid compartment and T cells including Treg cells was carried out. Functional assays on T regulatory cells was also done to look at effect of TIM-3 expression on T reg cells.

**Results**

At ten weeks after Tim-3 induction, Tim-3 transgenic mice had larger spleens and lymph nodes. This phenotype was observed to be milder in younger mice. Lymphoid organs in constitutive Tim-3 transgenic mice showed systemic lymphoid hyperplasia. T cells in these mice displayed a more activated phenotype. Overall frequency, numbers and phenotype of Treg cells in the peripheral lymphoid organs were also altered in constitutive Tim-3 transgenic mice. In the inducible Tim-3 mice however, we do not find systemic lymphoid hyperplasia but changes in numbers and phenotype of Treg were consistent with constitutive Tim-3 transgenic mice. Ectopic Tim-3 expression on Treg was also associated with changes in Treg function both in vitro and in vivo.

**Conclusions**

TIM-3 is sufficient to change the basic regulatory function of T reg cells, thereby studying how checkpoint therapies effect T reg in tumormicroenvironment and chronic infection may lead us to better Understanding the role of Tim-3 in Treg, and could contribute to novel therapeutic approaches for diseases such as cancer and chronic infection.

#### P398 Activation of the T Cell costimulatory protein CD137 using multivalent bicyclic peptides

##### Kristen Hurov, Punit Upadhyaya, Jessica Kublin, Xueyuan Zhou, Julia Kristensson, Rachid Lani, Gemma Mudd, Katerine van Rietschoten, W. Frank An, Johanna Lahdenranta, Liuhong Chen, Gavin Bennett, Kevin McDonnell, Nicholas Keen, Peter U. Park, PhD

###### Bicycle Therapeutics, Lexington, MA, USA

####### **Correspondence:** Peter U. Park (peter.park@bicycletx.com)

**Background**

CD137 (4-1BB/TNFRSF9) is a costimulatory receptor belonging to the TNF receptor superfamily. It was originally cloned as an inducible gene from stimulated helper and cytotoxic T cells and has since been shown to also be expressed on natural killer (NK) cells. Agonistic anti-CD137 antibodies have shown potent, often curative anti-tumour activity in preclinical models. These effects are mainly mediated by cytotoxic T cells and generate long lasting, memory responses. Two human anti-CD137 antibodies, binding to the extracellular domain of CD137, urelumab and utomilumab are currently undergoing clinical testing. Urelumab has shown several single-agent, partial responses, but its use has been hampered by hepatoxicity, whilst utomilumab has shown little or no single agent activity.

**Methods**

Bicycles® are a new class of drugs - fully synthetic, constrained bicyclic peptides that combine the attributes of three therapeutic modalities (antibodies, small molecules, and peptides) by delivering high affinity, good PK, and rapid clearance. Their small size (1.5-2 kDa) delivers advantages in tumour penetration, and rapid renal elimination may avoid the liver and GI toxicity often associated with other drug modalities, including certain antibodies. We hypothesised that a fully synthetic Bicycle CD137 agonist with rapid renal clearance, minimal liver interaction and no Fc receptor interaction may induce CD137 mediated anti-tumour activity while avoiding liver toxicity. We screened for CD137 binders with a library of 10e12 Bicycles using phage display and following phage and chemical optimization, a high affinity lead BCY3814 (KD ~30 nM) was selected.

**Results**

BCY3814 binds to the human CD137 ligand-binding site. In common with many TNF receptors, CD137 activation requires receptor crosslinking, thus multivalent binders would be expected to recapitulate the action of its natural trimeric ligand. We generated more than 50 different bi-, tri- and tetra-valent variants of BCY3814 with chemical linkers and hinges of various lengths and rigidity using different sites of attachments, while maintaining a compact size (<15 kDa). We developed molecules exhibiting a wide range of potency in a cell-based CD137-dependent reporter assay. In addition, these molecules activate human T cells in vitro as monitored by increased cytokine release. Selected CD137 multimers are being tested in a humanized CD137 mouse model to demonstrate T cell activation and anti-tumour activity, without the liver toxicity reported for urelumab.

**Conclusions**

We hypothesise that such molecules could be promising, novel cancer immunotherapy candidates and importantly, they pave the way for development of synthetic agonists of other TNF receptors.

#### P399 Induction of tumor-specific immune responses and modulation of the tumor micro-environment by TLR9 agonist lefitolimod in murine syngeneic tumor models

##### Kerstin Kapp, PhD^1^, Barbara Volz^1^, Detlef Oswald^1^, Burghardt Wittig, MD, PhD^2^, Manuel Schmidt, MSc^1^

###### ^1^Mologen AG, Berlin, Germany; ^2^Advisor to Mologen AG, Berlin, Germany

**Background**

Preclinical and ongoing clinical studies support the application of TLR9 agonists for immunotherapy. The immune surveillance reactivator (ISR) lefitolimod is in advanced clinical development for single-agent maintenance treatment in metastatic colorectal cancer (phase III, IMPALA) and extensive disease small cell lung cancer (phase II, IMPULSE). Lefitolimod activates plasmacytoid dendritic cells to secrete interferon-alpha, followed by a broad activation of cells of the innate and adaptive immune system. Lefitolimod therefore provides the necessary and sufficient signals for the initiation of an immunotherapeutic anti-tumor response.

**Methods**

It was evaluated, if lefitolimod is able to induce local and systemic anti-tumor immune responses in the murine syngeneic colon carcinoma CT26 and the breast cancer EMT-6 models. The presence and activation state of CD8+ T cells within tumor infiltrating cells was determined via flow cytometry. Tumor antigen-specific T cells were analyzed via IFN-gamma ELISpot using spleen cells stimulated with either tumor cells or the peptide AH1, derived from an immunodominant antigen of CT26 cells.

**Results**

Intratumoral administration of lefitolimod resulted in a beneficial modulation of the tumor micro-environment (TME) characterized by increased infiltration of activated CD8+ T cells, which showed an up-regulation of Granzyme B. Notably, an increase of IFN-gamma secreting CD8+ T cells within the spleen was detected after re- stimulation with the tumor-specific AH1 peptide antigen or CT26 tumor cells. This beneficial TME modulation and antigen-specific effects were associated with a markedly reduced tumor growth in the CT26 model. The anti-tumor effect was even more pronounced in the EMT-6 model, where nine out of ten mice showed complete tumor regression. The 9 tumor-free mice subsequently rejected both, the initially used EMT-6 as well as CT26 tumor cells in re-challenge studies, in contrast to age-matched naïve mice. This indicates that treatment with lefitolimod induces a sustained, long-lasting immune memory against shared antigens of both tumor types.

**Conclusions**

Treatment of tumors with lefitolimod resulted in a beneficial modulation of the TME with an increase in anti-tumor effector cells. A strong systemic immune response as well as a sustained immune memory against different tumors was induced. These data indicate that lefitolimod provides the essential requirements for use as mono- immunotherapy or as an optimal combination partner of other immunotherapeutic drugs like checkpoint inhibitors in immuno-oncological trials.

#### P400 Tumor-localizing NKp30/ICOSL vIgD fusion proteins direct effective dual CD28/ICOS T cell costimulation to B7-H6+ tumor cells in vitro and tumors in vivo

##### Steven Levin, PhD^2*^, Lawrence Evans, BS^*2*^, Erika Rickel^*2*^, Katherine Lewis, PhD^*2*^, Daniel Demonte^*2*^, Martin Wolfson, BS^*2*^, Stacey Dillon, PhD^*2*^, Ryan Swanson, BS^*2*^, Kristine Swiderek, PhD^*2*^, Stanford Peng, MD, PhD^*2*^

###### ^1^Alpine Immune Sciences, Inc., Seattle, WA, USA; ^2^Alpine Immune Sciences, Seattle, WA, USA

####### **Correspondence:** Steven Levin (steve.levin@alpineimmunesciences.com)

**Background**

Background: Although checkpoint inhibitor therapies have significantly improved outcomes in multiple cancers, complete and durable responses remain infrequent, possibly attributable to a lack of adequate T cell costimulation and/or activating signals. Novel therapeutic proteins which confer T cell costimulation may be particularly effective anti-tumor therapies, particularly in combination with checkpoint inhibitors. But at the same time, localization of such costimulatory activity to tumors, such as via a tumor-specific targeting antigen, may be simultaneously important to maintain tolerability of such agonist therapeutics. B7-H6, a cell surface immunoglobulin superfamily (IgSF) member which binds the NKp30 receptor, appears to be expressed specifically in multiple tumor types, and may serve as such a tumor-specific antigen. Novel therapeutic proteins which localize costimulatory agonist domains to B7 H6 may therefore be capable of significant antitumor efficacy yet may be safely administered systemically by preferentially localizing agonist activity to the B7-H6 tumor microenvironment.

**Methods**

Methods: Using our platform technology, which is based on the directed evolution of IgSF members, NKp30/ICOSL variant immunoglobulin domain (vIgD) fusion proteins were created from NKp30 vIgDs with high affinity against B7-H6 and ICOSL vIgDs, which dually agonize the T cell costimulatory receptors ICOS and CD28. These tumor- localizing vIgD proteins were evaluated in vitro in T cell costimulation assays with target cells with or without B7- H6, and in vivo in a B7-H6+ CT26 mouse tumor model.

**Results**

Results: NKp30/ICOSL vIgD-Fc fusion proteins conferred effective B7-H6-dependent costimulation to T cells in vitro, with enhanced T cell proliferation and cytokine production (IFN-gamma, TNF-alpha, IL-2) in response to B7- H6-expressing but not B7-H6-negative target cells. Isolated ICOSL and NKp30 vIgDs alone were not efficacious. Importantly, NKp30/ICOSL vIgD-Fc fusion proteins demonstrated anti-tumor efficacy in a B7 H6+ mouse tumor model, especially when administered in combination with a PD-1 inhibitor (Figure 1).

**Conclusions**

Conclusions: Tumor-localizing NKp30/ICOSL vIgDs confer potent T cell costimulation via CD28 and ICOS dependent upon the tumor antigen B7-H6 and elicit encouraging efficacy against B7-H6+ tumors in vivo, including in combination with PD-1 inhibitors. Such fusion proteins may provide particularly effective therapeutics for B7- H6+ tumors either as monotherapy or in combination with checkpoint blockade. These findings further suggest tumor-localized immunomodulation is possible and may improve cancer outcomes.

**Ethics Approval**

All animal procedures were approved by the appropriate Institutional Animal Care and Use Committee overseeing the vivarium where the studies were conducted (Alpine Immune Sciences and Charles River Laboratories), and followed the guidelines set forth in the 8th Edition of the Guide for the Care and Use of Laboratory Animals (National Research Council, 2011).

**Fig. 1 (abstract P400).**
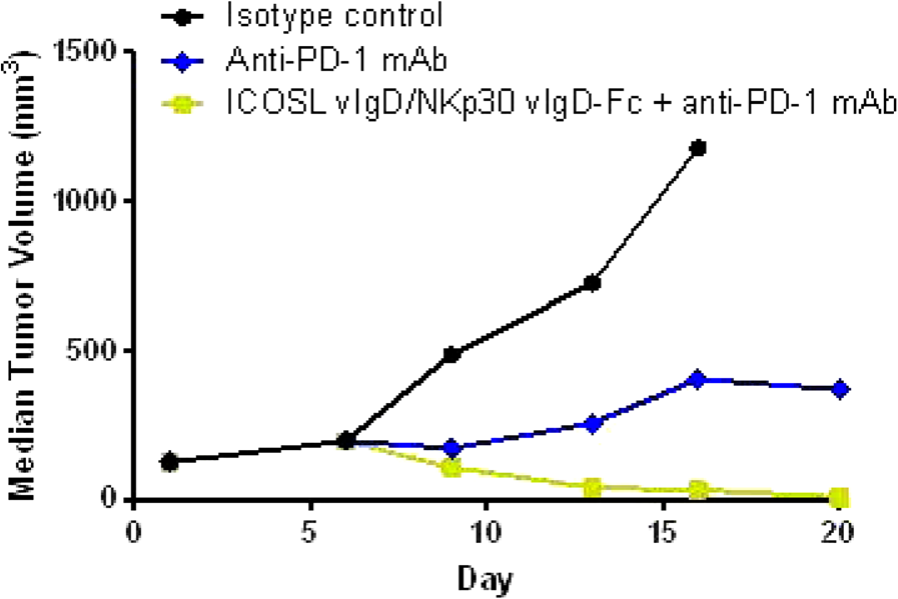
See text for description

#### P401 Blockade of T cell immunoreceptor with Ig and ITIM domains (TIGIT) leads to increased proliferation of bone marrow T cells from patients with acute myeloid leukemia (AML)

##### Yoko Kosaka, PhD^1^, Adam Lamble, MD^2^, Fei Huang, PhD^*3*^, Evan Lind, PhD^1^

###### ^1^Oregon Health & Science University, Portland, OR, USA; ^2^Seattle Children’s Hospital, Portland, OR, USA;^3^Janssen Pharmaceutical R&D, Spring House, PA, USA

####### **Correspondence:** Evan Lind (linde@ohsu.edu)

**Background**

Background: The success of immunotherapeutic checkpoint blockade in cancer has led to great interest in finding novel targets that play a pivotal role in immune responses. One such molecule is T cell immunoreceptor with Ig and ITIM domains (TIGIT), which has been shown to be inhibitory and expressed by nonresponsive and suppressive T cells in the tumor microenvironment.

**Methods**

Methods: In the present study, we investigate the role of TIGIT on immune suppression of T cell responses in bone marrow microenvironment of patients with AML. Bone marrow aspirates were subjected to T cell proliferation assays using stimulation though TCR with or without accompanying TIGIT blockade. Samples were also subjected to high parameter mass-spec based flow cytometry and both mutational and transcriptional profiling by deep sequencing and clinical parameters (age, sex, blast count, ELN risk stratification) were recorded.

**Results**

Results: Of 57 total samples tested, 24 (42.1%) showed a profound defect in T cell proliferation in response to anti-CD3 stimulation (<5% of T cells responding to stimulation). Of these 24 that showed the most functional impairment, 12 (50%) had at least a 2-fold and 6 (25%) at least a 5-fold increase in the frequency of dividing T cells with the addition of an anti-TIGIT blocking antibody.

**Conclusions**

Conclusions: These results indicate that in many samples, TIGIT blockade can partially overcome functional suppression of T cells in AML bone marrow, and suggest that TIGIT is involved in mediating immune defects in AML. A better understanding of the role of TIGIT in the AML microenvironment will provide insight in determining whether TIGIT blockade could represent an effective therapy in AML.

**Acknowledgements**

This work was also funded in part by generous support from the Leukemia and Lymphoma Society of America Beat AML project (PIs Brian Druker MD/Jeffrey Tyner PhD).

**Ethics Approval**

All human experiments are approved under IRB protocol #00004422 Marc Loriaux MD, PI.

#### P402 DuoBody-CD40x41BB conditionally enhances immune activation by crosslinking of CD40- and 4-1BB positive cells

##### Alexander Muik, PhD^1^, Friederike Gieseke^1^, Isil Altintas, PhD^2^, Saskia Burm^2^, Mustafa Diken^2^, Christian Grunwitz^2^, Sebastian Kreiter^2^, David Satijn, PhD^2^, Danita Schuurhuis^2^, Ozlem Tureci^2^, Ugur Sahin^2^, Esther Breij, PhD^2^

###### ^1^BioNTech AG, Mainz, Germany; ^2^Genmab B.V., Utrecht, Netherlands

####### **Correspondence:** Alexander Muik (Alexander.Muik@biontech.de)

**Background**

Immune checkpoint inhibitor antibodies that can (re-)activate anti-tumor immunity show great promise for the treatment of cancer. Similarly, therapeutic agents that boost anti-tumor immunity by direct activation of immunostimulatory molecules may provide clinical benefit. In this context, targeting tumor necrosis factor (TNF) receptor superfamily members, which deliver essential co-stimulatory activity for immune responses, gained attention. We hypothesized that simultaneous engagement of the T-cell co-stimulatory molecule 4-1BB and CD40 on antigen-presenting cells (APCs) using a bispecific antibody could be an elegant and potent mechanism to induce conditional activation of both CD40-positive immune cells and 4-1BB positive T cells.

**Methods**

DuoBody-CD40x4-1BB (GEN1042) is an IgG1 Fc-silenced bispecific antibody that was obtained by controlled Fab- arm exchange of humanized parental CD40- and 4-1BB-specific monoclonal antibodies. The binding characteristics and functional activity of DuoBody-CD40x4-1BB were analyzed in vitro using flow cytometry, cell-based reporter assay systems and primary human lymphocyte assays. To evaluate the capacity of DuoBody-CD40x4-1BB to induce proliferation of tumor-infiltrating lymphocytes (TILs) in the tumor microenvironment, ex vivo TIL expansion assays were conducted using freshly isolated human tumor specimen.

**Results**

DuoBody-CD40x4-1BB induced activation of both CD40 and 4-1BB intracellular signaling, which was dependent on simultaneous binding to CD40- and 4-1BB positive cells as measured by reporter assays. The monospecific control antibodies did not show agonist activity. This demonstrates that the bispecific antibody confers conditional activity upon receptor cross-linking. Using human primary T cells and monocyte-derived dendritic cells, both obtained from human healthy donor PBMCs, antigen-specific T-cell assays were conducted in vitro. DuoBody- CD40x4-1BB enhanced T-cell proliferation as well as concomitant pro-inflammatory cytokine secretion. Enhanced T-cell proliferation was again dependent on binding of DuoBody-CD40x4-1BB to both CD40 and 4-1BB. Importantly, DuoBody-CD40x4-1BB did not enhance proliferation of T cells that were not pre-activated by TCR stimulation. Furthermore, in ex vivo cultures of fresh human tumor tissue resections DuoBody-CD40x4-1BB increased the expansion of TILs up to 10-fold over control antibody treatment.

**Conclusions**

In summary, DuoBody-CD40x4-1BB is a bispecific antibody that crosslinks CD40 and 4-1BB positive cells, thereby inducing conditional stimulation and subsequently co-stimulatory activity. In the context of cancer, DuoBody-CD40x4-1BB can enhance anti-tumor immunity by (re-)activating tumor-specific T cells, either intratumorally or in the tumor-draining lymph nodes. The unique mechanism of action, its conditional agonist activity, distinguishes DuoBody-CD40x4-1BB from agonistic antibodies targeting CD40 or 4-1BB. Therefore, DuoBody-CD40x-4-1BB represents a novel therapeutic agent with potential for treatment of solid tumors.

**Ethics Approval**

The use of tumor tissue resections was approved by BioNTech AG’s Ethics Board, approval number 837.309.12 (8410-F).

#### P403 Preliminary results from a first-in-human phase 1 study of the CD40 agonist monoclonal antibody (mAb) CDX-1140

##### Rachel Sanborn, MD^1^, Michael S. Gordon, MD^2^, Mark O’Hara, MD^3^, Nina Bhardwaj, MD, PhD^4^, Yi He, PhD^5^, Tracey Rawls^5^, Tibor Keler, PhD^5^, Michael Yellin, MD^5^

###### ^1^Providence Portland Medical Center, Portland, OR, USA; ^2^Scottsdale Healthcare Hospital, Scottsdale, AZ, USA; ^3^Hospital of the University of PA, Philadelphia, PA, USA; ^4^Icahn School of Medicine Mt. Sinai, New York, NY, USA; ^5^Celldex Therapeutics, Inc., Hillsborough, NJ, USA

####### **Correspondence:** Michael S. Gordon (msgordon@u.arizona.edu)

**Background**

Agonist CD40 mAbs can mediate antitumor immunity through multiple mechanisms, including enhancing tumor antigen presentation, activation of tumoricidal macrophages, and direct growth inhibition/killing of CD40- expressing tumor cells. To fully exploit these mechanisms may require the mAb to be dosed at levels that provide significant tumor and tissue penetration, without dose-limiting-toxicities (DLT) from systemic CD40 activation. Our agonist CD40 mAb, CDX-1140, was selected based on its unique and linear dose-dependent in vitro and in vivo activity and is postulated will achieve maximum agonist activity at dose levels associated with good systemic exposure. CDX-1140 is a fully human IgG2 agonist anti-CD40 mAb that activates dendritic cells (DCs) and B cells in an Fc receptor independent manner and has potent antitumor activity against CD40-expressing cancer cells. In addition, CDX-1140 does not block the natural CD40-CD40L interaction; combination of CDX-1140 with added soluble CD40L is synergistic in the activation of immune cells suggesting a potential to enhance in vivo CD40L dependent immune responses. In toxicology studies, CDX-1140 demonstrated potent CD40-mediated pharmacological effects without significant toxicities.

**Methods**

A phase 1 dose-escalation study of CDX-1140 (CDX1140-01; NCT03329950) is underway in patients with advanced tumors who have exhausted standard-of-care treatment options. The primary endpoint is determining the safety profile and maximum tolerated dose. Secondary endpoints include pharmacokinetics, immunogenicity, clinical and biological outcome assessments. Baseline and on-study biopsies will be used to explore the pharmacodynamic effects of CDX-1140 in the tumor microenvironment (TME). The dose escalation (DE) portion evaluates CDX-1140, given every 4 weeks, at doses from 0.01 to 3 mg/kg; the first 2 cohorts are single-patient cohorts and all subsequent DE cohorts are conducted utilizing a 3+3 design. Tumor-specific expansion cohorts will further explore the activity of CDX-1140. This study will also evaluate CDX-1140 in combination with CDX-301 (rhFLT3L), a DC growth factor that markedly increases DC numbers, including the CD141+ subset which are critical to an antitumor immune response and are often scarce within the TME.

**Results**

To date, CDX-1140 cohorts at 0.01 (n=2), 0.03 (n=1), and 0.09 (n=3) mg/kg have been completed without any drug-related serious adverse events, infusion reactions, or DLTs reported. The only drug related toxicity has been grade 1 fatigue (n=2) . Expected pharmacodynamic effects, including transient, dose-dependent decreases in lymphocyte counts and dose-dependent increases in serum IL-12p40 and TNF-Alpha, have been observed.

**Conclusions**

The early data suggest that CDX-1140 has the expected immune activating and safety profile.

**Ethics Approval**

The study was approved by University of Pennsylvania, approval number 828733; Mount Sinai School of Medicine, approval number IRB-18-00213; Providence Health and Services, approval number 201700532 and Western Institutional Review Board, approval number 115925

#### P404 The discovery and characterization of PTZ-522 (ASP1951), a fully-human, high affinity agonistic anti-GITR tetravalent monospecific monoclonal antibody

##### Cynthia Seidel-Dugan, PhD^3^, Sonja Kleffel^1^, Sandra Abbott^1^, Heather Brodkin^1^, Daniel Hicklin^1^, Nels Nielson^2^, Christopher Nirschl^1^, Rebekah O'Donnell^1^, Andreas Salmeron^1^, Philipp Steiner, PhD^1^, Christopher Thomas^1^, William Winston^1^

###### ^1^Potenza Therapeutics, Inc, Cambridge, MA, USA; ^2^Adimab, LLC, Lebanon, NH, USA; ^3^Potenza Therapeutics, Cambridge, MA, USA

####### **Correspondence:** Cynthia Seidel-Dugan (cseideldugan@potenzatherapeutics.com)

**Background**

Multiple studies have demonstrated that tumors establish an immunosuppressive microenvironment (TME) to escape immune surveillance and promote tumor development. Tumor-infiltrating lymphocytes (TILs) become suppressed in the TME so their proliferative capacity and effector functions are impaired. Members of the TNF Receptor (TNFR) family and their ligands modulate the proliferation, differentiation, and activation of immune effector cells. Glucocorticoid-induced TNFR-related (GITR) is a receptor belonging to the TNFR family with costimulatory activity. In preclinical studies, GITR agonists increase effector T cell proliferation and function, and decrease the tumor infiltration, stability, and/or survival of Tregs, resulting in a more pro-inflammatory TME. In multiple syngeneic mouse tumor models, treatment with GITR agonists demonstrates compelling anti-tumor activity. Based on these promising preclinical data, a number of GITR agonist agents are being tested in the clinic.

**Methods**

Functional and structural studies have demonstrated that optimal activation of human GITR requires an adequate clustering of the receptor with trimeric GITR ligand (GITRL). Traditional bivalent agonistic antibodies are not as efficacious as trimeric GITRL and are expected to require FcR mediated cross-linking for full activity, which introduces potentially undesired FcR activation, cytokine release, and/or elimination of key effector cells expressing GITR. Potenza Therapeutics has identified PTZ-522 (also known as ASP1951), a novel, tetravalent monospecific (TM) anti-GITR agonist antibody designed to overcome these potential liabilities.

**Results**

PTZ-522 is a hinge-stabilized IgG4 antibody which binds with high affinity to human and cynomolgus monkey GITR. PTZ-522 has agonistic activity in engineered cell assays and primary T cells from peripheral blood of healthy donors. The TM-formatted antibody PTZ-522 is more active in cell assays than the same antibody in a bivalent format (522-IgG4) and has similar or greater activity than trimeric GITRL. Moreover, this activity was observed in the absence of any FcR cross linking. Analysis of GITR expression on T cells using human tumor samples as well as PBMCs demonstrates that GITR is more highly expressed on TILs, highlighting the different immune phenotype of cells found in the tumor microenvironment. The TM-formatted antibody PTZ-522 increases proliferation and inflammatory cytokine production by GITR expressing TILs. Increased activity in the TIL assay is also observed with the combination of PTZ-522 with a PD-1 inhibitor. Mechanistically, PTZ-522 binding to T cells results in rapid GITR activation and internalization not observed with bivalent antibodies.

**Conclusions**

These data support the continued development of PTZ-522 as a novel GITR agonist for the treatment of cancer.

#### P405 Agonistic IgM antibodies targeting immunostimulatory TNFRSF family members GITR and OX40 enhance immune responses beyond that of IgGs

##### Angus Sinclair, PhD, Dalya Rosner, PhD, Beatrice Wang, Tasnim Kothambawala, Ling Wang, Susan Calhoun, Avneesh Salini, Sachi Rahman, Ramesh Baliga, PhD, Bruce Keyt

###### IGM Biosciences, Mountain View, CA, USA

####### **Correspondence:** Angus Sinclair (angus.m.sinclair@gmail.com)

**Background**

Tumor necrosis factor receptor (TNFR) superfamily T cell immunostimulatory receptors OX40 and GITR require trimerization to induce agonistic signaling. Anti-OX40 and anti-GITR therapeutic IgG antibodies have demonstrated limited anti-tumor activity in the clinic, perhaps due to inefficient multimerization through FcγR engagement in the tumor microenvironment. We generated and evaluated the functional properties of agonistic anti-OX40 and anti- GITR IgM antibodies and have compared their activities to corresponding IgGs.

**Methods**

Agonistic anti-OX40 and anti-GITR IgG1 and IgM antibodies were generated using variable regions inserted into the same light chains and either the IgG or IgM heavy chain framework. Antibody binding was evaluated using antigen ELISAs or by flow cytometry with human T cells. Activated human T cells were used to evaluate antibody induced proliferation and cytokine release. Effects of these antibodies on regulatory T cell (Treg) suppression of T effector proliferation and cytokine release were measured.

**Results**

Agonistic anti-OX40 and anti-GITR IgM antibodies demonstrated superior binding potencies to recombinant antigens (>10 fold in molar comparisons) compared to IgG antibodies, which were amplified at low antigen density. Improved binding was due to the enhanced avidity of the multivalent IgMs compared to bivalent IgGs. As OX40 and GITR require trimerization with ligand or antibodies for function, the agonistic properties of the IgMs and IgGs were compared. In NF-kB-luciferase reporter assays driven by either GITR or OX40, a significant increase in potency was observed with the IgM in both EC50 and max activity compared to IgG in presence or absence of cross-linking agents. In primary human T cell activation assays, IgMs significantly enhanced T cell proliferation and cytokine secretion compared to IgGs. OX40 and GITR are also expressed on immunosuppressive Tregs. In co- culture of Tregs with T effector cells, IgM antibodies significantly reversed the suppressive activity of Tregs on T cell proliferation and inflammatory cytokine secretion, whereas IgGs had little to no effect.

**Conclusions**

We have discovered IgM antibodies significantly bind to and signal more efficiently through OX40 and GITR than corresponding IgGs, even in presence of cross linking agents. Efficient multimerization and agonism of OX40 and GITR with IgM antibodies may therefore enhance the anti-tumor immunostimulatory effects of immunotherapeutic antibodies targeting these T cell agonists.

**Acknowledgements**

We thank Sarah Wadsworth for her assistance with some of the studies.

#### P406 ALPN-202, a combined PD-L1/CTLA-4 antagonist and PD-L1-dependent CD28 T cell costimulator, elicits potent intratumoral T cell immunity superior to and differentiated from PD-L1 inhibitor monotherapy

##### Ryan Swanson, BS, Mark Maurer, BS, Chris Navas, BS, Chelsea Gudgeon, BS, Katherine Lewis, PhD, Martin Wolfson, BS, Sherri Mudri, BS, Kayla Susmilch, MS, Joe Kuijper, Stacey Dillon, PhD, Steven Levin, PhD, Kristine Swiderek, PhD, Stanford Peng, MD, PhD

###### Alpine Immune Sciences, Seattle, WA, USA

####### **Correspondence:** Ryan Swanson (ryan.swanson@alpineimmunesciences.com)

**Background**

ALPN-202 is a variant CD80 vIgD™-Fc fusion protein blocking the PD-L1 and CTLA-4 checkpoints while providing PD-L1-dependent T cell activation via CD28. This strategy delivers potent T cell costimulation, which is currently missing from checkpoint inhibitor only regimens, and may be critical for the generation of clinical anti- tumor responses, seeking to broadly improve cancer outcomes. ALPN-202 has previously demonstrated preclinical anti-tumor activity superior to PD-L1 inhibition, but the specific mechanism(s) of superiority remain unreported.

**Methods**

In a hPD-L1-transduced MC38 tumor model treated with ALPN-202 or durvalumab, an approved PD-L1 inhibitor, anti-tumor responses were evaluated by serial tumor volume measurements, and intratumoral immune responses were assessed by RNA-Seq, flow cytometry, and immunoSEQ TCR repertoire analysis (Adaptive Biotechnologies).

**Results**

Multiple doses of ALPN-202 elicited anti-tumor responses superior to durvalumab as judged by tumor volume measurements. Efficacy was importantly also observed with single ALPN-202 doses administered intraperitoneally or intratumorally. RNA-Seq and flow cytometric analyses of tumors revealed higher T cell, NK cell, macrophage, and dendritic cell markers after ALPN-202 treatment vs. durvalumab, along with higher T cell effector transcripts, including IL-2, IFN-gamma, granzyme B, and T-bet. TCR repertoire analysis demonstrated increased clonality and richness in response to ALPN-202, two characteristics previously not reported in response to PD-(L)1 or CTLA-4 inhibition alone.

**Conclusions**

ALPN-202 elicits intratumoral immune responses superior to PD-L1 inhibition alone, including T cell infiltration and T cell effector molecules. This suggests it may translate into clinical anti-tumor responses in cancers currently resistant to checkpoint inhibition alone and/or may improve outcomes in cancers when administered in combination with existing therapies. Ongoing studies seek to further define such potential and specific clinical indications and modalities to guide upcoming clinical trials.

#### P407 CTX-471, a novel agonistic antibody targeting CD137, eradicates very large tumors in vivo by selectively reprogramming the tumor microenvironment without causing hepatic toxicity

##### Ugur Eskiocak, PhD^1^, Wilson Guzman, BS^1^, Nora Zizlsperger, PhD^1^, Benjamin Wolf^1^, Christine Cummings^1^, Thomas Daly^1^, Puru Nanjappa^1^, Lauren Milling^1^, Xianzhe Wang^1^, Lucy Liu^1^, Samantha Ottinger^1^, Jason Lajoie^1^, Michael Schmidt^1^, Robert Tighe, BS^2^

###### ^1^Compass Therapeutics, Cambridge, MA, USA; ^2^Compass Therapeutics LLC, Cambridge, MA, USA

####### **Correspondence:** Robert Tighe (robert.tighe@compasstherapeutics.com)

**Background**

CD137 (4-1BB) is a member of the TNFR superfamily that provides costimulatory signals to activated cytotoxic lymphocytes. Agonistic antibodies against CD137 have shown promising therapeutic activity in mouse tumor models. However, hepatic toxicity has been observed in animals and humans with a few anti-CD137 antibodies [1,2]. Recent advances in our understanding of TNFR agonist antibodies implicate epitope, affinity, and IgG subclass as important contributors to function [3,4]. Here we describe the preclinical characterization of CTX-471, a fully human IgG4 agonist of CD137 with a differentiated pharmacology and toxicology profile.

**Methods**

CTX-471 was identified based on epitope binning and antigen-binding assays. The in vitro bioactivity of CTX-471 was measured in a human IFN-γ release assay. The in vivo efficacy of CTX-471 was assessed in multiple syngeneic mouse tumor models that included various mechanistic endpoints: FACS analysis of TILs, effector cell depletion, tumor histology, and Fc receptor profiling. The efficacy of CTX-471 was further evaluated in mice bearing very large (~500 mm3) CT26 tumors. Finally, the toxicity profile of CTX-471 was evaluated in mice and cynomolgus monkeys.

**Results**

CTX-471 binds to a unique epitope shared by human, cynomolgus monkey, and mouse CD137. In vitro, CTX-471 increased IFN-γ production by human T cells in an FcγR-dependent manner, displaying an intermediate level of activity between two clinical-stage anti-CD137 antibodies. In Vivo, CTX-471 exhibited curative monotherapy activity in CT26, A20, and EMT-6 models. When compared to known anti-CD137, OX-40, PD-1, PD-L1, and CTLA-4 antibodies, only an affinity-optimized version of CTX-471 showed the ability to eradicate very large tumors. All mice cured by CTX-471 rejected tumors upon rechallenge. CTX-471 profoundly reprogramed the TME, leading to an influx of inflammatory cells, decreased T cell exhaustion, Treg depletion, and TAM modulation, while having very little effect on the peripheral immune system. Tumor models with abundant expression of FcγR’s responded more strongly to CTX-471 treatment, and Fc silencing mutations attenuated efficacy. In mice and monkeys, extremely high doses of CTX-471 (up to 100 mg/kg weekly for 4 weeks) were well-tolerated, with no signs of hepatic toxicity.

**Conclusions**

CTX-471 displays a favorable and well-differentiated efficacy-safety profile that is attributed to a unique epitope, optimized affinity, and FcγR-dependent activity. To our knowledge, CTX-471’s level of monotherapy efficacy against very large tumors is unprecedented for an IO antibody. IND-enabling toxicology studies are underway, and a Phase 1 trial is planned for the first-half of 2019.

**References**

1. Bartkowiak T, Jaiswal AR, Ager CR, et al. Activation of 4-1BB on liver myeloid cells triggers hepatitis via an interleukin-27–dependent pathway. Clinical Cancer Research. 2018 Apr;24(5):1138–51.

2. Segal NH, Logan TF, Hodi FS, et al. Results from an integrated safety analysis of urelumab, an agonist Anti- CD137 monoclonal antibody. Clinical Cancer Research. 2016;23(8):1929–36.

3. Yu X, Chan HTC, Orr CM, et al. Complex interplay between epitope specificity and isotype dictates the biological activity of anti-human CD40 antibodies. Cancer Cell. 2018;33(4):664-675.e4. 4. Chodorge M, Züger S, Stirnimann C, et al. A series of fas receptor agonist antibodies that demonstrate an inverse correlation between affinity and potency. Cell Death Differ. 2012;19(7):1187-95.

**Ethics Approval**

All animal studies were approved by Compass Therapeutics Institutional Animal Care and Use Committee under protocol CTX-016-01.

#### P408 Preclinical identification of the pharmacologically active dose range of the tumor targeted 4-1BB agonist MP0310 based on tumor regression, receptor occupancy and CD8 T lymphocyte expansion

##### Elmar Vom Baur, PhD, MBA, MEng, Ivana Tosevski, PhD, Laurent Juglair, MSc, Alexander Link, PhD, Guy Lemaillet, Heïdi Poulet, Christian Reichen, PhD, Patricia Schildknecht, MSc, Joanna Taylor, MSc, Alexander Titz, MSc, Waleed Ali, Doris Schaible, Mirela Matzner, Christof Zitt, PhD, Jörg Herbst, PhD, DABT, Keith Dawson, PhD, Julia Hepp, PhD, Dan Snell, PhD, Michael T. Stumpp, PhD, Victor Levitsky, MD, PhD, Hong Ji, MD, PhD, Ivana Tosevski, PhD

###### Molecular Partners AG, Schlieren, Switzerland

###### **Correspondence:** Elmar Vom Baur (elmar.vombaur@molecularpartners.com)

**Background**

In animal models, agonistic antibodies targeting the T cell costimulatory receptor 4-1BB (CD137) have shown promise as anti-tumor agents, but clinical studies have shown only limited signs of efficacy as well as dose-limiting hepatotoxicity with one of the candidates. To avoid systemic toxicities and to direct immune activation to the tumor, we have generated the tumor-targeted 4-1BB agonist MP0310. MP0310 is a multi-domain DARPin® molecule comprising domains binding to 4-1BB, fibroblast activation protein (FAP), and human serum albumin, the latter for half-life extension. FAP binding targets MP0310 activity to tumors as FAP is highly expressed in many solid tumors and crucially, activation of 4-1BB by MP0310 is dependent on FAP-mediated clustering of 4-1BB. Previously we have shown, in vitro and in vivo, that MP0310 is at least as potent as the agonistic 4-1BB antibodies and, in contrast to the antibodies, does not induce hepatotoxicity or exacerbate graft versus host disease in humanized mouse models. Also, no systemic T cell activation has been observed in cynomolgus monkeys. The present study, in support of the clinical development of MP0310, is to establish the pharmacologically active dose range of MP0310 in a mouse model based on parameters such as receptor occupancy, CD8+ T cell expansion and anti-tumor activity.

**Methods**

NSG mice were implanted subcutaneously with HT-29 tumor cells and subsequently were injected with human PBMCs. Mice were then treated with a suboptimal dose of a tumor associated antigen binding T cell engager and with a variant of MP0310 containing a mouse FAP binding domain (mMP0310) at doses from 0.0128 to 40mg/kg. Tumor volume and potential biomarkers of T cell activation were measured.

**Results**

mMP0310 strongly increased intra-tumoral CD8 T cell expansion in a dose-dependent manner compared to monotherapy with the T cell engager. Maximal increases in intra-tumoral CD8 T cell numbers and the ratio of CD8 versus CD4 cells correlated with 100% receptor occupancy (RO) reached at a dose of 1.6mg/kg. Only marginal activity on T cells was seen at a dose of 0.0128mg/kg correlating with a 4-1BB RO level below 10%. In addition, mMP0310 enhanced tumor regression induced by the T cell engager in a manner suggesting dose-dependency starting at 0.32mg/kg

**Conclusions**

The novel FAP-targeted 4-1BB agonist mMP0310 has been shown to enhance T cell activation and antitumor activity without producing toxicity. Dose dependency of pharmacodynamic activities related to 4-1BB RO has been demonstrated and biomarkers for clinical development have been identified

#### P409 Pharmacodynamic activity of MEDI1873, a Glucocorticoid-Induced tumor necrosis factor family-related protein (GITR) agonist molecule, administered intravenously to patients with advanced solid tumors

##### Nicholas Durham, PhD^2^, Nathan Standifer, PhD^2^, Jennifer Cann, PhD^2^, Christopher Morehouse, MD^2^, Li Yan^2^, Kristina Kovacina^2^, Xu Liu^2^, Jia Li^2^, Yuling Wu^2^, Katie Streicher, PhD^2^, Paolo Vicini^2^, Ayesh Perera^2^, Rakesh Kumar, PhD^2^, Raid Aljumaily, MD^3^, Aung Naing, MD, FACP^4^, Ashish Chintakuntlawar, MBBS, PhD^5^, Naiyer Rizvi, MD^6^, Helen Ross, MD^5^, Michael Gordon, MD^7^, Jeffrey Infante^8^, Crystal Denlinger^9^, Ani Balmanoukian, MD^10^

###### ^1^Ashfield Healthcare; ^2^MedImmune, Gaithersburg, MD, USA; ^3^Oklahoma University College of Medicine, Oklahoma City, OK, USA; ^4^University of Texas, Houston, TX, USA; ^5^Mayo Clinic, Rochester, MN, USA; ^6^Columbia University Medical Center, New York, NY, USA; ^7^HonorHealth, Scottsdale, AZ, USA; ^8^Sarah Cannon Research Institute, Spring House, PA, USA; ^9^Fox Chase Cancer Center, Philadelphia, PA, USA; ^10^The Angeles Clinic, Los Angeles, CA, USA

####### **Correspondence:** Nicholas Durham (durhamn@medimmune.com)

**Background**

MEDI1873 is a novel GITR-ligand/IgG1 agonist fusion protein that binds to the co-stimulatory molecule GITR, which is upregulated on activated T cells. This Phase 1 study (NCT02583165) evaluated safety, pharmacokinetics, pharmacodynamics, and preliminary antitumor activity in patients with advanced solid tumors.

**Methods**

MEDI1873 was administered IV Q2W in sequential dose escalation (DE) cohorts: 2 single patient cohorts (1.5 and 3 mg), followed by six 3+3 DE cohorts (7.5, 25, 75, 250, 500 and 750 mg). Antitumor response was assessed using RECIST v 1.1. Pharmacodynamic cohorts of patients with non-small cell lung cancer, head and neck squamous cell carcinoma (HNSCC) or colorectal cancer receiving 75 or 250 mg were evaluated for intratumoral CD8, FOXP3, GITR and tumoral PD-L1 expression by immunohistochemistry using matched biopsies from pretreatment and day 29. Peripheral blood from all patients was monitored for type-2 IFN cytokine levels, gene expression, and lymphocyte phenotype by flow cytometry up to day 43.

**Results**

As of 1 March 2018, 40 patients have been dosed in the DE (28) and pharmacodynamic (12) cohorts. MEDI1873 elimination half-life was ~2.0 days. MEDI1873 pharmacokinetics was dose-proportional over a range of 1.5 to 500 mg. Antidrug antibodies were rare and had minimal impact on pharmacokinetics. MEDI1873 engaged GITR as evidenced by >50% reductions in peripheral GITR-expressing memory CD4+ T cells using a competitive target engagement assay. The duration of GITR+ T-cell suppression correlated with MEDI1873 dose. At doses ≥75 mg, peripheral IFNγ, IP-10, I-TAC and MIG were elevated on days 2 and 3. This was followed by increased Ki67+CD4+ T cells on day 15. Additionally, patients with high baseline GITR levels (≥ median) had a sustained elevation of Ki67+CD4+ T cells to Day 43. Of 8 patients with evaluable paired tumor biopsies, one patient with HPV+ HNSCC and the highest baseline GITR on CD4+ T cells showed a 2x increase in intratumoral CD8+ and tumoral PD-L1+ cells. Intratumorally, MEDI1873 induced a ≥25% reduction in GITR+/FOXP3+ T cells in 5 of 5 evaluable patients. Patients with prolonged stable disease (≥40 weeks) had an average 2x elevation in Ki67+CD4+ peripheral T cells compared to all other patients.

**Conclusions**

MEDI1873 engages GITR on circulating blood cells resulting in increased peripheral IFNγ production and CD4+memory T-cell proliferation coupled with a decrease in intratumoral GITR+/FOXP3+ cells. These pharmacodynamic effects are consistent with the compound’s mechanism of action and may be enhanced in patients with high baseline GITR expression.

**Trial Registration**

ClinicalTrials.gov [NCT02583165]

**Ethics Approval**

Multicenter study conducted at 11 sites:(1) Pinnacle Oncology Hematology, 9055 E Del Camino Dr., Scottsdale, AZ 85358, USA [PI: Michael Gordon; IRB Registration No. IRB00002349](2) Mayo Clinic, AZ, 5777 E. Mayo Boulevard, Phoenix, AZ 85054, USA [PI: Helen Ross; IRB Registration No. IRB00000020](3) The Angeles Clinic and Research Institute, 11818 Wilshire Boulevard, Los Angeles, CA 90025, USA [PI: Ani Balmanoukian; IRB Registration No. IRB00002349](4) Mayo Clinic, MN, 200 First Street SW, Rochester, MN 55905, USA [PI: Asish Chintakuntlawar; IRB Registration No. IRB00000020](5) H Lee Moffitt Cancer Center and Research Institute, 12902 Magnolia Drive, Tampa, FL 33612, USA [PI: Scott Antonia; IRB Registration No. IRB00000790](6) Sarah Cannon Research Institute, 250 25th Avenue North, Nashville, TN 37203, USA [PI: Howard Burris; IRB Registration No. IRB00001035](7) Columbia University Medical Center, 161 Fort Washington Avenue, Herbert Irving Pavilion Mezzanine, New York, NY 10032, USA [PI: Richard Carvajal; IRB Registration No. IRB00006882](8) Greenville Health System, 120 Dillon Drive, Spartanburg, SC 29307, USA [PI: Ki Chung; IRB Registration No. IRB00002227](9) Fox Chase Cancer Center, 333 Cottman Avenue, Philadelphia, PA 19111, USA [PI: Crystal Denlinger; IRB Registration No. IRB00002349](10) Oklahoma University, 1000 Stanton L Young Blvd, Oklahoma City, OK 73117, USA [PI: Raid Aljumaily; IRB Registration No. IRB00001035](11) MD Anderson, 1515 Holcombe Blvd, Houston, TX 77030, USA [PI: Aung Naing; IRB Registration No. IRB00002203]

### Cytokines in Anti-Tumor Immunity

#### P410 MV-626, a potent and selective inhibitor of ENPP1 enhances STING activation and augments T-cell mediated anti-tumor activity in vivo

##### Jason Baird, PhD^1^, Gregory Dietsch, PhD, DABT^2^, Vincent Florio, PhD^2^, Michael Gallatin, PhD^2^, Clayton Knox, MD^2^, Joshua Odingo, PhD^2^, Marka Crittenden, MD, PhD^1^, Michael J. Gough, PhD^1^

###### ^1^EACRI Providence Portland Medical Center, Portland, OR, USA; ^2^Mavupharma, Kirkland, WA, USA

####### **Correspondence:** Michael J. Gough (michael.gough@providence.org)

**Background**

STING is an endogenous sensor of cGAMP, which is synthesized by cGAS following detection of cytoplasmic DNA. STING activation leads to interferon production and activation of inflammatory pathways that facilitate cytolytic T cell priming. STING agonists administered intratumorally show potent anti-tumor efficacy in a range of preclinical models; several agonists are in clinical development. Radiation therapy also increases cytoplasmic DNA levels in cancer cells, resulting in STING activation and secretion of inflammatory cytokines. Ectonucleotide pyrophosphatase/phosphodiesterase 1 (ENPP1) is the phosphodiesterase that negatively regulates STING by hydrolyzing cGAMP. MV-626, a highly potent and selective ENPP1 inhibitor with 100% oral bioavailability in rats and mice, blocks cGAMP hydrolysis and increases STING activation in cells where cGAS is active. We hypothesize that by conditionally enhancing STING activation, ENPP1 inhibitors will facilitate development of anti-tumor cellular immune responses, particularly following radiation therapy.

**Methods**

The effects of ENPP1 inhibition on STING activation using cGAMP or DNA treatment of cells were assessed. Panc02-SIY tumors were implanted in C57BL/6 mice and randomized to receive 20Gy CT-guided radiation therapy, 5 daily ip doses of MV-626, or both MV-626 and radiation. Mice were followed for outcome, tumor antigen specific T cell responses and changes in the tumor immune environment. Additional studies were conducted in mice bearing MC38 tumors.

**Results**

In vitro, MV-626 blocks ENPP1-mediated hydrolysis of cGAMP and enhances STING activation by DNA-mediated cGAS activation or exogenous cGAMP. Therapeutic doses of MV-626 were well tolerated in mice, with no evidence of toxicity or clinically-significant increases in systemic cytokine levels. Systemic administration of MV- 626 monotherapy caused tumor growth delay. MV-626 combined with radiation therapy significantly increased overall survival, and most animals achieved durable tumor cures. Additional studies in the MC38 model confirmed MV-626 activity. Studies characterizing effects of MV-626 in the tumor microenvironment are underway.

**Conclusions**

These data demonstrate that a potent, selective ENPP1 inhibitor augments STING activation and enhances immune responses to tumors. We demonstrate for the first time that, in combination with radiation therapy, ENPP1 inhibition improves outcomes and cures tumors in preclinical models through changes in the tumor immune environment. These translational studies represent a novel approach to enhancing tumor directed immune response following radiation, and provide a foundation for clinical development of an ENPP1 inhibitor as a cancer immunotherapy.

#### P411 An IL15/IL15Rα heterodimeric Fc-fusion engineered for reduced potency demonstrates an optimal balance of in vivo activity and exposure

##### Matthew Bernett, PhD^1^, Rajat Varma, PhD^1^, Christine Bonzon, PhD^1^, Liz Bogaert, PhD^1^, Rumana Rashid, PhD^1^, Ke Liu, PhD^1^, Irene Leung, PhD^1^, Suzanne Schubbert, PhD^1^, Sung-Hyung Lee, PhD^1^, Daniel Kirouac, PhD^2^, Fei Hua, PhD^2^, Nicole Rodriguez, PhD^1^, Yoon Kim, PhD^1^, Kendra Avery, PhD^1^, Connie Ardila^1^, Nargess Hassanzadeh- Kiabi, PhD^1^, Umesh Muchhal, PhD^1^, Seung Chu, PhD^1^, Gregory Moore, PhD^1^, John R. Desjarlais^1^

###### ^1^Xencor Inc., Monrovia, CA, USA; ^2^Applied BioMath, LLC, Oakland, CA, USA

####### **Correspondence:** John R. Desjarlais (jrd@xencor.com)

**Background**

IL15 and IL2 are similar cytokines that bind to the IL2Rβ/γc receptor complex and induce the proliferation of lymphocytes. Their therapeutic potential has been well established in animal models and human trials. As potential drugs, both IL2 and IL15 are extremely potent and suffer from low tolerability and very fast clearance that limits therapeutic window. To engineer a more druggable version of IL15, we created various IL15/IL15Rα heterodimeric Fc-fusions (IL15/IL15Rα-Het-Fc) with reduced potency to improve tolerability, slow receptor-mediated clearance, and prolong half-life.

**Methods**

We engineered IL15/IL15Rα-Het-Fc by fusing IL15 to one side of a heterodimeric Fc, and the sushi domain of IL15Rα to the other. Fc-fusions were tuned for optimal activity by engineering amino acid substitutions in IL15 - at the IL2Rβ or γc interface - that reduced in vitro potency. In vitro proliferation of lymphocytes in normal human PBMCs was monitored by counting Ki67+ cells after incubation with Fc-fusions for 4 days and by measuring signaling in a STAT5 phosphorylation assay. In vivo activity was evaluated using a huPBMC-NSG mouse model by measuring the extent of human leukocyte engraftment by flow cytometry and IFNγ. Tolerability, immune stimulation, and pharmacokinetics were evaluated in non-human primates (NHP). A computational PK/PD model was developed and trained on available data to quantify relationships between affinity, dose, and biological activity.

**Results**

IL15/IL15Rα-Het-Fc were produced with good yield and purity. The Fc-fusions enhanced proliferation of CD8+ T and NK cells in vitro. Variants with substitutions at the IL2Rβ and/or γc interface reduced potency up to ~700-fold compared to wild-type IL15/IL15Rα-Het-Fc. Treatment of huPBMC-NSG mice with IL15/IL15Rα-Het-Fc promoted enhanced T cell engraftment and elevated IFNγ. NHP studies indicated half-lives of several days for potency-reduced IL15/IL15Rα-Het-Fc, which are significantly longer than the <1 hr half-life of IL15. In both in vivo settings, a marked inverse correlation of pharmacodynamics and clearance was observed, with reduced potency variants allowing higher, more tolerated doses and enhanced lymphocyte proliferation due to more sustained exposure. Our mechanism-based PK/PD model was used to predict optimal drug affinities, balancing potency vs. target-mediated clearance, and will be used to facilitate prediction of human PK/PD and regimen design. A lead candidate XmAb24306 was selected based on combined experimental observations and modeling predictions, and has been selected for clinical development.

**Conclusions**

Multiple IL15/IL15Rα heterodimeric Fc-fusions were engineered for reduced potency and evaluated in vitro and in vivo. We identified a variant, named XmAb24306, that optimally balanced potency and exposure.

#### P412 Tumor cell-intrinsic defects in STING pathway signaling

##### Blake Flood, BS^1^, Leticia Corrales^2^, Thomas Gajewski, MD, PhD^1^

###### ^1^University of Chicago, Chicago, IL, USA; ^2^Aduro, Berkeley, CA, USA

####### **Correspondence:** Blake Flood (blakeflood@uchicago.edu)

**Background**

Our laboratory has previously shown that immunogenic tumors spontaneously activate the innate immune system through the STING pathway. The STING pathway senses cytosolic DNA, which activates a signal transduction pathway culminating in phosphorylated IRF3 that translocates to the nucleus where it acts as a transcription factor to induce several genes including IFN-β. STING signaling and IFN-β receptor signaling in tumor-infiltrating immune cells, in turn, are required for optimal priming of CD8+ T cells against tumor antigens. Based on this notion, STING agonists have been pursued as a pharmacologic approach to activate the pathway. However, whether tumor cells themselves also can experience STING pathway activation through to IFN-β production has been unclear.

**Methods**

We stimulated various cell populations present in the tumor microenvironment as well as multiple tumor cell lines with STING agonists to test their ability produce IFN-β, and analyzed each step in STING pathway signaling. Further biochemical techniques including Western blotting and intracellular immunofluorescence were used to carefully analyze each step of the STING pathway in tumor cells or controls.

**Results**

We observed that tumor cells themselves usually fail to produce IFN-β in response to STING agonists or cytoplasmic DNA, arguing that loss of activation of this pathway might occur regularly as a component of oncogenesis. Surprisingly, we found that most tumor cells retain expression of each gene in the STING pathway, and that STING signal transduction was intact up to and including nuclear translocation of IRF3 in most instances. However, ChIP assays demonstrated that IRF3 was unable to bind the IFN-β promoter but could still bind the promoters of other genes. B16 melanoma cells, in particular, demonstrated a concurrent deficiency in NF-κB signaling downstream of STING pathway activation.

**Conclusions**

These results suggest that defective IRF3 DNA binding to the IFN-β locus may be a frequent alteration in cancer. Uncovering the detailed molecular mechanism of this effect could lead to new therapeutic interventions to restore the STING pathway in cancer cells.

#### P413 The FAP-IL2v immunocytokine is a versatile combination partner for cancer immunotherapy

##### Valeria Nicolini^1^, Inja Waldhauer^1^, Anne Freimoser-Grundschober^1^, Federica Cavallo^2^, Sara Colombetti^1^, Marina Bacac^1^, Gonzalo Acuna^1^, Jehad Charo, PhD^1^, Stefan Evers, PhD^1^, Volker Teichgraeber, MD^1^, Pablo Umana, PhD^1^, Christian Klein, Dr rer nat^1^

###### ^1^Roche Innovation Center Zurich, Schlieren, Switzerland; ^2^University of Turin, Turin, Italy; ^3^ROCHE Innovation Center Zurichoche, Schlieren, Switzerland

####### **Correspondence:** Christian Klein (christian.klein.ck1@roche.com)

**Background**

FAP-IL2v (RG7461) is a novel FAP-targeted immunocytokine based on a novel IL-2 variant (IL2v) with abolished binding to CD25 (IL2Ra) to overcome preferential expansion of Tregs, activation induced cell death and to reduce IL-2 toxicities due to CD25 binding. Binding of FAP-IL2v to the intermediate affinity IL-2Rbg heterodimer is retained resulting in induction of activation and expansion of immune cells, particularly NK cells and cytotoxic CD8 T-cells. These properties make FAP-IL2v a promising partner for combination with checkpoint inhibitors, ADCC- competent/enhanced therapeutic and T-cell bispecific antibodies (TCBs).

**Methods**

muFAP-IL2v, a murinized surrogate of FAP-IL2v, was tested in combination with muPD-L1 and muCD40 specific surrogate antibodies and the T-cell bispecific muCEA-TCB surrogate in syngeneic orthotopic pancreatic Panc02 or Panc02-CEA models, in C57BL/6 or human CEA transgenic C57BL/6 mice, respectively. The combination of muFAP-IL2v with a anti-ratHER2 muIgG2a antibody was evaluated in the fully immunocompetent BALB-neuT genetically engineered mouse model for breast cancer, whereas the combination of FAP-IL2v with the glycoengineered Type II anti-CD20 antibody obinutuzumab was evaluated in the WSU-DLCL2 xenograft model in hCD16 transgenic Scid mice.

**Results**

In the pancreatic orthotopic Panc02 model in C57BL/6 mice, FAP-IL2v can boost the activation of pre-existing antigen specific T-cells in combination with anti-PD-L1 checkpoint inhibition. In the same model FAP-IL2v further enhances the efficacy of PD-L1 checkpoint inhibition when combined with an agonistic CD40 antibody resulting in long term survival in the majority of animals, and in the induction of immunological memory as evidenced by protection from tumor cell re-challenge. Furthermore, FAP-IL2v is able to enhance the activity of 1) an ADCC- competent HER2 antibody in the BALB-neuT genetically engineered mouse model and 2) the ADCC-enhanced CD20 antibody obinutuzumab in the aggressive non-Hodgkin’s lymphoma model WSU-DLCL2 in human CD16 transgenic SCID mice. Finally, the activity of the T-cell bispecific antibody CEA-TCB was enhanced by combination with FAP-IL2v in the syngeneic pancreatic orthotopic Panc02 model stably expressing CEA in human CEA transgenic C57BL/6 mice.

**Conclusions**

The presented efficacy studies support the role of the FAP-targeted immunocytokine FAP-IL2v as a versatile combination partner for cancer immunotherapy and serve to inform the selection of combination partners for clinical studies. Particularly, they demonstrate FAP-IL2v's potential in combination with the PD-L1 checkpoint inhibitor atezolizumab, ADCC-competent antibodies e.g. trastuzumab, cetuximab or obinutuzumab, and T-cell bispecific antibodies such as CEA-TCB. Based on these data FAP-IL2v is currently being tested in Phase 1b clinical trials in combination with atezolizumab, trastuzumab and cetuximab.

#### P414 Efficacy of anti-PD-L1/IL-15 fusion protein in multiple tumor models

##### Stella Martomo, PhD, Dan Lu, MA, Zhanna Polonskaya, Xenia Luna, Kevin McCracken, Jeegar Patel

###### Kadmon Corporation, New York, NY, USA

####### **Correspondence:** Jeegar Patel (Jeegar.patel@kadmon.com)

**Background**

Therapeutic antibodies targeting immune checkpoint inhibitors such as PD-1/PD-L1 effectively expand and reactivate T cells in patients, leading to durable objective response rates in select cancers. However, a substantial number of patients fail to respond or become resistant to these therapies. Thus, combination therapies which include these agents as well as therapies targeting alternative effector cell types are needed. IL-15 promotes survival and cytotoxicity of both CD8 T and NK cells in the absence of the concomitant expansion of Treg population, making it a strong candidate for immunotherapy. Direct administration of IL-15 proved to be clinically challenging; durability of responses likely hindered by the short half-life and toxicity. To capitalize on the anti-tumor potential of IL-15, we generated a therapeutic fusion protein (KD033), combining a proprietary high affinity human-PD-L1 antibody (or mouse-PD-L1 surrogate antibody (KD033-surrogate)) with human IL-15. Initial assessment of this fusion antibody showed enhanced tolerability relative to a non-targeted IL-15 fusion antibody and potent anti-tumor activity.

**Methods**

Mouse syngeneic tumors were grown to 100 mm3 prior to a single IV administration of KD033-surrogate. Immune cell activation in cynomolgus monkeys was assessed following IV administration of KD033 (Day 1 and 15). Where applicable, tumor volumes were measured and immune cell infiltration and modulation was evaluated by immunohistochemistry, flow cytometry and Luminex.

**Results**

To assess broad anti-tumor potential of our molecule, single dose of KD033-surrogate was tested against a panel of 12 murine syngeneic tumors. Pronounced tumor growth inhibition was observed in multiple tumor types. In CT-26, colorectal tumor model, KD033-surrogate treatment achieved complete tumor regression in multiple animals, and consistent with generation of immune memory, tumors in these animals failed to regrow following CT-26 re- challenge. Interestingly, KD033-surrogate demonstrated synergistic response when co-administered with anti-PD-1 antibody, suggesting that KD033 could be effectively combined with other checkpoint modulators. Supporting the IL-15-dependent mechanism of action, KD033 (in monkeys) or KD033-surrogate (in mice) increased peripheral blood CD8, NK, NKT and/or gamma delta T (CD3+CD4-CD8-) cells. Additionally, an increase in tumor CD8 cells was observed in mice treated with KD033-surrogate compared to the non-targeted IL-15 fusion antibody.

**Conclusions**

KD033 treatment led to a robust activation of multiple effector cell types associated with a potent and durable anti-tumor activity. Based on the therapeutic activity and improved safety of the fusion protein, Kadmon is developing KD033 with the aim of clinical testing in 2019.

#### P415 Preclinical characterization of IL-2 Superkines engineered with biased CD8+ T cell stimulating properties

##### Fahar Merchant, PhD^1^, Shafique Fidai^1^, Aaron Ring^2^

###### ^1^Medicenna Therapeutics Inc., Toronto, Canada; ^2^Yale University School of Medicine, New Haven, CT, USA

####### **Correspondence:** Fahar Merchant (fmerchant@medicenna.com)

**Background**

Interleukin-2 (IL-2) is a cytokine immunotherapy approved by the FDA in 1992 that shows rare, but dramatic activity in metastatic renal cell carcinoma and melanoma. However, IL-2 therapy is hampered by limited efficacy, severe toxicities, and a short circulating half-life that necessitates frequent administration. These limitations may be overcome by engineering IL-2 variants with extended half-life and decreased reliance on the IL-2 accessory receptor CD25 that is believed to mediate toxicity and unwanted stimulation of Tregs.

**Methods**

A series of novel IL-2 ‘Superkines’ were engineered with biased potency towards the intermediate affinity IL-2 receptor (heterodimer of CD122 and CD132) and fused to a low-effector function Fc moiety for extended serum half-life. These Superkines were assayed for in vitro signaling potency on IL-2 receptor reporter cells lines and human peripheral blood mononuclear cells (PBMCs). Selected muteins were evaluated for their efficacy in syngeneic mouse models and for their in vivo PK and safety.

**Results**

A novel Fc-fused IL-2 mutein, MDNA109-Fc, was found to have a unique biased activation profile for cells expressing the intermediate affinity receptor, through a unique mechanism of action involving >1000 times increased affinity for CD122 vs. wild-type IL-2, while having similar affinity for CD25. This change improved IL-2 receptor dimerization in human cell lines and greatly enhanced phospho-STAT5 signaling and proliferation of CD8 lymphocytes vs. Tregs, leading to a 43-fold increase in potency at stimulating CD8+ T cells vs. wild-type IL-2. MDNA109-Fc also had improved potency on CD4+ Foxp3- T cells and NK cells. In vivo, this potency bias translates to an increased splenic CD8/Treg ratio. In addition, MDNA109-Fc demonstrated improved tumor growth inhibition over wild-type IL-2 in the aggressive B16F10 melanoma model. MDNA109-Fc was characterized at multiple doses and with several administration methods, demonstrating a greatly extended serum half-life that enabled a semiweekly to weekly subcutaneous dosing schedule in mice, paired with a good safety profile in vivo.

**Conclusions**

MDNA109-Fc is an improved interleukin-2 agent with a unique biased activation profile targeting effector versus immunosuppressive immune cells, and improved efficacy in a melanoma model. Unlike other next-generation IL-2 molecules in development, MDNA109-Fc specifically targets CD122, resulting in potent activation of effector T cells relative to Treg. MDNA109-Fc could improve the therapeutic potential of an effective, but limited use IL-2 immunotherapy by improving its efficacy, safety, and dosing convenience, a profile that may synergize well with immune checkpoint therapy.

#### P416 Short-course IL-15 given as a continuous infusion leads to massive expansion of NK cells: Implications for combination therapy with anti-tumor antibodies

##### Milos Miljkovic, MD, MSc^1^, Sigrid Dubois, PhD^1^, Thomas Fleisher, MD^2^, Jennifer Albert, RN^1^, Thomas Waldmann, MD^1^, Kevin C. Conlon, MD^1^

###### ^1^National Cancer Institute, Bethesda, MD, USA; ^2^NIH Clinical Center, Bethesda, MD, USA

####### **Correspondence:** Kevin C. Conlon (conlonkc@mail.nih.gov)

**Background**

Successful development of cytokines as immunotherapeutics for the treatment of cancer requires defining the optimal treatment regimen[1]. Post-infusional reactions limited dose escalation and immune activation in the first- in-human clinical trial of recombinant human IL-15 (rhIL-15) given as a 30-minute intravenous bolus (IVB)[2].

Ten-day treatment schedules of subcutaneous injection (SC) and continuous intravenous infusion (CIV-10) were better tolerated at 2 mcg/kg, with the CIV-10 schedule producing noteworthy increases in CD8 lymphocytes and NK cells[3, 4]. We report the results of the 5-day (CIV-5) rhIL-15 regimen, with a safety profile and stimulation of effector cells comparable to CIV-10, dosed up to 5 mcg/kg without dose-limiting toxicities.

**Methods**

Eleven patients were treated at 3 (n=4), 4 (n=3), and 5 mcg/kg/day (n=4, Table 1) with the CIV-5 regimen in a standard phase I dose-escalation study of rhIL-15 for patients with refractory metastatic cancers.

**Results**

There were no dose-limiting toxicities, but two patients did not complete cycle 1 for reasons unrelated to rhIL-15 (NSAID-induced SIADH and infectious gastroenteritis). The most common adverse events were fever, chills, fatigue, nausea, transient liver function test abnormalities, anemia, and thrombocytopenia (Tables 2-3). The best response was stable disease. Impressive expansion of NK cells was seen at all dose levels (21 to 44-fold, mean 33- fold) as well as an increase in CD8 cells (1.6-8.9-fold, mean 3.8-fold). The mean increase was greatest at 4 mcg/kg: NK cells 42-fold, and CD8 cells 4.8-fold. This effector lymphocyte expansion exceeded results seen with other rhIL- 15 dosing regimens or other IL-15 formulations (Table 4). The emergence of pulmonary capillary leak symptoms and slower patient recovery from toxicities at 5 mcg/kg dose level, without a further rise in immune cell subsets, led to our choice of 4 mcg/kg as the highest CIV-5 dose to be tested in new combination treatment trials.

**Conclusions**

The shorter duration of the CIV-5 rhIL-15 regimen and its safety profile may make outpatient administration via an ambulatory infusion pump feasible. The massive expansion of NK cells and increases in CD8 cells it produced were greater than other IL-15 regimens. NK cells are key mediators of antibody-dependent cell cytotoxicity (ADCC); in mice, the increase of NK cell number following CIV IL-15 was associated with increased ADCC of anti-tumor antibodies and their efficacy[5]. Our upcoming trials of CIV-5 rhIL-15 with obinutuzumab or avelumab will leverage the observed massive NK cell expansion to augment the ADCC and therefore their efficacy as anti-tumor antibodies.

**References**

1. Conlon, K.C., M.D. Miljkovic, and T.A. Waldmann, Cytokines in the Treatment of Cancer. J Interferon Cytokine Res, 2018. [epub ahead of print]2. Conlon, K.C., et al., Redistribution, hyperproliferation, activation of natural killer cells and CD8 T cells, and cytokine production during first-in-human clinical trial of recombinant human interleukin-15 in patients with cancer. J Clin Oncol, 2015. 33(1): p. 74-82.3. Conlon, K.C., et al., Abstract 1596: Phase I trial of recombinant human Interleukin 15 (rhIL-15) administered as continuous intravenous infusion (CIV) for 10 days (240 hours) in patients with refractory metastatic cancers. Cancer Research, 2017. 77(13 Supplement): p. 1596-1596.4. Miller, J.S., et al., A First-in-Human Phase I Study of Subcutaneous Outpatient Recombinant Human IL15 (rhIL15) in Adults with Advanced Solid Tumors. Clinical Cancer Research, 2018. 24(7): p. 1525-1535.5. Miljkovic, M.D., et al., IL-15-enhanced antibody-dependent cellular cytotoxicity mediated by NK cells and macrophages: Implications for immunotherapy of T-cell lymphoma [abstract], In: Proceedings of the 2018 AACR Meeting on Advances in Malignant Lymphoma. 2018, Philadelphia (PA): AACR: Boston, Massachusetts.

**Ethics Approval**

The study was approved by the National Cancer Institute's Institutional Review Board, approval number 385250.

**Table 1 (abstract P416).**
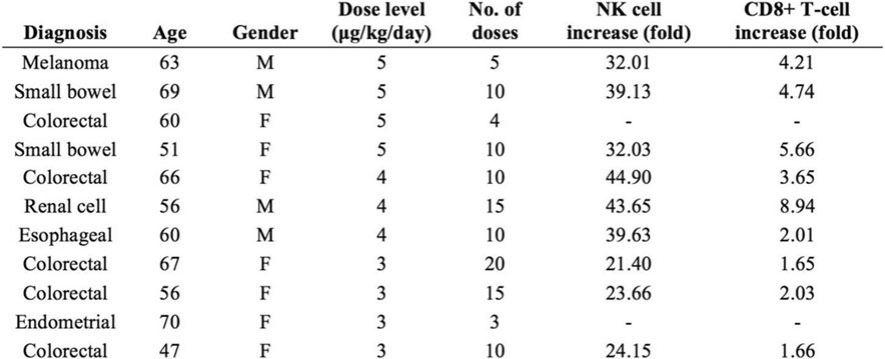
See text for description

**Table 2 (abstract P416).**
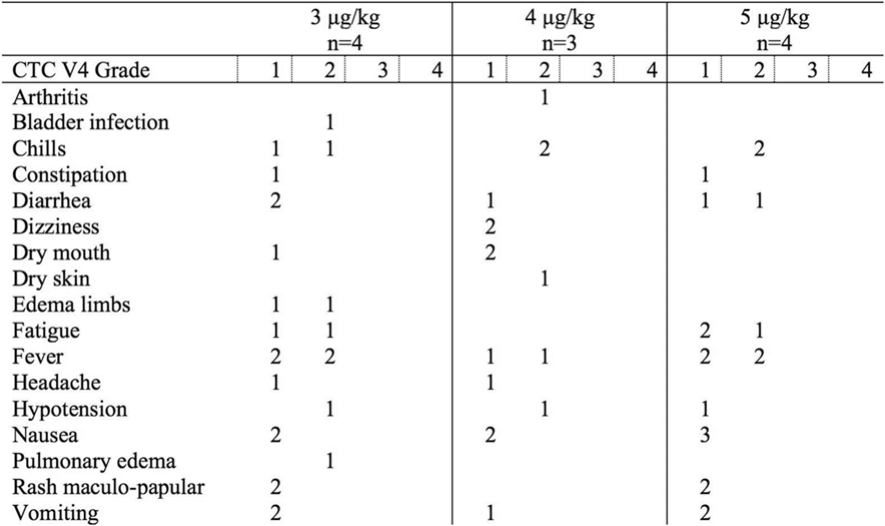
See text for description

**Table 3 (abstract P416).**
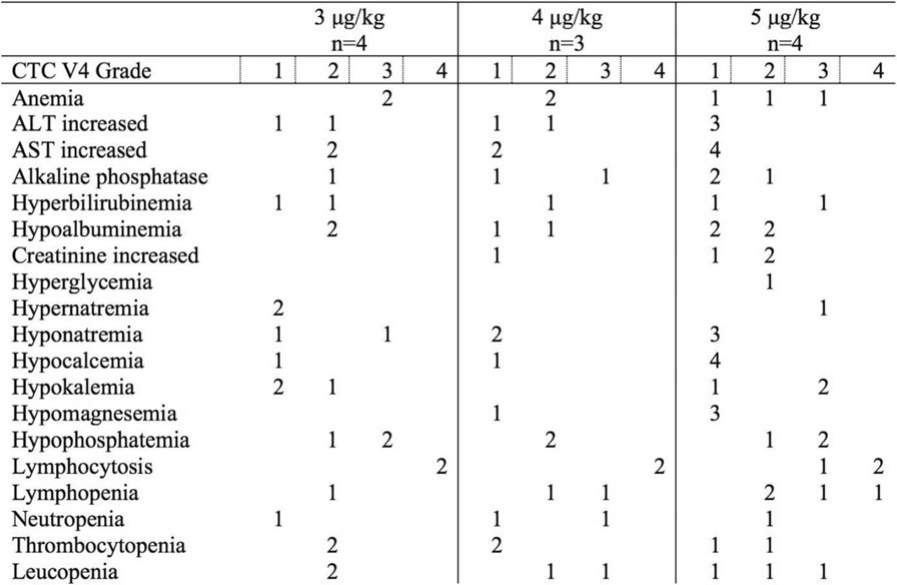
See text for description

**Table 4 (abstract P416).**
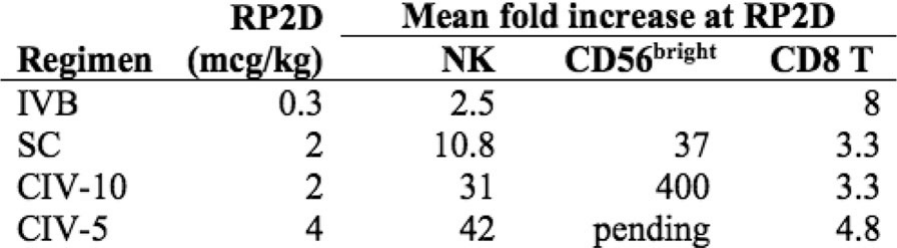
See text for description

#### P417 SYTX80-013-A: an engineered IL-2 for the treatment of solid tumors with superior pre-clinical efficacy and safety evidence

##### Marcos Milla, PhD^1^, Jerod Ptacin, PhD^1^, Carolina Caffaro^1^, Hans Aerni, PhD^1^, Lina Ma^2^, Kristine San Jose^1^, Michael Pena^1^, Robert Herman^1^, Yelena Pavlova^1^, David Chen^1^, Laura Shawver^2^, Lilia Koriazova^1^, Ingrid Joseph^1^

###### ^1^Synthorx, Inc., La Jolla, CA, USA; ^2^Synthorx.com, La Jolla, CA, USA

####### **Correspondence:** Marcos Milla (mmilla@synthorx.com)

**Background**

Aldesleukin, a recombinant form of IL-2, is the first approved immuno-oncology drug leading to complete, durable remissions in metastatic melanoma and renal cell carcinoma patients. Yet, its use is very limited because of vascular leak syndrome (VLS), a severe dose-limiting adverse event stemming from the engagement of the high affinity IL-2 receptor alpha chain in group 2 innate lymphoid cells, eosinophils and vascular endothelial cells. IL-2’s high potency for activation of CD4+ regulatory T cells (Tregs) that suppress T cell-mediated tumor killing responses further reduces its therapeutic window.

**Methods**

N/A

**Results**

We applied our Expanded Genetic Alphabet technology platform to the engineering of SYTX80-013-A: a site-directed, singly pegylated form of IL-2 completely lacking IL-2 receptor (IL-2R) alpha chain engagement yet retaining normal binding to the intermediate affinity beta-gamma IL-2R signaling complex present at the surface of natural killer (NK) and CD8+ tumor-killing cells. SYTX80-013-A potently induces pSTAT5, Ki67 and the proliferation of peripheral NK and CD8+ effector T cells in vivo in mice. Remarkably, dosing of SYTX80-013-A in those animals has minimal effect on molecular and clinical markers of VLS, even at high dose levels. In the mouse CT-26 and B16F10 syngeneic tumor models, SYTX80-013-A induces NK and CD8+ T cell tumor infiltration with marked elevation of CD8+/Treg TIL ratios. In non-human primates, SYTX80-013-A can be dosed for maximal elevation in lymphocytes (pharmacodynamic marker) with negligible elevation in eosinophils (toxicology marker).

**Conclusions**

This demonstrates that SYTX80-013-A is a re-programmed IL-2 that changes the pharmacological profile of that cytokine from low lymphocyte/high eosinophil to high lymphocyte/no eosinophil induction: an IL-2 with a therapeutic window. We are now advancing this molecule into GLP toxicology studies, in preparation for FIH studies in 2019.

#### P418 Pre-clinical investigation of NKTR-255, a polymer-conjugated human IL-15 with a potent NK cell dependent anti-tumor efficacy

##### Takahiro Miyazaki, MS, Murali Addepalli, PhD, Arunasree Lanka, PhD, Amol Murkar, MSc, Ravikumar Nutakki, Palakshi Obalapur, PhD, Peiwen Kuo, PhD, Phi Quach, BS PhD, Mekhala Maiti, PhD, Laurie VanderVeen, PhD, Ping Zhang, MS PhD, Loui Madakamutil, Jonathan Zalevsky, PhD

###### Nektar Therapeutics, San Francisco, CA, USA

####### **Correspondence:** Takahiro Miyazaki (tmiyazaki@nektar.com)

**Background**

IL-15 is a cytokine that activates and provides survival benefit to T and NK cells and has potential as an immunotherapeutic agent for the treatment of cancer. Exploiting the therapeutic value of native IL-15 has been challenging due to, for example, its unfavorable pharmacokinetic properties and tolerability. NKTR-255 is a polymer-conjugated human IL-15 that retains binding affinity to the alpha subunit of IL-15 receptor and exhibits reduced clearance to thereby provide a sustained pharmacodynamics response. Here we investigate the pharmacological properties of NKTR-255 on NK cells and the effect of NKTR-255 in NK cell-dependent tumor models.

**Methods**

For in vivo NK cell characterization, mice received single IV doses of 0.03, or 0.3 mg/kg of NKTR-255. Blood and spleen samples were collected to assess the NK population and function. Flow cytometry was used to measure pSTAT5 and Ki-67 in NK cells. Purified splenic NK cells were co-cultured with YAC-1, a mouse T lymphoma cell line, to measure cytotoxic function. In the CT26 model, 1x105 cells were administered intravenously on Day 0, treatment was initiated on Day 1 at 0.3, 1, or 3 mg/kg, and on Day 13 lungs were scored for metastases. In the orthotopic 4T1 model, 5x105 cells were implanted in the mammary fat pad on Day 0, treatment was initiated on Day 5 at 0.3 mg/kg, and on Day 14, metastases were determined from culture of single lung cell isolates.

**Results**

In vitro, NKTR-255 showed a dose-dependent phosphorylation of STAT5 and enhancement of cytotoxic function in mouse NK cells. NKTR-255 administration increased thebpSTAT5+ populations, the Ki67+ populations and the absolute number of NK cells. In addition, NKTR-255 provided sustained effects of NK cell activation, as determined by enhanced Granzyme B and CD16 expression and cytotoxic function. In the disseminated CT26 model, NKTR-255 treatment resulted in a significant increase of NK cells in lung and a dose-dependent reduction in the number of lung metastases in a NK cell-dependent manner. In the physiological 4T1 metastasis model, NKTR- 255 also showed a significant anti-metastatic effect although it did not affect primary tumor growth.

**Conclusions**

NKTR-255 is a powerful immune stimulator of NK cells that provides a dose-dependent effect in the proliferation and activation of NK cells. This property of NKTR-255 translates into enhanced anti-metastatic activity in mouse lung metastasis models. These results indicate that NKTR-255 has the therapeutic capacity to be an anti-tumor agent that enhances NK cell expansion and survival.

**Ethics Approval**

All animal care and procedures were ethically approved and performed according to AAALAC accredited Nektar Therapeutics IACUC guidelines.

#### P419 NKTR-214 in combination with radiation produces a potent in situ vaccine in the syngeneic B78 melanoma model

##### Alexander Pieper, BS^1^, Alexander Rakhmilevich, MD, PhD^1^, Jacob Slowinski, Mr^1^, Amy Erbe, PhD^1^, Jacquelyn Hank, PhD^1^, Zachary Morris, MD, PhD^1^, Deborah Charych, PhD^2^, Paul Sondel, MD, PhD^1^

###### ^1^University of Wisconsin Madison, Madison, WI, USA; ^2^Nektar Therapeutics, San Francisco, CA, USA

####### **Correspondence:** Alexander Pieper (aapieper@wisc.edu)

**Background**

NKTR-214 is an engineered agonist of the IL2 pathway, biased to the CD122 receptor resulting in sustained signaling and increased CD8/Treg ratios in human and murine tumors. NKTR-214 has shown promising clinical results by enhancing systemic anti-tumor responses. Radiation therapy (RT) alone rarely generates an effective in situ vaccination due, in part, to poor persistence of activated tumor-specific lymphocytes. However, RT can increase tumor immunogenicity by local release of immune stimulatory cytokines, immunogenic tumor cell death, and phenotypic changes that enhance immune susceptibility of tumor cells surviving RT. NKTR-214 may sustain, expand, and drive the systemic anti-tumor response initiated by RT leading to tumor clearance and tumor specific immunologic memory.

**Methods**

C57BL/6 mice were inoculated with B78 melanoma cells on the right flank. Once average tumor volumes reached 125mm3 (~4 weeks), mice were randomized and treated with 12 Gy external beam local RT to this tumor site (defined as treatment day 0). Cohorts of mice were then treated with one of the following: 1) intravenous (IV) IL-2 (0.47 mg/kg), qdx5 starting on day 5; or 2) intra-tumoral (IT) IL2 (0.47 mg/kg), qdx5 starting on day 5; or 3) IV NKTR-214 (0.8 mg/kg) q9dx3 starting on day 5; or 4) buffer alone, q9dx3 starting on day 5. Tumor growth was monitored biweekly. All mice with complete response (CR) were rechallenged at day 90 with a second inoculation of B78 melanoma to test for immunologic memory.

**Results**

Both RT and NKTR-214 alone slowed tumor growth compared to the buffer alone group; however, neither RT nor NTKR-214 alone caused tumor regression. In contrast, the combination of RT + NTKR-214 resulted in significant tumor regression (p<0.01). The rate of complete response (CR) was significantly greater with RT + NKTR-214 compared to RT + IV IL-2 (80% CR vs. 16% CR, p<0.05). RT + NKTR-214 also performed better than RT + IT IL- 2 causing significantly more tumor regression (p<0.01) and a higher CR rate (80% CR vs. 60% CR). The combination of RT + NKTR-214 resulted in stronger immunologic memory than RT + IT IL-2 as more mice receiving RT + NKTR-214 rejected a second B78 inoculation (100% rejection vs. 55% rejection, p<0.01).

**Conclusions**

Previously, IT IL-2 was required to activate and sustain tumor-specific lymphocytes generated from RT of B78. Here we showed that this effect of in-situ vaccination can be realized through IV administration of systemic NKTR- 214 coupled with standard RT.

#### P420 Outpatient staccato pulse intravenous Interleukin-2 in metastatic melanoma

##### Walter Quan, MD^1^, Leah Gutierrez, RN BSN^2^, Erin Johnson^1^, Francine Quan, RN MSN OCN^3^

###### ^1^Loma Linda University, Loma Linda, CA, USA; ^2^Western Regional Medical Center, Goodyear, AZ, USA; ^3^Loma Linda University Beaumont, Tempe, AZ, USA

####### **Correspondence:** Walter Quan (wquan@llu.edu)

**Background**

Daily single intravenous Interleukin-2 (IL-2) infusions (pulses) have been developed to decrease toxicity while maintaining anticancer activity of this molecule against melanoma. Such IL-2 schedules have previously been shown to elicit Lymphokine Activated Killer cell (LAK) activity [1]. Hank has demonstrated in vitro that LAK generated by IL-2 then subsequently exposed to additional IL-2 displayed enhanced cytotoxicity [2]. In patients receiving IL-2 therapy, a rebound lymphocytosis occurs approximately 2-3 days later. The staccato schedule was developed to administer an additional IL-2 pulse during the time of rebound lymphocytosis.

**Methods**

In this retrospective study, twenty-two patients with metastatic melanoma were treated with IL-2 18 Million IU/M2 intravenously over 15-30 minutes on days 1-3 and 21.6 Million IU/M2 intravenously over 15-30 minutes on day 5 on an outpatient basis. Cycles were repeated every 3 weeks.

**Results**

Patient characteristics: 9 males/13 females, median age-55 (range: 21-74), median ECOG performance status-1 (0-1); common metastatic sites: lymph nodes (17), lungs (15), subcutaneous (12), bone (6), liver (4). Prior systemic therapy: Ipilimumab (8); Interferon (7); Pembrolizumab or Nivolumab (7); Interleukin-2 (5); oral targeted therapy (4); none (4). Most common toxicities were nausea/emesis, decreased appetite, sinus/catarrhal symptoms, myalgia/arthralgia, peripheral swelling, and rigors. No patients required hospitalization for toxicity of therapy. One patient (5%) has had a complete response (ongoing at 12.5+ months) while ten other patients (45%) had partial responses (total response rate =50%; 95% CI: 28-72%). Two of the patients with partial responses have been rendered free of disease following surgical resection of their residual cancer. Responses occurred in lung, bones, lymph nodes, pancreas, peritoneum, breast, small bowel, and subcutaneous sites. Median response duration is 10.1 months.

**Conclusions**

Outpatient staccato pulse intravenous Interleukin-2 has activity in melanoma.

**References**

1. Mitchell MS, Kempf RA, Harel W, Shau H, Boswell WD, Lind S, Dean G, Moore J, Bradley EC. Low-dose cyclophosphamide and low-dose interleukin-2 for malignant melanoma. Bull NY Acad Med 1989; 65:128-144.

2. Hank JA, Weil-Hillman G, Surfus JE, Sosman JA, Sondel PM. Addition of interleukin-2 in vitro augments detection of lymphokine-activated killer activity generated in vivo. Cancer Immunol Immunother 1990; 31:53-59.

**Ethics Approval**

The study was approved by Loma Linda University's Institutional Review Board, approval number 5180218.

#### P421 Combination of Pegilodecakin (AM0010) with Docetaxel improves immune cell-mediated anti-tumor response in mouse 4T1 tumor model

##### Navneet Ratti, BS, MBA, Rakesh Verma, PhD, Martin Oft, MD

###### ARMO BioSciences, a wholly owned subsidiary of Eli Lilly and Company, Redwood City, CA, USA

####### **Correspondence:** Navneet Ratti (navneet.ratti@armobio.com)

**Background**

Pegilodecakin is a PEGylated-recombinant hIL-10 that has single agent and combination efficacy with chemotherapy and checkpoint inhibitors across multiple cancers. Pegilodecakin stimulates the survival, proliferation and cytolytic ability of the CD8^+^ T-cells. Clinical studies with Pegilodecakin have reported 41% ORR in combination with anti-PD1 in 2^nd^ line NSCLC. Pegilodecakin induced expansion of PD1^+^Lag3^+^CD8^+^ T-cells correlates with clinical response. Microtubule inhibiting molecules are used as chemotherapeutic agents but combination efficacy with immuno-oncology therapies is not well understood. Here we report the enhanced immune responses and efficacy of AM0010 when combined with Docetaxel.

**Methods**

Pegilodecakin is active, but immunogenic in mice. Therefore, B-cell deficient mice were employed for in-vivo studies. 5x10^3^ 4T1 cells were inoculated subcutaneously and allowed to reach a median tumor volume of 100 mm^3^ prior to treatment. Mice received Pegilodecakin alone at 0.5mpk/qd and/or Docetaxel alone at 40mpk/qw. Tumor size and body weights were monitored twice weekly. Immune cells were phenotyped by flow cytometry. Sera were analyzed for cytokines.

**Results**

The control cohort reached the terminal tumor size by Day 39 PI. Compared to control, Tumor Growth Inhibition percentage (TGI) was 80.91% on Pegilodecakin, 21.39% on Docetaxel and 97.04% on the combination cohort.Docetaxel cohort showed body-weight loss in mice, which was alleviated on Pegilodecakin+Docetaxel. Systemic metastases were only observed in control and Docetaxel cohorts.In the tumors, Pegilodecakin showed an increase of 82-fold in tumor infiltrating T-cells (TILs), 622-fold increase in PD1^+^Lag3^+^CD8^+^ T-cells and a 545-fold increase in proliferative Ki67^+^PD-1^+^Lag-3^+^CD8^+^ T-cells compared to the control cohort.Docetaxel showed an 11- fold increase of TILs but no significant changes in further subsets (CD8^+^/PD1^+^Lag3^+^CD8^+^/Ki67^+^PD1^+^Lag3^+^CD8^+^ T-cells).Pegilodecakin+Docetaxel showed the largest increase in TILs (>400-fold), PD1^+^Lag3^+^CD8^+^ (>1300-fold) and proliferating Ki67^+^PD1^+^Lag3^+^CD8^+^ TILs (1641-fold).Serum IFNG was increased on Pegilodecakin+Docetaxel (6.03pg/mL), compared to 3.39pg/mL on Pegilodecakin, 0.30pg/mL on Docetaxel and 0.72pg/mL in untreated mouse. IFNG was undetectable in control mice at 3 weeks and not available at the terminal endpoint.

**Conclusions**

Pegilodecakin stimulated T-cell mediated tumor regression of 4T1 breast cancers was increased on Pegilodecakin/Docetaxel. Tumor regression correlated with presence and proliferation of PD1^+^Lag3^+^CD8^+^ T-cells in the tumor. Tumor regression and TIL activation was most enhanced on Pegilodecakin+Docetaxel. The immune stimulation of the combination therapy is further reflected in the systemic increase of IFNG in the combination arm compared to monotherapy. These results provide rationale to clinically test a combination Docetaxel with Pegilocakin in tumors with low T-cell infiltration and resistance to available immunotherapies.

#### P422 A polymer-associated human IL-15 (NKTR-255) has optimized biological activity and unique mechanisms of action on CD8 T Cells and NK Cells

##### Tanya Robinson, PhD^1^, Shweta Hegde, Research Assistant^1^, Sarai Rivas, BS^1^, Takahiro Miyazaki, MS^2^, Kimberly S. Schluns, PhD^1^

###### ^1^University of Texas MD Anderson Cancer Center, Houston, TX, USA; ^2^Nektar Therapeutics, San Francisco, CA, USA

####### **Correspondence:** Tanya Robinson (TORobinson@mdanderson.org); Kimberly S. Schluns

**Background**

IL-15 has anti-tumor activity but with limited efficacy due to its unfavorable pharmacokinetic properties and tolerability. Nektar Therapeutics has developed a polymer-conjugated human IL-15 (NKTR-255) that exhibits a prolonged in vivo half-life and enhanced potency, which is currently being examined as a potential cancer immunotherapeutic agent. Since responses by IL-15 can be mediated by transpresentation via the IL-15Rα, as soluble IL-15/IL-15Rα complexes, or by cis-presentation, we investigated the role of IL-15Rα in driving NKTR-255 responses by naïve and memory CD8 T cells and NK cells in mice.

**Methods**

The effects of NKTR-255 were examined by the adoptive transfer of CFSE-labeled naïve ovalbumin-specific CD8 T cells (OT-I) or established memory OT-I T cells followed by systemic administration of NKTR-255. To assess responses by central and effector memory T cell subsets, sorted CD44hi memory phenotype CD8 T cells were transferred into wild-type (Wt) recipients followed by NKTR-255 treatment. Additionally, NK cell responses to NKTR-255 were analyzed in IL-15Rα bone marrow (BM) chimeras by BrdU incorporation.

**Results**

Naïve CD8 OT-I T cells transferred into Wt and IL-15Rα-/- mice proliferated at similar levels and acquired a central memory phenotype in response to NKTR-255. Interestingly, naive IL-15Rα-/- OT-I T cells had a deficient response to NKTR-255 but not to rhIL-15 or soluble IL-15 complexes. Additionally, proliferation by memory IL-15Rα-/- OT- I T cells in response to NKTR-255 was partially impaired compared to Wt OT-I cells. Sorted memory CD8 T cells maintained their proportion of CD62L+ and - subsets after NKTR-255-stimulated proliferation. Since IL-15Rα expression is essential for NK cell development, BM chimeras were generated with either IL-15Rα-/- or Wt BM in Wt recipients. In this model system, similar levels of BrdU were incorporated in IL-15Rα-/- and Wt NK cells after treatment with NKTR-255.

**Conclusions**

These findings suggest naive CD8 T cells are critically dependent on cis-presentation of NKTR-255, while memory CD8 T cells are only partially dependent. For both naive or memory CD8 T cells, transpresentation of NKTR-255 was not required. In contrast to CD8 T cells, NK cell responses to NKTR-255 are not dependent on cis-presentation. Overall, these findings highlight the potential of polymerized IL-15 to modify IL-15Rα dependency leading to different mechanisms of action on CD8 T cells and NK cells and unique therapeutic effects.

**Ethics Approval**

All animal procedures were conducted in accordance with the animal care and use protocols (00000851-RN01) approved by the IACUC at the UT MD Anderson Cancer Center.

#### P423 Safety, pharmacokinetics and pharmacodynamic effects of ALKS 4230 in patients with advanced solid tumors from the ongoing dose escalation portion of a first in human (FIH) study

##### Ulka Vaishampayan, MD^1^, Vamsidhar Velcheti, MD FACP^2^, David McDermott, MD^3^, Mayer Fishman, MD, PhD^4^, Chris Hoimes, MD^5^, Daniel Cho, MD^6^, Lei Sun, Ph.D^7^, Juan Alvarez, PhD^8^, Heather Losey, PhD^7^, Rose Marino, MD^7^, Emily Putiri, PhD^7^, Sean Rossi^7^, Lisa Von Moltke, MD^7^, William Slichenmyer, MD^9^, Marc Ernstoff, MD^10^

###### ^1^Barbara Ann Karmanos Cancer Institute, Detroit, MI, USA; ^2^Cleveland Clinic, Pepper Pike, OH, USA; ^3^Beth Israel Deaconess Medical Center, Boston, MA, USA; ^4^Moffitt Cancer Center, Tampa, FL, USA; ^5^University Hospital, Cleveland, OH, USA; ^6^New York University, New York, NY; ^7^Alkermes, Inc., Waltham, MA, USA; ^8^Merck, Boston, MA, USA; ^9^Alacrita, Waltham, MA, USA; ^10^Roswell Park Cancer Institute, Buffalo, NY, USA

####### **Correspondence:** Lei Sun (Lei.Sun@alkermes.com)

**Background**

ALKS 4230 is a fusion of circularly permuted IL-2 and IL-2 Receptor (IL-2R) α designed to selectively activate the intermediate-affinity IL-2R, comprised of IL-2Rβ and γ, for activation of cytotoxic CD8+ T cells and NK cells. ALKS 4230 has previously been shown to have enhanced antitumor activity relative to IL-2 in murine models.

**Methods**

In the ongoing FIH Phase 1 study in patients with advanced solid tumors, ALKS 4230 is administered as a 30 minute intravenous infusion once daily for 5 consecutive days repeating in treatment cycles of 14 days (first cycle) or 21 days (subsequent cycles). The primary objectives are to investigate ALKS 4230 safety and tolerability and to determine the maximum tolerated dose and recommended Phase 2 dose. Other assessments include pharmacokinetics, lymphocyte sub-population expansion, immunogenicity, and anti-tumor activity.

**Results**

Twenty-four patients have received ALKS 4230 at doses ranging from 0.1 to 3 μg/kg/day. Patients with multiple tumor types were enrolled, including 5 with prostate carcinoma, 4 with renal cell carcinoma, and 3 with melanoma.

Patients had a median of 3 (range 1-8) prior lines of systemic therapy. The most common treatment emergent adverse events (AEs) seen in ≥ 60% of patients were fever and chills. Grade 3 treatment-related AEs seen in 1-2 patients occurred at the 3 μg/kg/day dose level and included neutropenia, leukopenia, jaundice, febrile neutropenia, lymphopenia, diarrhea, cholangitis, hyperbilirubinemia and hypoalbuminemia. There were no Grade 4 or 5 AEs. Systemic exposure to ALKS 4230 increased with increasing dose and serum ALKS 4230 concentrations at 3 μg/kg/day have exceeded the EC50 values for NK and CD8+ T cell activation determined in in vitro pharmacology studies. Treatment with ALKS 4230 resulted in a dose-dependent increase in circulating NK and CD8+ T cells with an approximately 4-fold and 2-fold expansion at 3 μg/kg/day, respectively, and minimal, non-dose dependent change in Tregs. Transient, dose dependent elevations in serum IL-6 levels occurred 4-6 hours post-dose and were associated with transient fever and chills but not cytokine storm. No objective responses have been seen, and dose escalation is ongoing.

**Conclusions**

ALKS 4230 was well tolerated at the doses tested, with treatment-related AEs that were manageable and transient. The 3 μg/kg/day dose level induced expected immunologic effects, supporting the rationale for assessing combination therapies with ALKS 4230, as well as continued dose escalation in the monotherapy setting.

**Acknowledgements**

Study was sponsored by Alkermes, Inc. The authors gratefully acknowledge the patients and their families who participated in this study.

**Trial Registration**

Trial Registration at Clinicaltrials.gov: NCT02799095

**Ethics Approval**

The study was approved by Beth Israel Deaconesses Medical Center Investigation Review Board (IRB), approval number 16-229, Roswell Park Cancer Institute IRB, approval number MOD00002327 / PH 285316, Cleveland Clinic IRB, approval number 16-804, Western IRB, approval number 1166122, New York University IRB, approval number i15-01394, University Hospitals IRB, approval number 16-804, and Chesapeak IRB approval number 00000790.

#### P424 NKTR-214, an engineered IL-2, selectively depletes intratumoral Tregs and expands immunotherapy- induced effector T cell responses

##### Meenu Sharma, PhD^1^, Hiep Khong, PhD^1^, Faisal Fa'ak, MD^1^, Brent Chesson^1^, Barbara Pazdrak^1^, Laura Maria S Kahn^1^, Louise Janssen, MSc^1^, Uddalak Bharadwaj^1^, Binisha Karki^1^, Zhilan Xiao, MD^1^, Yared Hailemichael, PhD^1^, Manisha Singh, PhD^1^, Christina Vianden, MSc^1^, David Tweardy^1^, Salah Eddine Bentebibel^1^, Cara Haymaker, PhD^1^, Chantale Bernatchez^1^, Adi Diab, MD^1^, Ute Hoch, PhD^2^, Jonathan Zalevsky, PhD^2^, Willem W. Overwijk, PhD^1^

###### ^1^UT MD Anderson Cancer Center, Houston, TX, USA; ^2^NEKTAR Therapeutics, San Francisco, CA, USA

####### **Correspondence:** Willem W. Overwijk (woverwijk@mdanderson.org)

**Background**

High dose IL-2 has been used in treatment of metastatic melanoma and renal cell carcinoma. However, expansion of suppressive Tregs and physiologic toxicities associated with IL-2 has limited its use in anti-cancer therapies. NKTR- 214 is an engineered IL-2 cytokine that provides sustained activation of the IL-2 pathway through controlled release of IL-2 with a bias to the IL-2 receptor CD122 (IL-2Rbeta gamma), so that it can selectively enhance CD8+ T cell over regulatory T cells (Tregs). We tested this idea by assessing the therapeutic synergy of NKTR-214 with CTLA-4 and PD-1-based checkpoint blockade therapy or with peptide-vaccination in CT26 colon carcinoma and B16 melanoma models. We investigated impact of treatment on proliferation and apoptosis of effector CD8+ T cells and immunosuppressive CD4+Foxp3+ Tregs, as well as effector cytokines and chemokines in tumor and peripheral tissues. In vivo cytokine neutralization experiments and in vitro assays revealed that NKTR-214 plus vaccine treatment induced CD8+ effector T cell responses and enhanced associated cytokines IFN-gamma and TNF-alpha that mediate specific depletion of intratumoral, but not peripheral, Tregs.

**Methods**

CT26 colon carcinoma tumor bearing mice were treated with NKTR-214 or CTLA-4 and/or PD-1 checkpoint blockade. Tumor size, survival and tumor-specific effector T cell response was analyzed. To monitor antigen-specific immune response, we adoptively transferred naïve gp100-specific pmel-1 CD8+ T cells into mice bearing established B16 tumors, followed by a single vaccination (gp100 peptide + anti-CD40 mAb + TLR-7 agonist) alone or in combination with NKTR-214 or Aldesleukin (IL-2) given every 8 days. Detailed analysis of CD8+ T cells and Tregs was done by flow cytometry. Chemokines/cytokines levels in tumor and spleen were measured by luminex- based assay.

**Results**

NKTR-214 efficiently synergized with checkpoint blockade and with vaccination, improving overall survival and cure of mice in models of colon carcinoma and melanoma. NKTR-214 promoted the CD8+ T cell survival, expansion and release of associated cytokines, IFN-gamma and TNF-alpha, which synergized to induce apoptosis and inhibited proliferation of Tregs specifically in tumors (Figure 1) while preserving Tregs in peripheral tissues. In vitro cytokine treatment also confirmed that IFN-gamma and TNF-alpha together are both sufficient and required to block Treg proliferation. Preliminary results confirm similar therapeutic effects with cancer patients receiving clinical doses of NKTR-214.

**Conclusions**

NKTR-214 synergizes with checkpoint blockade as well as with vaccination to improve the survival, proliferation and tumor infiltration of effector CD8+ T cells while promoting selective intratumoral depletion of Tregs to establish effective anti-tumor immunity.

**Fig. 1 (abstract P424).**
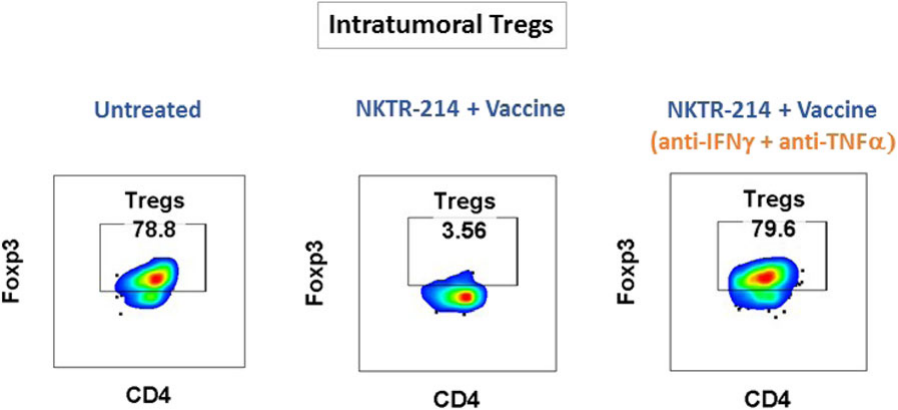
NKTR-214 mediated intratumoral Treg depletion

#### P425 Pharmacokinetics and pharmacodynamic effects of ALKS 4230, an investigational immunotherapeutic agent, in cynomolgus monkeys after intravenous and subcutaneous administration

##### Lei Sun, PhD, Jared Lopes, PhD, Heather Flick, MS, Erin Murphy, MS, Heather Losey, PhD

###### Alkermes, Inc., Waltham, MA, USA

####### **Correspondence:** Lei Sun (Lei.Sun@alkermes.com)

**Background**

ALKS 4230 is an engineered cytokine designed to selectively activate the intermediate-affinity interleukin-2 receptor (IL-2R), expressed predominantly on natural killer (NK) cells and CD8+ T cells, which play an important role in driving immune responses in cancer. A first-in-human study of intravenous administration of ALKS 4230 in patients with advanced solid tumors (NCT02799095) is currently ongoing. To compare the pharmacodynamic responses in response to the intravenous and subcutaneous administration of ALKS 4230, two studies were carried out in cynomolgus monkeys.

**Methods**

In the first study, a single dose of ALKS 4230 was administered intravenously or subcutaneously. In the second study, ALKS 4230 was administered intravenously once daily on Days 1-5 or subcutaneously on Days 1 and 4. Serial blood samples were collected from each animal for determination of serum concentrations of ALKS 4230 and multiple proinflammatory cytokines as well as for immunophenotyping by flow cytometry.

**Results**

Overall systemic exposure to ALKS 4230 as measured by area under the serum concentration vs. time (C-T) curve (AUC) after a single subcutaneous dose of 1 mg/kg was comparable to that after an intravenous dose of 0.3 mg/kg, suggesting a subcutaneous bioavailability of ~30%. With comparable AUC but lower Cmax, subcutaneous administration elicited greater expansion of CD8+ T cells and CD56+ NK cells as well as a superior ratio of CD8+ T cells to CD4+CD25+FoxP3+ Tregs compared to intravenous administration. In addition, expansion of CD8+ T cells and CD56+ NK cells was sustained up to 12 days after a single dose. Total systemic exposure to ALKS 4230 was comparable after 5 daily intravenous doses of 0.1 mg/kg and 2 subcutaneous doses of 0.5 mg/kg (on Days 1 and 4) and resulted in similar expansion of total CD8+ T cells, NK cells and Tregs between the two dosing regimens. The serum IL-6 C-T profile mirrored the ALKS 4230 C-T profile, with a higher peak IL-6 level and a higher Cmax of ALKS 4230 following the last intravenous dose of 0.1 mg/kg compared to the last subcutaneous dose of 0.5 mg/kg.

**Conclusions**

Subcutaneous administration of ALKS 4230 can achieve similar total systemic exposure to ALKS 4230 compared to intravenous administration with less frequent dosing and a lower Cmax, leading to similar expansion of total CD8+ T cell and NK cell populations. Therefore subcutaneous administration may be a practical alternative to intravenous dosing and merits clinical evaluation.

### Emerging Models and Imaging

#### P426 Chaos-based fractal radiomic features of nodule vasculature predicts response to immunotherapy on non- contrast lung CT

##### Mehdi Alilou, PHD^1^, Marjan Firouznia, PHD^1^, Pradnya Patil^2^, Kaustav Bera, MBBS^1^, Robert Gilkeson^3^, Prabhakar Rajiah^3^, Vamsidhar Velcheti, MD FACP^2^, Anant Madabhushi, PhD^1^

###### ^1^Case Western Reserve University, Cleveland, OH, USA; ^2^Cleveland Clinic, Cleveland, OH, USA; ^3^University Hospital Case Medical Center, Cleveland, OH, USA

####### **Correspondence:** Mehdi Alilou (me.alilou@gmail.com)

**Background**

Immune-checkpoint blockade treatments, particularly drugs inhibiting programmed death-ligand 1 (PD-L1) with its receptor, programmed cell death protein-1 (PD-1) has demonstrated promising clinical efficacy in patients with advanced non-small cell lung cancer (NSCLC). In spite of recent regulatory approval of several immunotherapy (IO) drugs, the objective response rate of these drugs is modest (~20%) at best. The complex nature of the host immune response makes tissue based biomarker development for IO response assessment challenging. Consequently, there is an urgent and critical unmet need to develop accurate, validated biomarkers to predict which NSCLC patients will benefit from IO. Previous research has shown that the morphology of the tumor feeding vessels plays a role in cancer aggressiveness as well as therapeutic refractoriness. Post-treatment tumors show significant improvement in vessel tortuosity abnormalities when compared before therapy initiation. Hence, we sought to evaluate whether computer extracted measurements of fractal features of nodule associated vessel morphology on baseline CT scans in NSCLC patients treated with Nivolumab could distinguish between patients who did and did not respond to the PD-1 inhibitor.

**Methods**

Our study comprised non-contrast CT scans of 61 patients obtained retrospectively from the Cleveland Clinic, including 31 patients who responded to Nivolumab and 30 non-responders. Patients who did not receive Nivolumab after 2 cycles due to lack of response or progression as per RECIST were classified as ‘non-responders’, patients who had radiological response or stable disease as per RECIST were classified as ‘responders’. From nodule annotations provided by a trained radiologist, a region-growing algorithm was used to segment the surrounding vasculature (Figure1A). A set of 12 vessel fractal radiomic (VFR) measurements pertaining to the fractal analysis, the state space reconstruction and Lyapunov exponent were extracted from each nodule associated vasculature. A Naive Bayes classifier was then used, in a 3-fold cross-validation setting through 200 iterations, to construct a classifier to identify which patients respond to nivolumab therapy.

**Results**

VFR features (Figure1B) were found to distinguish responders from non-responders to Nivolumab with an AUC=0.73±0.08 . Statistically significant difference was observed for two VFR features between responders and non-responders (p<0.009).

**Conclusions**

VFR were able to distinguish responders from non-responders for patients with NSCLC and treated with Nivolumab. The VFR could potentially serve as a predictive tool for response assessment for immune checkpoint inhibitors and enable selection of NSCLC patients who will benefit from IO; paving the way for design of more rational clinical trials with combination of IO agents.

**Ethics Approval**

The study protocol was approved under University Hospitals (UH) IRB 02-13-42C.

**Fig. 1 (abstract P426).**
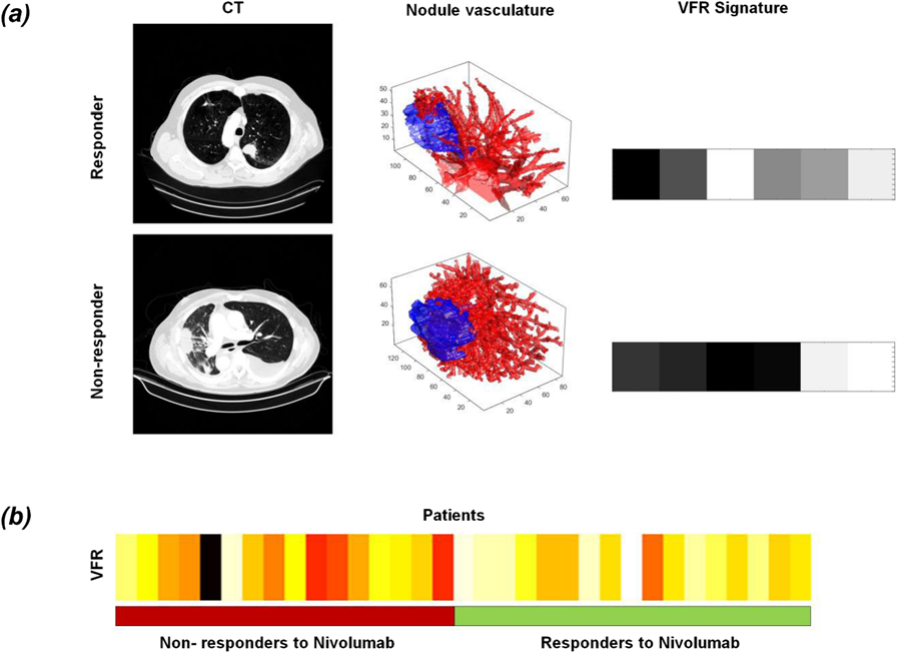
See text for description

#### P427 Novel immune competent murine glioblastoma models derived from Nestin-CreER^T2^ Quaking^L/L^; P53^L/L^; PTEN^L/L^ mice

##### Chao-Hsien Chen, MD^1^, Renee Chin, MS^1^, Genevieve Hartley, PhD^1^, Takashi Shingu, PhD^2^, David Hong, MD^1^, Jian Hu, PhD^1^, Michael A. Curran, PhD^1^

###### ^1^The University of Texas MD Anderson Cancer Center, Houston, TX, USA; ^2^University of Texas MD Anderson Cancer Center, Houston, TX, USA

####### **Correspondence:** Michael A. Curran (mcurran@mdanderson.org)

**Background**

Despite the success of immunotherapy in several cancers, antibody blockade of the immune checkpoint receptor PD-1 failed to improve the survival of recurrent glioblastoma multiforme (GBM) patients [1]. In contrast to this clinical reality, the widely used immunocompetent mouse model of GBM, GL261, is highly immunogenic and readily cured by T-cell checkpoint blockade therapy [2]. The resulting inability to model the immunotherapeutic sensitivity of human GBM preclinically prevents effective translation of murine observations to clinical therapies. Quaking (QKI) is a GBM tumor suppressor gene which is deleted, mutated or downregulated in the majority of human GBM [3,4], the expression level of which strongly correlates with patient survival [5]. We describe novel murine immunocompetent glioblastoma stem cell (GSC) lines derived from Nestin-CreER^T2^ Quaking (QKI)^L/L^; P53^L/L^; PTEN^L/L^ (QPP) mice [5] and determine their sensitivities to immunotherapies.

**Methods**

We selected four lines, namely QPP4, 5, 7 and 8, after validation of their engraftment in C57BL6/J mice. The immunotherapeutic sensitivities in response to systemic CTLA-4 and PD-1 blockade therapies were determined by tumor growth kinetics and survival. The tumor microenvironment (TME) was evaluated by flow cytometry analysis.

**Results**

All four QPP lines express GSC markers, such as CD171 and α2β5, but lack PD-L1 or PD-L2 expression *in vitro* and *in vivo*, excepting limited PD-L1 expression by QPP7 *in vivo*. This fits the observation that only a small proportion of human GBM expresses PD-L1 [6]. These QPPs have distinct sensitivities to systemic checkpoint blockade in different niches. Subcutaneously, QPP4, 5 and 8 are sensitive to CTLA-4 blockade, and QPP7 is sensitive to both PD-1 and CTLA-4 blockades. In the brain, QPP5 and 7 remain sensitive to CTLA-4 blockade (*n*= 8-15, *p*<0.05), while QPP4 and 8 resist both PD-1 and CTLA-4 blockades (*n*= 9-15, *p*>0.05) (Figure 1). Preliminary analysis of the orthotopic TME of the checkpoint-resistant QPP8 line reveals no significant change in CD8 T cells, regulatory CD4 T cells (Treg), myeloid-derived suppressor cells (MDSCs), tumor associated macrophages (TAMs) and microglia infiltration, or CD8/Treg and CD8/MDSCs ratios with either CTLA-4 or PD-1 blockade (*n*=3-5). PD- L1 expression on monocytic MDSCs, TAMs and microglia in the PD-1 or CTLA-4 blockade group are significantly increased (*p*<0.05) (Figure 2), however, which could reveal the origins of the prognostic value of the PD-1/PD-L1 axis in human GBM [7].

**Conclusions**

The distinct checkpoint blockade sensitivities of QPP lines could fill the critical need for preclinical GBM models suitable for evaluating immunotherapeutics.

**References**

1. Reardon DA, Omuro A, Brandes AA, Rieger J, Wick A, Sepulveda J, Phuphanich S, de Souza P, Ahluwalia MS, Lim M, Vlahovic G, Sampson J OS10.3 Randomized phase 3 study evaluating the efficacy and safety of nivolumab vs bevacizumab in patients with recurrent glioblastoma: CheckMate 143. Neuro-Oncology. 2017; 19: iii21-iii21.

2. Reardon DA, Gokhale PC, Klein SR, et al. Glioblastoma eradication following immune checkpoint blockade in an orthotopic, immunocompetent model. Cancer Immunol Res. 2016; 4(2):124-135.

3. Hu J, Ho AL, Yuan L, et al. From the cover: neutralization of terminal differentiation in gliomagenesis. Proc Natl Acad Sci U S A. 2013; 110(36):14520-14527.

4. Brennan CW, Verhaak RG, McKenna A, et al. The somatic genomic landscape of glioblastoma. Cell. 2013; 155(2):462-477.

5. Shingu T, Ho AL, Yuan L, et al. Qki deficiency maintains stemness of glioma stem cells in suboptimal environment by downregulating endolysosomal degradation. Nat Genet. 2017; 49(1):75-86.

6. Hodges TR, Ott M, Xiu J, et al. Mutational burden, immune checkpoint expression, and mismatch repair in glioma: implications for immune checkpoint immunotherapy. Neuro Oncol. 2017; 19(8):1047-1057.

7. Nduom EK, Wei J, Yaghi NK, et al. PD-L1 expression and prognostic impact in glioblastoma. Neuro Oncol. 2016; 18(2):195-205.

**Fig. 1 (abstract P427).**
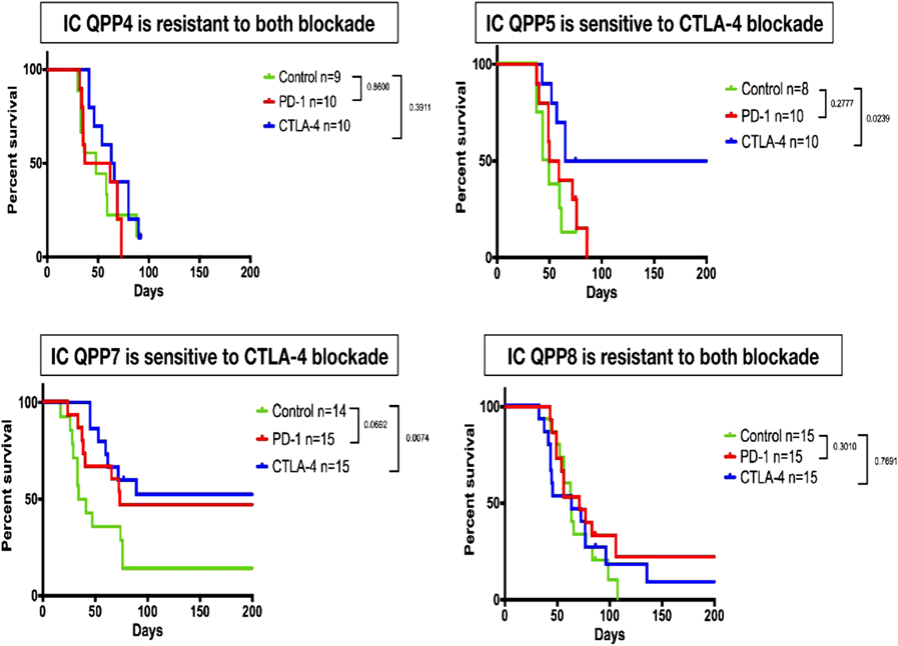
Orthotopic survival and immune sensitivities

**Fig. 2 (abstract P427).**
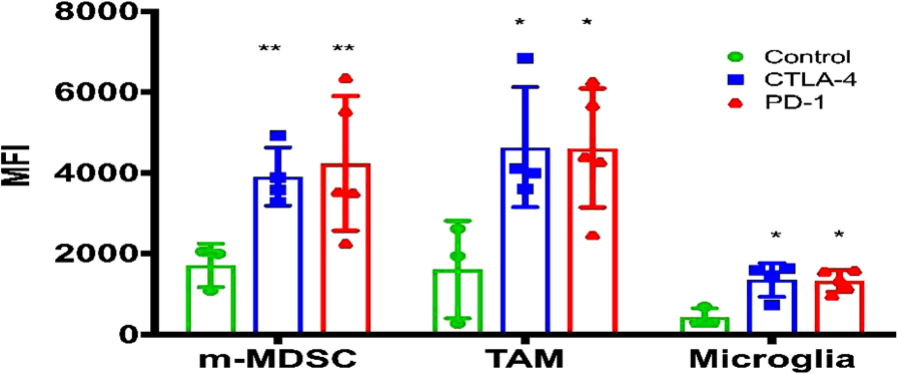
PD-L1 expression on myeloid cells in QPP8 TME

#### P428 Spatially-resolved, high-plex digital profiling enables characterization of complex immune biology of the colorectal cancer microenvironment

##### Sarah Church, Chris Merritt, PhD, Andrew White, BSc, Douglas Hinerfeld, PhD, Dan Zollinger, Giang Ong, MS, Kristi Barker, MS, Sarah Warren, PhD, Joseph Beechem, PhD

###### NanoString Technologies, Seattle, WA, USA

####### **Correspondence:** Sarah Church (schurch@nanostring.com)

**Background**

Spatial characterization of the tumor microenvironment (TME) interface between cancer cells, stroma and immune cells is essential for understanding tumor progression and discovering prognostic and predictive biomarkers. However, it has proven difficult to perform such studies in a highly multiplexed manner using limited sample quantity. Digital Spatial Profiling (DSP) has been developed as a research use instrument, software and chemistry for hi-plex profiling of mRNA and protein using an optical-barcode read-out. In this study, microsatellite stable (MSS) or instable (MSI) characterized colorectal tumors were characterized using DSP with 40 proteins or 48 RNA probes to evaluate active and suppressive immune mechanisms in both immune dense regions and tumor versus stroma.

**Methods**

Sixteen FFPE colorectal tumors that were characterized for Microsatellite stability status were mounted on slides. Tissue sections were stained with a cocktail of pan-cytokeratin, CD45, CD3 and DNA fluorescent markers and 48 RNA probes or 40 antibodies, each conjugated to a UV-photocleavable DNA barcode. Regions of interest (ROI) were delineated using the immunofluorescence followed by UV excitation of the defined ROIs, which releases the DNA barcodes for downstream quantitation on the NanoString nCounter® platform. Two strategies were used for selecting ROIs, 1) Geometric profiling of CD45-enriched hotspots in the tumor center and invasive margin and 2) Segment profiling of cytokeratin-positive tumor regions compared to cytokeratin-negative regions.

**Results**

We show that deep profiling of CD45-enriched regions from the invasive margin and tumor center of MSS and MSI tumors have different immunosuppressive and activated immune phenotypes. Comparing colorectal tumors characterized as MSS, DSP was able to differentiate immune hot and cold tumors despite MSS status. Further evaluation using segment profiling of tumors versus stroma also identified specific immune proteins and RNA pathways that were distinctly related to each compartment and were different between MSI and MSS tumors.

**Conclusions**

Our results suggests DSP has the potential to be used to predict patients' response to PD-1 immune checkpoint blockade with greater sensitivity than standard MSS/MSI profiling, and furthermore DSP may allow identification of unique localized immune characteristics that would guide combination therapeutic approaches.

#### P429 Integrative spatially-resolved, high-plex digital profiling enables characterization of complex immune biology in the tumor microenvironment of mesothelioma

##### Carmen Ballesteros Merino, PhD^1^, Moritz Widmaier, PhD^2^, Sarah Church^3^, Thomas Herz, PhD^2^, Alexei Budco, MSC^2^, Dasa Medrikova, PhD^2^, Ivan Kanchev, PhD^2^, Andrew White, BSc^3^, Douglas Hinerfeld, PhD^3^, Shawn Jensen, PhD^1^, John Handy, MD^1^, Rachel Sanborn, MD^1^, Carlo Bifulco, MD^1^, Sarah Warren, PhD^3^, Joseph Beechem, PhD^3^, Bernard A. Fox, PhD^1^

###### ^1^Providence Portland Cancer Center, Portland, OR, USA; ^2^Definiens, Munich, Germany; ^3^NanoString Technologies, Seattle, WA, USA

####### **Correspondence:** Bernard A. Fox (foxb@foxlab.org)

**Background**

Malignant mesothelioma is an aggressive cancer with poor prognosis and few effective therapies. Since mesothelioma is derived from the mesothelium of the lung, we hypothesize that immune cells in the tumor microenvironment (TME) may behave differently than other solid tumors. In our previous studies, utilizing multi- plexed immunofluorescence, we did not find immune phenotypes associated with improved patient survival. Here we describe a novel combination of two technologies to spatially characterize the interface between mesothelioma cells, stroma and immune cells in the TME in a high-plex capacity.

**Methods**

Ten FFPE mesothelioma tumors were characterized by Definiens’ Immune-Oncology Profiling (IOP) and NanoString Digital Spatial Profiling (DSP). Three alternating sequential sections were stained with Definiens’ IOP (CD8/PD-1/FOXP3, CD68/PD-L1/CD3, Granzyme B). Definiens analysis was combined to identify localization of each marker in the tumor center, invasive margin or stroma. Twelve regions-of-interest (ROIs) were then selected based on the Definiens analysis for high-plex analysis on DSP on the interleaving slide: 4 CD68-enriched, 6 CD8- enriched and 2 CD3-low. For DSP analysis, each slide was stained with a combination of fluorescent-labeled antibodies (pan-cytokeratin, CD3, CD68) and a panel of 38-antibodies each conjugated to a unique UV- photocleavable DNA barcode. ROIs from Definiens’ defined analysis were overlayed on DSP fluorescent scans, followed by UV excitation of the defined ROIs, which releases the DNA barcodes for downstream quantitation on the NanoString nCounter® platform.

**Results**

We found strong correlation between Definiens and NanoString analysis of T cell and macrophage markers in selected regions. Generally, patients with longer survival (>6 months) had increased density of immune infiltrates including higher density of T cells, T-cell activation markers (PD-1), higher cytokeratin levels and decreased Ki67 in the tumor center and increased tertiary lymphoid structure makers (B cells) in the invasive margin. Furthermore, STING and VISTA were highly expressed across all mesotheliomas. However, the patient with the longest survival (>31 months) expressed an immune-excluded phenotype. Co-localization analysis revealed that high CD68 density was tightly correlated to PD-L1 expression and in at least one case additional suppressive macrophage markers, including CD163 and B7-H3.

**Conclusions**

Already this small data set demonstrates that integration of two novel high-plex spatial analysis techniques separates distinct immune mechanisms in the TME. Our analysis suggests that macrophages are highly associated with expression of immune-inhibitory signals in mesothelioma. Therefore, we hypothesize that analysis of additional mesotheliomas may guide the development of combination immunotherapy trials that will be effective against this incurable disease.

#### P430 Radiomic texture features from MR perfusion images predicts pseudoprogression from true progression in glioblastoma patients: A multi-institutional study

##### Nabil Elshafeey^1^, Aikaterini Kotrotsou^1^, Srishti Abrol^1^, Islam Hassan^1^, Ahmed Hassan^1^, Kamel El Salek, MD^1^, Fanny Moron^2^, Meng Law^3^, Pascal Zinn^2^, Rivka Colen, MD^4^

###### ^1^MD Anderson Cancer Center, Houston, TX, USA; ^2^Baylor College of Medicine, Houston, TX, USA; ^3^University of Southern California, Los Angeles, CA, USA; ^4^The University of Texas, Houston, TX, USA

####### **Correspondence:** Rivka Colen (rcolen@mdanderson.org)

**Background**

Pseudoprogression (PsP) is an inflammatory response associated with radiation and necrotic induced changes reflective of treatment, appearing as areas of increased enhancement on postcontrast T1-weighted images. Response assessment criteria, such as RANO, struggle to distinguish between true progression and PsP. Advanced imaging techniques (MR perfusion, MR diffusion) have been proposed as an alternative way of distinguishing between PsP and progressive disease (PD). However, the outcome of such studies underscores the need for novel tools distinguish between these. In this study, we sought to evaluate the utility of radiomic analysis of MR perfusion [Dynamic contrast enhancement (DCE) and Dynamic susceptibility contrast (DSC)] maps in differentiating PsP from PD.

**Methods**

Patients: A total of 98 patients were included in this multi-institutional IRB-approved study. All had pathological confirmation; 78 patients with PD and 20 patients with PsP. Radiomic Analysis: All patients underwent DSC and DCE perfusion MRI as part of their routine clinical care. Images were analyzed using Nordic ICE 2.3 (NordicNeuroLab); rCBV and Ktrans maps were obtained. Subsequently, an experienced radiologist delineated the entire tumor on DCE and DSC maps using 3D slicer (http://www.slicer.org) (Figure 1). The extracted 3D region-of- interest (ROI) parametric maps were imported in the radiomic pipeline. A total of 475 features (10 histogram-based and 375 higher-order texture features) were calculated for each parametric map. Statistical Analysis: An advanced feature selection method based on Minimum Redundancy Maximum Relevance (MRMR) was used to analyze the featureset and extract core features. Selected features were used to build a Support Vector Machine (SVM) model for prediction of PD versus PsP. To evaluate the robustness of the estimates made with the SVM models, leave-one- out cross-validation (LOOCV) was conducted. Finally, box plots of the 10 most relevant features and probability maps were calculated.

**Results**

MRMR identified 50 radiomic features that were further used to build the SVM model. The prediction of progression by LOOCV was significant p-value=0.031. Area under the curve (AUC), sensitivity and specificity were 89.26%, 81.82% and 100% respectively and the most discriminating features were variance and sum entropy (Figure 2). Box plots of the 10 most relevant features are shown in Figure 3.

**Conclusions**

This study demonstrates that MR perfusion radiomic analysis can discriminate between PsP and PD. Further validation and a comparative study of radiomic analysis of MR perfusion maps and conventional MR images would be valuable to determine which approach is more effective, and the added value in combining the two approaches.

**Fig. 1 (abstract P430).**
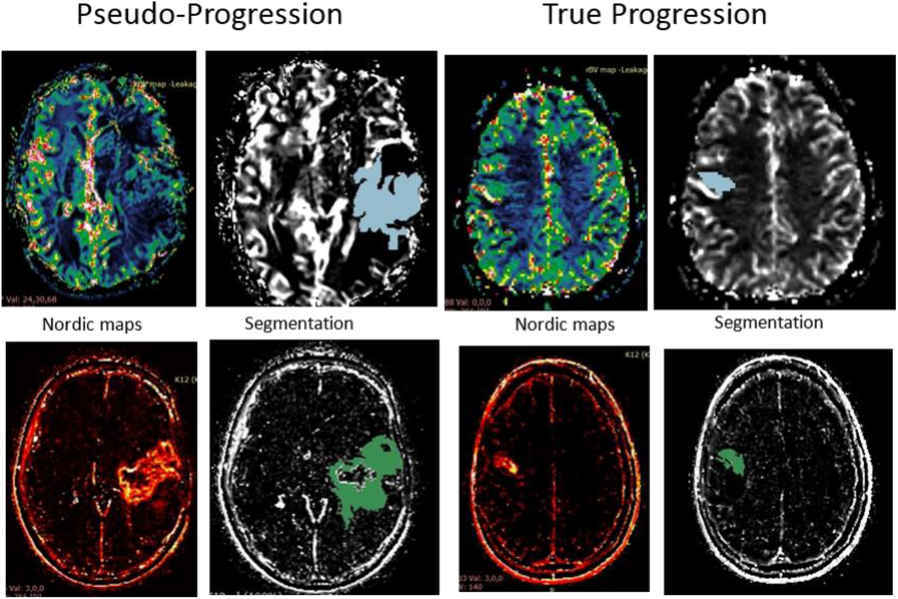
See text for description

**Fig. 2 (abstract P430).**
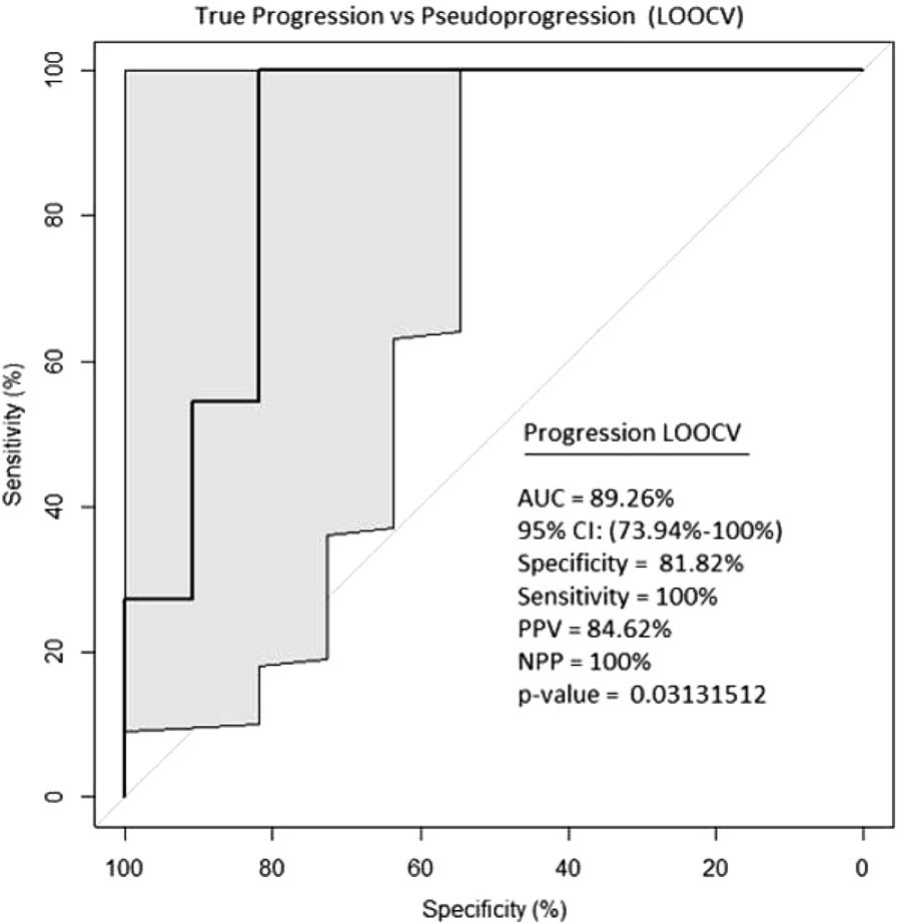
See text for description

**Fig. 3 (abstract P430).**
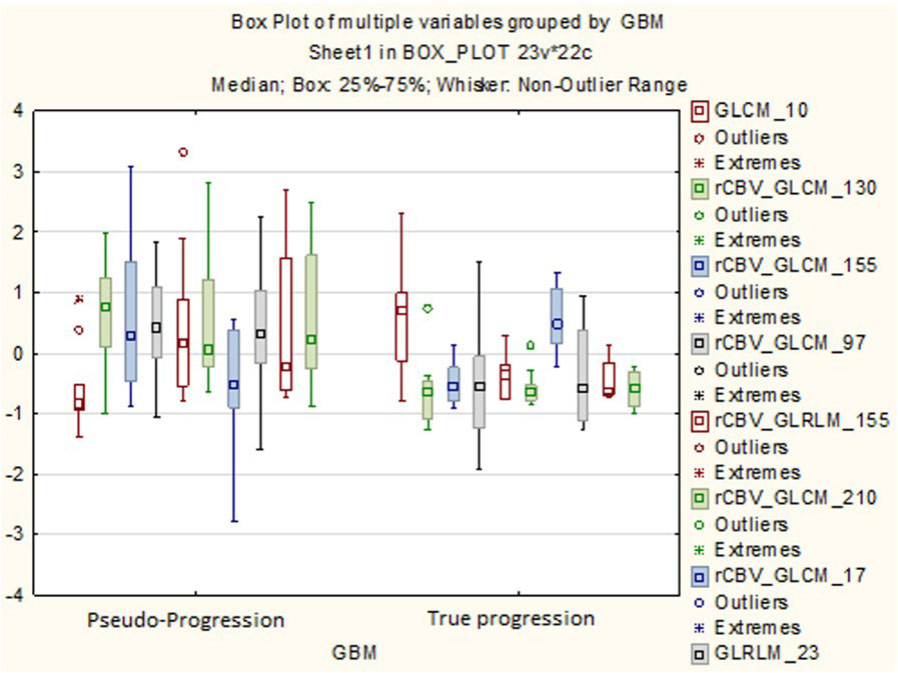
See text for description

#### P431 Radiomic Analysis differentiates between True Progression and Pseudo-progression in Glioblastoma patients: A large scale multi-institutional study

##### Srishti Abrol^1^, Aikaterini Kotrotsou^1^, Nabil Elshafeey^1^, Islam Hassan^1^, Ahmed Hassan^1^, Tagwa Idris, MD^1^, Kamel El Salek, MD^1^, Ahmed Elakkad, MD^1^, Kristin Alfaro-Munoz^1^, Shiao-Pei Weathers^1^, Fanny Moron^2^, John deGroot^1^, Meng Law^3^, Rivka Colen, MD^1^

###### ^1^MD Anderson Cancer Center, Houston, TX, USA; ^2^Baylor College of Medicine, Houston, TX, USA; ^3^University of Southern California, Los Angeles, CA, USA; ^4^The University of Texas, Houston, TX, USA

####### **Correspondence:** Rivka Colen (rcolen@mdanderson.org)

**Background**

Treatment-related changes can occur as a result of multiple factors; these changes are often difficult to distinguish from true progression (PD) of the tumor using conventional MRI. Treatment-related changes or pseudoprogression (PsP) subsequently subside or stabilize without any further treatment, whereas progressive tumor requires a more aggressive approach. PsP mimics PD radiographically and may potentially alter the physician’s judgement. Hence, it can predispose a patient to overtreatment or be categorized as a non-responder and exclude him from clinical trials. Radiomic analysis results in the quantification of grey tone spatial variation thereby providing textural features that characterize the underlying structure of the object under investigation. This study aims at assessing the potential of radiomics to discriminate PsP from PD in glioblastoma (GBM) patients.

**Methods**

In this multi-institutional study, we evaluated 304 GBM patients retrospectively. All patients showed radiographic worsening in MRI, with/without clinical deterioration, and were evaluated for PD our PSP. 149 patients had histopathological evidence of PD and 27 of PsP. Remaining 128 patients were categorized into PD or PsP based on RANO criteria . Conventional MR images were acquired using typical clinical acquisition parameters. Three tumor phenotypes (ROIs), namely edema/invasion, necrosis, and enhancing tumor, were delineated by an experienced radiologist. A total of 1800 radiomic features were obtained for each patient. Statistical Analysis: An advanced feature selection method based on Minimum Redundancy Maximum Relevance (MRMR) was used to analyze the featureset and extract core features. Selected features were used to build a Support Vector Machine (SVM) model for prediction of PD versus PsP status. To evaluate the robustness of the estimates made with the SVM models, leave-one-out-cross-validation (LOOCV) and a 70-30% split was performed.

**Results**

Using the MRMR feature selection method, we could identify 100 significant features that were further used to build a SVM model. On LOOCV, the area under curve (AUC) was 90%, with a sensitivity and specificity of 97% and 72% respectively (Figure 3). Using 70% of the patient data for training and 30% for validation an AUC of 94% was achieved, with sensitivity of 97% and specificity of 75%. Five texture features i.e. energy, cluster shade, sum average, maximum probability and cluster prominence were found to be most predictive of nature of disease progression.

**Conclusions**

The proposed tool has the potential to advance clinical management strategies. Apart from its non-invasive nature, our methodology doesn’t require additional imaging and may act as a complementary tool for the clinicians.

**Fig. 1 (abstract P431).**
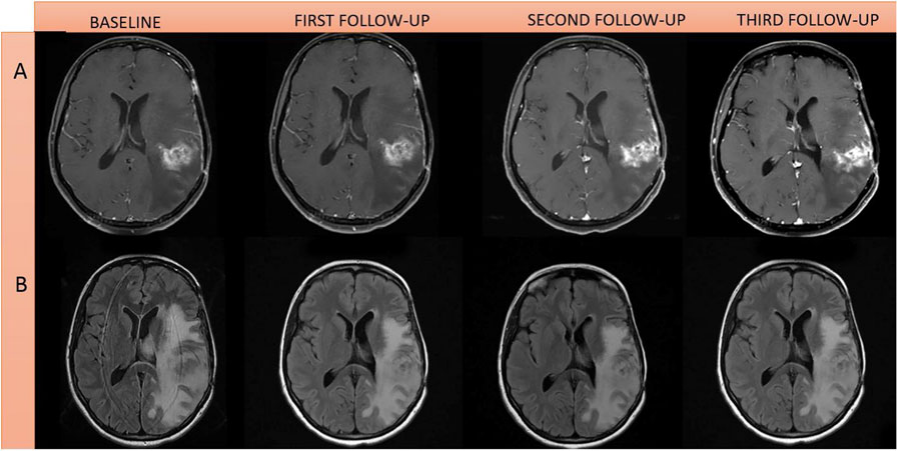
See text for description

**Fig. 2 (abstract P431).**
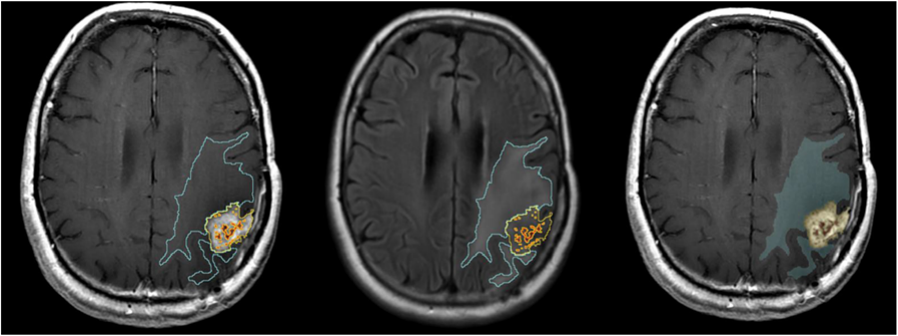
See text for description

**Fig. 3 (abstract P431).**
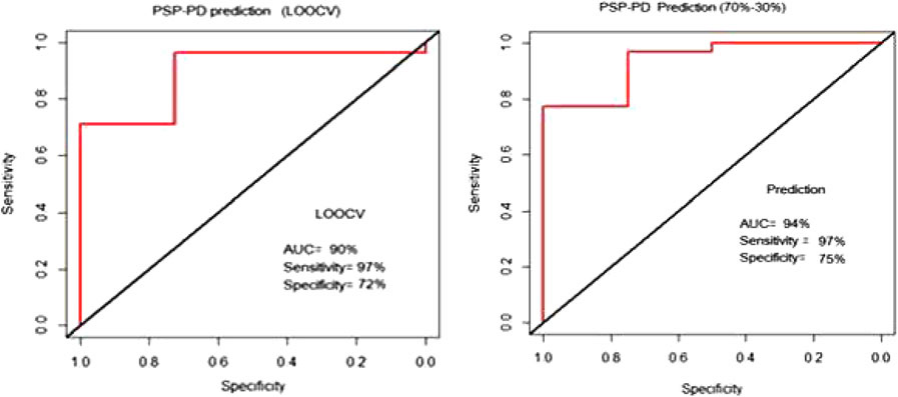
See text for description

#### P432 High tumor mutation burden (Hypermutation) in gliomas exhibit a unique predictive radiomic signature

##### Islam Hassan^1^, Aikaterini Kotrotsou^1^, Carlos Kamiya Matsuoka^1^, Kristin Alfaro-Munoz^1^, Nabil Elshafeey^1^, Nancy Elshafeey^1^, Pascal Zinn^2^, John deGroot^1^, Rivka Colen, MD^3^

###### ^1^MD Anderson Cancer Center, Houston, TX, USA; ^2^Baylor College of Medicine, Houston, TX, USA; ^3^The University of Texas, Houston, TX, USA

####### **Correspondence:** Rivka Colen (rcolen@mdanderson.org)

**Background**

Increase in tumor mutation burden (TMB) or hypermutation is the excessive accumulation of DNA mutations in cancer cells. Hypermutation was reported in recurrent as well as primary gliomas. Hypermutated gliomas are mostly resistant to alkylating therapies and exhibit a more immunologically reactive microenvironment which makes them a good candidate for immune checkpoint inhibitors. Herein, we sought to use MRI radiomics for prediction of high TMB (hypermutation) in primary and recurrent gliomas.

**Methods**

In this IRB-approved retrospective study, we analyzed 101 patients with primary gliomas from the University of Texas MD Anderson Cancer Center. Next generation sequencing (NGS) platforms (T200 and Foundation 1) were used to determine the Mutation burden status in post-biopsy (stereotactic/excisional). Patients were dichotomized based on their mutation burden; 77 Non-hypermutated (<30 mutations) and 24 hypermutated (>=30 mutations or <30 with MMR gene or POLE/POLD gene mutations). Radiomic analysis was performed on the conventional MR images (FLAIR and T1 post-contrast) obtained prior to tumor tissue surgical sampling; and rotation-invariant radiomic features were extracted using: (i) the first-order histogram and (ii) grey level co-occurrence matrix. Then, we performed Logistic regression modelling using LASSO regularization method (Least Absolute Shrinkage and Selection Operator) to select best features from the overall features in the dataset. ROC analysis and a 50-50 split for training and testing, were used to assess the performance of logistic regression classifier and AUC, Sensitivity, Specificity, and p-value were obtained. (Figure 1)

**Results**

LASSO regularization (alpha = 1) was performed with all the 4880 features for feature selection and 40 most prominent features were selected for logistic regression modelling. Our entire dataset ROC analysis showed an accuracy of 100%, sensitivity of 100% and specificity of 100% with p-value of 1.256-12, while our 70-30 split ROC analysis showed an accuracy of 96.7%, sensitivity of 85% and specificity of 100% and a p-value of 0.003; Our 50- 50 split ROC analysis showed an accuracy of 94%, sensitivity of 75%, and specificity of 100% and a p-value of 0.0008. (Figure 2, 3, 4)

**Conclusions**

An MRI-radiomic phenotype is predictive of the increase in TMB (Hypermutation) in both primary and recurrent gliomas.

**Fig. 1 (abstract P432).**
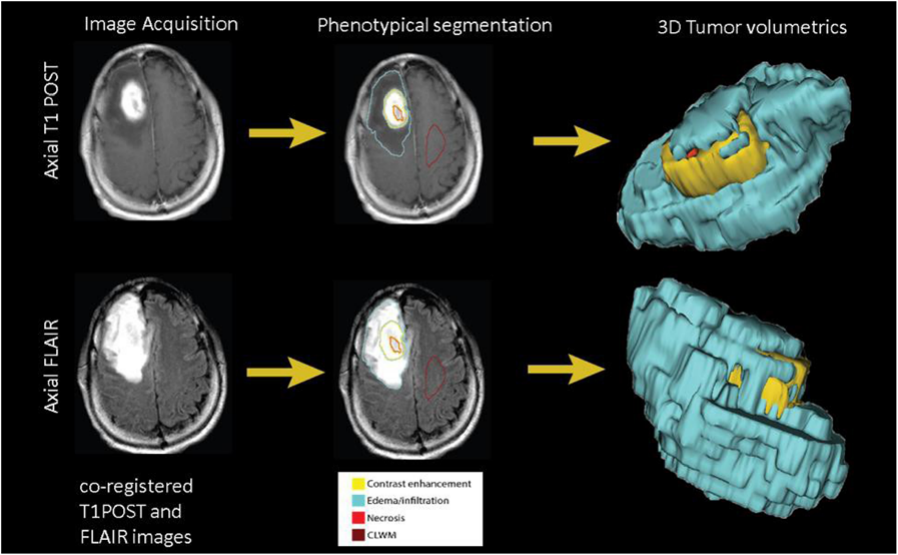
See text for description

**Fig. 2 (abstract P432).**
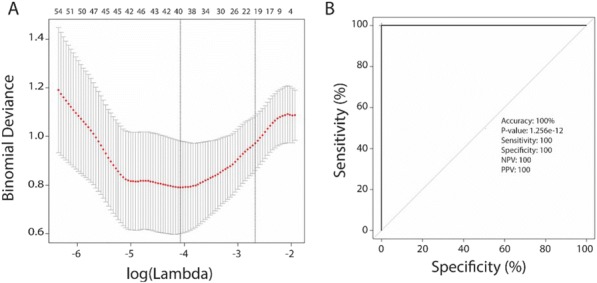
See text for description

**Fig. 3 (abstract P432).**
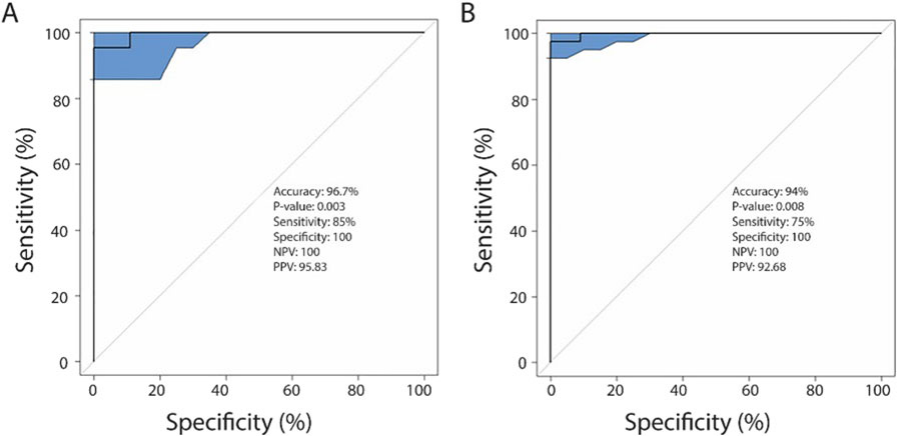
See text for description

**Fig. 4 (abstract P432).**
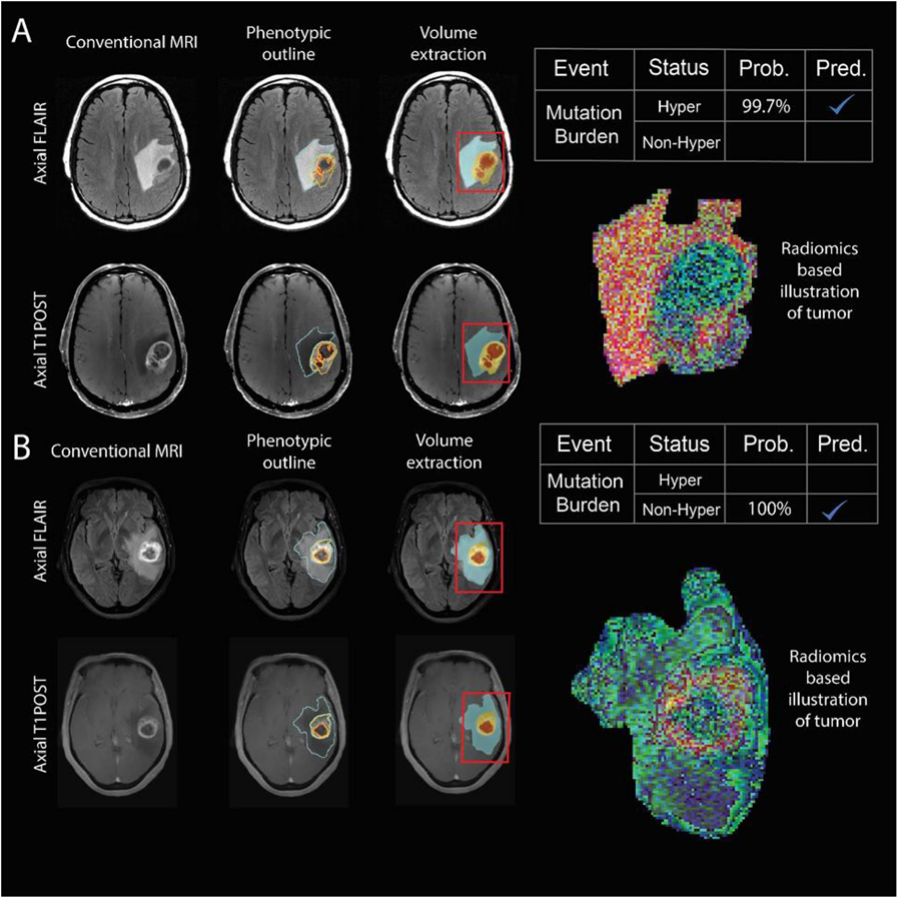
See text for description

#### P433 Advances in multiplex fluorescence immunohistochemistry: 9 color imaging; whole slide multispectral

##### Carla Coltharp, PhD, Yi Zheng, PhDRachel Schaefer, Ryan Dilworth, PhD, Linying Liu, Chichung Wang, Kristin Roman, MS, Clifford Hoyt, MS, Peter Miller, MS

###### PerkinElmer, Inc., Hopkinton, MA, USA

####### **Correspondence:** Peter Miller (peter.miller@perkinelmer.com)

**Background**

We describe two advances in multispectral fluorescence immunohistochemistry, a powerful tool for quantifying interactions within the tumor microenvironment. First, a fully-automated 8-plex assay plus DAPI counterstain on the same tissue section. Second, a novel scanning method that produces a multispectral whole slide scan of 6 markers plus DAPI counterstain in ~6 minutes (1x1.5 cm tissue section).

**Methods**

FFPE primary tumors were immunostained using Opal™ reagents manually or on a Leica BOND RX™. Imagery was acquired on a Vectra Polaris® automated imaging system and analyzed with inForm® and MATLAB® software.

**Results**

Two new Opal™ reagents (Opal 480 and Opal 780) were combined with currently available Opal 7-color kits to stain and distinguish 8 markers plus DAPI when imaged on the Vectra Polaris®.Figure 1 shows a 9-color panel on lung cancer: CD20 (Opal 480), PD-L1 (Opal 520), CD8 (Opal 540), FoxP3 (Opal 570), CD68 (Opal 620), PD-1 (Opal 650), Ki67 (Opal 690), and PanCK (Opal 780). Colors assigned to each marker, and associated component planes, are shown in Figure 1B.These 8 markers combine to generate more than 20 phenotypes relevant to immuno- oncology that can be studied in relation to local PD-L1 expression and proliferation state (Ki67+/-). For example, while the density of CD8+ cells was 8-fold lower in tumor than stroma (150 vs 1200 cells/mm^2), those CD8+ cells were >4x more likely to be proliferating in tumor vs stroma (28% vs. 6%).To interrogate interactions across a whole section, we additionally developed a multispectral whole-slide scanning method, demonstrated on lung cancer using a subset of 7 stains from the 9-color panel above. Phenotype and expression-level assessments of the unmixed whole slide scan describe distribution patterns of immune cells across the entire section.In measurements of crosstalk and dynamic range, whole-slide multispectral scanning performed comparably to established field-based multispectral imaging, and outperformed conventional fluorescence scanning by reducing crosstalk from up to 8% to under 2% (typically <0.5%) and extending the dynamic range of some channels by more than 50-fold.

**Conclusions**

We introduce a 9-color fIHC assay that distinguishes 8 markers plus DAPI counterstain on the same tissue section, increasing the depth of cellular interactions that can be studied within the tumor microenvironment.Additionally, we introduce a whole slide multispectral imaging method that provides rich quantitation of interactions among 6 markers at length scales spanning from cell biology to tumor physiology.

**Fig. 1 (abstract P433).**
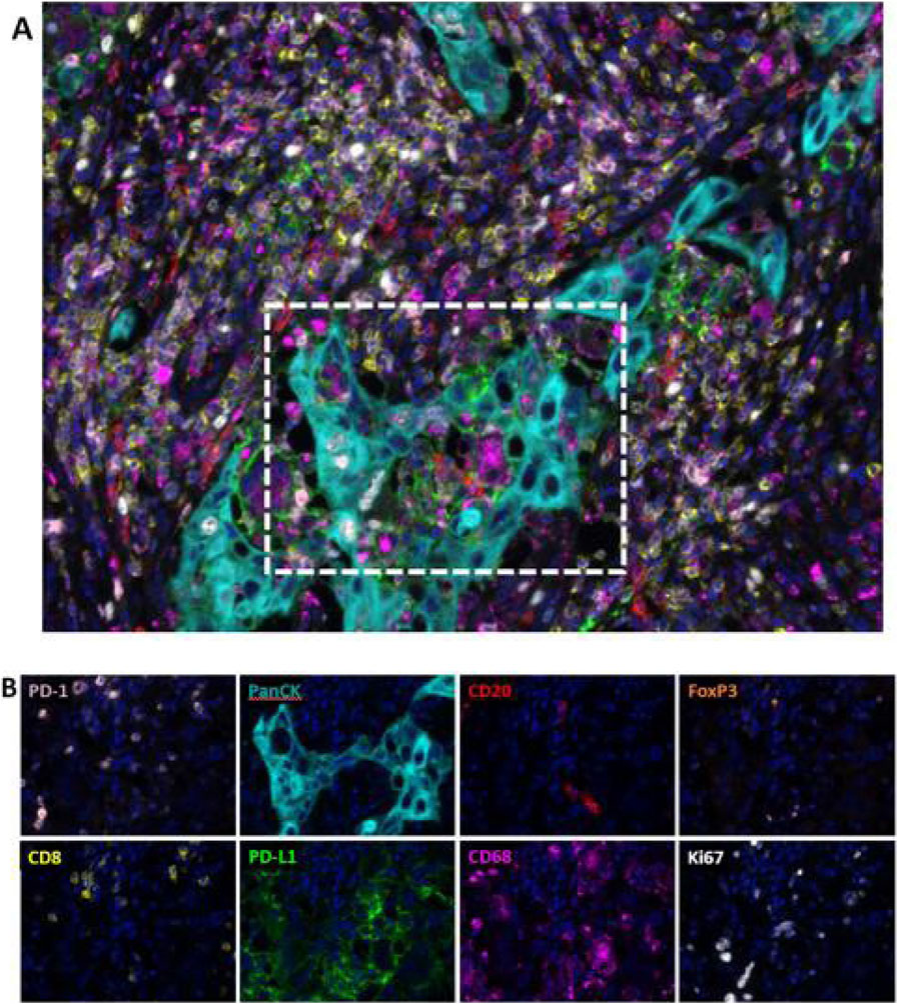
See text for description

#### P434 Mathematical modeling of CAR T cell therapy outcomes to develop design specifications for CAR T cell engineering

##### Amritava Das, PhD^1^, Rachel Grosser, undergraduate^2^, Ambar Velazquez Albino, BS Student^3^, Krishanu Saha^2^, Christian M. Capitini, MD^2^

###### ^1^Morgridge Institutes for Research, Madison, WI, USA; ^2^University of Wisconsin - Madison, Madison, WI, USA; ^3^University of Puerto Rico - Mayaguez, Mayaguez, PR, USA; ^4^Morgridge Institute for Research, Madison, WI, USA

####### **Correspondence:** Christian M. Capitini (ccapitini@pediatrics.wisc.edu)

**Background**

Chimeric antigen receptor (CAR) T cell therapy has demonstrated success in clinical trials [1], and two such therapies have now been approved within the USA [2]. Due to the heterogeneity of apheresis products from heavily treated cancer patients, no algorithms exist to predict the efficacy of manufactured CAR T cell products. CAR T cells are living drugs, that are capable of division, anti-tumor cytotoxicity and cytokine secretion post infusion. Based on previous models of virus-T cell interaction [3], we developed new models to estimate post-infusion CAR T cell division and cytotoxicity. Simulation results reveal important characteristics when elite populations of CAR T cells are present in the pool of infused CAR T cells.

**Methods**

Models were implemented in COPASI [4], a biochemical network simulation platform. Patient CAR T cell performance data extracted from previously published studies using WebPlotDigitizer [5]. Fitting of model parameters to published patient data and model inference performed using ABC-SysBio [6], a python-based toolkit implementing Approximate Bayesian Computation. Post-processing of outputs from COPASI and ABC-SysBio was performed on MATLAB.

**Results**

Any of the models developed (selection shown in Figure 1) could be fit to patient data, and ABC-SysBio can be implemented to select between the models given patient data. Model presented in figure 1A was used to determine the effects of having a large population of CAR T cells which can only undergo one cell division and a smaller elite population (1/1000th of maximum at infusion) capable of unlimited expansion. Broadly, the rates of division of high performance clonal CAR T cells (at most 4 h doubling time), and the rates of memory formation of CAR T cells (at least 0.383/day) were found to most significantly impact tumor clearance, while the cytotoxicity of the CAR T cells (ranging from 2 – 16 /day/cell) did not significantly impact tumor clearance in the mathematical models (Figure 2).

**Conclusions**

Surprisingly memory formation is more associated with complete remission than cytotoxicity and mirrors previous findings that correlate therapeutic success with memory formation [7]. Estimation of the parameter values for number of CAR T cell divisions, rates of division, memory formation, memory reactivation, CAR T cell depletion (exhaustion and non-exhaustion induced death) and anti-tumor cytotoxicity can be useful in determining the design specifications of successful CAR T cell therapy administrations across various clinical trials. Extrapolation of this model in a prospective setting will be needed for further validation.

**References**

1. Gill S, Maus MV,Porter DL. Chimeric antigen receptor T cell therapy: 25years in the making. Blood Rev. 2016; 30(3): 157-67.

2. June, CH, Sadelain M. Chimeric Antigen Receptor Therapy. N Engl J Med. 2018; 379(1): 64-73.

3. Wodarz D,Thomsen AR. Effect of the CTL proliferation program on virus dynamics. International Immunology. 2018; 17(9): 1269-1276.

4. Hoops S, et al. COPASI--a COmplex PAthway SImulator. Bioinformatics. 2006; 22(24): 3067-74.

5. Burda, BU, et al. Estimating data from figures with a Web-based program: Considerations for a systematic review. Res Synth Methods. 2017; 8(3): 258-262.

6. Liepe J, et al. ABC-SysBio--approximate Bayesian computation in Python with GPU support. Bioinformatics. 2010; 26(14): 1797-9.

7. Fraietta JA, et al. Determinants of response and resistance to CD19 chimeric antigen receptor (CAR) T cell therapy of chronic lymphocytic leukemia. Nat Med. 2018; 24(5): 563-571.

**Fig. 1 (abstract P434).**
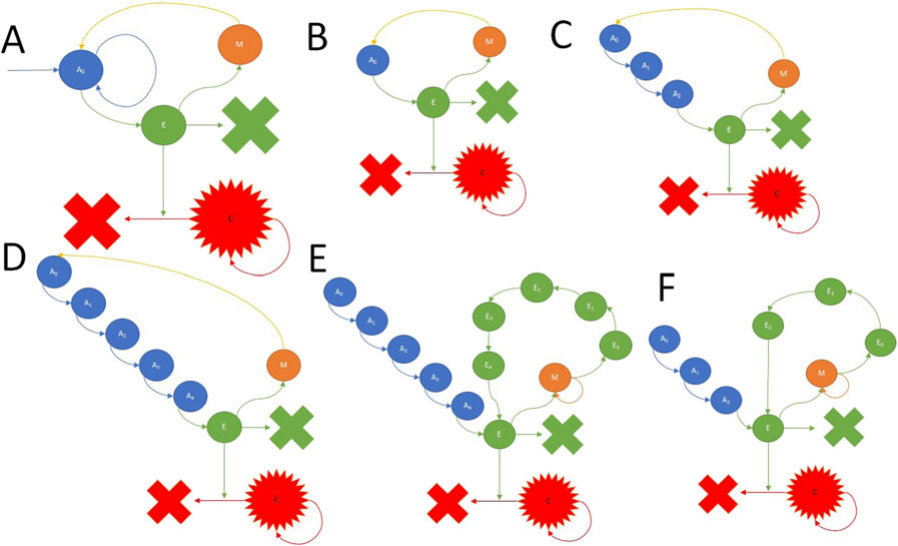
Mathematical modeling frameworks developed

**Fig. 2 (abstract P434).**
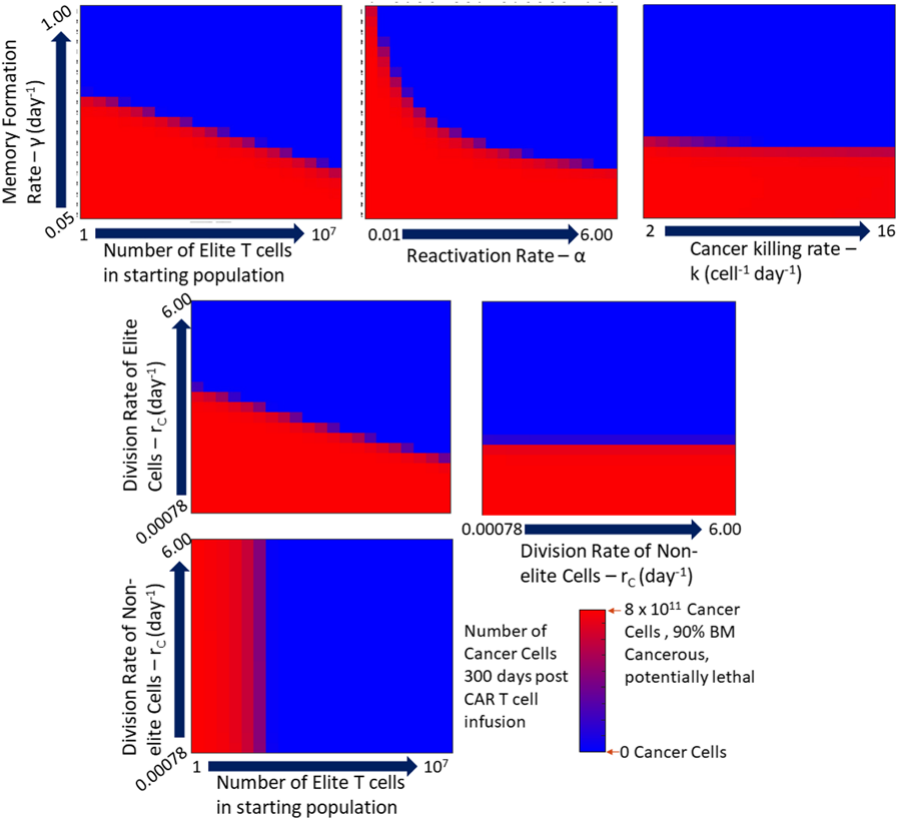
Heatmaps indicating the number of cancer cells

#### P435 Image-based analysis of the myeloid cell landscape in the 3D co-culture with tumor cells

##### Gera Goverse, PhD^1^, Kuan Yan, PhD^1^, Lars Geulen^2^, Paul Vink, BS^2^, Leo Price^1^, Lidia Daszkiewicz, PhD^1^

###### ^1^OcellO B.V., Leiden, Netherlands; ^2^Aduro Biotech, Oss, Netherlands

####### **Correspondence:** Gera Goverse (gera.goverse@ocello.nl)

**Background**

The myeloid cell compartment plays an important role in anti-tumor immune responses and represents a heterogeneous population with both cancer-promoting and cancer-restraining actions. Unleashing the full potential of cancer immunotherapies requires an understanding of the cellular mechanisms that govern these opposite actions. To date, high throughput relevant preclinical models for dissecting the interactions between different cellular players in the tumor microenvironment are lacking. Previously we have shown that our 3D image-based co-culture system allows assessing efficacy of immune modulators to enhance PBMC infiltration and tumoroid killing. Our main goal was to improve this model by incorporating a more complete human immune system. To do that we first generated diverse myeloid populations in a 3D environment and then used our image-based platform to describe the different subsets. The image analysis software was trained on a set of features that reproducibly allowed discrimination between undifferentiated monocytes, M1 and M2 macrophages and dendritic cells. The different myeloid subsets were next co-cultured with tumor cells to analyze the complex cellular interplay of the TME.

**Methods**

Different myeloid populations were generated in 3D from monocytes derived from healthy donors PBMCs. Polarized M1 and M2 macrophages, DCs and undifferentiated monocytes were then co-cultured in 3D with SKBR3 tumor cells or 3D tumoroids derived from this cell line. The cellular interactions were visualized using high-content microscopy and quantified with multiparametric morphometric analysis with OMinerTM software.

**Results**

3D image analysis enabled the discrimination of immune-tumor cell interactions and revealed the effect of myeloid cells on tumor growth in co-culture. Our approach also enables the analysis of how tumor-driven mechanisms regulate myeloid cell differentiation and contribute to the immunosuppressive microenvironment. These results provide a means to elucidate the bi-directional interplay between tumor and immune cells and allows for analysis of functional reprograming of the suppressive population towards a M1 phenotype induced by drug candidates.

**Conclusions**

The 3D assay presented here enables visualization and measurement of effects of immunotherapies on cells that engage in a more physiologically relevant spatial setting than when culturing them in traditional 2D cultures. Using morphological measurements different myeloid cell subsets can be distinguished, which offers a very attractive alternative for complex and labor-intensive phenotyping based on markers expression and cytokine release profiling. The ultimate goal is to develop a highly sophisticated platform for testing cancer immunotherapies that combines the complexity of the TME and the robustness of a high throughput screening platform.

#### P436 Image analysis simulations of needle biopsy tumor specimens to investigate CD8+ TIL heterogeneity

##### Thomas Herz, PhD^1^, Victor Matvienko^1^, Tobias Wiestler, PhD^1^, Rene Korn, PhD^1^, Keith Steele, DVM, PhD^2^

###### ^1^Definiens AG, Munich, Germany; ^2^MedImmune, Gaithersburg, MD, USA

####### **Correspondence:** Thomas Herz (therz@definiens.com)

**Background**

Core needle biopsies are used to histologically assess tumors when surgical excision is impractical. Such small samples may not be representative given the known heterogeneity of immune cell distribution, including CD8+ tumor infiltrating lymphocytes (TILs)[1].

**Methods**

Initially, 20 immunolabeled slides from purchased non-squamous NSCLC tumor resections were scanned and tumor region was manually annotated[1]. CD8(+) TILs were detected using Definiens Developer XD™ software[1,2]. Needle biopsies were simulated using an elliptical shape, with multiple iterations applied by varying the size, angle and positioning of that ellipse across the full resection using Python programming language[3], totaling in 24,200 single needle simulations per case. CD8(+) TIL density was determined for the tumor region contained within each simulated portion. Using the statistical software R[4], individual cores were compared to other cores in each sample, to the full tumor region and across all 20 cases.

**Results**

The heterogeneity of the CD8(+) TIL distribution is very well reflected in the statistical analysis of the number of CD8(+) TILs actually found within the needle biopsy to the expected number, based on the size of the needle ellipse and full slide CD8(+) TIL density. Even in cases with generally high correlation, a single biopsy location with changing the needle size or the angular component of the needle direction only can already produce a set of non- representative CD8(+) TIL densities. In a about 15% of all simulated cores, no CD8(+) TIL was found in the tumor region, spanning all dimensions of variation used in the simulation equally as well as cases.

**Conclusions**

One needle biopsy insufficiently represents the CD8+ TIL density of resected non-squamous NSCLC. Determining a clinically-feasible number of cores to accurately assess CD8 requires further study. Systematic measurement of sampling error should be extended to other markers of the immune response to cancer whose expression is known to be heterogenous, such as PD-L1.

**References**

1. Steele K, Tan TH, Korn R. Measuring multiple parameters of CD8+ tumor-infiltrating lymphocytes in human cancers by image analysis. J Immunother Cancer 2018;6:202. Baatz M, Zimmermann J, Blackmore CG. Automated analysis and detailed quantification of biomedical images using Definiens Cognition Network Technology®. Combinatorial Chemistry & High Throughput Screening 2009;12:908–163. Python Software Foundation [https://www.python.org]4. R Core Team (2017). R: A language and environment for statistical computing. R Foundation for Statistical Computing, Vienna, Austria. [https://www.R-project.org]

#### P437 In vivo synergistic effect of checkpoint blockade and radiation therapy against chordomas in a humanized mouse model

##### Wataru Ishida, MD^1^, Kyle McCormick, BA^2^, Aayushi Mahajan, MS^2^, Eric Feldstein, BS^2^, Michael Lim, MD^1^, Jeffrey Bruce^2^, Peter Canoll, MD PhD^2^, Sheng-fu L. Lo, M.D.^1^

###### ^1^Johns Hopkins University, Baltimore, MD, USA; ^2^Columbia University Medical Center, New York, NY, USA

####### **Correspondence:** Sheng-fu L. Lo (larrylo@jhmi.edu)

**Background**

It has been a challenge to apply immunotherapy (IT) to patients with chordomas, due to lack of clinically-translatable *in vivo* models. Currently, there are no well-established murine chordoma cell lines that can be injected to syngeneic mice or no transgenic mouse models that develop chordomas spontaneously, which would allow us to study the interaction between murine chordomas and murine immune cells. Hence, we aimed to develop a humanized mouse model, where human immune cells are engrafted into immunodeficient mice,[1,2] to overcome this limitation by studying the interaction between human immune system and human chordomas. We also sought to utilize it to study synergistic effect between IT and radiation therapy (RT) against chordoma.

**Methods**

Fifteen 10-12-week-old NSG mice were sub-lethally (1.5Gy) irradiated and then implanted with fetal thymic tissue and CD34+ stem cells that had been harvested from a fetus, whose HLA-types were partially-matched with those of the U-CH1 chordoma cell line. Reconstitution of immune cells in NSG mice was confirmed 8 weeks post- transplantation and then each animal (15 humanized NSG mice and 12 naïve NSG mice) was injected with U-CH1 cell suspension bilaterally and subcutaneously. Next, they were treated for 4 weeks as follows: A) control, isotype antibodies (Abs) injection (n=3), B) anti-human-PD-1 Abs (n=4), C) RT + isotype Abs (n=3, unilaterally to the left- sided tumor, 8Gy x 4), D) anti-human-PD-1 Abs and RT (n=5), E) naïve NSG mice (n=6, without the engraftment of human immune cells) + isotype, and F) naïve NSG mice (n=6) + anti-human-PD-1 Abs. During and after the treatment, anti-tumor activities were monitored via tumor size, flow cytometry, qRT-PCR, and immunohistochemistry.

**Results**

One week after the treatment, on the irradiated side, (D) demonstrated lowest tumor volume (Figure 1), highest number of human PBMCs, highest % of CD8+ human T cells, highest % of CD45RO+CD4+ human (memory) T cells, and lowest % of PD-1+CD8+ human T cells in the tumors via flow cytometry (Figure 2), and highest IFN- gamma in the tumors via qRT-PCR, compared to the other five groups with statistical significance. On the non- irradiated side, similarly D) had the smallest tumor compared to the others (P=0.09).

**Conclusions**

We demonstrated that this humanized mouse model could be a revolutionary platform to investigate IT against rare cancers such as chordomas, where murine equivalent cell lines are currently unavailable. The direct synergistic effect between IT and RT against chordoma as well as the potential abscopal effect was observed.

**Acknowledgements**

We would like to thank all members of Herbert Irving Comprehensive Cancer Center at Columbia University Medical Center for generous support and its shared resource as well as CCTI, especially Drs. Hui Wang and Yong- Guang Yang at the CCTI humanized mouse core as well as Dr. Siu-Hong Ho, the director of the CCTI flow cytometry core and Assistant Professor of Medical Sciences. We also would like to thank The Sidney Kimmel Comprehensive Cancer Center at Johns Hopkins University and its oncology shared resources, particularly Drs. Alan Meeker and Sujayita Roy. These studies used the resources of the Herbert Irving Comprehensive Cancer Center Flow Cytometry Shared Resources funded in part through Center Grant P30CA013696. Research reported in this publication was performed also in the CCTI Flow Cytometry Core, supported in part by the Office of the Director, National Institutes of Health under awards S10RR027050. The content is solely the responsibility of the authors and does not necessarily represent the official views of the National Institutes of Health.

**References**

1. Kalscheuer H, Danzl N, Onoe T, et al. A model for personalized in vivo analysis of human immune responsiveness. Sci Transl Med. 2012;4(125):125ra130.2. Zitvogel L, Pitt JM, Daillere R, Smyth MJ, Kroemer G. Mouse models in oncoimmunology. Nat Rev Cancer. 2016

**Ethics Approval**

This study was approved by Columbia IACUC, protocol number AAAQ8458.

**Fig. 1 (abstract P437).**
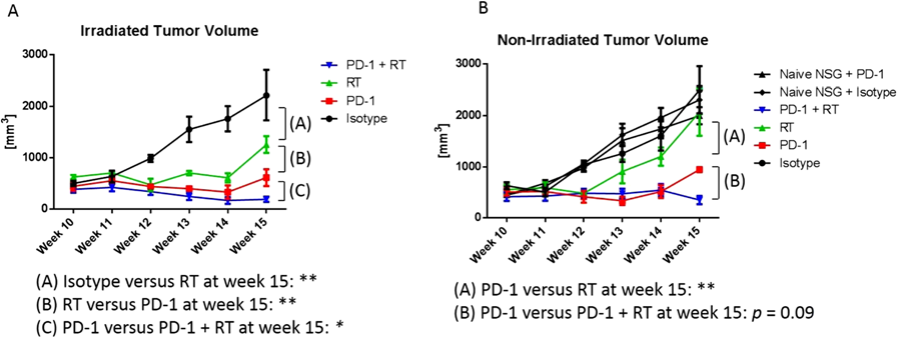
See text for description

**Fig. 2 (abstract P437).**
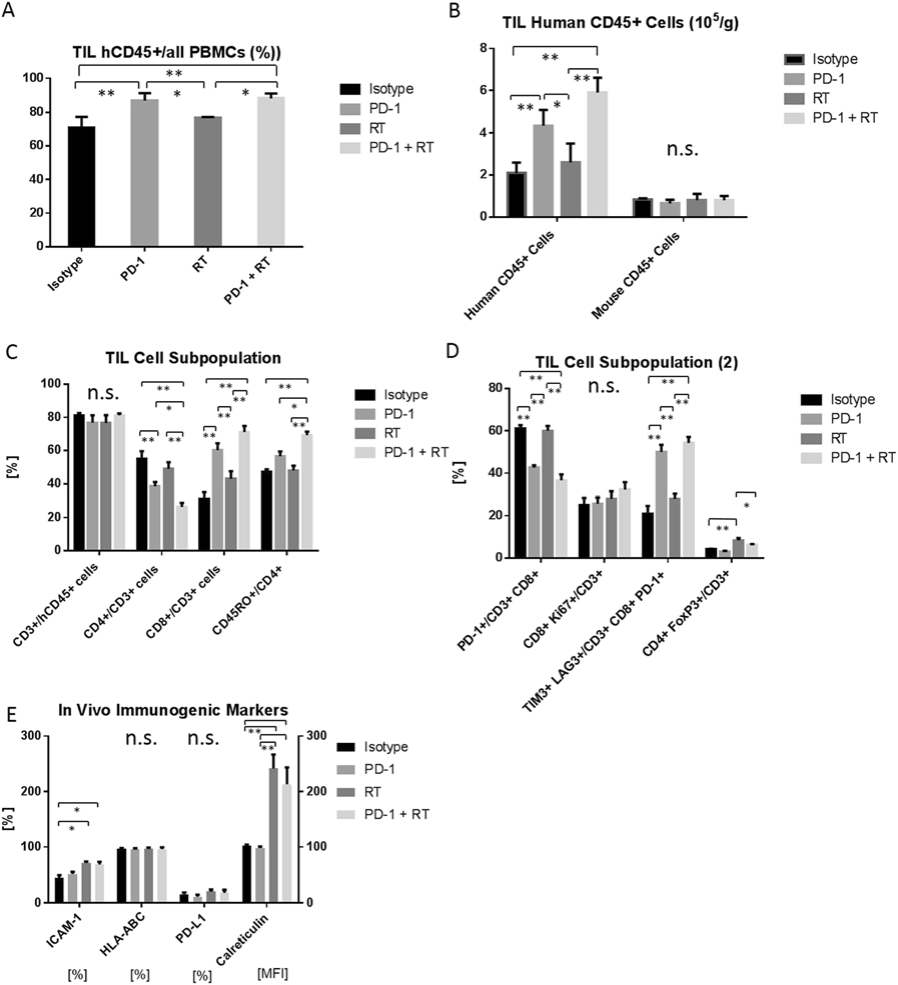
See text for description

#### P438 Effect of CD3 affinity and normal tissue expression on the biodistribution and tumor targeting of MUC16xCD3 bispecific antibodies in MUC16 and CD3 humanized mice

##### Marcus Kelly, PhD, Alison Crawford, PhD, Jason Giurleo, PhD, Richard Tavaré, PhD, Sosina Makonnen, Carlos Hickey, Makenzie Danton, Cody Arnold, Lauric Haber, Eric Smith, PhD, Dangshe Ma, William Olson, PhD, Gavin Thurston, PhD, Jessica Kirshner, PhD

###### Regeneron Pharmaceuticals Inc., Tarrytown, NY, USA

####### **Correspondence:** Marcus Kelly (marcus.kelly@regeneron.com)

**Background**

The tumor associated glycoprotein MUC16 is highly expressed in ovarian cancer with limited normal tissue expression, making it a suitable target for the development of CD3 binding T-cell engaging bispecific antibodies. Here we used non-invasive immuno-PET imaging as a powerful tool to determine the impact of each antigen binding arm on bio-distribution of MUC16-CD3 bispecific antibodies in mice. To dissect the role of CD3 affinity on antibody distribution, we assessed two bispecifics with varying CD3 affinity; MUC16-CD3_low_ and MUC16-CD3_high_^,^ alongside the bivalent parental MUC16 antibody.

**Methods**

Antibodies were radiolabeled with positron emitting radionuclide Zirconium-89 (^89^Zr) using the chelator deferoxamine (DFO) and demonstrated high radiochemical purity and immunoreactivity. Initial imaging and biodistribution studies were performed in SCID mice bearing MUC16+ OVCAR3 ovarian tumor xenografts to validate the MUC16 binding arm of the antibodies. Localization of ^89^Zr-MUC16-CD3_low_ and ^89^Zr-MUC16-CD3_high_ was next measured in tumor-free MUC16 and CD3 double humanized immunocompetent mice. A subsequent study assessed blocking CD3-dependent localization of ^89^Zr-MUC16-CD3_high_ by a control CD3 antibody. Parental ^89^Zr- MUC16-MUC16 antibody was assessed in matched humanized and normal mice to determine localization to MUC16 expressing normal tissues. Lastly, the uptake of ^89^Zr-MUC16-CD3_low_ or ^89^Zr-MUC16-CD3_high_ to ID8/VEGF/hMUC16 tumors was assessed in the double humanized mice.

**Results**

Immuno-PET imaging of 89Zr-MUC16-CD3_low_, ^89^Zr-MUC16-CD3high and ^89^Zr-MUC16-MUC16 all demonstrated high and specific targeting to OVCAR3 xenografts (~70%ID/g). In the MUC16 and CD3 humanized mice, very high localization of ^89^Zr-MUC16-CD3_high_ to CD3+ lymphoid tissues (spleen and lymph nodes) was observed. Relative ^89^Zr-MUC16-CD3_low_ uptake in lymphoid tissues was greatly reduced. Conversely, blood levels of ^89^Zr- MUC16-CD3_high_ were lower than ^89^Zr-MUC16-CD3_low_, resulting in higher tissue:blood ratios by ^89^Zr-MUC16- CD3_high_. Blocking with control CD3 bispecific significantly reduced localization of ^89^Zr- MUC16-CD3_high_ to lymphoid tissues. Specific uptake of ^89^Zr-MUC16-MUC16 in normal tissues was not observed. ^89^Zr-MUC16-CD3_low_ and 89Zr-MUC16-CD3_high_ both showed significant uptake (50-60%ID/g) in ID8/VEGF/hMUC16 tumors. Tumor uptake between the antibodies was generally not significantly different despite the high lymphoid uptake of ^89^Zr- MUC16-CD3_high_.

**Conclusions**

^89^Zr-MUC16-CD3_low_ and ^89^Zr-MUC16-CD3high demonstrated specific localization to MUC16+ tumors and CD3+ lymphoid tissues, with lymphoid distribution correlating to relative CD3 affinity. Both MUC16-CD3 bispecifics demonstrated clear tumor localization in the presence of CD3+ tissues. This work demonstrates that immuno-PET is an ideal technology to monitor bispecific localization in vivo. Further studies may investigate any correlation between antibody biodistribution as monitored by immuno-PET and toxicity or efficacy observed during the optimization of these promising therapeutics.

#### P439 A novel high-throughput, high-content real-time imaging platform to assess immunogenic cell killing activity of immunotherapeutic agents using patient-derived tumor samples

##### Jenny Kreahling, PhD, Melanie Mediavilla-Varela, PhD, Melba Marie Page, PhD, Soner Altiok, MD, PhD

###### Nilogen Oncosystems, Tampa, FL, USA

####### **Correspondence:** Jenny Kreahling (jenny@nilogen.com)

**Background**

Immuno-oncology has revolutionized cancer care for many cancer types, however, the development of novel immunotherapeutics still faces many challenges due to lack of drug screening platforms that represent the complexity of the tumor microenvironment. Conventional cytotoxicity assays, such as chromium 51 and LDH release are limited in providing clinically relevant data about immunogenic cell death. The goal of this study was to develop an integrated confocal-based high-throughput, high-content real-time imaging platform to assess immunogenic cell killing activity of novel immunotherapeutic agents and to develop rational drug combinations using patient-derived tumor samples.

**Methods**

All patient tumor samples were obtained with patient consent and relevant IRB approval. For the confocal imaging platform, unpropagated 3D tumoroids measuring 100-150 micron in size were prepared from fresh tumor samples of non-small cell lung cancer using a proprietary technology developed at Nilogen Oncosystems. Cell-match studies utilized autologous patient-derived cell lines that were isolated and propagated from each patient’s tumor.

**Results**

In Cell-match studies, tumor cells and tumor infiltrating lymphocytes (TILs) were labeled with different cell tracker fluorescent dyes to monitor cell movements and locations. For 3D tumoroid assays samples were pre-labeled with proprietary fluorogenic markers to identify live and dead tumor cells. After treatment with different immune- stimulatory agents, real-time confocal imaging analysis was performed to assess apoptotic tumor cell death which was evaluated via the detection of changes in the permeability of cell membranes and activation of caspase 3 pathway. Comprehensive flow cytometry analysis was performed to corroborate confocal imaging findings on immunogenic tumor cell death (LIVE/DEAD viability markers and cleaved caspase 3) and TIL activation (CD25, CD69, Ki-67 and granzyme expression in CD4 and CD8 positive lymphocytes). A custom image analysis algorithm was developed for the collection of data in a structurally relevant environment on quantification of marker-specific cell number, cell viability and apoptosis in addition to structural and functional analysis of cells in intact 3D tumoroids.

**Conclusions**

The confocal-based high-throughput and high-content real-time imaging platform described here is physiologically relevant and allows rapid screening of multiple drugs and drug combinations based on their immunogenic cell killing activity in a cost-effective manner to accelerate drug discovery.

#### P440 Open-source digital image analysis of whole-slide multiplex immunohistochemistry

##### Nikhil Lonberg, HSDG, Nikhil Lonberg, HSDG, Nikhil Lonberg, HSDG, Carmen Ballesteros Merino, PhD, Shawn Jensen, PhD, Bernard Fox, PhD

###### Robert W Franz Cancer Center, Earle A Chiles Research Institute, Portland, OR, USA

####### **Correspondence:** Bernard Fox (foxb@foxlab.org)

**Background**

Successful digital image analysis (DIA) of cancer tissue is accurate and reproducible. These points of emphasis have brought procedures like the tissue microarray (TMA) and hotspot regions of interest (ROI) under scrutiny. The nature in which a pathologist selects TMAs and ROIs is conducive to bias. Whole Slide Imaging (WSI) offers a solution in its unbiased region selection and consideration of a larger tissue sample. However, options for softwares that can handle such large throughput are scarce. Additionally, while multiplex immunohistochemistry (mIHC) is becoming popular [1], documentation of its digital analysis tools remains minimal [2]. The combination of these procedures potentiates a deeper understanding of the tumor microenvironment. This study presents the whole-slide mIHC analysis capabilities of QuPath, an open-source application developed at Queen’s University Belfast [3].

**Methods**

A multiplex fluorescent stain panel was performed on patient samples. The slides were imaged and cells were detected and segmented in QuPath. QuPath parallelizes its workload to manage whole-slide throughput efficiently. Custom scripts were written that exhibit machine-learning and thresholding techniques to aggregate cell phenotype totals. Additionally, cell detection numbers were generated for specific ROIs and compared to a commercial DIA software. All scripts and protocols in this study are made public for replication and improvement by the community.

**Results**

QuPath’s automated cell segmentation and classification were demonstrated as a proof-of-concept for whole-slide multiplex immunohistochemistry analysis. Across an entire slide, cells positive for multiple markers were effectively segmented and properly phenotyped.

**Conclusions**

Open-source applications have become a driving force for innovation and collaboration in the field of digital image analysis. In litigating the strengths and weaknesses of QuPath for whole-slide mIHC analysis, we aim to advance the field’s knowledge of available software tools and bring attention to necessary points of growth in this rapidly changing industry.

**References**

1. Feng Z, Jensen SM, Messenheimer DJ, Farhad M, Neuberger M, Bifulco CB, Fox BA. Multispectral imaging of T and B cells in murine spleen and tumor. J Immunol. 2016;196:3943-3950. 2. Blom S, Paavolainen L, Bychkov D, Turkki R, Mäki-Teeri P, Hemmes A, Välimäki K, Lundin J, Kallioniemi O, Pellinen T. Systems pathology by multiplexed immunohistochemistry and whole-slide digital image analysis. Sci Rep. 2017; 7:1-13. 3. Bankhead P, Loughrey MB, Fernández JA, Dombrowski Y, McArt DG, Dunne PD, McQuaid S, Gray RT, Murray LJ, Coleman HG, James JA, Salto-Tellez M, Hamilton PW. Qupath: open source software for digital pathology image analysis. Sci Rep. 2017; 7:1-7.

#### P441 Withdrawn

#### P442 Automated quantification of whole-slide multispectral immunofluorescence images to identify spatial expression patterns in the lung cancer microenvironment

##### Lorenz Rognoni, PhD^1^, Vinay Pawar, PhD^1^, Tze Heng Tan, MSc, PhD, DiplIng^1^, Felix Segerer, PhD^1^, Philip Wortmann, PhD^1^, Sara Batelli, PhD^1^, Pierre Bonneau^1^, Andrew Fisher, PhD^2^, Gayathri Mohankumar, MS^2^, David Chain, PhD^3^, Michael Surace, PhD^3^, Keith Steele, DVM, PhD^3^, Jaime Rodriguez-Canales, MD^3^

###### ^1^Definiens AG, Munich, Germany; ^2^Definiens Inc., Cambridge, MA, USA; ^3^Medimmune, Gaithersburg, MD, USA

####### **Correspondence:** Jaime Rodriguez-Canales (rodriguezcanalesj@MedImmune.com)

**Background**

Advancement in cancer immunotherapy is associated with unraveling the complexities of immune suppressive mechanisms across different cancers. Quantification on multispectral multiplex-immunofluorescence (mIF) images allows detection of several biomarkers in a single section. In addition, new evidence using mIF techniques suggests that spatial analysis reveals novel insights in the tumor microenvironment. However, multispectral imaging is tile based due to long scanning periods, which leads to insufficient data acquisition for significant spatial analysis. In this study, our goal is to develop an automated workflow to study the spatial patterns of infiltrating cells in the tumor microenvironment based on multispectral mIF whole slide scans. This was used to study the relationship between tumor proliferation and immune-response in non-small cell lung cancer (NSCLC) resections.

**Methods**

45 formalin fixed, paraffin embedded NSCLC resection samples were stained with a custom-developed 7-plex mIF panel (CD68, CD8, Ki67, PD1, PD-L1, pancytokeratins-CK & DAPI) using the Opal method (PerkinElmer). Tiled scans were acquired with a Vectra Polaris (PerkinElmer) multispectral imaging system. Definiens Insights services with custom algorithms was used to analyze the unmixed multispectral data as whole slide images.

**Results**

The 7-plex Opal staining was optimized for an automated staining platform to ensure high throughput and consistent sample processing. We developed a workflow which composes the tiled unmixed multispectral data to a whole-slide image and optimizes the layers for screen display and automated image analysis. Furthermore, images were shared on Definiens collaboration platform along with a chromogenic-IHC pseudocolor of the IF CK/DAPI signals and co- registered H&E section for pathologist annotations. These annotations were used in defining tumor center and invasive margin. The image analysis includes single-cell detection on the complete slide along with classification of subpopulations based on multi-marker positivity of individual cells. Part of the analysis is a high-quality tumor stroma separation based on the CK signal. The single-cell readouts were used to construct spatial biomarker- expression patterns (Figure 1), which shows distinct immunological areas in the tumor region and a possible correlation between tumor proliferation (Ki67) with the immune activity in the invasive margin.

**Conclusions**

We developed an automated workflow for quantitative mIF image analysis on whole-tissue slides. Additionally, our image analysis permitted identification of spatial patterns for immunoprofiling, where we could overcome the limitation of small regions of interests and provide significant amount of data on the whole tumor region.

**Ethics Approval**

Commercially available samples were obtained according to the declaration of Helsinki for this study.

**Fig. 1 (abstract P442).**
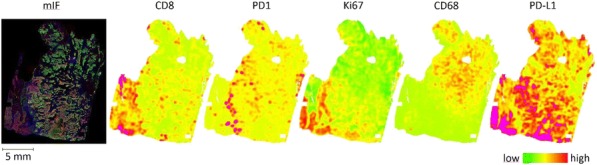
See text for description

#### P443 Haplotype human immune system (HIS) modeling and co-engraftment of PDX: ImmunoGraft® platform for evaluation of pharmacodynamics of Immuno Oncology therapeutics

##### Bhavana Verma, PhD^1^, Champions Oncology c/o Mancini, PhD^1^, Angela Davies, MD^1^, David Sidransky, MD^2^, Amy Wesa, PhD^1^, Neal Goodwin, PhD^1^

###### ^1^Champions Oncology, Rockville, MD, USA; ^2^Johns Hopkins University, Baltimore, MD, USA

####### **Correspondence:** Amy Wesa (awesa@championsoncology.com)

**Background**

Recent success of several immunotherapeutic regimens, such as checkpoint modulators has boosted development of next generation IO agents underscoring the need for robust preclinical platform to evaluate IO-therapies. The Champions ImmunoGraft® model utilizing humanized NOG mice is an innovative pre-clinical model for assessing the efficacy of IO agents against solid tumors. Improved immunodeficient mouse strains, such as triple transgenic NOG-EXL mice expressing huIL-3 and huGM-CSF, allows for superior HIS development. In this study, we evaluated human immune lineage development, tumor infiltrating leukocytes, and tumor response to checkpoint inhibitor utilizing this humanized mouse platform.

**Methods**

Human immune system component development in peripheral blood was assessed by flow cytometry across 9 donors 8 weeks post intravenous transplantation of cord-blood (CB) C34+ hematopoietic cells (HSC) in NOG and NOG-EXL mice. Next, NOG-EXL mice were humanized with CB-HSC from 2 donors, monitored for engraftment then implanted with a patient-derived xenograft (PDX) tissue from a non-small cell lung carcinoma (NSCLC) patient. Immune cell populations (T cells, macrophages, myeloid-derived suppressor cells (MDSC) and dendritic cells (DC)) were evaluated by flow cytometry at 4 and 6 weeks post-tumor implantation in various tissues. For nivolumab (α-PD-1; 10mg/kg) treatment, dosing was initiated at a tumor volume of 80-150 mm3. Responses were determined as changes in tumor volume.

**Results**

NOG-EXL (100%) consistently engrafted more readily than NOG (80%), with greater than 25% huCD45+ cells in the periphery. Some donor to donor variability was observed in HIS engraftment in both mouse strains; both strains permitted T cell, B cell and some myeloid cell development. T cell lineage development was equivalent in both strains at 12-weeks post–HSC transplantation. Improved myeloid lineage (CD33+) development was found in NOG- EXL animals. Macrophage, MDSC, as well as T cells were found in tumor infiltrates. Evaluation of PD-1 blockade in NSCLC PDX ImmunoGraft® in NOG-EXL mice indicated HIS donor variability impacted treatment efficacy in vivo.

**Conclusions**

ConclusionsImproved mouse strains that allow for robust reconstitution of immune compartments enhances value of ImmunoGraft® platform for screening new IO therapies. We demonstrated that NOG-EXL mice allow better engraftment and HIS development compared to NOG. Evaluation of nivolumab efficacy in NSCLC PDX model in this enhanced ImmunoGraft® indicates that PD-1 blockade is feasible, and offers an opportunity to evaluate therapeutics targeting myeloid populations. The ImmunoGraft® has the potential to advance translational IO drug discovery and development.

#### P444 Autologous human immune system (HIS) ImmunoGraft®: Mobilized peripheral blood (MPB) derived CD34 engraftment and lineage development

##### Bhavana Verma, PhD^1^, Georgia Chen, PhD^2^, Edmund Waller, MD, PhD, FACP^2^, Neal Goodwin, PhD^1^, Angela Davies, MD^1^ , Amy Wesa, PhD^1^, Nabil Saba, MD^2^

###### ^1^Champions Oncology, Rockville, MD, USA; ^2^Winship Cancer Institute of Emory University, Atlanta, GA, USA

####### **Correspondence:** Amy Wesa (awesa@championsoncology.com)

**Background**

Humanized mice generated by hematopoietic stem cells (HSC) transplant and co-engrafted with a patient-derived xenograft (PDX) represents a promising pre-clinical platform for studying immunological response to cancer and evaluation of immunotherapeutic interventions. These models are limited by the fact that the immune system developed in these mice is allogeneic to the tumor. To address this, we have innovated a platform to reconstitute autologous HIS in immunodeficient NOG-EXL mice with mobilized peripheral blood (MPB)-CD34 cells derived from a head and neck cancer patients along with PDX generated from the same patient tumor tissue.

**Methods**

Patients with head and neck squamous cell carcinoma (HNSCC) were consented for tumor and IRB approved apheresis for stem cell collection at Winship Cancer Institute of Emory University. The HSC collection protocol included mobilization with G-CSF and Plerixafor, prior to apheresis, isolation and cryopreservation of MPB-CD34 cells. PDX were established from biopsies or surgical specimens by passaging in immunodeficient mice. In parallel, Irradiated NOG-EXL mice were humanized by intravenous transplantation of HSC. Engraftment of human immune components (T cells, B cells and myeloid cells) in peripheral blood was assessed by flow cytometry up to 25 weeks, with terminal collections and assessment of immune components in spleen and bone marrow at 30 weeks.

**Results**

Twenty-eight PDX models were generated from 43 patients with HNSCC and evaluated by next-generation sequencing. In parallel, HIS engraftment assessed in circulation was observed at 8 weeks post-transplant in 100% of NOG-EXL mice; with 5-20% hCD45+ cells present. B cell development was predominant at early timepoints and declined over time. T cell development was observed starting at 15 weeks, with both CD4 and CD8 T cell subsets observed. Strong myeloid lineage (CD33+) development was observed starting at 8 weeks and persisted throughout the study. At 30 weeks we plan to evaluate immune compartments in blood, spleen and bone marrow of the humanized mice.

**Conclusions**

HSC mobilized from an adult patient with HNSCC was used to engraft and generate HIS-mice with B cells, T cells, and myeloid cells. In parallel, a matched PDX model was established from the same patient. The co-engraftment of HIS mice with an autologous PDX is in progress. This data demonstrate that mobilization and apheresis of HNSCC patients is technically and clinically feasible, and may permit the establishment of autologous HIS-PDX mice. The advanced autologous CD34-ImmunoGraft® has the potential to advance translational ImmunoOncology drug discovery and development.

**Ethics Approval**

Apheresis was performed based on an IRB approved protocol implemented at Winship Cancer Institute of Emory University

#### P445 Human CD33+ myeloid cells support metastatic colonization in a humanized mouse model of melanoma

##### Chun Yu, PhD^1^, Jan Martinek, PhD^1^, Te-Chia Wu^1^, Kyung Kim^1^, Elaheh Ahmadzadeh, PhD^1^, Rick Maser^2^, Florentina Marches^1^, Patrick Metang^2^, Pierre Authie^2^, Hannah Brookes^1^, Joshy George, PhD^1^, Jacques Banchereau^1^, A. Karolina Palucka, MD, PhD^2^

###### ^1^The Jackson Laboratory for Genomic Medicine, Farmington, CT, USA; ^2^The Jackson Laboratory for Mammalian Gen, Bar Harbor, ME, USA

####### **Correspondence:** A. Karolina Palucka (karolina.palucka@jax.org)

**Background**

Metastatic melanoma remains an incurable disease for some patients due to treatment resistance and metastatic dissemination. Here we show that metastatic melanoma tumor samples from patients are infiltrated with myeloid cells and display STAT3-driven transcriptional profiles.

**Methods**

To study the biology of myeloid cells and melanoma cells in vivo, we used NSG mice with transgenic expression of human hematopoietic cytokines SCF/GM-CSF/IL-3 (NSG-SGM3) engrafted with human CD34+ hematopoietic progenitor cells.

**Results**

Humanized NSG-SGM3 mice when implanted with Me275 human melanoma cell line subcutaneously, developed multi-organ distant melanoma tumors. This was linked with the presence of circulating tumor cells and elevated serum biomarker lactate dehydrogenase (LDH). Among six melanoma cell lines analyzed, potential to form distant tumors was correlated with G0/G1 cell cycle status and proliferative capacity. Treatment with VEGF inhibitor Avastin significantly decreased the number of melanoma tumors in the spleen but not in the liver. Adoptive transfer experiments confirmed the critical role of human CD33+ myeloid cells in metastatic colonization and these cells displayed STAT-3-driven transcriptional profiles.

**Conclusions**

Thus, our model enables mechanistic and pre-clinical studies for the development of novel treatment strategies targeting human-specific molecular pathways controlling melanoma dissemination.

#### P446 Using monocytes to instill brain tumors with TILs

##### Tomasz Zal, PhD, Meenakshi Shanmugasundaram, Anna Zal, Shouhao Zhou, Amy Heimberger, MD, Tomasz Zal, PhD, Felix Nwajei, PhD

###### University of Texas MD Anderson Cancer Center, Houston, TX, USA

####### **Correspondence:** Tomasz Zal (tzal@mdanderson.org)

**Background**

Tumors that localize in brain, including primary cancers and brain metastases, typically contain infrequent lymphocytic infiltrates. The paucity of tumor infiltrating lymphocytes (TILs) in brain tumors (BTs) presents a challenge for current immunotherapies reliant on TIL reactivation or lymphocyte (T cell) delivery. In emergent view, TILs are hailed by various types of tumor-associated myeloid cells, which can originate either from the organ’s tissue-resident macrophages, or from bone marrow sources. In healthy brain, the myeloid compartment is dominated by the resident microglia, whereas extracranial-derived myeloid cell subsets are rare outside of brain inflammation. However, the basis of TIL recruitment to brain tumor (BT) sites, or lack thereof, remains unclear.

**Methods**

We investigated TIL dynamics in BTs using longitudinal skull window multiphoton microscopy in multi- reporter mouse strains. Cyan fluorescent brain metastases were induced by infusing, into the carotid artery, syngeneic MCA205 (sarcoma), LLC (lung adenocarcinoma) or B16F10 (melanoma) cells, and GL261 glioblastoma was deposited directly into the brain. Endogenous T cells (hCD2-DsRed) were co-imaged with the microglia and monocytes and/or dendritic cells (CX3CR1-GFP and CD11c-YFP). We further distinguished the microglia, monocytic and dendritic cell subsets using three-color myeloid reporter mice (CX3CR1-GFP/CD11c-YFP/CCR2- RFP).

**Results**

Present in various densities across BT models, TILs were accompanied by myeloid cells expressing the fractalkine receptor CX3CR1, which is expressed predominantly on the microglia and monocytes or monocyte-derived cells. Fractalkine (CX3CL1) was upregulated on BT-juxtaposed neurons and CX3CR1-expressing monocytes adhered to and scanned the local vasculature. TILs were decreased, and their motility aimless, in mice lacking CX3CR1, resulting in increased BT growth. However, the TIL’s spatial and temporal densities correlated with dendritic-form cells expressing high levels of CD11c (DCs), rather than CD11c-negative monocytes or microglia. TIL migration was confined around CD11c-high DCs and the confinement radius was tighter, in coincidence with cancer cell killing, in tumors undergoing T-cell mediated immune rejection, compared with BTs that were progressing. Depletion of CD11c cells from mice with established BTs led to a rapid abandonment of BTs by the TILs, whereas infusion of (CD11c-negative) monocytes into the blood gave rise to intratumoral CD11c-high dendritic cells, which could attract TILs.

**Conclusions**

Our results identify the patrolling monocytes and their development into intratumoral DCs as critical cellular mediators of the adaptive immune surveillance of tumors in brain. Moreover, our results establish a proof of principle for the use of monocyte adoptive cell transfer as a potential therapeutic strategy for instilling BTs with TILs.

**Acknowledgements**

Funding was provided by the NCI (5R01CA137059, 5P50CA127001/DP150072), the University of Texas MD Anderson Cancer Center (IRG 30026195, MRP1152014) and the Schissler Family Fellowship (FN).

**Ethics Approval**

The study was approved by the MD Anderson Institutional Animal Care and Use Committee, protocol number 00000878-RN02.

### Immune Effects of Chemotherapy

#### P447 A case of checkpoint inhibitor-induced celiac disease

##### Dana Alsaadi, Neil Shah, MD, Aline Charabaty, MD, Michael Atkins, MD

###### Georgetown University, Washington, DC, USA

####### **Correspondence:** Neil Shah; Michael Atkins (mba41@gunet.georgetown.edu)

**Background**

Immune checkpoint inhibitors (ICIs) have now become standard of care treatment for many malignancies. ICIs are associated with unique immune mediated adverse events (irAEs) due to dysregulation of immune activation. As treatment with ICIs is becoming more common, rare irAEs are also being recognized. Here we report a case of ICI- induced celiac disease.

**Methods**

N/A

**Results**

A 74-year-old Caucasian female with metastatic renal carcinoma received second line nivolumab (anti-PD1 antibody) after initial disease progression on sunitinib. Ipilimumab was added after she failed to respond to six cycles of nivolumab monotherapy. One week after her first cycle of combo treatment, she presented with nausea, vomiting, grade 1 diarrhea, and weight loss. She underwent endoscopy, which showed bile stasis in the stomach, normal appearing stomach mucosa, and non-bleeding erythematous mucosa in the duodenal bulb. Stomach biopsy showed moderate active chronic gastritis. Duodenal biopsy showed moderate chronic active duodenitis with focal neutrophilic cryptitis, mucosal erosions, villous atrophy, mildly increased intraepithelial lymphocytes, and moderate chronic inflammation in the lamina propria pathognomonic of celiac disease. Symptoms improved with gluten-free diet, twice-daily omeprazole and anti-emetics and she was able to continue on treatment.

**Conclusions**

There has been only one published case reporting ICI-induced celiac disease.[1] Our case report highlights a rare irAE (celiac disease) associated with ICI treatment. It is unclear whether the patient had previously undiagnosed celiac disease or whether ICIs triggered her enteritis. Our patient was able to continue treatment with ICIs with dietary modifications, suggesting correct diagnosis is critical for optimal patient outcome.

**References**

1. Gentile NM, D’Souza A, Fujii LL, Wu TT, Murray JA. Association between ipilimumab and celiac disease. Mayo Clin Proc 2013; 88: 414-417.

**Consent**

Consent was received.

#### P448 Blockade of EphB4-ephrin-B2 interaction remodels the tumor immune microenvironment in head and neck cancers

##### Shilpa Bhatia^1^, Sana Karam, MD, PhD^2^

###### ^1^University of Colorado, Anschutz Medical Campus, Aurora, CO, USA; ^2^University of Colorado Denver, Aurora, CO, USA

####### **Correspondence:** Shilpa Bhatia (Sana.karam@ucdenver.edu)

**Background**

Identifying targets in the tumor microenvironment (TME) that act as barriers to an effective anti-tumor immune response has become an area of intense investigation. In the current study, we established EphB4-ephrin-B2 signaling as a key pathway that regulates both innate and adaptive arms of the immune system. Eph receptor tyrosine kinases and their membrane-bound ephrin ligands have been implicated in human malignancies and in immune cell development, migration, and activation in inflammatory models. However, direct evidence that supports the role of Eph-ephrin interaction in cancer-related immune response is lacking. We hypothesized that EphB4-ephrin-B2 interaction regulates TME by sustaining immunosuppressive cells-Tregs and TAMs thus negatively impacting the functional ability of CD8 T cells.

**Methods**

We used orthotopic models of head and neck squamous cell carcinoma to determine the role of EphB4-ephrin-B2 interaction in tumor immune microenvironment. Mice were treated with control agent or an EphB4-ephrin-B2 blocker in the absence or presence of radiation (RT). Tumor immune cell infiltrates were analyzed using mass cytometry and flow cytometry applications. ELISA or multiplex cytokine array were utilized to determine circulating cytokine/chemokine levels in plasma.

**Results**

We observed that inhibition of EphB4-ephrin-B2 signaling in vivo significantly reduced tumor growth and decreased the infiltration of Tregs, TAMs, and increased infiltration and activation of Teffector cells, without affecting CD4 T cell numbers. This was correlated with decreased Treg proliferation and activation when EphB4- ephrin-B2 signaling is inhibited. Since RT remains the mainstay in treatment of head and neck squamous cell cancer (HNSCC) patients, we combined EphB4-ephrin-B2 inhibitor with RT in our tumor model and observed further increase in CD8 and CD4 T cell infiltrates and activation status, and a significant decline in circulating IL-10 and TGF-β1 levels compared to the control group. A significant reduction of TAMs, favoring a polarization towards an anti-tumoral M1 phenotype, was also observed in EphB4-ephrin-B2 inhibitor+RT group. We also compared the efficacy of combining EphB4-ephrin-B2 inhibitor with RT to anti-PDL1+RT in an in vivo model known to develop resistance to anti-PDL1+RT therapy. Our data demonstrated that combining EphB4-ephrin-B2 inhibitor with RT was equally effective to that of anti-PDL1+RT in terms of anti-tumor growth response.

**Conclusions**

Our study provides the first insight into a novel role for EphB4-ephrin-B2 interaction in modulating tumor immune microenvironment in HNSCC. Our findings present a potential alternative in the form of EphB4-ephrin-B2 targeted therapeutics that can be tested in clinical trials in combination with RT for HNSCC patients.

#### P449 Improving PDAC outcomes through targeting immune populations and fibrosis by EphB4-ephrinB2 or Treg inhibition combined with radiation

##### Sana Karam, MD, PhD^2^, Shilpa Bhatia^1^

###### ^1^University of Colorado, Anschutz Medical Campus, Aurora, CO, USA; ^2^University of Colorado Denver, Aurora, CO, USA

####### **Correspondence:** Sana Karam (Sana.karam@ucdenver.edu)

**Background**

A driving factor in pancreatic ductal adenocarcinoma (PDAC) treatment resistance is the tumor microenvironment, which is highly immunosuppressive. One potent immunological adjuvant is radiation therapy (RT). Radiation, however, has also been shown to induce immunosuppressive infiltration, which can contribute to tumor progression. Another negative effect is the potential contribution to formation of fibrotic tumor stroma. To capitalize upon the immunogenic effects of radiation and obtain a durable tumor response, radiation must be rationally combined with targeted therapies to mitigate the influx of immunosuppressive cells and fibrosis. One such target is ephrinB2, which is overexpressed in PDAC and correlates negatively with prognosis. Based upon previous studies of ephrinB2 ligand-EphB4 receptor signaling, we hypothesized that inhibition of ephrinB2-EphB4 combined with radiation would regulate the microenvironment response post radiation, leading to increased tumor control in PDAC.

**Methods**

Immunocompetent C57BL/6 and immune compromised athymic nude mice were injected subcutaneously with either a patient derived xenograft (PDX) tumor, PANC 272, or a mouse pancreata derived cell line (FC1242) and randomized into PBS, B11 (an inhibitor of ephrinB2-EphB4 interaction), RT and B11+RT groups. Depletion studies were conducted using anti-IgG or anti-CD35 antibodies. To determine tumor immune cell infiltration, tumors were subjected to flow cytometric analysis. Plasma samples were subjected to ELISA to determine circulating TGFβ1 levels in control and treatment groups. Fibrosis was quantified following Masson’s Trichrome staining and PicroSirius Red staining.

**Results**

Our data show that combining ephrin-B2-EphB4 inhibitor with RT significantly reduces regulatory T-cell and neutrophil infiltration, TGFβ1 secretion, and stromal fibrosis, enhancing effector T-cell activation and decreasing tumor growth. Further, our data demonstrate that depletion of regulatory T-cells in combination with radiation reduces tumor growth and fibrosis as demonstrated by Masson’s Trichrome staining and PicroSirius Red staining.

**Conclusions**

These are the first findings to suggest that in PDAC, ephrinB2-EphB4 interaction has a profibrotic, pro-tumorigenic role, presenting a novel and promising therapeutic target.

#### P450 The stapled peptide ALRN-6924, a dual inhibitor of MDMX and MDM2, displays immunomodulatory activity and enhances immune checkpoint blockade in syngeneic mouse models

##### Luis Carvajal, PhD, Narayana Narasimhan, PhD, Jian-Guo Ren, PhD, Solimar Santiago, MS, Manoj Samant, PhD, David Sutton, PhD, Vincent Guerlavais, PhD, D. Allen Annis, PhD, Manuel Aivado, MD, PhD

###### Aileron Therapeutics, Inc., Cambridge, MA, USA

####### **Correspondence:** Manuel Aivado (maivado@aileronrx.com)

**Background**

The tumor suppressor p53 is one of the most pursued targets in oncology, playing a central role inducing cell cycle arrest, apoptosis and senescence in response to cellular stress and oncogenic signals. In addition to its intrinsic anti- tumor activity in cells, p53 activation can induce anti-tumor immunity and plays an important role in the regulation of innate and adaptive immunity. Therefore, p53-reactivating agents in combination with immune checkpoint blockade (ICB) may represent a powerful approach to optimize the body’s immunological response against cancer. ALRN-6924 is an α-helical stapled p53 peptide currently in clinical testing that has demonstrated anticancer activity as monotherapy [1]. In this study, we investigated whether p53 reactivation with ALRN-6924 can be leveraged as new combination partner for ICB.

**Methods**

Peripheral blood mononuclear cells (PBMCs) were stimulated with ALRN-6924 ex vivo for 24 hr. Gene expression and cytokine levels were measured using a validated TaqMan assay (ThermoFisher) and the Human XL Cytokine Array Kit (R&D Systems). Immune profiles from pre- and post-treatment tumor biopsy samples were evaluated by NanoString PanCancer IO360 and Immune Profiling RNA gene expression panels. Immunophenotyping of PBMCs was done by flow cytometry. Efficacy and immune cell profile (determined by flow cytometry and IHC) were evaluated in CloudmanS91 and MC38 syngeneic murine tumor models following treatment with ALRN-6924 alone and in combination with anti-PD-1 or anti-PD-L1, including re-challenge studies to test for immunological memory.

**Results**

Ex vivo stimulation of PBMCs with ALRN-6924 promotes transcriptional activation of genes involved in innate and adaptive immunity, and the production of immune-stimulating cytokines including INF-γ, IL-6 and IL-12. mRNA analysis of pre- and post- treatment tumor biopsies from patients treated with ALRN-6924 revealed a differential gene expression pattern consistent with conversion to an inflamed tumor phenotype. In syngeneic mouse models, ALRN-6924 was sufficient to promote infiltration of CD8+ T cells, polarization of M1 macrophages in mouse tumors and immunological memory. Moreover, ALRN-6924 synergizes with anti-PD-1 and anti-PD-L1 to induce anti-tumor immunity resulting in an increased number of mice achieving complete regressions (CR), in both p53 wild-type and mutant tumors, compared to single agents.

**Conclusions**

Reactivation of p53 with ALRN-6924 enhanced the effects of ICB therapy in mice. Furthermore, the present study suggests that ALRN-6924 modulates anti-tumor immunity in p53 wild type, and p53 mutant tumors, possibly via tumor cell extrinsic effects in the tumor microenvironment.

**References**

1. Funda Meric-Bernstam, M. S. Saleh, J. R. Infante, S. Goel, G. S. Falchook, G. Shapiro,K. Y. Chung, R. M. Conry, D. S. Hong, J. S. Wang, U. Steidl, L. D. Walensky, V. Guerlavais, M. Payton, D. A. Annis, M. Aivado, M. R. Patel, Phase I trial of a novel stapled peptide ALRN-6924 disrupting MDMX- and MDM2- mediated inhibition of WT p53 in patients with solid tumors and lymphomas. J. Clin. Oncol. 35, 2505–2505 (2017).

**Ethics Approval**

All studies involving human material or animal (in vivo) studies were approved under Aileron's IND122392 protocol and by the Institutional Animal Care and Use Committee at Charles River Laboratories, Morrisville, N.C. ASP #: 990202, respectively.

#### P451 Gal9/Tim-3 expression level is higher in patient with failed chemotherapy in AML

##### Justin Kline, MD, Paola Dama, PhD, Hongtao Liu, MD, PhD

###### University of Chicago, Chicago, IL, USA

####### **Correspondence:** Hongtao Liu (hliu2@medicine.bsd.uchicago.edu)

**Background**

Activation of immune checkpoint pathways in Acute Myeloid Leukemia (AML) may interfere with effective T-cell anti-tumor immunity, and is associated with immune evasion in pre-clinical leukemia models as it has been demonstrated. [1,2] It was previously reported that overexpression of CTLA4 and PD-1 is associated with more aggressive leukemia and progression from MDS to AML or AML relapse. While PD-1/PD-L1 blockade therapy can be effective as cancer immunotherapy, interruption of PD-1/PD-L1 interactions alone does not completely restore T cell function in some patients indicating the involvement of additional negative regulatory pathways, such as Tim- 3/Gal-9, in T cell exhaustion. Immune checkpoint pathways active in Acute Myeloid Leukemia (AML) patients, especially during the course of remission induction chemotherapy, have not been well-studied. We characterized these pathways in newly diagnosed AML patients enrolled in a phase I dose escalation trial that combined Selinexor a Selective Inhibitor of Nuclear Export (SINE) with high-dose cytarabine (HiDAC) and mitoxantrone (Mito) (NCT02573363) as induction therapy.

**Methods**

Multi-parameter flow-cytometry was performed on bone marrow specimens at diagnosis and following remission induction therapy in 26 patients with AML enrolled to the study to monitor the changes in expression of immune checkpoint receptors. Expression of CD47, PD-L1, PD-L2 and Gal-9 was assessed on CD34+ AML blasts and CD34- cell populations. In parallel, expression of inhibitory (PD1, CTLA4, LAG3, TIM3) and stimulatory co- receptors (CD28, ICOS, CD137, OX40, CD40L, HLA-DR) on CD4+ and CD8+ T cell subsets were evaluated. The positivity and frequency of parent in percentage of each markers was gauged by comparing with their FMO controls. Samples were analyzed using LSR Fortessa or LSRII Cytometers. The Mann Whitney Test, Spearman’s rank correlation and Runs Test analysis were applied. For all analyses, P-values <0.05 were considered statistically significant.

**Results**

The percentage of CD34- Gal9+ cells was significantly higher and was positively correlated with higher numbers of TIM-3-expressing T cells at the time of diagnosis in patients who experienced treatment failure (TF) after chemotherapy, compared to those in complete remission (CR). When comparing TIM-3 expression on CD4+ and CD8+ T cells in pre-treatment (diagnosis) to post induction therapy samples, the magnitude of increase measured by median fluorescence intensity (MFI) inversely correlated to response to therapy with increase TIM-3 MFI of > 50% in patients with TF.

**Conclusions**

This study provides preliminary evidence to support a rationale for incorporating antibodies against the Gal9/TIM3 pathway during and/or following remission induction therapy for AML.

**References**

1. Zhang L, Gajewski TF, Kline J. PD-1 / PD-L1 interactions inhibit antitumor immune responses in a murine acute myeloid leukemia model. Blood. 2009; 114(8), 1545–1552.

2. Zhou Q, Munger ME, Blazar BR. Coexpression of Tim-3 and PD-1 identifies a CD8+T-cell exhaustion phenotype in mice with disseminated acute myelogenous leukemia. Blood, 2011;117(17), 4501–4510.

**Ethics Approval**

The study was approved by the Institutional Review Board at The University of Chicago, approval number 150412.

#### P452 Cisplatin treatment induces anti-tumor immune response in NSCLC by activation of the innate immune response pathway

##### Triparna Sen, PhD^2^, Lixia Diao^2^, Kavya Ramkumar^2^, Carl Gay^2^, Pan Tong^2^, You-Hong Fan^2^, Robert Cardnell^2^, Don Gibbons, MD^2^, John Heymach^2^, Jing Wang^2^, Lauren Byers, MD^2^, Carminia Della Corte, MD^2^

###### ^1^The University of Texas MD Anderson Cancer Center, Houston, TX, Houston, TX, USA; ^2^MD Anderson Cancer Center, Houston, TX, USA

####### **Correspondence:** Carminia Della Corte (cmdella@mdanderson.org)

**Background**

Platinum-based doublet chemotherapy plus anti-PD1 immunotherapy is a new standard of care for the treatment of advanced NSCLC patients. It is known that DNA damage can activate antitumor immune responses in cancer through release of cytosolic DNA leading to Stimulator of Interferon Genes (STING) pathway activation, production of neo-antigens, and release of pro-inflammatory cytokines. Our group has previously demonstrated that mesenchymal tumors with high EMT scores have the highest expression of targetable immune markers (1). However, the underlying mechanism of how platinum-based chemotherapy modulate the immune microenvironment is far from being fully understood in NSCLC. The aim of this study is to elucidate the effect of platinum-based chemotherapy on anti-tumor immune response and identify novel biomarkers to aid patient selection for chemotherapy and immunotherapy combination clinical trials.

**Methods**

We analyzed transcriptomic and proteomic expression of immune markers in NSCLC samples from two clinical datasets (MDACC-PROSPECT, n= 209) and The Cancer Genome Atlas (TCGA, n=1016). We also treated NSCLC cell lines with cisplatin to investigate its effect on DNA damage and changes in immune markers expression by western blot and Reverse-Phase Protein Array (RPPA analysis).

**Results**

Treatment with cisplatin increased DNA damage (increased γH2AX), and significantly upregulated PD-L1 and STING pathway protein expression in a panel of NSCLC cell lines.In the TCGA cohort, immune checkpoints and inflammatory cytokines mRNA expression is highly coordinated and positively correlated with EMT genes. In the TCGA lung adenocarcinoma (LUAD, n=515) cohort, high expression of effector chemokines (CXCL10, CCL5) and mediators of STING pathway (TBK1, TMEM173) were associated with high levels of CD274 (PD-L1) and other targetable immune markers (LAG3, IDO1, PDCD1LG2, CTLA4). These findings were further validated both in lung squamous (LUSC) TCGA and the PROSPECT cohorts (LUAD and LUSC). Interestingly, in the LUAD TCGA cohort, smoking status (another source of DNA damage) was significantly correlated with higher expression of STING and other immuno-modulatory genes, and EMT signature.

**Conclusions**

Our results demonstrate that in treatment naïve-NSCLC tumors, expression of PDL1 and other targetable immune markers correlate with expression of STING, EMT, smoking status and DDR pathway genes, and that treatment with cisplatin further enhances the immunogenicity of tumors through activation of the STING pathway in NSCLC cells. Our findings identify a novel mechanism by which cisplatin activates an innate immune response pathway in NSCLC. Furthermore the results identify potential biomarkers (EMT, smoking status, DDR protein expression) for patient selection in clinical trials.

**References**

1. Mak MP, Tong P, Diao L, et al. A Patient-Derived, Pan-Cancer EMT Signature Identifies Global Molecular Alterations and Immune Target Enrichment Following Epithelial-to-Mesenchymal Transition. Clin Cancer Res. 2016;22:609-20.

#### P453 Enhancing the anti-tumor immunity elicited by alpha radiation-based brachytherapy using immunoadjuvants and blockade of suppressor cells

##### Vered Domankevich-Bachar, DR^1^, Adi Cohen^2^, Margalit Efrati^2^, Michael Schmidt^2^, Hans Georg Rammensee^3^, Itzhak Kelson^2^, Yona Keisari^2^

###### ^1^Tel Aviv University, Tel Aviv, Israel; ^2^Tel Aviv Uiniversity, Tel-Aviv, Israel; ^3^University of Tübingen, Tübingen, Germany

####### **Correspondence:** Yona Keisari (ykeisari@tauex.tau.ac.il)

**Background**

Diffusing alpha emitters Radiation Therapy (DaRT) is a novel brachytherapy treatment for solid tumors. DaRT seeds disperse short-lived alpha-emitting atoms in a therapeutically-significant range, which diffuse inside the tumor and destroy a sizeable part of it. Thus, for the first time, an efficient and safe method for treating the entire solid tumor by highly destructive alpha radiation is used. In situ tumor ablation is known to release tumor antigens and damage associated molecular pattern molecules (DAMPs) that lead to the induction of systemic anti-tumor immunity. Indeed, we previously reported that in the breast cancer carcinoma mice model DA3, DaRT-treated mice showed increased survival rates, and reduced rates of lung metastases and of primary- or challenged- tumor development. Here we aimed to boost the anti-tumor immune response induced by DaRT, locally and systematically, and to investigate the specificity of the response.

**Methods**

Mice breast (4T1) and colon (CT26) tumors, implanted subcutaneously, were treated with DaRT seeds with/without immunomodulatory agents. Immunomodulatory agents studied are the immunoadjuvants polyIC, CpG, and XS15, the MDSC inhibitor sildenafil, and the Treg inhibitor cyclophosphamide. Non-radioactive seeds (inert) served as control. Local- and systemic- responses were determined by tumor progression, host survival, response to challenge and lung metastasis. The specificity of the immune response was studied by Winn Assay and tumor challenge to cured tumor-bearing mice.

**Results**

It was found that in the CT26 colon cancer mice model: (1) combining DaRT with polyIC, CpG or XS15 significantly reduced tumor progression and prolonged survival. (2) Complete response was achieved when using DaRT combined with CpG and immune suppressor cells inhibitors. (3) Cured mice became resistant to CT26 cells but not to DA3 (breast cancer) cells. (4). Splenocytes from CT26 bearing mice cured by DaRT specifically reduced CT26 but not DA3 tumor take in naïve mice. In the triple negative breast cancer model, 4T1, treating the primary tumor with polyIC, prior to DaRT treatment, reduced tumor progression and eliminated lung metastases.

**Conclusions**

DaRT is currently tested under clinical trials in squamous cell carcinoma patients showing effective tumor control without adverse effects. The current results provide strong evidence for the induction of a specific- and systemic- immune response against tumor antigens following DaRT treatment. We propose DaRT as a safe and efficient novel strategy, not only for tumor ablation, but also for in situ vaccination of cancer patients.

#### P454 Elucidating the functional role of type-1 interferon signaling following a medium-dose intermittent cyclophosphamide schedule in preclinical breast cancer models

##### Kshama Doshi, PhD, Cameron Vergato, Kshama Doshi, PhD, Darren Roblyer, PhD, David Waxman, PhD

###### Boston University, Allston, MA, USA

####### **Correspondence:** David Waxman (djw@bu.edu)

**Background**

Many cytotoxic chemotherapy drugs, including the breast cancer standard of care drug cyclophosphamide (CPA), can induce immunogenic cell death when administered at medium-dose and intermittent (MEDIC) schedule [1]. Adaptive and innate immune responses generated in this manner can greatly potentiate chemotherapy drug efficacy and generate tumor-specific long-term immune memory. Cancer cells have also been shown to up-regulate type-1 interferon (IFNα/β) signaling in response to many chemotherapy drugs. Here we set out to elucidate the effects and mechanisms of immune activation in a breast cancer preclinical model using a MEDIC schedule of CPA.

**Methods**

We used an in-vitro IFN-based biomarker strategy to identify breast cancer models that can induce immunogenic responses following treatment with 4-hydroperoxy cyclophosphamide (4HC), a chemically activated form of CPA. Sub-lethal concentrations of 4HC were established by MTS assay and used to study induction of interferon- stimulated genes by qPCR in five breast cancer cell lines: 4T1, E0771, Emt6, Py230 and MCF7. Anti-IFN receptor- 1 antibody was used to verify the role of IFNα/β in 4HC-induced interferon-stimulated gene induction. CPA- induced immune activation was also evaluated in a syngeneic mouse tumor model. Mice with orthotopic tumors implanted in the 4th mammary fat pad were treated with a MEDIC schedule of CPA and tumor progression was monitored. Effects of drug treatment on IFN signaling and immune cell infiltration into the tumor compartment was evaluated by marker gene expression.

**Results**

Screening results revealed sub-lethal concentrations of 4HC significantly induced multiple interferon-stimulated genes, including Cxcl10 (~8 fold), Igtp (~15 fold) and Mx1 (~20 fold) in 4T1 breast cancer cells. In contrast partial to minimum induction was seen in E0771, Emt6, Py230 and Mcf7 cells. Further, anti-IFN receptor-1 antibody blocked > 90% of 4HC-induced interferon-stimulated gene induction in 4T1 cells. Conditioned media from 4HC- treated 4T1 cells also stimulated IFNα/β signaling in drug-naive recipient 4T1 cells. Finally, in syngeneic mouse tumor models, MEDIC CPA scheduling significantly reduced tumor progression resulting in tumor stasis. Diminished tumor growth kinetics was accompanied by significant increases in expression of interferon-stimulated genes and cytolytic enzymes in the tumor compartment.

**Conclusions**

Breast cancer cell autonomous activation of type-1 IFN signaling and downstream gene induction is activated in a syngeneic 4T1 breast cancer model treated with a MEDIC CPA regimen. Mechanistic features of this pathway, involving autocrine and paracrine type-1 IFN signaling loop, were characterized in-vitro. Studies assessing key immune players and their role in anti-tumor responses are in progress, and will be presented.

**Acknowledgements**

Grant support: DOD grant # U.S. Department of Defense (DOD) (W81XWH-15-1-0070)

**References**

1. Wu J, Waxman DJ. Immunogenic chemotherapy: Dose and schedule dependence and combination with immunotherapy. Cancer Lett. 2018;419:210-221.

#### P455 Inducible T cell Co-stimulator (ICOS) is upregulated on lymphocytes following radiation of tumors and ICOS agonism in combination with radiation results in enhanced tumor control

##### Michael Gough, PhD^1^, Shelly Bambina^1^, Monica Gostissa, PhD^2^, Christopher Harvey, PhD^2^, David Friedman, PhD^1^ Marka Crittenden, MD, PhD^1^

###### ^1^Earle A. Chiles Research Institute, Portland, OR, USA; ^2^Jounce Therapeutics Inc., Cambridge, MA, USA

####### **Correspondence:** Marka Crittenden (marka.crittenden@providence.org)

**Background**

Radiation and co-stimulatory ligands or checkpoint inhibitors have demonstrated improved anti-tumor immunity and overall survival in preclinical animal studies. However, the results of human trials suggest we have not yet found the optimal combination. Here we demonstrate upregulation of ICOS expression on T cells following focal tumor radiation and test the hypothesis that ICOS agonism in combination with radiation will enhance the immunologic effect of radiation resulting in increased survival.

**Methods**

BALB/c mice bearing CT26 tumors or C57BL/6 mice bearing Panc02 tumors were treated at d14 with 20Gy CT guided radiation therapy and anti-ICOS antibody or isotype control antibody was administrated i.p. Mice were followed for overall survival to 100 days post implantation. Animals were euthanized when tumors reached 1.2cm in greatest diameter. Flow cytometry was performed using a T cell panel on fresh whole blood, PBMC, or tumor infiltrating immune cells.

**Results**

24 hours following 20Gy focal radiation to a CT26 tumor there was a significant increase in the percent of circulating CD4 Treg that express ICOS in the blood (27.42% vs 18.02%, p<0.0001, n=5/group). Similarly, 7 days following radiation there was an increase in non-Treg CD4 cells expressing ICOS in the blood (7.73% vs 3.68%, p<0.0001, n=5/group) and the tumor (62.16% vs 34.04%, p=0.004, n=5/group). ICOS expression was also increased on CD8 T cells in irradiated tumors (25.34% vs 14.02%, p=0.007). In mice bearing CT26 tumors, ICOS agonist antibody was administered prior to, concurrent with, or 7 days post radiation. Concurrent administration was associated with the most significant increase in survival (50%) when compared to isotype control (0%), ICOS agonist antibody alone (10%), or radiation plus isotype (0%). In the less immunogenic Panc02 tumor model, no survival benefit was seen with radiation and ICOS therapy. However in the same model, dual PD-1 antagonism and ICOS agonism plus radiation led to a significant increase in survival when compared to all other combinations, with an increase in median survival from 46 days to 68 days, p=0.01 compared to radiation alone and was associated with a 25% long term survival.

**Conclusions**

ICOS is upregulated on T cells following radiation and targeting ICOS in combination with radiation is associated with improved survival. Timing appears important as the benefit is optimal when ICOS agonism is delivered concurrent with radiation rather than preceding or 7 days post-radiation. In poorly immunogenic tumors, addition of PD-1 antagonism to the combination can lead to improved survival.

**Ethics Approval**

Animal protocols were approved by the Earle A. Chiles Research Institute IACUC (Animal Welfare Assurance No. A3913-01). All experiments were performed in accordance with relevant guidelines and regulations.

#### P456 Pressure-enabled delivery of CAR-T cells into the porcine pancreas results in highly-targeted pancreatic delivery with minimal systemic exposure and no pancreatitis or severe systemic cytokine release

##### John Hardaway, MD, PhD^1^, James Chomas, PhD^2^, David Jaroch, PhD^2^, Prajna Guha, PhD^1^, N. Joseph Espat, MD, FACS^3^, Steven Katz, MD^3^, Aravind Arepally, MD^4^

###### ^1^Roger Williams Medical Center, Providence, RI, USA; ^2^Surefire Medical, Inc., WESTMINSTER, CO, USA; ^3^Roger Williams Medical Center, Boston University, Providence, RI, USA; ^4^Surefire Medical, Inc., Piedmont Medical Center, Westminster, CO, USA

####### **Correspondence:** Aravind Arepally (aravind.arepally@surefiremedical.com)

**Background**

The purpose of this preclinical study is to determine whether highly preferential delivery of T cells into the pancreas can be achieved while minimizing systemic exposure and avoiding systemic and pancreatic inflammation using the Surefire® Retrograde Venous-Pressure Enabled Drug Delivery (RV-PEDD) method and device, as compared to systemic venous infusion (SVI).

**Methods**

Healthy human donor CAR-T cells (Sorrento Therapeutics) or unmodified activated T cells were transferred into 10 normal adult swine by either (a) SVI (n=5) or (b) RV-PEDD via trans-hepatic access into pancreatic veins (n=5). Samples of peripheral blood (PB) were obtained at 15, 30, and 120 minutes after infusion. Serum was analyzed for porcine tumor necrosis factor-alpha (TNF-α) and interleukin-6 (IL-6) by enzyme-linked immunosorbent assay (ELISA) as indices of systemic inflammation, whereas circulating CAR-T were quantified using flow cytometry. Liver and pancreatic tissues were harvested for histology, immunofluorescence (IF) of human CD3, and determination of human CD3 mRNA expression via qPCR.

**Results**

After SVI, the donor CAR-T cell fraction among circulating mononuclear cells was 13.7% at 15 minutes, 31.7% at 30 minutes, and 20.5% at 120 minutes, versus RV-PEDD that yielded 1.8% detection at 15 minutes, and undetectable cells at 30 and 120 minutes. With SVI, IF found substantial accumulation of donor CAR-T cells in PB and minimal pancreatic staining, as opposed to RV-PEDD infusion where substantial pancreatic accumulation and minimal PB staining had occurred (Figure 1). qPCR analysis of pancreatic tissues from RV-PEDD specimens revealed a 147-fold increase in CAR-T penetration, as compared to SVI. Alternatively, analysis of PB following SVI revealed a 61-fold increase in systemic exposure with negligible detection in the pancreas. Histologically defined pancreatic inflammation was not evident in any animal after RV-PEDD (Figure 2). Systemic inflammation, based on TNF-α and IL-6 levels, was not evident within 2 hours after RV-PEDD infusion, whereas pronounced increases of TNF-α and IL-6 levels were observed with systemic infusion.

**Conclusions**

RV-PEDD was associated with efficient and specific delivery of donor T cells into porcine pancreas compared to systemic infusion. RV-PEDD did not result in significant systemic exposure nor induce pancreatic or systemic inflammation. These data support clinical testing of Surefire® RV-PEDD technology to improve the therapeutic index with selective delivery of cellular therapeutics into the pancreas.

**Ethics Approval**

The study was approved by the T3 Labs IACUC on 6/15/16, with amendments on 11/17/16, 2/24/17.

**Fig. 1 (abstract P456).**
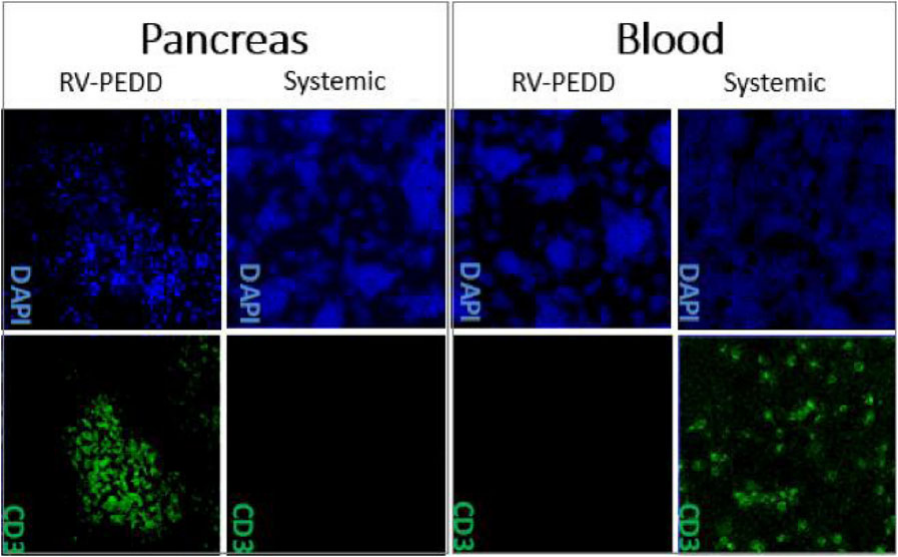
See text for description

**Fig. 2 (abstract P456).**
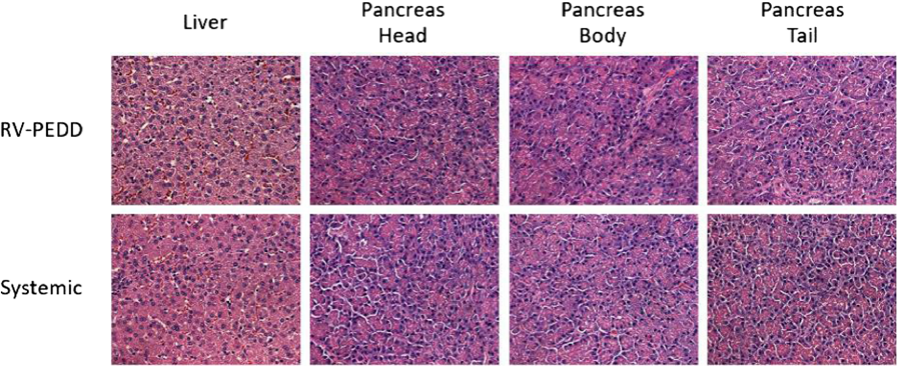
See text for description

#### P457 TP53 gene therapy emanating from the investigational agent SGT-53 is capable of augmenting cancer immunotherapy in multiple murine syngeneic tumor models

##### Joe Harford, PhD^1^, Sang-Soo Kim, PhD^1^, Kathleen Pirollo, PhD^2^, Antonina Rait, PhD^2^, Manish Moghe^2^, Esther Chang, PhD^2^

###### ^1^SynerGene Therapeutics, Inc., Potomac, MD, USA; ^2^Georgetown University Medical Center, Washington, DC, USA

####### **Correspondence:** Joe Harford (harfordj@synergeneus.com)

**Background**

Manipulation of immune checkpoints has emerged as an important form of cancer immunotherapy [1,2]. However, a large number of patients do not respond or develop resistance to checkpoint blockade, and treatment-related toxicities can be limiting. The tumor suppressor p53 exerts anti-tumor activity by inducing apoptosis, but also participates in the regulation of cellular immune responses [3.4]. We have investigated the potential of TP53 gene therapy to augment cancer immunotherapy by combining an anti-PD1 antibody with SGT-53, an investigational nanomedicine that targets tumors and carries a plasmid encoding human wild-type p53 (Figure 1). SGT-53 is now in Phase II clinical trials [5,6].

**Methods**

We utilized mouse syngeneic tumor models that are relatively resistant to immunotherapy including an aggressive metastatic breast cancer (4T1), a non-small cell lung carcinoma (LL2), and a glioblastoma (GL261). Anti-tumor efficacies of an anti-PD1 antibody alone, SGT-53 alone or the combination of these agents were compared [7]. A number of markers with relevance to immune responses or tumor-induced immunosuppression were assessed by FACS or gene expression profiling.

**Results**

In all syngeneic models, SGT-53 increased tumor apoptosis and rendered the tumors immunologically “hot”. SGT- 53 plus anti-PD1 inhibited growth more than either single agent (Figure 2). The combination therapy dramatically reduced lung metastases by 4T1 breast tumors, while the anti-PD1 alone was ineffective. Based on relevant markers, SGT-53 treatment increased tumor immunogenicity, enhanced both innate and adaptive immune responses, and reduced tumor-induced immunosuppression. In mice bearing 4T1 tumors, injections of the anti-PD1 antibody killed the mice before tumors were themselves fatal. Addition of SGT-53 to the treatment regimen alleviated this fatal xenogeneic hypersensitivity to the anti-PD1 and extended the lives of 4T1-bearing mice (see Figure 3). We have identified genes that potentially underlie these observations.

**Conclusions**

Collectively, our data indicate that restoring p53 function via SGT-53 is able to boost anti-tumor immunity to enhance anti-PD1 immunotherapy by sensitizing tumors while reducing immune-related adverse events. Our data suggest that SGT-53, representing tumor-targeted TP53 gene therapy, has potential to augment immune checkpoint blockade agents while minimizing toxicity to improve outcomes in a variety of malignancies. Our data provide a strong mechanistic rationale for combining the investigational agent SGT-53 with checkpoint blockade agents in a clinical trial setting. It is possible that the SGT-53 would not only improve outcomes for cancer patients who already respond to immunotherapy, but also increase the percentage responding while minimizing adverse events related to the immunotherapy.

**References**

1. Mellman I, Coukos G, Dranoff, G, Cancer immunotherapy comes of age. Nature 2011; 480, 480-489.

2. Pardoll DM, The blockade of immune checkpoints in cancer immunotherapy. Nature Reviews Cancer 2012; 12, 252-264.

3. Lowe J, Shatz M, Resnick MA, Menendez D, Modulation of immune responses by the tumor suppressor p53. BioDiscovery 2013; 8, 2.

4. Munoz-Fontela C, Mandinova A, Aaronson SA, Lee SW, Emerging roles of p53 and other tumour-suppressor genes in immune regulation. Nature Reviews Immunology 2016; 16, 741-750.

5. Senzer D, Nemunaitis J, Nemunaitis D, Bedell C, Edelman G, Barve M, Nunan R, Pirollo KF, Rait A, Chang EH, Phase I study of a systemically delivered p53 nanoparticle in advanced solid tumors. Molecular Therapeutics 2013; 21, 1096-1103.

6. Pirollo K F, Nemunaitis J, Leung PK, Nunan R, Adams J, Chang EH, Safety and efficacy in advanced solid tumors of a targeted nanocomplex carrying the p53 gene used in combination with docetaxel: Phase 1b study. Molecular Therapeutics 2016; 24, 1697-1706.

7. Kim SS, Harford JB, Moghe M, Rait A, Chang EH, Combination with SGT-53 overcomes tumor resistance to a checkpoint inhibitor, OncoImmunology 2018; DOI: 10.1080/2162402X.2018.148498

**Ethics Approval**

All animal experiments were performed in accordance with and under approved Georgetown University GUACUC protocols.

**Fig. 1 (abstract P457).**
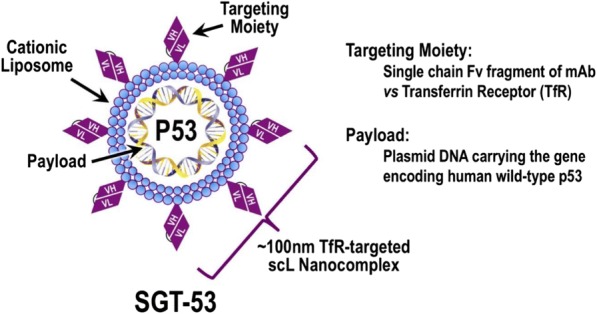
See text for description

**Fig. 2 (abstract P457).**
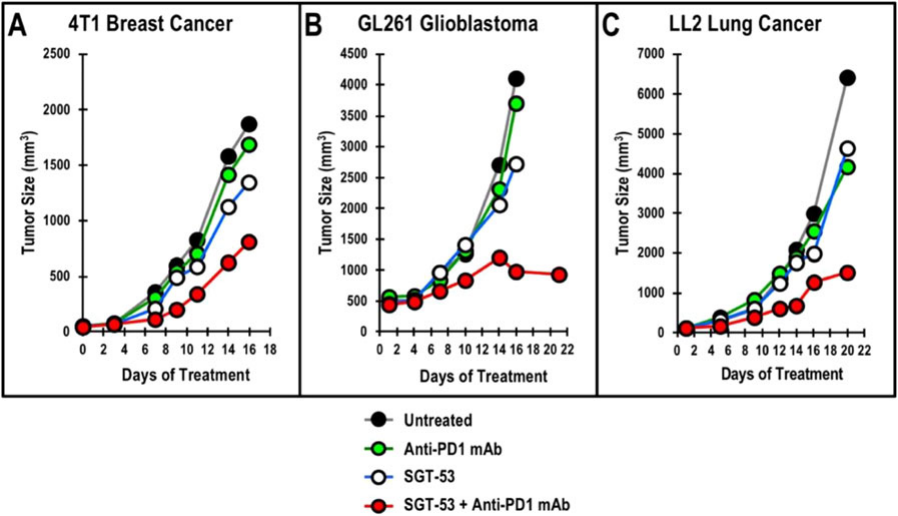
See text for description

**Fig. 3 (abstract P457).**
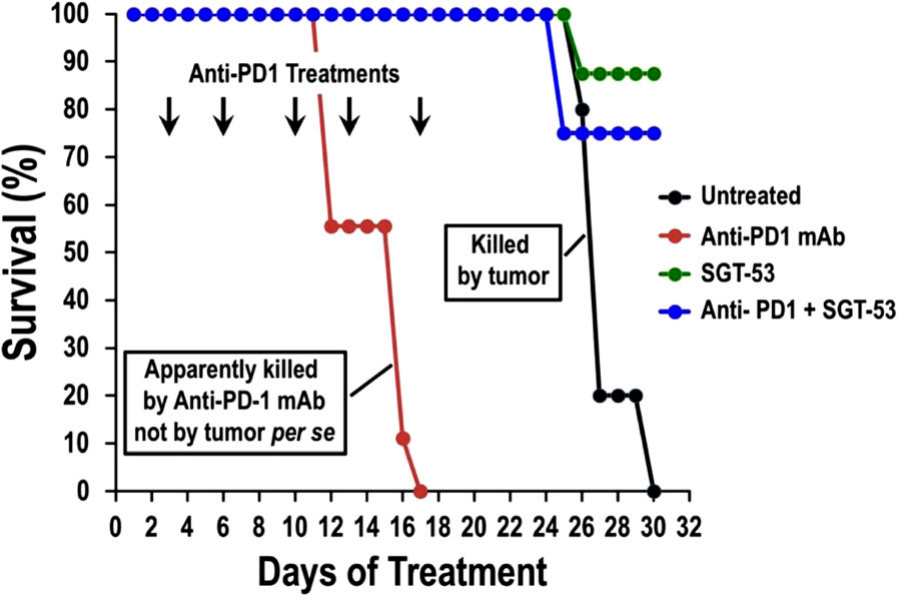
See text for description

#### P458 Contribution of an immune system to RACPP mediated drug delivery

##### Dina Hingorani, PhD, Maria Camargo, Matthew Doan, BS, Joesph Aguilera, Stephen Adams, Sunil Advani

###### University of California San Diego, La Jolla, CA, USA

####### **Correspondence:** Dina Hingorani (dhingoraniucsd@gmail.com)

**Background**

The interaction of tumor and host derived stroma is a complex process involving enzymes including matrix metalloproteinases (MMPs), specifically MMP2 / 9 [1]. The lack of efficacy from small molecule inhibitors to MMPs [2,3] and new approaches of macrophage mediated drug delivery strategies [4], propelled us to test the contributions of intrinsic tumor cell vs host stroma in the recruitment of macrophages in-vivo. We tested the hypothesis that drug delivering peptides that target the tumor-immune interplay have improved therapeutic efficacy and specificity.

**Methods**

Cy5:Cy7 FRET based ratiometric activatable cell penetrating peptides (RACPPs) were used to image syngeneic WT and MMP2/9 KO Polyoma middle T (PyMT) orthotopic tumors in C57/Bl6 WT and KO mouse models. The excised tumors were stained F4/80 pan-macrophage antibody markers. ACPP carrying the tumor radiosensitiser monomethyl auristatin E (MMAE) along with Beta integrin targeting moiety cRGD was injected in syngeneic lung tumor cell line (LL2) implanted in immune compromised athymic Nu/Nu mice and immune competent C57/Bl6 mice. The amount of released MMAE drug was analyzed in tumor vs surrounding normal tissue by LC-MS.

**Results**

The normalized Cy5/Cy7 ratio for the WT tumors established in WT mice (1.87 ± 0.11) was significantly higher than the ratio for the DKO tumors in WT mice (1.34 ± 0.07; p <0.003) or the DKO tumors in DKO mice (1.20 ± 0.08; p < 0.0002) (Figure 1A, 1B). As expected, the majority of the immune infiltration was at the periphery of the tumor with WT tumor having a significantly greater ability to recruit host macrophages into the tumor than DKO tumor cells (Figure 1C-1F). At 24 h, biodistribution measurements of released MMAE drug, revealed a higher tumor/muscle ratio of delivered drug (14 fold) in immune competent mice compared to immune-deficient mice(Figure 1G, 1H).

**Conclusions**

Interestingly, genotype tumor cells was more important than the host stromal component in promoting MMP-2/-9 activity in the tumors in this model system. Importantly, exploiting drugs that inhibit macrophage recruitment into tumors [4] and harnessing macrophage mediated drug delivery [5,6] in the tumor extracellular matrix may prove superior in eradicating tumors. In summary, our novel RACPP-drug conjugates can selectively localize to tumors and where they can be cleaved both by tumor cells and tumor-associated macrophages to provide improve the therapeutic index of systemically administered drugs [6,7].

**References**

1. Noël A, Jost M, Maquoi E. Matrix metalloproteinases at cancer tumor–host interface. InSeminars in cell & developmental biology 2008 Feb 1 (Vol. 19, No. 1, pp. 52-60). Academic Press.

2. Gura, Trisha. "Systems for identifying new drugs are often faulty." (1997): 1041-1042.

3. Richmond, Ann, and Yingjun Su. "Mouse xenograft models vs GEM models for human cancer therapeutics." (2008): 78-82.

4. Panni, Roheena Z., David C. Linehan, and David G. DeNardo. "Targeting tumor-infiltrating macrophages to combat cancer." Immunotherapy 5, no. 10 (2013): 1075-1087.

5. Li, Fu, Michelle Ulrich, Mechthild Jonas, Ivan J. Stone, Germein Linares, Xinqun Zhang, Lori Westendorf, Dennis R. Benjamin, and Che-Leung Law. "Tumor-Associated Macrophages Can Contribute to Antitumor Activity through FcγR-Mediated Processing of Antibody–Drug Conjugates." Molecular cancer therapeutics 16, no. 7 (2017): 1347-1354.

6. Buckel, Lisa, Elamprakash N. Savariar, Jessica L. Crisp, Karra A. Jones, Angel M. Hicks, Daniel J. Scanderbeg, Quyen T. Nguyen et al. "Tumor radiosensitization by monomethyl auristatin E: mechanism of action and targeted delivery." Cancer research 75, no. 7 (2015): 1376-1387.

7. Crisp, Jessica L., Elamprakash N. Savariar, Heather L. Glasgow, Lesley G. Ellies, Michael A. Whitney, and Roger Y. Tsien. "Dual targeting of integrin αvβ3 and matrix metalloproteinase-2 for optical imaging of tumors and chemotherapeutic delivery." Molecular cancer therapeutics 13, no. 6 (2014): 1514-1525.

**Ethics Approval**

All animal were performed under approved IACUC protocol number S15290 at the University of California San Diego.

**Fig. 1 (abstract P458).**
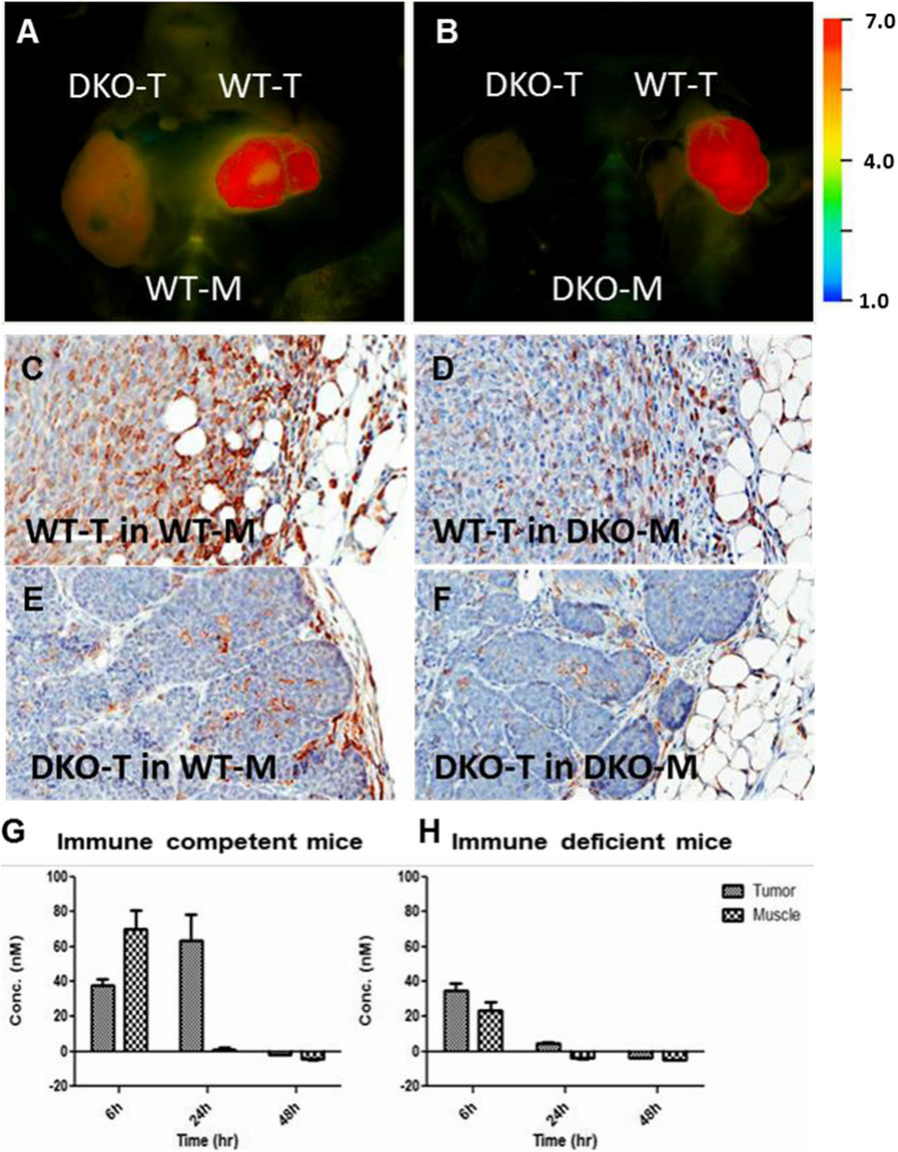
See text for description

#### P459 Detection of tumor-specific antibodies and their binding regions in mice cured from B78 melanoma

##### Anna Hoefges, MS^1^, Amy Erbe, PhD^1^, Drew Melby^1^, Alexander Rakhmilevich, MD, PhD^1^, Jacquelyn Hank, PhD^1^, Claire Baniel, BS, BA^1^, Clinton Heinze, BS^1^, Irene Ong, PhD^1^, Sean Mcilwain, PhD^1^, Hanying Li, PhD^2^, Richard Pinapati, PhD^2^, Bradley Garcia, PhD^2^, Jigar Patel, PhD^2^, Zachary Morris, MD, PhD^1^, Paul Sondel, MD, PhD^1^

###### ^1^University of Wisconsin Madison, Madison, WI, USA; ^2^Roche Sequencing Solutions, Inc., Madison, WI, USA

####### **Correspondence:** Anna Hoefges (hoefges@wisc.edu)

**Background**

Antibodies can play an important role in both innate and adaptive immune responses against cancer. We present a study that identifies possible new targets for antibody-based immunotherapy. We have developed a peptide array to assess potential protein-targets for antibodies that are activated in melanoma-cured mice through a combined immunotherapy regimen. By using Roche-Nimblegen’s unique technology, we were able to test antibody-reactivity to ~650 proteins, using 12 separate serum samples per array chip. This technology will enable us to accurately determine the linear peptide-binding sequences recognized by the anti-tumor antibodies produced in cured mice.

**Methods**

Mice bearing large GD2-expressing B78 melanoma tumors were treated with a triple-combination of immunotherapy capable of inducing an “in situ vaccine” effect, enabling mice to be cured of their tumors with long-term immune memory [1,2]. This triple combination therapy includes external beam radiation to the tumor, intratumoral injection of a tumor-specific immunocytokine (anti-GD2 mAb linked to IL2) and anti-CTLA-4. Serum was collected from mice when mice had macroscopic tumors, as well as after mice were cured of large tumors and rejected a re-challenge with the same tumor type. Using flow cytometry, mouse serum was tested for antibody- binding against B16 (parental cell line of B78). Afterwards, the serum was used on a Roche-Nimblegen peptide-array to determine specific antibody-protein binding sites and affinity towards the tumor.

**Results**

We analyzed sera from 4 mice that rejected established B78 tumors with this combination immunotherapy and compared their early-tumor and post-rejection serum antibody binding. We also included serum from mice bearing large tumors and analyzed the data generated by assessing differential expression in mice that rejected tumors vs mice that had large tumors or serum from naïve mice. Flow results showed increased signal after treatment. Multiple proteins of interest were selectively identified on the peptide array with sera from the 4 mice that rejected their tumors. We are continuing to investigate these proteins.

**Conclusions**

We were able to identify murine proteins that were selectively recognized by antibodies in mice that were cured of a tumor with immunotherapy but not by sera from to mice that were not cured of the same tumor or sera from naïve mice. The identified candidates may be new targets for antibody-based therapies, for adaptive recognition and could help in the development of new treatments.

**References**

1. Morris ZS, et al. Cancer Research, 76:3929-3941, 20162. Morris ZS, et al. Cancer Immunology Research, Published online, May 2018

#### P460 Chemotherapy induced immunogenic cell death and response to STING agonist in high-grade serous ovarian cancer

##### Sarah Nersesian, MSc, Nichole Peterson, MSc, Julie-Ann Francis, Madhuri Koti, DVM, MVSc, PhD

###### Queen's University, Kingston, ON, Canada

####### **Correspondence:** Sarah Nersesian; Madhuri Koti (kotim@queensu.ca)

**Background**

High Grade Serous Carcinoma of the ovary (HGSC) is mostly diagnosed at late stages and primarily treated with surgery followed by platinum/taxane-based chemotherapy. Unfortunately, majority of the patients exhibit resistance to chemotherapy and ultimately succumb to the disease. We previously demonstrated that chemotherapy naïve HGSC patient tumours with early recurrence show an immunosuppressed or immunologically cold pre-existing tumour immune microenvironment with decreased expression of genes involved in Type I Interferon (IFN1) and T helper type 1 response. We also reported the efficacy of a novel “Stimulator of Interferon Genes” agonist in combination with carboplatin chemotherapy and PD-1 immune checkpoint blockade using the ID8-Trp53-/- immunocompetent mouse model of HGSC. Based on previous reports on the distinct immunogenic cell death inducing potential of carboplatin and doxorubicin and that HGSC patients are treated with liposomal doxorubicin as a second line chemotherapy, the current study was performed to determine whether the effect of STING agonist can be further enhanced using a specific chemotherapy drug.

**Methods**

ID8-Trp53-/- and ID8-Trp53-/-;Brca1-/- cells were implanted in C57/BL6 immunocompetent mouse model of HGSC. At four-week time point established tumours were treated with carboplatin or doxorubicin chemotherapy followed by STING agonist treatment. Immune profiling was performed at early mid and late time points by measuring systemic responses in splenic immune cells, plasma cytokine profiles and tumour immune transcriptomic profiling. Overall survival was measured as per our previously established protocols.

**Background**

High Grade Serous Carcinoma of the ovary (HGSC) is mostly diagnosed at late stages and primarily treated with surgery followed by platinum/taxane-based chemotherapy. Majority of the patients exhibit resistance to chemotherapy and ultimately succumb to the disease. Contemporary immunotherapies targeting the PD-1/PD-L1 immune checkpoints have not proven be efficacious in HGSC patients. Based on our patient tumour based findings [1] that chemotherapy naïve HGSC patient tumours, with early recurrence and resistant to chemotherapy, show an immunologically cold pre-existing tumour immune microenvironment (TME), we conducted pre-clinical evaluation of a novel “Stimulator of Interferon Genes” (STING) agonist in combination with carboplatin chemotherapy and PD-1 immune checkpoint blockade using the ID8-Trp53-/- mouse model of HGSC [2]. This report demonstrated the potential of STING agonists in sensitization of ovarian tumours to PD-1 immune checkpoint blockade therapy, for ovarian cancer patients. Given the distinct immunogenic cell death (ICD) inducing potential of carboplatin and doxorubicin and that HGSC patients are treated with liposomal doxorubicin as a second line chemotherapy, the current study was performed to determine whether the effect of STING agonist can be further enhanced using a specific chemotherapy drug.

**Methods**

ID8-Trp53-/- cells were implanted in C57/BL6 immunocompetent mice. At four-week time point, established tumours were treated with carboplatin or doxorubicin chemotherapy followed by STING agonist treatment. A custom NanoString panel of 60 known ICD associated genes was used to measure the chemotherapy type related gene expression changes at early time point post single or combination treatments. Doxorubicin treated tumours showed significantly higher expression of Cxcl10, Cd274, Isg15, Psmb9 and Calr. Addition of STING agonist to each chemotherapy treatment showed significantly higher expression of Cxcl10 and IsG15 in the doxorubicin + STING agonist treated mice compared to carboplatin. Interestingly, Ccl5 gene expression was higher in the tumours from carboplatin treated mice compared to those treated with doxorubicin. Plasma cytokine profiles showed distinct profiles of interferon induced cytokines post treatment. Doxorubcin + STING agonist treated mice showed longer survival compared to carboplatin + STING agonist treated mice.

**Results**

Findings from our study demonstrate that efficacy of STING agonists can be further exemplified by selectively combining with potent ICD inducing chemotherapy.

**Conclusions**

Our study shows that clinical potential of STING agonists can be best achieved via combining with a potent ICD inducing chemotherapy and are key to the design of STING agonist based clinical trials.

**Acknowledgements**

This study was funded by the Canadian Institutes for Health Research and Early Research Award support to MK.

**References**

1. Au KK, Le Page C, Ren R, Meunier L, Clément I, Tryshkin K, Peterson N, Kendall-Dupont J, Childs T, Francis JA, Graham CH, Craig A, Squire JA, Mes-Masson AM, and Koti M. STAT1 induced intratumoural TH1 immunity predicts chemotherapy resistance in high‐grade serous ovarian cancer. Journal of Pathology: Clinical Research. 2016. Sep 19;2(4):259-270.

2. Haffari A, Peterson, Khalaj N NK, Robinson A, Francis JA and Koti M. STING agonist therapy in combination with PD-1 immune checkpoint blockade enhances response to carboplatin chemotherapy in high-grade serous ovarian cancer. British Journal of Cancer. 2018. Jul 26. doi: 10.1038/s41416-018-0188-5.

#### P461 Withdrawn

#### P462 Fractionated radiation with PD-1 blockade promotes anti-tumor activity in mouse head and neck cancer

##### Go Inokuchi, MD, PhD^1^, Elizabeth McMichael, PhD^2^, Masahiro Kikuchi, MD, PhD^1^, David Clump, MD PhD^1^ Robert Ferris^1^

###### ^1^University of Pittsburgh, Pittsburgh, PA; ^2^University of Pittsburgh Hillman Cancer Center, Pittsburgh, PA, USA

####### **Correspondence:** Robert Ferris (ferrrl@upmc.edu)

**Background**

Resistance to RT could be explained by the increased myeloid cells and upregulation of PD-L1 on tumor and myeloid cells. As 2Gy fractionated radiation therapy (RT) is the standard of care in HNSCC, pre-clinical investigations suggest that the addition of PD-1 blockade to RT could be clinically beneficial. Here, we investigated the immune response in a murine model of HNSCC to fractionated irradiation with or without PD-1 blockade.

**Methods**

Mice were inoculated with 2x106 murine tonsil epithelium E6/E7/H-ras transformed head and neck cancer cells (MEER) s.c. into both the neck and flank. Ten days following implantation, the neck tumor was irradiated with 20 Gy in 10 fractions. Anti-PD-1 therapy began following the initial dose of RT and continued every 3-4 days thereafter. Tumor growth was monitored and tumor volume was determined. Splenic and tumors tissues were collected 4 days after the final radiation dose for flow cytometric analysis.

**Results**

The effects of conventional 2Gyx10 fractionated RT was found to be greatly enhanced by the addition of PD-1 blockade, reducing tumor volumes by 7.2-fold. No clear abscopal effect on the non-irradiated flank tumor was observed. 2Gyx10 RT was better able to recruit myeloid and CD8+ T cells to the tumor site, an increase of 1.5-fold, as compared to 2Gyx5 fractionation. RT was shown to upregulate PD-L1 both on CD45- tumor cells and CD45+CD11b+ myeloid cells (p<0.05). Fractionated RT was also shown to increase CD8+ T cells activation through the production of IFN-gamma and TNF-alpha (p<0.001).

**Conclusions**

Concurrent PD-1 blockade with fractionated 2Gyx10 RT could activate the anti-tumor response in mouse head and neck cancer and warrants further investigation.

#### P463 HfO2 nanoparticles exposed to radiotherapy generate abscopal effect through activation of CD8+ T cells

##### Audrey Darmon, BS^1^, Ping Zhang, MD, PhD^1^, Sébastien Paris, PhD^1^

###### Nanobiotix, Paris, France

####### **Correspondence:** Sébastien Paris (sebastien.paris@nanobiotix.com)

**Background**

When exposed to radiotherapy (RT), nanoparticles of hafnium oxide (HfO2-NP) increase radiation dose deposition from within the cancer cells. HfO2-NP is intended for a single intratumor injection. Results of phase II/III in locally advanced Soft Tissue Sarcoma patients demonstrated a significant superiority and clinical benefits of HfO2-NP activated by radiotherapy compared to the standard of care, with a good local tolerance among this patient’s population, validating their first-in-class mode of action. HfO2-NP+RT is currently evaluated in six other clinical trials including head and neck, prostate, liver and rectum cancers. Moreover, preclinical studies have demonstrated that HfO2-NP+RT can generate the abscopal effect, where RT alone cannot. Here, we further explored the role of T cells infiltrates in the establishment of abscopal effect following HfO2-NP intratumor injection and activation with RT.

**Methods**

In a first experiment, CT26 (murine colorectal cancer cells) were subcutaneously injected in both flanks of BALB/c mice. Once the right tumors reached a mean tumor volume of 115±30 mm3, they were intratumorally injected with HfO2-NP (or vehicle) and irradiated 24 hours later with 4Gy per fraction for 3 consecutive days. Tumors from both flanks were collected 3 days after the last RT fraction and immune cell infiltrates were measured using immunohistochemistry (IHC) and digital pathology analyses.In order to investigate the specific role played by CD8+ T cells in the antitumor immune response and the abscopal effect, the experiment was subsequently repeated with CD8+ T cells depletion prior treatment with HfO2-NP+RT or RT alone (use of anti-CD8 antibody).

**Results**

In the first experiment, the abscopal effect was observed in the group treated with HfO2-NP+RT only. Correspondingly, IHC analyses showed a stark increase of CD8+ T cells infiltrates and other immune cells in both flanks of mice with HfO2-NP+RT, while RT alone had no significant effect.In the CD8+ T cells depletion experiment, no abscopal effect was observed. Besides, the control of the tumor treated with HfO2-NP + RT was less efficient than the control of the tumor treated with HfO2-NP+RT in absence of CD8+ T cells depletion.

**Conclusions**

These in vivo data suggest that the immunogenic conversion of the tumor microenvironment induced by HfO2-NP+RT triggers the abscopal effect through the activation of CD8+ T cells. HfO2-NP+RT may potentiate a pro- inflammatory environment suitable for immune enabling drugs: it may act as effective in-situ cancer vaccine and be combined with immunotherapeutic agents across oncology.

**Ethics Approval**

All experiments were approved by the Institutional Animal Care and Use Committee of Institut Gustave Roussy, approval number 2016_ 031_4340.

#### P464 Molecular targeted radiotherapy (MTRT) enhances the efficacy of immunotherapy increasing complete response rates of both local and distant disease in a “cold” tumor models

##### Ravi Patel, MD, PhD, Reinier Hernandez, PhD, Peter Carlson, Ryan Brown, Abigail Jaquish, Luke Zangl, Raghava Sriramaneni, PhD, Joseph Grudzinski, PhD, Bryan Bednarz, PhD, Jamey Weichert, PhD, Paul Sondel, MD, PhD, Zachary Morris, MD, PhD

###### University of Wisconsin, Madison, WI, USA

####### **Correspondence:** Zachary Morris (zmorris@humonc.wisc.edu)

**Background**

Studies have shown that in some immunologically “cold” tumor models, distant disease can suppress the effect of in situ vaccines (IS) even at the primary site[1]. This may be overcome by delivering low dose radiotherapy (RT) to all tumor sites; yet delivering large field RT to metastatic disease can cause systemic lymphopenia. We have developed a strategy using a molecular targeted RT (MTRT), Y90-NM600 (YN6), that has selective uptake in nearly any tumor type or location to deliver RT to all sites of disease in a functionally “cold” metastatic tumor model.

**Methods**

Large (~150-200 mm3) B78 melanoma primary tumors and occult secondary (non-palpable at treatment) as well as B16 melanoma lung metastases were established in syngeneic mice. Combinations of immune checkpoint inhibition (ICI; anti-CTLA-4 and anti PD-1), IS (12 Gy RT + IT anti-GD2-mAb + IL2), or MTRT (50 μCi) were given [Figure 1]. Tumor growth was tracked to day (D) 30, Survival to D60, and mice with complete response (CR) were re- challenged with injection of B78 cells (D90) and unrelated Panc02 cells(D120). Tumor growth and survival studies were replicated in syngeneic 4T1 breast and NXS2 neuroblastoma models. Mechanistic studies using T-cell depletion, whole body external beam RT (WBEBRT), histology, and gene expression profiling were conducted.

**Results**

Tumor response was significantly improved with the addition of MTRT to each group, with highest response rate in the triple combination treatment group which had a CR as well as tumor specific immune memory in 83% of mice (p < 0.05). Development of secondary tumors and distant metastatic disease was also reduced in the triple combination treatment group (ICI + IS + MTRT), while dual treatment groups had varying levels of efficacy in treating primary, occult secondary, or metastatic disease [Figure 2]. Similar response and survival was replicated in 4T1 and NXS2 models with addition of MTRT. T-cell depletion revealed a reversal of the enhanced response seen with MTRT. Unlike MTRT, delivering WBEBRT did not enhance efficacy of immunotherapy. QPCR of MTRT gene expression demonstrated upregulation of STING/IFN/apoptosis pathways (Mx1/Ifnb/PDL1/DR5/ICAM1) that were greater than that achieved with equivalent doses of EBRT. Histological analysis of tumor samples showed significantly increased CD8+ infiltrates in the combination treatment group (p < 0.05).

**Conclusions**

Our results demonstrate that MTRT can effectively stimulate and enhance the generation of an immune response to combination IS and ICI immunotherapy treatments, enabling tumor eradication at primary, occult secondary, and metastatic sites of disease.

**Acknowledgements**

RSNA Fellow Award, ASCO Young Investigator Award, UW 20/20 Award, UW Cancer Center Core Grant

**References**

1. Morris, ZS, et al. Cancer Immunol Res 2018 Jul;6(7):825-34

**Ethics Approval**

This study was approved by the UW Institutional Animal Care and Use Committee.

**Fig. 1 (abstract P464).**
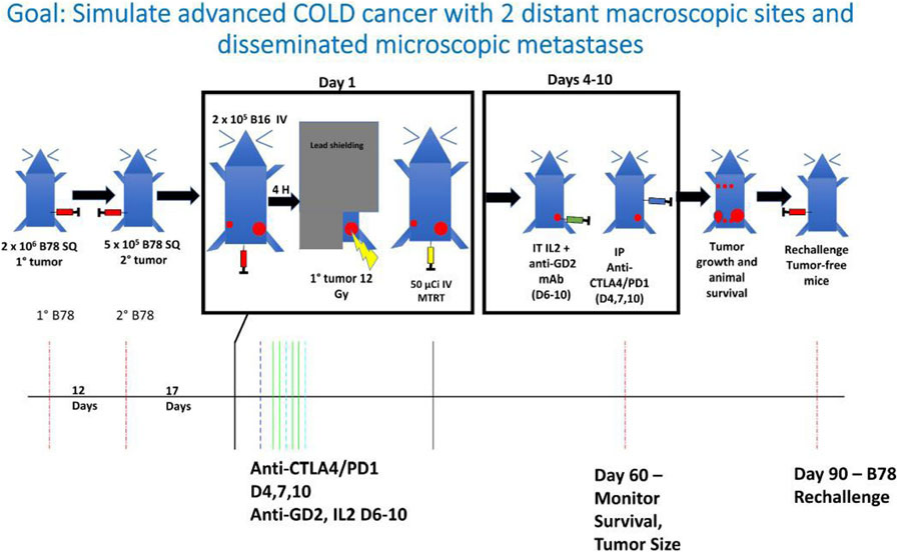
Treating mice with 1 large, 1 small and disseminated COLD B78/B16 tumors

**Fig. 2 (abstract P464).**
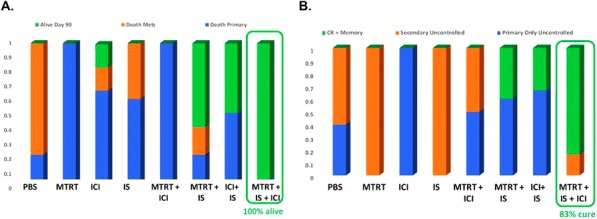
See text for description

#### P465 Comparison of peripheral immune response during chemoradiotherapy (CRT) with and without PD-1 blockade in patients with head and neck squamous cell carcinoma (HNSCC)

##### Juan Callejas-Valera, PhD^1^, Juan Callejas-Valera, PhD^1^, Daniel Vermeer^1^, Christopher Lucido^2^, Caitlin Williamson^1^, Marisela Killian^1^, William Spanos, MD^1^, Paola Vermeer, PhD^1^, Steven F. Powell, MD^3^

###### ^1^Sanford Research, Sioux Falls, SD, USA; ^2^University of South Dakota, Sioux Falls, SD, USA; ^3^Sanford Cancer Center, Sioux Falls, SD, USA

####### **Correspondence:** Steven F. Powell (Steven.Powell@sanfordhealth.org)

**Background**

While inhibitors of the programmed death-1 and its ligands (PD-1 and PD-L1/2) are active in recurrent/metastatic (R/M) HNSCC, their effects during curative intent therapy are unknown. Previous translational data demonstrated that standard, high-dose CRT decreases circulating CD4+ and CD8+ T-cell populations while increasing PD-1 expression and myeloid derived suppressor cells (MDSCs) [1]. To overcome this suppressive immunophenotype, we developed a clinical trial exploring the combination of the PD-1 inhibitor, pembrolizumab, with CRT using a low- dose chemotherapy regimen. Here we present data comparing the peripheral blood immune response during this novel therapy to standard CRT.

**Methods**

We evaluated peripheral blood mononuclear cells (PBMCs) from HNSCC patients from two clinical trials (NCT02586207, NCT01386632) and healthy volunteers (controls) to compare the peripheral blood immune response during CRT. Trial 1 used low-dose cisplatin (40 mg/m2 weekly x 6 doses) with pembrolizumab and Trial 2 used standard high-dose cisplatin (100 mg/m2 every 3 weeks x 3 doses) without PD-1 inhibition. We compared circulating immunocytes, including CD4+ and CD8+ T-cells, regulatory T-cells (T-regs), and MDSCs, utilizing multi-color flow cytometry at baseline, during (mid-treatment) and after (3 months post-radiation) CRT. Immune checkpoint expression (PD-1, TIM3, LAG3) on CD4/CD8+ cells was also compared between the groups. Changes in memory T-cell populations (effector memory; EM, central memory; CM, and effector memory RA; EMRA) were also evaluated.

**Results**

18 patient samples from trial 1 and 15 samples from trial 2 were viable for evaluation. Comparing the two treatments, there was no significant difference in key immunocyte populations during therapy (Figure 1). However, there was a significant decline in PD-1 expressing CD4+ and CD8+ T-cell populations during treatment with PD-1 inhibition and low-dose chemotherapy compared to standard treatment (Figure 2). Expression of other markers of immune exhaustion (TIM3, LAG3) rise in both groups throughout treatment. Memory T-cell populations (Figure 3) show that the pembrolizumab-based treatment increased the percentage of both EM and EMRA helper T-cells in contrast with standard treatment.

**Conclusions**

Our data demonstrates that the addition of a PD-1 inhibitor to a low-dose cisplatin CRT regimen can reduce circulating PD-1+ T-cells during therapy. However, these populations, as well as other markers of T-cell exhaustion rise by the end of therapy and could play a role in immune escape. Further characterization of this immune response is needed to determine the best approach to add novel immunotherapy agents in this treatment setting.

**Acknowledgements**

Sanford Research Flow Cytometry CoreSupported by a Center of Cancer Biology Research Excellence (COBRE) grant (5P20GM103548-08) from the National Institutes of Health

**References**

1. Parikh F, Duluc D, Imai N, Clark A, Misiukiewicz K, Bonomi M, Gupta V, Patsias A, Parides M, Demicco EG, Zhang DY, Kim-Schulze S, Kao J, Gnjatic S, Oh S, Posner MR, Sikora AG. Chemoradiotherapy-induced upregulation of PD-1 antagonizes immunity to HPV-related oropharyngeal cancer. Cancer Res. 2014;74(24):7205- 16.

**Ethics Approval**

Trial 1 (NCT02586207) was approved by the Western Institutional Review Board (WIRB), approval number 20152167. Trial 2 (NCT01386632)was approved by the Sanford Research Institutional Review Board (IRB), approval number MODCR00000588.

**Fig. 1 (abstract P465).**
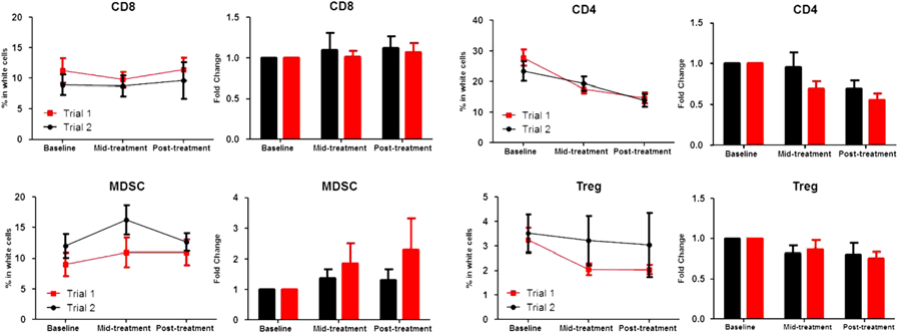
Peripheral Blood Immunocyte Response during CRT

**Fig. 2 (abstract P465).**
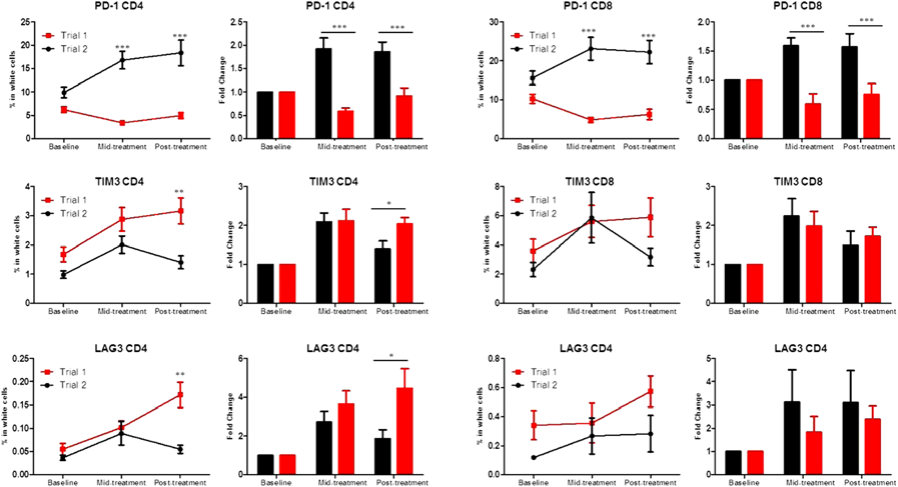
Changes in Immune Checkpoint Expression

**Fig. 3 (abstract P465).**
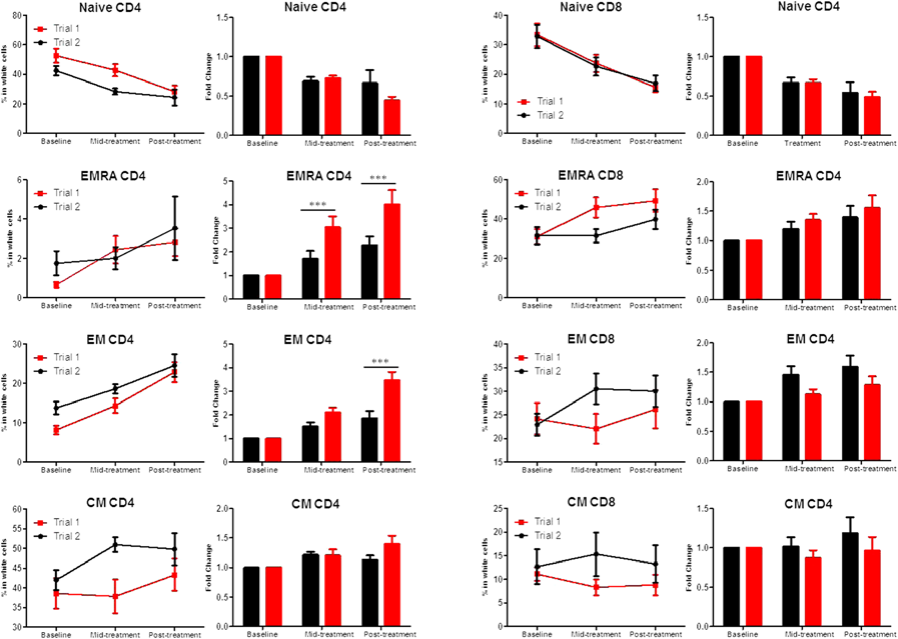
The Effect on Memory T-cell Response

#### P466 TGF beta blockade enhances radiotherapy abscopal efficacy effects in combination with anti-PD1 and anti- CD137 immunostimulatory monoclonal antibodies

##### Inmaculada Rodriguez^2^, Lina Mayorga^1^, Tania Labiano^3^, benigno Barbes^1^, inaki etxeberria^2^, Mariano Ponz-Sarvise^1^, arantza Azpilicueta^2^, elisabeth Bolanos^2^, Miguel F.Sanmamed^1^, Pedro Berraondo^2^, Felipe Manuel Alfonso Calvo^1^, Mary Helen Barcelos-Hoff^4^, Jose Luis Perez-Gracia^1^, Ignacio Melero, MD, PhD^3^, Maria Rodriguez-Ruiz, MD, PhD^1^

###### ^1^Clinica Universidad de Navarra, Pamplona, Spain; ^2^Centro de Investigacion Medica Aplicada, Pamplona, Spain; ^3^Complejo Hospitalario de Navarra, Pamplona, Spain; ^4^University of California, San Francisco, San Francisco, CA, USA

####### **Correspondence:** Ignacio Melero (imelero@unav.es)

**Background**

Radiotherapy can be synergistically combined with immunotherapy in mouse models, extending its efficacious effects outside of the irradiated field (abscopal effects) [1]. We previously reported that a regimen encompassing local radiotherapy in combination with anti-CD137 plus anti-PD-1 mAbs achieves potent abscopal effects against syngeneic transplanted murine tumors up to a certain tumor size. Knowing that TGFβ expression or activation increases in irradiated tissues, we tested whether TGFβ blockade may further enhance abscopal effects in conjunction with the anti-PD-1 plus anti-CD137 mAb combination [2].

**Methods**

Mice bearing bilateral MC38 or 4T1 tumors were randomly assigned to 6 groups receiving or not radiotherapy (8Gy/3fx), in combination or not with intraperitoneal antibodies (anti-PD1 plus anti-CD137 and/or anti-TGFβ). Tumor tissue was processed to obtain single-cell suspensions for flow cytometry analyses Levels of TGFβ-1 in mouse tumor tissue homogenates and IFNγ in mouse plasma samples were measured by commercial ELISAs.

**Results**

TGFβ blockade with 1D11, a TGFβ neutralizing monoclonal antibody, markedly enhanced abscopal effects and overall treatment efficacy against subcutaneous tumors of either 4T1 breast cancer cells or large MC38 colorectal tumors. Increases in CD8 T cells infiltrating the non-irradiated lesion were documented upon combined treatment, which intensely expressed Granzyme-B as an indicator of cytotoxic effector capability.

**Conclusions**

Radiotherapy-induced TGFβ hampers abscopal efficacy even upon combination with a potent immunotherapy combination. Therefore TGFβ blockade in combination with radioimmunotherapy regimens results in greater efficacy.

**Acknowledgements**

We are in debt to Dr. Alan Korman (BMS, San Francisco CA) for his kind gift of monoclonal antibodies. We acknowledge generous help from Drs. Martinez-Monge, Aristu, Castañon and Gil-Bazo from the department of oncology at CUN. Excellent dosimetry by Arantza Zubiria and dedicated animal care by Eneko Elizalde are also acknowledged.

**Trial Registration**

NA

**References**

1. Rodriguez-Ruiz ME, Rodriguez I, Barbes B. Abscopal effects of radiotherapy are enhanced by combined immunostimulatory mAbs and are dependent on CD8 T cells and crosspriming. Cancer Res. 2016 Oct 15;76(20):5994-6005.

2. Vanpouille-Box C, Diamond JM, Pilones KA. TGFβ is a master regulator of radiation therapy-induced antitumor immunity. Cancer Res. 2015 Jun 1;75(11):2232-42.

**Ethics Approval**

The study was approved by Navarra Institutution‘s Ethics Board, approval number 117/14

#### P467 Augmenting immunity with IAP antagonists in PDAC

##### Kevin Roehle, PhD^1^, Michael Dougan, MD, PhD^1^, Stephanie Dougan, PhD^2^

###### ^1^Dana-Farber Cancer Institute, Bosotn, MA, USA; ^2^Massachusetts General Hospital, Boston, MA, USA

####### **Correspondence:** Michael Dougan (mldougan@partners.org)

**Background**

Pancreatic ductal adenocarcinoma (PDAC) is responsible for about 7% of all cancer-related deaths in the US. PDACs are fibrotic and dense tumors with little vasculature, and are rapidly metastatic. Thus far, cancer- immunotherapy with immune checkpoint blocking antibodies have largely failed in PDAC. The Inhibitor of Apoptosis (IAP) protein family comprises a diverse group of proteins, many of which have immunoregulatory roles. IAP antagonists are small molecule drugs that primarily inhibit cellular (c-)IAP1 and c-IAP2 protein leading to TNFa mediated apoptosis in tumor cells through alternative NF-kB signaling. In immune cells IAP antagonism leads to increased alternate NF-kB signaling, leading to increased survival of B cells, activation of dendritic cells and supporting activation of T cells in a costimulatory manner.

**Methods**

We evaluated the effects of the IAP antagonist LCL-161 in multiple syngeneic models of pancreatic cancer.

**Results**

Although LCL-161 did not induce TNFa mediated apoptosis in any of our tumor cell lines in vitro, we were able to induce robust immune-mediated regressions in an orthotopic and subcutaneous tumor models.

**Conclusions**

These responses were dependent on CD8 and CD4 T cells, and we show evidence for a direct effect of LCL-161 in augmenting T cell priming in pancreatic cancer.

#### P468 Transcriptomic profiles conducive to immune-mediated tumor rejection in human breast cancer skin metastases treated with Imiquimod

##### Mariya Rozenblit, MD^1^, Wouter Hendrickx, PhD^2^, Adriana Heguy^3^, Luis Chiriboga^3^, Cynthia Loomis^3^, Karina Ray^3^, Farbod Darvishian, MD^3^, Mikala Egeblad, PhD^4^, Davide Bedognetti, MD, PhD^5^, Sylvia Adams, MD^3^

###### ^1^Yale University, Connecticut, CT, USA; ^2^Sidra Medicine, Doha, Qatar; ^3^NYU, New York, USA; ^4^Cold Spring Harbor Laboratory, Cold Spring Harbor, NY, USA; ^5^SIDRA, Doha, Qatar

####### **Correspondence:** Mariya Rozenblit (Mariya.Rozenblit@yale.edu)

**Background**

Imiquimod is a topical toll-like-receptor-7 agonist currently used for treating basal cell carcinoma. Recently, imiquimod has demonstrated tumor regression in melanoma and breast cancer skin metastases. However, the molecular perturbations induced by imiquimod in breast cancer metastases have not yet been characterized. Here, we describe transcriptomic profiles associated with responsiveness to imiquimod in breast cancer skin metastases.

**Methods**

Baseline and post-treatment tumor samples from eight patients treated with imiquimod in a clinical trial were profiled using Nanostring nCounter ® Human v1.1 PanCancer Immune Profiling Panel. Two of the patients had stable disease during the initial study and were found to have a systemic complete clinical response after subsequent treatment with fulvestrant after study completion which continued for several years. On follow up, these two patients also had disease remission for two years. An additional patient had a local partial anti-tumor response after eight weeks of imiquimod treatment and was labeled as a partial responder (PR). Five of the eight patients did not have an anti-tumor response and were defined as non-responders (NR). An integrative analytic pipeline was used to analyze gene expression data including pathway analysis and deconvolution.

**Results**

We showed that tumors from patients who achieved a durable clinical response displayed a permissive microenviroment, substantiated by the upregulation of transcripts encoding for molecules involved in leukocyte adhesion and migration, cytotoxic functions, and antigen presentation (Figure 1AB). Imiquimod triggered a strong T-helper-1 (Th-1)/cytotoxic immune response, characterized by the coordinated upregulation of Th-1 chemokines, migration of Th-1 and cytotoxic T cells into the tumor, and activation of immune-effector functions, ultimately mediating tumor destruction (Figure 1C).

**Conclusions**

Topical imiquimod can induce a robust immune response in breast cancer metastases, and this response is more likely to occur in tumors with a pre-activated microenvironment. In this setting, imiquimod could be utilized in combination with other targeted immunotherapies to increase therapeutic efficacy.

**Acknowledgements**

The work was supported by the AMA Foundation Seed Grant

**Ethics Approval**

The clinical trial was approved by the New York University Institutional Review Board

**Fig. 1 (abstract P468).**
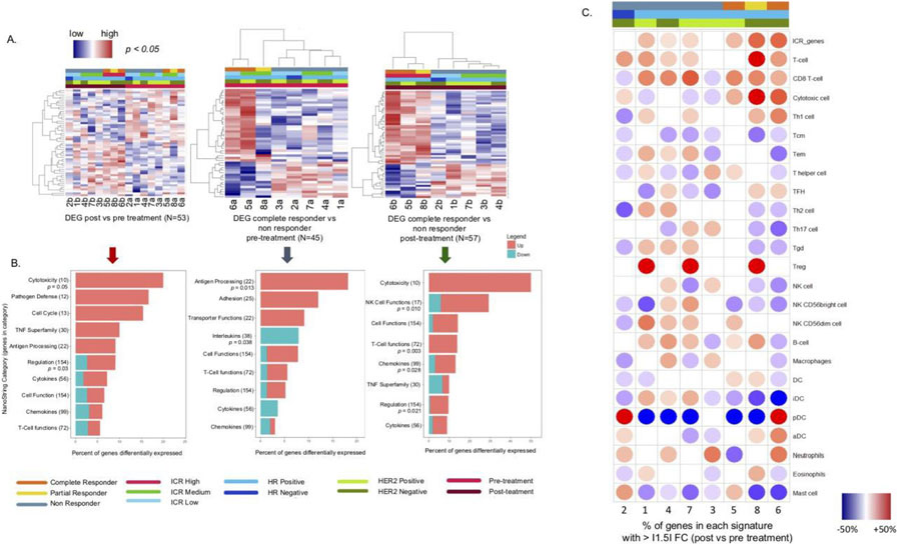
See text for description

#### P469 TCR repertoire correlates of response in tumor-bearing mice treated with radiotherapy and CTLA-4 blockade

##### Nils-Petter Rudqvist, PhD^1^, Claire Lhuillier, PhD^1^, Erik Wennerberg, PhD^1^, Jennifer Sims, PhD^2^ , Sandra Demaria, MD^1^

###### ^1^Weill Cornell Medical College, New York, NY, USA; ^2^Memorial Sloan Kettering Cancer Center, New York, NY, USA

####### **Correspondence:** Sandra Demaria (szd3005@med.cornell.edu)

**Background**

Tumor-targeted radiation therapy (RT) in combination with immune checkpoint blockade can activate tumor-specific T-cells to reject tumors. Yet, predictive features of effectively primed T cell repertoires (TCR) remain poorly understood. Using the 4T1 mouse model of triple negative breast cancer, where RT+CTLA-4 blockade elicits an anti-tumor T cell response that controls both the irradiated tumor and non-irradiated lung metastases and extends survival, we previously reported increased intratumoral CD8/CD4 ratio and CD8+ T cell clonality following RT+anti-CTLA-4 treatment [1]. Here, we determined the longitudinal changes of the TCR repertoires in the 4T1 carcinoma and its correlates with treatment response.

**Methods**

To analyze longitudinally the TIL repertoire before and after treatment with RT+anti-CTLA-4, mice were inoculated in both flanks with 4T1 cells (n=8/group). One tumor was resected 2 days before treatment (pre-TX) and the other was treated with RT (3X8 Gy) or anti-CTLA-4 antibody (3x200 μg i.p.) monotherapy or in combination and resected 1 day after treatment when immune-mediated tumor rejection is occurring in tumors treated with RT+anti- CTLA-4 (post-TX). No local tumor recurrence was observed, but mice succumbed of lung metastasis with the largest increase in survival (vs. untreated) in mice given RT+anti-CTLA-4 (p=0.0041). To assess the TIL TCR repertoire, dual-stage PCR amplification and high-throughput sequencing of the TCRa and b CDR3 regions was performed using mRNA isolated from total tumor.

**Results**

In tumors treated with RT and RT+anti-CTLA-4, both the TCRa and b repertoires increased in clonality compared to pre-TX, whereas a smaller increase in TCRb clonality was found after anti-CTLA-4 monotherapy. We have previously characterized the TCRb repertoire of expanded and activated CD8+ T cells recognizing the AH1 epitope from gp70 antigen (a tumor antigen expressed by 4T1 cells) in tumors of mice treated with RT+anti-CTLA-4 [1]. Using GLIPH [2], we identified a major AH1-specific CDR3b motif and found it present in pre-TX tumors of all animals, and its frequency increased in mice treated with RT or RT+anti-CTLA-4, consistent with our previous findings. In contrast, the V-J Jensen-Shannon divergence, assessed excluding AH1-specific T cells, was higher between pre- and post-TX tumors in all mice treated with RT, (independent of anti-CTLA-4 treatment) and correlated strongly with survival (cox regression; p=0.00054), suggesting priming and expansion of T cells targeting antigens other than AH1.

**Conclusions**

Together these data support the dominant role of RT in priming emergent or low-abundance T cell clonotypes, rather than the driving of already-prevalent clonotypes.

**References**

1. Rudqvist NP, Pilones KA, Lhuillier C, Wennerberg E, Sidhom JW, Emerson RO, Robins HS, Schneck J, Formenti SC, Demaria S. Radiotherapy and CTLA-4 blockade shape the TCR repertoire of tumor-infiltrating T cells. Cancer Immunol Res. 2018; 6(2): 139-150.

2. Glanville J, H. Huang A, Nau O, Hatton LE, Wagar F, Rubelt X, Ji A, Han SM, Krams C, Pettus N, Haas CSL, Arlehamn A, Sette SD, Boyd TJ, Martinez S, Davis MM. Identifying specificity groups in the T cell receptor repertoire. Nature. 2017; 547(7661): 94-98.

**Ethics Approval**

All experiments were approved by the Weill Cornell Medicine Institutional Animal Care and Use Committee, approval number 2015-0028.

#### P470 Targeting DNA damage response promotes anti-tumor immunity through STING-mediated T-cell activation in small cell lung cancer

##### Triparna Sen, PhD^1^, Bertha Leticia Rodriguez^1^, Limo Chen, PhD^1^, Naoto Morikawa^1^, Junya Fujimoto^1^, Lixia Diao^1^, Youhong Fan^1^, Jing Wang^1^, Bonnie Glisson^1^, Ignacio Wistuba, MD^1^, Julien Sage^2^, John Heymach^1^, Don Gibbons, MD^1^, Lauren Byers, MD^1^

###### ^1^UT MD Anderson Cancer Center, Houston, TX, USA; ^2^Stanford University, Houston, TX, USA

####### **Correspondence:** Lauren Byers (lbyers@mdanderson.org)

**Background**

Despite recent advances in the use of immunotherapy, only a minority of small cell lung cancer (SCLC) patients respond to immune checkpoint blockade (ICB) with programmed cell death protein 1 (PD-1) or programmed death ligand 1 (PD-L1) antibodies as monotherapy or combination. We have previously established that SCLC is vulnerable to agents targeting the DNA damage response (DDR) pathway, including inhibitors of PARP and CHK1, and these findings have led to several clinical trials of DDR inhibitors for SCLC. Our recent findings demonstrate that CHK1 inhibitor, prexasertib, potentiate the anti-tumor response of PD-L1 blockade in SCLC. In the present study we have established the efficacy of combined PARP (olaparib) and PD-L1 blockade and further elucidated the underlying mechanism of DDR-mediated anti-tumor immune response in SCLC models.

**Methods**

SCLC models were treated with small molecule inhibitors prexasertib or olaparib with or without anti-PD-L1. End point analyses were done by immunoblot, immunohistochemistry, qRT-PCR, flow cytometry and reverse phase protein array (RPPA).

**Results**

We treated the tumor bearing B6129F1 mice with either IgG, olaparib (100mg/kg,4/7), anti-PD-L1 or combination. By 10days, all mice treated with olaparib+anti-PD-L1 combination had a complete tumor regression with no tumor growth upto 80days. Co-targeting DDR+PD-L1 significantly increased the level of CD8+ cytotoxic-T cell infiltration and decreased PD-1+/TIM3+ exhausted T-cell population (p<0.0001). CD8 depletion was able to reverse the anti-tumor effect of the combination demonstrating the role of cytotoxic T-cell infiltration and function in DDR+PD-L1 response. Next, we established, by genetic knockdown studies, that DDR-mediated immune response was facilitated through activation of the STING/TBK1/IRF3 innate immune response pathway. STING pathway activation enhanced expression of Type-1 interferon gene IFNβ and downstream chemokines, CXCL10/CCL5 and resulted in T-cell recruitment.

**Conclusions**

Our results demonstrate,for the first time, the remarkable efficacy of the combination of PD-L1 blockade with PARP/CHK1 inhibition and provide a strong scientific rationale for combining these modalities in clinical trials for SCLC patients. Combining DDR inhibition with ICB leads to T-cell recruitment and enhanced effector cell function in SCLC tumors, mediated by the activation of the innate immune response pathway STING/TBK1/IRF3 and increased IFNβ. The immune profiling and correlative biomarker data from our study provide valuable mechanistic insight and indicate the subset of the patient population who are most likely to respond to these treatments. Because prexasertib, olaparib and other PARP inhibitors are already in clinical trials for SCLC, we expect that this hypothesis has the potential for rapid translation into the clinic.

#### P471 Mertk is a therapeutic target in combination with radiation to promote adaptive immune tumor responses

##### Garth Tormoen, MD, PhD^1^, Jason Baird, PhD^2^, Gwen Kramer, BS^2^, Shelly Bambina^2^, Marka Crittenden, MD, PhD^2^, Michael Gough, PhD^2^

###### ^1^Oregon Health & Science University, Portland, OR, USA; ^2^Earl A. Chiles Research Institute, Portland, OR, USA

####### **Correspondence:** Garth Tormoen (tormoeng@ohsu.edu)

**Background**

Mertk is a member of the Tyro3-Axl-Mertk (TAM) family of receptors and regulates phagocytosis of dying cells by macrophages. Cancer cells killed by radiation therapy direct repolarization of macrophages into immune suppressive phenotypes. Mertk-/- mice grafted with immunogenic tumors have enhanced tumor control following ionizing radiation compared to Mertkwt mice. Gas6 is the endogenous ligand for Mertk and its ability to signal through Mertk requires a post-translational vitamin k-dependent modification that is inhibited by warfarin.

**Methods**

Mertk-/- and WT mice were injected subcutaneously in the flank with 5E4 CT26 cells (BALB/c) or 5E6 Panc02-SIY cells (C57BL/6) and allowed to grow to 5 mm before treatment with 250 μg anti-CD8α antibodies, warfarin (0.5 mg/L drinking water) and subjected to a single dose of ionizing radiation (16 Gy) followed by 250 μg of OX40 or PBS I.P. 1-day post-RT. Peripheral blood was collected 6 days after RT and evaluated by Flow Cytometry for SIY- pentamer+CD8+ T cells.

**Results**

Radiation therapy results in tumor control in BALB/c mice, but tumor cure in Mertk-/- BALB/c mice. Tumor cure in Mertk-/- BALB/c mice was abrogated by depletion of CD8 T cells indicating that ligation of Mertk in tumor macrophages suppresses endogenous anti-tumor immunity following radiation therapy. Similarly, warfarin-treated mice had higher rates of tumor cure following radiation that was also abrogated by CD8 depletion. In C57BL/6 mice, Mertk-/- alone does not affect responses to radiation therapy in the Panc02 tumor model, but the combination of radiation therapy with anti-OX40 costimulation of T cell responses resulted in a significant increase in peripheral blood SIY+ CD8 T cells 5 days after treatment, and significantly improved survival compared to radiation alone.

**Conclusions**

Mertk-/- mice, and Mertkwt mice treated with warfarin to inhibit Gas6 experience increased tumor control following ionizing radiation in an adaptive-immune mediated manner in CT26 tumor models. In less immunogenic tumors, loss of Mertk-/- permitted tumor cure following radiation therapy when combined with the T cell costimulatory molecule OX40. These data demonstrate that Mertk suppresses adaptive immunity in irradiated tumors. Mertk is an attractive therapeutic target in combination with ionizing radiation and immune therapy to promote adaptive immune anti-tumor responses.

**Ethics Approval**

All animal studies were approved by the Earl A. Chiles Research Institute IACUC, Assurance No. A3913-01.

#### P472 Immunogenic tumor antigen is required in antitumor effect of cisplatin monotherapy and its combination with anti-PD-L1

##### Daiko Wakita, PhD^1^, Toshiki Iwai, BS^1^, Masamichi Sugimoto, PhD^1^, Osamu Kondoh^1^

###### Chugai pharmaceutical CO., LTD., Kamakura, Japan

####### **Correspondence:** Osamu Kondoh (kondoosm@chugai-pharm.co.jp)

**Background**

Although anti-PD-L1/PD-1 immunotherapy has shown marked clinical effect in a broad range of cancer, a subset of patients respond to monotherapy, and tumor mutation burden have been identified as potential predictive marker for responders. To extend the clinical benefits, combination of anti-PD-L1 and chemotherapy has been actively investigated. However the association of immunogenicity of neoantigen with antitumor effects of the combination of immunotherapy and chemotherapy is remained unknown. Here we investigated tumor antigen-specific T cell responses and antitumor effect of the anti-PD-L1 plus cisplatin combination therapy in mouse tumor models.

**Methods**

E.G7-OVA cell, expressing ovalbumin (OVA) gene as an immunogenic model tumor antigen, and its parental less immunogenic EL4 cell were subcutaneously inoculated into C57BL/6 mouse. The tumor-bearing mice were intraperitoneally treated with anti-mouse PD-L1 mAb (anti-PD-L1; 10 mg/kg, three times a week) and cisplatin (CDDP; 1 mg/kg, once at Day1). For CD8+ cell depletion experiments, the tumor-bearing mice were treated with CDDP (4 mg/kg, once at Day1) and anti–mouse CD8 mAb (twice a week from one day before the treatment initiation). To evaluate cytolytic activity, CD8+ T cells isolated at Day7 were co-cultured with CFSE labeled-tumor cells and the frequency of dying-tumor cell was measured by flow cytometry.

**Results**

Anti-PD-L1 alone and CDDP alone exhibited significant antitumor effect in E.G7-OVA-bearing mice and the combination therapy resulted in further effect than the each monotherapy at Day15. In parallel with the therapeutic effect, CD8+ T cells from tumor-draining lymph node exhibited higher cytolytic activity against E.G7-OVA in monotherapy group than that in control group, and highest cytolytic activity was observed in combination group. In contrast, even after the combination therapy, cytolytic activity against parental EL4 cells was hardly detected. In addition, less immunogenic EL4 were insensitive to monotherapies with anti-PD-L1, CDDP and their combination. Moreover, in immunogenic E.G7-OVA-bearing model, the higher dose of CDDP (4 mg/kg) showed direct cytolytic activity as well as CD8+ T cell dependent antitumor effect, whereas only direct cytotoxic effect was observed in EL4-bearing model.

**Conclusions**

In our model, not only anti-PD-L1 alone but also CDDP alone enhanced T cell responses against immunogenic tumor antigen (OVA) but not neoantigens in EL4 cell, indicating the higher impact of immunogenic tumor antigen in antitumor effects during anti-PD-L1 therapy and chemotherapy. We are further exploring the contribution of subdominant epitopes of OVA or antigens other than OVA to antitumor effect.

#### P473 Enhancing abscopal responses to radiation therapy by manipulating autophagy

##### Takahiro Yamazaki, PhD, Marissa Rybstein, Aitziber Buqué, PhD, Ai Sato, Lorenzo Galluzzi, PhD

###### Weill Cornell Medicine, New York, NY, USA

####### **Correspondence:** Takahiro Yamazaki (tay2007@med.cornell.edu)

**Background**

Macroautophagy (autophagy) is an evolutionary conserved cellular mechanism culminating with the lysosomal degradation of dispensable, damaged or potentially toxic cytoplasmic structures (e.g., permeabilized mitochondria). Autophagy helps cancer cells to adapt to harsh environmental conditions and to resist therapy. However, autophagy is also key for multiple steps of the anticancer immune response. Thus, whether autophagy should be inhibited or activated in the context of cancer therapy remains debated [1]. Since autophagy has been shown to play a key role in the removal of cytosolic DNA, which is one mechanism leading to type I interferon (IFN) secretion, and since type I IFN is required for systemic immune responses activated by radiation therapy (RT), we asked the question as to whether selectively inhibiting autophagy in cancer cells may boost the ability of RT to initiate anticancer immunity.

**Methods**

CRISPR/Cas9 technology was used to render mouse mammary carcinoma TSA and EO771 cells autophagy deficient, and chemical inhibitors of autophagy were employed. Autophagy-competent versus –deficient cells were characterized for autophagic proficiency (by immunoblotting), growth (in vitro and in vivo), resistance to cell death induced by starvation, chemotherapy and RT (by multicolor flow cytometry and clonogenic assays) and production of type I IFN (by PCR and ELISA). Alongside, cancer cells were employed to generate synchronous tumors in immunocompetent syngeneic mice. Only one of these tumors (that was either autophagy-competent or-deficient) was irradiated in the context of CTLA4 inhibition, and the response of both the irradiated and non-irradiated (abscopal) tumor was monitored.

**Results**

Autophagy inhibition reduced the growth of mouse mammary carcinoma cells, in vitro and in vivo, limited their clonogenic potential (at baseline) and increased their sensitivity to multiple stressors. Moreover, pharmacological and genetic autophagy inhibition increased the capacity of mouse mammary carcinoma cells to secrete type I IFN in response to radiation. Finally, immunocompetent mice bearing syngeneic autophagy-deficient mouse mammary carcinoma cells mounted improved abscopal responses to RT (in the context of CTLA4 blockade) as compared to immunocompetent mice bearing syngeneic autophagy-competent cells, as determined by growth inhibition of a distant, non-irradiated, autophagy-competent lesion.

**Conclusions**

In conclusion, autophagy inhibits abscopal responses by limiting the release of type I IFN by irradiated cancer cells. We will test the innovative hypothesis that selective autophagy inhibition in cancer cells may synergize with autophagy activation at the whole-body level (by nutrient restriction or physical exercise), hence enabling superior therapeutic responses to radiation.

**References**

1. Rybstein MD, Bravo-San Pedro JM, Kroemer G, Galluzzi L. The autophagic network and cancer. Nat Cell Biol. 2018;20(3):243-251.

**Ethics Approval**

The study was approved by Weill Cornell Medicine‘s Ethics Board, approval number 2017-0007.

#### P474 Antibody targeting of WNT signaling modulator dickkopf1 (DKK1) enhances innate anti-tumor immunity and complements anti-PD-1 therapy

##### Mike Haas, Heidi Heather, Franziska Schürpf-Huber, Lane Newman, Walter Newman, PhD, Mike Kagey, Min Yang, PhD

###### Leap Therapeutics, Cambridge, MA, USA

####### **Correspondence:** Min Yang (myang@leaptx.com)

**Background**

DKK1, a secreted modulator of the Wnt and PI3K/AKT signaling pathways, may contribute to an immunosuppressive tumor microenvironment by influencing Tregs, MDSCs and NK cell functions [1] while also affecting tumor cell NK target expression [2]. DKK1 is expressed in a variety of tumor types and elevated levels frequently correlate with poor survival. DKN-01 is a neutralizing IgG4 monoclonal against DKK1, and mDKN-01 is a murine version of DKN-01.

**Methods**

mDKN-01 has been evaluated in B16 and 4T1 syngeneic mouse tumor models as monotherapy and in combination with anti-PD-1

**Results**

As a monotherapy mDKN-01 demonstrated a highly reproducible inhibition of tumor growth in a B16 melanoma syngeneic model, with concomitant infiltrates of CD45+ CD11b immune cells. Intratumoral MDSC are reduced and show elevated expression of PD-L1. mDKN-01 anti-tumor activity is lost in immunocompromised NSG mice and following depletion of NK cells in immune competent mice. In addition, mDKN-01 anti-tumor activity is retained in a RAG1-/- mouse, highlighting the T cell independence and NK dependence of the anti-tumor mechanism. Combination therapy with anti-PD-1 and mDKN-01 showed cooperative tumor growth inhibition in the B16 model, and reduction of lung metastasis in a 4T1 breast tumor model. These data indicate the potential for DKN-01 complementarity with checkpoint blockers.

**Conclusions**

In murine studies mDKN-01 is an innate-immune IO agent with effects on NK and MDSC cells. mDKN-01 plus anti-PD-1 are more active together than either agent alone in two murine syngeneic models. Preliminary clinical results demonstrate that DKN-01 in combination with pembrolizumab is well tolerated and is clinically active in esophagogastric cancer, including in patients previously treated with other checkpoint inhibitors or in immune resistant phenotypes not expected to respond to pembrolizumab alone. Clinical trial information: NCT02013154

**Trial Registration**

Clinical trial information: NCT02013154

**References**

1. Chae WJ, Ehrlich AK et al. The wnt antagonist dickkopf-1 promotes pathological type 2 cell-mediated inflammation immunity. 2016; 44:246-258.

2. Chae WJ, Park JH, et al. Membrane-bound dickkopf-1 in foxp3+ regulatory T cells suppresses T-cell-mediated autoimmune colitis. Immunology. 2017;265-275.

3. D'Amico L, Mahajan S. Dickkopf-related protein 1 (Dkk1) regulates the accumulation and function of myeloid derived suppressor cells in cancer. J Exp Med. 2016; 213:827-40.

4. Malladi S, Macalinao DG, et al. Metastatic latency and immune evasion through autocrine inhibition of WNT Cell. 2016;165:45-60.

#### P475 Avadomide in combination with nivolumab results in increased activated and memory T-cells and enhances CD8+ tumor infiltration in hepatocellular carcinoma patients

##### Patrick Hagner, PhD^1^, Fadi Towfic, PhD^1^, Alfredo Romano, MD^1^, Julien Edeline, MD^2^, Carlos Gomez-Martin, MD, PhD^3^, Antoine Hollebecque, MD^4^, Robin Kate Kelley, MD^5^, Armando Santoro, MD, PhD^6^, Michael Pourdehnad, MD^1^, Anita Gandhi, PhD^1^

###### ^1^Celgene Corporation, Summit, NJ; ^2^Centre Eugene Marquis, Rennes, France; ^3^Hospital 12 de Octubre, Madrid, Spain; ^4^Institut G. Roussy, Villejuif, France; ^5^University of California San Francisco, San Francisco, CA, USA; ^6^Humanitas University, Rozzano-Milan, Italy

####### **Correspondence:** Patrick Hagner (phagner@celgene.com)

**Background**

Avadomide (CC-122) binds E3 ubiquitin ligase CRL4^CRBN^, resulting in degradation of transcription factors Aiolos and Ikaros, and activation of T cells. Preclinical and clinical data indicate that avadomide exerts strong immunomodulatory activity through enhanced ADCC and a shift in T-cell subsets from naïve to effector and memory subsets. Avadomide is in clinical development in multiple hematologic diseases and has been explored in solid tumors, including hepatocellular carcinoma (HCC), as single agent (NCT01421524) and in combination with nivolumab (NCT02859324). In preclinical models, avadomide plus nivolumab demonstrates synergistic activation of T cells and significantly enhanced immune-mediated cytotoxicity against HCC cells. Here, we report the effects of combining avadomide with nivolumab on peripheral blood T-cell subsets and activation status and on trafficking of immune cells to the tumor in patients with HCC.

**Methods**

Peripheral blood T-cell subsets were analyzed by flow cytometry. Tumor biopsies were analyzed by immunohistochemistry or RNA sequencing with deconvolution analyses to identify immune cell populations.

**Results**

Avadomide, as single agent and in combination with nivolumab, results in decreased absolute peripheral CD4+ and CD8+ naïve (CD45RA+/CD45RO–) T cells and increased memory (CD45RA–/CD45RO+) and activated (HLA- DR+) T cells, without significantly affecting total CD3+, CD4+ or CD8+ populations. Interestingly, the combination demonstrated a trend towards greater increase in activated (+182%) and memory (+257.9%) CD4+ T cells compared with avadomide alone (+123.2% and +12.2%). Increased levels of peripheral Treg populations were detected within 15 days of treatment initiation, and the CD8/Treg ratio declined from 7.8 at screening to 2.7 on C1D15. To understand the effects on tumor microenvironment, we performed RNA sequencing on paired tumor biopsies from patients receiving combination treatment collected at enrollment and six weeks after treatment initiation (n=9). Deconvolution analyses identified increased infiltration of T-cell populations, dendritic cells and macrophages, and decreased B-cell populations in on-treatment biopsies relative to pre-treatment. Immunohistochemistry confirmed significantly increased CD8+ T-cells in on-treatment biopsies relative to pre-treatment in patients receiving the combination (*P*=0.04), while no significant changes in CD8+ T-cell infiltration were observed in patients receiving single agent avadomide (*P*=0.65).

**Conclusions**

Avadomide is a potent immunomodulating agent with multiple immune activating properties. Avadomide plus nivolumab leads to significantly greater CD8+ T-cell tumor infiltration compared with single agent avadomide. These findings provide proof-of-concept for the combination of avadomide with checkpoint blockade in solid tumors and demonstrate potential for further clinical and biomarker studies to ascertain relative contribution of avadomide over nivolumab monotherapy and assess efficacy.

**Trial Registration**

ClinicalTrials.gov identifier NCT01421524 and NCT02859324.

**Ethics Approval**

This study was approved by the research ethics boards of all participating institutions.

**Consent**

Written informed consent was obtained from the patient for publication of this abstract and any accompanying images. A copy of the written consent is available for review by the Editor of this journal.

### Immunosuppressive Cells in the Tumor Microenvironment

#### P476 EWS-FLI1 expression level modulates T-cell mediated tumor apoptosis in Ewing sarcoma

##### Claire Julian, Ariel Klinghoffer, Hether Bernard, MD, Linda McAllister-Lucas, Kelly Bailey, MD, PhD

###### University of Pittsburgh, Pittsburgh, PA, USA

####### **Correspondence:** Kelly Bailey (kelly.bailey@chp.edu)

**Background**

Metastatic Ewing sarcoma is a deadly bone cancer most commonly diagnosed in children and is driven by the fusion oncoprotein EWS-FLI1. The level of EWS-FLI1 expression can change Ewing cell behavior. Interestingly, lower levels of EWS-FLI1 are associated with increased expression of ICAM-1, a surface protein reported in some cancers to influence tumor cell: T-cell interaction and promote T-cell activation. Very little is known about the immune response to Ewing sarcoma tumor cells and elucidating the mechanisms regulating the immune response to Ewing tumor cells may reveal much needed new treatment opportunities for patients with metastatic disease. In this study, we seek to determine the impact of tumor cell EWS-FLI1 expression level on T-cell mediated Ewing sarcoma tumor cell apoptosis.

**Methods**

We performed real-time monitoring of tumor cell caspase 3 activity in Ewing tumor cell/T-cell co-cultures. For this analysis, Ewing sarcoma tumor cell populations with ‘high’ or ‘low’ EWS-FLI1 expression were prepared by either: 1) using flow cytometry to isolate naturally occurring populations or 2) using EWS-FLI1 siRNA to generate EWS- FLI1 ‘low’ cells. Human T-cells were isolated from random donor buffy coat, and T-cells were activated using a CD2/3/28 antibody cocktail. Surface expression of ICAM-1, PD-L1 and PD-L2 was determined by flow cytometry analysis. Blocking antibodies were also utilized.

**Results**

EWS-FLI1 ‘low’ cells demonstrated a significant decrease in T-cell mediated tumor cell apoptosis upon introduction of ICAM-1 blocking antibody. Based on this, we questioned whether EWS-FLI1 ‘low’ cells would be more susceptible to T-cell mediated apoptosis that EWS-FLI1 ‘high’ cells (that lack surface ICAM-1). Notably, despite having higher ICAM-1, we found that EWS-FLI1 ‘low’ cells are actually less susceptible to T-cell mediated apoptosis that EWS-FLI1 ‘high’ cells, suggesting that EWS-FLI1 ‘low’ cells possess an ability to evade T-cell- mediated tumor killing. Further, in comparison to EWS-FLI1 ‘high’ cells, EWS-FLI1 ‘low’ cells respond to interferon-gamma treatment with dramatically greater transcriptional upregulation of PD-L1 and PD-L2. We then assessed the impact of PD-1 blocking antibody on T-cell mediated tumor cell apoptosis and found that treatment of EWS-FLI1 ‘low’ cell/T-cell co-cultures with blocking antibody significantly enhances T-cell-induced tumor cell apoptosis.

**Conclusions**

We have shown that Ewing cells with lower EWS-FLI1 are more resistant to T-cell mediated apoptosis than cells with higher EWS-FLI1. As such, EWS-FLI1 ‘low’ cells may serve as negative regulators of the immune response in Ewing tumors. These data highlight that Ewing tumor cell heterogeneity can influence the anti-tumor immune response.

**Acknowledgements**

KMB is supported by Alex’s Lemonade Stand Foundation Young Investigator Award, The Children’s Cancer Research Fund Emerging Scientist Award and the NIH 2K12HD052892-11A1

#### P477 HMBD-002-V4: A novel anti-VISTA antibody that uniquely binds murine and human VISTA and potently inhibits tumor growth by remodeling the immunosuppressive tumor microenvironment

##### Jerome Boyd-Kirkup, PhD, Dipti Thakkar, PhD, Vicente Sancenon, PhD, Siyu Guan, PhD, Konrad Paszkiewicz, PhD, Piers Ingram, PhD

###### Hummingbird Bioscience, South San Francisco, CA, USA

####### **Correspondence:** Piers Ingram (p.ingram@hummingbirdbio.com)

**Background**

Immune checkpoint therapies have shown unprecedented clinical activity in several types of cancer, however, less than 30% of patients respond. VISTA is a co-inhibitory immune checkpoint receptor of the B7 family and functions to suppress human T-cell activity. VISTA is highly expressed on tumor infiltrating myeloid cells including myeloid derived suppressive cells (MDSC), which have been associated with resistance to immunotherapy. Increases in VISTA+ cells have also been observed in response to PD1 and CTLA4 therapy. Targeting VISTA could represent a novel treatment axis in the non-responder population.Despite the promise of VISTA, limited structural information, lack of a definitive ligand, and incomplete data on expression in normal vs. disease contexts, have made development of drug candidates challenging. Further, previous anti-VISTA antibodies have only bound to rodent *or* human VISTA, making it impossible to translate pre-clinical efficacy and safety data to predict patient response.

**Methods**

HMBD-002-V4 is a humanized anti-VISTA antibody developed using Hummingbird Bioscience’s proprietary Rational Antibody Discovery platform to target a specific epitope predicted by structural modeling to block ligand binding and be conserved between human, cyno and murine VISTA.

**Results**

*In vitro*, HMBD-002-V4 showed dose-dependent inhibition of the interaction between VISTA and the putative ligand VSIG3 for both human and mouse orthologs, and further demonstrated release of VISTA inhibition on T-cell activity and increased secretion of pro-inflammatory cytokines in human *ex vivo* assays.*In vivo*, HMBD-002-V4 showed single agent tumor growth inhibition (TGI) of up to 40% in syngeneic murine CDX models, however, efficacy was significantly improved if combined with anti-PD(L)1 antibody where TGI above 94% was possible. Profiling of representative tumors by FACS revealed MDSC infiltration in these models that was significantly increased after treatment with anti-PD(L)1 antibody and associated with an increase in immunosuppressive serum cytokines. Conversely, HMBD-002-V4 efficacy was associated with decreased MDSC infiltration for both monotherapy and combination arms and a remodeling of the tumor microenvironment towards a pro-inflammatory phenotype. In models without MDSC infiltration, HMBD-002-V4 showed poor efficacy. HMBD-002-V4 was evaluated for pharmacokinetics and toxicology and demonstrated excellent serum half-life of 11 days, with no observable toxicity in multiple animal models. Further, HMBD-002-V4 has been optimized for manufacturability, including high expression titers and stability.

**Conclusions**

HMBD-002-V4 represents a promising therapeutic candidate for the treatment of VISTA-mediated suppression of anti-tumor immunity. Predictive biomarkers of response to HMBD-002-V4 are currently being explored in multiple indications and the first-in-human trial of HMBD-002-V4 is planned for 2019.

**Ethics Approval**

The study was approved by the SingHealth Institutional Animal Care and Use Committee, approval number 2016/SHS/1230.

#### P478 Platelets as immune suppressors in anti-cancer immune responses

##### Ana Micaela Carnaz Simões, Morten Holström, PhD, Mads Andersen, PhD, Per Thor Straten, PhD

###### Center for Cancer Immune Therapy, Herlev, Herlev, Denmark

####### **Correspondence:** Ana Micaela Carnaz Simões (ana.micaela.carnaz.simoes@regionh.dk)

**Background**

Platelets (PLTs) are well-known players during cancer progression. For several cancers, an increased number of circulating PLTs correlates with poor prognosis. PLTs help cancer cells by modulating angiogenesis and/or directly binding cancer cells, which facilitates the metastatic process [1,2]. These cells and their soluble factors can also protect cancer cells from immune attack by mechanisms that are poorly understood. Studies focused on autoimmune conditions, have shown that exhausted PLTs form aggregates with T cells, downregulating T cell activation, proliferation and interferon-ɣ production [3,4]. Nevertheless, no similar study has been conducted in the context of cancer.

**Methods**

Our study investigated the presence of circulating PLT-immune cell aggregates in myeloproliferative neoplasm (MPN) patients. To that purpose, cryopreserved peripheral blood mononuclear cells were analyzed by multicolor flow cytometry for PLT bound -T, -NK, -B and -CD3+/CD56+ cells, as well as CD4 and CD8 T cell subpopulations. Furthermore, to assess the effect PLT-binding has on T and NK cell anti-tumor reactivity, in vitro cytotoxic response was continuously monitored over 40 hours, using the xCELLigence technology.

**Results**

Our preliminary results show that, when compared to healthy donors, MPN patients have an increased number of PLT bound CD8+ T, NK and CD3+/CD56+ cells. Finally, our results indicate that platelets can modulate the T and NK cells tumor reactivity in distinct manners; the presence of PLTs impairs the killing capacity of T cell whereas it seems to enhance it on NK cells. However, further studies are needed to confirm our preliminary results.

**Conclusions**

N/A

**References**

1. Borsig L. The role of platelet activation in tumor metastasis. Expert Rev Anticancer Ther. 2008;8(8):1247-1255. doi:10.1586/14737140.8.8.1247.

2. Bambace NM, Holmes CE. The platelet contribution to cancer progression. J Thromb Haemost. 2011;9(2):237- 249. doi:10.1111/j.1538-7836.2010.04131.x.

3. Zamora C, Canto E, Nieto JC, et al. Functional consequences of platelet binding to T lymphocytes in inflammation. J Leukoc Biol. 2013;94(3):521-529. doi:10.1189/jlb.0213074.

4. Zamora C, Cantó E, Nieto JC, et al. Binding of platelets to lymphocytes: A potential anti-inflammatory therapy in rheumatoid arthritis. J Immunol. 2017;198(8):3099-3108. doi:10.4049/jimmunol.1601708.

#### P479 NG-641: an oncolytic T-SIGn virus targeting cancer-associated fibroblasts in the stromal microenvironment of human carcinomas

##### Matthieu Besneux^1^, Brian Champion, PhD^1^, Nalini Marino^1^, Marilena Patsalidou^1^, Gianfranco di Genova^1^, Sam Illingworth^1^, Stefania Fedele^1^, Lorna Slater^1^, Darren Plumb^1^, Katy West^1^, Joshua Freedman, BS^2^, Len Seymour^2^, Kerry Fisher, MD PhD^1^, Alice Brown, PhD^1^

###### ^1^PsiOxus Therapeutics Ltd, Abingdon, UK; ^2^Oxford University, Oxford, UK

####### **Correspondence:** Brian Champion (brian.champion@psioxus.com)

**Background**

NG-641 is a modified variant of enadenotucirev (EnAd), an Ad11p/Ad3 chimeric group B adenovirus, which retains all the functional properties of enadenotucirev, while also mediating the expression of transgenes designed to target the breakdown of the stromal barrier and reverse immune suppression within the tumor microenvironment (TME). As an approach to immunogene therapy targeting stromal rich tumors, we have created a transgene-modified variant of EnAd expressing a bi-specific T-cell activator molecule (FAP-TAC) recognizing human fibroblast activating protein (FAP) on cancer associated fibroblasts (CAFs) and CD3 on T-cells. To enhance the activity of the bispecific molecule, particularly in tumors with poor immune cell infiltration (“excluded phenotype”), NG-641 also encodes an immune-enhancer module (IM), consisting of the chemokines CXCL9 and CXCL10 and the type I interferon IFNa..

**Methods**

FAP-TAC constructs comprising linked ScFv antibodies specific for human FAP and CD3 were designed and used to generate EnAd viruses expressing the FAP-TAC transgene such that expression was under the virus major late promoter (MLP) to allow broad or tumor-selective expression, respectively. Tumor and fibroblast cell lines and freshly isolated malignant peritoneal ascites and surgically excised tumor samples from carcinoma patients were used to evaluate virus activities.

**Results**

Initial studies with different viruses expressing FAP-TAC alone or with different additional transgenes showed that FAP-TAC activity generated by NG-641 infection was essentially the same as that with viruses bearing only the FAP-TAC transgene. In tumor cell lines, NG-641 selectively secretes functional FAP-TAC molecules, as determined in cocultures of FAP+ fibroblasts and PBMC-derived T cells. Production of the IM transgenes CXCL9, CXCL10 and IFNa was confirmed by specific ELISA assays, with functionality evaluated by reporter cell, FACS and cell migration assays. T cell-activation mediated by FAP-TAC leads to cytokine secretion and cytotoxicity towards the fibroblasts. Studies with unseparated primary tumor samples (containing tumor cells, CAFs and infiltrated immune cells) also demonstrated potent activation of the endogenous T-cells, indicating that virus produced FAP-TAC is a potent T-cell activator despite suppressive influences of the TME.

**Conclusions**

In this study, we have shown that CAFs can be effectively targeted for T-cell mediated destruction by NG-641, a tumor stroma targeting transgene-bearing oncolytic virus. This is associated with strong activation of endogenous T- cells to kill CAFs even in the presence of an immunosuppressive microenvironment. Systemic dosing of such a virus to patients with stromal rich tumors may provide an effective approach for reversing immune suppression within the TME and driving effective anti-tumor immunity.

#### P480 Transcriptomic characterization of tumor vs. peripheral blood NK cells in head and neck cancer patients

##### Fernando Concha-Benavente, MD, PhD^2^, Robert Ferris^1^

###### ^1^University of Pittsburgh, Pittsburgh, PA, USA; ^2^UPMC Hillman Cancer Center, Pittsburgh, PA, USA

####### **Correspondence:** Robert Ferris (ferrrl@upmc.edu)

**Background**

NK cells play a crucial role in tumor immunosurveillance with a unique capacity of killing cancer cells via antibody dependent cellular cytotoxicity (ADCC), particularly in the setting of head and neck cancer (HNC) where cetuximab, an EGFR-specific mAb, has been used since 2006. Cetuximab-activated NK cells induce dendritic cell (DC) activation, tumor antigen cross-presentation and expansion of EGFR-specific T cells, linking innate and adaptive antitumor immunity. However, the benefit of cetuximab is only seen in 10-15% of patients [1]. Moreover, NK cell dysfunction has been associated with increased risk of cancer [2-5] and poor clinical prognosis [6-8]. Therefore, characterizing whether NK cells infiltrate HNC tumors and their expression profile is important in order to reverse NK cell dysfunction and improve cancer immunotherapy

**Methods**

NK cells were sorted by flow cytometry from freshly isolated HNC peripheral blood lymphocytes (PBL) and paired tumor infiltrating lymphocytes (TIL). Sequential RT reactions produced amplified dsDNA which was fragmented, end labeled with biotin and hybridized to the Human Clariom S array. First level data analysis was performed using Affymetrix Expression Console using RMA normalization algorithm

**Results**

Initial exploratory grouping analysis (EGA) showed that PBL (n=9) and TIL (n=6) NK cells had a divergent expression profile. Hierarchical clustering (HC) analysis of paired PBL and TIL NK cells (n=4) revealed that TIL NK cells had a total of 1345 differentially expressed genes, from which 713 were downregulated more than 2-fold when compared to PBL NK cells. We found that Th1 type transcription factors such as EOMES and BLIMP-1, activation/cytolytic markers such as CD38, NKG2F, CD247, granzyme A, granzyme H, perforin 1 and FCGR3A (CD16a) and FCGR3B (CD16b) which are key molecules for ADCC were significantly downregulated in TIL NK cells. Likewise, sphingosine-1-phosphate receptor 1 (S1PR1), a critical mediator of NK cell traffic and retention in inflamed tissues, Th1 type induced T/NK cell attracting chemokines CCL5 and CCL4 and innate immunity activator, toll-like receptor 3 (TLR3) were downregulated in TIL NK cells.

**Conclusions**

These findings suggest an exhausted phenotype of TIL NK cells characterized by downregulation of Th1 type activation markers compared to NK cells in the periphery. Reversing NK cell dysfunction is key in order to improve antitumor immunity of HNC patients.

**References**

1. Ferris R L, Jaffee E M, Ferrone S. Tumor antigen-targeted, monoclonal antibody-based immunotherapy: clinical response, cellular immunity, and immunoescape. J Clin Oncol 2010. 28: 4390-4399.

2. Hersey P, Edwards A, Honeyman M, McCarthy W H. Low natural-killer-cell activity in familial melanoma patients and their relatives. Br J Cancer 1979. 40: 113-122.

3. Fauriat C, Just-Landi S, Mallet F, Arnoulet C, Sainty D, Olive D, Costello R T. Deficient expression of NCR in NK cells from acute myeloid leukemia: Evolution during leukemia treatment and impact of leukemia cells in NCRdull phenotype induction. Blood 2007. 109: 323-330.

4. Saito H, Osaki T, Ikeguchi M. Decreased NKG2D expression on NK cells correlates with impaired NK cell function in patients with gastric cancer. Gastric Cancer 2012. 15: 27-33.

5. Schantz S P, Shillitoe E J, Brown B, Campbell B. Natural killer cell activity and head and neck cancer: a clinical assessment. J Natl Cancer Inst 1986. 77: 869-875.

6. Coca S, Perez-Piqueras J, Martinez D, Colmenarejo A, Saez M A, Vallejo C, Martos J A, Moreno M. The prognostic significance of intratumoral natural killer cells in patients with colorectal carcinoma. Cancer 1997. 79: 2320-2328.

7. Takeuchi H, Maehara Y, Tokunaga E, Koga T, Kakeji Y, Sugimachi K. Prognostic significance of natural killer cell activity in patients with gastric carcinoma: a multivariate analysis. Am J Gastroenterol 2001. 96: 574-578.

8. Ishigami S, Natsugoe S, Tokuda K, Nakajo A, Che X, Iwashige H, Aridome K, Hokita S, Aikou T. Prognostic value of intratumoral natural killer cells in gastric carcinoma. Cancer 2000. 88: 577-583.

#### P481 The epigenetic underpinnings of regulatory T cell fragility in the tumor microenvironment

##### Becky Dadey, BS^1^, Abigail Overacre-Delgoffe, PhD^2^, Ting Wang^1^, Zhe Sun^1^, Rong Zhang, MS^2^, Wei Chen^2^, Natalie Rittenhouse^2^, Amanda Poholek^2^, Tracy Tabib^1^, Robert Lafyatis^1^, Creg Workman, PhD^1^, Dario Vignali, PhD^1^

###### ^1^University of Pittsburgh, Pittsburgh, PA, USA; ^2^Children's Hopsital of Pittsburgh, Pittsburgh, PA, USA

####### **Correspondence:** Dario Vignali (dvignali@pitt.edu)

**Background**

Regulatory T cells (Tregs) are a suppressive cell population that limit the anti-tumor response. However, systemic ablation of Tregs cannot be utilized as a therapy due to massive autoimmune defects. Our lab has demonstrated that Treg-restricted deletion of cell surface protein Neuropilin (Nrp1, CD304) results in substantially reduced tumor growth with no autoimmune defects [1]. We have shown that Treg-restricted deletion of Nrp1 in the TME does not result in loss of Foxp3 expression and “ex-Treg” generation but rather causes them to exhibit an effector-like phenotype including loss of suppressive function and production of interferon gamma (IFNү), which we refer to as Treg fragility [2].

**Methods**

We sought to understand the epigenetic underpinnings between Nrp1-sufficient and -deficient Tregs from the tumor microenvironment that could lead to this ‘fragile’ state. To do so we performed bisulfite treatment from ZymoEZ Direct Kit followed by Sanger Sequencing to identify differences in DNA methylation. We utilized ATAC sequencing to identify discrepancies in chromatin accessibility following the Greenleaf protocol [3]. We also utilized TCR sequencing from Adaptive Biotechnologies per the manufacturer’s protocol. For single cell RNAseq, we loaded 3500 cells/sample using ChromiumTM Single Cell 3' Gel Bead Kit and Chromium Single Cell 3'v2 Library Kit. Samples were sequenced on a NextSeq500. Finally, CUT&RUN ChIP-seq was perform following the Henikoff protocol [4].

**Results**

We found that Tregs lacking Nrp1 in the TME have a differential methylation signature at the Conserved Non-coding Sequence 2 (CNS2) locus of the Foxp3 gene, albeit no difference in the chromatin accessibility at this locus, no change in single cell RNAseq, and maintenance of Foxp3 protein expression. We also found that Nrp1-deficient Tregs are not peripherally-induced Tregs but rather are thymically-derived.

**Conclusions**

We have identified an intriguing change in the DNA methylation status of the CNS2 locus of Foxp3 in the Nrp1-deficient Tregs from the tumor microenvironment but no loss in Foxp3 expression. This finding conflicts with current data suggesting that CNS2 hypermethylation shuts off Foxp3 expression. Additional experiments will be required to understand how this locus maintains Foxp3 protein despite DNA methylation. Future studies will also examine the epigenetic mediators that might cause this differential methylation or if extrinsic factors in the TME promote differential methylation.

**References**

1. Delgoffe GM, Woo SR, Tunis ME, Gravano DM, Guy C, Overacre AE, Bettini ML, Vogel P, Finkelstein D, Bonnevier J, Workman CJ, Vignali DA. Stability and function of regulatory T cells is maintained by a neuropilin-1- semaphorin-4a axis. Nature. 2013; 7466, 252-6

2. Overacre-Delgoffe AE, Chikina M, Dadey RE, Yano H, Brunazzi EA, Shayan G, Horne W, Moskovitz JM, Kolls JK, Sander C, Shuai Y, Normolle DP, Kirkwood JM, Ferris RL, Delgoffe GM, Bruno TC, Workman CJ, Vignali DAA. Interferon-γ derives treg fragility to promote anti-tumor immunity. Cell. 2017; 169, 1130-41

3. Buenrostro JD, Giresi PG, Zaba LC, Chang HY, Greenleaf WJ. Transposition of native chromatin for fast and sensitive epigenomic profiling of open chromatin, DNA-binding proteins and nucleosome position. Nat Methods. 2013; 10:1213-1218

4. Skene PJ, Henikoff S. An efficient targeted nuclease strategy for high-resolution mapping of DNA binding sites. Elife 2017; 6:e21856

#### P482 Humanization and characterization of novel, best in class isoform-specific anti-TGFβ monoclonal antibodies

##### Matteo Brioschi^1^, Pamela Cheou^2^, Jacques Van Snick, PhD^2^, Catherine Uyttenhove, PhD^2^, George Coukos, MD, PhD^3^, Gerd Ritter^4^, Steven Dunn, PhD^3^, Steven Dunn, PhD^3^

###### ^1^Ludwig Institute for Cancer Research, Epalinges, Switzerland., Epalinges, Switzerland; ^2^Ludwig Institute for Cancer Research Ltd, Brussels, Belgium., Brussels, Belgium; ^3^Ludwig Institute for Cancer Research, Epalinges, Switzerland. CHUV, Lausanne, Switzerland., Epalinges, Switzerland; ^4^Ludwig Institute for Cancer Research, New York, NY., New York, NY, USA

####### **Correspondence:** Steven Dunn (steven.dunn@chuv.ch)

**Background**

TGFβ is a conserved, highly pleiotropic and potent cytokine that has been implicated in tumor escape and progression via its modulation and suppression of multiple immune-cell related pathways within the tumor microenvironment [1]. Three homo-dimeric isoforms have been identified, which individually have been shown to drive context-dependent physiological and phenotypic responses, including aspects of proliferation, migration, differentiation, angiogenesis, and immune responsiveness [2]. Although a compelling target for tumor immunotherapy, the use of anti-TGFβ antibodies that do not adequately distinguish between the individual isoforms could give rise to on-target off-tumor toxicity, undesirable inflammatory adverse events or a zero-sum anti-tumor activity. To address this unmet need for more specific reagents, we have selected two antibodies for humanization from a panel of recently generated high-affinity murine mAb hybridomas which have extraordinary mono-isoform selectivity for TGFβ1 or TGFβ3 and which neutralize TGFβ-driven signalling in a cell reporter system with high potency [3]. Isoform specific TGFβ blockade with these antibodies is effective at delaying in-vivo tumor growth in melanoma and breast cancer models [4].

**Methods**

Antibody humanization was performed using a molecular engineering approach combining framework grafting, competitive screening of antibody fragments and selective back mutation of individual residues guided by assaying for TGFβ neutralization potency of the antibody constructs using a transformed mink lung epithelial cell (tMLEC) TGFβ reporter line. Binding kinetics were assayed by surface plasmon resonance. Physicochemical properties of the engineered antibodies and suitability for down-stream development were characterized using several techniques including transient expression in singular cell system, size-exclusion chromatography and thermal differential scanning fluorimetry.

**Results**

We report the successful conversion of two TGFβ isoform-specific mouse mAbs into humanized recombinant IgG4 molecules targeting human TGFβ1 or TGFβ3 respectively. Engineered variants of both parental TGFβ antibodies express efficiently in a transient human cell system, display good thermal stability and retain their potent binding and neutralization activity with regard to their cognate TGFβ isoforms. We were able to generate several humanized LCR1901 anti-TGFβ3 variants with significantly improved neutralization potency in cellular TGFβ signalling assays relative to the parental mAb, and with essentially undetectable levels of binding to TGFβ1 and TGFβ2.

**Conclusions**

We have generated a panel of humanized antibodies that specifically neutralize TGFβ3 or TGFβ1 signalling. Recent studies assigning a dominant gatekeeper role for TGFβ3 in malignant glioblastoma progression [5], as well as specifically implicating TGFβ1 in breast and gastric cancers [6,7], support the continued pre-clinical and clinical investigation of these novel TGFβ antibodies.

**References**

1. Neuzillet C, Tijeras-Raballand A, Cohen R, Cros J, Faivre S, Raymond E, de Gramont A. Targeting the TGFβ pathway for cancer therapy. Pharmacol Ther. 2015 Mar;147:22-31

2. Poniatowski ŁA, Wojdasiewicz P, Gasik R, Szukiewicz D. Transforming growth factor beta family: Insight into the role of growth factors in regulation of fracture healing biology and potential clinical applications. Mediators Inflamm. 2015;2015:137823

3. Uyttenhove C, Marillier RG, Tacchini-Cottier F, Charmoy M, Caspi RR, Damsker JM, Goriely S, Su D, Van Damme J, Struyf S, Opdenakker G, Van Snick J. Amine-reactive OVA multimers for auto-vaccination against cytokines and other mediators: perspectives illustrated for GCP-2 in L. major infection. J Leukoc Biol. 2011 Jun;89(6):1001-7

4. Gupta A, Budhu S, Giese R, van Snick J, Uyttenhove C, Ritter G, Wolchok J, Merghoub T. Isoform specific TGF-β inhibition in combination with radiation therapy as a novel immune therapeutic approach to cancer therapy. Journal for ImmunoTherapy of Cancer 2017; 5 (suppl 2) :87 Abstract 326 AND Gupta A, Budhu S, Giese R, van Snick J, Uyttenhove C, Ritter G, Wolchok J, Merghoub T. Targeting specific TGF-β isoforms in combination with radiation therapy leads to differential antitumor effects in mouse models of cancer. Cancer Res. 2018; 78(13 suppl): Abstract 4716

5. Seystahl K, Papachristodoulou A, Burghardt I,Schneider H, Hasenbach K, Janicot M, Roth P, Weller M. Biological Role and Therapeutic Targeting of TGF-β3 in Glioblastoma. Mol Cancer Ther. 2017 Jun;16(6):1177- 1186

6. Rodriguez G, Abrahamsson A, Jensen L, Dabrosin C. Estradiol Promotes Breast Cancer Cell Migration via Recruitment and Activation of Neutrophils. Cancer Immunol Res; 5(3); 234–47

7. Peng LS, Zhang JY, Teng YS, Zhao YL, Wang TT, Mao FY, Lv YP, Cheng P, Li WH, Chen N, Duan M, Chen W, Guo G, Zou QM, Zhuang Y. Tumor-Associated Monocytes/Macrophages Impair NK-Cell Function via TGFb1 in Human Gastric Cancer. Cancer Immunol Res. 2017 Mar;5(3):248-256

#### P483 Iron and HLA-G: Neglected immunosuppressive molecules in the human microenvironment

##### Xian Jiang^2^, Robert Elliott^1^

###### ^1^Mastology, Baton Rouge, LA, USA; ^2^the Breast Foundation, Baton Rouge, LA, USA; ^3^EEB clinic, Baton Rouge, LA, USA

####### **Correspondence:** Robert Elliott (relliot@eehbreastca.com)

**Background**

There are many immunosuppressive molecules in the tumor microenvironment that need to be inhibited, if we are to improve immunotherapy in the Stage IV patients. There are many reports on the possible role of HLA-G in cancer immunosuppression, but very little on the role of iron. The immunosuppressive effect of iron and HLA-G in the tumor microenvironment has been neglected and ignored as therapeutic targets. This study was implemented to determine if iron and HLA-G, as neglected molecules enhance tumor immunosuppression, and could be cancer immunotherapy targets.

**Methods**

We examined HLA-G expression in normal mammary and breast cancer cell lines and human normal and breast cancer tissue. This examination was done by reverse transcription polymerase chain reaction (RT-PCR) and immunohistochemistry (IHC). Intracellular iron levels were manipulated in the human MCF-7 and MDA-MB-231 breast cancer cell lines. Cytolysis of these cell lines was measured after exposure to the natural killer cell line NK-92 MI (NK). The gene expression of ferritin heavy chain (FTH1) was determined as was the production of nitric oxide (NO) and tumor necrosis factor alpha (TNFa).

**Results**

RT-PCR confirmed HLA-G expression was absent in the normal epithelial MCF-12A cells showing no mRNA expression, however, the cell lines MCF-7, MDA-MB-231and T-47D had various levels of HLA-G mRNA expression. IHC was performed on 38 breast cancer specimens and on 12 normal breast specimens. Fifty-eight percent (22/38) of the cancer had medium to strong staining, but only 8.3% (1/12) of the normal specimens had medium staining. The difference was significant (p<0.05). When NK-92 MI cells were co-cultured with MCF-7 and MDA-MB-231 cells, NO and TNF-a were released into the media. The addition of iron inhibited the cytolysis of cancer cell lines. Deferoxamine (DFOM), an iron chelator, increased NK-92 MI cytolysis of MCF-7 and MDA-MB- 231 cells. The cytotoxicity of the breast cancer cells was reversed by the addition of iron. This cytotoxicity is induced by NO released from S-nitro-N-acetyl – penicillamine (NO donor). RT-PCR showed the iron chelator reduced FTH1 expression, while iron upregulated the expression of FTH1.

**Conclusions**

HLA-G antigen is expressed in trophoblastic placental cells as an immunotolerant molecule to protect the fetus from maternal alloreactivity. Its expression in cancer cells contributes to cancer immunosuppression. Increased iron in the tumor microenvironment and cancer cells inhibited cancer cells cytolysis by NK cells by antagonizing NO and TNFa cytotoxicity and the upregulation of ferritin expression. We hope this study will stimulate researchers to investigate the role of HLA-G and iron as therapeutic targets of the cancer microenvironment. Cancer immunotherapy in Stage IV patients will be improved by the inhibition of these neglected molecules.

#### P484 Pharmacokinetics, pharmacodynamics, and safety of FLX475, an orally-available, potent, and selective small- molecule antagonist of CCR4, in healthy volunteers

##### Sjoerd van Marle, MD^2^, Ewoud-Jan van Hoogdalem, PhD, RPh^2^, Daniel Johnson^1^, Abood Okal, PhD^1^, Paul Kassner, PhD^1^, David Wustrow, PhD^1^, William Ho, MD, PhD^1^, Steven Smith^1^

###### ^1^FLX Bio, South San Francisco, CA, USA; ^2^PRA Health Sciences, Groningen, Netherlands

####### **Correspondence:** William Ho (bill.ho@flxbio.com)

**Background**

Regulatory T cells (Treg) are essential for immune tolerance to self antigens, but can also dampen anti-tumor immune responses in the tumor microenvironment (TME). The predominant chemokine receptor on human Treg is CCR4, the receptor for the chemokines CCL17 and CCL22 [1], which are produced by tumor cells, tumor- associated macrophages and dendritic cells, as well as by effector T cells (Teff) in the setting of an inflammatory anti-tumor response. Preclinical studies with orally-available CCR4 antagonists have demonstrated potent inhibition of Treg migration into tumors, an increase in the intratumoral Teff/Treg ratio, and anti-tumor efficacy as a single agent and in combination with checkpoint inhibitors. [2]

**Methods**

A first-in-human, randomized, double-blind, placebo-controlled trial was conducted to examine the safety, pharmacokinetics (PK), and pharmacodynamics (PD) in healthy volunteers (HVs) of single and repeat dosing of FLX475, an orally-available, potent, and selective small-molecule antagonist of CCR4. Seven cohorts of 8 subjects each (6 drug, 2 placebo) were administered single doses ranging from 5 mg to 1000 mg. Six cohorts were administered daily doses of FLX475 for 14 days ranging from 25 mg to 150 mg, including two cohorts evaluating a loading dose administered on Day 1.

**Results**

FLX475 was well-tolerated, with no significant laboratory abnormalities or dose-limiting clinical adverse events. Dose-dependent increases in exposure were observed with low peak-to-trough ratios and a half-life of approximately 72 hours. Daily dosing without a loading dose demonstrated approximately 4-5x accumulation of FLX475 over 14 days. A receptor occupancy (RO) PD assay using study subject peripheral blood Treg [3] demonstrated a tight PK/PD relationship, suggesting that doses of approximately 75 mg PO QD and above are sufficient to maintain target drug exposure above the IC90 for human in vitro Treg migration.

**Conclusions**

In this first-in-human HV study, the oral CCR4 antagonist FLX475 was demonstrated to be well tolerated with outstanding PK properties. A robust PD assay measuring receptor occupancy on circulating Treg demonstrated the ability to safely achieve exposure levels predicted to maximally inhibit Treg recruitment into tumors via CCR4 signaling. These data have enabled the optimized design of an ongoing Phase 1/2 study of FLX475 both as monotherapy and in combination with checkpoint inhibitor in cancer patients.

**Trial Registration**

EudraCT 2017-003952-22

**References**

1. Talay O et al. Potent and selective C-C chemokine receptor (CCR4) antagonists potentiate anti-tumor immune responses by inhibiting regulatory T cells (Treg) [abstract]. In: Proceedings of the 110th Annual Meeting of the American Association for Cancer Research; 2017 Apr 1–5; Washington, DC. AACR; 2017. Abstract nr 4600.
2. Talay O et al. Potent and selective C-C chemokine receptor 4 (CCR4) antagonists inhibit regulatory T cell recruitment, increase effector T cell numbers, and potentiate anti-tumor responses in mice. J Immunother Cancer. 2017; 5(Suppl 2):467.
3. Okal A, Ho W, Wong B, Kassner P, Cutler G. Patient selection strategies and pharmacodynamic assays for CCR4 antagonists. J Immunother Cancer. 2017; 5(Suppl 2): 44.

**Ethics Approval**

Approved by the Independent Ethics Committee of the foundation “Stichting Beoordeling Ethiek Biomedisch Onderzoek” (Assen, The Netherlands), Study Code FLB307EC-173071, CCMO code NL63737.056.17.

#### P485 Testing of two bispecific SNIPER™ antibodies targeting mouse tumor-infiltrating Tregs

##### Anna Hoefges, MS^1^, Bonnie Hammer^2^, Amy Erbe, PhD^1^, Alexander Rakhmilevich, MD, PhD^1^, Jacquelyn Hank, PhD^1^, Bryan Glaser, PhD^2^, Lucas Bailey, PhD^2^, Roland Green, PhD^2^, Paul Sondel, MD, PhD^1^

###### ^1^University of Wisconsin Madison, Madison, WI, USA; ^2^Invenra, Madison, WI, USA

####### **Correspondence:** Anna Hoefges (hoefges@wisc.edu)

**Background**

T regulatory cells (Tregs) are essential to help prevent autoimmune diseases. In the setting of cancer, however, Tregs can help cancer evade anti-tumor immunity by suppressing immunity. Murine studies have shown that if Tregs are selectively depleted, anti-tumor immunity can be enhanced and synergistic immunotherapy achieved, promoting tumor regression. However, currently available Treg-depletion agents can be non-specific and deplete/suppress other T cells, can fail to sufficiently deplete Tregs, or can potently deplete all Tregs, leading to toxic autoimmunity. We have developed and tested a way to selectively eliminate Tregs in the tumor microenvironment (TME) while leaving peripheral Tregs by using bispecific mAbs created using Invenra’s SNIPER™ technology. SNIPER™ bispecific antibodies have relatively weak affinity for two separate targets, limiting their binding and activity when only one target is present. However, when both targets are present, binding is much stronger due to the avidity effect. This allows specific subpopulations of cells to be more specifically selected for elimination by antibody drug conjugates or antibody dependent cellular cytotoxicity.

**Methods**

Two separate SNIPER™ bispecific mAbs, Inv-1 and Inv-2, were created. C57Bl/6 mice were injected with B78 melanoma tumors. Established tumors and spleens were harvested from mice and analyzed by flow cytometry to identify T cell populations and binding specificity of Inv-1 and Inv-2.

**Results**

We analyzed binding of the Inv-1 and Inv-2 to lymphocytes harvested from spleens and tumors from the B78 tumor-bearing mice. We used a standard Treg verification panel (CD4, CD25, Foxp3) to identify known Treg populations. Separate panels included the bispecific antibodies (Inv-1 or Inv-2). We found that Inv-1 binds to 59% of Foxp3+ cells extracted from the TME, but only to 18% of the splenic Foxp3+ cells. This shows a preferential binding for tumor-infiltrating-Tregs. Separately, Inv-2 bound to 81% of Foxp3+ cells extracted from the TME, but only to about 51% of the splenic Foxp3+ cells.

**Conclusions**

Both Inv-1 and Inv-2 selectively target Tregs, with a preference for Tregs present in the TME. In vivo administration of these antibodies may allow for selective depletion of tumor-associated Tregs. Selective depletion of TME-Tregs may result in a reduction in toxic autoimmune side effects associated with immune-activation in the setting of global Treg depletion. In turn, the removal of Tregs specifically from the TME, coupled with a reduction of potential toxic side effects, may enhance the efficacy and applicability of combining Treg depletion with other immune-activating immunotherapies.

#### P486 Antisense oligonucleotides targeting CD39 and PD-L1 modulate the immunosuppressive tumor microenvironment and have potent anti-tumor activity

##### Frank Jaschinski, PhD^1^, Tamara Thelemann^1^, Richard Klar, PhD^1^, Monika Schell^1^, Lisa Hinterwimmer^1^, Sven Michel^1^, Melanie Buchi^2^, Abhishek Kashyap^2^, Alfred Zippelius, MD^2^

###### ^1^Secarna Pharmaceuticals GmbH & Co. KG, Planegg-Martinsried, Germany; ^2^University of Basel, Basel, Switzerland

####### **Correspondence:** Frank Jaschinski (frank.jaschinski@secarna.com)

**Background**

Antisense oligonucleotides (ASOs) are a new therapeutic modality and have the potential to suppress expression of any RNA target. On the one hand they allow selective targeting of factors previously considered as undruggable, on the other hand -due to their different pharmacokinetic and pharmacodynamic properties- they can offer a complementary approach to more established modalities such as small molecule drugs or antibodies. In the present study, locked-nucleic-acid (LNA)-modified antisense oligonucleotides targeting PD-L1 and the ectonucleotidase CD39 were designed and their activity was tested in cell culture and syngeneic mouse models

**Methods**

In vitro activity of ASOs on target mRNA and protein expression was investigated in tumor cell lines and confirmed in isolated human T cells. Degradation of extracellular ATP and proliferation of immune cells were tested in isolated human T cells. In vivo, target activity and investigation of frequency of intratumoral Treg were investigated in the syngeneic MC38 mouse model. The MC38 model and the syngeneic EMT6 model were used to test effects on tumor growth or survival.

**Results**

In vitro, unformulated ASOs targeting PD-L1 and CD39 achieved potent target knockdown on mRNA and protein level in tumor cell lines and in isolated human T cells. CD39-specific ASOs potently reduced degradation of extracellular ATP in T cells. While treatment of T cells with ATP potently suppressed their proliferation, CD39- specific ASOs could reverse this effect. In syngeneic mouse tumor models, systemic treatment with CD39-specific ASO resulted in potent knockdown of CD39 expression e.g. in Treg, tumor-associated macrophages and myeloid- derived suppressor cells and in a reduction of the frequency of intratumoral Treg. Moreover, tumor growth was strongly reduced by CD39-specific ASO, as monotherapy. In combination with PD-1 antibodies, anti-tumor efficacy of antibodies was improved by ASO.Anti-tumor efficacy of-murine PD-L1 ASOs was demonstrated in syngeneic mouse models. In a breast cancer model, all tumor-bearing mice treated with the PD-L1 ASO rejected the tumor and remained tumor-free. Upon rechallenge, the vast majority of mice rejected the tumor cells demonstrating immunological memory formation. No signs of toxicity were observed.

**Conclusions**

We have shown, that ASOs targeting immunosuppressive factors are able to achieve potent target suppression in the relevant cell types in vivo and can induce potent anti-tumor effects as monotherapy and in combination therapy with antibody-based checkpoint inhibitors, thereby enhancing survival. Taken together, we developed innovative immunotherapeutic tools that will potentially improve treatment options for cancer patients in the future.

**Ethics Approval**

PBMC were obtained from leukapheresis products (Klinikum rechts der Isar, TU München, ethics commission reference: 329/16 S)

#### P487 The role of MultiOmyx in illustrating the pancreatic tumor microenvironment

##### Juncker-Jensen Juncker-Jensen, PhD, Jun Fang, Judy Kuo, Mate Nagy, Qingyan Au, Eric Leones, Flora Sahafi, RaghavKrishna Padmanabhan, Nicholas Hoe, Josette William, PhD, MD

###### NeoGenomics, Aliso Viejo, CA, USA

####### **Correspondence:** Juncker-Jensen Juncker-Jensen (anna.juncker-jensen@neogenomics.com)

**Background**

Pancreatic ductal adenocarcinoma (PDAC) is characterized by an excessive amount of desmoplastic stroma seeded with inflammatory cells and it is one of the most aggressive forms of cancer with no current specific therapies. Tumor-associated macrophages (TAMs) are a major component of the tumor microenvironment (TME), and in most solid cancers increased TAM infiltration is associated with a poor prognosis. TAMs can be described as classically activated M1 types with pro-inflammatory antitumor functions, versus alternatively activated M2 types with immunosuppressive pro-tumor functions. The immunosuppressive functions of M2 TAMs can be exerted through release of cytokines and growth factors as well as via direct recruitment of T regulatory cells (Tregs), a subset of lymphocytes responsible for immune tolerance of the system to the tumor. While the differentiation from M1 to M2 in PDAC has been shown to be associated with a worse prognosis [1], not much is known about PDAC TAM polarization and its potential correlation to Treg recruitment.

**Methods**

We have used MultiOmyx, a proprietary, multiplexing assay with similar staining characteristics as standard IHC stains but with the significant advantage that 60 protein biomarkers can be interrogated from a single FFPE section. MultiOmyx protein immunofluorescence (IF) assays utilize a pair of directly conjugated Cyanine dye-labeled (Cy3, Cy5) antibodies per round of staining. Each round of staining is imaged and followed by novel dye inactivation chemistry, enabling repeated rounds of staining and deactivation.

**Results**

Using the pan macrophage marker CD68 in combination with either M1 marker HLA-DR or M2 marker CD163 we confirmed the presence of M1 (CD68+HLA-DR+) and M2 (CD68+CD163+) populations in 9 stage IIB non- metastatic PDAC FFPE samples, the vast majority being of the M2 subtype. Moreover, we found a positive significant correlation (Pearson’s correlation p<0.05) between the presence of M2 TAMs and Tregs (CD3+CD4+FoxP3+), but not between M1 TAMs and Tregs. Using our proprietary algorithms that takes into account the staining pattern for each specific biomarker, we will now examine the spatial relationship between the M1/M2 subtypes of TAMs and Tregs in the stromal and intratumoral areas and compare our findings to those found in samples from patients with stage IV metastatic PDAC.

**Conclusions**

We demonstrate a positive significant correlation between the presence of M2-TAMs and Tregs in the TME of PDAC, suggesting a possible pathway in which TAM-polarization plays an immunosuppressive function by recruiting Tregs.

**References**

1. Kurahara H et al. Significance of M2-polarized tumor-associated macrophages in pancreatic cancer. JSurgRes. 2011;167:e211-219

#### P488 Targeting hIDO1 with 3rd generation antisense oligonucleotides for modulation of the tumor microenvironment

##### Richard Klar, PhD^1^, Sandra Kallert, PhD^2^, Tamara Thelemann^1^, Sven Michel^1^, Monika Schell^3^, Lisa Hinterwimmer^1^, Alfred Zippelius, MD^2^, Frank Jaschinski, PhD^3^

###### ^1^Secarna pharmaceuticals GmbH & Co. KG, Planegg/Martinsried, Germany; ^2^University of Basel, Department of Biomedicine, Switzerland; ^3^Secarna pharmaceuticlas GmbH & Co. KG, Planegg-Martinsried, Germany

####### **Correspondence:** Frank Jaschinski (frank.jaschinski@secarna.com)

**Background**

Targeting the immunosuppressive microenvironment of tumors has emerged as a promising treatment option for oncologic indications in the last years. However, despite long lasting remissions in a small subset of patients the majority does not respond to the currently available immunotherapies, possibly caused by the existence of a plethora of immune suppressive factors. One of those factors is indoleamin-2,3-dioxygenase 1 (IDO1), an enzyme that degrades tryptophan to kynurenines which in turn can result in a suppression of immune effector cells.

**Methods**

As an alternative approach to small molecule IDO1-inhibitors, we designed antisense oligonucleotides (ASOs) with specificity for human IDO1 (hIDO1). ASOs were synthesized as GapmeRs with flanking locked nucleic acids to increase stability and affinity to the target RNA. The knockdown efficacy of ASOs on the mRNA and protein level was investigated in cancer cells and human immune cells without addition of a transfection reagent. The effect of hIDO1 knockdown in cancer cells on the production of L-Kynurenine and the proliferation of cocultured T cells was investigated. We furthermore developed ASOs with specificity for murine IDO1 (mIDO1) to investigate the efficacy of ASO-mediated IDO1 knockdown in syngeneic tumor models.

**Results**

We identified a subset of ASOs that resulted in a hIDO1 mRNA knockdown of >90% in cancer cell lines. Two highly potent ASOs with IC50 values in the low nanomolar range were selected for further experiments. The treatment of cancer cells as well as human immune cells resulted in reduction of IDO protein levels by >85%. Importantly, we observed a complete block in the production of immunosuppressive L-Kynurenine in ASO treated cells and IC50 values in the low nanomolar range. In line with those results, we observed a strongly increased proliferation of T cells when hIDO1 was knocked down in cocultured tumor cells. Preliminary in vivo experiments suggest that treatment of tumor-bearing mice with mIDO1-specific ASOs results in the knockdown of IDO1 in tumor cells as well as tumor infiltrating myeloid cells.

**Conclusions**

We selected highly potent hIDO1 ASOs that efficiently knock down hIDO1 mRNA and protein in cancer cells as well as primary human cells and potently reduce the immunosuppressive capacity of cancer cells. Potent mouse specific IDO1 ASOs have been identified and will be used for in vivo efficacy studies in tumor-bearing mice. Taken together, we developed an innovative immunotherapeutic tool to block the expression of hIDO1 that will potentially improve treatment options for cancer patients in the future.

**Ethics Approval**

PBMC were obtained from leukapheresis products (Klinikum rechts der Isar, TU München ethics commission reference: 329/16 S)

#### P489 Prognostic significance of tumor-associated macrophage content in head and neck squamous cell carcinoma: A meta-analysis

##### Ayan Kumar, BS, Alexander Knops, BA, Brian Swendseid, MD, Ubaldo Martinez-Outschoom, MD, Larry Harshyne, PhD, Nancy Philp, PhD, Ulrich Rodeck, MD PhD, Christopher Snyder, Adam Luginbuhl, MD, David Cognetti, MD, Jennifer Johnson, MD, Joseph Curry, MD

###### Thomas Jefferson University, Philadelphia, PA, USA

####### **Correspondence:** Joseph Curry (joseph.curry@jefferson.edu)

**Background**

Head and neck squamous cell carcinoma (HNSCC) develops within a complex cellular microenvironment that promotes tumor growth, thus providing multiple potential therapeutic targets. Among those are macrophages, which are abundant in and around tumor tissue, and have been implicated in the growth, development, and persistence of HNSCC [1]. However, the relationship between the density and composition of tumor-associated macrophages (TAMs) and clinicopathologic markers of disease is poorly defined [2,3]. Inconsistent findings may be a result of differences in approach to TAM detection. Some authors have measured total macrophage content in tumor tissue, while others have stained tumor samples for individual subtypes of TAMs, which include M1 (pro-inflammatory) and M2 (immunosuppressive). Here we review the published evidence concerning the relationship of the phenotypes of tumor-associated macrophages with HNSCC prognosis.

**Methods**

We conducted a meta-analysis of the existing publications investigating the relationship between TAM density (total and M2 subtype) and T stage, nodal involvement, vascular invasion, lymphatic invasion, and tumor differentiation (Figure 1). A total of thirteen studies were included (Table 1) [2-14]. Forest plots and risk ratios were generated to illustrate overall effects.

**Results**

Higher density of both total and M2 subtype of TAMs in the tumor microenvironment is associated with advanced T stage, increased rates of nodal positivity, presence of vascular invasion, and presence of lymphatic invasion (p < 0.0001; Figures 2-5). There is no significant association between TAM density, either total or M2 subtype, and tumor differentiation (Figure 6).

**Conclusions**

Increased TAM density, particularly those of the M2 phenotype, correlates with poor clinicopathologic markers in HNSCC and is associated with poor clinical prognosis. Yet, it is unknown whether and how TAMs contribute to poor prognosis in this tumor type. Additional investigation into the mechanisms behind TAM recruitment and polarization will help define the feasibility of TAM-targeted therapies.

**References**

1. Marcus B, Arenberg D, Lee J, et al. Prognostic factors in oral cavity and oropharyngeal squamous cell carcinoma. Cancer. 2004; 101(12):2779-2787.
2. Fang J, Li X, Ma D, et al. Prognostic significance of tumor infiltrating immune cells in oral squamous cell carcinoma. BMC Cancer. 2017; 17.
3. Shigeoka M, Urakawa N, Nakamura T, et al. Tumor associated macrophage expressing CD204 is associated with tumor aggressiveness of esophageal squamous cell carcinoma. Cancer Science. 2013; 104(8):1112-1119.
4. Balermpas P, Rödel F, Liberz R, et al. Head and neck cancer relapse after chemoradiotherapy correlates with CD163+ macrophages in primary tumour and CD11b+ myeloid cells in recurrences. Br J Cancer. 2014; 111(8):1509-1518
5. Fujii N, Shomori K, Shiomi T, et al. Cancer‐associated fibroblasts and CD163‐positive macrophages in oral squamous cell carcinoma: their clinicopathological and prognostic significance. Journal of Oral Pathology & Medicine. 2012; 41(6):444-451.
6. Hu Y, He M-Y, Zhu L-F, et al. Tumor-associated macrophages correlate with the clinicopathological features and poor outcomes via inducing epithelial to mesenchymal transition in oral squamous cell carcinoma. J Exp Clin Cancer Res. 2016; 35.
7. Huang H, Liu X, Zhao F, et al. M2-polarized tumour-associated macrophages in stroma correlate with poor prognosis and Epstein-Barr viral infection in nasopharyngeal carcinoma. Acta Otolaryngol. 2017; 137(8):888-894.
8. Liu S-Y, Chang L-C, Pan L-F, Hung Y-J, Lee C-H, Shieh Y-S. Clinicopathologic significance of tumor cell-lined vessel and microenvironment in oral squamous cell carcinoma. Oral Oncology. 2008; 44(3):277-285.
9. Lu C-F, Huang C-S, Tjiu J-W, Chiang C-P. Infiltrating macrophage count: a significant predictor for the progression and prognosis of oral squamous cell carcinomas in Taiwan. Head Neck. 2010; 32(1):18-25.
10. Matsuoka Y, Yoshida R, Nakayama H, et al. The tumour stromal features are associated with resistance to 5‐FU‐based chemoradiotherapy and a poor prognosis in patients with oral squamous cell carcinoma. APMIS. 2014;123(3):205-214.
11. Sugimura K, Miyata H, Tanaka K, et al. High infiltration of tumor‐associated macrophages is associated with a poor response to chemotherapy and poor prognosis of patients undergoing neoadjuvant chemotherapy for esophageal cancer. Journal of Surgical Oncology. 2015; 111(6):752-759.
12. Wang S, Sun M, Gu C, et al. Expression of CD163, interleukin-10, and interferon-gamma in oral squamous cell carcinoma: mutual relationships and prognostic implications. Eur J Oral Sci. 2014; 122(3):202-209.
13. Yamagata Y, Tomioka H, Sakamoto K, et al. CD163-positive macrophages within the tumor stroma are associated with lymphangiogenesis and lymph node metastasis in oral squamous cell carcinoma. Journal of Oral and Maxillofacial Surgery. 2017; 75(10):2144-2153.
14. Zhu Y, Li M, Bo C, et al. Prognostic significance of the lymphocyte-to-monocyte ratio and the tumor-infiltrating lymphocyte to tumor-associated macrophage ratio in patients with stage T3N0M0 esophageal squamous cell carcinoma. Cancer Immunol Immunother. 2017; 66(3):343-354.

**Fig. 1 (abstract P489).**
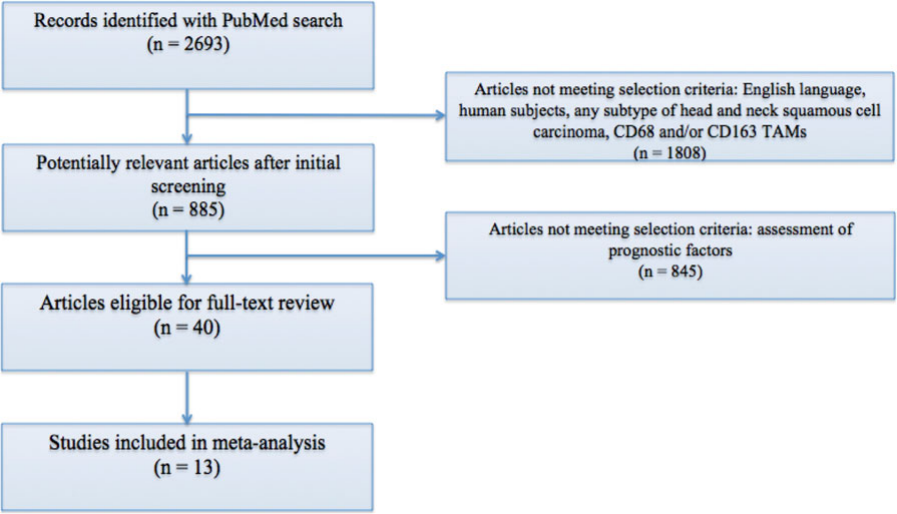
See text for description

**Table 1 (abstract P489).**
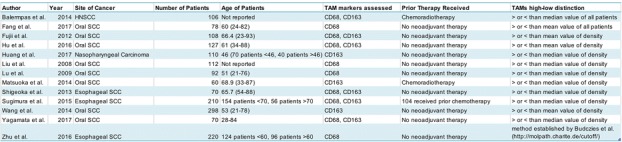
See text for description

**Fig. 2 (abstract P489).**
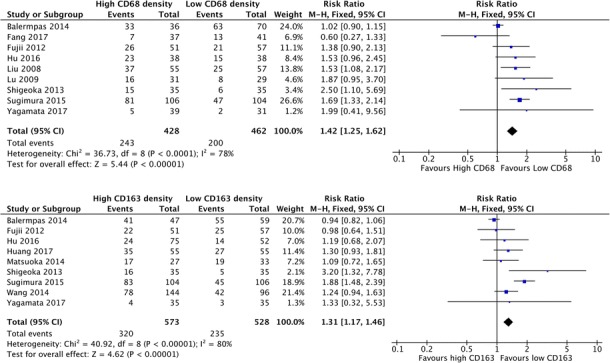
See text for description

**Fig. 3 (abstract P489).**
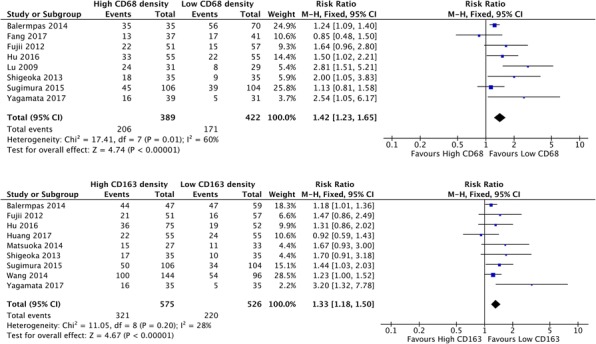
See text for description

**Fig. 4 (abstract P489).**
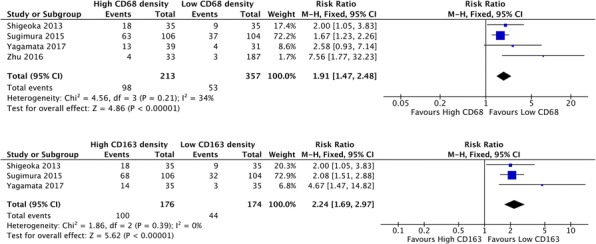
See text for description

**Fig. 5 (abstract P489).**
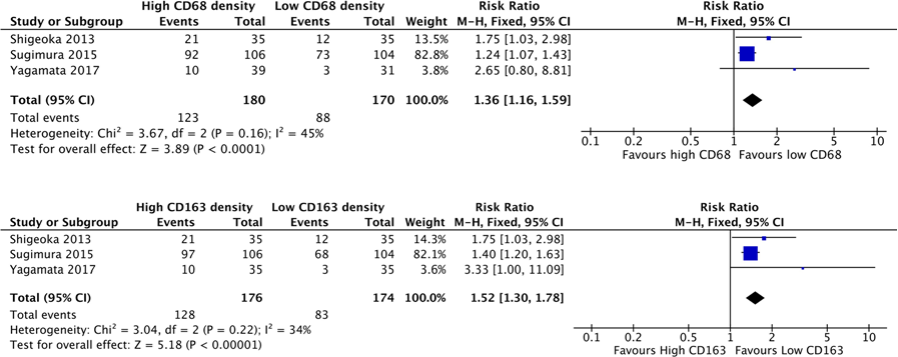
See text for description

**Fig. 6 (abstract P489).**
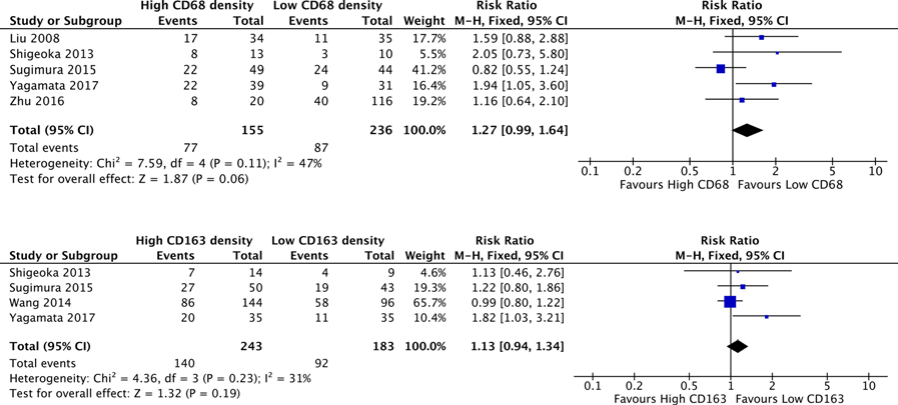
See text for description

#### P490 Characterization of the tumor immune microenvironment in treatment-naïve EGFR-mutant NSCLC uncovers a suppressed immune phenotype

##### Xiuning Le^1^, Alexandre Reuben^1^, Marcelo Negrao, MD^1^, Won-Chul Lee, PhD^1^, Edwin Parra^1^, Carmen Behrens, MD^1^, Humam Kadara, PhD^2^, Ignacio Wistuba, MD^1^, Jianjun Zhang, MD, PhD^1^, John Heymach^1^

###### ^1^MD Anderson Cancer Center, Houston, TX, USA; ^2^American University of Beirut, Beirut, Lebanon

####### **Correspondence:** John Heymach (jheymach@mdanderson.org)

**Background**

Although immune checkpoint blockade (ICI) has been successfully utilized in treating patients with non-small cell lung cancer (NSCLC), the benefit of ICI for patients with advanced EGFR-mutant NSCLC has been limited. Intriguingly, recent data from IMpower150 subgroup analysis demonstrated that combination of VEGF blockade with anti-PD-L1 and chemotherapy induces response in patients with EGFR/ALK driven NSCLC, suggesting modulating the tumor microenvironment may enhance response to anti-PD-1 blockade in EGFR/ALK lung cancers. In order to develop effective immune therapy combinations, it is critical to understand tumor immune microenvironment (TME) and identify negative regulators in EGFR-mutant lung cancer for rational design of clinical trials. [1]

**Methods**

We leveraged a published set of stage I-III lung adenocarcinomas with immune profiling and sequencing data (PROSPECT cohort, Kadara et al Ann Oncol 2018). We selected a set of 94 adenocarcinomas including 14 cases with EGFR-sensitizing mutations with immune profiling by immunohistochemistry for ten immune markers (PD- L1, PD-1, CD3, CD4, CD8, CD45RO, CD57, CD68, FoxP3 and Granzyme B). Gene microarray data were also available to evaluate the tumor microenvironment while CIBERSORT was used to infer immune cell subpopulations.

**Results**

PDL1 and GzmB were significantly lower in the EGFR-mutant cases, as expected. CD4 was higher in EGFR-mutant tumor center. Forty-four key immune regulators’ levels we evaluated to further evaluate the tumor microenvironment. IFNG was lower while TGFbeta was higher in EGFR-mutant cases, suggesting a suppressive TME. Other negative regulators, including CTLA4, LAG3, TIM3, TIGIT, IL6 and VEGFA were not differentially expressed. Lastly, CIBERSORT analysis revealed CD4+ memory T cells were decreased in the EGFR-mutant cases.

**Conclusions**

Results from this analysis are consistent with prior knowledge of EGFR-mutant NSCLC, demonstrating a PD-L1 low, GzmB low, IFNG low and TGFbeta high immune phenotype, suggesting a suppressive tumor microenvironment. CD4+ T cells composition needs to be further understood as subpopulation of CD4+ T cells (T regs) might be contributing to the suppressed TME. These results represent an initial step for rationale combination of immune therapy to modulate the suppressive TME, which might lead to enhanced treatment efficacy to benefit patients with EGFR-mutant lung cancers.

**References**

1. Kadara H, et al. Ann Oncol. 2018 29(4):1072

#### P491 The DNA methyltransferase inhibitor, guadecitabine, has beneficial immunomodulatory effects on myeloid derived suppressor cells, and augments adoptive immunotherapy for solid tumors

##### Andrea Luker, PhD candidate, Harry Bear, M.D., Ph.D., Daniel Conrad, Ph.D., Laura Graham

###### Virginia Commonwealth University, Richmond, VA, USA

####### **Correspondence:** Harry Bear (harry.bear@vcuhealth.org)

**Background**

Myeloid Derived Suppressor Cells (MDSC) are a regulatory population that accumulates in tumor microenvironments. They are a significant hurdle in treating cancer because they dampen anti-tumor responses as well as hinder the effects of immunotherapy. Here we show that the DNA methyltransferase inhibitor (DNMTi), Guadecitabine, has beneficial immunomodulatory effects on MDSCs and 4T1 tumor cells, both *in vitro* and *in vivo*.

**Methods**

Purified MDSCs or 4T1 cells were cultured for 48-72 hours with 1μM Guadecitabine and then analyzed for surface molecules by flow cytometry or further incubated with fluorescently-labeled Ovalbumin for 30 minutes to allow for antigen uptake. 4T1 tumors (50k, flank) were established in syngeneic Balb/c mice for 10 days before administering four daily doses of Guadecitabine (50μg, i.p.); mice were sacrificed at day 16 to collect tissues for analysis. Guadecitabine and AIT (50 million expanded draining lymph node cells) were administered concurrently starting at day 3, and tumor size was monitored until a humane endpoint. All experiments include n=3+ with at least two experimental repeats.

**Results**

MDSCs cultured with Guadecitabine had increased expression of MHC II, CD80, and CD86 exclusively in Ly6C+cells, with no effect on the Ly6G+ subpopulation. These Ly6C+ MDSCs also demonstrated significantly enhanced antigen uptake *in vitro*. Guadecitabine-treated 4T1 cells exhibited significantly increased MHC I and PD-L1 expression over vehicle-treated cells. Guadecitabine treatments in 4T1 tumor-bearing mice immediately reduced the tumor-induced MDSC accumulation in the spleen (Figure 1), bone marrow, and blood, thereby restoring normal cellularity. Similar to the *in vitro* findings, the remaining MDSCs in Guadecitabine-treated mice had increased levels of MHC II and co-stimulatory molecules. Subsequently, animals sacrificed at day 16 exhibited a significant reduction in tumor area (Figure 2). Finally, we tested the effectiveness of Guadecitabine in combination with T cell adoptive immunotherapy (AIT). Individually, Guadecitabine or AIT alone slowed the initial tumor progression over the first 2-3 weeks. When applied together, however, there was an additional synergistic effect that strongly suppressed tumor growth and prolonged overall survival for an additional 2+ weeks (Figure 3).

**Conclusions**

*In vitro*, the DNMTi Guadecitabine selectively alters the Ly6C+ MDSC subpopulation toward an immune-stimulatory phenotype, and induces expression of immunogenic surface molecules on 4T1 cells. *In vivo*, treatment with Guadecitabine reduces tumor burden by mainly affecting MDSC accumulation and phenotype. In addition, Guadecitabine enhances the effectiveness of AIT to suppress tumor progression and prolong overall survival.

**Acknowledgements**

We would like to thank Astex Pharmaceuticals for providing the Guadecitabine, as well as the Massey Cancer Center for pilot grant funding.

**Ethics Approval**

These studies were conducted with the permission and oversight of the VCU Institutional Animal Care and Use Committee.

**Fig. 1 (abstract P491).**
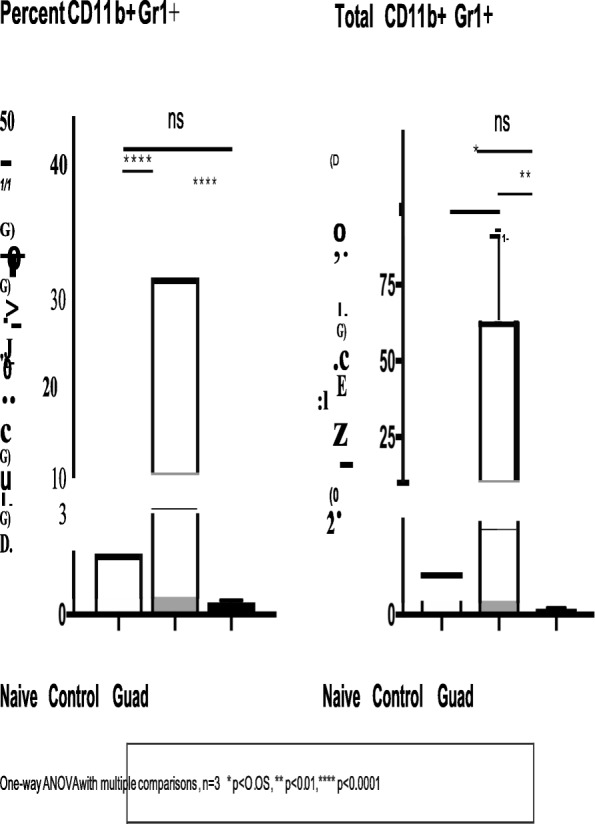
See text for description

**Fig. 2 (abstract P491).**
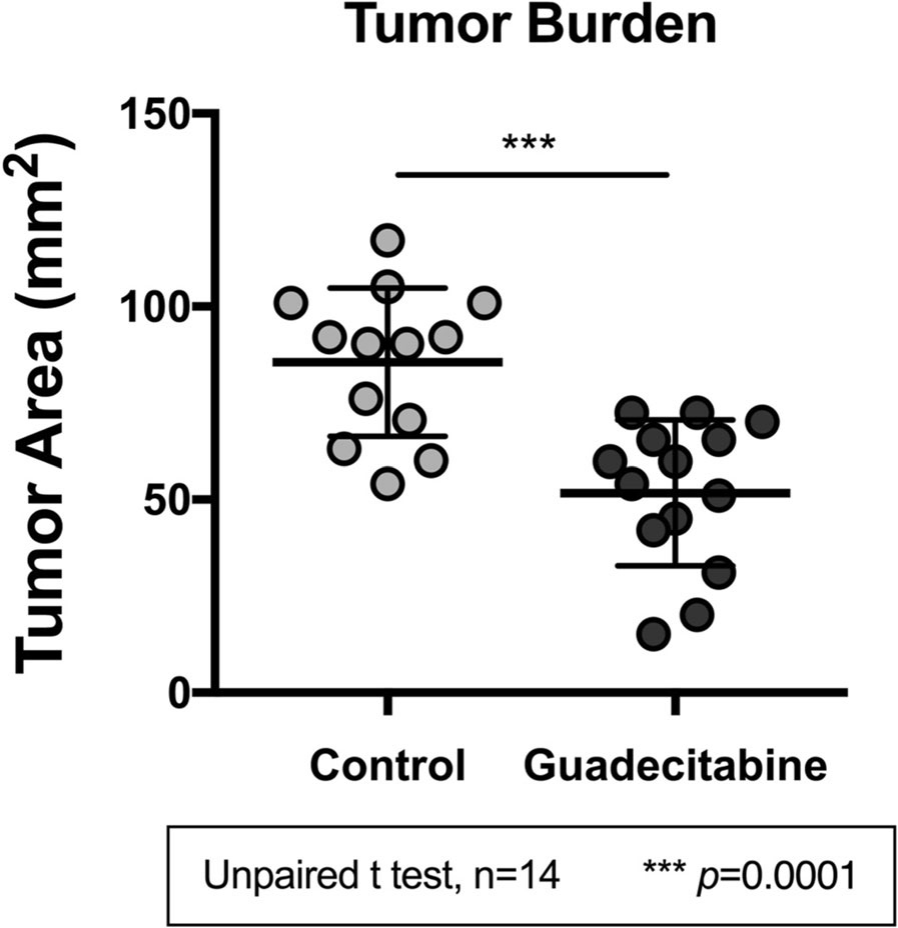
See text for description

**Fig. 3 (abstract P491).**
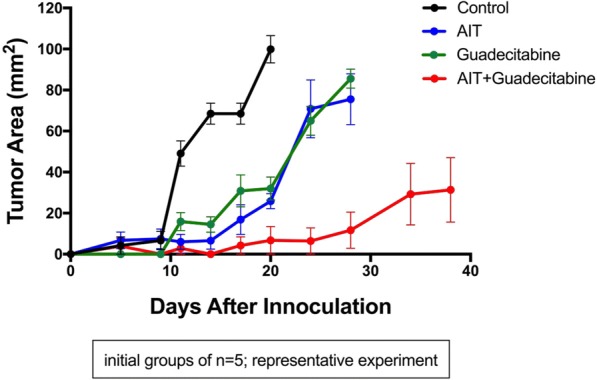
See text for description

#### P492 Myeloid-derived suppressor cells (MDSC) assessment using a fully automated sequential chromogenic multiplex assay

##### Anna Martirosyan, Dr, Assil Benchaaben, Aurélie Collignon, MS, Emilie Bonzom, Trainee, Matthieu Duval, Apprentice, Emmanuel Prestat, PhD, Christophe Haond, Jacques Fieschi, PhD

###### HalioDx, Marseille, France

####### **Correspondence:** Jacques Fieschi (Jacques.Fieschi@haliodx.com)

**Background**

Despite significant advances in the recent years, the response rate to immune checkpoint inhibitor therapies for non-small cell lung cancer (NSCLC) is only about 20%. There is a strong and urgent need to identify new diagnostic biomarkers to predict which patients can benefit from an immune checkpoint blocker treatment. Extensive animal data and several clinical trials indicate that immunosuppression is a limiting factor of effective anti-tumoral immunotherapy. In this context, the presence of immunosuppressive elements such as myeloid-derived suppressor cells (MDSC) in the tumoral microenvironment might be a major factor contributing to resistance to checkpoint inhibitors. In recent years, several studies have shown a correlation between the level of MDSC and stage, overall survival, and response to therapy in NSCLC patients. For instance circulating MDSC were negatively associated with the immune response to cancer vaccine. Furthermore, the accumulation of MDSC has also been reported to correlate with the progression-free survival and the response to chemotherapy, as well as metastatic burden in NSCLC patients. Last, but not least the intra-tumoral accumulation of MDSC is associated with unfavorable prognosis.

**Methods**

Here we assessed the presence and abundance of this major immunoregulatory population within the NSCLC microenvironment by using an automated sequential chromogenic multiplex assay.

**Results**

A unique combination of biomarkers (CD11b, CD15, HLA-DR, CD14, LOX1, and S100A9) was developed to detect and quantify different populations of MDSC on a single FFPE tumor tissue section. Briefly, a tissue section was sequentially stained, digitized, unstained and re-stained with antibodies targeting the six markers. Images of the whole slide were then analyzed by digital pathology: first, a newly developed software was used to co-register the 6 virtual slides and perform colors deconvolution. Then detection of positive cells was performed for each marker independently, using Indica Lab’s HALO software. The M-MDSC (monocytic MDSC) and PMN-MDSC (polymorphonuclear MDSC) populations were defined as being respectively CD11b+CD14+HLA- DRnegativeS100A9+cells and CD11b+CD15+HLA-DRnegativeLOX1+cells. In addition, tissue segmentation tools were used to assess MDSC densities in parenchyma, tumor stroma and invasive margin regions.

**Conclusions**

The detection and quantification of MDSC in NSCLC patients could be a key parameter to predict patient’s responses to anti-tumoral immunotherapy. This new tool will allow to evaluate the immunosuppressive landscape of NSCLC tumors.

#### P493 B cells in glioblastoma are associated with diminished survival

##### Ileana Mauldin, PhD, Jasmin Jo, MD, Nolan Wages, PhD, Samuel Young, BS, Loren Erickson, PhD, Maria- Beatriz Lopes, MD, PhD, Craig Slingluff, MD, Camilo Fadul, MD, FAAN

###### University of Virginia, Charlottesville, VA, USA

####### **Correspondence:** Camilo Fadul (fadul@virginia.edu)

**Background**

The demonstrated efficacy of immunotherapy, in the treatment of some cancers, has not been observed with glioblastoma (GBM). Characterization of the immune infiltrates in GBM may identify targets to drive the immune activation that improves the efficacy of immunotherapy. We have observed that GBM can harbor dense B cell infiltrates usually found cuffing blood vessels. B cells can act as antigen presenting cells driving local immune activation, or as regulatory B cells (Bregs) to suppress immune activation; they may also be localized in tertiary lymphoid structures (TLS) usually associated with better patient survival. The prevalence and role of B cell infiltrate in GBM is unknown.

**Methods**

Surgically-resected GBM from 48 patients were evaluated for immune infiltrate by multispectral Immunohistochemistry using the OPAL staining system, Vectra3 microscope, and Inform Software (PerkinElmer). Three multiplex panels were designed to enumerate CD20 infiltrates from three serial sections of tumors and to evaluate additional immune markers. RNA-seq gene expression data from the Cancer Genome Atlas (TCGA) was evaluated from GBM patients.

**Results**

In univariate assessments, low CD20+ cell densities were associated with better overall survival (OS) (median OS 31.7 months for 12 CD20low patients vs. 18.7 months for 36 CD20high patients, p=0.019). Multivariate analyses were done to assess key prognostic factors (CD20 density (low vs high), Karnofsky performance status (KPS; high vs. low), age, and MGMT methylation (methylated vs unmethylated). Decreased OS was observed from patients with high CD20+ infiltrate (Hazard ratio (HR)=2.84, p=0.03) and longer OS was associated with MGMT methylation (HR=0.49, p=0.04). Differences in age (HR=1.13, p=0.05) and KPS (HR=0.49, p=0.08) were trending in significance. We found that B cell infiltrates in GBM do not constitute TLS when examined for high endothelial venules. TCGA data also suggests that in GBM overexpression of MS4A1 (encoding CD20) is associated with decreased OS (p=0.01).

**Conclusions**

Our studies suggest a correlation between B cells and diminished patient survival, thus CD20 infiltrates may constitute a novel prognostic factor for GBM. We hypothesize that B cells in GBM may function as Bregs which would suppress immune activation. However, this hypothesis needs to be further evaluated by examining Breg function in tumors and evaluating the impact of GBM-infiltrating B cells on tumor-infiltrating T cells.

**Ethics Approval**

The study was approved by the University of Virginia's Ethics Board, approval number 20210.

#### P494 Targeting the CCR2/MCP-1 chemokine axis for cancer therapy

##### Payal Mittal^1^, Tatiana Akimova^2^, Craig Leach^3^, Jose Clemente^3^, Matthew Sender^3^, Liqing Wang^2^, Joseph Marino^3^, Yao Chen^3^, Peiling Chen^3^, Brandon Turunen^3^, Wayne Hancock, MD, PhD^2^, Payal Mittal, MD^2^

###### ^1^Children's hospital of Philadelphia/GSK, Philadelphia, PA, USA; ^2^Children's hospital of Philadelphia, Philadelphia, PA, USA; ^3^GlaxoSmithKline, Collegeville, PA, USA

####### **Correspondence:** Wayne Hancock (Whancock@pennmedicine.upenn.edu)

**Background**

Host anti-tumor responses, including the actions of cytotoxic T cells, play a key role in curtailing tumors, but can be hindered by multiple mechanisms active within the tumor microenvironment. One such mechanism involves the recruitment and expansion of immunosuppressive cells such as myeloid derived suppressor cells (MDSCs) in the tumor microenvironment. We are targeting the signaling pathways implicated in recruitment of MDSCs in tumor microenvironment.

**Methods**

We have utilized transplantable lung tumor cell line (TC1) to address the role of the CCR2/MCP-1 axis in MDSC associated tumor progression. Additionally, I utilized CCR2 knockout mice to demonstrate the dependence of progression of TC1 tumor on CCR2 signaling.I also used a thioglycolate-induced peritonitis model of inflammation to validate the ability of a CCR2 antagonist to inhibit trafficking of MDSCs to the site of inflammation and then tested the compound at this inhibitory concentration for its ability to impair CCR2 chemokine signaling within established tumors.

**Results**

Phenotypic profiling of TC1 tumors revealed maximal expression of CCR2 by tumor resident MDSCs, and MCP-1 by transplanted tumor cells, tumor associated macrophages (TAMs) and tumor associated neutrophils (TANs) respectively. Additionally, utilization of CCR2 knockout mice showed the dependence of progression of TC1 tumor on CCR2 signaling (tumors were significantly smaller in CCR2KO mice compared to WT mice). We used a thioglycolate-induced peritonitis model of inflammation to validate the ability of a CCR2 antagonist to inhibit trafficking of MDSCs to the site of inflammation in a dose-dependent manner with a maximal effect at a dose of 10 mg/kg, and then tested the compound at this inhibitory concentration for its ability to impair CCR2 chemokine signaling within established tumors. CCR2 antagonist promoted antitumor immunity in TC1 tumors.

**Conclusions**

In summary, genetic and pharmacologic data indicate that CCR2 targeting may be an important new component of immuno-oncology based therapies.

#### P495 Pegfilgrastim, but not Plinabulin, generates a blood myeloid cell (BMC) repertoire with a predominant immunosuppressive phenotype

##### Douglas Blayney, MD^1^, Stephan Ogenstad^2^, Yuankai Shi^3^, Lihua Du^4^, Lan Huang^5^, Ramon Mohanlal, MD, PhD, MBA^5^

###### ^1^Stanford Cancer Institute, Stanford, CA, USA; ^2^Statogen Consulting, LLC, Zebulon, NC, USA; ^3^Chinese Academy of Medical Sciences, Beijing, China; ^4^Wanchun Bulin Pharmaceuticals Limited, Dalian, China; ^5^BeyondSpring Pharmaceuticals, Inc., New York, NY, USA

####### **Correspondence:** Ramon Mohanlal (rmohanlal@beyondspringpharma.com)

**Background**

Tumors recruit BMC to the tumor microenvironment and modulate BMCs [immunosuppressive tumor-associated macrophages (TAM), neutrophils (TAN), and myeloid derived suppressor cells (MDSC)] in tumor microenvironment (Schupp, Cellular Immunology, 2017; Ginhoux, Nat Rev Immunology, 2014). Predominantly immature BMCs are associated with poor prognosis (Bergenfelz, PLoS One, 2015; Toor, Cancer Immunol Immunother, 2017). An elevated N-to-L Ratio (NLR) of NLR > 5, and reduced L-to-M ratio (LMR) of <3.2 are predictive of poor prognosis in cancer patients (pts) (Zhou; Nature, 2017; Sierzega; Ann Surg Onc, 2017). Chemotherapy (chemo) induced neutropenia (CIN) is mitigated with G-CSF such as pegfilgrastim (Peg). Plin is a novel non-G-CSF small molecule, with a different mechanism of action for CIN (LSK inhibition reversal; Lloyd AACR, 2017). Plin (by IV) and Peg (by SC) are given as a single dose-per-cycle. In contrast to Peg, Plin is given on the same day of chemo, 30 minutes after chemo, vs 24 hours after chemo with Peg. Plin does not cause bone pain, and has anti-cancer, immune-enhancing activity (Mohanlal, ASCO-SITC 2018). The Phase (Ph)2 portion of Study BPI-2358-105 (NCT03P171

*Corresponding author email: mbagarazzi@inovio.com6) in NSCLC pts, compared Plin (at different doses; n=55) with Peg for the prevention of Docetaxel (Doc) CIN. Plin (20 mg/m2) and Peg are equally effective for the prevention of Doc CIN, in respect to frequency and duration of severe neutropenia (Blayney, ASCO 2018). Since Plin and Peg both improve BMCs, we evaluated their respective immunosuppressive potential.

**Methods**

BMCs from cycle 1 of Ph2 study 105 was analyzed with either Plin (20 mg/m2; n=14) or Peg (6 mg; n=14). BMCs, including immature Ns ((pro)myelocytes and bands) were available through day (D) 15.

**Results**

In contrast to Peg, Plin did not show NLR>5 or LMR<3 (Table below). N bands were observed in 25% vs 0% of pts with Peg and Plin resp. (Pro)myelocytes were observed in 77% vs 14% of pts with Peg and Plin, resp (p<0.001).

**Conclusions**

Peg, but not Plin generates a BMC profile with a predominant immunosuppressive phenotype, while both are equally effective for the prevention of Doc CIN.

**Table 1 (abstract P495).**
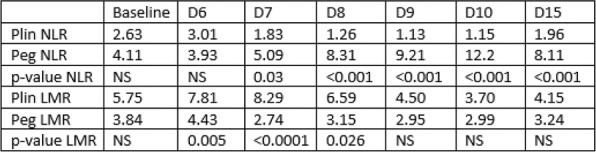
Blood Immune Cell Repertoire after Peg or Plin

#### P496 Innate and adaptive immune responses to metastatic colorectal cancer differ by sex and correlate with survival

##### Anita Ray, PhD^1^, Robert Nofchissey, BS^1^, Sarah Adams, MD^2^, William Berry^1^, Katherine Morris, MD, FACS^3^

###### ^1^OUHSC, Oklahoma City, OK, USA; ^2^UNM, Albuquerque, NM, USA; ^3^University of Oklahoma Stephenson Cancer Center, Oklahoma City, OK, USA

####### **Correspondence:** Katherine Morris (kmorris4@ouhsc.edu)

**Background**

Women with colorectal cancer (CRC) have a survival advantage over men. The mechanism behind this is unclear. CRC is strongly influenced by the tumor immune microenvironment (TME), with multiple immune cell types and signaling pathways implicated in its initiation, progression, and metastasis. Furthermore, murine models of sepsis have demonstrated increased numbers of peritoneal leukocytes and increased activation in females that correlate with improved survival [1,2]. Macrophages are vital participants in the CRC TME and can drive pro- and anti- inflammatory shifts. We hypothesized that the immune CRC TME is sex-dependent and contributes to improved survival in females.

**Methods**

Male and female C57/Bl6 mice were injected with 105 MC38 cells intraperitoneally. Age and sex-matched mice were sacrificed for normal controls (n=6 for all groups). Tumors and ascites developed in the peritoneal cavity in the tumor model. Mice were sacrificed when moribund or at Day 23 post-injection. Serum and tumor cytokine secretion were measured with Luminex™ 32-Plex Immunology assay and results compared by sex. Cells were stained with fluorescent antibodies and run on a flow cytometer alongside controls.

**Results**

Similar to human CRC patients, female mice survived longer than males (p=0.0354). While there were no significant differences in cytokine production in healthy mice, male and female mice bearing tumors had significant differences in both systemic and tumor-produced cytokines. In female TME, more G-CSF, IL-10, and GM-CSF were secreted. These cytokines influence macrophage polarization. Macrophage numbers at the tumor site were similar between the sexes, but tumors from female mice had increased IL-10 producing macrophages (p=0.0685), which is characteristic of M2-type macrophages. Mice with elevated IL-10+ macrophages survived longer (p=0.0005). M2 macrophages and G-CSF signaling cause differentiation of Th2 cells. Tumors from females showed increased total CD4+ T cells (p=0.0398), which also correlated with increased survival (p=0.0222). Animals with elevated IL-4 producing CD4 T cells survived longer (p=0.0195), suggesting that sex-specific innate and adaptive responses to CRC may contribute to the survival benefit seen in women.

**Conclusions**

Shifts within the TME can alter the trajectory of tumor progression and patient outcome. We report here marked differences in the TME of females and males in immune cell populations and behavior. These changes within the tumor are strongly correlated with survival, suggesting that they play a role in the survival gap between men and women with CRC. The origins of these sex-linked responses, and the potential effects on immune therapies warrant further study.

**Acknowledgements**

We would like to acknowledge the American Cancer Society, the Stephenson Cancer Center, and the University of Oklahoma Health Sciences Center Department of Surgery for funding, and the Laboratory for Molecular Biology and Cytometry Research at OUHSC for the use of the Flow Cytometry and Imaging facility which provided equipment and services.

**References**

1. Scotland R, Stables M, Madalli S, et al. Sex differences in resident immune cell phenotype underlie more efficient acute inflammatory responses in female mice. Blood 2011; 118:5918-5927.
2. Angele M, Schwacha M, Ayala A, et al. Effect of gender and sex hormones on immune responses following shock. Shock 2000; 14(2):81-90.

**Ethics Approval**

The study was approved by the OUHSC IACUC, protocol number 17-008-C.

#### P497 Study of anti-HER2 CAR T cells in the immunosuppressive ependymoma tumor microenvironment

##### Marissa Kane^2^, Andrea Griesinger^2^, Andrew Donson^2^, Vladimir Amani^2^, Nicholas Foreman^2^, Davis Witt^3^, Jean Mulcahy Levy^2^, Anandani Nellan, MD^2^

###### ^1^Children's Hospital Colorado, Aurora, CO, USA; ^2^University of Colorado Denver, Aurora, CO, USA; ^3^Thomas Jefferson University, Philadelphia, PA, USA

####### **Correspondence:** Anandani Nellan (anandani.nellan@ucdenver.edu)

**Background**

Ependymoma is the third most common childhood brain tumor and novel treatment methods are urgently needed. Complete surgical resection and radiation still results in a 10-year relapse rate of over 70%. Chemotherapy has failed to improve survival in patients with ependymoma. Chimeric antigen receptor (CAR) T cell therapy has been very effective in hematologic malignancies, but progress in solid tumors has lagged. The hostile tumor microenvironment of solid tumors has been implicated as a primary reason why CAR T cell therapy has only resulted in modest and temporary responses in patients. Previous research has shown that ependymoma tumor cells secrete cytokines that polarize surrounding monocytes into an immunosuppressive phenotype, which in turn renders tumor infiltrating T cells ineffective. Native T cells found in patients’ ependymoma tumor samples are incapacitated and this phenomenon may also affect engineered CAR T cells.

**Methods**

811 and 928 are two high-risk patient derived ependymoma cell lines that have confirmed HER2 surface expression and are used for in-vitro experiments. Human peripheral blood mononuclear cells are activated to promote T cell proliferation and transduced with retrovirus to express anti-HER2 CAR on the surface. Monocytes are cultured in 811 and 928 tumor conditioned media to polarize cells into an immunosuppressive phenotype (polarized monocyte media). T cells and monocytes from the same donor are used in each experiment. Flow cytometry is used to characterize exhaustion markers, as well as surface CAR expression of transduced T cells. Cytokine secretion will be analyzed with a Millliplex Human Cytokine Panel. T cell function will be assessed with an Incucyte live cell imager to quantify immune cell killing of tumor cells over time.

**Results**

Anti-HER2 CAR T cells have excellent pre-clinical efficacy against 811 and 928 cells as demonstrated by cytokine release after co-incubation and robust tumor cell killing. Anti-HER2 CAR T cells co-cultured in 811 and 928 polarized monocyte media exhibit higher numbers of surface inhibitory markers (PD-1, TIM-3, and LAG-3) compared to anti-HER2 CAR T cells cultured in AIMV media. Anti-HER2 CAR T cells co-cultured in 811 or 928 polarized monocyte media also have decreased CAR surface expression and a trend towards decreased tumor cell killing.

**Conclusions**

Anti-HER2 CAR T cells cultured in 811 and 928 polarized monocyte media have increased inhibitory markers and decreased CAR expression. Understanding the mechanism of exhaustion and downregulation of CAR expression may have therapeutic implications to improve the efficacy of CAR T cells against solid tumors.

#### P498 Characterization of the tumor microenvironment in a spontaneous mouse model of cholangiocarinoma: a robust model for evaluating therapeutic interventions for treating the disease

##### Luis Ruffolo, MD, Katherine Jackson, MD, Joseph Murphy, MSc, Nathania Figueroa, MD, Brian Belt, JD, David Linehan, MD, Peter Prieto, MD, MPH

###### University of Rochester Medical Center, Rochester, NY, USA

####### **Correspondence:** Peter Prieto (Apeter_prieto@urmc.rochester.edu)

**Background**

Cholangiocarcinoma is the second most common primary liver malignancy. Prognosis is dismal due to its resistance to conventional therapy and propensity to metastasize. Therefore, the development of effective strategies for treating cholangiocarcinoma represents a significant unmet clinical need, but models for evaluating more advanced approaches like targeted and immune based therapies are lacking. Cholangiocarcinoma is characterized by the presence of a dense fibro-inflammatory reaction and extensive tumor stroma that confers resistance to therapy. Although mice with targeted activation of Kras and loss of p53 (Kras-p53) in the liver spontaneously develop cholangiocarcinoma recapitulating the histological features of human disease, the composition of the tumor microenvironment (TME) remains largely unknown. Here, we examined the composition of the TME in Kras-p53 mice to identify additional targets for evaluating more advanced strategies for treating cholangiocarcinoma.

**Methods**

Histological staining and immunohistochemistry were performed on formalin-fixed, paraffin embedded and frozen tissue sections from human and mouse cholangiocarcinoma tumors for stromal and immune markers. Bone marrow (BM), peripheral blood, spleen, and single cell tumor and normal liver suspensions from Kras-p53 mice and littermate controls were processed for flow cytometry analysis. RNA was extracted from tumor and normal liver tissue and RNA-seq and qRT-PCR analysis were performed. Myeloid cells from BM, spleen, and tumor were isolated and functional assays were performed in-vitro.

**Results**

Human cholangiocarcinoma featured prominent immunosuppressive signatures including a dense inflammatory leukocyte infiltrate mainly comprised of myeloid cells of both monocytic and granulocytic origin including tumor associated macrophages (TAM) and neutrophils (TAN) respectively. Tumors from Kras-p53 mice featured a prominent fibro-inflammatory reaction (Figure 1) with a dense network of fibroblasts, collagen deposition and, hyaluronic acid. Flow cytometry demonstrated Kras-p53 tumors were infiltrated with significantly elevated levels of inflammatory leukocytes (Figure 2) including TAM and TAN. RNA-seq and qRT-PCR demonstrated Kras-p53 tumors expressed elevated levels of cytokines associated with myelopoiesis and mobilization of myeloid cells. In addition, tumors expressed increased levels of soluble factors and checkpoint markers associated with immune suppression including Tgf-β, Il10, Arg-1, Pd-l1, Pd-1, and Ctla-4. Myeloid cells isolated from Kras-p53 tumors were functionally trophic and suppressed T cell proliferation. Thus, these data suggest the TME of cholangiocarcinoma features additional targets for testing more advanced strategies including immune based therapies.

**Conclusions**

Cholangiocarcinoma tumors derived from Kras-p53 mice are highly desmoplastic and feature a prominent inflammatory immune infiltrate including highly immunosuppressive myeloid cells. Thus, Kras-p53 mice are a robust model to evaluate targeted and immune therapeutic interventions.

**Ethics Approval**

The study was approved by the University of Rochester UCAR Committee, approval number 2014-037E

**Fig. 1 (abstract P498).**
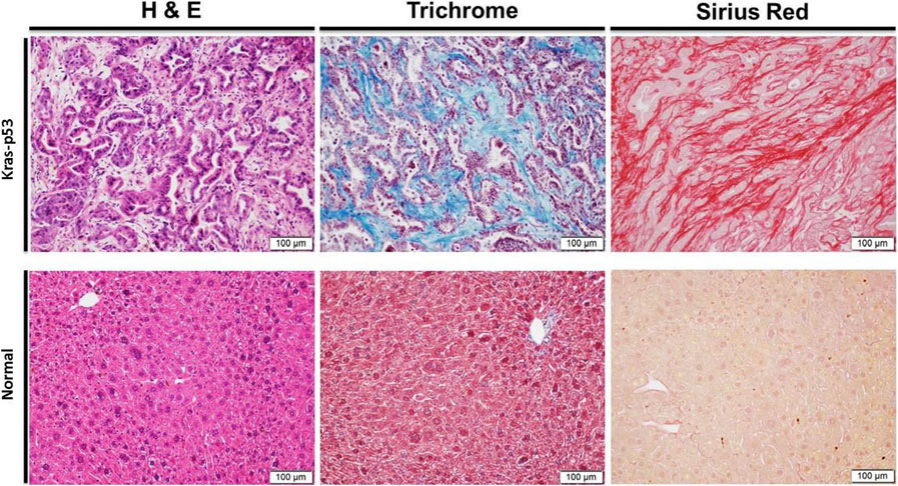
Tumors derived Kras-p53 mice highly desmoplastic

**Fig. 2 (abstract P498).**
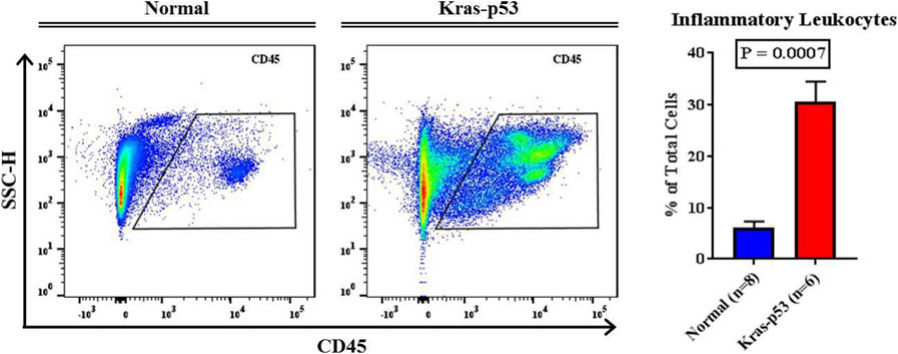
Kras-P53 tumors increased inflammatory leukocytes

#### P499 Correlation of immune escape mediated defects in the HLA class I antigen-presenting machinery with the immune cell infiltration and prognosis in oral squamous cell carcinoma

##### Claudia Wickenhauser, MD, PhD, Barbara Seliger, MD, PhD

###### Luther University Halle-Wittenber, Halle, Germany

####### **Correspondence:** Barbara Seliger (Barbara.Seliger@uk-halle.de)

**Background**

Progression of oral squamous cell carcinoma (OSCC) is often associated with an evasion of tumor cells from the host immune surveillance, which is accompanied by a worse outcome of patients and might influence the efficacy of immunotherapies.

**Methods**

Since little information exist about the molecular mechanisms leading to tumor immune evasion and its correlation with the immune cell contexture the expression of HLA class I antigens and components of the antigen processing machinery (APM) was analyzed in three untreated and interferon (IFN)-γ treated OSCC cell lines as well as a panel of 160 human papilloma virus (HPV)-negative OSCC lesions and correlated with the composition of immune cell infiltration and clinical parameters.

**Results**

Immunohistochemical analyses of the OSCC lesions revealed that HLA class I heavy chain and β2-microglobulin (β2-m) as well as selected HLA class I APM components were significantly downregulated in OSCC lesions vs. non-neoplastic cells. This was in accordance to the predominantly low basal mRNA and protein expression of HLA class I APM components in OSCC cell lines, which was accompanied by low HLA class I surface expression. The impaired HLA class I APM component expression was enhanced by IFN-γ treatment suggesting a deregulation rather than structural alterations as a major mechanism of impaired expression. This was in line with a positive association of HLA class I APM expression levels with the frequency and composition of the immune cell infiltration of OSCC lesions and mediated by the T cell produced IFN-γ: Intra- as well as peri-tumoral density of CD8+ T lymphocytes and intra-tumoral density of FoxP3+ regulatory T cells correlated with membranous HLA class I heavy chain (HC) and β2-m expression and the trimeric HLA classI/β2-m/peptide complex, while membranous β2-m and cytoplasmic HLA class I HC expression positively correlated with the intra-tumoral density of CD4+ T lymphocytes. High cytoplasmic expression levels of HLA class I HC and β2-m, low cytoplasmic expression of the peptide transporter associated with antigen processing (TAP) subunit 1 and a high nuclear expression of LMP2 were significantly correlated with a poor overall survival (OS) of OSCC patients.

**Conclusions**

In conclusion, this study revealed marked differences between HLA class I-positive and negative tumors related to tissue structure, the composition of intra- and peri-tumoral leukocyte infiltration of the TME and OSCC patients’ prognosis. This knowledge might help to overcome immune escape and to improve the efficacy of immunotherapeutic strategies for OSCC patients in the future.

#### P500 Novel bispecific antibody preferentially and efficiently depletes tumor-associated immunosuppressive myeloid cells and enhances therapeutic responses to PD-L1 blockade in immune-excluded tumor model

##### Seng-Lai Tan, PhD, Sangeetha Palakurthi, PhD, Jacqueline Lee

###### Elstar Therapeutics, Cambridge, MA, USA

####### **Correspondence:** Seng-Lai Tan (thomas.tan@elstartherapeutics.com)

**Background**

Accumulation of tumor-associated macrophages (TAMs) and myeloid-derived suppressor cells (MDSCs) has been associated with tumor progression, poor prognosis, and inferior response to immune checkpoint therapy in many cancers. These cells render effector immune cells dysfunctional and promote angiogenesis and metastasis. Thus, TAMs and MDSCs are considered promising therapeutic targets in cancer immunotherapy.

**Methods**

We determined co-expression of CSF-1R and CCR2 on TAMs and MDSCs and generated a bispecific antibody (UniTI-01), which simultaneously binds CSF-1R and CCR2, to deplete these immunosuppressive cell populations, while sparing tissue-resident macrophages. In vitro and ex vivo assays using recombinant proteins, cell lines, and primary cells were conducted to determine UniTI-01 binding to both CSF-1R and CCR2 on the same cell and its effects on CSF-1 and CCL2-dependent functional assays. Plasma exposure, pharmacodynamic and therapeutic effects of UniTI-01 were assessed in murine syngeneic tumor models.

**Results**

We confirmed CSF-1R and CCR2 are co-expressed on TAMs and monocytic MDSCs (M-MDSCs) from ovarian and colorectal cancer patients as well as murine syngeneic tumors. Specific binding of the murine-reactive surrogate bispecific antibody UniTI-01 was demonstrated using murine CSF-1R and/or CCR2 in heterologous expression systems. Additionally, UniTI-01 effectively bound TAMs and M-MDSCs derived from several syngeneic tumor models. The monovalent anti-CSF-1R arm and anti-CCR2 arm of UniTI-01 exerted inhibitory activity in CSF-1- and CCL2-dependent functional assays in vitro, respectively. Importantly, UniTI-01 preferentially depleted TAMs and M-MDSCs over major organ tissue-resident macrophages, including Kupffer cells, in tumor-bearing mice. In contrast, an anti-CSF-1R monoclonal antibody induced significant depletion of tissue-resident macrophages in several organs. UniTI-01 treatment increased intratumoral T cells and CD8+ T cells:CD4+ Treg ratio across different syngeneic tumor models. In the immune-excluded model EMT6, a combination regimen of UniTI-01 and an anti-PD-L1 monoclonal antibody induced significant tumor regression compared to either agent alone. Finally, mice that cleared EMT6 tumor on the combination therapy developed specific and durable anti-tumor response demonstrated by their protection when re-implanted with EMT6 cells, but not with a different tumor cell line (CT- 26).

**Conclusions**

Dual targeting of CSF-1R and CCR2 using a bispecific antibody efficiently depletes TAMs and M-MDSCs without significantly affecting tissue-resident myeloid cells and may serve as a novel approach to enhance therapeutic efficacy of checkpoint blockade immunotherapy with a wider therapeutic window. Our data support development of a synonymous human UniTI-01 for clinical evaluation.

#### P501 Multiplex immunofluorescence evaluation of immune cell relationships within PDAC resection tissues using tailored analysis of multi-spectral image component data

##### Hannah Thomson^1^, Alison Bigley, CSci, FIBMS^2^, Lorcan Sherry, PhD^2^, Mark Anderson, BSc^2^, Mariana Beltran^3^, Dawn Lyster, MSc^3^, Mike Millar^3^

###### ^1^OracleBio Ltd., North Lanarkshire, Scotland, UK; ^2^OracleBio, Glasgow, UK; ^3^Aquila BioMedical, Edinburgh, UK

####### **Correspondence:** Lorcan Sherry (lorcan.sherry@oraclebio.com)

**Background**

Pancreatic ductal adenocarcinoma (PDAC) is an aggressive exocrine tumour with an extremely poor prognosis where the application of checkpoint inhibitors has proven to be disappointing. One of the characteristics of PDAC is a desmoplastic process that is thought to create a barrier to potential responses of immune cells and reduce accessibility of therapeutic agents. PDAC phenotype is also known to be immune cell deficient. However, M2 macrophage aggregations have been identified within the tumour milieu that often co-express programmed cell death markers. The evaluation of PD-L1 expression and associated immune cell responses in Whipple’s resection tissues may be utilised to aid predicting patient outcome [1]. Here we use a 7-plex evaluation to exemplify the potential of multiplex immunofluorescence (mIF) combined with multispectral imaging and quantitative image analysis to examine relationships in immune, inflammatory and checkpoint expressing cell populations within PDAC surgical resection samples.

**Methods**

Exemplar PDAC resection sections were mIF labelled by Aquila BioMedical for 5 cell markers, including PD-L1, CD3, CD8, FoxP3, CD163, a pan cytokeratin epithelial marker and DAPI nuclear marker. The stained slides were digitised using the Vectra Polaris multispectral scanner (Perkin Elmer) and defined region of interest (ROI) images exported in multi-layered component data format. The mIF images were analysed by OracleBio using tailored applications developed in Visiopharm Oncotopix Software. These enabled the identification of tumour and stroma ROI, facilitated cell detection, classification and analysis and the determination of cell relationships within the tumour microenvironment.

**Results**

Across the n=5 resection samples, selected ROI displayed a range of tumour, stroma, lymphoid aggregates and connective tissue content. Analysis of cell populations indicated varying levels of CD3, CD8 and FoxP3 immune cell infiltrations. PD-L1 also showed a varied expression within tumour cells across samples while higher numbers of CD163 positive macrophage aggregations were identified within tumour.

**Conclusions**

Although knowledge of the underlying mechanisms of PDAC have advanced over the recent years, much still remains unclear. Multiplex IF data potentially enables a greater understanding of the complex mechanisms involved in PDAC, thereby furthering the development of drugs that target immune cells and may be indicative of response to treatment or predicting patient outcome.

**References**

1. Yamaki S, Yanagimoto H, Tsuta K, Ryota H, Kon M. PD-L1 expression in pancreatic ductal adenocarcinoma is a poor prognostic factor in patients with high CD8+ tumor-infiltrating lymphocytes: highly sensitive detection using phosphor-integrated dot staining. International Journal of Clinical Oncology. 2017 March 18. 22(4): 726–733.

#### P502 Novel approach of modulating immune cell metabolism in the tumor microenvironment to enhance efficacy of immunotherapy

##### Frank Boriello, MD/PhD^2^, HongBum Lee^3^, Vincent O'Neil^3^, Ted Kim, PhD^3^, James Lederer, PhD^4^, Sanghee Yoo, PhD^3^

###### ^1^ImmunoMet Therapeutics Inc., Houston, TX, USA; ^2^Alloplex Biotherapeutics, Boston, MA, USA; ^3^ImmunoMet Therapeutics, HOUSTON, TX, USA; ^4^Brigham and Women’s Hospital/Harvard, Boston, MA, USA

####### **Correspondence:** James Lederer (jlederer@rics.bwh.harvard.edu)

**Background**

Cells adopt different metabolic strategies depending on their functional needs. Tumor cells deplete glucose by aerobic glycolysis, which can inhibit effector immune cells that may rely on aerobic glycolysis for effector activity [1]. It has been shown that immune cells that use mitochondrial oxidative phosphorylation (OXPHOS) for energy are able to co-exist with tumor cells in the TME. OXPHOS dependent immune cells include CD4+ regulatory T cells (Tregs), myeloid-derived suppressor cells (MDSC), and tumor associated macrophages (TAM). These immune cell types are immune suppressive and metabolically compatible with tumor cells [2].

**Methods**

Human PBMC was used for immune suppressive cell differentiationCyTOF mass cytometry was used to characterize immune cellsSyngeneic in vivo animal studies using RENCA and CT26 were conducted for in vivo efficacy studies

**Results**

IM188 is an OXPHOS inhibitor drug with a biguanide core structure. Metformin is the canonical biguanide drug that has been safely used to control glucose levels in people with type II diabetes. The mechanisms for how biguanide drugs influence immune cells has not been well characterized. Since IM188 is an optimized biguanide targeting OXPHOS dependent immune cells, we studied the effects of IM188 on human blood immune cells (PBMCs) and on immune responses in mouse models of infection or cancer. PBMCs were differentiated under conditions to promote Treg or MDSC expansion in vitro in the absence or presence of IM188. Analysis of differentiated T cells by CyTOF mass cytometry showed reduced expression of multiple Treg markers such as Foxp3, CTLA4, and TGF-beta. In MDSC differentiation studies, we found that IM188 reduced MDSC expansion and their functional activity to suppress T cell proliferation. In mouse bacteria and virus infection studies, the most intriguing finding was the IM188 treatment caused increased CD8+ T cell expansion and increased IFN-gamma and TNF-alpha cytokine expression in CD8+ T cells. These observations suggest that IM188 can enhance T cell mediated immune responses. Finally, in syngeneic mouse tumor models, IM188 showed a good range of combination efficacy with anti-PD1 therapy. We measured increased T effector cells and decreased immune suppressive cell types at the tumor site in mice treated with IM188 or anti-PD-1 antibody.

**Conclusions**

In summary, IM188 shows metabolic reprogramming activity that may enhance immune functions by modulating immune cell differentiation and/or function by inhibiting OXPHOS-dependent cells and promoting aerobic glycolysis by effector immune cells.

**References**

1. Chang HA, Qiu J, O’Sullivan D, Buck MD, Noguchi T, Curtis JD, Chen Q, Gindin M, Gubin, MM, van der Wind GWJ, Tonc E, Schreiber,RD, Pearce EJ, and Pearce EL. Metabolic competition in the tumor microenvironment is a driver of cancer progression. 2015 ; 162:1229-1241.
2. Hossain F, Al-Khami AA, Wyczechowska D, Hermandez C, Zheng L, Reiss K, Valle LD, Trillo-Tinoco J, Maj T, Zou W, Rodriguez PC, Ochoa AC. Inhibition of fatty acid oxidation dodulates immunosuppressive functions of myeloid-derived suppressor cells and enhances cancer therapies. Cancer Immunol Res. 2015; 2:1236-127.

#### P503 Characterization of the immune desert in metastatic non-small cell lung cancer (NSCLC) and the use of cell proliferation to predict clinical response to immune checkpoint inhibitors (ICIs)

##### Jason Zhu, MD^1^, Matthew Labriola, MD^1^, Daniele Marin, MD^1^, Shannon McCall, MD^1^, Edwin Yau, MD, PhD^2^, Grace Dy^2^, Sarabjot Pabla, MSc, PhD, BS^3^, Sean Glenn, PhD^3^, Carl Morrison, MD, DVM^3^, Daniel George, MD^1^, Tian Zhang, MD^1^, Jeffrey Clarke, MD^1^

###### ^1^Duke University, Durham, NC, USA; ^2^University of Buffalo, Buffalo, NY, USA; ^3^Omniseq Inc, Buffalo, NY, USA

####### **Correspondence:** Tian Zhang (tian.zhang2@duke.edu)

**Background**

Immune checkpoint inhibitors (ICIs) have profoundly reshaped the treatment landscape for advanced NSCLC. While patients who have an ICI response may have a deep and durable response, in unselected populations, the majority of patients will not respond, but will be exposed to potential immune toxicities. Current biomarkers such as PD-L1 are not sensitive nor specific for predicting response, particularly for non-inflamed (NI) or “immune desert” tumor microenvironments. Here, we describe the use of cell proliferation to evaluate response in immune desert NSCLC.

**Methods**

113 formalin-fixed, paraffin-embedded (FFPE) tumor samples of metastatic NSCLC were evaluated by RNA-sequencing to measure transcript levels of genes related to tumor infiltrating lymphocytes and cell proliferation, DNA-sequencing of 409 genes for tumor mutational burden (TMB), and PD-L1 status (Dako 22C3 antibody assay). Tumors were defined as inflamed or NI based upon RNA-sequencing analysis of CD8 compared to a reference population of more than 500 cases of multiple tumors. NI/immune desert tumors were defined as the lower 15th percentile of rank for CD8+ T-cells. Cell proliferation, defined as the mRNA expression of 10 genes (BUB1, CCNB2, CDK1, CDKN3, FOXM1, KIAA0101, MAD2L1, MELK, MKI67, TOP2A) was evaluated for association with PD-L1 IHC expression, TMB, and response to ICIs by RECIST 1.1 criteria.

**Results**

In our cohort of 113 cases, 12% (15/113) were classified as having an NI, immune desert tumor microenvironment. Of these 15 NI cases, 8 (53%) were proliferative and 7 (47%) were non-proliferative; in addition, 10 of 15 cases (67%) had high TMB (TMB-H). The majority (11/15, 73%) of NI cases were non-responders, with 4 cases having an objective response. For the 4 responders, 3 were non-proliferative; conversely, for the 11 nonresponders,7 were proliferative. For NI/TMB-H there were 9 were nonresponders, with 7 proliferative and 1 responder who was non- proliferative. For NI/TMB-L the ORR was 60% (3/5) with 2 of 3 responders being non-proliferative. Only 2 of these NI cases were PD-L1 positive (TPS>50%), one of which as a non-proliferative responder (50%). PD-L1 was negative in the other 13 cases, 3 of whom were responders (23%).

**Conclusions**

Biomarkers such as PD-L1 and TMB on their own may not be sufficient in predicting overall response in immune desert tumor microenvironments. In these cases, cell proliferation may be important in distinguishing patients who may have a clinical benefit to ICI. These results support that NI non-proliferative tumors are more likely to respond to ICI than NI proliferative tumors.

**Ethics Approval**

OmniSeq’s analysis utilized deidentified data that qualified as non-human subject research under IRB-approved protocols, approved by both Roswell Park Comprehensive Cancer Center (Buffalo, NY, BDR #080316) and Duke Cancer Institute (Durham, NC, PRO00088762).

### Impact of Diet, Exercise, and/or Stress on Antitumor Immunity

#### P504 Nutritional measures to boost immunosurveillance of breast cancer by NK cells

##### Lorenzo Galluzzi, PhD^1^, Aitziber Buqué, PhD^1^, Maria Perez-Lanzon, MSc (Master of Science)^2^, Takahiro Yamazaki, PhD^1^, Guido Kroemer, MD, PhD^2^

###### ^1^Weill Cornell Medical College, New York, NY, USA; ^2^Centre de Recherche des Cordeliers, Paris, France

####### **Correspondence:** Guido Kroemer (kroemer@orange.fr)

**Background**

Hormone receptor (HR+) breast cancer (BC) is currently responsible for the majority of BC-related deaths in the US [1]. HR+ BC patients are usually managed by surgery, followed by adjuvant endocrine therapy ± chemotherapy. However, chemotherapy provides limited clinical benefits and is often associated with severe side effects[2], calling for the development of alternative treatment regimens. Over the past decade, immunotherapy with immune checkpoint blockers (ICBs) has achieved unprecedented clinical success in patients with a variety of tumors [3]. However, despite the fact that BC is also under immunosurveillance [4,5], BC patients are generally resistant to ICBs [6], especially in the case of HR+ disease [7]. Thus, there is an unmet need for improved therapeutic approaches to HR+ BC, at least in part reflecting the lack of adequate preclinical models to recapitulate the incidence, natural progression, metastatic dissemination and response to therapy of HR+ BC in immunologically competent hosts.

**Methods**

We extensively characterized endogenous BC driven in wild-type or genetically engineered C57BL/6 or BALB/c mice by slow-release medroxyprogesterone acetate (MPA) pellets and oral 7,12-dimethylbenz[a]anthracene (DMBA) for incidence, progression, histology, transcriptomic profile, and sensitivity to standard therapeutic agents as well as nutritional interventions.

**Results**

We demonstrate that MPA/DMBA-driven tumors resemble human HR+ BC in that (1) they display a similar morphology, (2) they express hormonal receptors, (3) they have a gene signature that largely overlaps with that of HR+/HER2- human BCs, (4) tumorigenesis depend on nuclear estrogen receptors, (5) tumor insurgence can initially be delayed by tamoxifen administration, but acquired resistance rapidly subsides, (6) they are under active immunosurveillance by the host immune system with a predominant role for NK cells, and once they form palpable nodules they exhibit limited immune infiltration, and (7) they develop according to rather heterogeneous kinetics. Moreover, MPA/DMBA-driven tumors resemble human HR+ BC because they respond to chemotherapy, PD-1 blockade and RT in a rather heterogeneous and poor fashion. We demonstrate that MPA/DMBA-driven carcinogenesis can be delayed by caloric restriction as well as administration of vitamin B6, vitamin D, and nicotinamide mononucleotide (NAM), in immunocompentent, but not immunodeficient, mice, an effect paralleled by increased amounts of NK cells in the spleen.

**Conclusions**

HR+ BC appears to evolve by evading NK cell-dependent immunosurveillance, suggesting that NK cell-activating strategies, including nutritional measures like NAM, as well as specific antibodies targeting NK cell receptors, may improve the efficacy of (immuno)therapeutic agents currently employed in the clinics for HR+ BC patients.

**References**

1. Munoz D, et al. Effects of screening and systemic adjuvant therapy on ER-specific US breast cancer mortality. J Natl Cancer Inst. 2014;106. 2. Jagsi R, et al. Impact of adjuvant chemotherapy on long-term employment of survivors of early-stage breast cancer. Cancer. 2014;120:1854-1862. 3. Sharma P, Allison JP. The future of immune checkpoint therapy. Science.2015;348:56-61. 4. Savas P, et al. Clinical relevance of host immunity in breast cancer: from TILs to the clinic. Nat Rev Clin Oncol. 2016;13:228-241. 5. Kroemer G, Senovilla L, Galluzzi L, Andre F, Zitvogel L. Natural and therapy-induced immunosurveillance in breast cancer. Nat Med. 2015;21:1128-1138. 6. Emens LA. Breast cancer immunotherapy: facts and hopes. Clin Cancer Res. 2018;24:511-520. 7. Rugo HS, et al. Safety and antitumor activity of pembrolizumab in patients with estrogen receptor-positive/human epidermal growth factor receptor 2-negative advanced breast cancer. Clin Cancer Res. 2018.

**Ethics Approval**

The study was approved by IACUC at the hosting institutions

#### P505 The gut microbiome of metastatic melanoma patients initiating systemic therapy is influenced by host factors including diet, probiotic and antibiotic use

##### Vancheswaran Gopalakrishnan, MPH, PhD^1^, Christine Spencer, PhD^2^, Jennifer McQuade, MD^1^, Miles Andrews, MD, PhD^1^, Beth Helmink, MD PhD^1^, Alexandria Cogdill, MEng^1^, Md Khan^1^, Elizabeth Sirmans^1^, Lauren Haydu, MS, BChe, MIPH^1^, Eliza Posada^1^, Elizabeth Burton^1^, Isabella Glitza, MD, PhD^1^, Rodabe Amaria, MD^1^, Sapna Patel, MD^1^, Adi Diab, MD^1^, Michael Wong, MD PhD FRCPC^1^, Hussein Tawbi, MD, PhD^1^, Wen-Jen Hwu, MD, PhD^1^, Michael Davies, MD, PhD^1^, Patrick Hwu, MD^1^, Robert Jenq, MD^1^, Kelly Nelson, MD^1^, Carrie Daniel- MacDougall, MPH, PhD^1^, Lorenzo Cohen^1^, Jennifer Wargo, MD, MMSc^1^

###### ^1^UT MD Anderson Cancer Center, Houston, TX, USA; ^2^Parker Institute for Cancer Immunotherapy, New York, NY, USA

####### **Correspondence:** Jennifer Wargo (JWargo@mdanderson.org)

**Background**

The diversity and composition of the gut microbiome has been implicated in differential responses to immune checkpoint blockade in melanoma and other cancers [1-3]. However, little is known about the impact of diet and other lifestyle factors in this population.

**Methods**

We assembled a large cohort of early and late-stage melanoma patients (n=312) initiating systemic treatment at UT MD Anderson Cancer Center. In addition to biological specimens, we collected a comprehensive lifestyle survey, including the NCI dietary screener questionnaire, in a subset of patients (n=113). The fecal microbiome was characterized via sequencing of the V4 region of the 16S rRNA gene to determine diversity and compositional structure. Dietary components were dichotomized into high and low categories based on the median of estimated consumption. Differences in compositional structure between groups was determined using analysis of similarity (ANOSIM) for unweighted UniFrac beta diversity distances, and pairwise Mann-Whitney tests for taxonomic comparisons.

**Results**

The median age of melanoma patients in our cohort was 62 yrs (59% male; 86% Stage III/IV), and the most common treatment type was anti-PD1 based therapy (53.1%). There were no significant associations observed between alpha diversity and age, sex or body mass index among the melanoma patients. “Biotic” use, defined as self-reported use of either biotic was quite common (29% antibiotics, 42% probiotics), and was associated with lower alpha-diversity (p=0.01), with significant associations observed for both antibiotics alone (p=0.05) and for probiotics alone (p=0.02). Additionally, consumption of red meat (p=0.006), sugar-sweetened beverages (SSB) (p=0.048), and fruits and vegetables (FV) (p=.049) were also associated with differences in compositional structure with selective enrichment of Desulfovibrionales (high vs. low red meat =0.03), Mollicutes (in low vs. high SSB consumers, p=0.008), and Porphyromonadaceae (in high vs low FV consumers, p=0.001).

**Conclusions**

Prospective longitudinal studies are underway to assess the relationships between “biotic” use, dietary factors and the gut microbiome, and treatment response among patients, as well as functional studies in preclinical models. These data provide preliminary evidence that the gut microbiome of melanoma patients may be modifiable by host factors such as diet, use of antibiotics and probiotics, with potential therapeutic implications.

**References**

1. Gopalakrishnan V, et al. Gut microbiome modulates response to anti–PD-1 immunotherapy in melanoma patients. Science. 2018; 359(6371): 97-103.
2. Routy B, et al. Gut microbiome influences efficacy of PD-1–based immunotherapy against epithelial tumors. Science. 2018; 359(6371): 91-97.
3. Matson V, et al. The commensal microbiome is associated with anti–PD-1 efficacy in metastatic melanoma patients. Science. 2018; 359(6371): 104-108.

**Ethics Approval**

The study was approved by The University of Texas MD Anderson Center's Ethics Board, approval numbers LAB00-063, and PA15-0232

#### P506 Subcutaneous and intramuscular fat indices predict survival in advanced stage cancer patients treated with immunotherapy

##### Dylan Martini, BA^1^, Julie Shabto, BA^1^, Yuan Liu, PhD^2^, Milton Williams^1^, Amir Khan^1^, Colleen Lewis^3^, Hannah Collins^3^, Mehmet Akce^3^, Haydn Kissick^1^, Bradley Carthon, MD, PhD^3^, Walid Shaib, MD^3^, Olatunji Alese, MD^3^, Conor Steuer, MD^3^, Christina Wu, MD^3^, David Lawson, MD^3^, Ragini Kudchadkar, MD^3^, Bassel El-rayes, MD^3^, Suresh Ramalingam, MD^3^, Taofeek Owonikoko, MD, PhD^3^, R. Donald Harvey, PharmD^1^, Viraj Master, MD, PhD^1^, Mehmet Bilen, MD^3^

###### ^1^Emory University School of Medicine, Atlanta, GA, USA; ^2^Emory University, Atlanta, GA, USA; ^3^Winship Cancer Institute, Atlanta, GA, USA

####### **Correspondence:** Mehmet Bilen (mehmet.a.bilen@emory.edu)

**Background**

Obesity has been investigated as a prognostic indicator in patients with cancer [1]. In this study, we explored the association between different types of fat and clinical outcomes in advanced stage cancer patients treated with immunotherapy by developing a risk group classification.

**Methods**

We performed a retrospective analysis of 90 patients treated on immunotherapy-based phase 1 clinical trials at our center from 2009-2017. Baseline CT images at mid-L3 were obtained, and subcutaneous fat density, intramuscular fat density, and visceral fat density (cm2) were calculated using SliceOmatic (TomoVision, version 5.0) and converted to indices (SFI: subcutaneous fat index, IFI: intramuscular fat index, and VFI: visceral fat index) after dividing by height in meters-squared. Risk groups by PFS were created by a recursive partitioning and regression trees method for SFI and IFI, which were selected by a stepwise variable selection among all fat related variables (Figure 1). Cox proportional hazard model and Kaplan-Meier method were used for association with OS and PFS.

**Results**

Most patients (59%) were males and more than two-thirds (69%) had at least 2 prior lines of therapy. Melanoma (33%) and gastrointestinal (22%) tumors were the most common histologies. The medians for each of the indices were as follows: SFI = 62.78, IFI = 4.06, and VFI = 40.53. Low-risk patients (SFI 73) had significantly longer OS and PFS than intermediate-risk (SFI < 73 and IFI < 3.4) and poor-risk patients (SFI < 73 and IFI 3.4) (Table 1). Intermediate-risk patients also trended towards longer OS and PFS than poor-risk patients. Patients in the low-risk group had substantially longer median OS and PFS than intermediate and poor-risk patients per Kaplan-Meier estimation (Table 1, Figures 2-3).

**Conclusions**

Decreased subcutaneous fat and increased myosteatosis may decrease survival in advanced stage patients treated with immunotherapy. Future studies should investigate the interaction between different fat composition, the immune system, and the tumor microenvironment.

**References**

1. Demark-Wahnefried W, Platz EA, Ligibel JA, Blair, CK Courneya KS, Meyerhardt JA, et al. The Role of Obesity in Cancer Survival and Recurrence. Cancer Epidemiology, Biomarkers, and Prevention. 2012 Aug;21(8):1244-59.

**Ethics Approval**

The study was approved by the Emory University Institutional Review Board, approval number IRB00100973.

**Fig. 1 (abstract P506).**
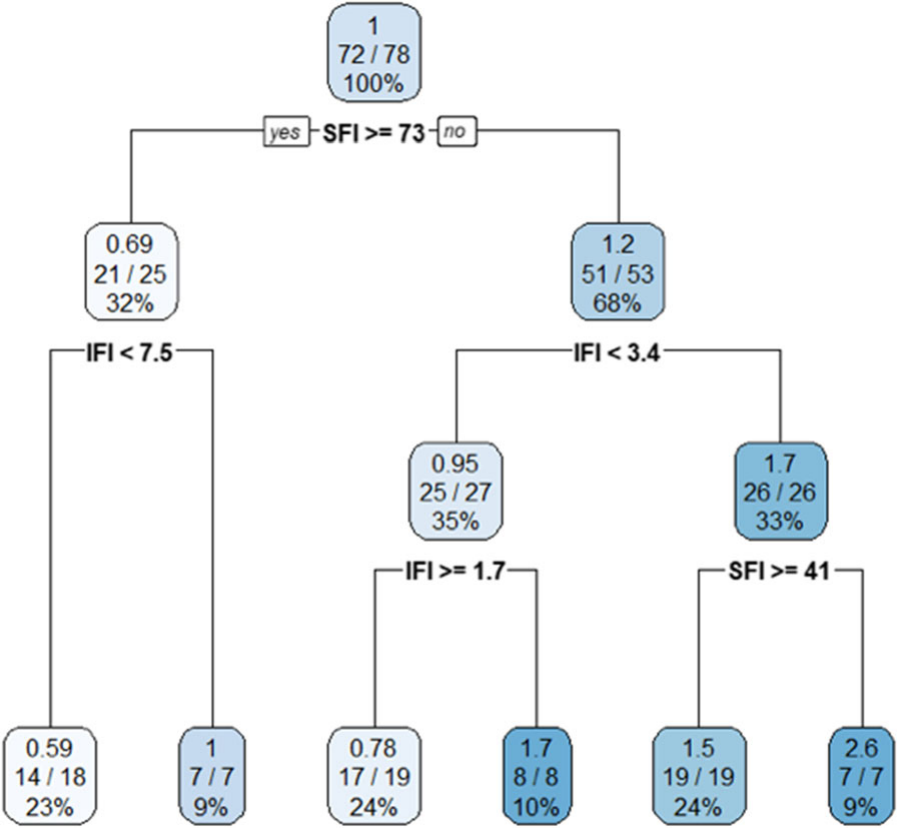
See text for description

**Table 1 (abstract P506).**
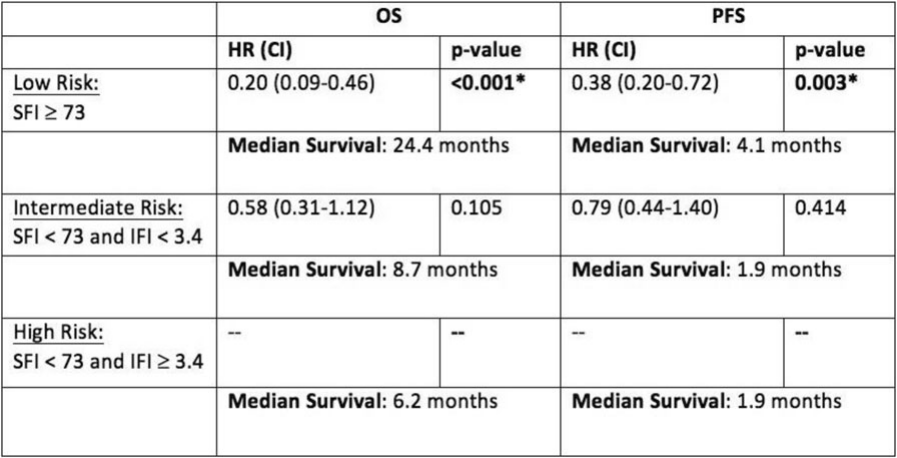
MVA† of fat risk with survival

**Fig. 2 (abstract P506).**
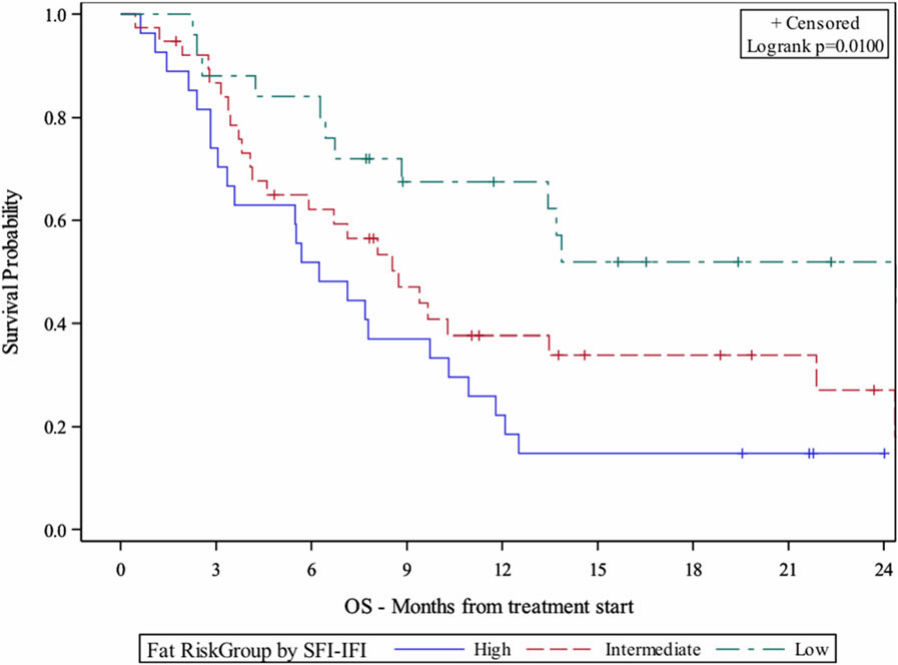
See text for description

**Fig. 3 (abstract P506).**
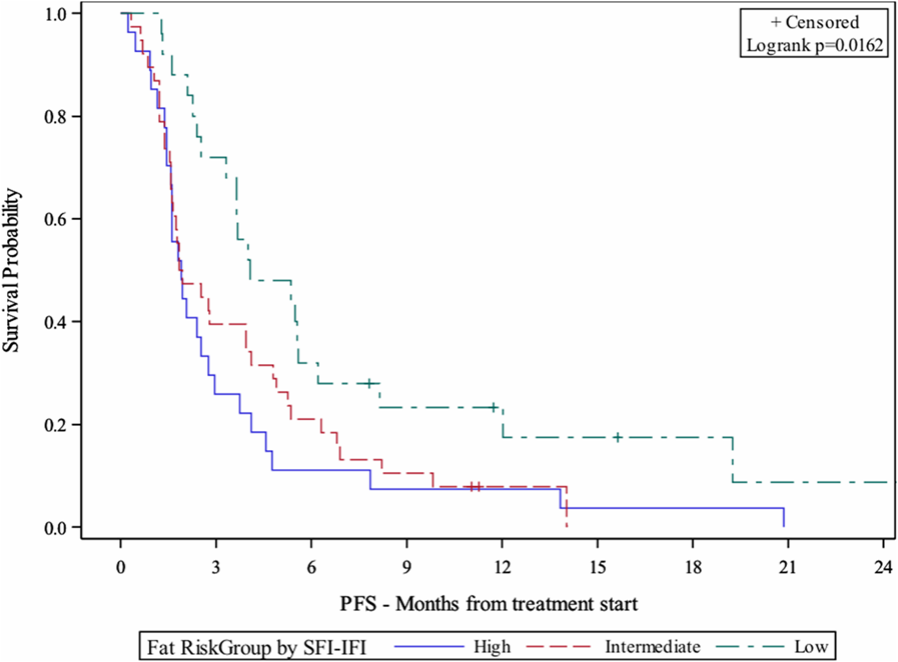
See text for description

#### P507 Body mass index as a predictor of survival in advanced stage cancer patients treated with immunotherapy

##### Julie Shabto, BA^1^, Dylan Martini, BA^1^, Yuan Liu, PhD^2^, Milton Williams^1^, Amir Khan^1^, Colleen Lewis^3^, Hannah Collins^3^, Mehmet Akce^2^, Haydn Kissick^2^, Bradley Carthon, MD, PhD^2^, Walid Shaib, MD^2^, Olatunji Alese, MD^2^, Conor Steuer, MD^2^, Christina Wu, MD^2^, David Lawson, MD^2^, Ragini Kudchadkar, MD^2^, Bassel El-rayes, MD^2^, Suresh Ramalingam, MD^2^, Viraj Master, MD, PhD^2^, Taofeek Owonikoko, MD, PhD^2^, R. Donald Harvey, PharmD^2^, Mehmet Bilen, MD^2^

###### ^1^Emory University School of Medicine, Atlanta, GA, USA; ^2^Emory University, Atlanta, GA, USA; ^3^Winship Cancer Institute, Atlanta, GA, USA

####### **Correspondence:** Mehmet Bilen (mehmet.a.bilen@emory.edu)

**Background**

Body mass index (BMI) has been investigated as a prognostic factor for cancer patients [1], but the effect of BMI on clinical outcomes in patients on phase 1 clinical trials using immunotherapy-based treatment is not known. We investigated the association between BMI and survival in advanced staged cancer patients treated with immunotherapy.

**Methods**

We completed a retrospective analysis of 90 patients treated on phase 1 clinical trials using immunotherapy-based treatment regimens at Winship Cancer Institute of Emory University from 2009-2017. Baseline BMI was collected from the electronic medical records. Overall survival (OS) and progression-free survival (PFS) were measured from the first dose of immunotherapy to date of death or hospice referral and radiographic or clinical progression, respectively. Cox proportional hazard model was used for association with OS and PFS. BMI was analyzed as a continuous variable and as a categorical variable (normal or underweight: BMI < 25, overweight: 25 ≤ BMI < 30, obese: BMI ≥ 30).

**Results**

Most patients (59%) were males and the majority (81%) were Royal Marsden Hospital (RMH) good risk. Approximately two-thirds (69%) of patients received at least 2 lines of systemic therapy before being treated with immunotherapy on the clinical trial. Melanoma (33%), gastrointestinal (22%), and lung and head & neck (20%) were the most common tumor types. The median BMI was 27.4. When treated as a continuous variable in multi- variable analysis, a higher BMI was significantly associated with longer OS and PFS (Table 1). Patients with a normal or underweight BMI had significantly shorter OS (HR: 3.27, p-value: 0.005) and trended towards shorter PFS when compared to overweight and obese patients. The median OS and PFS of obese patients was 19.1 months and 4.7 months, respectively, while median OS and PFS of normal or underweight patients was 6.7 months and 1.9 months and median OS and PFS for overweight patients was 8.6 months and 2.3 months, respectively, per Kaplan- Meier estimation (Table 1, Figures 1-2).

**Conclusions**

Obesity may help prolong survival in advanced stage cancer patients treated with immunotherapy. Further studies are needed to elucidate the underlying biologic effect of adiposity on the tumor microenvironment and the immune system in patients treated with immunotherapy.

**References**

1. Azvolinsky A. Cancer Prognosis: Role of BMI and Fat Tissue. JNCI. 2014; Volume 106: page 6-7.

**Ethics Approval**

The study was approved by the Emory University Institutional Review Board, approval number IRB00100973.

**Table 1 (abstract P507).**
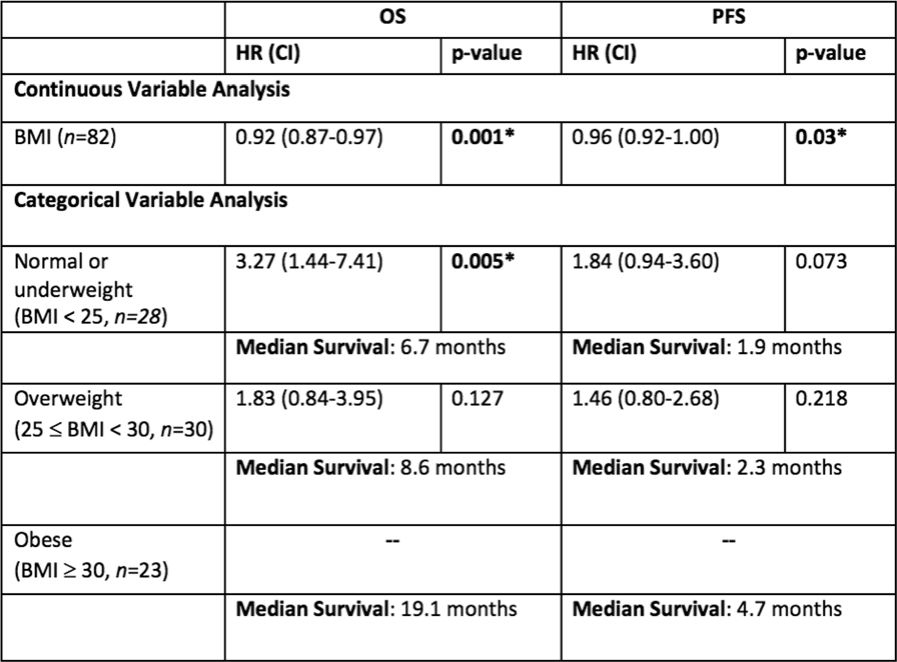
MVA† of the association between BMI and survival

**Fig. 1 (abstract P507).**
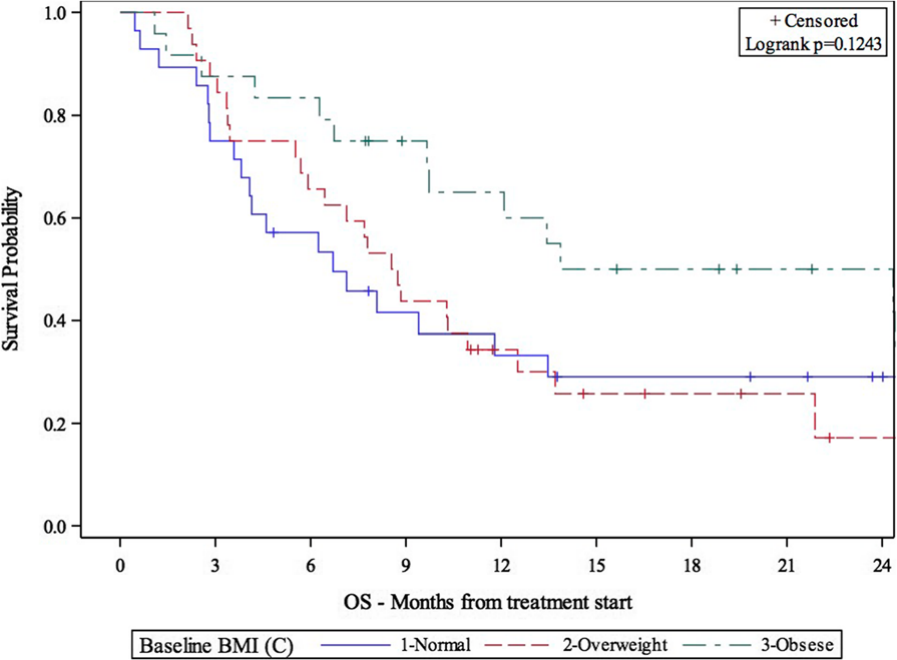
See text for description

**Fig. 2 (abstract P507).**
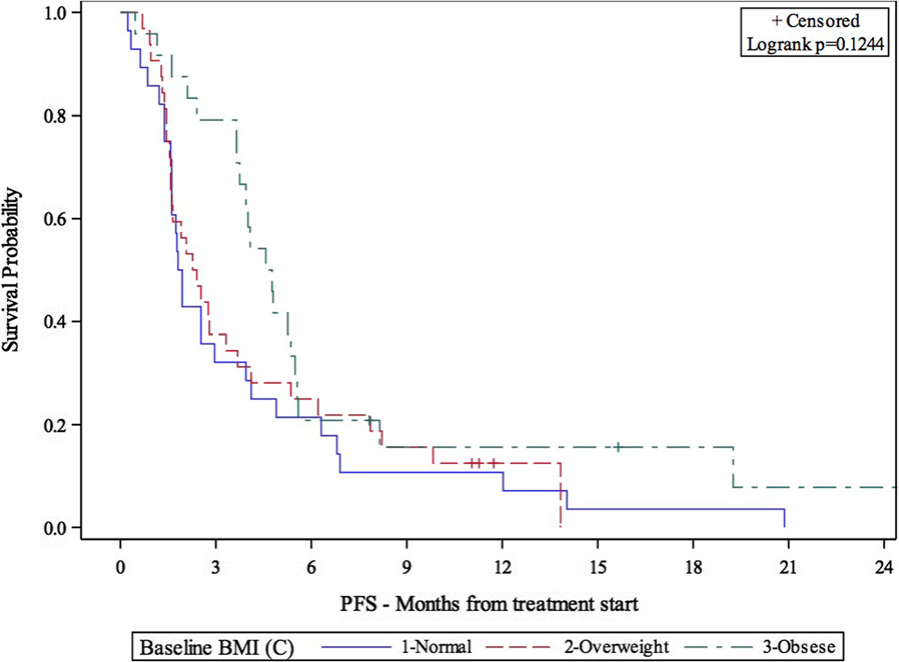
See text for description

#### P508 Obesity promotes PD-1 mediated T cell dysfunction and tumor pro-gression but superior anti-tumor effects upon checkpoint blockade

##### Ziming Wang, MS, Jesus Luna, PhD, Cordelia Dunai, MS, Lam Khuat, Catherine Le, BS, Ethan Aguilar, Annie Mirsoian, Christine Minnar, PhD, Kevin Stoffel, MS, Ian Sturgill, Steven Grossenbacher, Robert Canter, MD, MAS, FACS, Arta Monjazeb, MD, PhD, William Murphy, PhD, Ziming Wang, MS

###### University of California, Davis, Sacramento, CA, USA

####### **Correspondence:** William Murphy (wmjmurphy@ucdavis.edu)

**Background**

PD-(L)1 signaling is central to T cell exhaustion which occurs with chronic antigen stimulation and results in T cell dysfunction. Blockade of the PD-(L)1 pathway augments T cell responses in a variety of viral and cancer models. Obesity, defined by high body mass index (BMI >30 kg/m2), is reaching pandemic proportions and is a major cancer risk factor. The impact of obesity on immune responses in general, and cancer immunotherapy in particular, is poorly understood.

**Methods**

Male B6 and female BALB/c mice were fed diets consisting of either 60% or 10% fat, respec-tively, starting from 6-week until 6-month old. DIO and control mice were injected with either B16-F0 (non-metastatic melanoma), B16- F10 (metastatic melanoma), 3LL (metastatic Lewis lung carcinoma), or 4T1 (metastatic breast carcinoma) cells. Tumor-bearing mice were treated intraperitoneally with aPD-1 mAb every other day at 250μg/mouse after an initial dose of 500μg/mouse for a total of 6 injections. Tumor progression was determined by caliber measure-ment, PET- CT, and quantification of metastases. Immune phenotypes and T cell function were measured by flow cytometry. Transcriptomes were analyzed by RNAseq.

**Results**

DIO mice were significantly heavier than control mice, with an average weight of 60g vs 42g in B6 mice, and 40g vs 20g in BALB/c mice. Tumors grew significantly faster in DIO mice com-pared to control counterparts as quantified by caliber measurement and PET-CT. T cells in the tumor microenvironment (TME) of DIO mice demonstrated features of exhaustion, including significantly increased expression of PD-1, Tim3 and Lag3, but decreased expression of Ki67. Transcriptomic analysis of sorted (>95% purity) CD8+ memory T cells from B16- bearing control and DIO mice also demonstrated the upregulation of exhaustion-related transcripts and down- regulation of effector-related transcripts in T cells from DIO mice. aPD-1 treatment led to signifi-cant reduction of tumor burden, inhibited development of metastases in DIO mice, and overall improved survival times. The enhanced checkpoint blockade responsiveness in DIO mice was associated with significantly increased CD8+ tumor- infiltrating lymphocytes (TILs), as well as increased TNFα and IFNγ-production by CD8+ T cells.

**Conclusions**

These data indicate a paradoxical impact of obesity on cancer in which immune dysfunction, and tumor progression are heightened, but checkpoint blockade efficacy is also enhanced. This study highlights obesity as a biomarker for cancer immunotherapy.

### Innate Anti-Tumor Immunity

#### P509 Tumor spheroid model to dissect the interplay between myeloid cells and cancer cells

##### Elaheh Ahmadzadeh, PhD, Jan Martinek, PhD, Florentina Marches, Chun Yu, PhD, A. Karolina Palucka, MD

###### Jackson Laboratory, Farmington, CT, USA

####### **Correspondence:** A. Karolina Palucka (karolina.palucka@jax.org)

**Background**

The tumor microenvironment includes cancer cells as well as stromal cells, immune cells, epithelial cells, vasculature and extracellular matrix. Interactions between these components regulate tumor development, migration and metastasis. Current 2-dimensional (2D) in vitro culture methods fail to represent the multidimensional complexity of tumor microenvironment. The multicellular tumor spheroids support co-culture conditions and allow a 3D system that can be employed to investigate the integration of multiple cell types and gain insight into the interaction of cancer cells with other cells in their environment. We established such model to investigate the molecular mechanisms regulating the interactions between melanoma, myeloid cells and T cells.

**Methods**

Tumor Spheroids were developed using hanging drop technique with multiple melanoma cell lines, dermal fibroblasts and immune cells. Preliminary experiments were carried out to determine the optimum conditions for spheroid formation. Spheroids were cultured at density of 104 per cell type per drop and incubated for 3, 4, or 5 days.

**Results**

Spheroid formation occurred within 72 hours and the integrity of spheroids were maintained throughout the experiments. Cell viability and cell proliferation was monitored within spheroids for up to 5 days using Live and dead staining and CellTrace™ CFSE Cell Proliferation Kit, respectively. Immunofluorescence analysis of spheroid cryosections showed a homogeneous distribution of fibroblasts in spheroids. However, spheroid compaction and fibronectin expression varied between spheroids formed by different cell lines. We assessed the integration and localization of monocytes within the spheroids by adding purified blood CD14+ cells to the mixture of tumor cells and dermal fibroblasts at equal density of 104 cells per drop. Immunofluorescence analysis of serial sections of spheroid showed CD45+ cells scattered throughout spheroid. Furthermore, addition of CD14+ cells to tumor spheroids on day 3 resulted in infiltration of monocytes into spheroids in less than 24 hours.

**Conclusions**

Thus, our 3D model can be used to assess the distribution of immune infiltrates and the interaction of cancer cells with myeloid cells.

#### P510 Monoclonal antibodies targeting the MerTK receptor de-repress the innate immune response

##### Diego Alvarado, PhD, Mike Murphy, Laura Vitale, BS, Thomas O'Neill, BA, Andrew Proffitt, Jay Lillquist, Gwenda Ligon, Craig Polson, Anna Wasiuk, Jeffrey Weidlick, BS, Jenifer Widger, BA, Laura Mills-Chen, Andrea Crocker, BS, Colleen Patterson, Russell Hammond, Li-Zhen He, MD, Joel Goldstein, PhD, Lawrence Thomas, PhD, Henry Marsh, BS PhD, Tibor Keler, PhD, Richard Gedrich

###### Celldex Therapeutics, New Haven, CT, USA

####### **Correspondence:** Diego Alvarado (dalvarad.da@gmail.com)

**Background**

MerTK, a member of the TAM (Tyro3/Axl/MerTK) family of receptor tyrosine kinases (RTKs), is an important negative regulator of innate immunity. Activation of MerTK in myeloid cells by its ligands Gas6 or Protein S (PROS) promotes phosphatidylserine-dependent efferocytosis of apoptotic cells, inducing a tolerogenic state and mediating resolution of inflammation. MerTK deficient mice exhibit phenotypes consistent with systemic inflammation and auto-immunity. Importantly, MerTK ablation confers tumor immunity, increased pro- inflammatory cytokines and tumor lymphocyte infiltration. We hypothesize that pharmacological targeting of MerTK with monoclonal antibodies (mAbs) may lead to a similar pro-inflammatory response and recapitulate the anti-tumor effects observed in MerTK-/- mice.

**Methods**

Cultured human PBMCs, or monocyte-derived dendritic cells and macrophages were treated with a panel of MerTK-targeting mAbs for 24 hours alone, or in the presence of pro-inflammatory stimuli (LPS, CD40L or IFN-gamma). A panel of cytokines (eg. IL-1RA, TNF-a) were measured in supernatants by ELISA, or using a multiplex approach. Phospho-immunoreceptor (R&D) profiling from PBMC lysates was performed after treatment with mAbs or saline for 1 hour in full serum. The generation and characterization of MerTK knock-out and human MerTK transgenic mice will be presented.

**Results**

From a panel of human anti-MerTK mAbs derived from phage-display libraries or human IgG mice we identified several mAbs that enhanced cytokine and chemokine release from primary human immune cells, alone or in the presence of inflammatory stimuli. Surprisingly, mAb activity required Fc receptor binding as introduction of mutations in the Fc domain to abrogate FcR binding rendered the Abs ineffective, despite maintaining target binding. Surrogate mAbs targeting mouse MerTK elicited similar responses in vivo. Treatment of human PBMCs with a MerTK mAb resulted in reduced phosphorylation of ITIM-bearing immunoreceptors known to negatively regulate the immune response. In addition, human MerTK transgenic, and MerTK knockout mice have been generated and characterized with a view to establish in vivo proof-of-concept with human MerTK mAbs.

**Conclusions**

Pharmacological targeting of MerTK with monoclonal antibodies modulates cytokine production in human and murine model systems in a manner consistent with genetic ablation of the target. MerTK mAbs enhanced production of inflammatory cytokines and decreased the activity of inhibitory immunoreceptors. The anti-tumor activity of MerTK mAbs is planned using surrogate models and human MerTK transgenic mice.

#### P511 Co-expression of a chimeric NKG2D receptor with membrane bound IL-15 enhances natural killer cell function and long-term persistence in vitro and in vivo

##### Luxuan Buren (lburen@nkartatx.com)

###### Nkarta Therapeutics, South San Francisco, CA, USA

**Background**

Chimeric antigen receptors have been used successfully to retarget T cells in patients with hematologic malignancies. Natural killer (NK) cells offer an alternative to T cells for cellular immunotherapy, highly active and suitable for allogeneic use as they are not HLA-restricted and do not cause GVHD. A goal of NK cell engineering is to improve their in vivo persistence and recognition of cancer cells. Ligands of the natural killer group 2D (NKG2D) receptor are broadly expressed in solid tumor and hematological malignancies, making NKG2D an attractive target for NK cell engineering. This work was undertaken to demonstrate that NK activity and persistence can be elevated by simultaneous expression of chimeric constructs directing the expression of an activating NKG2D receptor (aNKr) and a membrane-bound form of IL-15 (mbIL-15).

**Methods**

NK cells were generated by co-culture of peripheral blood mononuclear cells (PBMC) with genetically modified irradiated K562 feeder cells. NK cells were transduced at an MOI of 1-2 with a bicistronic virus encoding an NKG2D aNKr and mbIL-15. NK expansion and NKG2D aNKr expression were evaluated by flow cytometry to detect the CD56+ CD3- cell population and the elevation of NKG2D expression over endogenous levels. In vitro cytotoxicity of transduced NK cells was measured using both flow cytometry and the IncuCyte S3 live cell analysis system. The in vivo activity of engineered NK cells was further assessed in a xenograft tumor model, using the osteosarcoma cell line U2OS engineered to express luciferase, with tumor growth measured using bioluminescence in NSG mice.

**Results**

NK cells were expanded for 7 days (40 fold to >100 fold) prior to transduction. Transduction increased of NKG2D expression in NK cells (>70%, N=8 donors) relative to control cells. NKG2D aNKr-mbIL15 NK cells could be maintained for up to 6 weeks in low IL-2 culture. aNKr-mbIL-15 expression significantly elevated cytotoxicity in NK cells against multiple tumor cell lines, inducing cell death of > 60% of target cells within 4 hours at 1:1 E:T ratio. One infusion of transduced NK cells tumor-bearing NSG mice resulted in long-term anti-tumor responses. Moreover, co-expression of mbIL-15 significantly delayed tumor growth relative to that observed in cells expressing only the NKG2D aNKr.

**Conclusions**

Transduction of NK cells with an NKG2D aNKr and mbIL-15 increases their cytotoxic activity and persistence. Based on these data, further development of NKG2D aNKr-mbIL-15 NK cells for clinical use will be pursued.

#### P512 AO-176, a next generation CD47 antibody, induces immunogenic cell death

##### Ben Capoccia, PhD, Ronald Hiebsch, Michael Donio, Alun Carter, Robyn Puro, Benjamin Capoccia, W. Casey Wilson, Daniel Pereira, Pamela Manning, Robert Karr

###### Arch Oncology, St. Louis, MO, USA

####### **Correspondence:** Daniel Pereira (dpereira@archoncology.com); Ben Capoccia

**Background**

Recent success in cancer immunotherapy has targeted immune checkpoints such as PD-1, PDL-1, and CTLA-4 to enhance the cytotoxic activity of the adaptive T cell immune response. While the clinical response to these therapies has been dramatic for some, many others have shown partial or even no response highlighting the need for alternative or synergistic approaches that activate innate immunity. Disruption of the interaction between SIRPα and CD47, an innate checkpoint inhibitor, using anti-CD47 antibodies, for example, is known to enhance innate immunity by increasing the phagocytosis of tumor cells by macrophages and dendritic cells (DCs) leading to processing and presentation of tumor antigens. Recently, we described AO-176, a next generation anti-CD47 antibody that blocks the CD47/SIRPα interaction, induces phagocytosis and causes a direct tumor cell-autonomous death while negligibly binding RBCs.Herein, we characterize the ability of our CD47 antibodies such as AO-176, to induce Immunogenic cell death (ICD) and Damage Associated Molecular Patterns (DAMPs) in tumor cells and to potentiate chemotherapy-induced ICD/DAMPs. ICD is a process whereby an agent induces cell surface exposure and release of DAMPs from dying cells which stimulates DCs and adaptive immune responses.

**Methods**

Tumor cells were treated in vitro with our CD47 antibodies either alone or in combination with chemotherapeutics followed by assessment of ICD/DAMPs using flow cytometry and biochemical assays. RNAseq was also performed on cells undergoing CD47 antibody mediated ICD/DAMP induction to better understand how CD47 inhibition may regulate ICD.

**Results**

AO-176 and other CD47 antibodies, developed by Arch Oncology, caused mitochondrial stress and loss of outer-membrane integrity, typically observed prior to cells undergoing apoptosis. In addition, CD47 antibody treatment induced a significant ER stress response at the genetic level resulting in the surface exposure of ER chaperone proteins calreticulin, Hsp90, and PDIA3. Concomitantly, our CD47 antibodies increased autophagy and JAK/STAT signaling which resulted in both ATP and HMGB1 release, respectively. Finally, we demonstrated that in combination, our antibodies potentiated the effects of ICD/DAMP-inducing chemotherapy (eg. Doxorubicin).

**Conclusions**

Here, we describe the unique ability of a specific subset of next generation CD47 antibodies, such as AO-176 to induce ICD/DAMPs. RNAseq analysis of treated cells also revealed alteration of several pathways, including those where DAMPs play a role. In summary, next generation CD47 antibodies such as AO-176 may provide a novel approach to enhancing the current landscape of checkpoint immunotherapy by enhancing both the innate and adaptive immune responses against tumors.

#### P513 Targeting adenosinergic immunometabolic suppression with engineered natural killer cells for immunotherapy of CD73+ solid tumors

##### Andrea Chambers, MS, Kyle B. Lupo, Jiao Wang, PhD

###### Purdue University, Lafayette, IN, USA

####### **Correspondence:** Andrea Chambers (sandro@purdue.edu)

**Background**

Genetically engineered natural killer (NK) cells have shown promise as immunotherapies for hematologic malignancies; however, clinical treatment of solid tumors is lagging. This setback is caused by many mechanisms, including accumulation of immunosuppressive adenosine (ADO) [1,2,3] generated from ectoenzymes CD39 and CD73 by cancer cells [4]. We have shown that ADO suppresses NK cell anti-tumor immunity, resulting in downregulation of activating receptor expression and impaired metabolic activity. To overcome immunometabolic suppression due to adenosinergic signaling, we are engineering NK cells directed against CD73 by imparting in situ ADCC-like activation upon NK cells using a novel genetic construct.

**Methods**

Peripheral blood-derived NK cells were isolated from healthy human donors. For ADO studies, NK cells were primed 24 hours with IL-2 (200 IU/ml or 400 IU/ml), IL-15 (100 ng/ml), or IL-12 (20 ng/ml) and IL-15 (100 ng/ml) with or without exogenous ADO (1 mM). Treatments were performed with adenosine A2 receptor inhibitor SCH58261, and EHNA, an ADO deaminase inhibitor. Cytotoxicity against CD73+ cells was measured using 7- AAD/CFSE staining, while IFNγ and activating marker expression were measured by flow cytometry. Differential gene expression due to ADO was determined by RNAseq. Engineering NK cells using a novel genetic construct was made by fusing CD73 scFv with CD16a-derived signaling domains before transcribing into mRNA. CAR NK cells were generated by electroporation of the mRNA, and tested for NK transfection efficiency and effector function against CD73+ solid tumors.

**Results**

Results show that ADO reprograms NK cells’ anti-tumor responses, and priming NK cells with IL-12 and IL-15 can partially mitigate ADO-induced immunosuppression. Using IL-12 and IL-15 was preferential to using IL-2, and IFNγ production in response to ADO was enhanced. Furthermore, ADO resulted in altered gene expression signatures that matched impaired IFNγ signaling and cellular metabolism in NK cells. To block adenosinergic signaling, a novel genetic construct that fuses CD73 scFv with in situ ADCC-like signaling was generated. Human NK cells were successfully electroporated with mRNA encoding the construct. These cells are currently being evaluated for effector function and ability to block CD73-induced immunosuppression on solid tumor targets.

**Conclusions**

The microenvironment of solid tumors is highly immunosuppressive via the activity of CD73 expressed on cancer cells. Our results demonstrate that ADO can act on specific NK cell pathways to cause NK cell inhibition. Harnessing these results, a novel CD73-targeting construct is currently being investigated to redirect NK cell function when targeting solid tumors.

**References**

1. Rezvani K, Rouce RH. The application of natural killer cell immunotherapy for the treatment of cancer. Front Immunol. 2015; 6. 2. Rezvani K, Rouce R, Liu E, Shpall E. Engineering natural killer cells for cancer immunotherapy. Molec. Therapy. 2017;25: 1769-1781 3. Ohta A. A metabolic immune checkpoint: adenosine in tumor microenvironment. Front Immunol. 2016; 6. 4. Antonioli L, Blandizzi C, et al. Anti-CD73 immunotherapy: A viable way to reprogram the tumor microenvironment. Oncoimmunology. 2016;5.

#### P514 High efficiency electroporation of primary human NK cells

##### Jian Chen, PhD, Xiaofeng Xia, PhD

###### Celetrix LLC, Manassas, VA, USA

####### **Correspondence:** Jian Chen (jchen@celetrix.com)

**Background**

(Looking to give a short talk as this technical breakthrough would help many labs in the field.) Natural killer (NK) cells have a great potential as a therapeutic agent against tumor cells. Genetic modifications of NK cells such as gene knock-out or exogenous protein expression are important for boosting NK cell expansion or NK cell killing specificity. Unfortunately, viral vectors that are commonly used for other types of cell immunotherapy have poor efficiency in NK cell transduction. As a physical method, electroporation theoretically should work well with NK cells but the special biology of NK cells have made it difficult to achieve high efficiency in NK cell electroporation and cell viability was also a major issue. For example, electroporation of plasmids used to have poor efficiency and high cell mortality in expanded NK cells.

**Methods**

Here we used a two-pronged approach to tackle the NK cell electroporation problem. First, a novel electroporation method was used involving a new device that has surpassed the performance of all other electroporation technologies on the market. Second, instead of using expanded NK cells, we used fresh un-expanded NK cells that were previously considered harder for electroporation.

**Results**

Using a relatively high cell concentration, we selected a high electric field strength and were able to quickly achieve a very high efficiency (40% to 50%) for fresh NK cells electroporated with plasmids. The viability of the NK cells after electroporation was between 85% and 95%. Electroporation of mRNA or Cas9/gRNA ribonucleoproteins (RNPs) is much easier than electroporation of plasmids and the new method would allow complex experimental designs such as co-transfection of RNP and plasmids for knock-in.

**Conclusions**

With the new high efficiency NK cell electroporation method, genetic modification for NK cells has become easily accessible, thereby allowing more possibilities of clinical anti-tumor applications of NK cells.

#### P515 Novel class of small molecule direct STING agonists as potential cancer immunotherapy

##### Monika Dobrzanska, Stefan Chmielewski, PhD, Magdalena Zawadzka, Adam Radzimierski, Karolina Gluza, Katarzyna Wojcik-Jaszczynska, Maciej Kujawa, Grzegorz Topolnicki, Grzegorz Cwiertnia, Aleksandra Poczkaj, Izabela Dolata, Jolanta Mazurek, Magdalena Mroczkowska, Agnieszka Gibas, Tushar Mahajan, Marcin Les, Wojciech Schonemann, Sylwia Sudol, Kinga Michalik, Magdalena Sieprawska-Lupa, Katarzyna Banaszak, Charles-Henry Fabritius, Karolina Wiatrowska, Agnieszka Adamus

###### S.A., Krakow, Poland

####### **Correspondence:** Monika Dobrzanska (monika.dobrzanska@selvita.com)

**Background**

Type I interferons are major players in mounting immune response to cancer cells. IFNβ release by antigen-presenting cells promotes spontaneous anti-tumor CD8+ T cell priming being largely dependent on activation of Stimulator of Interferon Genes (STING). In preclinical murine models intratumoral injection of cyclic dinucleotide (CDN) STING agonists induced regression of established tumors and generated long-term immunologic memory. Relative instability and chemical nature of cyclic dinucleotides limit their use as systemically available immunotherapeutics. Therefore, herein we report the discovery of potent and selective first-in-class non-nucleotide, non-macrocyclic, small molecule direct STING agonists, structurally unrelated to known chemotypes with potential for systemic administration routes.

**Methods**

Cytokine release has been determined by ELISA or AlphaLisa in human peripheral blood mononuclear cells (PBMC) obtained from healthy human subjects. Activation of STING pathway was monitored in THP-1 Dual reporter monocytic cell line. Human monocyte-derived macrophages (HMDM) and human monocyte-derived dendritic cells (HMDC) were differentiated from CD14+ cells (obtained from PBMC) in the presence of GM-CSF and GM-CSF with IL-4 for HMDM and HMDC, respectively. Mouse bone marrow-derived macrophages (BMDM) were obtained from C57BL/6 mice. Surface expression of the antigen-presenting cell maturation markers i.e. CD80, CD86, CD83 and HLA-DR was assessed by flow cytometry with corresponding isotype controls. The binding affinity was confirmed by Fluorescence Thermal Shift, Fluorescence Polarisation and Microscale Thermophoresis.

**Results**

STING agonists have confirmed direct binding to both mouse and human STING protein in three independent biophysical binding assays (FTS, MST and FP) and by additional crystallography studies. The compounds have fine-tunable ADME properties with particularly good solubility, permeability and human plasma stability. They selectively activated STING-dependent signaling in both THP-1 reporter assays and in primary cells of human and mouse origin. In vitro functional assays demonstrated their ability to induce cytokine responses (IFNβ, TNFα) in a panel of human peripheral blood mononuclear cell (PBMC), human monocyte derived macrophage (HMDM) and human dendritic cells samples with various STING haplotypes. Additionally, the compounds efficiently induced cytokine release in mouse bone marrow-derived macrophages. Pro-inflammatory cytokine profile was accompanied by up-regulation of the maturation markers, CD80, CD86, CD83 and HLA-DRon the surface of human antigen presenting cells.

**Conclusions**

These data demonstrate potent, novel, next-generation small molecule STING agonists activating STING-dependent signaling in both mouse and human immune cells to promote potential antitumor immunity. The compounds show good selectivity and in vitro ADME properties enabling further development for systemic administration as a single agent or in combinatory cancer immunotherapies.

#### P516 SIRPα blockade increases the activity of multiple myeloid lineage cells, enhances dendritic cell cross- presentation, and aids in remodeling the tumor microenvironment

##### Brian Francica, Jay Hyok Chung, Brandy Chavez, Erik Voets, PhD, Andrea van Elsas, Hans van Eenennaam, PhD, Meredith Leong

###### Biotech Europe, Oss, Netherlands

####### **Correspondence:** Brian Francica (BFrancica@aduro.com)

**Background**

Antagonizing the SIRPα-CD47 pathway is gaining traction as an effective and novel approach to immune manipulation as design of immunotherapies broadens to include blockade of innate immune checkpoints. Recently, the combination of tumor-targeting antibodies with SIRPα-CD47 blockade has provided promising clinical results, suggesting that increased phagocytosis of cancer cells is clinically relevant for treatment of hematologic cancers [1]. However, the ability for this combination to enhance phagocytosis in the context of solid tumors may be remarkably diminished for several reasons including reduced expression of “eat-me” signals like SLAMF7, increased immune suppression in the tumor microenvironment (TME), and the physical size of tumor cells when adhered in a complex heterogeneous environment. To achieve efficacy in solid tumor indications, it is important that therapies blocking the SIRPα-CD47 axis also potentiate adaptive immune mechanisms and not solely phagocytosis.

**Methods**

Subcutaneous mouse tumor models and a mouse bone marrow-derived dendritic cell (BMDC) cross-presentation assay were used to assess the efficacy of SIRPα blockade in solid tumors.

**Results**

Here we demonstrate that, in addition to increasing macrophage uptake of tumor cells in suspension, SIRPα blockade also functions to modify the myeloid compartment in the TME of solid tumors. In four independent subcutaneous mouse tumor models, we demonstrate that SIRPα blockade combines in a synergistic manner with PD-1 blockade to reduce tumor burden. In these models, anti-SIRPα therapy skews the DC population towards cross-presenting DC1 cells and increases the CD86 expression on myeloid cells in multiple immune tissues. In vivo and in vitro, SIRPα blockade correlates with lower levels of SIRPα present on the cell surface, and we hypothesize that a combination of downregulation and blockade may cause the skewing of myeloid lineages. Using a mouse BMDC cross-presentation assay, we also demonstrate that the blockade of SIRPα results in increased T cell expansion, supporting a role for SIRPα blockade in enhancing DC function.

**Conclusions**

Together, these data suggest that antagonizing SIRPα functions to skew myeloid cells. This results in enhanced T cell activation and that, when combined with PD-1 blockade, improves therapeutic efficacy in multiple mouse models. Based on these data in mouse models, an antibody with specificity for human SIRPα, ADU-1805, is being developed for use in clinical trials.

**References**

1. Liu X, Kwon H, Li Z, Fu Y-X. Is CD47 an innate immune checkpoint for tumor evasion? Journal of Hematology & Oncology. 2017Nov;10(1).

#### P517 Pan-allele anti-SIRPα antibodies that block the SIRPα–CD47 innate immune checkpoint

##### Erik Voets, PhD, Jay Hyok Chung, Paul Vink, BS, David Lutje Hulsik, Marc Paradé, Sanne Spijkers, Inge Reinieren-Beeren, Joost Rens, Wout Janssen, BS, Brian Francica, Meredith Leong, Andrea van Elsas, Hans van Eenennaam, PhD

###### Biotech Europe, Oss, Netherlands

####### **Correspondence:** Erik Voets (EVoets@aduro.com)

**Background**

SIRPα immunoregulatory activity on myeloid cells is activated by binding of its ligand CD47 [1,2], and blockade of the pathway may enhance anti-tumor immunity [3,4]. Hence the pathway is thought to represent a novel immune checkpoint. CD47, being ubiquitously expressed on normal cells and upregulated on many cancer cells, has been extensively studied in the context of “don’t-eat-me” [5,6]. Alternative strategies are focusing on directly targeting SIRPα because of its more restricted expression to myeloid-derived lineages [7].However, the identification of functional human SIRPα antagonistic antibodies has been hampered by the allelic variation in the SIRPα locus and its homology with the activating receptor SIRPβ and the decoy receptor SIRPγ.

**Methods**

Using Aduro Biotech’s B-select platform, we have identified and characterized ADU-1805: a highly selective pan-allele anti-SIRPα antibody (EC50 SIRPαV1/SIRPαV2 ≤ 3nM) that lacks appreciable SIRPβ binding (EC50 > 120nM) and cross-reacts with SIRPγ (EC50 ≤ 5nM).

**Results**

ADU-1805 potently blocks CD47 binding (IC50 ≤ 1.5nM) in all known human *SIRPA* genotypes (including homozygous and heterozygous genotypes) and antagonizes SIRPα–CD47 interaction on primary SIRPα+ myeloid cells (IC50 ≤ 4nM). In line with its antagonistic properties, ADU-1805 enhances tumor cell clearance by human granulocytes and macrophages. Furthermore, on the IgG2 subclass backbone selected during the humanization process, ADU-1805 exhibits improved activity relative to other IgG subclasses tested. Finally, unlike data with CD47-targeting antibodies, ADU-1805 does not trigger hemagglutination or platelet binding/aggregation *in vitro*, suggesting a reduced risk of red blood cell (RBC) and platelet depletion *in vivo*.

**Conclusions**

In summary, we have identified ADU-1805 as a potentially best-in-class antagonistic anti-SIRPα antibody with a unique binding profile as it binds all reported human SIRPα alleles but does not appreciably bind to the activating SIRPβ receptor. Blocking the SIRPα–CD47 innate immune checkpoint with ADU-1805 may modulate myeloid cells in the tumor microenvironment and promote antigen presentation and cross-priming of dendritic cells. We are currently advancing ADU-1805 through preclinical studies.

**References**

1. Oldenborg PA, Zheleznyak A, Fang YF, Lagenaur CF, Gresham HD, Lindberg FP. Role of CD47 as a marker of self on red blood cells. Science. 2000;288(5473):2051–4.
2. Oldenborg PA, Gresham HD, Lindberg FP. Cd47-signal regulatory protein α (Sirpα) regulates Fcγ and complement receptor–mediated phagocytosis. The Journal of Experimental Medicine. 2001Feb;193(7):855–62.
3. Tseng D, Volkmer J-P, Willingham SB, Contreras-Trujillo H, Fathman JW, Fernhoff NB, et al. Anti-CD47 antibody-mediated phagocytosis of cancer by macrophages primes an effective antitumor T-cell response. Proceedings of the National Academy of Sciences. 2013;110(27):11103–8.
4. Liu X, Pu Y, Cron K, Deng L, Kline J, Frazier WA, et al. CD47 blockade triggers T cell–mediated destruction of immunogenic tumors. Nature Medicine. 2015;21(10):1209–15.
5. Jaiswal S, Jamieson CH, Pang WW, Park CY, Chao MP, Majeti R, et al. CD47 is upregulated on circulating hematopoietic stem cells and leukemia cells to avoid phagocytosis. Cell. 2009;138(2):271–85.
6. Majeti R, Chao MP, Alizadeh AA, Pang WW, Jaiswal S, Gibbs KD, et al. CD47 is an adverse prognostic factor and therapeutic antibody target on human acute myeloid leukemia stem cells. Cell. 2009;138(2):286–99.
7. Yanagita T, Murata Y, Tanaka D, Motegi S-I, Arai E, Daniwijaya EW, et al. Anti-SIRPα antibodies as a potential new tool for cancer immunotherapy. JCI Insight. 2017Dec;2(1).

#### P518 Non-canonical cross-presenting dendritic cells mediate anti-tumor immunity

##### Ellen Duong, ScB, Stefani Spranger, PhD

###### MIT, Cambridge, MA, USA

####### **Correspondence:** Stefani Spranger (spranger@mit.edu)

**Background**

Recent studies revealed a critical role for cross-presenting CD103^+^ dendritic cells (DC1) in both the induction and maintenance of CD8^+^ T cell immunity in the tumor. Exclusion of DC1 from the tumor microenvironment (TME) is a mechanism of immune evasion by the tumor and contributes to impaired responses to checkpoint blockade therapy. Elucidating the contributions of different DC subsets to the TME will be instrumental towards improving current immunotherapies.

**Methods**

We compared the myeloid infiltrate of acutely cleared regressor tumors versus progressively growing tumors, an approach that was previously used to phenotype dysfunctional T cells in the TME [1]. Murine syngeneic tumor lines expressing SIY were categorized as ‘progressor’ or ‘regressor,’ and implanted in wild-type, Rag2^-/-^, Batf3^-/-^, Clec9a^GFP/GFP^, and CD11c-DTR bone marrow (BM) chimera mice. Flow immunophenotyping was used to profile the intratumoral myeloid compartment, and ELISpot was performed to determine the number of IFNγ-producing CD8^+^ T cells. To assess function, sorted myeloid cells were co-cultured with CD8^+^ T cells to evaluate their ability to induce T cell proliferation. Single cell-RNA-sequencing was performed to profile the cellular components of the TME in an unbiased fashion.

**Results**

In contrast to progressor tumors, regressor tumors were more infiltrated with DC1 than other DC types (non-DC1). A high DC1/non-DC1 ratio was correlated with increased intratumoral CD8^+^ T cell infiltration and was predictive of tumor control across different tumor types and mouse strains. Batf3^-/-^ and Clec9a^GFP/GFP^ mice, which lack functional DC1, were able to eliminate a subset of regressor tumors, suggesting that the regression of these tumors was independent of Clec9a-mediated cross-presentation by Batf3-driven DC1. By IFNγ-ELISpot, we found that while CD8^+^ T cell priming was completely ablated in Batf3^-/-^ and Clec9a^GFP/GFP^ mice implanted with progressor tumors, mice with regressor tumors retained ~50% CD8^+^ T cell priming. Ex vivo co-culture assays of 2C CD8^+^ T cells with sorted myeloid cells from SIY-expressing tumors in Batf3^-/-^ mice indicated the presence of CD11c^+^ cells capable of non-canonical cross-presentation. Ablation of the CD11c^+^ compartment using diphtheria toxin-treated CD11c-DTR BM chimeras resulted in loss of T cell priming and anti-tumor immunity in the regressor tumor. Single-cell sequencing of the regressor tumor indicated the presence of a novel DC subset capable of non-canonical cross- presentation in DC1-deficient mice.

**Conclusions**

Identifying the cell type(s) involved and the mechanism of non-canonical cross-presentation in regressor tumors can open new therapeutic avenues to stimulate the anti-tumor immune response when Batf3-driven DC1 are excluded from the tumor.

**References**

1. Williams et al. The EGR2 targets LAG-3 and 4-1BB describe and regulate dysfunctional antigen-specific CD8^+^ T cells in the tumor microenvironment. J Exp Med. 2017; 214(2):381-400.

#### P519 Agonist redirected checkpoint platform (ARC), engineering bi-functional fusion proteins (SIRP -Fc-CD40L), for cancer immunotherapy

##### George Fromm, PhD^1^, Suresh de Silva, PhD^2^, Taylor Schreiber, MD, PhD^2^

###### ^1^Shattuck Labs, Inc, Apex, NC, USA; ^2^Shattuck Labs, Inc., Durham, NC, USA

####### **Correspondence:** George Fromm (gfromm@shattucklabs.com)

**Background**

A majority of clinical responses to PD1/L1 blockade occur in patients with abundant intratumoral PD-L1 expression and lymphocyte infiltration, suggesting that additional efficacy may be found in combination therapies that increase either of these variables. Because PD1/L1 blockade augments the cytotoxic potential of T cells, synergistic pathways could include those that reduce immunosuppressive myeloid cells or enhance antigen presentation. Here we report the generation of a novel, two-sided human fusion protein (Agonist Redirected Checkpoint, ARC), incorporating the extra cellular domains of SIRPα and CD40L, adjoined by a central Fc domain; termed SIRPα-Fc-CD40L. The SIRPα-Fc-CD40L construct was designed to simultaneously enhance antigen uptake and cross-presentation (CD47 axis) and enhance antigen presenting cell maturation and function (CD40 axis), with a single compound.

**Methods**

Human and mouse SIRP -Fc-CD40L were produced and characterized using a range of biochemical assays to determine molecular weight, subunit composition & binding affinity; molecular assays to characterize in vitro/ex vivo binding, in vitro functional activity; and anti-tumor efficacy in multiple syngeneic tumor model systems. SIRP-Fc-CD40L has entered late stage manufacturing.

**Results**

The SIRPα end of the ARC bound immobilized CD47 at 3.59 nM affinity and also CD47 on the surface of human tumor cells both in vitro and in vivo, but importantly, did not bind human platelets, RBCs, nor induce hemolytic activity. The CD40L end of the ARC bound immobilized CD40 at 756 pM affinity and also CD40 on primary macrophages. The SIRP -Fc-CD40L ARC stimulated Fc receptor-independent NF B-luciferase signaling and also induced cytokine secretion from human PBMCs, both with and without TCR stimulation. Furthermore, when activated human dendritic cells or macrophages were co-cultured with CD47 positive human tumor cells, SIRPα - Fc-CD40L was shown to enhance phagocytosis of human tumor cells, and in vivo in mice, induced rapid activation of CD4+ and CD8+ dendritic cells. Finally, the therapeutic activity of SIRPα-Fc-CD40L in established murine MC38 and CT26 tumors was superior to CD47-blocking antibody, CD40-agonist antibody, and combination antibody therapy. Interestingly, anti-tumor response was heightened significantly when SIRPα-Fc-CD40L was combined with antibody blockade of CTLA4, PD1, or PDL1.

**Conclusions**

These data demonstrate feasibility of a novel chimeric fusion protein platform, providing checkpoint blockade and TNF superfamily costimulation in a single molecule. Signal replacement of CD47 by CD40L may uniquely poise DCs/macrophages in the tumor microenvironment for activation and cross-presentation of tumor antigens following enhanced tumor cell phagocytosis.

#### P520 Natural killer (NK) cells orchestrate the antitumor activities of Listeria monocytogenes (Lm)-based immunotherapy

##### Rachelle Kosoff, PhD^1^, Lauren Pettit, MS^1^, Nithya Thambi, MS^1^, Kimberly Ramos, Bachelors in Small Animal Science^1^, Jeff Jones^1^, Skye Kuseryk^1^, Robert Petit, PhD^1^, Michael Princiotta, MS, PhD^1^, Kim Jaffe, PhD^1^, Sandy Hayes, PhD^2^

###### ^1^ADVAXIS, INC, Princeton, NJ, USA; ^2^Advaxis Immunotherapies, Inc, Princeton, NJ, USA

####### **Correspondence:** Sandy Hayes (hayes@advaxis.com)

**Background**

Advaxis’ Lm-based immunotherapies are antigen-based immunotherapies that are designed to elicit tumor antigen- specific T cell effectors that recognize and kill tumor cells. However, because the tumor antigens are delivered by a bacterial vaccine vector, innate cytotoxic effectors, such as NK cells, may also be recruited to play a role in controlling tumor growth. The purpose of this study is to determine whether and how NK cells contribute to the antitumor activities of Lm-based immunotherapy.

**Methods**

Tumor growth inhibition was evaluated in C57BL/6 mice that were implanted with human papillomavirus (HPV)16+ TC-1 tumor cells and then immunized on days 8, 15 and 22 after tumor implantation with PBS or with axalimogene filolisbac (AXAL), an Lm-based immunotherapy expressing the HPV16 E7 protein. To in vivo deplete NK cells, anti-asialo GM1 antibody (Ab) was administered 1 day before tumor implantation and at 3-day intervals during the PBS or AXAL treatment regimen. For mechanistic studies, flow cytometric analysis and immune-related gene profiling of tumor infiltrating leukocytes (TILs) were performed at various time points after tumor implantation.

**Results**

We first compared intratumoral NK cell frequency and maturation in PBS- and AXAL-treated mice. Although the percentages of intratumoral NK cells in PBS- and AXAL-treated mice were equivalent, NK cells in tumors of AXAL-treated mice were more functionally mature, based on their high expression of CD11b and granzyme A, than NK cells in tumors of PBS-treated mice. To determine whether AXAL-induced NK cell activity was required for AXAL-mediated tumor control, we used anti-asialo GM1 Ab to in vivo deplete NK cells. In AXAL-treated mice, NK cell depletion resulted in a complete loss of tumor growth inhibition. Phenotypic and functional analyses of TILs revealed impaired dendritic cell (DC) maturation and significantly reduced infiltration of functional HPV- specific CD8+ T cells in NK cell-depleted AXAL-treated mice compared to AXAL-treated mice. Gene profiling and pathway analysis showed that the genes significantly downregulated in tumors of NK cell-depleted AXAL-treated mice versus tumors of AXAL-treated mice were involved in NK cell signaling, DC maturation, and interferon signaling.

**Conclusions**

Treatment of tumor-bearing mice with AXAL leads to NK cell activation, DC maturation and, by extension, an effective antitumor T cell response. These data suggest that NK-DC cross-talk, which leads to activation and maturation of both cell types, is a mechanism by which NK cells contribute to AXAL’s antitumor activities.

**Ethics Approval**

All mouse experiments were performed under approved IACUC protocols (0914A2016 and 0914B2016).

#### P521 T cell immunotherapies trigger innate immunity and aseptic inflammation leading to potent anti-tumor and off-targets effects

##### Daniel Hirschhorn-Cymerman, PhD^1^, Jacob Ricca^2^, Billel Gasmi, MD^2^, Olivier De Henau, MD^2^, Levi Mangarin, BS^2^, Sadna Budhu, PhD^2^, Yanyun Li, PhD MD^2^, Czrina Cortez, BS^2^, Cailian Liu, MD^2^, Roberta Zappasodi, PhD^2^, Sean Houghton^3^, Allison Betof^2^, Katherine Panageas, PhD^2^, Mario Lacuoture, MD^2^, Tracvis Hollmann, MD PhD^2^, Jean Albrengues, PhD^3^, Mikala Egeblad, PhD^3^, Taha Merghoub, PhD^2^, Jedd Wolchok, MD, PhD^2^

###### ^1^Memorial Sloan Kettering Cancer Center, New York, NY, USA; ^2^MSKCC, New York, NY, USA; ^3^Cold Spring Harbor Laboratory, Cold Spring Harbor, NY

####### **Correspondence:** Jedd Wolchok (wolchokj@mskcc.org)

**Background**

Mobilizing the immune system to treat advanced cancers is now a clinical reality. Successful immune-based therapies that treat tumors are often accompanied by immune-related adverse events (irAE) that can occasionally present with severe and lethal symptoms. Currently, there are no well-defined preventative approaches to uncouple anti-tumor immunity from irAEs. The primary immunotherapies currently in clinical use include agents that activate T cell responses such as checkpoint blockade of inhibitory pathways and infusion of ex-vivo tumor-derived, or T cell receptor (TCR)-transgenic or chimeric antigen receptor-modified T cells. While the beneficial and toxic effects of T cell-based immunotherapies in the clinic are being extensively explored, the precise mechanisms underlying their activity remain the subject of intense investigation.

**Methods**

In the present study, we treated established tumors with melanoma-specific adoptive CD4+ T cell transfer and costimulation via OX40 or CTLA-4 blockade.

**Results**

We found that, in spite of adequate T cell stimulation, acute local inflammation plays a fundamental role in tumor elimination and related irAEs. While stimulated T cells are necessary for initiating a therapeutic response, activation of endogenous neutrophils constitute an important and necessary effector mechanism of tumor destruction and irAEs. Extensive neutrophil extracellular traps (NETs) were associated with irAEs. Furthermore, melanoma patients treated with checkpoint blockade who developed skin rashes equivalent to irAEs found in mice, showed increased survival and NETs were found in biopsies from rashes and tumors.

**Conclusions**

Our results bring forward a novel paradigm where T cells enact an anti-tumor immune response that is followed by an inflammatory effector mechanism provided by the innate immune system with curative as well as morbid effects in mice and patients.

**Ethics Approval**

All tissues were collected at MSKCC following consent to an institutional biospecimen collection study protocol approved by the MSKCC Institutional Review Board. Informed consent was obtained for all patients. The study was in strict compliance with all institutional ethical regulations

#### P522 Immune-profiling reveals DNAM-1 downregulation in tumor-infiltrating lymphocytes of renal cell carcinoma patients

##### Veronika Kremer, MSc^1^, Christina Seitz, MSc^1^, Ann-Helen Scherman Plogell, MD^2^, Eugenia Colon, MD, PhD^2^, Evren Alici, MD, PhD^1^, Andreas Lundqvist, PhD^1^

###### ^1^Karolinska Institutet, Stockholm, Sweden; ^2^Stockholm South General Hospital, Stockholm, Sweden

####### **Correspondence:** Christina Seitz (christina.seitz@ki.se)

**Background**

Natural killer (NK) cells infiltrate renal cell carcinoma (RCC) tumors and may play a key role in modulating tumor progression, as high NK cell frequencies have been correlated with improved patient survival. However, NK cells, as well as T cells, have been found be phenotypically and functionally suppressed in the tumor microenvironment.

**Methods**

We investigated immune cell phenotypes and secretion signatures in blood and primary tumors of RCC patients and applied the supervised multivariate analysis tool Orthogonal Projections to Latent Structures (OPLS) to correlate those with disease parameters.

**Results**

We found that DNAM-1 expression on intratumoral NK cells is associated with a lower Fuhrman grade, whereas PD-1 expression on peripheral blood lymphocytes correlates with a lower primary tumor stage. Furthermore, we identified differences in the immune profiles of blood and tumor of each patient using an OPLS approach. We showed that DNAM-1 is significantly downregulated on tumor-infiltrating T and NK cells compared with peripheral blood (p=0.0006). Indeed, our in vitro experiments suggested that this is likely triggered by contact with tumor cells.

**Conclusions**

Our results suggest that while T and NK cells can be activated by RCC tumors, they are also inhibited through DNAM-1 down-regulation, which seems to be a central mechanism of immune escape by RCC tumors.

**Ethics Approval**

The study was approved by the Regional Ethical Review Board in Stockholm, approval number #2013-570-31.

#### P523 Engineering responsive multi-functional natural killer cells derived from induced pluripotent stem cells capable of overcoming immunometabolic suppression for immunotherapy of solid tumors

##### Kyle B. Lupo, Andrea M. Chambers, Jiao Wang, PhD, Sandro Matosevic, PhD

###### Purdue University, Lafayette, IN, USA

####### **Correspondence:** Sandro Matosevic (sandro@purdue.edu)

**Background**

Targeted immunotherapy with engineered natural killer (NK) cells has proven to be a promising approach for the treatment of solid tumors. The pathogenesis of solid cancers, however, causes severe immunosuppression, due to mechanisms which include the generation of immunosuppressive adenosine by the cancer-associated enzyme CD73, as well as the expression of CD155, which causes immune cell dysfunction. Moreover, challenges in sourcing NK cells impair the development of immunotherapies for solid tumors, promoting interest in using induced pluripotent stem cells (iPSCs) as a source of allogeneic NK cells.

**Methods**

We have generated “off-the-shelf” NK cells differentiated from iPS cells using a novel feeder-free cell culture protocol. These cells can act as a source of allogeneic NK cells that can be genetically engineered for use in cancer immunotherapy. Additionally, we are genetically engineering NK cells with a responsive genetic construct which combines extracellular TIGIT, a ligand for CD155, with intracellular signaling elements that redirect inhibition induced by CD155/TIGIT interaction to trigger release of CD73 scFv. These multi-functional NK cells are capable of competitively binding to CD155+ cancer cells, displacing the inhibition induced by CD155/TIGIT interaction, and blocking CD73. Engineered NK cells are generated via transfection using mRNA electroporation and assessed for cytotoxic function against CD73+ targets.

**Results**

Initially, iPS cells were differentiated into hematopoietic progenitor cells. These cells were characterized via flow cytometry using cell surface markers expressed on hematopoietic progenitors – CD34, CD43, and CD45. Differentiation yielded CD34+/CD45+ and CD34+/CD43+ cell populations of approximately 20% and 13%, respectively, consistent with results described in literature using feeder-based protocols [1]. Following four weeks of NK cell culture, cells were further analyzed and yielded high expression of several NK cell maturation markers under optimal conditions (greater than 70%, 45%, and 55%, for CD3-/CD56+, CD94+, and NKp46+ cell populations, respectively). In parallel, we have generated a responsive construct targeting CD155 with the concomitant release of CD73 scFv and are characterizing its effect on NK cells’ anti-tumor immunity.

**Conclusions**

We have shown that NK cells can be generated from iPSCs using an efficient feeder-free protocol. We also synthesized a novel genetic construct which redirects TIGIT-induced inhibition and triggers release of therapeutic CD73 scFv. NK cells engineered with our construct are expected to inhibit immunometabolic suppression due to multi-functional CD73 and CD155 activity and enhance the killing ability of NK cells against solid tumor targets, allowing improved cancer targeting over traditional chimeric antigen receptor-NK therapies.

**Acknowledgements**

Jungil Moon, Ph. D., iPS Facility Coordinator, Purdue University, West Lafayette, I.N.

**References**

1. Bock A, Knorr D, and Kaufman D. Development, expansion, and in vivo monitoring of human NK Cells from human embryonic stem cells (hESCs) and induced pluripotent stem cells (iPSCs). Journal of Visualized Experiments : JoVE. 2013;74: 50337.

#### P524 Preclinical characterization of BMS-986299, a first-in-class NLRP3 innate agonist with potent antitumor activity, alone and in combination with checkpoint blockade

##### James Burke^2^, Michael Wichroski, PhD^2^, Huilin Qi^2^, Jie Fang^2^, Adam Bata^2^, Ramola Sane^2^, Anwar Murtaza, PhD^2^, Ashvinikumar Gavai, PhD^2^, Ragini Vuppugalla^2^, Frederic Reu^2^, Dana Banas^2^, Julie Carman, PhD^2^, Damien Bertheloot^3^, Dennis Dean^3^, Luigi Franchi^3^, Shomir Ghosh^3^, Gary Glick^3^, Jonathan Graves^3^, Ana Kitanovic, PhD^3^, Eicke Latz^3^, Xiaokang Lu, BS^3^, Edward Olhava^3^, William Roush^3^, Brian Sanchez^3^, Andrea Stutz^3^, David Winkler, PhD^3^, John Hunt, PhD^2^, Miguel Sanjuan, PhD^2^, James Burke^2^

###### ^1^Chrysalis Medical, Hayward, CA, USA; ^2^Bristol-Myers Squibb, Lawrence Township, NJ, USA; ^3^IFM Therapeutics, Princeton, NJ, USA

####### **Correspondence:** Michael Wichroski (Michael.Wichroski@bms.com)

**Background**

Immune checkpoint inhibitors (CPI) targeting adaptive immunity have significantly improved patient outcomes in many tumor types, but other approaches are needed to extend clinical benefit to more patients. Targeting innate immunity to provide broader activation of the immune system may be one approach to complement CPI activity. The NLRP3 inflammasome pathway, a key mediator of innate immunity and immune homeostasis, promotes pro- inflammatory response through the maturation of cytokines interleukin-1β (IL-1β) and IL-18, which drive augmented adaptive immune and T-cell memory responses. Targeting innate immune activation through the NLRP3 inflammasome represents a novel and differentiated approach to activating the antitumor immune cycle. Here, we present the preclinical evaluation of a first-in-class NLRP3 agonist BMS-986299 ± CPI.

**Methods**

Cellular activation of the NLRP3 inflammasome was investigated using cell imaging, biochemical methods, and cytokine release assays. Selectivity of BMS-986299 was confirmed in NLRP3-deficient cells. To investigate whether local activation of NLRP3 could drive systemic antitumor immunity, resulting in abscopal antitumor effects, BMS-986299 (intratumorally 6 to 100 μg Q2D×3 or Q1W) ± anti-PD-1 or anti-CTLA-4 CPI (intraperitoneally) was evaluated in mouse syngeneic tumor models (eg, EG7 thymoma and MC38 colon adenocarcinoma); abscopal activity was measured in a noninjected distal tumor.

**Results**

BMS-986299 selectively activated the human and mouse NLRP3 inflammasome, resulting in inflammasome assembly (ASC speck formation), caspase-1 activation, and IL-1β and IL-18 cleavage/release. BMS-986299 induced IL-1β at EC_50_ ≈ 0.5 μM in human PBMCs. In allogeneic mixed lymphocyte reaction assays, single-agent BMS-986299 enhanced T-cell activation; the addition of CPI resulted in further improvements. Pharmacodynamic studies revealed that intratumoral administration of BMS-986299 led to localized release of IL-1β and IL-18 within injected tumors. In the EG7 model, BMS-986299 was sufficient to induce complete regression in both injected and abscopal tumors; efficacy was abrogated in NLRP3 deficient mice, confirming that antitumor activity was NLRP3-dependent for this molecule. In the MC38 model, BMS-986299 in combination with PD-1 blockade resulted in reduced tumor growth in both injected and abscopal tumors (≥ 50% complete tumor regressions). In both models, all mice achieving complete tumor regression with BMS-986299 ± CPI rejected fresh tumor cells when rechallenged without further treatment, demonstrating long-term, durable antitumor immunity.

**Conclusions**

BMS-986299 is a first-in-class, selective NLRP3 innate agonist, with robust preclinical antitumor activity ± CPI. These data support the ongoing clinical evaluation of BMS-986299 as a novel therapeutic for the treatment of solid tumors in combination with CPI [NCT03444753].

**Ethics Approval**

This preclinical study was conducted in accordance with ethical principles and local laws/regulations. The use of samples were reviewed and approved by an institutional review board or independent ethics committee.

#### P525 Preclinical characterization of BMS-986301, a differentiated STING agonist with robust antitumor activity as monotherapy or in combination with anti–PD-1

##### Gary Schieven, PhD^2^, Jennifer Brown^2^, Jesse Swanson, BS^2^, Caitlyn Stromko^2^, Ching-Ping Ho, BS^2^, Rosemary Zhang^2^, Bifang Li-Wang^2^, Hongchen Qiu^2^, Huadong Sun^2^, Brian Fink, PhD^2^, Anwar Murtaza, PhD^2^, John Hunt, PhD^2^

###### ^1^Chrysalis Medical, Hayward, CA, USA; ^2^Bristol-Myers Squibb, Princeton, NJ, USA

####### **Correspondence:** Gary Schieven (gary.schieven@bms.com)

**Background**

Immune checkpoint inhibitors (CPI) targeting adaptive immunity have improved patient outcomes in many tumors, but other approaches are needed to extend benefit to more patients. Targeting innate immunity to provide broader activation of the immune system may be one approach to complement CPI activity. Stimulator of interferon genes (STING) enhances antitumor immunity by inducing innate immune responses leading to T-cell priming and activation, resulting in a more effective antitumor response. Here we present the preclinical evaluation of the novel STING agonist BMS-986301 ± anti–PD-1.

**Methods**

BMS-986301 activity was studied in reporter cell lines and mouse/human peripheral blood mononuclear cells (PBMCs). T-cell proliferation and survival were evaluated in resting and activated T cells. Antitumor activity of BMS-986301 (intratumorally) ± anti–PD-1 (intraperitoneally) was evaluated in bilaterally implanted staged (100 mm^3^) CT26 or MC38 mouse tumor models; abscopal activity was measured in the noninjected distal tumor. Immune cell levels were measured by flow cytometry, with tetramer staining of tumor-reactive CD8^+^ T cells. The STING agonist ADU-S100 was used as a reference.

**Results**

BMS-986301 induced cytokine and Type I interferon response gene expression, with comparable potency in human and mouse PBMCs. In human PBMCs, comparable activity was observed across major STING variants. No responses were observed in STING-deficient cells or mice, demonstrating specificity. Because STING agonists can inhibit T-cell proliferation and survival, BMS-986301 was tested and showed low cytotoxicity toward CD8^+^ resting human T cells and decreased inhibition of proliferation of activated human T cells in vitro relative to ADU-S100. BMS-986301 monotherapy (250 ug every 4 days [Q4D]×3) achieved >90% complete regressions of injected and noninjected tumors in both tumor models, showing abscopal effects in a dose-dependent manner. Identical dosing with ADU-S100 (250 ug Q4D×3) provided 13% complete regressions in both tumor models. In the CT26 model, anti–PD-1 plus a single dose of BMS-986301 (100 ug) provided 80% complete regressions of injected/noninjected tumors; no complete regressions were observed with anti–PD-1 alone. BMS-986301 induced increased expression of genes associated with T-cell activation in tumors and draining lymph nodes, induced T-cell proliferation, and increased NK-cell infiltration into tumors. In the CT26 model, antitumor activity correlated with induction of circulating tumor-reactive T cells. All CT26 mice achieving complete regressions with BMS-986301 rejected fresh tumor cells without further treatment, demonstrating immunological memory.

**Conclusions**

BMS-986301 is a differentiated STING agonist, with promising preclinical antitumor activity alone and in combination with anti–PD-1, supporting its evaluation in future clinical studies.

**Ethics Approval**

This preclinical study was conducted in accordance with ethical principles and local laws/regulations. The use of samples were reviewed and approved by an institutional review board or independent ethics committee.

#### P526 The potential role of fibroblast activation protein as a natural killer cell immune checkpoint in pancreatic cancer

##### Louis Weiner, MD, Shangzi Wang, PhD, Allison O'Connell, MD/PhD Candidate

###### Georgetown University, Washington, DC, USA

####### **Correspondence:** Louis Weiner (weinerl@georgetown.edu)

**Background**

Immunotherapy has been largely ineffective in pancreatic cancer, partially due to the dense stromal fibrosis surrounding the tumor that creates an immunosuppressive microenvironment. The main cellular component of this fibrosis, activated pancreatic stellate cells (aPSCs), are marked by elevated expression of fibroblast activation protein (FAP). Here we investigate the relationship between FAP and the cytotoxic activity of natural killer (NK) cells.

**Methods**

To assess the relationship between aPSCs and NK cells we used a novel in vitro co-culturing system that utilizes primary donor-derived PSCs and a human NK cell line, NK92. We tested the ability of NK cells to kill aPSCs using CytotoxGlo and Annexin V assays. We monitored FAP expression and markers of activation in aPSC and NK cells using rt-qPCR, western blot and flow cytometry. To assess the effects of FAP inhibition we used a non-specific FAP inhibitor, talabostat, in vitro and in vivo. 1 μM of talabostat was added to coculturing conditions and NK lysis of aPSCs was determined. For in vivo studies forty female C57BL/6 mice were injected subcutaneously with 1X105 syngeneic MT3-2D cells (Kras+/G12D, p53+/-R172H derived from a PDAC KPC GEMM model [1]). Once tumors reached 40-50 mm3, ten mice per group were given either 30 ug of talabostat per mouse daily by oral gavage, 200 ug of anti-PD-1 per mouse twice a week by i.p., both, or neither. Control mice were treated with PBS. Treatment was terminated after 4 weeks and the mice were monitored, with tumor measurements occurring weekly.

**Results**

Here we demonstrate that the human NK cell line (NK92) is activated by and kills aPSCs, potentially via recognition of MICA/B on aPSCs by NK cell surface receptor NKG2D. Upon direct contact with PSCs, PSCs downregulate FAP expression and NK92 cells upregulate FAP. This is the first-time NK cells have been shown to produce FAP and that induction of FAP is mediated by cell-to-cell contact. Furthermore, FAP expression by NK92 cells is associated with an inactivate phenotype. FAP inhibition enhances NK92 killing of PSCs in vitro and enhances PDAC tumor clearance in vivo. The anti-tumor activity of FAP inhibition was enhanced upon addition of anti-PD-1 therapy. (Figures 1-5)

**Conclusions**

This suggests FAP functions as an NK cell immune checkpoint. FAP is expressed in NK cells after activation to attenuate cytotoxicity and can be inhibited to enhance anti-tumor immunity.

**Acknowledgements**

I'd like to acknowledge Dr. Stephen Byers and Dr. Ivana Peran for provided the PSCs and Dr. Kerry Campbell for providing the NK92 cells.

**References**

1. Boj SF, Hwang C-I, Baker LA, Chio IIC, Engle DD, Corbo V, Jager M, Ponz-Sarvise M, Tiriac H, Spector MS, et al. Organoid models of human and mouse ductal pancreatic cancer. Cell. 2015; 160:324–338.

**Ethics Approval**

This study was approved by Georgetown University’s IACUC, protocol #2016-1254

**Fig. 1 (abstract P526).**
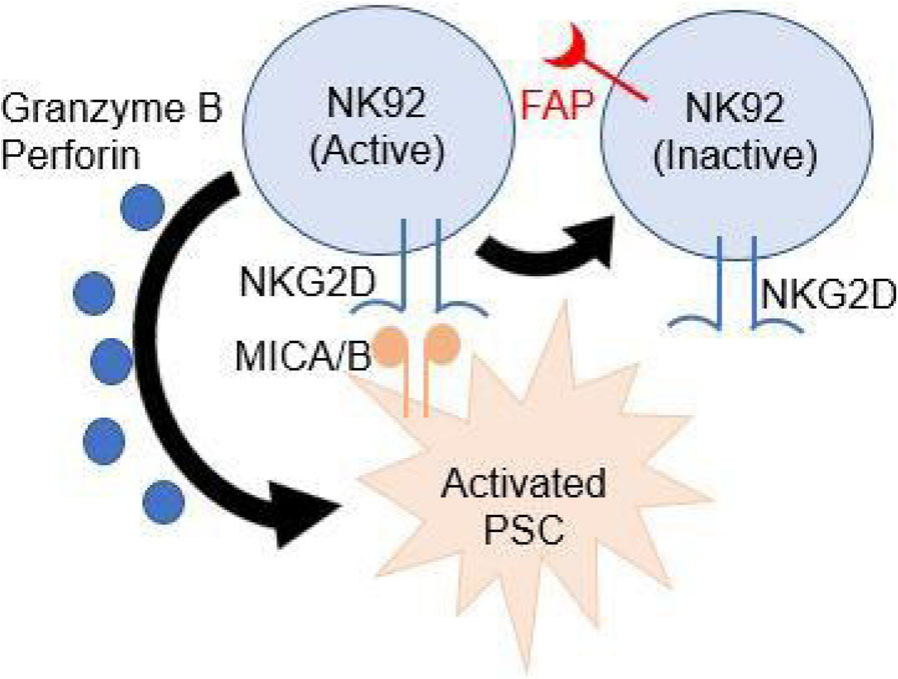
See text for description

**Fig. 2 (abstract P526).**
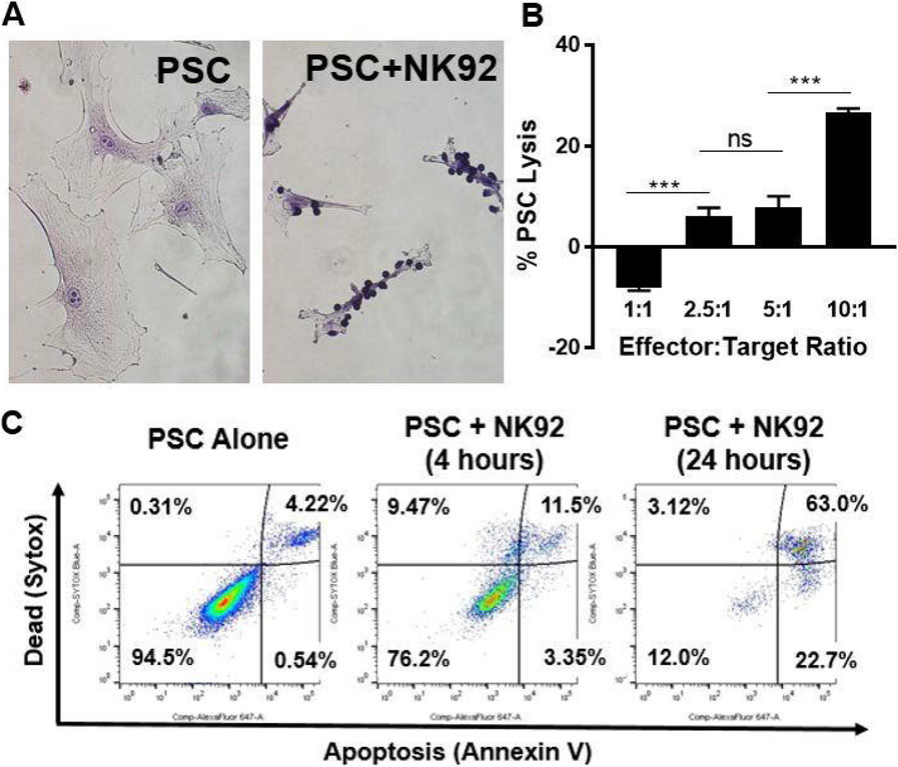
See text for description

**Fig. 3 (abstract P526).**
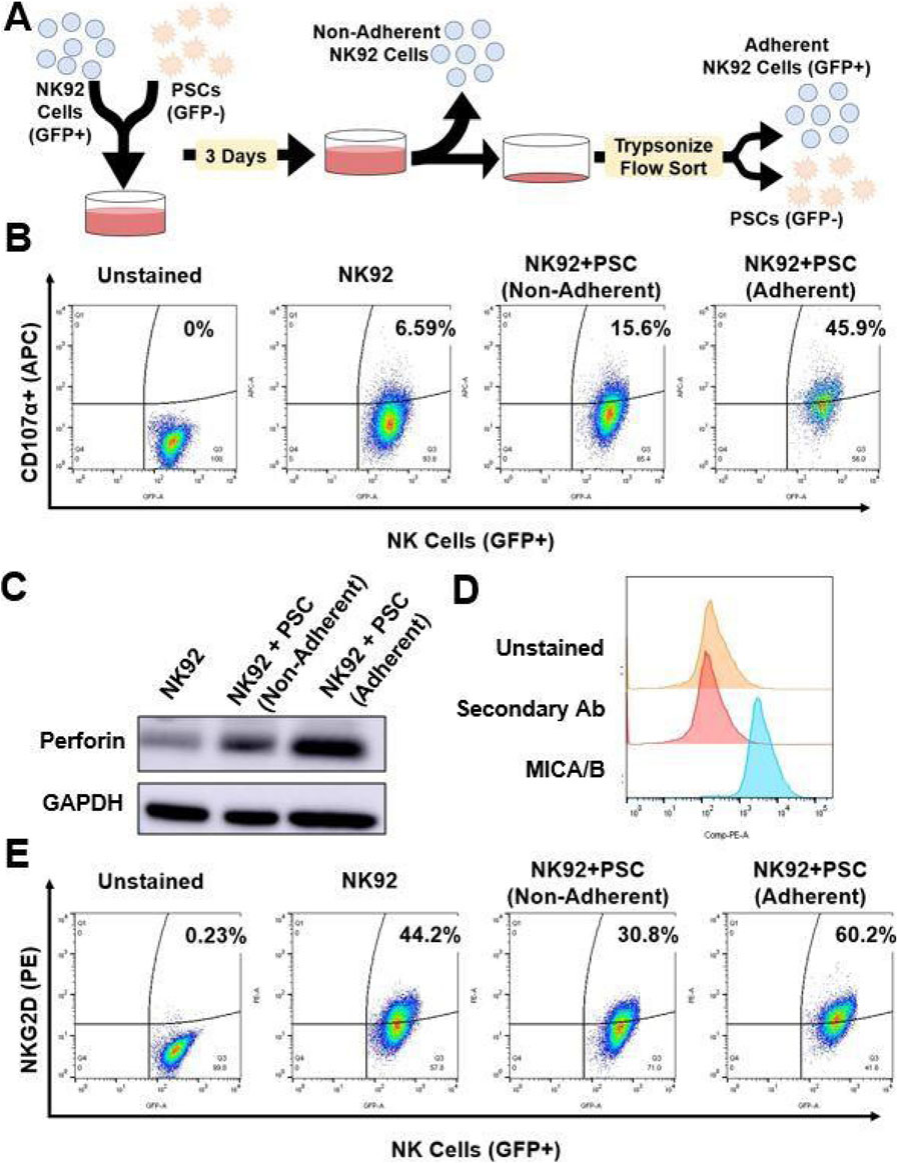
See text for description

**Fig. 4 (abstract P526).**
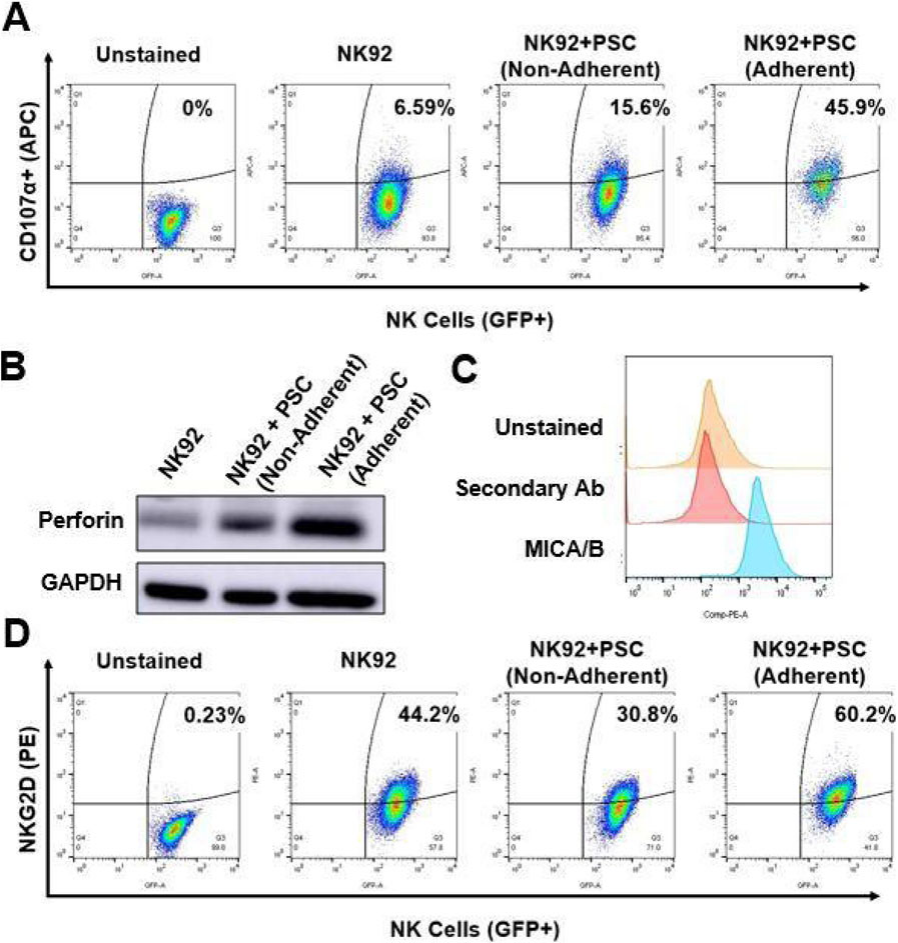
See text for description

**Fig. 5 (abstract P526).**
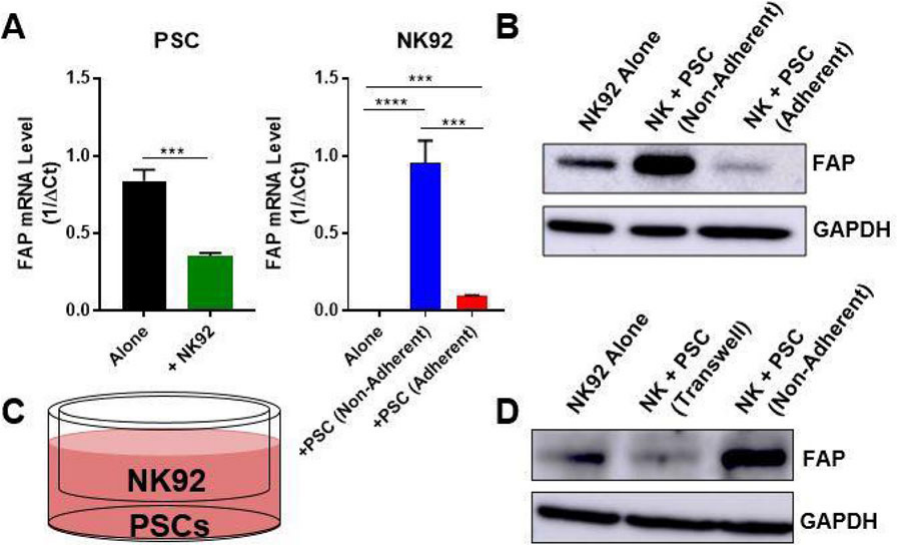
See text for description

#### P527 Imprime PGG, a novel cancer immunotherapeutic, engages the complement system to prime innate immune effector functions

##### Xiaohong Qiu, BS, Ben Harrison, MS, Adria Jonas, MS, Anissa Chan, PhD, Nadine Ottoson, BS, Nandita Bose, PhD, Keith Gorden, BS

###### Biothera Pharmaceuticals, Eagan, MN, USA

####### **Correspondence:** Keith Gorden (kgorden@biothera.com)

**Background**

Imprime PGG (Imprime), an intravenously-administered soluble, yeast β-1,3/1,6-glucan, is currently in clinical development with tumor-targeting antibodies, anti-angiogenics, and checkpoint inhibitors. The fundamental mechanistic rationale for these therapeutic combinations is that Imprime, being a PAMP, primes innate immune effector functions to ultimately inspire an adaptive immune response-based anti-cancer immunity cycle. Imprime forms a tripartite immune complex (IC) comprising of Imprime, naturally occurring anti-β-glucan antibodies (ABA) and iC3b complement opsonin in subjects with sufficient ABA levels. Ex vivo human and in vivo mouse studies have shown that the innate immune receptor, FcgRIIA, and the pattern recognition receptors, complement receptor 3 (CR3) and Dectin-1, are critical for Imprime’s innate immune responses. However, the contributions of the complement system, a vital component of innate immunity, towards the functional activity of Imprime has not been thoroughly investigated. Imprime-ABA IC activates the classical complement pathway and releases C5a. As C5a is a well-known priming agent, and cross-talks with the other innate immune receptors, we hypothesized that Imprime- induced C5a will engage the C5a-C5a receptor (C5aR) signaling pathway to enhance Imprime binding and innate immune effector functionalities.

**Methods**

The role of C5a in Imprime-ABA binding to isolated neutrophils was evaluated by: a) adding exogenous C5a; b) using C5a-depleted serum, and c) using C5aR antagonist (C5aRA). Cytokine production in healthy subjects with sufficient ABA levels were measured 24hrs post-Imprime treatment in the presence or absence of C5aRA by multiplex luminex assays. The effect of C5a inhibitors was also evaluated in a chemiluminescence-based oxidative burst assay measuring reactive oxygen species (ROS) generated by Imprime-treated isolated neutrophils in response to Rituxan-bound B cell lymphoma cells. In order to test these endpoints in complement-depleted conditions, the whole blood was washed extensively to remove the plasma.

**Results**

Addition of exogenous C5a increased the percentage of neutrophils binding to Imprime in a dose-dependent manner. Furthermore, Imprime binding in the presence of C5aRA and C5a-depleted serum was significantly reduced. Functionally, C5aRA abrogated cytokine production ( IL-8, MCP-1, MIP-1alpha, and IL-6) in Imprime-treated blood. Likewise, Imprime-ABA induced ROS in high-ABA blood was greatly inhibited in C5a-depleted serum and could be rescued by replenishing complements. C5aRA also inhibited Imprime-induced ROS production. In a non- physiological, complement-depleted condition, Imprime bound predominantly via FcgRIIA, resulting in diminished cytokine and ROS responses.

**Conclusions**

These results collectively demonstrate that Imprime-induced C5a play a critical role in enhancing Imprime binding and functional responses, potentially by lowering the signaling threshold of the other innate immune receptors.

#### P528 Tumor-derived alpha fetoprotein suppression of mitochondrial metabolism via PGC1-α and SREBP-1 expression and activity in human dendritic cells

##### Patricia Santos, PhD, Ashley Menk, BS, Jian Shi, MD, Allan Tsung, MD, Greg Delgoffe, PhD, Lisa Butterfield, PhD

###### University of Pittsburgh, Pittsburgh, PA, USA

####### **Correspondence:** Lisa Butterfield (butterfieldl@upmc.edu)

**Background**

Alpha-fetoprotein (AFP) is an oncofetal antigen expressed during fetal development and by over 50% of hepatocellular carcinomas (HCC). AFP-L3 is the major isoform present in the serum of HCC patients and is associated with poor patient prognosis. While tumor-derived AFP (tAFP) contains >80% of AFP-L3, cord blood serum-derived AFP (nAFP) contains less than 5% of AFP-L3. We have previously shown that monocyte-derived dendritic cells (DC) cultured in the presence of AFP (in particular tAFP), retained a monocyte-like morphology, had decreased expression of DC maturation markers, and are poor stimulators of antigen-specific T cell responses. In this study, the effect of AFP on DC metabolism was examined.

**Methods**

PBMC were isolated from healthy donor (HD) or HCC patients using Ficoll-Paque density gradient centrifugation. HD monocytes were isolated from PBMC and cultured for 5 days with IL-4 and GM-CSF to generate DC in the presence of 10 μg/mL ovalbumin (OVA), nAFP or tAFP. DC were collected and tested for 1) mitochondria levels and function by flow cytometry, 2) metabolic function by seahorse extracellular flux analyzer, 3) expression of oxidative phosphorylation proteins, SREBP-1 and downstream gene targets via Western Blot, and 4) expression of PGC1-α via flow cytometry. PBMC from HCC patients were stained with surface markers to identify different circulating DC subsets prior to intracellular staining with PGC1-α.

**Results**

DC cultured in the presence of nAFP and tAFP show reduced expression of mitochondrial regulator PGC1-α. Furthermore, nAFP- and tAFP-DC had reduced mitochondrial mass and mitochondrial activity compared to OVA- DC. This was confirmed by a reduction in the basal oxygen consumption rate (OCR) in nAFP-DC and a more severe reduction in basal OCR in tAFP-DC, with changes in DC metabolism occurring within 24 hours of AFP exposure.

The decrease in oxygen consumption in DC exposed to nAFP and tAFP is attributed to downregulation of cytochrome c oxidase, responsible for the reduction of oxygen into water. Importantly, circulating myeloid DC from HCC patients have reduced PGC1-α expression compared to healthy donors. Lastly, there was a reduction in the expression of the transcription factor SREBP-1 and downstream targets FASN and ACLY in DC exposed to nAFP and tAFP, suggesting mechanistic inhibition of mTORC1 pathway in DC by AFP.

**Conclusions**

Collectively, these data show the profound negative effects of AFP on DC metabolism. These novel findings elucidate a key mechanism of immune suppression in HCC and may lead to new therapeutic approaches to reverse these effects.

**Ethics Approval**

The study was approved by the University of Pittsburgh's Institutional Review Board, approval number 0403105.

#### P529 Efficacy and safety profile of AU7R-104, a small molecule targeting CD47/SIRPα pathway

##### Murali Ramachandra, PhD^2^, Pottayil Sasikumar, PhD^1^, Chennakrishnareddy Gundala^2^, Wesley Balasubramanian, PhD^2^, Sudarshan Naremaddepalli, PhD^2^, Archana Bhumireddy, MSc^2^, Sandeep Patil, PhD^2^, Amit Dhudashiya^2^, Vijaysai Rayavarapu, MSc^2^, Dodheri Samiulla, PhD^2^, Sanjeev Giri, PhD^2^, Rajesh Eswarappa, PhD, DABT, ERT^2^, Kiran Aithal, PhD^2^, Girish Daginakatte, PhD^2^, Murali Ramachandra, PhD^2^

###### ^1^Aurigene Discovery Technologies, Bangalore, India; ^2^Aurigene Discovery Technologies Limited, Bangalore, India

####### **Correspondence:** Murali Ramachandra (murali_r@aurigene.com)

**Background**

CD47 is over expressed on many different human cancers and it is also known as a “don’t eat me” signal. Many studies have demonstrated that there is great potential for targeting the CD47-SIRPα pathway as therapy for cancer. Efforts have been made to develop therapies inhibiting the CD47-SIRPα pathway, through antibodies directed against CD47 and recombinant SIRPα proteins. We have developed a novel small molecule CD47 antagonist, AU7R-104, as therapeutic agent for solid and hematological cancers. AU7R-104 enhances phagocytosis of tumor cells and exhibits good drug-like properties with good anti-tumor activity. Here, we report the in vivo activity of AU7R-104 in different tumor models, biomarker characterization and safety profile of AU7R-104 in rodents and non-rodents.

**Methods**

We have identified preclinical candidate compound AU7R-104 with potent in vitro and in vivo activity. AU7R-104 was profiled extensively in different tumor models both as single agent and in combination with tumor specific antibodies and other anti-cancer agents. In the PK-PD and efficacy studies, efforts were made for biomarker characterization through multiplex and FACS analysis. Advanced profiling of AU7R-104 has been completed in DMPK and toxicological studies in rodents and non-rodents.

**Results**

AU7R-104 has potent anti-tumor activity both as a single agent and in combination with anti-cancer agents. In the PK-PD studies, AU7R-104 enhanced in vivo phagocytosis in both macrophages and dendritic cells. Multiplex analysis of serum samples indicated there was modulation of macrophage and T-cell mediated cytokines. In the advanced ADME assays, AU7R-104 demonstrated good drug-like properties without any significant alerts. AU7R- 104 combination treatments were well tolerated. Preliminary safety evaluation of AU7R-104 in both rodents and non-rodents indicated the lack of safety concerns typically associated with anti-CD47 antibodies or SIRPα-Fc protein therapeutics.

**Conclusions**

The above findings support further development of these orally bioavailable agents for use in the clinic

#### P530 Novel bispecific antibody targeting NKp30 receptor enhances NK-mediated killing activity against multiple myeloma cells and overcomes CD16A deficiency

##### Monia Draghi, PhD^1^, Jennifer Watkins-Yoon^1^, Jamie Schafer, PhD^1^, Sara Haserlat^1^, Sri Vadde^1^, Xin Kai^1^, Allison Nelson^1^, Lucy Liu^1^, Nora Zizlsperger, PhD^1^, Amanda Oliphant^1^, Michael Schmidt^1^, Robert Tighe, BS^2^,

###### ^1^Compass Therapeutics, Cambridge, MA, USA; ^2^Compass Therapeutics LLC, Cambridge, MA, USA

####### **Correspondence:** Monia Draghi (monia.draghi@compasstherapeutics.com)

**Background**

Multiple myeloma (MM) is a malignant hematological disease characterized by a dysregulated growth of malignant plasma cells. Different therapeutic options are available for MM patients; however, the disease remains mostly incurable. B-cell maturation antigen (BCMA) is a promising target in MM because of its restricted expression in normal and malignant plasma cells [1]. NK cells have been implicated in the clinical efficacy of several therapies against MM and may contribute to the success of stem cell transplantation (SCT) by clearing residual cancer cells [2]. In patients with advanced MM, NK cell function is impaired by downregulation of activating receptors including NKG2D, 2B4, and CD16A (FcγRIIIA) [3,4]. Downregulation of CD16A is particularly problematic for conventional anti-BMCA antibodies seeking to elicit ADCC. In contrast, expression of NKp30 remains stable, providing a compelling rationale for the design of BCMA-targeted multispecific molecules that redirect NK cell killing by engaging NKp30 to overcome deficiencies in other activating NK receptors.

**Methods**

Our Stitchmabs™ and common light chain (LC) bispecific antibody platforms were employed to discover and engineer CTX-4419, a tetravalent, fully human, bispecific molecule consisting of a novel IgG1 antibody recognizing BCMA fused at the c-terminus to two anti-NKp30 Fab fragments with a common LC sequence. The in vitro activity of CTX-4419 was compared against a conventional anti-BCMA IgG1 antibody format for its capacity to induce killing of MM cells and cytokine and chemokine production by primary NK cells and NK cell lines.

**Results**

When tested with primary NK cells expressing both CD16A and NKp30, CTX-4419 induced potent NK cell cytotoxicity and cytokine production against tumor cells which was superior to the monoclonal IgG1 anti-BCMA control. In contrast to the anti-BCMA IgG1 control that activated only CD16A+ NK cells, CTX-4419 induced potent killing of MM cells and IFN-γ production by KHGY-1, a CD16A- NKp30+ cell line, showing that CTX-4419 can redirect NK cell subsets with low expression of CD16A to kill MM cells. Additionally, an Fc-silent version of CTX-4419 retained activity, further supporting a CD16A-independent function. Importantly, CTX-4419 did not activate NK cells in the absence of BCMA-expressing tumor cells, indicating no off-target effects.

**Conclusions**

We have engineered and characterized a first-in-class, differentiated bispecific NK cell engager that potently redirects NKp30+ NK cells to kill BCMA+ tumor cells. Unlike traditional anti-BCMA mAbs, CTX-4419 remains highly active in the absence of CD16A engagement. CTX-4419 in undergoing monotherapy assessment in pre- clinical models including experiments with patient derived samples.

**References**

1. Tai YT, Anderson KC. Targeting B-cell maturation antigen in multiple myeloma. Immunotherapy. 2015;7(11):1187-99.
2. Cooley S, Parham P, Miller JS. Strategies to activate NK cells to prevent relapse and induce remission following hematopoietic stem cell transplantation. Blood. 2018;131(10):1053-1062.
3. Fauriat C, Mallet F, Olive D, Costello RT. Impaired activating receptor expression pattern in natural killer cells from patients with multiple myeloma. Leukemia. 2006;20(4):732-3.
4. Costello RT, Boehrer A, Sanchez C, et al. Differential expression of natural killer cell activating receptors in blood versus bone marrow in patients with monoclonal gammopathy. Immunology. 2013;139(3):338-41.

#### P531 Redirecting immunometabolic suppression by targeting purinergic signaling enhances immunotherapy of solid tumors with CAR-NK cells

##### Jiao Wang, PhD, Sandro Matosevic, PhD, Kyle B. Lupo, Andrea M. Chambers

###### Purdue University, West Lafayette, IN, USA

####### **Correspondence:** Sandro Matosevic (sandro@purdue.edu)

**Background**

Engineering natural killer (NK) cells with chimeric antigen receptors (CARs) can enhance their targeting of solid tumor malignancies [1]. However, the CD73-mediated generation of adenosine (ADO) within the hypoxic microenvironment of many solid tumors induces severe immunometabolic suppression which impairs their activation and anti-tumor immunity (Figure 1A) [2–4].

**Methods**

In an effort to overcome purinergic immunometabolic suppression and improve NK cell-mediated anti-tumor function, we have developed an immunotherapeutic treatment approach which combines non-virally engineered NKG2D-retargeted CAR-NK-92 (NKG2D.CAR-NK-92) cells alongside neutralization of CD73 ectonucleotidase activity (Figure 1B). We have quantified the cytotoxicity of these engineered cells in terms of IFN-γ expression and degranulation activity, and characterized their anti-tumor effects and homing of on CD73+ solid tumors in vivo.

**Results**

CAR-NK-92 cells, expressing a chimeric immunoreceptor targeting NKG2D based on the piggyBac transposon system, showed significantly higher IFN-γ production, degranulation capacity, and lytic ability against solid tumor cells compared with wild-type NK cells. CD73 blockade was able to further enhance the killing ability of CAR- engineered NK cells against CD73+ solid tumor targets. In vivo, neutralization of CD73 activity promoted anti- tumor efficacy of the engineered NK cells against CD73+ human lung cancer xenografts, including a greater delay in tumor growth, no obvious toxicity, and increased tumor-infiltrating NK cells (Figure 1C-G). CD73 blockade could contribute to the delay in tumor growth in vivo independently of adaptive immune cells, innate immunity or NK cell-mediated ADCC, suggesting that CD73 might contribute to tumor metastasis via autocrine-like mechanisms outside of its ectonucleotidase activity.

**Conclusions**

Immunometabolism is emerging as a profound mediator of NK cell anti-tumor immunity. Immunotherapies targeting the adenosinergic signaling pathway, such as by neutralizing CD73 ectoenzymatic activity, had, however, not been evaluated on NK cells. Our studies demonstrate, for the first time, the potential of targeting CD73 to modulate purinergic signaling and enhance adoptive NK cell immunotherapy via mechanisms that could implicate autocrine tumor control as well as by mediating adenosinergic signaling. We also provided the basis for targeting the regulation of cancer metabolism as a promising strategy for enhancing the therapeutic efficacy of CAR-modified NK cells for immunotherapy of solid tumors. Based on these results, in order to achieve affective redirection of purinergic signaling while enhancing cancer targeting specificity, we are currently designing and characterizing a multi-functional CAR-NK cell which consists of a single chain antibody targeting CD73 alongside dual chimeric antigen receptor targeting, as a next-generation, single-agent immunometabolic therapy of solid tumors with NK cells.

**References**

1. Guillerey C, Huntington ND, Smyth MJ. Targeting natural killer cells in cancer immunotherapy. Nat Immunol. 2016;17(9):1025-36.
2. Zhang B. CD73: a novel target for cancer immunotherapy. Cancer Res. 2010;70(16):6407–11.
3. Antonioli L, Blandizzi C, Pacher P, Haskó G. Immunity, inflammation and cancer: a leading role for adenosine. Nat Rev Cancer. 2013;13(12):842-57.
4. Ohta A. A metabolic immune checkpoint: adenosine in tumor microenvironment. Front Immunol. 2016;7:109.

**Fig. 1 (abstract P531).**
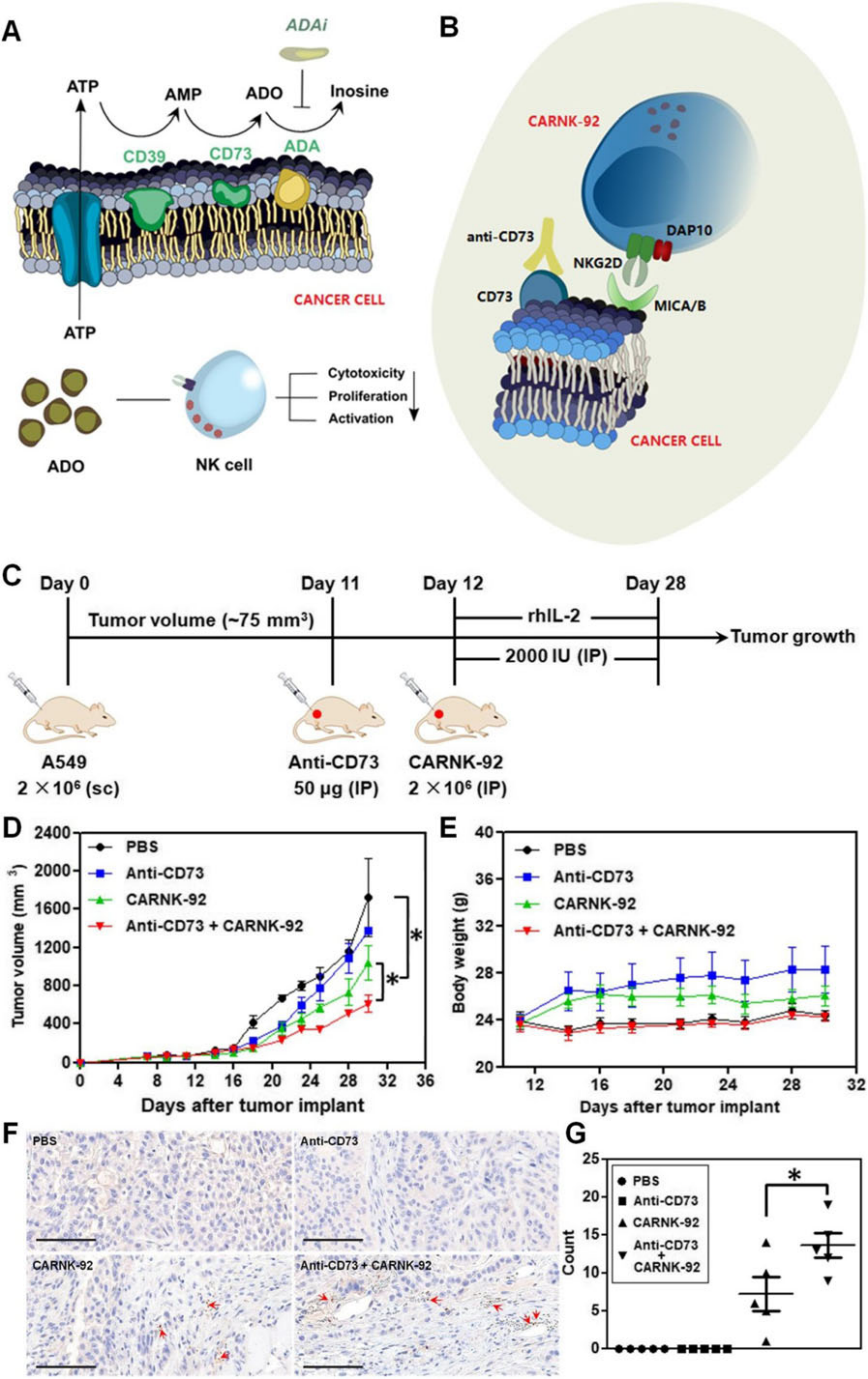
See text for description

#### P532 Preclinical characterization of a first-in-class ILT4 antagonist, MK-4830

##### Luis Zuniga, PhD^1^, Barbara Joyce-Shaikh, BS^1^, Douglas Wilson^1^, Holly Cherwinski^1^, Yi Chen^1^, Grein Jeff^1^, Wendy Blumenschein, BA^1^, Eric Muise, MS^1^, Xiaoyan Du^1^, Edward Hsieh^1^, Sripriya Dhandapani^1^, Gulesi Ayanoglu^1^, Maribel Beaumont^1^, Shuli Zhang^1^, Michael Rosenzweig, DVM, PhD^1^, Robert Kastelein, PhD^1^, Robert Stein, MD PhD^2^, Dennis Underwood, PhD^2^, Milan Blanusa^3^, Rachel Altura, MD^1^, Daniel Cua^1^

###### ^1^Merck Research Laboratories, Palo Alto, CA, USA; ^2^Agenus Inc., Lexington, MA, USA; ^3^Pieris Pharmaceuticals GmbH, Freising, Germany

####### **Correspondence:** Luis Zuniga (luis.zuniga@merck.com)

**Background**

Myeloid-derived suppressor cells in the tumor microenvironment contribute to tumor immune evasion by suppressing local T cell activation, proliferation, and anti-tumor effector responses, identifying them as targets for therapeutic intervention. ILT4 is an inhibitory member of the immunoglobulin-like transcript (ILT) family of proteins. It is expressed primarily by myeloid cells, including those in the tumor microenvironment, and interacts with major histocompatibility (MHC) class I complexes and angiopoietin-like (ANGPTL) ligands. ILT4 signaling is associated with the induction of a tolerogenic phenotype in antigen presenting cells. We demonstrate the pre-clinical anti-tumor properties of a first-in-class anti-ILT4 monoclonal antibody which has now entered clinical trials in patients with advanced solid tumors.

**Methods**

The clinical candidate MK-4830 is a fully human monoclonal antibody that we selected for specificity, ligand blockade, and functional downstream signaling antagonism of ILT4. Primary human tumor tissue, blood, and serum were profiled for the expression of ILT4 and its ligands. Primary human PBMCs were used to discover MK-4830- dependent, myeloid-associated cytokine responses in vitro. A humanized mouse model implanted with a patient- derived melanoma cell line (SK-MEL-5) was used to evaluate the mechanism of action of ILT4 antagonism and its anti-tumor efficacy.

**Results**

MK-4830 is specific to ILT4 and does not bind other ILT-family receptors. MK-4830 blocks ILT4 ligand binding and reverses ILT4-mediated suppression of signal transduction. Blocking ILT4 in vitro enhances proinflammatory cytokine expression of GM-CSF and TNFα in LPS-stimulated human PBMC cultures. ILT4 is expressed in primary human tumor samples and ILT4+ myeloid cells are observed both in the periphery and in the tumor infiltrate within the humanized mouse tumor model. Administration of MK-4830 in the humanized mouse tumor model resulted in approximately 50% reduction in tumor growth, alterations in both splenic and tumor myeloid subset distributions, as well as changes in myeloid-centric chemokine and cytokine profiles.

**Conclusions**

MK-4830, a novel first-in-class antagonist ILT4 antibody, induces robust anti-tumor activity in a humanized mouse tumor model. The preclinical data presented here support the ongoing clinical evaluation of MK-4830 as an anti- cancer therapy and suggests its potential to target tumor-associated myeloid cells in combination with other immune checkpoint blockers.

**Trial Registration**

Study of MK-4830 as Monotherapy and in Combination With Pembrolizumab (MK-3475) in Participants With Advanced Solid Tumors (MK-4830-001). (2018). Retrieved from https://clinicaltrials.gov/ct2/show/results/NCT03564691?term=NCT03564691&rank=1 (Identification No. NCT03564691)

**Ethics Approval**

In vivo experiments used in this study were approved by Merck Research Laboratories’ Ethics Board, approval number P2021-400265-JAN.

#### P533 Gastrointestinal symptoms observed after chimeric antigen receptor T–cell therapy

##### Hamzah Abu-Sbeih, MD, Tenglong Tang, MD, Jason R. Westin, MD, David Richards, MD, Sattva S. Neelapu, MD, Yinghong Wang, MD, PhD

###### MD Anderson Cancer Center, Houston, TX, USA

####### **Correspondence:** Yinghong Wang (ywang59@mdanderson.org)

**Background**

The new approach of chimeric antigen receptor T-cell therapy (CAR-T) has been proven to be a very effective treatment for hematological malignancies.[1, 2] The most notable drawbacks of CAR-T is cytokine release syndrome (CRS) and CAR-related encephalopathy syndrome (CRES).[3-6] Gastrointestinal adverse events (GI- AEs) associated with CAR-T have not been studied yet. Herein, we describe the incidence and features of GI-AEs observed after CAR-T.

**Methods**

This is a case series of patients with hematological malignancies who received CAR-T, as a clinical trial or standard of care where data publication was permitted by the primary investigators, and subsequently suffered from GI-AEs between 1/2012 and 5/2018. Other etiologies of diarrhea were excluded (Figure 1).

**Results**

Out of the 132 patients that received CAR-T, 21 (16%) experienced GI-AEs. The median age for the 21 patients was 59 years (range, 23-77; Table 1). Most patients had diffuse large B-cell lymphoma (67%). Ten patients experienced CRS, whereas, 7 experienced CRES. Interleukin-6 antagonist was required in 10 patients. Diarrhea was present in all 21 patients (Table 2); 62% grade 1, 33% grade 2 and 5% grade 3. Other associated gastrointestinal symptoms among these 21 were abdominal pain (38%), nausea and vomiting (38%), fever (38%), abdominal distension (10%), and bloody stool (5%). The median duration from CAR-T infusion to diarrhea onset was 5 days (range, 1-40). Eleven patients required treatment for GI-AEs with a median duration of 6 days. Sixteen patients had abdominal imaging evaluation; 3 (19%) of them had findings suggestive of gastrointestinal tract inflammation. Three (14%) patients experienced GI-AEs recurrence after improvement initially. Colitis was confirmed endoscopically in 1 patient; a 76 year old male who received 2 infusions of CAR-T. Five months later, he developed grade 3 diarrhea with abdominal cramps and 15 pounds weight loss. A stool infectious workup including PCR-based multiplex was negative. Colonoscopy demonstrated diffuse inflammation of the entire colon with histology showing glandular drop out, increased apoptosis, and focal erosions. He had no improvement with steroids and mesalamine, and was subsequently treated with oral immunoglobulin with partial improvement. However, his colitis relapsed and an additional trial of cholestyramine was unsuccessful. Lastly, a complete resolution of his gastrointestinal symptoms was achieved by an antibiotics course (vancomycin and piperacillin/tazobactam) for his new onset of pneumonia.

**Conclusions**

GI-AEs occur in 16% of patients receiving CAR-T. They are typically mild and self-limiting requiring only symptomatic treatment. Nevertheless, of a rare occurrence, it could lead to a refractory colitis.

**References**

1. Sadelain M, Brentjens R, Riviere I. The basic principles of chimeric antigen receptor design. Cancer Discov. 2013;3(4): 388-98.
2. Sadelain M. CAR therapy: the CD19 paradigm. J Clin Invest. 2015;125(9):3392-400.
3. Magee M.S., Snook A.E. Challenges to chimeric antigen receptor (CAR)-T cell therapy for cancer. Discov Med. 2014;18(100):265-71.
4. Grigor E.J.M., et al., Efficacy and safety of chimeric antigen receptor T-cell (CAR-T) therapy in patients with haematological and solid malignancies: protocol for a systematic review and meta-analysis. BMJ Open, 2017. 7(12): p. e019321.
5. Leick M.B., Maus M.V. Toxicities associated with immunotherapies for hematologic malignancies. Best Pract Res Clin Haematol. 2018;31(2):158-165.
6. Neelapu S.S., et al. Chimeric antigen receptor T-cell therapy - assessment and management of toxicities. Nat Rev Clin Oncol. 2018;15(1):47-62.

**Ethics Approval**

This case series was approved by the Institutional Review Board at The University of Texas MD Anderson Cancer Center (IRB No.: PA18-0472).

**Consent**

This case series was granted waiver of consent.

**Fig. 1 (abstract P533).**
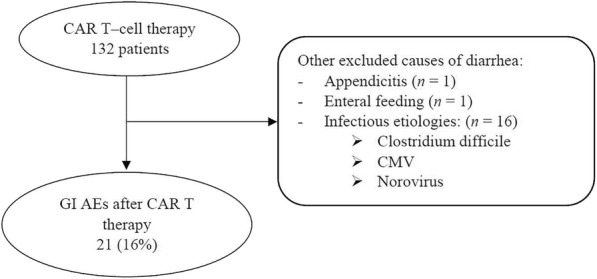
Included patients

**Table 1 (abstract P533).**
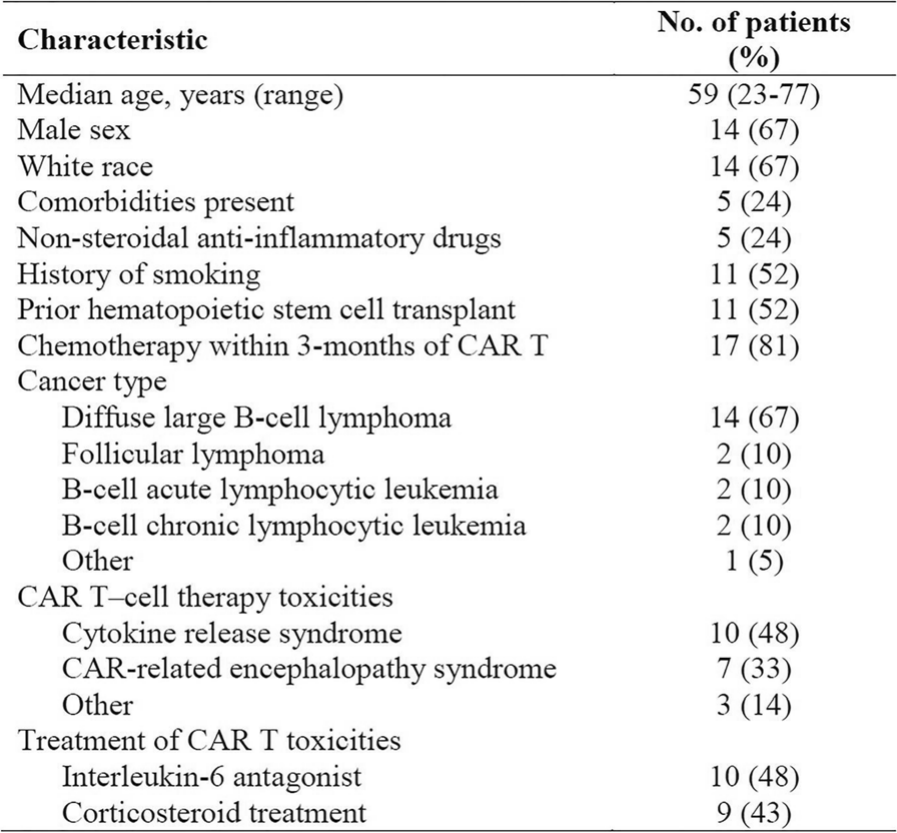
Patient characteristics (Number of patients = 21)

**Table 2 (abstract P533).**
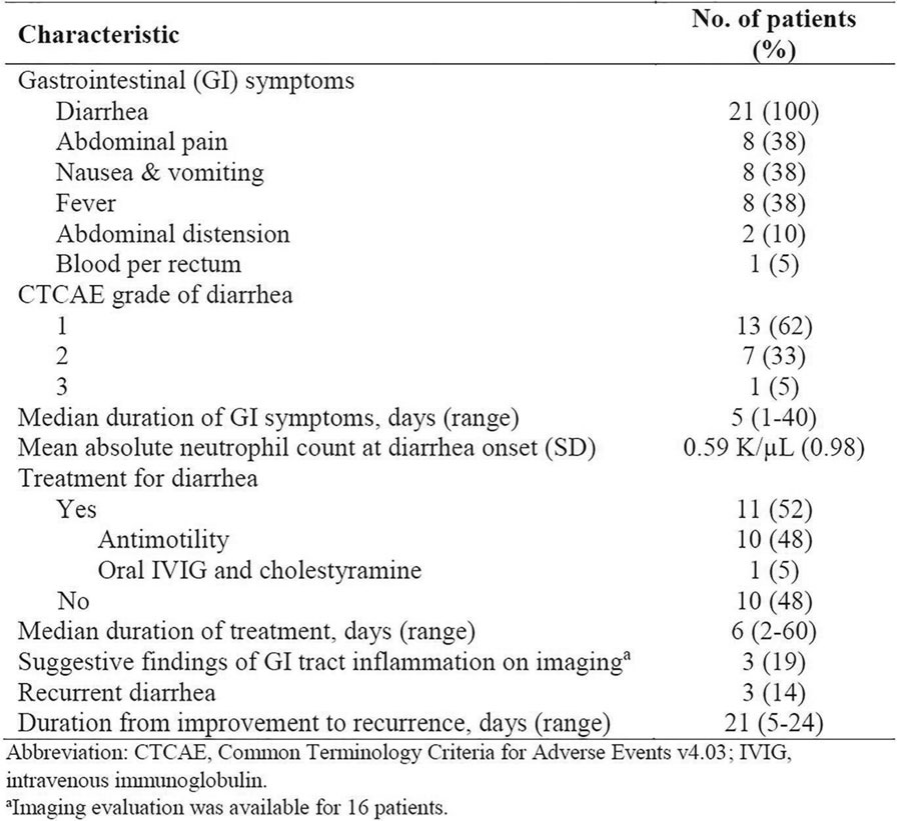
Characteristics of gastrointestinal adverse events observed in our cohort

#### P534 Timely endoscopic and histological evaluation is critical to provide appropriate management for immune checkpoint inhibitor induced colitis

##### Hamzah Abu-Sbeih, MD, Faisal S. Ali, Wenyi Luo, MD, Wei Qiao, PhD, Gottumukkala S. Raju, MD, Yinghong Wang, MD, PhD

###### MD Anderson Cancer Center, Houston, TX, USA

####### **Correspondence:** Yinghong Wang (ywang59@mdanderson.org)

**Background**

mmune checkpoint inhibitors (ICI) are efficacious treatments for advanced malignancies but can result in immune mediated diarrhea and colitis (IDC). Currently, the guidelines for the treatment of IDC depend only on clinical symptoms. Endoscopic and histologic features of such adverse events are not well studied in a manner that can help to gauge treatment plans. We aimed to characterize endoscopic and histologic features of IDC and to assess their association with clinical outcomes.

**Methods**

IOur study included patients who had undergone endoscopy for IDC (1/2010 to 4/2018). Patients with GI infection at time of onset were excluded. High-risk endoscopic features are ulcers deeper than 2mm, larger than 1cm, and extensive colonic involvement. Univariate and multivariate logistic regression were performed to assess the association of endoscopic and histological features with clinical outcomes.

**Results**

IA total of 182 patients was included; most were white (92%), males (65%) with a mean age of 60 years. Median time from ICI initiation to IDC was 7 weeks. Fifty-three percent had grade 3–4 diarrhea, and 32% grade 3–4 colitis. One-hundred forty-one patients received immunosuppressant therapy, and 41 received symptomatic therapy only (Table 1). Forty-nine patients had mucosal ulcerations, 66 non-ulcerative inflammation and 67 normal endoscopy. Calprotectin was higher in patients with ulceration (P=0.04). The sensitivity of lactoferrin to detect histologic and endoscopic inflammation was 90% and 70% respectively. Patients who underwent endoscopy >30 days of symptom onset required longer duration of steroids (P=0.02), had more recurrent symptoms (P<0.01) and received later infliximab/vedolizumab add-on therapy than did those who underwent endoscopy ≤30 days (P=0.03; Table 2). High-risk features were associated with more frequent (P=0.03) and longer duration (P=0.02) hospitalization and infliximab/vedolizumab requirement (P<0.01; Table 3). Patients with active histological inflammation had more recurrence (P<0.01) and repeat endoscopy (P<0.01; Table 4). Repeat endoscopy was required in 47 patients. A multivariate logistic regression revealed that longer ICPI treatment was associated with more frequent hospitalizations (OR 1.00; 95%CI 1.00-1.01; P<0.01; Table 5) and high-risk endoscopic features were associated with the requirement of infliximab/vedolizumab (OR 3.89; 95%CI 1.68-9.01; P<0.01).

**Conclusions**

IHigh risk endoscopic features and active histologic inflammation represent important markers of disease severity with clinical implications and should be used in a timely manner to devise IDC-focused treatment algorithms that incorporate a more intricate degree of specificity to improve upon the currently available guidelines.

**Ethics Approval**

This retrospective, single-center study was approved by the Institutional Review Board at The University of Texas MD Anderson Cancer Center (IRB No. PA18-0472).

**Consent**

This study was granted waiver for consent.

**Table 1 (abstract P534).**
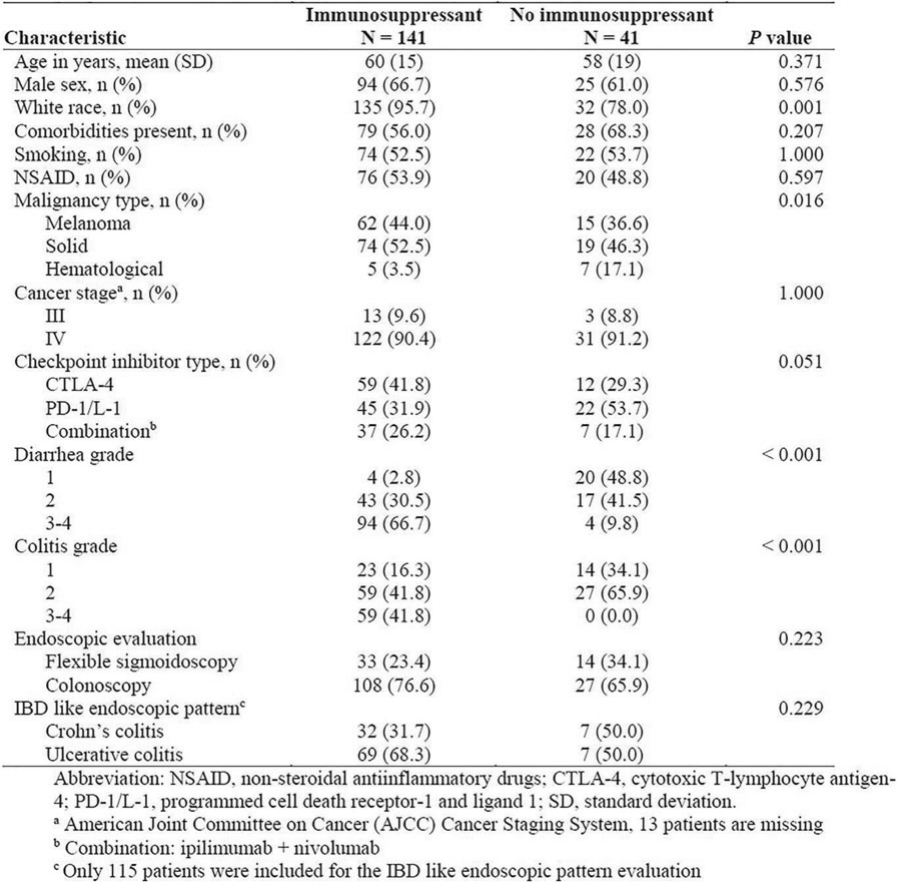
Association between patient characteristics and treatment group

**Table 2 (abstract P534).**
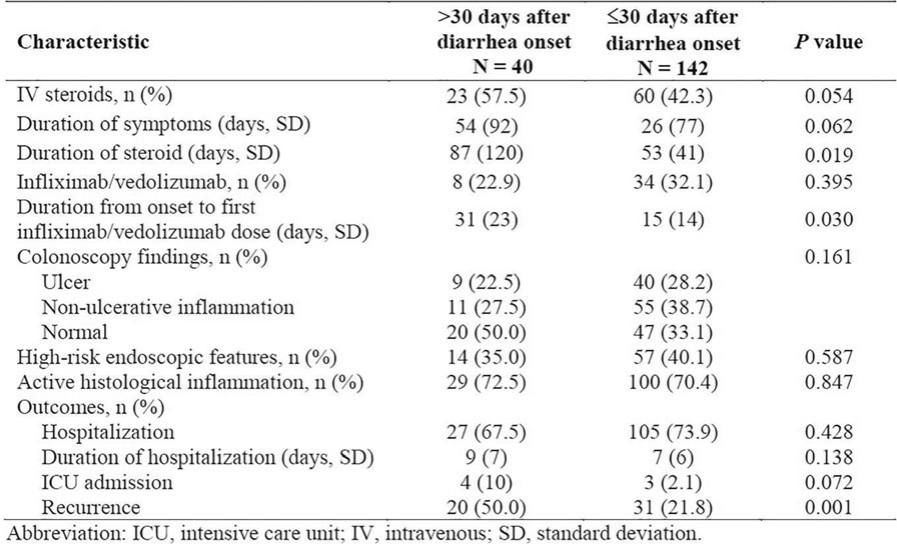
Clinical outcomes of patients according to the timing of endoscopy from IDC onset

**Table 3 (abstract P534).**
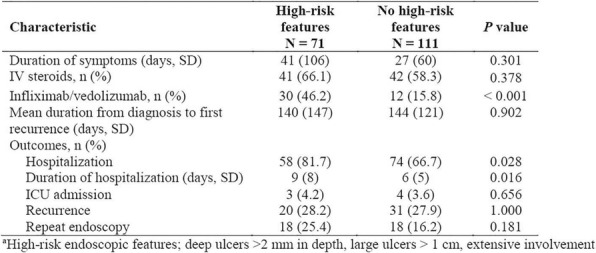
Patient with endoscopic inflammation involvement

**Table 4 (abstract P534).**
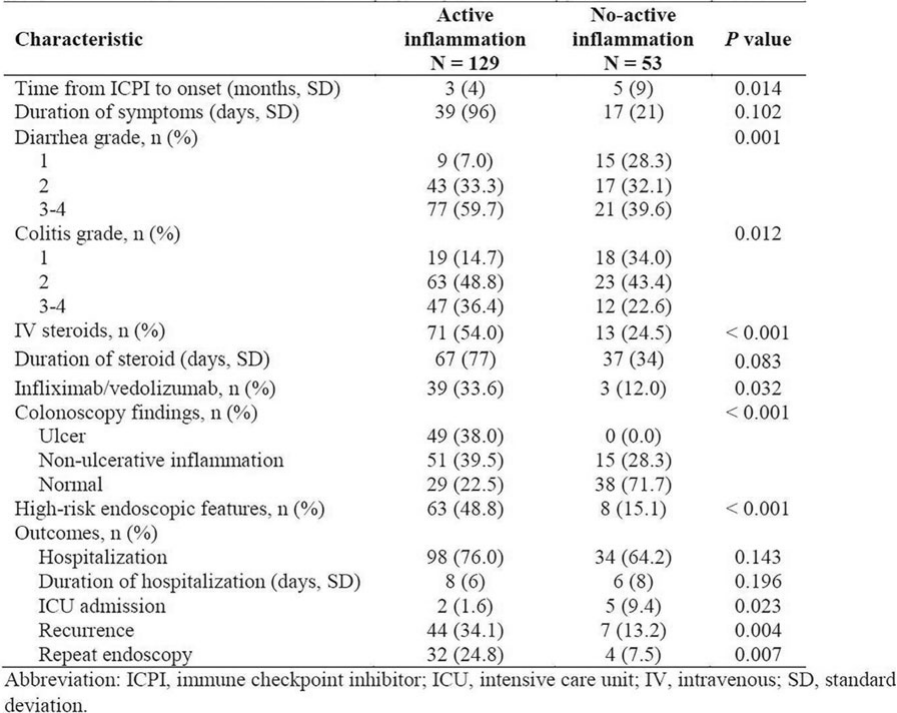
Association between histological active inflammation and clinical characteristics

**Table 5 (abstract P534).**
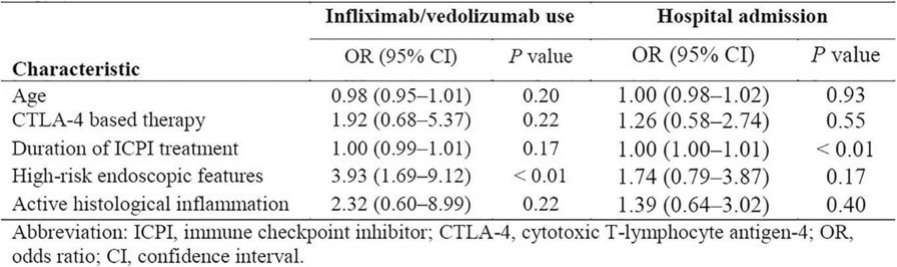
Multivariate logistic regression analysis of infliximab/vedolizumab use and hospital admission

#### P535 Upper gastrointestinal symptoms and associated endoscopic and histologic features in patients receiving immune checkpoint inhibitors

##### Hamzah Abu-Sbeih, MD, Tenglong Tang, MD, Wenyi Luo, MD, Wei Qiao, MD, David Richards, MD, Yinghong Wang, MD, PhD

###### MD Anderson Cancer Center, Houston, TX, USA

####### **Correspondence:** Yinghong Wang (ywang59@mdanderson.org)

**Background**

Immune checkpoint inhibitors (ICPIs) have demonstrated high effectiveness in treating many types of malignancies. Gastrointestinal (GI) immune-related adverse events (irAE) are commonly reported, however, limited literature describes upper gastrointestinal tract toxicity. Therefore, we aimed to describe clinical, endoscopic and histological characteristics of upper GI tract injury related to ICPI treatment.

**Methods**

We studied consecutive patients who received ICPIs between April 2011 and March 2018 and developed upper GI symptoms that required esophagogastroduodenoscopy (EGD). Patients with Helicobacter pylori gastritis were excluded from our study. We performed descriptive statistical analysis using means and standard deviations for continuous variables and frequencies and percentages for categorical variables.

**Results**

Sixty patients developed upper GI symptoms between ICPI initiation and 6 months after the last infusion (Table1); majority were of white race with a mean age of 59 years. In our cohort, 42 patients had other risk factors of gastritis such as chemotherapy, radiotherapy, and non-steroidal anti-inflammatory drugs (Table2). Patients without these risk factors had isolated gastric involvement on endoscopy. Overall, histologic inflammation of the stomach was evident in 83% of patients, and inflammation of the duodenum was evident only in 38% of patients. The rate of ulceration was the same in the cohorts with and without other risk factors for gastritis (11% vs. 12%). Among patients who had both upper and lower endoscopic evaluation (n=38), 17 (45%) had histological inflammation involving upper GI tract only; these patients developed GI toxicity later than patients with GI toxicity involving both upper and lower (P=0.060; Table3). Isolated upper GI tract involvement was more frequent in patients undergoing anti-PD-1/L1 treatment (P=0.071). Likewise, isolated upper GI toxicity was associated with more frequent mucosal ulceration (P=0.02; Table4). Patients with concurrent upper and lower GI tract involvement received immunosuppressive therapy more often than did patients with isolated upper GI tract involvement. Majority of the isolated upper GI symptoms were treated with proton pump inhibitors and H2 blockers, with less immunosuppressant use.

**Conclusions**

Overall ICPI-related upper GI-toxicities had gastric involvement more often than duodenal involvement on endoscopic and histological level, which is also observed more in patients treated with PD-1/L1. Mucosal ulcerations were more frequently found in isolated upper GI toxicity than concurrent upper and lower GI toxicities. Patients without other risk factors for gastritis had isolated gastric involvement on endoscopy, with duodenal inflammation in 39% of patients histologically. Concurrent GI tract involvement required immunosuppressive therapy more often than isolated upper GI tract involvement.

**Ethics Approval**

This retrospective, single-center study was approved by the Institutional Review Board at The University of Texas MD Anderson Cancer Center (IRB No. PA18-0472).

**Consent**

This study was granted waiver for consent.

**Table 1 (abstract P535).**
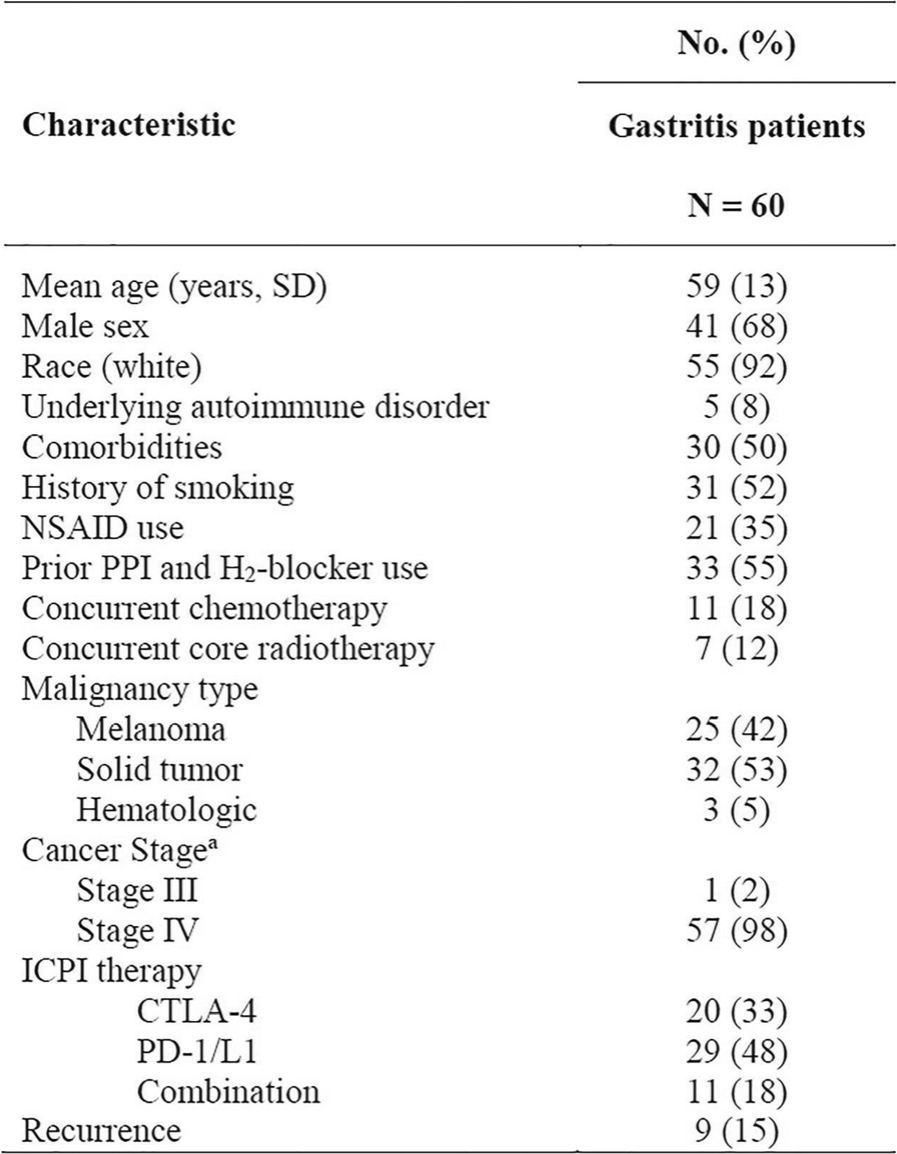
Patient baseline characteristics

**Table 2 (abstract P535).**
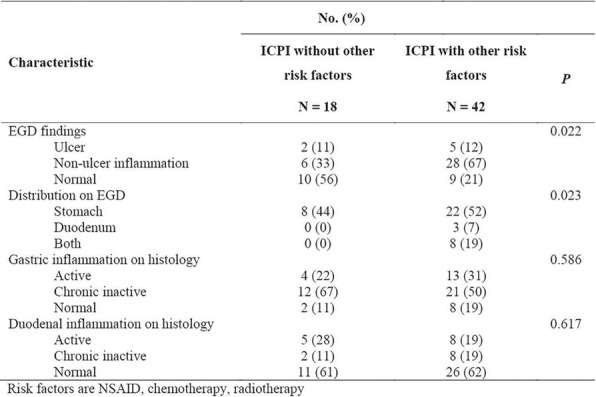
Comparison of EGD characteristics between patient who received ICPI and had other risk factors and those who received ICPI and had no other risk factors

**Table 3 (abstract P535).**
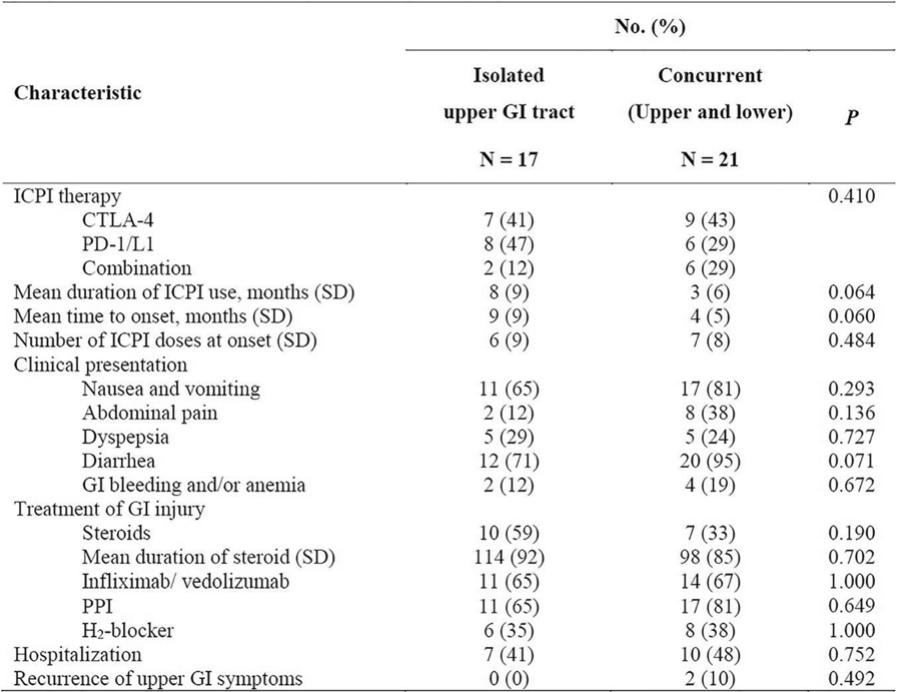
Clinical characteristics according to the endoscopic involvement of GI tract (*n* = 38)

**Table 4 (abstract P535).**
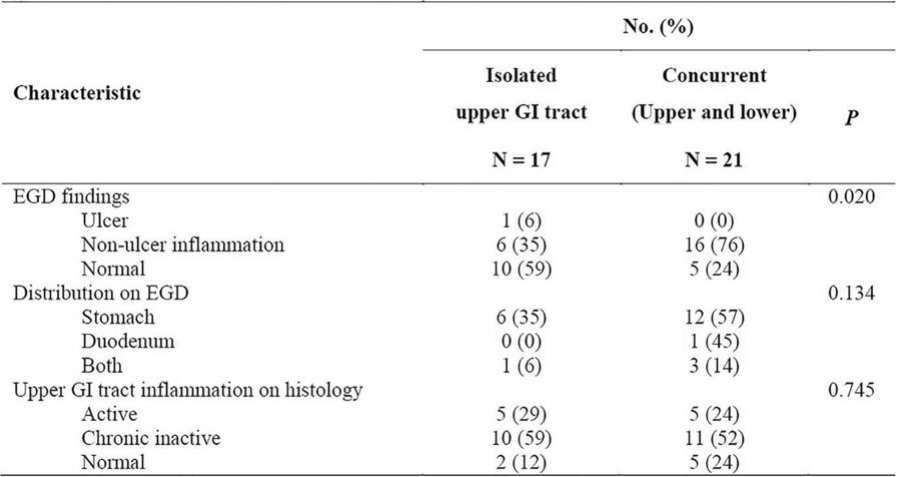
Endoscopic characteristics of concurrent and issolated upper involvement

#### P536 The fate of immune–mediated diarrhea after the resumption of immune checkpoint inhibitor treatment

##### Hamzah Abu-Sbeih, MD, Faisal S. Ali, Jianjun Gao, MD, PhD, Yinghong Wang, MD, PhD

###### MD Anderson Cancer Center, Houston, TX, USA

####### **Correspondence:** Yinghong Wang (ywang59@mdanderson.org)

**Background**

Immune–mediated diarrhea (IMD) is a leading cause for immune checkpoint inhibitor (ICPI) treatment discontinuation. Nonetheless, despite the occurrence of IMD initially, the remarkable efficacy of ICPIs encourages oncologists to resume ICPI treatment for cancer progression or as a maintenance. There is a paucity of evidence about the recurrence rate of IMD after ICPI resumption.[1] Hence, we assessed the risk and risk factors of IMD recurrence after ICPI resumption.

**Methods**

This is a cohort study of patients who had developed IMD and then resumed the same or different ICPI agent after improvement of IMD between 1/2010 and 4/2018. IMD was graded using CTCAE v4.03. A univariate followed by a multivariate logistic regression analyses were performed to assess the association of clinical covariates and IMD recurrence.

**Results**

Out of the 4864 patients who received ICPI treatment, 437 (8.9%) developed any grade IMD (Figure 1-2). Among them, 116 resumed ICPI treatment and were included in our analyses; 21 restarted anti-cytotoxic T-lymphocytes associated protein-4 (CTLA-4) and 95 anti-programmed death-1/ligand-1 (PD-1/L1). The median age was 60 years (Table 1). ICPI treatment discontinuation was due to IMD in 76 patients (66%). Seventy-nine patients (68%) required immunosuppressive therapy for the first event of IMD. The median duration from the last ICPI dose to the restart of ICPI treatment was 65 days (SD, 194). Overall, 37 (32%) patients experienced a recurrence of IMD (CTLA-4, 48%; PD-1/L1, 28%). Twenty-seven patients (73%) required immunosuppression for the recurrent IMD (Table 2); 15 of them discontinued ICPI treatment. The median duration from ICPI re-initiation to IMD recurrence was 63 days (range, 1–397). Severe IMD requiring immunosuppression initially was associated with higher grades (P<0.001) and more frequent immunosuppression requirement (P<0.001; Table 3) for the recurrent IMD. On multivariate logistic regression, patients who received anti-CTLA-4 based therapy initially had lower risk of IMD recurrence (odds ratio [OR], 0.20, 95% CI, 0.08-0.51; P=0.001; Table 4-5). The requirement for immunosuppression for IMD initially (OR, 3.04; 95% CI, 1.12-8.29; P=0.030) and the resumption of anti-CTLA-4 agents (OR, 3.89; 95% CI, 1.22-12.40; P=0.022) were associated with increased risk of IMD recurrence.

**Conclusions**

The resumption of anti-PD-1/L1 therapy has a lower IMD recurrence rate compared to anti-CTLA-4. Hence, ICPI therapy, especially anti-PD1-PD-L1, may be resumed in order to maximize the clinical benefit for patients who have limited alternative treatment options. Severe IMD requiring immunosuppression initially was a risk factor for the recurrence of severe IMD after ICPI resumption.

**References**

1. Pollack, MH, et al., Safety of resuming anti-PD-1 in patients with immune-related adverse events (irAEs) during combined anti-CTLA-4 and anti-PD1 in metastatic melanoma. Ann Oncol, 2018. 29(1): 250-255.

**Ethics Approval**

This retrospective, single-center study was approved by the Institutional Review Board at The University of Texas MD Anderson Cancer Center (IRB No. PA18-0472).

**Consent**

This study was granted waiver for consent.

**Table 1 (abstract P536).**
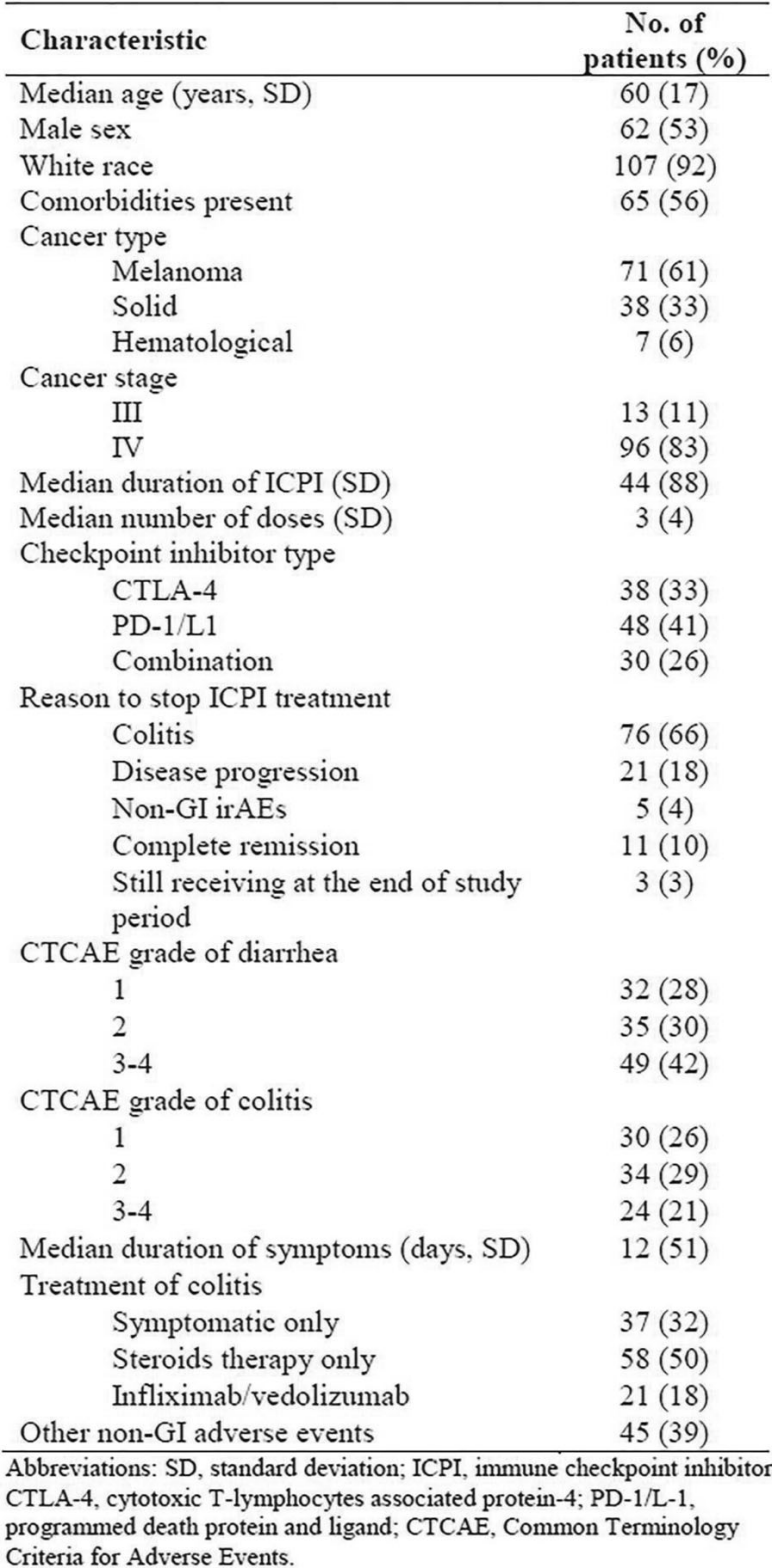
General characteristics of the initial colitis event

**Table 2 (abstract P536).**
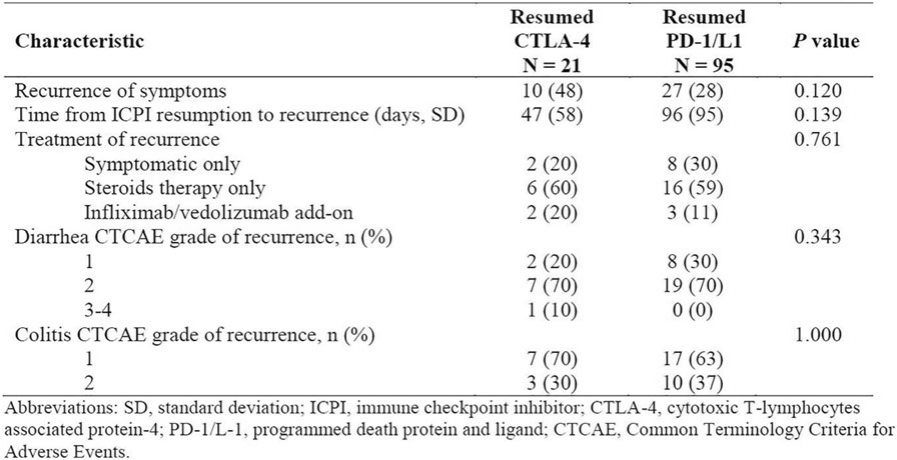
Characteristics of the recurrent immune-mediated diarrhea based on the ICPI therapy resumed

**Table 3 (abstract P536).**
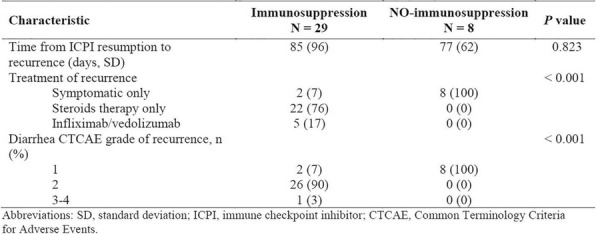
Characteristics of the recurrent immune-mediated diarrhea for patients who needed immunosuppression for the initial immune-mediated diarrhea

**Table 4 (abstract P536).**
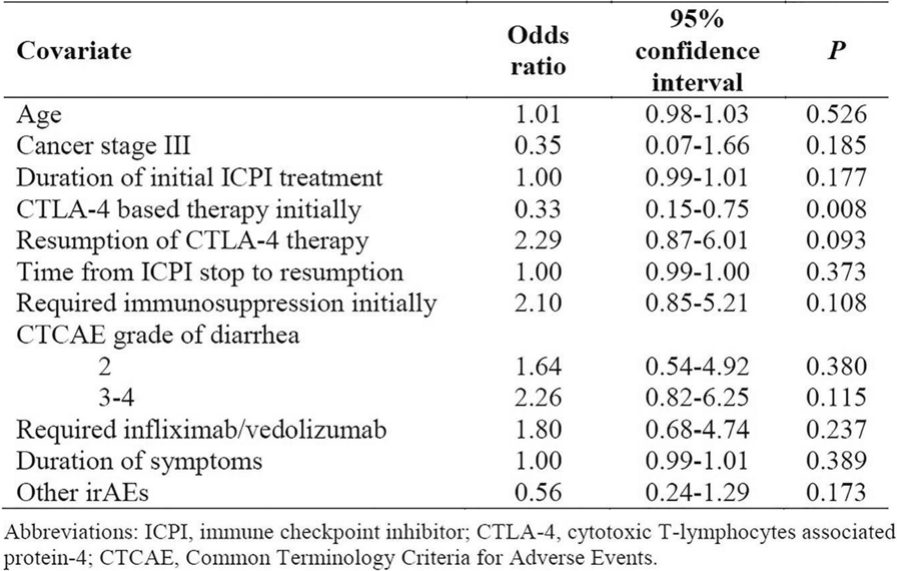
Univariate logistic regression analysis of immune-mediated diarrhea recurrence

**Table 5 (abstract P536).**
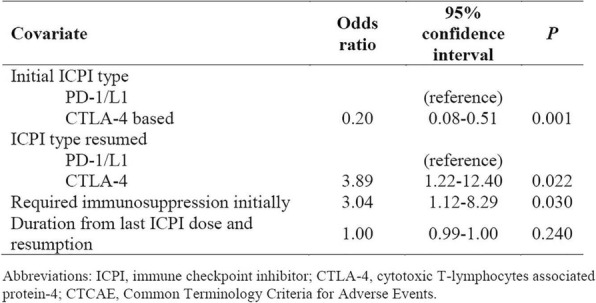
Multivariate logistic regression analysis of immune-mediated diarrhea recurrence

**Fig. 1 (abstract P536).**
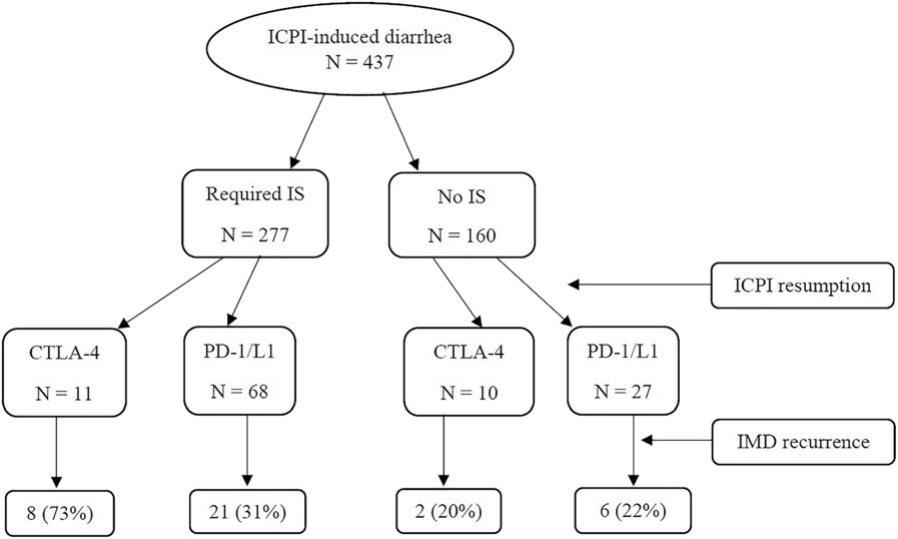
The recurrence rate of immune-mediated diarrhea (IMD) after ICPI resumption according to the immunosuppression (IS) requirement for the initial immune-mediated diarrhea

**Fig. 2 (abstract P536).**
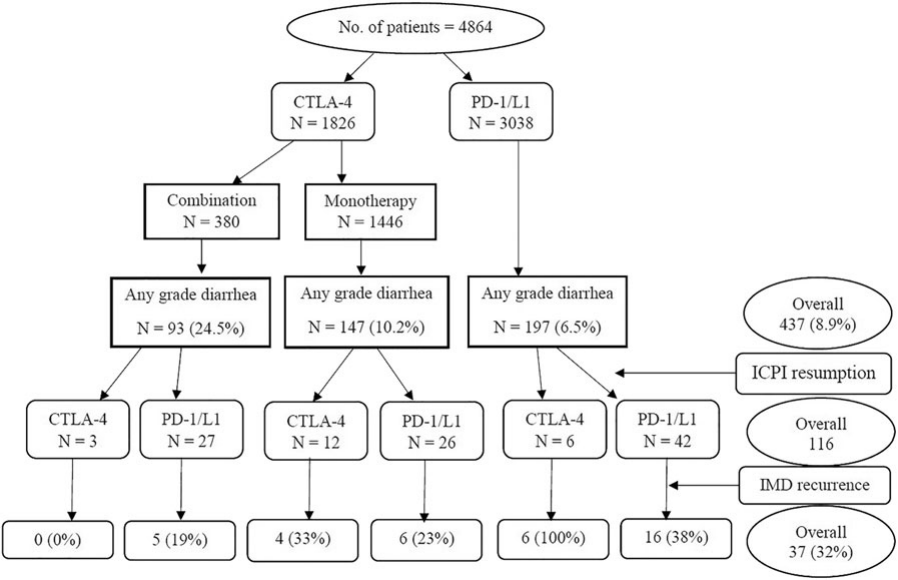
The reccurence immune-mediated diarrhea after ICPI resumption by the type of ICPI

#### P537 Immune checkpoint inhibitor–induced colitis as a predictor of survival in metastatic melanoma

##### Hamzah Abu-Sbeih, MD, Faisal S. Ali, Wei Qiao, PhD, Yang Lu, MD, Sapna Patel, MD, Adi Diab, MD, Yinghong Wang, MD, PhD

###### MD Anderson Cancer Center, Houston, TX, USA

####### **Correspondence:** Yinghong Wang (ywang59@mdanderson.org)

**Background**

Gastrointestinal (GI) immune related adverse events (irAEs) commonly limit immune checkpoint inhibitors’ (ICPIs) treatment, which is very effective for metastatic melanoma. The impact of GI-irAEs and their immunosuppressive therapy on patients’ survival is not well studied. We aimed to assess the impact of GI-irAEs on overall survival (OS) and progression free survival (PFS) of patients with metastatic melanoma.

**Methods**

This is a retrospective study of patients with metastatic melanoma who received ICPI treatment and developed GI-irAEs from 1/2010 through 4/2018 with a mean follow-up duration of 1.7 years. A number of randomized patients who did not have GI-irAEs were included in our analysis. ICPI treatment response on CT and/or FDG PET/CT images was evaluated based on combined immune-modified Response Evaluation Criteria in Solid Tumors (RECIST) and immune-related RECIST 1.1. OS and PFS were defined as the time from ICPI initiation until death or last follow-up and until progression, death, or last staging, respectively. OS was redefined as the time from diarrhea onset to study the effect of immunosuppressive therapy. Kaplan-Meier curves were used to estimate unadjusted OS and PFS time distributions (Figure1-2). The Cox proportional hazards model was used to evaluate survival predictors. GI- and non–GI-irAE were included in the Cox model as time-dependent variables.

**Results**

A total of 243 patients were included in our analyses, majority were white (93%), males (64%) with a mean age of 58 years (Table 1). In our cohort, 173 patients (71%) had GI-irAEs; 124 (72%) received immunosuppression (Table 2). In multivariate Cox regression, ECOG 2-3 (HR 4.36, 95%CI 2.38-7.99; P<0.01), LDH ≥618 IU/L (HR 2.85, 95%CI 1.79-4.49; P<0.01), stage M1c (HR 4.66, 95%CI 1.69-12.78; P<0.01) were associated with worse OS rates (Table3). In contrast, longer duration of ICPI treatment (HR 0.86, 95%CI 0.81-0.92; P<0.01) and any grade GI- irAEs (HR 0.51, 95%CI 0.31-0.83; P<0.01) were associated with improved OS rates. Immunosuppressive treatment did not affect OS (HR 1.5, 95%CI 0.82-2.74; P=0.19). High-grade diarrhea was associated with improved OS (P=0.0492; Figure 3). Additionally, patients who developed GI-irAEs had longer PFS durations on multivariate Cox model (HR 0.44, 95%CI 0.29-0.64; P<0.01; Table 4).

**Conclusions**

GI-irAEs are associated with improved survival rates in patients with metastatic melanoma. Furthermore, higher grades of diarrhea are associated with improved patients’ OS, which could explain the finding that immunosuppressive therapy did not adversely affect OS. Therefore, the onset of GI-irAEs should be conveyed to patients as a favorable sign rather than an alarming one.

**Ethics Approval**

This retrospective, single-center study was approved by the Institutional Review Board at The University of Texas MD Anderson Cancer Center (IRB No. PA18-0472).

**Consent**

This study was granted waiver for consent.

**Table 1 (abstract P537).**
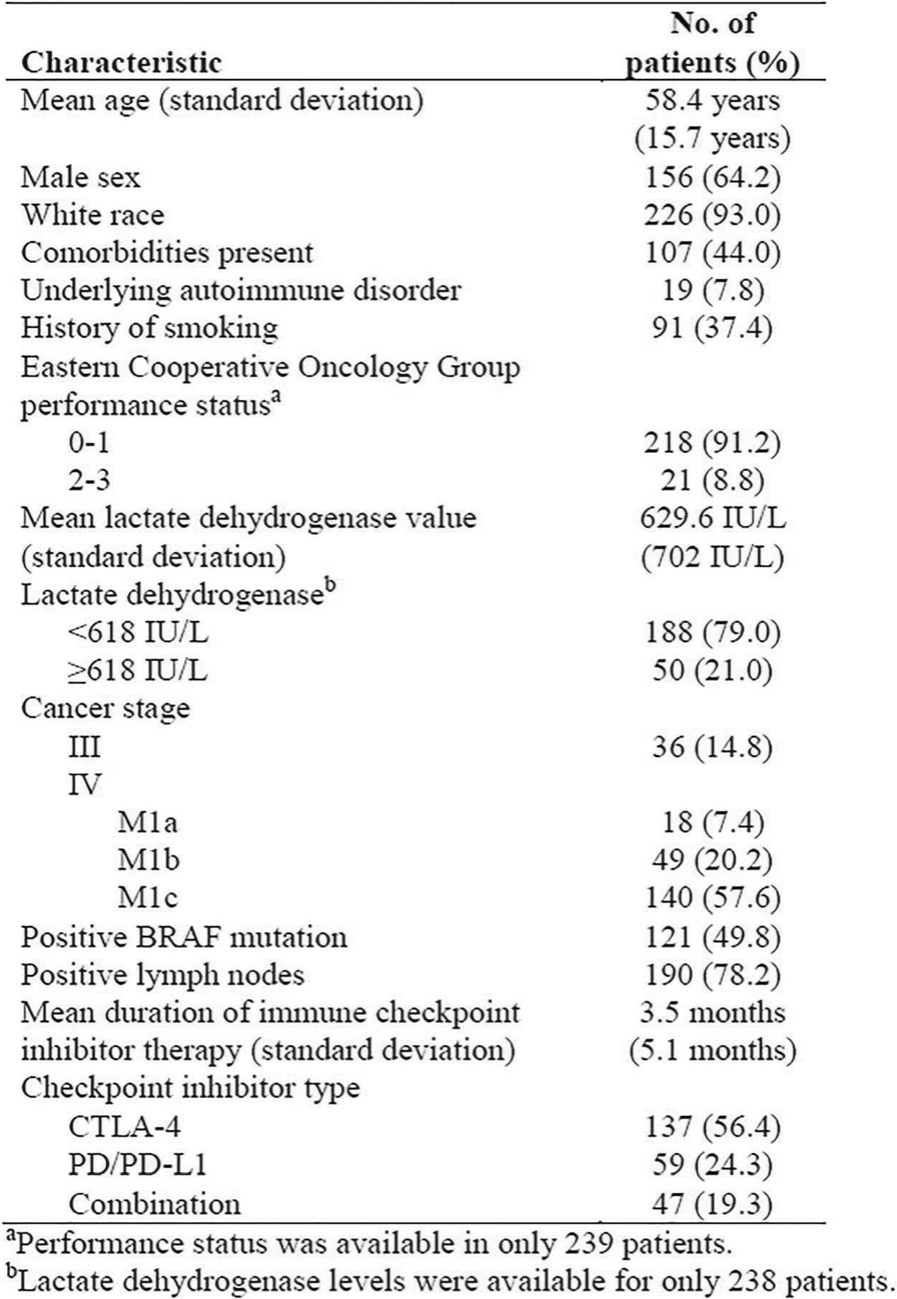
Patient characteristics (n = 243)

**Table 2 (abstract P537).**
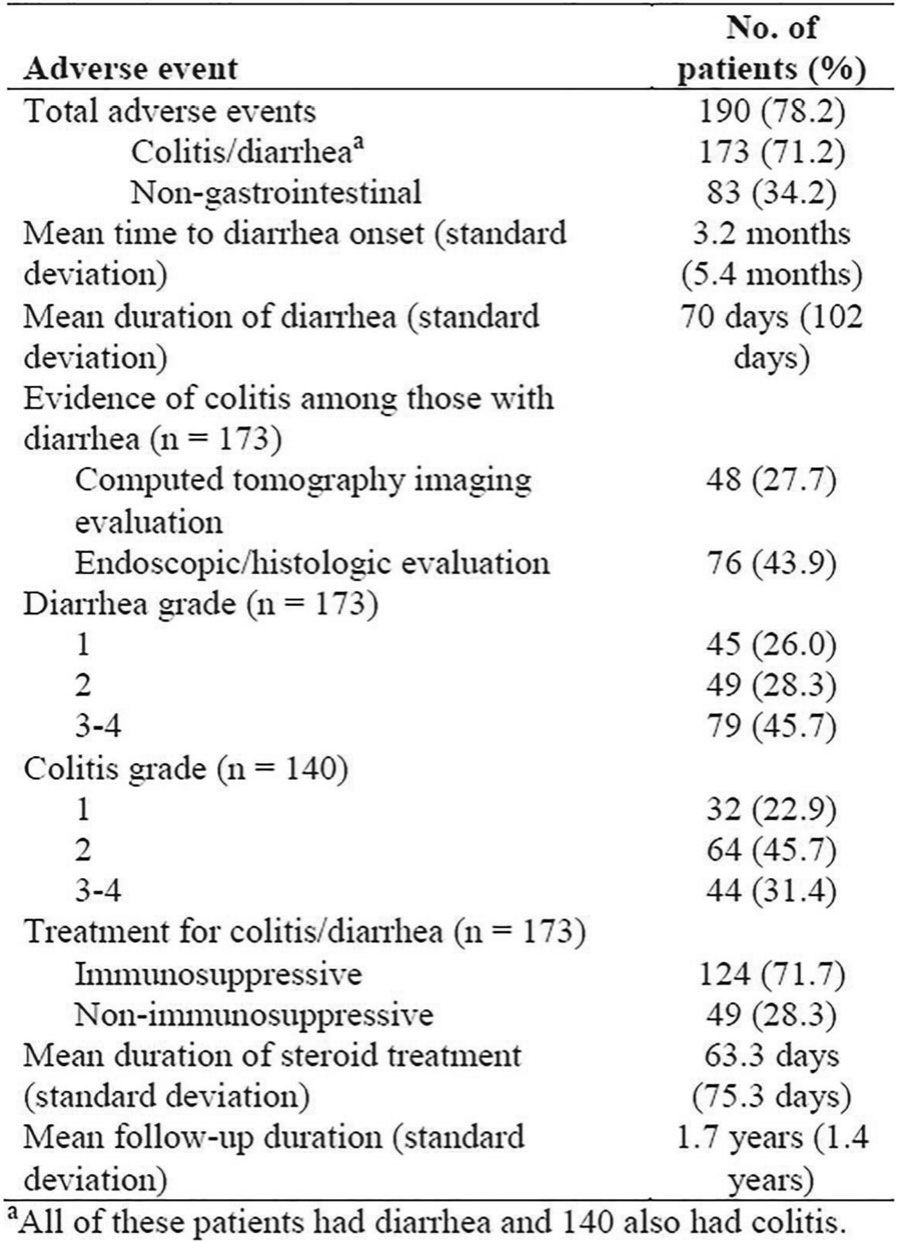
Adverse events observed in our cohort

**Table 3 (abstract P537).**
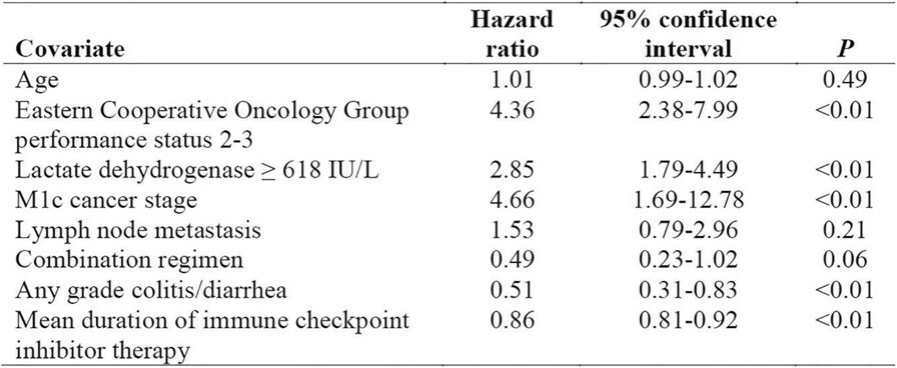
Multivariable Cox regression analysis for overall survival

**Table 4 (abstract P537).**
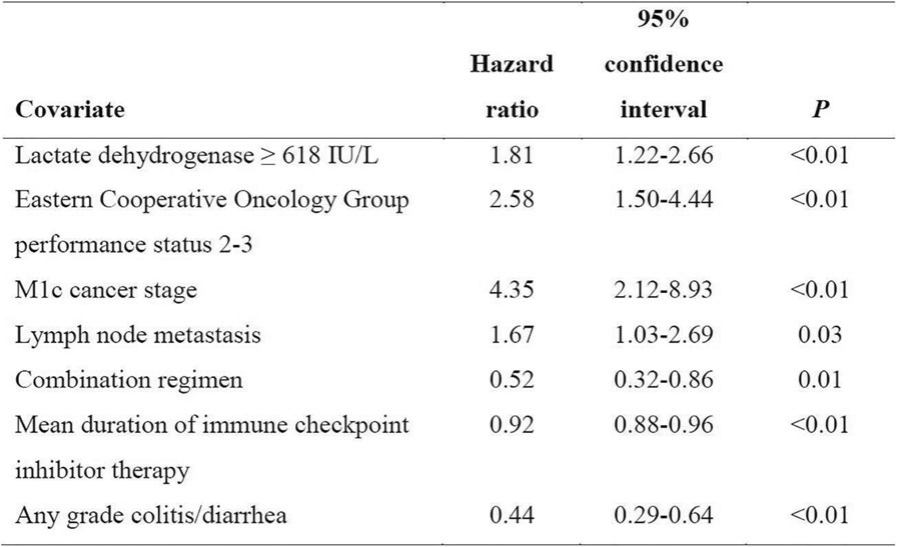
Multivariable Cox regression analysis for progression free survival

**Fig. 1 (abstract P537).**
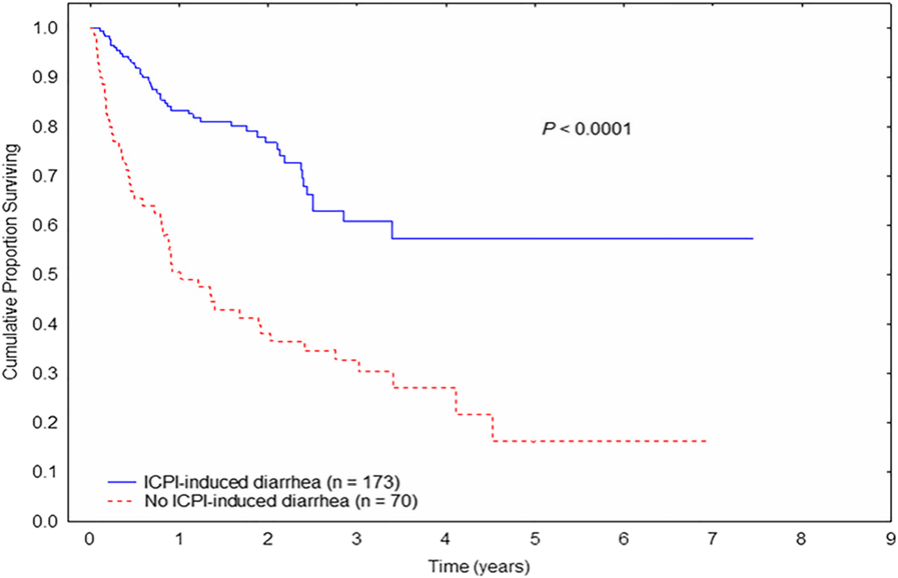
Kaplan-Meier overall survival curve stratified by immune checkpoint inhibitor (ICPI)-induced diarrhea/colitis status

**Fig. 2 (abstract P537).**
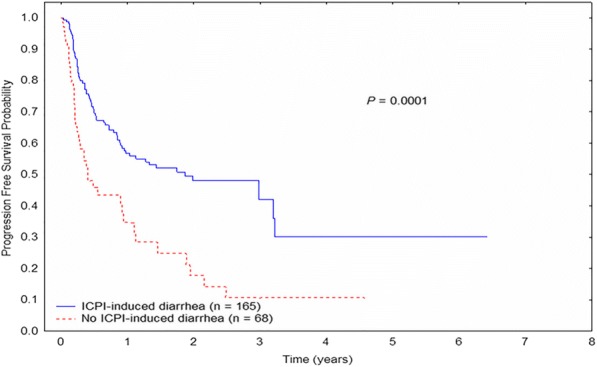
Kaplan-Meier progression-free survival curve stratified by immune checkpoint inhibitor (ICPI)-induced diarrhea/colitis status

**Fig. 3 (abstract P537).**
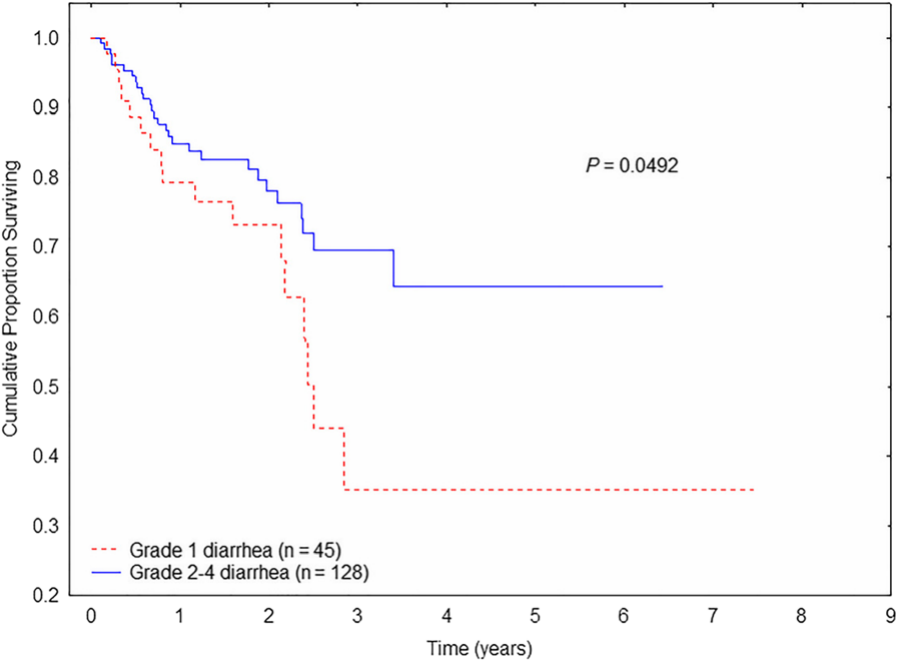
Kaplan-Meier overall survival curve stratified by diarrhea grade

#### P538 Precision medicine in immune checkpoint inhibitor–induced diarrhea and colitis treatment: the advent of organ targeted vedolizumab therapy

##### Hamzah Abu-Sbeih, MD^1^, Faisal S. Ali^1^, Dana Alsaadi^2^, Joseph Jennings, MD^2^, Wenyi Luo, MD^1^, Zimu Gong, MD^1^, David Richards, MD^1^, Aline Charabaty, MD^2^, Yinghong Wang, MD, PhD^1^

###### ^1^MD Anderson Cancer Center, Houston, TX, USA; ^2^Georgetown University, Washington, DC, USA

####### **Correspondence:** Yinghong Wang (ywang59@mdanderson.org)

**Background**

Immune checkpoint inhibitor (ICPI), which is an efficacious treatment for advanced malignancies, is commonly limited by immune mediated diarrhea and colitis (IMDC). Steroids and infliximab are often used to treat severe IMDC given its immune mediated mechanism. These agents induce systemic immunosuppression with its associated morbidity. Additionally, systemic immunosuppression might hamper the effect of ICPI. Hence, we aimed to assess clinical outcomes of vedolizumab (a gut-targeted anti-integrin agent) as an alternative treatment for IMDC.

**Methods**

This is a retrospective multicenter case series of adult patients who had IMDC and received vedolizumab from 12/2016 through 4/2018 from MD Anderson Cancer Center and Medstar-Georgetown University. All patients had IMDC that is refractory to steroids and/or infliximab.

**Results**

Twenty-eight patients were included; 20 males (71%), 25 Caucasians (89%) with a mean age of 63 years (Table 1). The most common malignancy was melanoma in 7 patients (25%). Eight patients (29%) received anti-cytotoxic T- lymphocyte associated antigen-4 (CTLA-4), 12 (43%) programmed death protein-1 or its ligand (PD-1/L-1) and 8 (29%) combination therapy. Median time from ICPI to IMDC onset was 10 weeks (IQR 1-70). Fifteen patients (54%) had grade 2 and 13 (46%) had grade 3 or 4 IMDC. Diagnostic evaluations for IMDC are shown in (Table 2). The median reduction in fecal calprotectin values was 347 for vedolizumab initiation <14 days of IMDC onset and 197 for >14 days (Figure 1). Mucosal ulceration was present in 8 patients (29%), whereas non-ulcerative inflammation was present 13 (46%). All of our patients had features of active histological inflammation; 14 (50%) had concurrent features of chronicity, and 10 (36%) had features of microscopic colitis. The treatment and outcomes of IMDC are shown in (Table 3). Mean duration of steroid treatment was 96 days (SD 74). Seven patients received infliximab in addition to steroids and were refractory to it. Median number of vedolizumab infusions was 3 (IQR 1- 4). Mean duration of follow-up was 15 months. Twenty four patients (86%) achieved and sustained clinical remission. Repeat endoscopic evaluation was performed in 17 patients. Endoscopic remission was attained in 7 (54%) of the 13 patients who had abnormal endoscopic findings initially with 5/17 (29%) patients reaching histological remission as well. (Table 4) lists the characteristics of patients who had clinical remission. In our cohort, 1 patient developed skin rash and 1 had joint pain.

**Conclusions**

Vedolizumab could be an appropriate treatment for steroid refractory IMDC, with favorable outcomes and good safety profile.

**Ethics Approval**

This retrospective, single-center study was approved by the Institutional Review Board at The University of Texas MD Anderson Cancer Center (IRB No. PA18-0472).

**Consent**

This study was granted waiver for consent.

**Table 1 (abstract P538).**
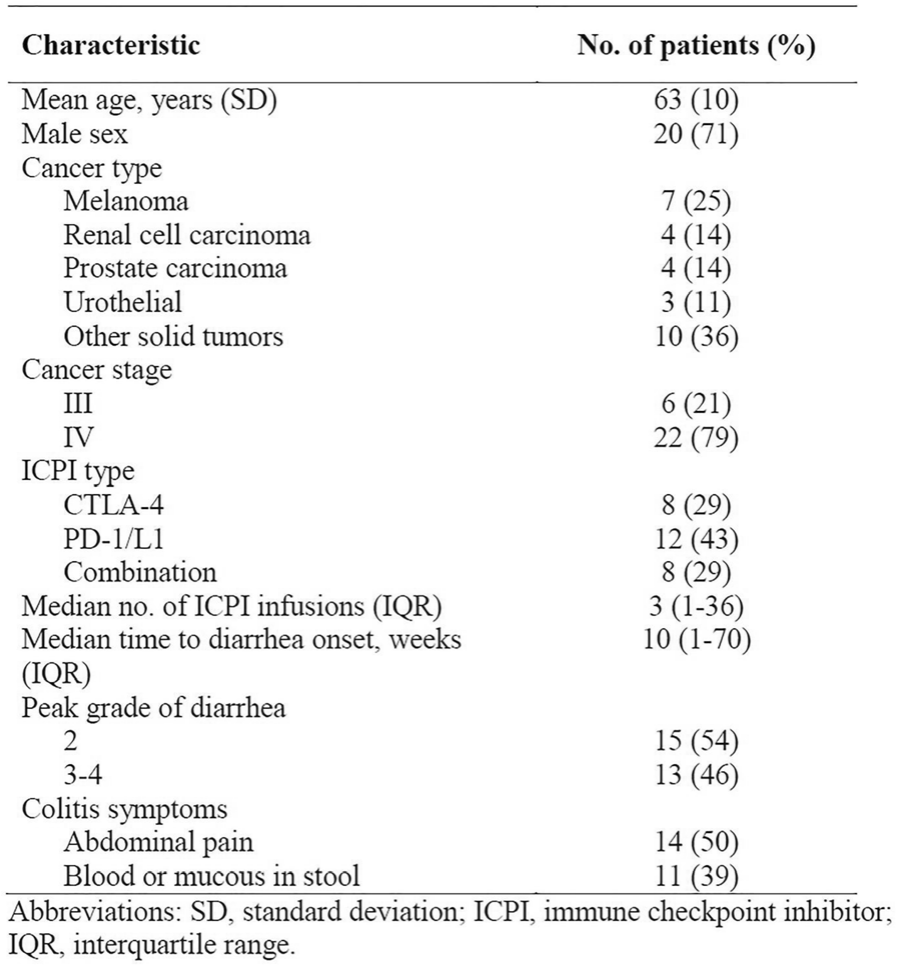
Patient clinical characteristics (*n* = 28)

**Table 2 (abstract P538).**
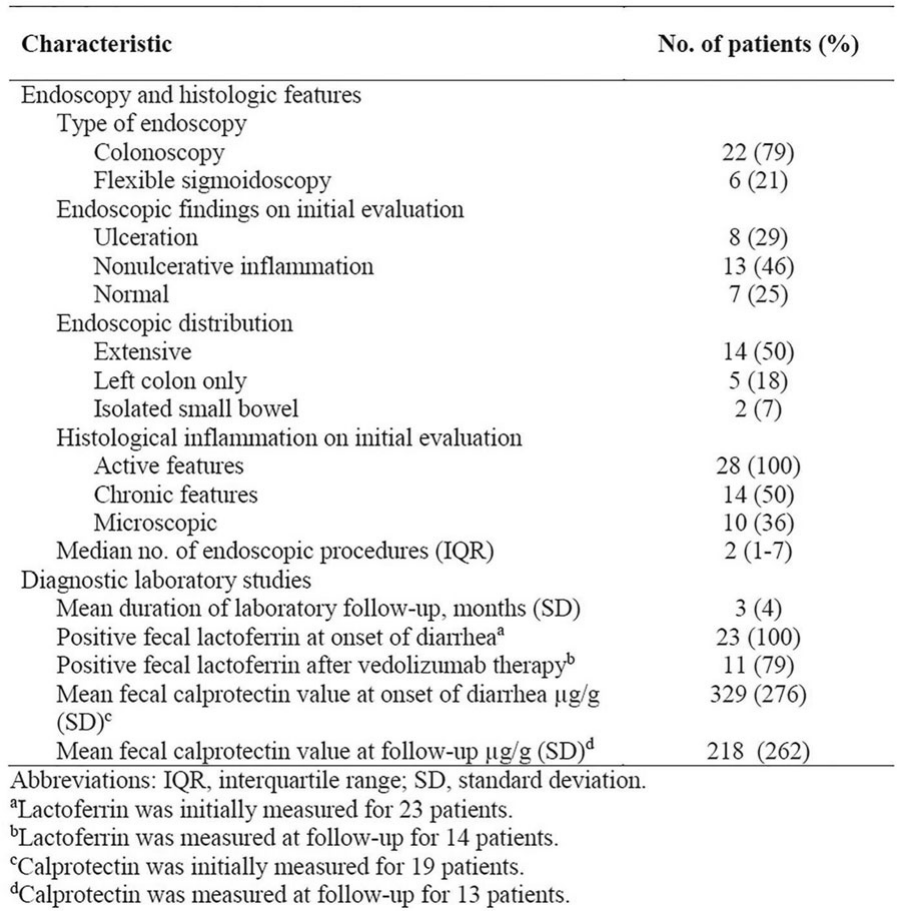
Patient diagnostic evaluation data (*n* = 28)

**Table 3 (abstract P538).**
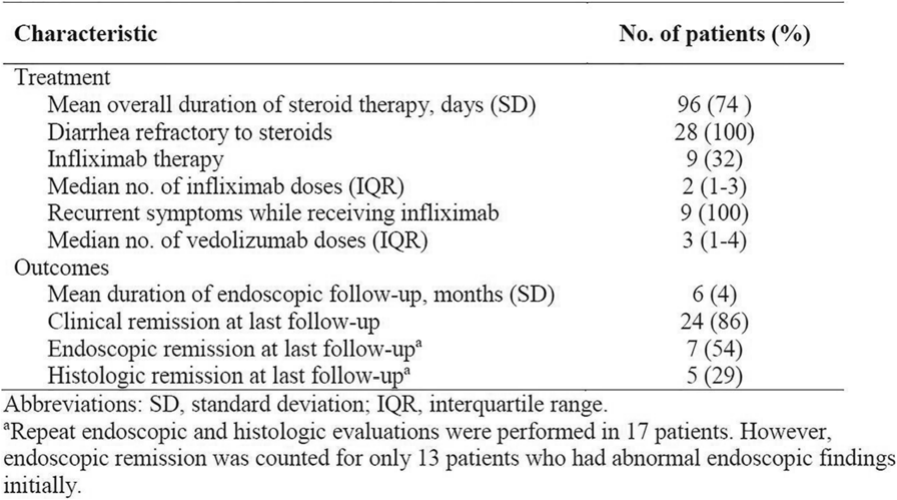
Treatment of colitis and outcomes (*n* = 28)

**Table 4 (abstract P538).**
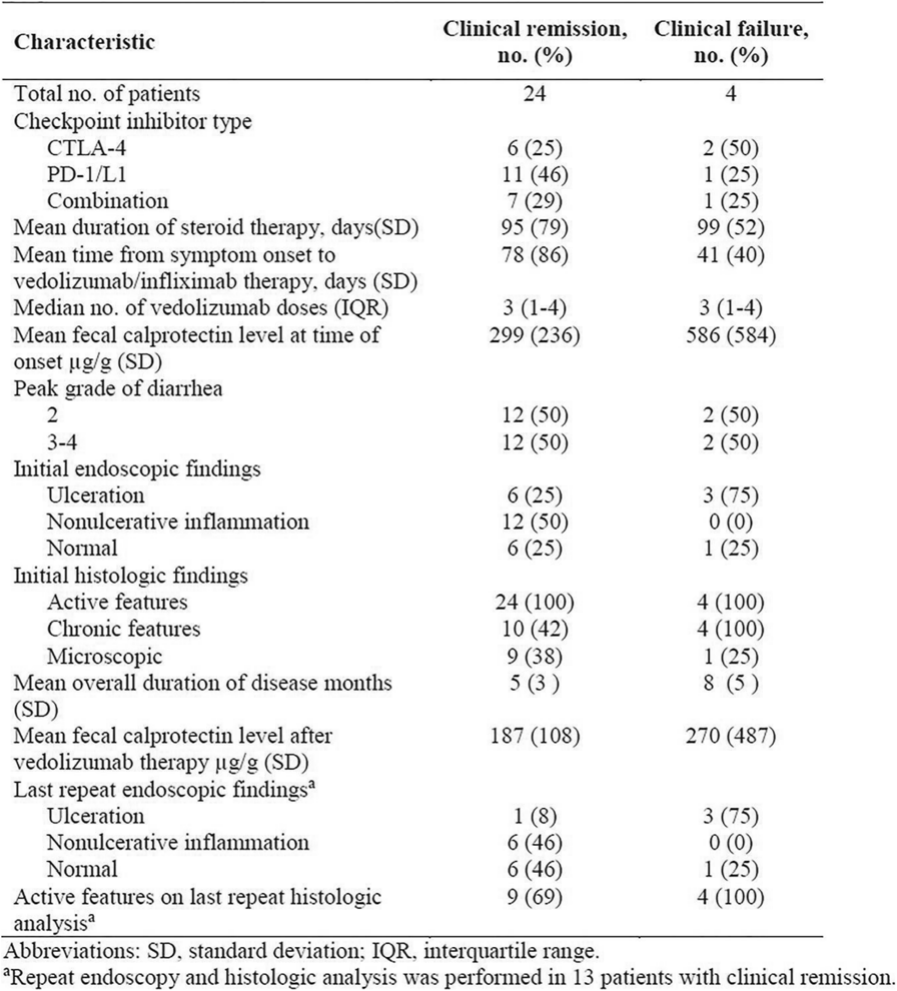
Vedolizumab therapy outcomes and clinical characteristics (*n* = 28)

**Fig. 1 (abstract P538).**
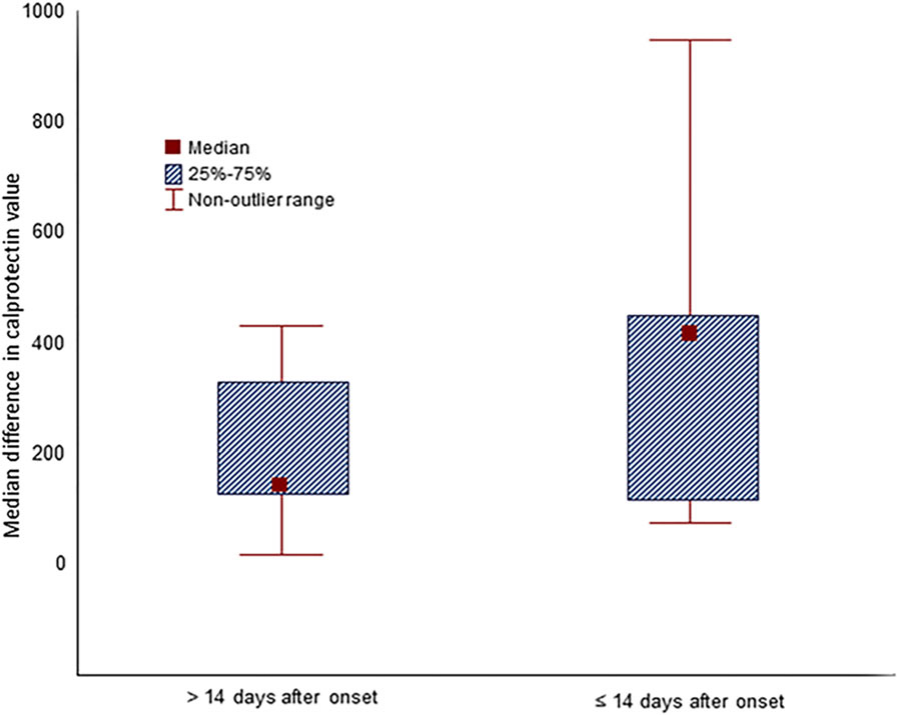
Decrease in calprotectin values after vedolizumab/infliximab therapy according to time from onset to treatment initiation

#### P539 Development of cutaneous squamous cell carcinoma in patients receiving anti PD-1/PD-L1 therapy

##### Amanda Herrmann, MD, PhD, Priyadharsini Nagarajan, MD, PhD, Vivek Subbiah, MD, Kelly Nelson, MD, Alexander J. Lazar, MD, PhD, Courteny Hudgens, MS, Khalida Wani, PhD, Michael T. Tetzlaff, MD, PhD, Jennifer A. Wargo, MD, MMSc, Anisha B. Patel, MD

###### UT MD Anderson Cancer Center, Houston, TX, USA

####### **Correspondence:** Anisha B. Patel (apatel11@mdanderson.org)

**Background**

The utilization of immune checkpoint inhibitors (ICPI) has advanced in recent years. Antibodies targeting programmed cell death-1 (PD-1) and its ligand (PD-L1) are successfully used for treatment in a variety of cancers, including metastatic and inoperable cutaneous squamous cell carcinoma (cSCC). [1] Cutaneous adverse events are seen in approximately 40% of anti PD-1 treated melanoma patients [2], but few have described the development of new cSCC in the setting of anti PD-1 therapy [3].

**Methods**

We present a summary of 10 patients treated at MDACC with anti-PD-1/PD-L1 therapy for non-squamous cell cancers who developed cSCC. Seven patients presented to the dermatology clinic following development of skin lesions during the course of their anti-PD-1/PD-L1 therapy; three more were discovered with a limited retrospective chart review. Skin biopsies were interpreted from H&E stain as well as immunohistochemical antibody analysis for CD3, CD8, PD-1, and PD-L1.

**Results**

Two female and 8 male patients aged 51- 81 years, and developed biopsy-proven well-differentiated cSCC after a median of 4 months (range 3-16 months) following therapy initiation. Four patients developed multiple lesions over the course of several months, and in 70% of cases lesions developed during anti-PD-1/PD-L1 therapy. These lesions were treated conservatively with no complications or recurrence after 12 months follow up. Skin biopsies revealed well-differentiated cSCC, with full thickness keratinocyte atypia and at least superficial dermal invasion. Immunohistochemical analysis was performed using 8 biopsies from 4 patients. PD-L1 expression was present in 63% of samples [4]. The infiltrate was predominant and non-brisk at the periphery of the tumor with normal proportions of CD3:CD8 T lymphocytes. A median of 5% (range 1-13%) of the peripheral infiltrate had PD-1 expression.

**Conclusions**

There is a potential link between the development of cSCC lesions and anti-PD-1/PD-L1 therapy. While this link has yet to be solidified, we are actively investigating factors that may contribute to the development of these lesions in the setting of ICPI therapy, including whole exome sequencing, RNAseq, and TCR sequencing. We plan to compare cSCC samples resulting from various etiologies, including BRAF inhibitor- induced cSCC and ultraviolet light-induced cSCC, to determine if there are unique characteristics in the cSCC from our ICPI- associated tumor cohort of patients. Preliminary data shows increased PD-L1 expression in the drug-induced tumors. [4] These results will be important to clinicians moving forward, as the use of this class of therapeutics rapidly increases, and may soon include the treatment of advanced cSCC.

**References**

1. Migden MR, Rischin D, Schmults CD, et al. PD-1 Blockade with Cemiplimab in advanced cutaneous squamous-cell carcinoma. N Engl J Med. 2018 Jul 26; 379(4):341-351.
2. Villadolid J, Amin A. Immune checkpoint inhibitors in clinical practice: update on management of immune- related toxicities. Transl Lung Cancer Res. 2015 Oct; 4(5):560-75.
3. Freites-martinez A, Kwong BY, Rieger KE, Coit DG, Colevas AD, Lacouture ME. Eruptive keratoacanthomas associated with pembrolizumab therapy. JAMA Dermatol. 2017; 153(7):694-697.
4. Gambichler T, Gnielka M, Rüddel I, Stockfleth E, Stücker M, Schmitz L. Expression of PD-L1 in keratoacanthoma and different stages of progression in cutaneous squamous cell carcinoma. Cancer Immunol Immunother. 2017; 66(9):1199-1204.

**Ethics Approval**

The study was approved by UT MD Anderson Cancer Center's Institutional Review Board, protocol number PA17-1060.

**Consent**

Not applicable. Waiver of consent obtained through the IRB for PA17-1060.

#### P540 Safety of cancer patients with preexisting autoimmune diseases presenting in the emergency center following immune checkpoint blockade

##### Mohsin Shah, MD, Mazen N. Jizzini, MD, Imad El. Majzoub, MD, Aiham Qdaisat, MD, Cielito C. Reyes, DrPH, Sai-Ching J. Yeung, MD, PhD

###### The Univeristy of Texas MD Anderson Cancer Center, Houston, TX, USA

####### **Correspondence:** Sai-Ching J. Yeung (syeung@mdanderson.org)

**Background**

Immune checkpoint inhibitors (ICI) have dramatically increased the survival of cancer patients, but their use can be limited by the occurrence of immune related adverse events (irAE) that may be serious and infrequently life threatening. Cancer patients with preexisting autoimmune diseases (PAD) were excluded from clinical trials. The safety of ICI in these patients is currently unknown. [1] The aim of this study was to evaluate the safety of ICI in patients with PAD that presented to the Emergency Center (EC) at a comprehensive cancer center.

**Methods**

Cancer patients with PAD who received ICI and presented to the EC between March 1, 2011 and February 29, 2016 at MD Anderson Cancer Center. We extracted data on patient’s characteristics (age, gender, preexisting autoimmune disease, treatment given for the underlying disease), type of checkpoint inhibitors used, reported irAE, how they were managed, whether immunotherapy was withheld or discontinued and their clinical outcome.

**Results**

Twenty two cancer patients with PAD were identified through institutional databases. Of these, 15 were males (68%). Mean age was 59.5 year (SD 11.6). Median Charlson Comorbidity Index was 9.5 (range 5-13). Most patients received anti PD-1 drugs (64%). Melanoma was the most common cancer (45%). Most frequent PAD were autoimmune thyroiditis and eczema. Eleven patients were actively receiving treatment for PAD at initiation of ICI therapy, of which 3 received immunosuppressive therapy (steroids in 2, disease-modifying antirheumatic drug plus rituximab in 1). Eighty six percent of cases experienced de novo irAE or PAD exacerbation. Fourteen percent were severe (Grade ≥3). Of these, 6 were managed with corticosteroids. Resolution of de novo irAE or exacerbation of PAD was achieved in 10 cases without the need to withhold or discontinue immunotherapy. Median time to last follow up or death from first infusion was 16.8 months [range 2-80]. Death was reported in 17 cases due to cancer progression.

**Conclusions**

Despite frequent de novo irAE or exacerbation of PAD, most patients with PAD who visited the ED tolerated ICI therapy well. Prospective studies are needed to establish the risk-benefit profile of ICI therapy in patients with PAD including those who did not need to visit EDs.

**References**

1. June CH, Warshauer JT, Bluestone JA. Is autoimmunity the Achilles׳ heel of cancer immunotherapy? Nat Med. 2017; 23: 540-547.

**Ethics Approval**

The study was conducted under a clinical research protocol approved by the institutional review board of The University of Texas MD Anderson Cancer Center.

### Mechanisms of Resistance to Immunotherapy

#### P541 The immunosuppressive tumor microenvironment (TME) in Epstein-Barr virus (EBV)-positive and EBV- negative gastric cancers: implications for immunotherapy

##### Sepideh Besharati, MD, Tracee McMiller, MS, Mark Yarchoan, Qingfeng Zhu, PhD, Elizabeth Engle, MSc, Janis Taube, MD, MSC, Alan Berger, Robert Anders, MD, PhD, Suzanne Topalian, MD

###### Johns Hopkins University, Baltimore, MD, USA

####### **Correspondence:** Suzanne Topalian (stopali1@jhmi.edu)

**Background**

Chemotherapy-refractory gastric carcinomas (GC) are aggressive malignancies, and only ~15% respond to drugs targeting the PD-1/PD-L1 pathway. EBV+ GCs (10% of GCs) often contain chromosomal amplifications for PD-L1 and PD-L2. They have been reported to contain robust CD8+ T cell infiltrates and an interferon-gamma (IFNg) gene signature, suggesting immune stimulation by strongly immunogenic EBV proteins. The current study aimed to characterize immune cell subsets and checkpoint expression in EBV+ GC compared to EBV(-) GC.

**Methods**

After screening >1000 cases, 25 invasive primary GC specimens AJCC stage 1A–4 (11 EBV+, 14 EBV-, confirmed with EBER ISH) were identified from treatment-naïve patients. Immunohistochemistry (IHC) was conducted for CD3, CD4, CD8, CD20, CD68, FoxP3, PD-1, PD-L1, LAG-3, GITR, IDO1, CSF1R and COX-2. Immune cell densities were quantified. RNA was isolated from macrodissected areas of dense CD3+ T cell infiltrates juxtaposed to PD-L1+ stromal cells, and gene expression profiling (GEP) was performed using multiplex qRT-PCR for a panel of 61 candidate immune-related genes.

**Results**

IHC revealed that 17/25 GCs contained PD-L1+ stromal cells (range 5-75% positive cells) with no significant difference between EBV+/- specimens; however, only 3/25 specimens contained PD-L1+ tumor cells (all EBV+). There was a higher proportion of CD8+ vs. CD4+ T cells in EBV+ tumors (p=0.051). IHC analysis of EBV+/- GCs did not show significant differences in the proportions of other immune cell subsets or expression of immune modulators. However, GEP revealed that EBV+ tumors had higher expression of IDO1 (11-fold, p=0.02). In contrast, EBV(-) tumors overexpressed CD163, CSF1R and IL10 associated with suppressive M2 macrophages (p<0.10). In addition, EBV(-) tumors overexpressed the cancer-promoting genes CXCR4 (p=0.09), IL32 (p=0.03), and IL1A (p=0.02). Notably, PTGS2 (COX-2) and IL1B, involved in prostaglandin production supporting cancer progression and metastasis, were the most highly overexpressed genes in EBV(-) tumors (270-fold, p<0.001; and 24-fold, p=0.06, respectively). IHC showed COX-2 overexpression by EBV(-) tumors (p=0.068), consistent with GEP. IHC also indicated expression of COX-2 by normal gastric epithelium.

**Conclusions**

Gastric cancers are characterized by an immunosuppressive TME regardless of EBV status, with abundant expression of PD-L1 and other immune checkpoints. GEP revealed that EBV(-) GCs, which are much more common than EBV+ GCs, overexpress molecules such as COX-2, IL-1A, IL-1B, IL-10 and CSF1R. Our findings provide novel insights into the immune microenvironment of EBV+ and EBV(-) GC, and offer potential targets to overcome resistance to anti-PD-1/PD-L1 therapies in this disease.

**Acknowledgements**

Funded by the Bristol-Myers Squibb International Immuno-Oncology Network and NCI R01 CA142779.

#### P542 Exposure to anti-PD-1 causes Functional Differences in Tumor-Infiltrating Lymphocytes in Solid Tumors

##### Caitlin Creasy, MS^1^, Cara Haymaker, PhD^2^, Marie-Andrée Forget, PhD^2^, Gopal Singh, PhD^2^, Coya Tapia, MD, PhD^2^, Chantale Bernatchez^2^, Jeane Painter, PhD^2^, Funda Meric-Bernstam, MD^2^, Caitlin Creasy, MS^1^

###### ^1^MD Anderson Cancer Center- UTHealth Graduate School of Biomedical Sciences, Houston, TX, USA; ^2^UT MD Anderson Cancer Center, Houston, TX, USA

####### **Correspondence:** Chantale Bernatchez (cbernatchez@mdanderson.org)

**Background**

The pervasive use of therapeutic antibodies targeting PD-1 puts it on target to become the standard of care for solid tumor malignancies. However, little is known as to how blockade of PD-1 may alter the function or phenotype of tumor-infiltrating lymphocytes (TIL). By investigating samples from pre-treatment and early on-treatment biopsies from patients with varying types of solid tumors treated with anti-PD1, we hope to elucidate drug-induced changes in TIL phenotype and function.

**Methods**

An ongoing Phase II clinical trial of anti-PD-1 in cohorts of patients with rare solid tumor types (NCT02721732) yielded mandatory core biopsies taken at baseline and day 15-21 after the first cycle of anti-PD-1 (Pembrolizumab, 200 mg). Upon receipt, half of the biopsy was mechanically disaggregated for TIL phenotyping, which we term “fresh” flow cytometry staining. The other half of the biopsy was used to propagate TIL ex vivo using the TIL 3.0 method, which includes IL-2, agonistic anti-4-1BB antibody (Urelumab, BMS), and anti-CD3 (clone OKT3). TIL phenotype and function were evaluated after 2 or 3 weeks of culture. Functionality was determined through sorting T cell subsets and measuring cytokine and chemokine secretion following anti-CD3 re-stimulation using MSD and Luminex platforms.

**Results**

Phenotypic analysis of the freshly stained and expanded TIL demonstrated an effector memory differentiation status before and after exposure to anti-PD-1. These TIL did not differ in their expansion of the CD4+ or CD8+ subsets. This is expected within the expanded TIL, given the predisposition to expand CD8+ TIL with the addition of anti-4- 1BB. Further, expanded TIL retained cytotoxic potential (perforin/granzyme B) after one dose of anti-PD-1. However, PD-1 expression on expanded CD8+ tended to be elevated after therapy (p=0.09). Further, TIL expanded after anti-PD-1 showed enriched CTLA-4 expression in CD4+ TIL (p=0.003). Functional analysis of 16 paired baseline and on-treatment expanded TIL show that CD4+ TIL with higher IL-4 secretion are accompanied by inhibited cellular growth (p<0.05). CD8+ TIL demonstrated an inverse relationship with growth and higher expression of PD-1, CTLA-4, and cytotoxic molecules, perforin/granzyme B (p<0.05). Further analysis in 12 of the 16 paired expanded CD8+ TIL samples for 65 soluble factors demonstrated an aberrant secretion profile post anti- PD1 treatment suggesting impaired function.

**Conclusions**

Our study assesses the ramifications of one dose of anti-PD-1 on TIL in rare solid tumors. We demonstrate that although phenotypically similar after undergoing checkpoint blockade, TIL tend to have a poorer functionality after anti-PD-1.

**Ethics Approval**

The study was approved by UT MD Anderson Cancer Center's IRB, approval number 2015-0948.

**Consent**

Written informed consent was obtained from the patient for publication of this abstract and accompanying images. A copy of the written consent is available for review by the Editor of this journal.

#### P543 Neoantigen heterogeneity as contributing factor for non-responders to neoantigen specific T-cell therapy in patients with metastatic gastrointestinal malignancies

##### Eric Groh, MD, Jared Gartner, MS, Todd Prickett, PhD, Yong Li, MS, Steven Rosenberg, MD, PhD, Paul Robbins, PhD

###### NCI, Bethesda, MD, USA

####### **Correspondence:** Paul Robbins (paul_robbins@nih.gov)

**Background**

In an initial pilot study, tumor infiltrating lymphocytes (TIL) that recognize neoepitopes were identified in 9 of 10 patients with metastatic gastrointestinal (GI) malignancies. These findings suggest that adoptive transfer of autologous neoepitope-reactive T cells may represent an attractive treatment strategy for patients with metastatic GI malignancies; however, objective response rates for this treatment strategy remain low. Factors that may influence therapies that target one or a relatively small number of mutations include intra- and inter-tumor mutational heterogeneity, which are evaluated in this study.

**Methods**

Whole exome sequencing (WES) was carried out on resected flash-frozen metastatic tumor samples and matched normal cellular DNA to identify somatically mutated gene products, and TIL cultures initiated from adjacent tumor regions. Co-culture of antigen presenting cells expressing individual tumor mutations and TIL allowed identification of neoepitope-reactive TIL which were then used for adoptive cell therapy. Additionally, FFPE tumor samples were obtained for each patient from which WES was performed to characterize tumor mutations.

**Results**

WES data was analyzed from 39 unique tumors from 12 patients with metastatic GI malignancy treated with neoepitope-reactive TIL. A mean of 3 tumors per patient were analyzed (range 2-6 tumors), and the mean number of mutations per sample was 101 (range 22-156 mutations). Inter-tumor heterogeneity was present in all 12 patients (Figure 1). The percent of mutations ubiquitously expressed in all samples from an individual patient ranged from 12.9% (Patient 4071) to 67.9% (Patient 3737). Mutation reactive TIL therapy resulted in an objective response in 2 of the 12 patients. The single neoantigen targeted by TIL administered to the 2 patients with objective responses was present in all additional FFPE samples studied. In contrast, for 7 of the 10 non-responders, targeted mutations that could not be detected 1 or more of the analyzed FFPE samples were identified (Table 1). These findings raised the concern that the lack of response seen in some patients receiving adoptive TIL transfer could be due to the absence of the targeted mutations in individual tumor lesions.

**Conclusions**

Inter-tumor heterogeneity of targeted mutations is presents in non-responders to neoepitope-reactive TIL therapy. This suggested one mechanism limiting the effectiveness of TIL therapy targeting a limited number of mutations. WES of primary and metastatic tumors from primary and metastatic tumors from individual patients may facilitate identification of ubiquitously expressed tumor mutations that, if successfully targeted by adoptively transferred TIL, may lead to improved object response rates.

**Fig. 1 (abstract P543).**
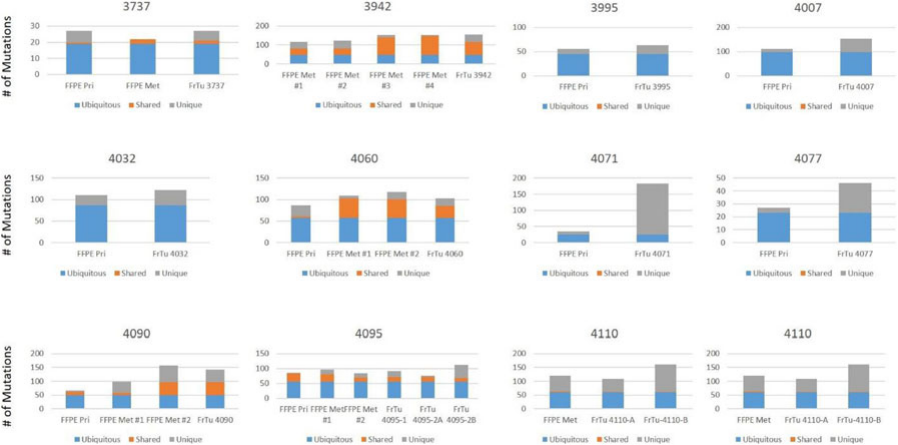
See text for description

**Table 1 (abstract P543).**
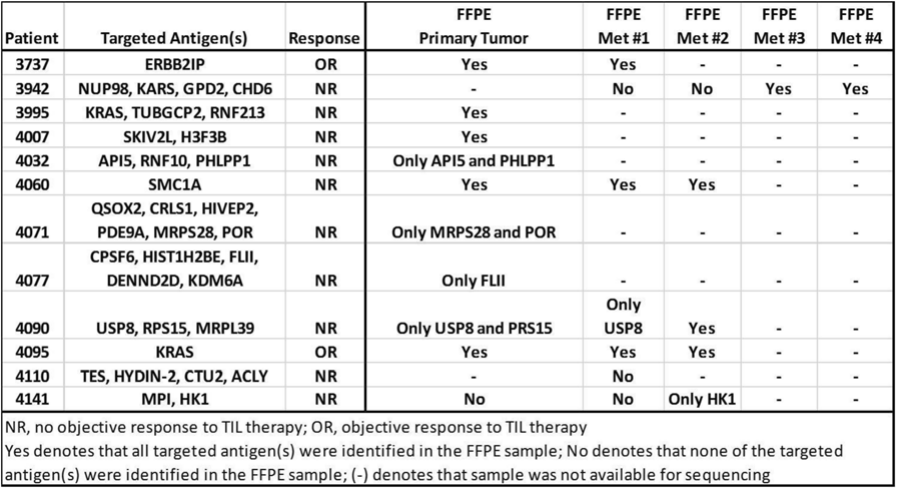
See text for description

#### P544 Interferon-γ regulates the expression of PD-L1 and NKG2D ligand families in nasopharyngeal carcinoma

##### Lingling Guo^1^, Yu Chen, MD, PhD^2^, Chuaben Chen^2^

###### ^1^Fujian medical university affiliated cancer hospital, Fuzhou, Peoples Republic of China; ^2^Fujian Province Cancer Hospital, Fuzhou, Peoples Republic of China

####### **Correspondence:** Chuaben Chen (bencc@sina.com)

**Background**

Nasopharyngeal carcinoma (NPC) is an Epstein-Barr virus-related malignancy that is characterized by a large number of infiltrating lymphocytes in the tumor microenvironment. The CD8+ T-lymphocytes can release interferon γ (IFN-γ), a cytokine proven to be a strong stimulant of programmed death-ligand 1(PD-L1). Hence the high expression of PD-L1 is another characteristic of NPC. It is reported that PD-1/PD-L1 inhibitors have good therapeutic effects on solid tumors with high PD-L1 expression and extensive CD8+ T-lymphocyte infiltration. However, current clinical trials only show a limited effect of PD-1 inhibitors on recurrent or metastatic NPC. To find the mechanism of resistance to PD-1/PD-L1 inhibitors in NPC, we studied the co-expression status of the NKG2D ligand family, a group of activating molecules expressing on the surface of activated CD8+ T-lymphocytes, when IFN-γ is up-regulating the expression of PD-L1 in NPC.

**Methods**

We downloaded the RNA-Seq data of IFN-γ, PD-L1, MICA and ULBP2-4 in head and neck squamous cancer (HNSC, except NPC) and NPC respectively from the TCGA and the GEO databases, screened for the most related molecules to IFN-γ. We also administrated exogenous IFN-γ to three types of NPC cell lines, CNE-1, CNE-2 and 5- 8F, and did qPCR, flow cytometry (FC), Western-blot (WB) and RNA-Seq after 0, 4, 12, and 24 hours, to determine the expression level of above molecules at each time point.

**Results**

In 501 HNSC cases from the TCGA database, the expression of PD-L1 is significantly related to IFN-γ (R=0.65, P<0.001) while the correlation between MICA and IFN-γ is very weak (R=0.12, P=0.004). Among the ULBPs, ULBP3 has the most significant correlation with IFN-γ (R=-0.18, P<0.001). The 113 NPC cases from the GEO database show the similar results. All the cell experiments proved the positive correlation between PD-L1 and IFN-γ and the negative correlation between MICA and IFN-γ (P<0.05). All the ULBPs had a significant negative correlation with IFN-γ in WB and FC, whereas ULBP3 showed a best correlation in RNA-Seq and qPCR(P<0.05).

**Conclusions**

We confirmed that IFN-γ can up-regulate the expression of PD-L1 and change the expression of NKG2D ligands. ULPB3 has a most significant and time-dependent negative correlation with both IFN-γ. Therefore, we hypothesize the down-regulation of ULBP3 mediated by IFN-γ may be a unique mechanism of immune escape and primary resistance in NPC patients. Further studies are needed to explore the deeper molecular mechanism.

**Fig. 1 (abstract P544).**
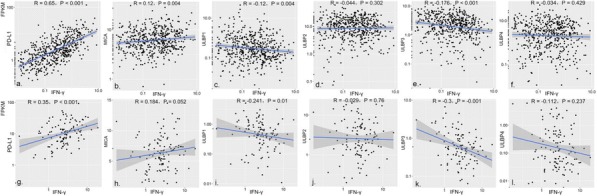
See text for description

**Fig. 2 (abstract P544).**
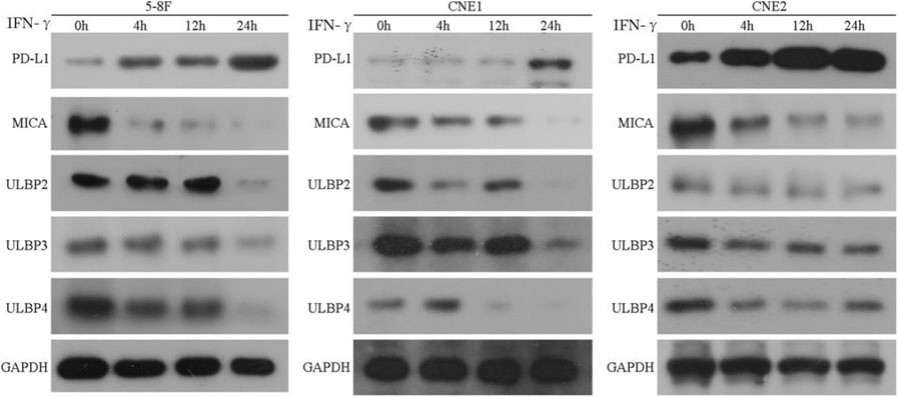
See text for description

**Fig. 3 (abstract P544).**
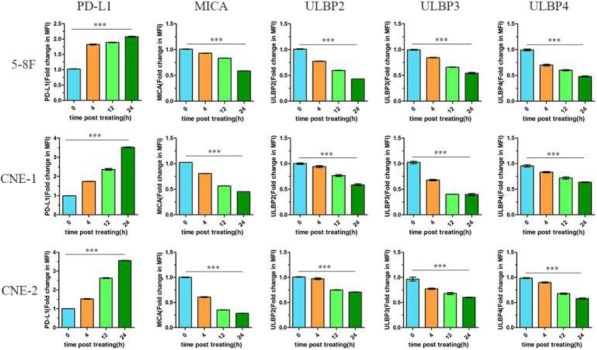
See text for description

**Fig. 4 (abstract P544).**
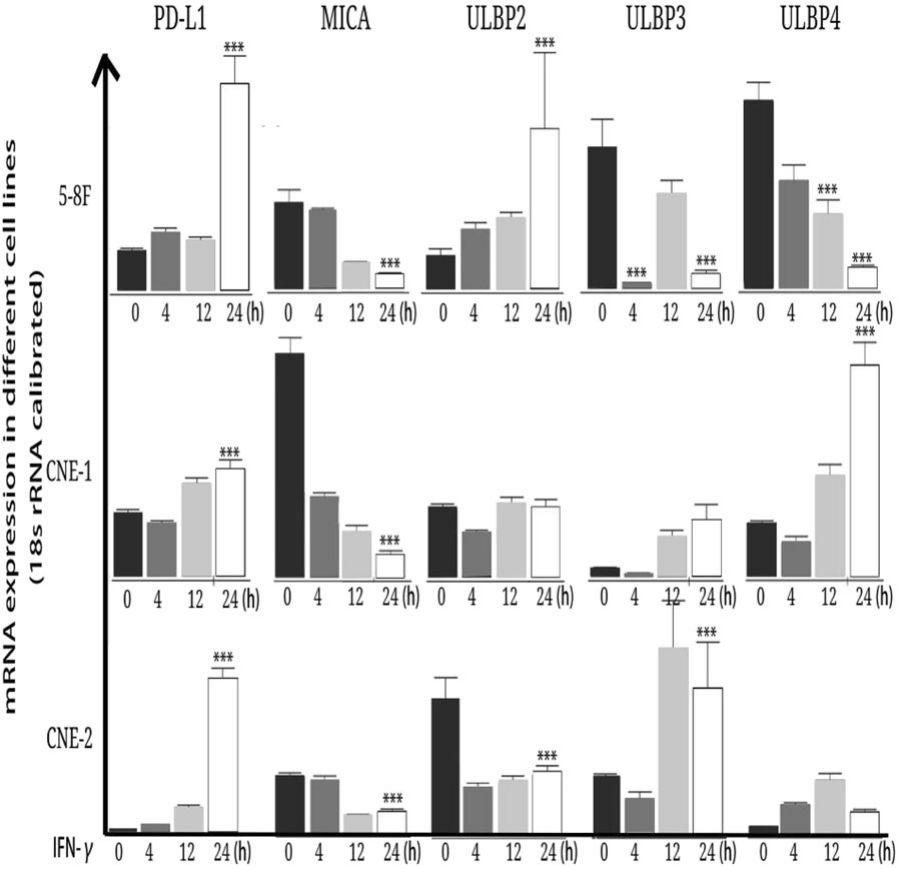
See text for description

#### P545 The tumor inflammasome functions as a rheostat for regulating the efficacy of anti-PD-1 antibody immunotherapy by driving the recruitment of myeloid-derived suppressor cells

##### Brent Hanks, MD, PhD^3^, Bala Thievanthiran, PhD^1^, Kathy Evans, BS^1^, Nicholas DeVito, MD^1^, Christine Xiao, BS^1^, Yubin Kang, MD^1^, David Hsu, MD, PhD^1^, Alisha Holtzhausen, PhD^2^

###### ^1^Duke University, Durham, NC, USA; ^2^University of North Carolina, Chapel Hill, NC; ^3^Duke University Medical Center, Durham, NC, USA

####### **Correspondence:** Brent Hanks (brent.hanks@duke.edu)

**Background**

Despite the impact of anti-PD-1 antibody immunotherapy in oncology, many cancer patients do not benefit from this treatment modality. Mechanisms of resistance to checkpoint inhibitor immunotherapy in cancer remain poorly understood.

**Methods**

Various pre-clinical syngeneic and transgenic cancer models as well as an autologous humanized mouse model were utilized to investigate the genetic and cellular alterations of tumors escaping anti-PD-1 antibody immunotherapy based on immunohistochemistry, flow cytometry, RNAseq transcriptomic sequencing, and tandem mass spectrometry.

**Results**

Our studies have demonstrated that anti-PD-1 antibody immunotherapy promotes the recruitment of granulocytic myeloid-derived suppressor cells (Gr-MDSCs) to the tumor bed in a CXCR2-dependent manner. This process involves the upregulation of the CXCR2 chemokine ligand, CXCL5, which is induced by a Wnt5a-YAP non- canonical autocrine signaling pathway that, in turn, is stimulated by a HSP70-TLR4 signaling axis. Further investigation has demonstrated that this cascade of events is initiated by CD8+ T cell-dependent stimulation of the NLRP3 inflammasome in tumor cells which drives the release of HSP70 and serves to dampen the efficacy of anti- PD-1 antibody immunotherapy. This phenomenon has been observed in pre-clinical models of non-small cell lung cancer, melanoma, and pancreatic cancer, as well as an autologous humanized mouse model of renal cell carcinoma in response to anti-PD-1 antibody therapy but not anti-CTLA-4 antibody therapy or chemotherapy. Additional studies have revealed that this pathway also drives the accumulation of Gr-MDSCs in distant tissues such as the lung in a manner that is dependent upon the presence of the primary tumor. Genetic silencing of CXCL5 and pharmacological blockade of Wnt ligand signaling suppresses Gr-MDSC recruitment in response to anti-PD-1 antibody therapy and enhances the efficacy of checkpoint inhibitor therapy. CXCR2 and CXCR2 ligand expression correlates with Wnt5a and with cytolytic T cell markers in human melanoma tissues indicating that this pathway is clinically relevant. We have determined that levels of CXCL5 and other CXCR2 ligands are increased in the plasma of transgenic tumor models escaping anti-PD-1 antibody therapy. Clinical studies are ongoing to investigate relationships between anti-PD-1 antibody response and the plasma levels of CXCR2-dependent chemokines as well as genetic inflammasome polymorphisms in advanced melanoma patients undergoing checkpoint inhibitor therapy.

**Conclusions**

By promoting the recruitment and accumulation of Gr-MDSCs, the tumor inflammasome functions as a rheostat that modulates the T cell response generated by anti-PD-1 antibody therapy. The elucidation of this pathway provides novel therapeutic opportunities to expand on the efficacy of checkpoint inhibitor immunotherapy for cancer patients.

**Ethics Approval**

This study was approved by the Institutional Animal Care and Use Committee at Duke University, registry number A249-12-09.

#### P546 Targeted hypoxia reduction restores T cell infiltration and sensitivity to immunotherapy in prostate cancer

##### Priyamvada Jayaprakash, PhD, Midan Ai, PhD, Arthur Liu, Pratha Budhani, BS, MS, Todd Bartkowiak, Jie Sheng, Casey Ager, BS, Courtney Nicholas, PhD, Ashvin Jaiswal, Yanqiu Sun, Krishna Shah, Sadhana Balasubramanyam, Nan Li, Guocan Wang, PhD, Jing Ning, Anna Zal, Tomasz Zal, PhD, Michael Curran, PhD

###### The University of Texas MD Anderson Cancer Center, Houston, TX, USA

####### **Correspondence:** Michael Curran (mcurran@mdanderson.org)

**Background**

Checkpoint blockade using anti CTLA-4 and anti PD-1 is effective in “hot” tumors like melanoma with pre-existing immune infiltrates (1); however, “cold” tumors like prostate cancer respond poorly. Within these tumors we find prevalent zones of hypoxia from which T cells are excluded. Hypoxia impedes T cell mediated antitumor immunity by creating a hostile microenvironment through acidification of the extracellular milieu, formation of abnormal vasculature lacking in adhesion receptors needed for T cell extravasation, and recruitment of immunosuppressive stromal populations such as myeloid derived suppressor cells (MDSC) and myofibroblasts (2-5) . We hypothesized that removing these hypoxic zones would restore T cell infiltration and effector function thus sensitizing prostate tumors to checkpoint blockade.

**Methods**

We utilized the hypoxia-activated prodrug, TH-302 (Evofosfamide) to ablate hypoxic zones of tumors and tested its efficacy alone and in combination with immune checkpoint blockade in reducing tumor burden and enhancing anti- tumor immunity using both transplantable and spontaneous models of prostate cancer. We investigated how this therapy impacted recruitment and function of tumor-infiltrating CD8+ T cells and immune suppressive MDSC.

**Results**

We found that TH-302 improved survival of prostate tumor bearing mice (OS 30%) and synergized with checkpoint blockade using αCTLA-4 and αPD-1 in curing 82% of mice compared to checkpoint blockade alone (OS 55%). This was concomitant with enhanced T cell and decreased MDSC infiltration into tumors. T cells infiltrating tumors treated with the combination of TH-302 and αCTLA-4/αPD-1 had higher proliferative capacity, improved effector cytokine production and greater mitochondrial biomass. In addition, MDSC from these tumors displayed reduced suppressive activity. Combination treatment caused a persistent defect in the ability of tumors to replenish their myeloid stroma. In addition, spontaneous prostate cancer in TRAMP transgenic mice, which is entirely checkpoint blockade resistant, responded dramatically to combination CTLA-4/PD-1 blockade when co-administered with the hypoxia-reducing agent TH-302.

**Conclusions**

We have shown that hypoxia reduction can sensitize otherwise resistant prostate tumors to immunotherapy. Hypoxic ablation is associated with the conversion of the immunosuppressive tumor microenvironment (TME) into an immune permissive one through improved survival, function and metabolic fitness of T cells and reduced infiltration and function of immunosuppressive MDSC. Our findings suggest that metabolic manipulation of the TME is a viable approach to enhance immunotherapy responses.

**References**

1. Curran MA, Montalvo W, Yagita H, and Allison JP. PD-1 and CTLA-4 combination blockade expands infiltrating T cells and reduces regulatory T and myeloid cells within B16 melanoma tumors. Proc Natl Acad Sci U S A. 2010;107(9):4275-80. 2. Corzo CA, Condamine T, Lu L, Cotter MJ, Youn JI, Cheng P, Cho HI, Celis E, Quiceno DG, Padhya T, et al. HIF-1alpha regulates function and differentiation of myeloid-derived suppressor cells in the tumor microenvironment. J Exp Med. 2010;207(11):2439-53. 3. Dang EV, Barbi J, Yang HY, Jinasena D, Yu H, Zheng Y, Bordman Z, Fu J, Kim Y, Yen HR, et al. Control of T(H)17/T(reg) balance by hypoxia-inducible factor 1. Cell. 2011;146(5):772-84. 4. Ben-Shoshan J, Maysel-Auslender S, Mor A, Keren G, and George J. Hypoxia controls CD4+CD25+ regulatory T-cell homeostasis via hypoxia-inducible factor-1alpha. Eur J Immunol. 2008;38(9):2412-8. 5. Becker JC, Andersen MH, Schrama D, and Thor Straten P. Immune-suppressive properties of the tumor microenvironment. Cancer immunology, immunotherapy : CII. 2013;62(7):1137-48.

#### P547 Adoptive cell therapy and intratumoral nanoplexed poly I:C as an effective immunotherapy in interferon- signaling deficient melanoma

##### Anusha Kalbasi, MD^2^, Kevin Hakimi, BS^1^, Sarah Kremer^1^, Christine Nguyen, BS^1^, Davis Torrejon, MD^1^, Giulia Parisi^1^, Pedro Lopez-Casas, PhD^3^, Marisol Quintero, PhD, MBA^3^, Antoni Ribas, MD, PhD^4^

###### ^1^University of California Los Angeles, Los Angeles, CA, USA; ^2^UCLA, Los Angeles, CA, USA; ^3^Bioncotech, Madrid, Spain; ^4^University of California, Los Angeles, Los Angeles, CA

####### **Correspondence:** Antoni Ribas (aribas@mednet.ucla.edu)

**Background**

We examined the impact of defects in type I and/or II interferon signaling on the efficacy of adoptive cell therapy (ACT) with tumor-specific T cells in B16 murine melanoma.

**Methods**

Using CRISPR, we generated B16 tumor cell lines deficient in type I (Ifnar1-KO) or type II (Jak2-KO) interferon signaling, or both (Jak1-KO). ACT was performed using gp100-specific pmel T cells. Tumor cell were labeled with RFP to determine tumor-specific MHC-I expression in vivo. We also tested a nanoplexed formulation of poly I:C (BO-112) that activates TLR3/MDA5/RIG-I.

**Results**

Pmel ACT was effective against B16 tumors lacking type I (Ifnar1-KO) or type II (Jak2-KO) interferon signaling, but ineffective against tumors lacking both (Jak1-KO) (Figure 1). We observed a similar phenomenon in vitro, where growth of B16 tumor cells lacking type I or II interferon signaling (but not both) were inhibited by tumor- specific pmel T cells compared to non-specific T cells, provided the alternate interferon pathway was activated (p < 0.0001). We then examined interferon-dependence of MHC-I expression in vivo. We observed that basal MHC-I expression by B16 in vivo is dependent on type II, but not type I, interferon signaling. After pmel ACT, tumors lacking type II interferon (Jak2-KO) signaling were able to augment MHC-I expression compared to B2m-KO tumors (p = 0.068). Jak1-KO tumors did not express MHC-I even after pmel ACT (p = 0.5283). To overcome resistance of B16 Jak1-KO tumor cells to pmel ACT, we tested intratumoral delivery of BO-112, which has direct anti-tumor efficacy against B16 and augments anti-tumor efficacy of pmel ACT against B16. Notably, the direct anti-tumor effects of BO-112 are abrogated in the B16 Jak1-KO compared to wildtype B16 tumors both in vitro and in-vivo. Regardless, in combination with BO-112, pmel ACT was effective against B16 Jak1-KO tumors compared to non-specific T cells in combination with BO-112 (Figure 2). RNA sequencing of tumors 5 days after ACT revealed 209 genes enriched (fold change > 2, adjusted p-value < 0.05) in tumors treated with pmel ACT and BO- 112, which were not enriched in tumors treated with pmel ACT and vehicle or non-specific ACT and BO-112, including genes involved in T cell recruitment, antigen presentation, direct T cell cytotoxicity, and interferon signaling.

**Conclusions**

Our findings suggest ACT can be an effective immunotherapy in tumors lacking type I or II interferon signaling. For tumors lacking both type I and II interferon signaling, intratumoral BO-112 can resensitize tumors to ACT.

**Fig. 1 (abstract P547).**
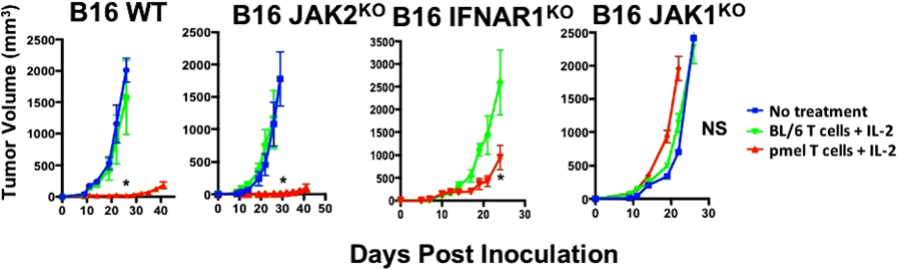
See text for description

**Fig. 2 (abstract P547).**
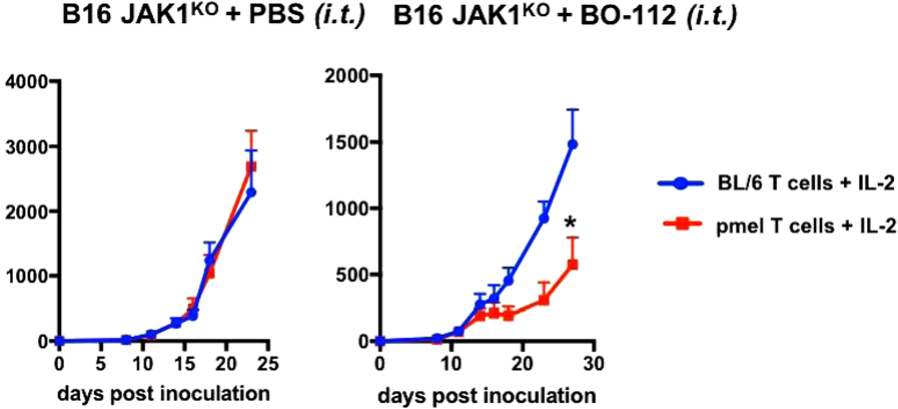
See text for description

#### P548 Association between corticosteroid use at time of immune checkpoint inhibitor (CPI) therapy initiation and CNS disease control/survival in patients with brain metastases (BM)

##### William Kelly, MD^1^, Neil Shah^1^, Michael Serzan, MD^1^, Barbara Ma^1^, Matthew Blackburn^1^, Sebastian Ochoa^1^, Alice Knoedler^1^, Jeevan Puthiamadathil^1^, Ariana Santopietro^1^, Subha Madhavan^2^, Anas Belouali^2^, Shruti Rao^2^, Kellie Gardner, NP^1^, Suthee Rapisuwon, MD^1^, Stephen Liu, MD^1^, Deepa Subramaniam^1^, Michael Atkins, MD^1^, Geoffrey Gibney, MD^1^

###### ^1^Medstar Georgetown University Hospital, Washington, DC, USA; ^2^Georgetown University, Washington, DC, USA

####### **Correspondence:** William Kelly (08wkelly@gmail.com)

**Background**

CPI therapy targeting CTLA-4 and PD-1 has improved clinical outcomes in patients with BM [1], including objective responses in patients (pts) with active, untreated melanoma or non-small cell lung cancer (NSCLC) BM [2-4]. Pts with newly diagnosed BM often require corticosteroids at CPI initiation and the impact of such concomitant therapy on disease control and survival remains unclear. The clinical outcomes of pts with BM receiving CPI therapy and associations with corticosteroids and other prognostic factors were analyzed.

**Methods**

Pts with metastatic solid tumors and BM history treated with ipilimumab (anti-CTLA-4), nivolumab or pembrolizumab (anti-PD-1), and nivolumab/ipilimumab (nivo/ipi) at 3 Medstar Hospitals were identified by pharmacy records and chart review. Pts were excluded if initial BM occurred after CPI initiation or if baseline pretreatment/follow up brain MRI/CT imaging were not available. Data collected included demographics, baseline performance status, systemic corticosteroid use within 14 days of CPI initiation, provider-assessed best disease response and overall survival (OS).

**Results**

71 pts were identified (40 melanoma, 25 NSCLC, 3 renal cell carcinoma, 3 other). 55% were male, 86% had ECOG PS 0-1, and 66% had ≥2 brain metastases. 82% of pts had surgery and/or stereotactic radiosurgery for BM management prior to therapy. 22% of pts received anti-CTLA-4, 54% received anti-PD-1, and 24% received nivo/ipi. 52% had neurological symptoms and 24% received corticosteroids within 14 days of CPI initiation. The response rate (extracranial) was 23% and median OS was 13.8months for all pts. Survival was superior in pts with melanoma and those treated with nivo/ipi. BM control (no new BM or progression in treated BM) was seen in 38%. Extracranial disease control was associated with intracranial disease control (p=0.001). The use of corticosteroids was associated with BM progression (but not extracranial disease progression) and worse OS (median 5.8months vs 19.8months for no corticosteroid use, P=0.011). There was a trend for worse OS in patients with greater number of BM (p=0.053). The presence of baseline neurological symptoms was not associated with OS.

**Conclusions**

Pts with BM can have long-term survival with CPI therapy, particularly with nivo/ipi. There is general concordance between extracranial disease control and BM control, but discordance with BM progression can occur. The use of corticosteroids at time of CPI therapy in pts with BM is associated with worse BM control and survival. Pts initially requiring corticosteroids may benefit from alternative systemic therapy options.

**References**

1. Sloot S, et al. Improved survival of patients with melanoma brain metastases in the era of targeted BRAF and immune checkpoint therapies. Cancer, 2018;124(2):297-305.
2. Goldberg S, et al. Pembrolizumab for patients with melanoma or non-small-cell lung cancer and untreated brain metastases: early analysis of a non-randomised, open-label, phase 2 trial. Lancet Oncol, 2016;17(7):976-983.
3. Long G, et al. Combination nivolumab and ipilimumab or nivolumab alone in melanoma brain metastases: a multicentre randomised phase 2 study. Lancet Oncol, 2018.
4. Margolin K, et al. Ipilimumab in patients with melanoma and brain metastases: an open-label, phase 2 trial. The lancet oncology, 2012.

#### P549 Cell proliferation defines an additional mechanism of immune escape in non-small cell lung cancer

##### Carl Morrison, MD, DVM^1^, Sarabjot Pabla, MSc, PhD, BS^1^, Mary Nesline, MS^1^, Jeffrey Conroy, BS^1^, Sean Glenn, PhD^1^, Antonios Papanicolau-Sengos, MD^1^, Paul DePietro, PhD^1^, Jacob Hagen^1^, Blake Burgher, BS, RN^1^, Vincent Giamo, BS, MS^1^, Jonathan Andreas, MS^1^, Felicia Lenzo^1^, Maochun Qin, MD, MS^1^, Yirong Wang, MS^1^, Devin Dressman, PhD^1^, Konstantin Dragnev, MD^2^, Laura Tafe^2^, Tian Zhang, MD^3^, Jeffrey Clarke, MD^3^, Matthew Zibelman, MD^4^, Luis de la Cruz-Merino^5^, Alexander MacKinnon^6^, Robin Jacob^7^, Oliver Binns^8^, Neel Shah^9^, Mark Gardner^1^, Grace Dy^10^

###### ^1^OmniSeq, Inc., Buffalo, NY, USA; ^2^Dartmouth-Hitchcock Medical Center, Hanover, NH, USA; ^3^Duke University, Durham, NC, USA; ^4^Fox Chase Cancer Center, Philadelphia, PA, USA ^5^Hospital Universitario Virgen Macarena, Sevilla, Spain ^6^Medical College of Wisconsin, Milwaukee, WI, USA ^7^Meharry Medical College, Nashville, TN, USA; ^8^Mission Health System, Asheville, NC, USA; ^9^Northwest Oncology, Munster, IN, USA; ^10^Roswell Park Comprehensive Cancer Center, Buffalo, NY, USA

####### **Correspondence:** Grace Dy (Grace.Dy@RoswellPark.org)

**Background**

Prior to this study, the most important mechanisms of immune escape in NSCLC include checkpoint blockade and low tumor mutational burden (TMB). In this study we have identified a third equally important mechanism of immune escape of cell proliferation in NSCLC.

**Methods**

169 NSCLC patients from ten institutions were evaluated for PD-L1 expression by IHC, TMB, and cell proliferation by RNA-seq of 10 immune and proliferation related genes including BUB1, CCNB2, CDK1, CDKN3, FOXM1, KIAA0101, MAD2L1, MELK, MKI67, and TOP2A, of which 46 represented historical controls and 113 had prior treatment with one more immune checkpoint inhibitors (ICIs) and for which response (RECIST v1.1) and survival were available.

**Results**

For all patients (n=169) the majority were non-proliferative (59.2%; 100/169), with a minority of proliferative results (40.8%; 69/169). Response rate in non-proliferative patients was 42.2% (27/64) versus 18.4% (9/49) in proliferative. Using a value of 10 mutations per megabase of DNA or greater as high the percent of TMB high cases in proliferative tumors (67%; 47/70) was higher than in non-proliferative tumors (47%; 47/99). The rate of PD-L1 IHC positive (TPS >50) was not different in proliferative (21.7%; 15/69) versus non-proliferative (26%; 26/100) supporting that PD-L1 expression is independent of proliferation status. Survival analysis using TMB high versus low did not show a survival advantage for all ICI-treated patients (p=0.7), proliferative cases (p=0.057), or non- proliferative cases (p=0.12). For 18 non-proliferative PD-L1 positive cases the response rate was 67% (12/18) and nearly double that in the corresponding proliferative group at 38% (5/14). For 46 non-proliferative PD-L1 negative cases the response rate was 35% (12/18) and more than three times that in the corresponding proliferative group at 11% (4/35). Survival for all combinations of PD-L1 and cell proliferation was greatest for PD-L1 positive non- proliferative cases, but perhaps equally if not more important was survival for PD-L1 negative non-proliferative cases was nearly equal to that for PD-L1 proliferative cases.

**Conclusions**

Cell proliferation, or more specifically high proliferation, is an important immune escape mechanism in NSCLC. Non-proliferation is an emerging biomarker for response to ICIs in NSCLC.

**Ethics Approval**

OmniSeq’s analysis utilized deidentified data that qualified as non-human subject research under IRB protocol (BDR #080316) approved by Roswell Park Comprehensive Cancer Center (Buffalo, NY).

#### P550 Defeating checkpoint resistance: Highly specific inhibition of latent TGFβ1 activation renders resistant solid tumors vulnerable to PD-1 blockade

##### Thomas Schürpf^1^, Constance Martin, PhD^1^, Christopher Littlefield, MSc^1^, Christopher Chapron, MS^1^, Stefan Wawersik, PhD^1^, Ashish Kalra, PhD^1^, Kevin Dagbay, PhD^1^, Allison Simpson, BS^1^, Francis Danehy, BS^1^, Christopher Boston^1^, Anastasia Nikiforov, MS^1^, Susan Lin, BS^1^, Justin Jackson, BS^1^, Pichai Raman, PhD^2^, Elizabeth Rainbolt, BS^3^, Laurie Comfort, BS^3^, David Harris^3^, Madelyn Cecil-Taylor^3^, Lorne Celentano^3^, Danielle Meadows^3^, gregory carven, PhD^1^, Alan Buckler, PhD^1^, Allan Capili, PhD^1^, Abhishek Datta, PhD^1^, Thomas Schürpf^1^

###### ^1^Scholar Rock, Cambridge, MA, USA; ^2^Pichai Raman Consulting, Bryn Mawr, PA, USA; ^3^Charles River Discovery Services, Morrisville, NC, USA

####### **Correspondence:** Abhishek Datta (adatta@scholarrock.com)

**Background**

Despite the clinical breakthroughs achieved by cancer immunotherapy, a majority of patients fail to respond to PD-(L)1 inhibition due to primary or acquired resistance. Profiling of human urothelial cancer and melanoma tumors has recently implicated TGFβ activation as a potential mechanism of primary resistance to checkpoint therapies. However, therapeutic targeting of the TGFβ pathway has been hindered by dose-limiting cardiotoxicities, most likely due to inhibition of signaling from multiple TGFβ isoforms. Upon secretion, TGFβ growth factor is held in a latent complex with its non-covalently associated prodomain. TGFβ activation is induced by extracellular events that release the growth factor from this latent complex. We previously demonstrated that isoform-specific inhibition of TGFβ activation can be achieved by targeting the latent TGFβ complex. We hypothesized that the identification and inhibition of the predominant TGFβ isoform in tumors would enable a more targeted and potentially safer approach to TGFβ inhibition.

**Methods**

The Cancer Genome Atlas (TCGA) database was interrogated to assess mRNA levels of TGFβ isoforms. Antibody- mediated inhibition of TGFβ1 activation was tested using luciferase-based reporter cells. Efficacy of TGFβ1-selective inhibition in combination with anti-PD-1 was assessed in the MBT-2 bladder cancer and CloudmanS91 melanoma models.

**Results**

Bioinformatic analysis of TCGA data identified TGFβ1 as the predominant isoform in many human tumors. We generated high affinity, fully-human antibodies against latent TGFβ1. They inhibit the activation of latent TGFβ1 with no detectable binding to or inhibition of latent TGFβ2 or latent TGFβ3. Efficacy was tested in MBT-2 and CloudmanS91, two syngeneic mouse models that recapitulate key aspects of the primary PD-1 resistance phenotype of human disease. Inhibition of TGFβ1 activation is sufficient to completely block TGFβ signaling in MBT-2 tumors. Both models are largely resistant to anti-PD-1 or anti-TGFβ1 alone. However, the combination of anti-PD-1 with blockade of TGFβ1 activation leads to tumor growth delay, a substantial number of complete responses, and prolonged survival coupled with increased effector CD8+ T cell infiltration.

**Conclusions**

We show here that in many human tumor types, especially those for which checkpoint inhibitors are approved as therapies, TGFβ1 is the predominant isoform. Pharmacologic blockade of TGFβ1 activation is sufficient to sensitize TGFβ1-predominant tumors to PD-1 inhibition. These encouraging efficacy data and the potentially favorable safety profile of TGFβ1 isoform-selective inhibition establish a strong rationale for exploring therapeutic application of combining PD-(L)1 blockade with latent TGFβ1 inhibition in treatment of multiple cancer types.

**Ethics Approval**

Animal studies were conducted in compliance with CR Disovery Services IACUC ASAP # 980701 & #980702, and AAALAC Certification

#### P551 Suppression of immune response by tumor cell-induced XIAP-NFκB signaling and targeting strategies to overcome immunotherapy resistance in breast cancer

##### Michael Morse^1^, Scott Sauer, PhD^2^, Myron Evans^3^, Mohamed Ibrahim, MD^2^, Xuhui Bao, MD^2^, Pranalee Patel^2^, Gayathri Devi, MSc, PhD^2^

###### ^1^Duke University Medical Center, Durham, NC, USA; ^2^Duke University School of Medicine, Durham, USA; ^3^St. Jude's, Memphis, USA

####### **Correspondence:** Gayathri Devi (gayathri.devi@duke.edu)

**Background**

Locally advanced breast cancers (LABC) that display lymphovascular invasion (LVI), such as inflammatory breast cancer (IBC), rapidly acquire therapeutic resistance and are highly lethal. A critical question is how, despite trafficking through lymphatics where they encounter immune effectors and inflammatory stress, do the tumor cells evade immune-mediated cell death. IBC expresses high levels of the anti-apoptotic protein, X-linked inhibitor of apoptosis protein (XIAP). We previously identified that, in addition to its canonical function as a potent caspase inhibitor in both the extrinsic and intrinsic apoptosis pathways, XIAP activates nuclear transcription factor (NFκB) in suppression of two mechanisms of cell death caused by anti-tumor immune effectors (antibodies and T cells), granzyme-mediated cell death and accumulation of reactive oxygen species. Further, we identified a mechanism of stress-induced protein translational upregulation of XIAP in promoting tumor cell survival in models of IBC. Therefore, we hypothesized that stress-mediated XIAP-NFκB signaling can lead to a tumor cell-promoted immunosuppressive environment and targeting this signaling axis can enhance the efficacy of immunotherapy.

**Methods**

Using multiple cell models of aggressive breast cancer, we observed that increased XIAP expression caused decreased immune-mediated caspase activation, lower ROS induction/increased antioxidant protein, and NFκB target gene transcripts in the immune/inflammatory network. To directly test if increased XIAP-NFκB survival signaling can suppress efficacy of immunotherapy, we tested the FDA approved EGFR-specific monoclonal antibody (cetuximab), widely used in cancer therapy, in in vitro antibody-dependent cellular cytotoxicity (ADCC) assays and in in vivo tumor growth kinetic analysis of IBC cells with differential apoptotic capability when implanted orthotopically in the mammary fat pad of mice with functional NK activity.

**Results**

IBC cells with XIAP overexpression and resultant increases in NFκB target genes regulating antioxidant and immune factors were insensitive to cetuximab-mediated ADCC and resistant to cetuximab-mediated inhibition of in vivo tumor growth when compared to the ADCC-sensitive cell lines. In order to re-sensitize these XIAP overexpressing cells towards ADCC, we tested two strategies- 1. targeting XIAP-NFkB signaling using NRAGE peptide that blocks the XIAP-Tab1-Tak1 complex; 2. SMAC mimetics/birinapant, a synthetic small molecule and peptidomimetic of second mitochondrial-derived activator of caspases (SMAC) and inhibitor of IAP family proteins. Our results reveal enhanced immune-mediated cell death/sensitivity to immunotherapy.

**Conclusions**

Our in vitro and in vivo preclinical studies identify the cellular stress-mediated induction of the XIAP-NFκB signaling axis as a novel mechanism of immune evasion and reveal the potential of targeting this signaling pathway to improve breast cancer immunotherapy.

**Acknowledgements**

Supported in part by Department of Defense Partnership Idea grant awards [W81XWH-13-1-0046 (GRD); W81XWH-13-1-0046 (MAM)]; Duke School of Medicine Bridge Funds (GRD); Duke University Diversity Enhancement Fellowship (MKE) and the National Cancer Institute T32CA009111 (SJS).

**Ethics Approval**

The animal research was approved by the Duke University IACUC

#### P552 Analysis of TIL from human carcinoma combined with tissue imaging and in vitro models uncovers tumor- inflicted T cell deviations related to immune escape and strategies of intervention

##### Elfriede Noessner, PhD^1^, Elfriede Noessner, PhD^1^, Petra Prinz^2^, Ilias Masouris^3^, Anna Mendler^2^

###### ^1^Helmholtz Zentrum Munchen, Munich, Germany; ^2^Helmholtz Zentrum München, Munich, Germany; ^3^Klinikum LMU München, München, Germany

####### **Correspondence:** Elfriede Noessner (noessner@helmholtz-muenchen.de)

**Background**

Many tumors are infiltrated with CD8 lymphocytes. Yet, tumors are not rejected suggesting that the tumor environment limits effector cell efficacy to control tumor growth.

**Methods**

Using human renal cell carcinoma, multiparameter fluorescence staining and confocal microscopy was performed to determine the status of lymphocytes in direct physical contact with malignant cells under the control of the local microenvironment. Ex vivo TIL analysis was used to identify TCR signaling alterations in CD8-TILs compared to CD8 T cells of non-tumor kidney.

**Results**

A special image analysis, modeled on the process of lytic granule exocytosis, was applied to identify CD8-TILs with active tumor recognition. The cytotoxic status of CD8-TILs, determined in relation to the TILs’ spatial distribution within the tumor, revealed a pivotal role of the tumor microenvironment in restraining lytic function of CD8-TILs. Some TILs appeared actively engaged in tumor recognition; however, there was no evidence that any CD8 cell was stimulated to produce IFNg. Application of in vitro models, which mimic conditions of solid tumors, identified tumor lactic acidosis as one potent factor abrogating TCR-stimulated IFNg production by inhibition of p38 and JNK/c-Jun activation. Ex vivo analyses of TILs identified TCR signaling alterations in CD8-TILs compared to CD8 T cells of non-tumor kidney, which were associated with failure to degranulate. These deviations were reversible concomitantly with gain in perforin and function.

**Conclusions**

The results reveal mechanisms of inhibition of CD8- and NK-TIL function imparted by the tumor environment which are related to immune escape in RCC. Based on identified alterations strategies to engineer T cells (i.e chimeric costimulatory proteins) and to modulate the tumor environment are designed to empower T cells for higher efficacy in adoptive therapy.

**Acknowledgements**

We acknowledge the patients and their families for donating tissue for analysis, and the clinicians and pathologists for the sample collection.

**Ethics Approval**

The ethics board of the Ludwig Maximilians University has approved the tissue collection

#### P553 Targeting mechanisms of immune suppression via CXCR2 inhibition to enhance checkpoint blockade

##### Elaine Pinheiro, PhD, Sarah Javaid, PhD, Ruban Mangadu, Marlene Hinton, Yun Wang, Sonia Feau, Amanda Watkins, PhD, Andrey Loboda, PhD, Xue Liang, Dario Gutierrez, Rebecca Ruck, Michael Rosenzweig, DVM, PhD, Vincent Giranda, MD, PhD

###### Merck Research Laboratories, Boston, MA, USA

####### **Correspondence:** Elaine Pinheiro (elaine.pinheiro@merck.com)

**Background**

Myeloid-derived suppressor cells (MDSCs) are an adverse cancer-wide prognostic population of immune infiltrating cells that contribute to tumor immune evasion by suppressing local T-cell activation and viability and influencing tumor progression by promoting tumor metastases, angiogenesis and tumor cell invasion [1]. PMN-MDSC cell signatures have emerged as a significant predictor of poor survival across solid tumors [2]. Here we explore the role of the CXCR2 pathway on PMN-MDSCs in the tumor microenvironment and the mechanisms of CXCR2-driven enhancement of checkpoint blockade.

**Methods**

Chemotaxis assays were performed. Anti-tumor activity was assessed in the B16-F10 syngeneic tumor model utilizing a small molecule antagonist of CXCR2, MK-7123 (Navarixin) and an anti-mouse PD-1 blocking antibody, muDX400. Molecular and cellular responses associated with anti-tumor activity were evaluated by RNA- Sequencing analysis, flow cytometry, and functional assays.

**Results**

Here we show that cancer patient PMN-MDSCs highly express the CXC G protein-coupled receptor, CXCR2, and that its chemokine ligand, IL-8, can influence the migration of this myeloid population. In addition, CXCR2 expression significantly correlates with poor survival across solid tumor types. In mouse tumor models, CXCR2 blockade disrupts the trafficking of CD11b(+)Ly6C(-)Ly6G(+) PMN-MDSCs/neutrophils in the tumor microenvironment and enhances the anti-tumor effect of checkpoint blockade. Preclinical studies to further explore the molecular and cellular mechanisms behind these combination effects will be presented, utilizing a small molecule antagonist of CXCR2, MK-7123 (Navarixin), and an anti-mouse PD-1 blocking antibody, muDX400. Tumor transcriptome and network analyses by RNA-sequencing reveal that MK-7123 + muDX400 combination treatment result in the enrichment of immune and tumor-related pathways. Moreover, changes to chemokine profiles favor enhanced T cell infiltration into the tumor. In addition, the effects of commensal microbiota on response to monotherapy and combination treatment will be explored.

**Conclusions**

Taken together, these preclinical oncology studies support the concept of targeting CXCR2 to increase the therapeutic efficacy of PD-1 blockade. Clinical investigation of Navarixin in combination with pembrolizumab/Keytruda is ongoing for the treatment of multiple cancers.

**References**

1. Veglia F, Perego M, Gabrilovich D. Myeloid-derived suppressor cells coming of age. Nat Immunol. 2018;19:108–119 2. Gentles AJ, Newman AM, Liu CL, Bratman SV, Feng W, Kim D, Nair VS, Xu Y, Khuong A, Hoang CD, Diehn M, West RB, Plevritis SK, Alizadeh AA. The prognostic landscape of genes and infiltrating immune cells across human cancers.Nat Med. 2015; 21(8):938-945

#### P554 Role of immune escape for resistance to cancer (immuno) therapy and its strategies targeting these mechanisms

##### Barbara Seliger, MD, PhD, Jurgen Bukur, PhD, MD

###### Martin Luther University Halle-Wittenber, Halle, Germany

####### **Correspondence:** Jurgen Bukur (juergen.bukur@uk-halle.de)

**Background**

Despite impressive durable clinical responses in tumor patients with distinct subtypes of cancer employing cancer immunotherapies, a high frequency of patients does not respond or develop resistances during treatment overtime. Therefore, identification of the underlying molecular mechanisms of these resistances as well as identification of novel therapeutic approaches to overcome them might significantly improve the clinical outcome and survival of patients.

**Methods**

A number of both tumor intrinsic as well as tumor extrinsic factors have been identified by us, which are involved in the escape of immune surveillance of different tumors.

**Results**

These include downregulation of MHC class I antigen processing and interferon signaling components, upregulation of co-inhibitory molecules, such as HLA-G and PD-L1, as well as downregulation of extracellular matrix proteins. These different alterations could occur either at the transcriptional, epigenetic or posttranscriptional level, while structural alterations leading to loss of expression of these immune modulatory molecules appear to be a rare event. Interestingly, impaired HLA class I APM component expression has been demonstrated to be directly associated with disease progression after adoptive T cell therapy. Next to these tumor intrinsic factors, the tumor microenvironment also plays an important role in immune escape. In particular, the immune cell compositions in peripheral blood as well as the spatial distribution of immune cells in the tumor microenvironment are key factors of immune suppression. This was directly associated with a worse prognosis, reduced survival and/ or lack of response to cancer (immuno)therapies. Furthermore, treatment of cells with cytokines, like interferons, as well as recombinant proteoglycans anti-oxidant substances, e.g. methyl selenic acid, and epigenetic drugs were able to enhance HLA class I surface expression thereby resulting in an enhanced immune response.

**Conclusions**

Thus, overcoming the acquired an intrinsic therapy resistance of tumors is an important tool for improving (immuno)therapeutic strategies.

#### P555 A role for mutant p53 in mediating T cell immune evasion in pancreatic adenocarcinoma and other solid tumors

##### Deborah Silverman, BS, Emily Ashkin, Simone Punt, PhD, Minying Zhang, Leila Williams, MSc, Anil Korkut, Jason Roszik, PhD, Anirban Maitra, MBBS, Patrick Hwu, MD

###### MD Anderson Cancer Center, Houston, TX, USA

####### **Correspondence:** Deborah Silverman (DASilverman@mdanderson.org)

**Background**

Harnessing the immune system by altering the ability of T-cells to attack cancer has led to long-lasting cures in some tumors. Nonetheless, pancreatic ductal adenocarcinoma (PDAC), one of the most lethal malignancies, remains resistant to immunotherapy. To design effective therapies, it is critical to learn how PDAC evades the immune system.

**Methods**

We hypothesize that p53 mutations mediate tumor escape from T-cells in PDAC, and test whether subsets of p53 mutations in PDAC tumors impact T-cell migration and killing, using novel in vitro and inducible in vivo models, to characterize a new role for p53’s regulation in cancer.

**Results**

Here we present results suggesting that a subset of p53 missense mutants impact T-cell infiltration into and autologous T-cell killing in PDAC tumors, in a possible dominant gain-of-function mechanism.

**Conclusions**

These findings begin to elucidate the role of p53 activating mutations in tumorigenesis,and providing rationale to investigate mutant p53 immune-regulation in PDAC and other tumor types.

**Acknowledgements**

The authors thank and acknowledge Eran Kotler and Moshe Oren for generously providing H1299-p53-mutant lines; Gigi Lozano, Florencia McAllister, Russell Broaddus, Stephanie Watowich, and Katy Rezvani for their valuable advice and insight; Sara Leahey, Soraya Zorro Manrique, Elien Doordjuin, and Weiyi Peng for their advice and support; and the University of Texas MD Anderson Cancer Center Pancreatic Cancer Moon Shot Program for their generous financial support of this project.

**References**

1. Carter S L, Cibulskis K, Helman E, McKenna A, Shen H, Zack T, Beroukhim R. Absolute quantification of somatic DNA alterations in human cancer. Nat biotechnology. 2012; 30(5): 413-421.
2. Hingorani S R, Wang L, Multani A S, Combs C, Deramaudt T B, Hruban R H, Tuveson D A. Trp53R172H and KrasG12D cooperate to promote chromosomal instability and widely metastatic pancreatic ductal adenocarcinoma in mice. Cancer cell. 2005; 7(5), 469-483.
3. Morton J P, Timpson P, Karim S A, Ridgway R A, Athineos D, Doyle B, Frame M C. Mutant p53 drives metastasis and overcomes growth arrest/senescence in pancreatic cancer. PNAS. 2010; 107(1), 246-251.
4. Aschauer L, Muller P A. Novel targets and interaction partners of mutant p53 Gain-Of-Function. Biochem Soc Transactions. 2016; 44(2), 460-466.
5. Joerger,A C, Fersht A R. Structure–function–rescue: the diverse nature of common p53 cancer mutants. Oncogene. 2007; 26(15), 2226-2242.
6. McKenzie, J A, Mbofung, R M ., Malu, S, Zhang, M, Ashkin, E, Devi, S, & Xu, C. The Effect of Topoisomerase I Inhibitors on the Efficacy of T-Cell-Based Cancer Immunotherapy. J Natl Cancer Inst. 2018;110(7):777-786.

#### P556 STAT3-related cytokines drive IR-specific immune suppression of effector, memory and naïve, peripheral blood CD8+ T cells in cancer patients

##### Ashwin Somasundaram, MD, Dario A. Vignali, PhD, Anthony Cillo, PhD, James Herman, John Kirkwood, MD, Robert Ferris, MD, PhD, Tullia Bruno, PhD

###### University of Pittsburgh, Pittsburgh, PA, USA

####### **Correspondence:** Dario A. Vignali (dvignali@pitt.edu)

**Background**

Cancer patients that do not respond to PD1 blockade have increased inhibitory receptor (IR) expression in peripheral blood lymphocytes (PBL) and increased cytokine concentrations in the plasma. Cancer patients off therapy and with normal white blood cell counts are often at greater risk for infections, immune dysregulation, progressive disease or reactivation of viral infections. However, the exact mechanism of this systemic immunosuppression in cancer patients is not fully understood. We performed flow cytometric assays to assess both phenotype and function of peripheral CD8+ T cells in cancer patient samples and healthy donor controls. We hypothesize that cancer patients may have systemic immune suppression via cytokine-driven IR expression in all CD8+ T cells subsets, including naïve cells.

**Methods**

PBL were obtained from healthy donors and treatment-naïve NSCLC, HNSCC, and melanoma patients. IR (i.e. LAG3, PD1, CTLA4, etc) expression was assessed on CD8+ T cells, CD4+ T cells, and regulatory T cells. Cytokine concentrations were compared by Luminex between plasma from healthy donors and plasma from cancer patients with high and low IR expression on peripheral CD8+ T cells. Autologous micro-stimulation assays were performed on peripheral CD8+ or CD4+ T cells with antigen presenting cells plus or minus IR blockade.

**Results**

CD8+ T cells, including CD45RA+CCR7+CD62L+CD8+ T cells, from cancer patient PBL contain elevated total LAG3 expression which correlated with stage and elevated expression of other IRs. Further, CD8+ T cells from these patients had decreased proliferation, which was rescued with the addition of anti-LAG3 or anti-PD1. Plasma from these patients had significantly elevated levels of cytokines that can signal via STAT3 (i.e. IL-6, IL-8, IL-9), which were independently found to increase total IR expression in healthy donor, naïve CD8+ T cells.

**Conclusions**

The current understanding of PD1 blockade resistance has been limited to the tumor microenvironment (TME) and our findings support the growing body of literature that tumor-related systemic immune suppression is a potent mechanism of cancer progression. Patients with cancer have systemic elevations of cytokines that signal via STAT3 leading to increased IR expression in naïve, peripheral CD8+ T cells making them poised for exhaustion even before TCR binding. These findings suggest that IR blockade also plays a significant role in reversing immune tolerance outside of the TME and cytokine blockade may play a role in reversing PD1 blockade resistance.

**Ethics Approval**

The study was approved by the University of Pittsburgh's IRB and Ethics Board, approval number: PRO16070383.

#### P557 Overcoming genetically-based resistance mechanisms to PD-1 blockade

##### Davis Torrejon, MD^1^, Gabriel Abril-Rodriguez, MS^2^, Jennifer Tsoi^2^, Ameya Champhekar^2^, Giulia Parisi^2^, Gardenia Cheung-Lau^2^, Tom Wohlwender^2^, Mykola Onyshchenko^2^, Beata Berent-maoz^2^, Catherine Grasso^2^, Begoña Comin- Anduix, PhD^2^, Siwen Hu-Lieskovan, MD, PhD^2^, Antoni Ribas, MD, PhD^2^

###### ^1^UCLA Hematology-Oncology, Los Angeles, CA, USA; ^2^UCLA, Los Angeles, CA, USA

####### **Correspondence:** Antoni Ribas (aribas@mednet.ucla.edu)

**Background**

We studied loss of function (LOF) mutations within the interferon (IFN) pathway (JAK1 or JAK2) and in the antigen presentation pathway (beta-2-microglobulin-B2M) found in biopsies from patients who are resistance to anti-PD-1 therapy, and tested strategies to overcome the resistance.

**Methods**

Using CRISPR/Cas9 genome editing we generated JAK1, JAK2 and B2M knockout (KO) sublines of the murine MC38 carcinoma, a model of high mutational load cancer that responds well to anti-PD-1, as well as of human MART-1-positive melanoma cell lines, tested using in-vitro T cell co-culture systems. We analyzed signaling changes in human cell lines (parental and KOs) exposed to IFN-gamma using RNAseq. In addition, we performed in-vivo antitumor activity in the MC38 variants using mass cytometry (CyTOF) to characterize the tumor microenvironment. Finally, we tested strategies to overcome resistance mechanisms with SD-101 (TLR-9-agonist) and NKTR-214 (CD-122 biased agonist).

**Results**

The JAK1-KO sublines lost sensitivity to IFN-alpha, IFN-beta and IFN-gamma, while the JAK2-KO cell line was insensitive only to IFN-gamma induced signaling (PD-L1, MHC class I) and growth arrest (p<0.001 compared with IFN-alpha or beta). There was no difference in the in-vitro cytotoxicity by MART-1 specific T-cells against JAK1/2- KO-MART-1+ melanoma cells compared to the parental (94%, 95% vs 90% cytotoxicity at 10:1 E:T ratio, pNS). However, B2M-KO was resistant to killing by MART-1 specific T-cells (2% vs 90% cytotoxicity at 10:1 E:T ratio, p<0.0001). RNAseq differential gene expression analysis showed that the IFN-gamma-induced increased expression of antigen presenting machinery, IFN-gamma signaling and chemokines (CXCL9, CXCL10) were not expressed by JAK1/2-KOs. In the MC38 model, the significant antitumor activity of anti-PD-1 against the parental was lost in JAK1/2 and B2M KOs (Table1); in these KO sublines, CyTOF analysis revealed that anti-PD-1 therapy was unable to change tumor CD8 T-cell infiltration. Using JAK1/2-KOs cell lines we showed that intratumoral administration of the TLR-9 agonist SD-101 was able to overcome local resistance to anti-PD-1 even in abscopal sites, and the NKTR-214 overcame resistance to anti-PD-1 in the B2M-KO tumor growth and significantly increased survival.

**Conclusions**

JAK1/2 LOF mutant tumors result in loss of sensitivity to IFN induced antitumor effects but do not impair T cell recognition and cytotoxicity, while B2M LOF results in lack of antigen presentation to T cells and loss of antitumor activity. Both lead to in-vivo resistance to anti-PD-1 therapy, and JAK1/2 KO resistance can be overcome by a TLR9 agonist, and B2M-KO resistance can be overcome by a new generation IL-2.

**Table 1 (abstract P557).**
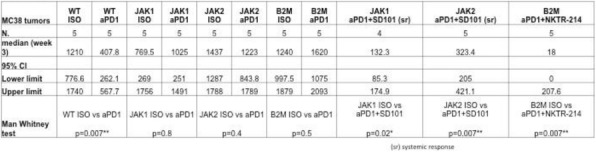
Overcome resistance to PD-1 blockade

#### P558 Secondary resistance to immunotherapy associated with β-catenin pathway activation or genetic loss of phosphatase and tensin homolog (PTEN) in metastatic melanoma

##### Jonathan Trujillo, MD, PhD^2^, Jason Luke, MD, FACP^1^, Stefani Spranger, PhD^3^, Yuanyuan Zha, PhD^1^, Karen Matijevich, RN, BSN^2^, Thomas Gajewski, MD, PhD^1^

###### ^1^University of Chicago, Chicago, IL, USA; ^2^Univeristy of Chicago, Chicago, IL, USA; ^3^MIT, Cambridge, MA, USA

####### **Correspondence:** Thomas Gajewski (tgajewsk@medicine.bsd.uchicago.edu)

**Background**

While immune checkpoint blockade therapy and even some vaccines have given rise to durable responses in many cases of advanced melanoma, a large fraction of patients subsequently develop secondary resistance to therapy. Mechanisms of immune-resistant cancer progression in this context are incompletely understood. Our lab previously showed tumor-intrinsic WNT/β-catenin activation can mediate T cell exclusion from tumor and primary resistance to anti-CTLA-4 + ant-PD-L1 therapy [1,2]. In addition, genetic loss of the tumor suppressor PTEN has been associated with defective T cell infiltration and primary resistance to PD-1 blockade [3]. However, whether secondary resistance might occur upon acquired oncogenic pathway alterations following initial response to immunotherapy is not known. We describe two metastatic melanoma patients who had a durable response to immunotherapy, but subsequently developed secondary resistance characterized by a phenotypic shift from a T cell- inflamed to non-T cell-inflamed tumor microenvironment, associated with oncogenic pathway alterations.

**Methods**

Case 1: A patient with metastatic melanoma was treated on a peptide/interleukin-12 vaccine protocol every 3 weeks for one year and achieved a partial response. Three years later a new metastatic lesion developed. Tumor gene expression profiling and histologic analysis for CD8 T cell infiltration and β-catenin expression were performed at baseline and at recurrence. Case 2: A patient with metastatic melanoma was treated with anti-CTLA-4 + PD-1 therapy and achieved a major partial response for a total of nineteen months. Baseline and treatment-resistant tumors underwent next-generation sequencing comprising a panel of commonly altered cancer genes for mutational and copy number analysis. Tumor biopsies were examined for CD8 T cell infiltration.

**Results**

Case 1: The baseline tumor prior to peptide vaccination demonstrated a T cell-inflamed gene signature and a robust intratumoral CD8 T cell infiltrate. In contrast, the recurrent treatment-resistant metastasis had a non-T cell inflamed phenotype and no infiltrating CD8 T cells. The new metastasis also acquired extensive expression of β-catenin, which was undetectable in the baseline lesion. Target antigens and circulating tumor-reactive T cells were detectable at the time of progression. Case 2: The on-treatment biopsy during anti-CTLA-4 + PD-1 therapy showed intratumoral CD8 T cells, while the recurrent metastasis lacked infiltrating CD8 T cells. The treatment-resistant metastasis uniquely harbored biallelic PTEN loss with no detectable PTEN protein present.

**Conclusions**

Our findings suggest that secondary resistance to immunotherapy may arise when tumor up-regulates β-catenin expression or undergoes genetic loss of PTEN, oncogenic events capable of driving T cell exclusion from the tumor microenvironment.

**References**

1. Spranger S, Bao R, Gajewski TF. Melanoma-intrinsic beta-catenin signalling prevents anti-tumour immunity. Nature 2015; 523:231-5.
2. Spranger S, Dai D, Horton B, Gajewski TF. Tumor-residing Batf3 dendritic cells are required for effector T cell trafficking and adoptive T cell therapy. Cancer Cell. 31:711-23.e4.
3. Peng W, Chen JQ, Liu C, et al. Loss of PTEN promotes resistance to T cell-mediated immunotherapy. Cancer Discovery. 2016; 6:202-16.

**Ethics Approval**

The study was approved by University of Chicago's Ethics board.

**Consent**

Consent was received

#### P559 Co-expression of TNF receptor 1 and 2 on human BRAF V600E+ melanomas is required for TNF-induced resistance to MAPK pathway inhibitors

##### Lazar Vujanovic, PhD, Cindy Sander, BS, Jian Shi, MD, John Kirkwood, MD, Lisa Butterfield, PhD

###### University of Pittsburgh, Pittsburgh, PA, USA

####### **Correspondence:** Lazar Vujanovic (vujanovicl@upmc.edu)

**Background**

The effectiveness of MAPK cascade-targeting therapies to treat patients with BRAF-V600E-mutant melanomas has been limited by a range of resistance mechanisms that may be driven by the tumor necrosis factor (TNF). TNF signaling is mediated through TNF receptor type-1 (TNFR1) and TNF receptor type-2 (TNFR2). TNFR1 signaling mediates apoptosis or cell survival/cytokine secretion, while TNFR2 selectively mediates cell survival/cytokine secretion. Although TNFR1 and TNFR2 are preferentially activated by soluble (sol)TNF and transmembrane (tm)TNF, respectively, they can crosstalk via shared signaling molecules. While TNF receptor 1 (TNFR1) is ubiquitously expressed, little is known about the expression patterns and functional roles of TNFR2 on melanomas.

The primary goals of this study are to evaluate whether TNFR2 is expressed on melanoma, to determine which TNFR mediates TNF- mediated resistance reprogramming to MAPK inhibitors (MAPKi) and to decipher whether INB03, a dominant-negative TNF biologic and specific antagonist of solTNF, can antagonize this therapeutic resistance pathway.

**Methods**

TNFR1/2 expression patterns on BRAF-mutant melanomas were evaluated by multi-color flow cytometry. Recombinant TNF was used to induce MAPKi resistance in melanomas. Activated human macrophages were used in transwell co-culture systems to induce MAPKi resistance in melanomas. The effectiveness of INB03 to antagonize this therapeutic resistance pathway was compared to an anti-TNF antibody and a selective NF-kB inhibitor. CRISPR/Cas9 system was utilized to edit out TNFR1 and TNFR2 on a melanoma cell line, and these knockout variants were used to test the intrinsic roles of these receptors in TNF-induced resistance to MAPKi. MTT viability assay was used as the readout for melanoma sensitivity to MAPKi.

**Results**

TNFR1 and TNFR2 were co-expressed by 48% of BRAF-V600E-mutant melanoma cell lines and primary melanomas. Interestingly, only cell lines that co-expressed TNFR1 and TNFR2 could acquire MAPKi resistance in response to recombinant and macrophage-derived TNF. Functional studies of TNFR1 and TNFR2 knockout cell lines indicated that both TNFR1 and TNFR2 signaling were necessary for the TNF-mediated induction of resistance to MAPKi. Finally, selective sequestration of both recombinant and macrophage-derived TNF using INB03 effectively prevented acquisition of resistance to MAPKi by BRAF-V600E mutant melanoma cell lines in vitro.

**Conclusions**

solTNF-mediated induction of MAPKi resistance in BRAF-V600E-mutant melanomas is predicated on the co-expression of TNFR1 and TNFR2. Our data indicate that almost half of BRAF-V600E-mutant melanomas express TNFR2. These results indicate that TNFR2 could be a biomarker that could be used to select for melanoma patients that could benefit from TNF-targeting therapies.

**Acknowledgements**

The authors thank David E. Szymkowski, Ph.D. of Xencor Inc. for providing the dominant-negative TNF biologic. This study was supported by the UPCI SPORE in melanoma and skin cancer (P50 CA121973) Developmental Research Project (DRP) award.We thank the following UPCI shared resources (supported in part by NIH P30CA047904): Flow Cytometry Facility and the Immunologic Monitoring Laboratory (Luminex).

**Ethics Approval**

Specimens collection was performed under IRB-approved protcol UPCI-96-099

#### P560 Resistance of CD44+ subpopulation to CTL though high production a protease inhibitor in colorectal cancer

##### Tomonori Yaguchi, MD, PhD, Tsubasa Miyauchi, Kenji Morii, MS, Yutaka Kawakami, MD PhD

###### Keio University School of Medicine, Tokyo, Japan

####### **Correspondence:** Yutaka Kawakami (yutakawa@keio.jp)

**Background**

Colorectal carcinoma (CRC) is generally resistant to immunotherapies, suggesting possible CRC-specific immunosuppressive mechanisms. In this study, we have identified markers which could define particular subpopulation harboring immuno-resistant properties and investigated the underlying mechanisms of the immunosuppression.

**Methods**

We analyzed the expression pattern of 30 CD (cluster of differentiation) antigens on 10 human CRC cell lines. We sorted a CRC cell line into the CD44-positive fraction and the CD44-negative fraction, and evaluated their sensitivity to CTL lysis. Gene expression profiles of the CD44-positive CRC fraction which showed resistance to CTL lysis were compared with those of the CD44-negative CRC fraction using the cDNA micro array. For the functional analysis of protease inhibitor X (PI-X) which was preferentially expressed in CD44-positive fraction, we evaluated the effect of PI-X knockdown by siRNA or PI-X overexpression in CRC cell lines on the sensitivity to CTL lysis in vitro and the effect of PI-X overexpression in murine CRC cells on the therapeutic efficacy of anti PD- 1 therapies in vivo. We also evaluated the correlation of the PI-X expression in human CRC and T cell infiltration and the patients’ prognoses by the analyses of immunohistochemistry and TCGA RNA-seq data.

**Results**

10 out of 30 tested CD antigens were heterogeneously expressed on the human CRC cell lines. Among these 10 CD antigens, we found that the CD44-positive fraction in human CRC cell lines were more resistant to tumor specific CTL-mediated killing compared to the CD44-negative fraction. cDNA microarray analysis revealed the CD44- positive fractions more highly expressed protease inhibitor X (PI-X) than the CD44-negative fractions. The expression level of PI-X was also positively correlated with that of CD44 in TCGA RNA-seq database. PI-X showed the highest expression in CRC among 17 human cancer tissues in meta-analysis using open-access gene expression data. The experiments of PI-X overexpression or PI-X knockdown in CRC cell liens showed PI-X could suppressed CTL-mediated killing. TCGA RNA-seq data showed the negative correlation between PI-X expression and CD8 expression. Moreover, analyses of immunohistochemistry and TCGA RNA-seq data revealed the best prognosis of the patients with low PI-X expression and high CD8+ T cell infiltration. In tumor-bearing mouse models, over expression of PI-X in a murine cancer cell line induced the resistance to anti PD-1 Ab therapies.

**Conclusions**

CD44 and PI-X may be potential biomarkers for prognosis and responses of CRC patients to cancer therapies including PD-1 blockade, and also be attractive therapeutic targets for combination immunotherapies.

**Ethics Approval**

The study was approved by Keio Univ. school of medicine Institutution‘s Ethics Board, approval number 20P16

#### P561 A new mechanism of ADCC resistance

##### David Zahavi, MS, BS^1^, Dalal Aldeghaither^1^, Louis Weiner, MD^1^, Joseph Murray, MD, PhD^2^, Elana Fertig^2^, Garrett Graham^1^, Yong-Wei Zhang^1^, Allison O'Connell, MD/PhD Candidate^1^, Junfeng Ma^1^, Sandra Jablonski, PhD^1^

###### ^1^Georgetown University, Washington, DC, USA; ^2^Johns Hopkins University, Ellicott City, MD, USA

####### **Correspondence:** Louis Weiner (weinerl@georgetown.edu)

**Background**

Antibody-dependent cell-mediated cytotoxicity (ADCC) is an important mechanism underlying targeted monoclonal antibody (mAb) therapy in cancer. The majority of patients develop resistance to mAb therapy; however the resistance mechanisms are not well characterized. In vitro modeling of ADCC provides an experimental system for uncovering tumor cell immune resistance mechanisms.

**Methods**

We continuously exposed epidermal growth factor receptor (EGFR) positive A431 cells to KIR-deficient NK92-CD16V effector cells and the anti-EGFR mAb Cetuximab.

**Results**

Persistent ADCC exposure yielded ADCC-resistant cells, that when compared with control ADCC-sensitive cells, exhibited reduced EGFR expression, overexpression of histone- and interferon-related genes, failure to activate NK cells, and no evidence of epithelial to mesenchymal transition. These properties gradually reversed following withdrawal of ADCC selection pressure. Remarkably, ADCC-resistant cells possessed lower expression of multiple cell surface molecules that contribute to intercellular interactions and immune synapse formation. Classic immune checkpoints did not modulate ADCC in this unique model system of immune resistance.

**Conclusions**

We show that the induction of ADCC resistance involves genetic and epigenetic changes that lead to a general loss of target cell adhesion properties required for the establishment of an immune synapse, killer cell activation, and target cell cytotoxicity.

**Acknowledgements**

We would like to thank Dr. Kerry Campbell for providing the NK92-CD16V cells.

### Mechanisms of Toxicity

#### P562 Delayed immune-related events after discontinuation of immunotherapy – DIRE syndrome?

##### Marcus Couey, MD, DDS^1^, Bell, MD, DDS, FACS^1^, Ashish Patel, MD^2^, Marka Crittenden, MD, PhD^1^, Brendan Curti, MD^1^, Rom Leidner, MD^1^

###### ^1^Earle A. Chiles Research Institute, Portland, OR, USA; ^2^Providence Cancer Center, Portland, OR, USA

####### **Correspondence:** Marcus Couey (marcus.couey@providence.org)

**Background**

Although the temporality of immune-related adverse events (irAE) is well-recognized during immunotherapy to be highly variable and often delayed,[1] post-immunotherapy irAE are rarely described and potentially under- recognized. In 2013, two cases were reported in abstract form in Deutschen Dermatologischen Gesellschaft.[2] In July 2018 a case of autoimmune hepatitis eight months post-immunotherapy was reported in The Oncologist[3] and a dermatologic series appeared online in JAMA Dermatology.[4] With expanding indications for IO and an increasing number of clinical trials in the curative-neoadjuvant setting, larger numbers of patients are being treated in earlier stages of disease and often for short courses. Given this trend, under-recognition of delayed immune- related events (DIRE) after completion of immunotherapy could pose a growing clinical hazard.

**Methods**

We performed a literature review in PubMed and Google Scholar (search terms included in Table 1); DIRE syndrome was defined as immune-related events post-immunotherapy, newly incident beyond two elimination half- lives (t 1/2) of drug.

**Results**

We identified 10 cases, 6 by literature review (5 melanoma, 1 cutaneous SCC) and an additional 4 cases at our institution (4 HNSCC). Median cumulative immunotherapy exposure was 4 doses (range: 2 to 22 doses). Median interval from last immunotherapy dose to DIRE onset was 5 months (range: 2 to 28 months). All literature cases were in the recurrent/metastatic context; we report four cases in the curative-neoadjuvant context (italicized) with one recurrence.

**Conclusions**

An influx of neoadjuvant clinical trial design over the last 2-3 years, incorporating brief IO exposure (typically checkpoint blockade) followed by surgical resection and/or adjuvant therapy, is attracting interest in multiple tumor types in the curative setting.[5–9] In this context, it will be necessary to recognize an emerging phenomenon, which we have termed DIRE syndrome (delayed immune-related events). Clinical vigilance has the potential to reduce morbidity from delayed diagnosis, as these conditions are generally manageable with prompt initiation of treatment; or from misdiagnosis, to avert unnecessary/harmful interventions (in the autoimmune meningitis case we report, an Omaya reservoir was placed at an out-of-state hospital based on erroneous diagnosis of leptomeningeal carcinomatosis). Several factors confound diagnosis in the neoadjuvant-IO context: 1) intervening treatments with potentially overlapping toxicities; 2) brief and remote IO exposure; 3) reduced vigilance during NED surveillance, in contrast to active disease follow-up; 4) protracted process of diagnosis-by-exclusion. DIRE syndrome should therefore figure prominently in the differential diagnosis of patients presenting with diseases of unclear etiology, irrespective of elapsed post-immunotherapy interval.

**References**

1. Champiat S, et al. Management of immune checkpoint blockade dysimmune toxicities: a collaborative position paper. Ann. Oncol. Off. J. Eur. Soc. Med. Oncol. 2016;27:559–574.
2. Abstracts of the 8th World Congress of Melanoma, the 9th Congress of the European Association of Dermatology (EADO), the 7th Interdisciplinary Melanoma/Skin Cancer Meeting, and the 3rd European Post-Chicago Melanoma Meeting. July 17-20, 2013. Hamburg, Germany. J. Dtsch. Dermatol. Ges. J. Ger. Soc. Dermatol. 2013;JDDG 11 Suppl 7:1–119.
3. Parakh S, Cebon J, Klein O. Delayed autoimmune toxicity occurring several months after cessation of anti-PD-1 therapy. The Oncologist. 2018;23:849–851.
4. Wang L, et al. Timing of onset of adverse cutaneous reactions associated with programmed cell death protein 1 inhibitor therapy. JAMA Dermatol. 2018; doi:10.1001/jamadermatol.2018.1912
5. Forde P, et al. Neoadjuvant PD-1 blockade in resectable lung cancer. N. Engl. J. Med. 2018;378:1976–1986.
6. Uppaluri R, et al. Neoadjuvant pembrolizumab in surgically resectable, locally advanced HPV negative head and neck squamous cell carcinoma (HNSCC). J. Clin. Oncol. 2017;35:6012–6012.
7. Ferris R, et al. LBA46An open-label, multicohort, phase 1/2 study in patients with virus-associated cancers (CheckMate 358): Safety and efficacy of neoadjuvant nivolumab in squamous cell carcinoma of the head and neck (SCCHN). Ann. Oncol. 2017;28.
8. Necchi, A. et al. Interim results from PURE-01: A phase 2, open-label study of neoadjuvant pembrolizumab (pembro) before radical cystectomy for muscle-invasive urothelial bladder carcinoma (MIUC). J. Clin. Oncol. 2018;36:TPS533-TPS533.
9. Powles, T. et al. A phase II study investigating the safety and efficacy of neoadjuvant atezolizumab in muscle invasive bladder cancer (ABACUS). J. Clin. Oncol. 2018;36: 4506–4506.

**Table 1 (abstract P562).**
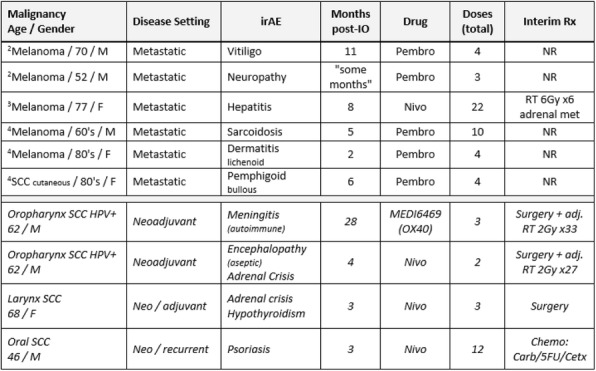
See text for description

#### P563 Characterizing immune-mediated adverse events of anti-PD-1 and anti-CTLA-4 monotherapies and combinations using a quantitative model based meta-analysis

##### Gabriel Helmlinger, PhD^1^, Boris Shulgin, PhD^2^, Yuri Kosinsky, PhD^2^, Andrey Omelchenko, PhD^2^, Lulu Chu, PhD^1^, Ganesh Mugundu, PhD^1^, Garrett DeYulia, PhD^1^, Rodrigo Pimentel, MD^1^, Kirill Peskov, PhD^2^

###### ^1^AstraZeneca, Waltham, MA, USA; ^2^M&S-Decisions LLC, Moscow, Russian Federation

####### **Correspondence:** Gabriel Helmlinger (gabriel.helmlinger@astrazeneca.com)

**Background**

Immune checkpoint inhibitors (ICIs) may be associated with treatment-mediated and immune-mediated adverse events (trAEs and imAEs, respectively), in particular when used in combination. The objective of this model-based meta-analysis (MBMA was to characterize immune mediated AEs of anti-PD-1 and anti-CTLA-4 monotherapies and combinations, normalized by drug exposure and drug potency.

**Methods**

We performed an exhaustive search of clinical ICI safety data in PubMed-Medline, the ASCO Abstracts, TrialTrove, and other sources. To quantitatively compare safety across monotherapy and combination studies, we normalized the data by drug exposure, as derived from pharmacokinetic models for anti-PD-1 and anti-CTLA-4 agents, and by drug potency. A cross-study model-based meta-analysis (MBMA) of safety was then performed for Grade 3&4 trAEs and imAEs, for five organ classes: gastrointestinal (GI), skin, pulmonary, hepatic, and endocrine. Exposure vs. safety meta-regression and subgroup analyses were implemented with the R software metafor package.

**Results**

Safety data from 133 cohorts out of 82 publications on 65 clinical trials were built into the study-level database for ICI trAEs and imAEs. Performing the corresponding MBMA, we determined: (a) no significant dependence on dose, for both Grade 3&4 total AEs and imAEs, under anti-PD-1 monotherapies, except for rare GI imAEs; (b) significant dose dependence, for both Grade 3&4 total AEs (increase from 23% [low] to 40% [high dose]) and hepatic AEs (increase 1% to 7%), under anti-CTLA-4 monotherapies, (c) significant anti-CTLA-4 dose dependence, for both Grade 3&4 total AEs (increase from 35% to 52%) and hepatic (increase 6.5% to 19%), GI, skin and endocrine AEs, under anti-CTLA-4 and anti-PD-1 combination treatments. AE rates for anti-PD-1 and anti-CTLA-4 combinations were supra additive vs. AE rates in the respective monotherapy, with an increase of ~10% for Grade 3&4 AES and ~15% for Hepatic AEs. Furthermore, under combination treatments, AE rates were from 5% (monotherapy) up to 20% (combination) higher in 1L patients (vs. other lines of treatment) and also about 15% higher in patients with positive PD-L1 status (vs. PD-L1 negative). Higher AE rates, generally, were also associated with higher efficacy responses to ICI therapies (Figure 1).

**Conclusions**

A comprehensive database combined with an exposure/potency-normalized MBMA of ICI-related imAEs enabled a quantitative comparison of AEs across anti-PD-1 / CTLA-4 mono- and combination therapies, and in relation to key patient characteristics (PD-L1 status, line of treatment) and efficacy measures. This analysis may support rational dose selection and can be applied to other ICI agents, in mono- and combination treatment settings.

**Fig. 1 (abstract P563).**
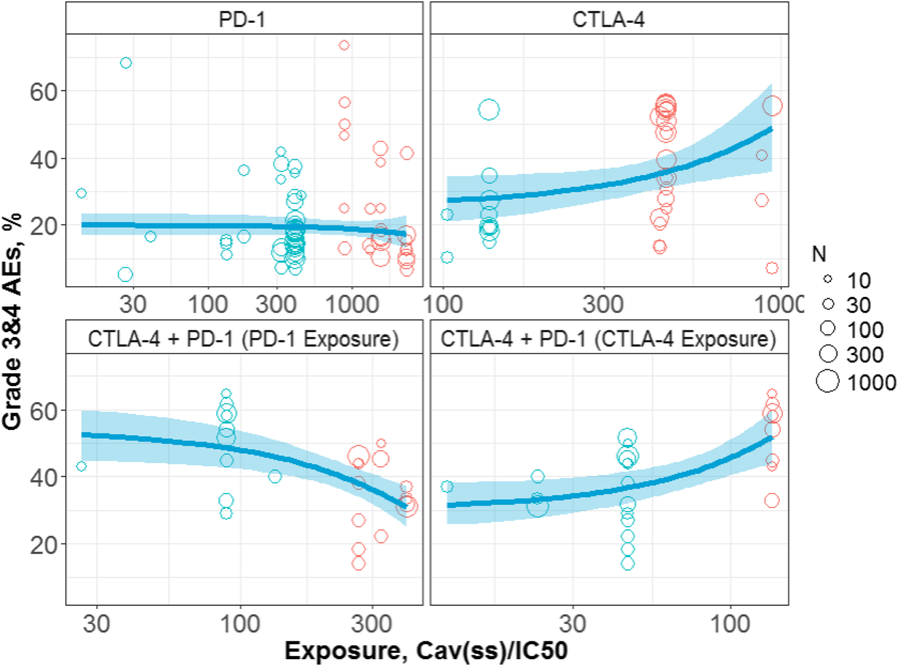
Dependence of Grade 3&4 AEs upon ICI drug exposure

#### P564 Interleukin-6 gene expression is highly upregulated in immune checkpoint mediated enterocolitis

##### Daniel Johnson, MD^1^, Cara Haymaker, PhD^1^, Khalida Wani, PhD^1^, Wai Chin Foo, MD^1^, Salah Eddine Bentebibel^1^, Yinghong Wang, MD, PhD^1^, Jonathan Curry, MD^1^, Adi Diab, MD^1^, Jennifer Wargo, MD, MMSc^2^, Alexandre Reuben^2^, Elizabeth Burton^2^

###### ^1^MD Anderson Cancer Center, Houston, TX, USA; ^2^MD Anderson, Houston, TX, USA

####### **Correspondence:** Adi Diab (adiab@mdanderson.org)

**Background**

A deep understanding of the immunobiology of checkpoint inhibitor (CPI) induced immune related toxicities (irAEs) could lead to development of strategies that uncouple autoimmunity from anti-tumor immunity. Immune- related enterocolitis (irEC) is the most common serious complication from CPIs. Interleukin-6 (IL-6) is a key cytokine in autoimmunity (rheumatoid-arthritis, inflammatory-bowel disease) contributing to acute and chronic inflammation and is an essential differentiating cytokine committing naïve CD4+T-cells into T-helper17 (Th17) lineage. The role of Th17 cells in irAEs is not fully explored, and their tumor immunity function is controversial. Through RNA gene-expression profiling, we sought to identify the critical immune pathways in irEC and how these compare to the immune signatures in CPI-responding tumor samples.

**Methods**

Total RNA from patient-matched irEC and normal colon FFPE tissue from patients [n=12] receiving CPIs (aPD-1 = 3, aCTLA-4= 7, aCTLA-4 + aPD-1 = 2) were profiled with the NanoString nCounter PanCancer Immune Profiling Panel (NanoPCIP). Of the 770 NanoPCIP-panel genes, fold change in gene expression were compared between the normal and inflamed colonic tissue using two-sample T tests. P-values were corrected using Benjamin-Yekutieli adjusted false discovery rate, and an adjusted p-value < 0.05 were considered significant. We also summarized fold- changes in gene-expression in CPI-responding melanoma tumors from a longitudinal NanoPCIP panel immune signature analysis previously performed at our institution.

**Results**

Significantly upregulated differentially expressed genes (DEGs) in the inflamed irEC tissue was observed in 52 genes compared to the normal colon control (adjusted p<0.05; figure 1). The highest up-regulated DEG encoded for IL6 (Fold change of +24.1). Other genes highly upregulated included IL-11 (a member of the IL-6-type cytokine- family) and genes that encode chemotactic molecules (Table-1).In our melanoma historical control, 173 DEGs significantly upregulated with a-CTLA-4 treatment (pre vs. on-treatment tumors) had a significant interaction with treatment-response. Only one gene was concordantly in our highest up-regulated DEGs in colitis (CXCL2). Most of our 10 highest colitis DEGs were not significantly up-regulated in tumors of CPI-responders compared to non- responders (Table-1). None of the 10 genes significantly and highest up-regulated in responding tumors were significantly upregulated in irEC (Table-2).

**Conclusions**

IL6 was the most significantly upregulated gene within inflamed-irEC samples compared to matched controls; the majority were not upregulated in association with tumor-response to CPIs. Our data suggest that IL-6 is important in irEC. IL-6-mediated inflammation may be more prevalent in irEC than in the responding tumors; targeting IL-6 may ameliorate irEC without hindering anti-tumor immunity.

**Table 1 (abstract P564).**
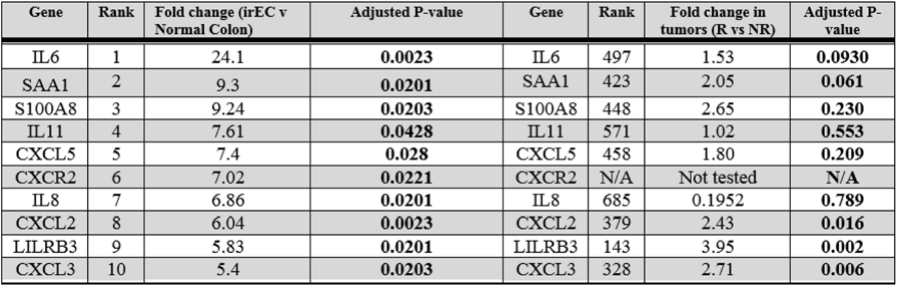
See text for description

**Table 2 (abstract P564).**
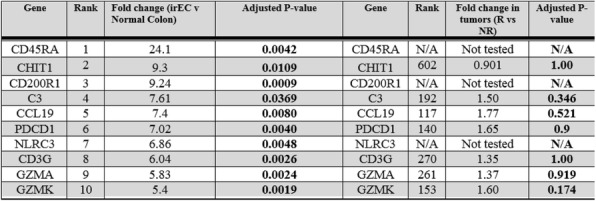
See text for description

**Fig. 1 (abstract P564).**
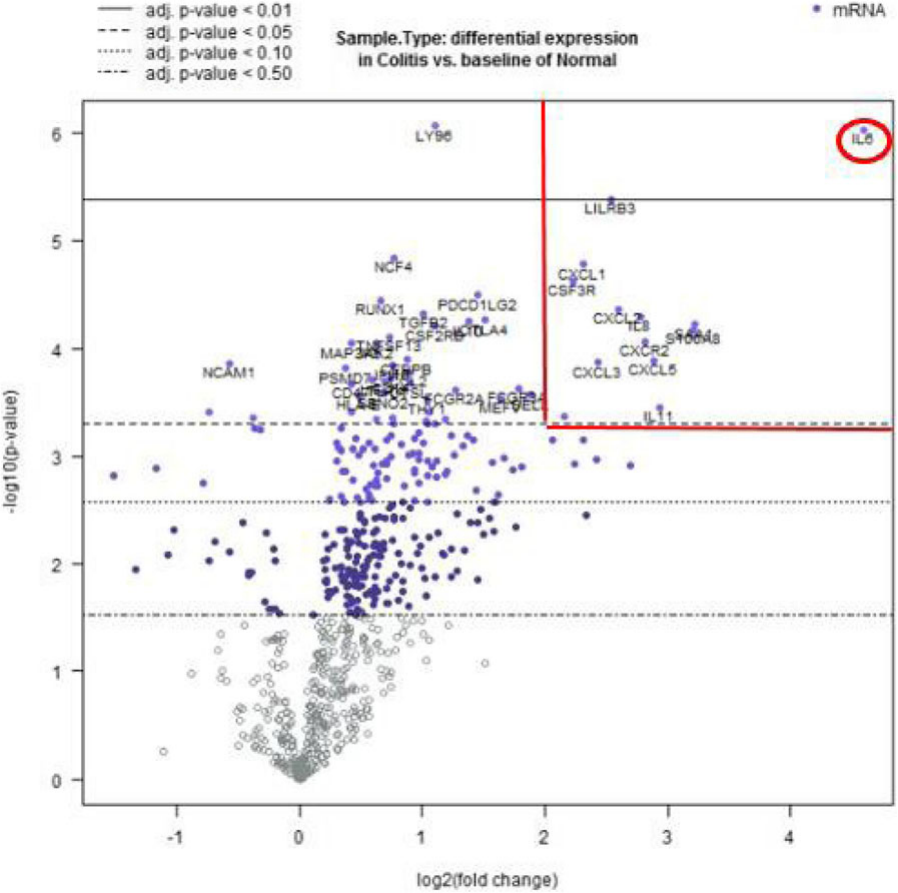
See text for description

#### P565 Characterization of lymphoid cells in synovial fluid from cancer patients with immunotherapy-associated arthritis

##### Sang Kim, MD, PhD, Roza Nurieva, PhD, Jean Tayar, MD, Huifang Lu, MD, PhD, Jennifer Wang, MD, Don Gibbons, MD, Guillermo Garcia-Manero, MD, Maria Suarez-Almazor, MD, PhD, Patrick Hwu, MD, Adi Diab, _MD_

###### MD Anderson Cancer Center, Houston, TX, USA

####### **Correspondence:** Adi Diab (adiab@mdanderson.org)

**Background**

Immune related arthritis (ir-arthritis) is well documented Checkpoint inhibitors (ICIs) toxicity [1]. Ir AR occur in ~2% of cancer patients who receive ICI [2,3,4]. Although it is not life-threatening toxicity, it is clinically symptomatic toxicity and can severely impact the patients’ quality of life and can lead to disconsolation of ICI treatment. Usually, ir-arthritis requires a substantially prolonged period of immune suppression compared to other irAEs, which we may negatively impact and compromise the clinical anti-cancer benefit. A deeper understanding of the immunobiology of ir-arthritis and detailed immune-characterization of the inflamed tissues will possibly allow us to develop treatment strategies that lead to uncoupling autoimmunity from anti-tumor immunity. Here, we characterize synovial immune cells isolated from five patients who developed ir-arthritis post-ICI.

**Methods**

We analyzed synovial fluid from five symptomatic patients, who developed ir-arthritis (joint pain and swelling) after ICI treatment. Using flow cytometry, we stained lymphoid immune cells with lineage-specific markers and measured effector cytokines in the CD4+ T cell populations.

**Results**

Median time of joint aspiration from the first ICI-infusion was 34 weeks (Range [4,166]). Arthritis was initially treated with systemic or local injection of prednisone. Two patients achieved complete resolution of arthritis (“steroid responders”) while three patients remained refractory with partial relief/response to prednisone requiring additional/alternative treatment (“steroid refractory”). Immune analysis demonstrated that CD4+ T cells (53.1±13.2%) most abundant lymphoid immune-cells followed by CD8+ T cells, NK cells, NK T cells, and B cells. Most CD4+ and CD8+ T cells were effector memory phenotype. Several relevant CD4+ T-cell subsets were identified in the synovial fluid, including regulatory T cells (Treg), naïve T cells, Th1 (CXCR3hi CCR6lo), Th17.1 (CXCR3hi CCR6hi), Th17 (CXCR3lo CCR6hi), and follicular helper T cells (CXCR5+). Effector CD4+ T cell cytokines, including interferon gamma, IL-4, IL-17, and IL-21, were produced by both Tregs and non-Tregs. Interestingly, IL-17 producing non-Tregs were expanded in synovial fluid from steroid –refractory patients compared to synovial fluid from steroid-responders, suggesting that Th17 cells might play a role in persistent ir- arthritis and steroid resistance.

**Conclusions**

Although our preliminary data is very limited and descriptive in nature, we find the observed expansion of IL-17 producing non-Tregs in synovial fluid from steroid- refractory very intriguing and consistent with our prior recent publication of a successfully treated patients with steroid resistant ir-arthritis with IL-6 blockade [5]. Larger and prospective studies with longitudinal collection of tissue and blood are planned.

**References**

1. Suarez-Almazor ME, Kim ST, Abdel-Wahab N, et al. Review: Immune-Related Adverse Events With Use of Checkpoint Inhibitors for Immunotherapy of Cancer. Arthritis Rheumatol 2017;69(4):687-99.
2. Cappelli LC, Gutierrez AK, Baer AN, et al. Inflammatory arthritis and sicca syndrome induced by nivolumab and ipilimumab. Annals of the rheumatic diseases 2017;76(1):43-50.
3. Robert C, Schachter J, Long GV, et al. Pembrolizumab versus Ipilimumab in Advanced Melanoma. The New England journal of medicine 2015;372(26):2521-32.
4. Prieto PA, Yang JC, Sherry RM, et al. CTLA-4 blockade with ipilimumab: long-term follow-up of 177 patients with metastatic melanoma. Clin Cancer Res 2012;18(7):2039-47.
5. Kim ST, Tayar J, Trinh VA, et al. Successful treatment of arthritis induced by checkpoint inhibitors with tocilizumab: a case series. Annals of the rheumatic diseases 2017;76(12):2061-64.

**Ethics Approval**

The study was approved by MD Anderson Cancer Center's ethic board (Protocol number: PA16-0935)

#### P566 Hypoalbuminemia as a predictor factor for immune related adverse events (irAEs) in advanced melanoma patients treated with immune checkpoint inhibitors (ICIs)

##### E Rahma, MD^2^, Steven Blum, MD^2^, Jeffrey Ishizuka, MD,DPhil^2^, Taha Qazi, MD^3^, Rawad Elias, MD^4^, Kruti Vora, BA^5^, Alex Ruan^5^, Anita Giobbie-Hurder, MS^2^, Shilpa Grover, MD, MPH^3^, Rizwan Haq, MD, PhD^2^, Meredith Davis^2^, Maria Gargano, PA-C^2^, Elizabeth Buchbinder, MD^2^, Patrick Ott, MD, PhD^2^, F. Stephen Hodi, MD^2^

###### ^1^Dana-Farber Cancer Institute, Boston, MA, USA; ^2^Dana Farber Cancer Institute, Boston, MA, USA; ^3^Brigham and Women Hospital, Boston, MA, USA; ^4^Boston Medical Center, Hartford, CT, USA; ^5^Harvard Medical School, Boston, MA, USA

####### **Correspondence:** E Rahma (osamae_rahma@dfci.harvard.edu)

**Background**

Despite the expansion of Immune checkpoint inhibitors (ICIs) indications there is limited data to date on predictors of ICIs’ immune related adverse events (irAEs).

**Methods**

Melanoma patients (pts) who received anti-CTLA-4 (ipilimumab), anti-PD-1 (pembrolizumab or nivolumab), or the combination at the Center for Immuno-Oncology and Melanoma at DFCI in the past 5 years (up to December 31- 2016) were included. Patients with irAEs were divided into 2 groups based on the severity of AEs: Grade (G) 1-2 and ≥ G 3. We analyzed the following patient characteristics and their prediction for irAE grade: Gender, age, BMI, ECOG, smoking and alcohol history, Flu or pneumonia vaccine administered within 6 months of starting ICIs, infections while on ICIs, history of autoimmune disease, asthma, and seasonal allergies. The following lab values were collected prior to starting ICIs: Albumin, LDH, neutrophil/lymphocyte ratio, and eosinophil count. We also analyzed the following: Prior chemo, targeted or radiation therapy, the presence of Kit, BRAF, or NRAS mutation, the number of metastatic sites, and concomitant medications (ACE and ARB inhibitors, NSAID, PPI, statins, antibiotics and vitamin D). Multivariable logistic regression of grade 3-4 vs. grade 1-2 irAEs was fit using the preceding characteristics as candidate predictors.

**Results**

We identified 213 patients who received a total of 246 ICIs (44 pts had 2 and 5 pts had 3 ICIs). The maximum grade irAEs reported were: G1 or 2 (92 pts, 43%), G 3 or 4 (121 pts, 57%). Table 1 summarizes the type of ICI the patient was taking at the time of the worst grade irAE. Patients who received combination of ICIs had significantly increased risk of grade 3-4 irAEs compared with patients who received single ICI. Patients with albumin levels above 4.2 had significantly reduced risks of G 3-4 irAEs compared with patients who had lower albumin level (table 2).

**Conclusions**

This is the first report to identify hypoalbuminemia as a predicting factor for the development of grade 3-4 irAEs while on ICIs. Hypoalbuminemia could represent poor nutritional status that may predispose patients to irAEs. We are in the process of performing correlative analyses using cytokine Luminex to identify inflammatory markers that could predict toxicity, and this will be correlated with the observation of an association between hypoalbuminemia and higher incidence of grade 3-4 irAE.

**Acknowledgements**

Parker Institute for Cancer Immunotherapy for providing funding for this project

**Ethics Approval**

The study was approved by Dana-Farber Institutional Review Board (IRB)

**Table 1 (abstract P566).**
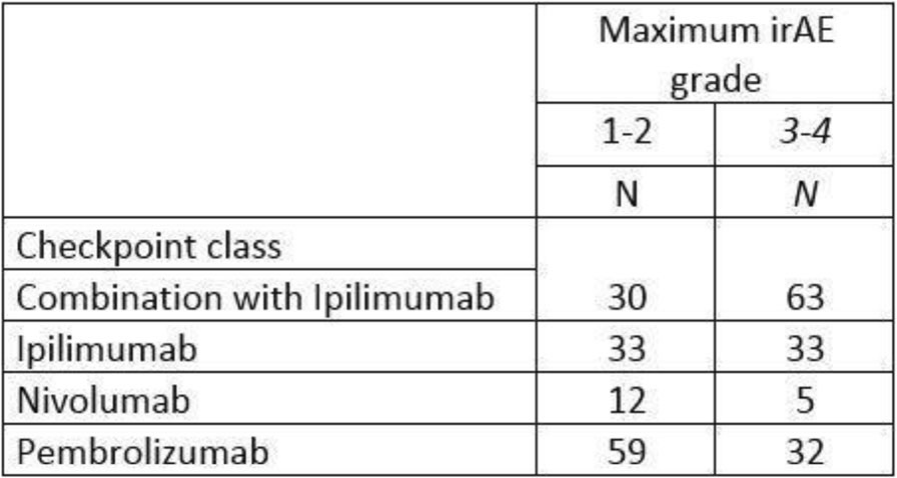
Type of ICI received at time of worst irAEs grade

**Table 2 (abstract P566).**
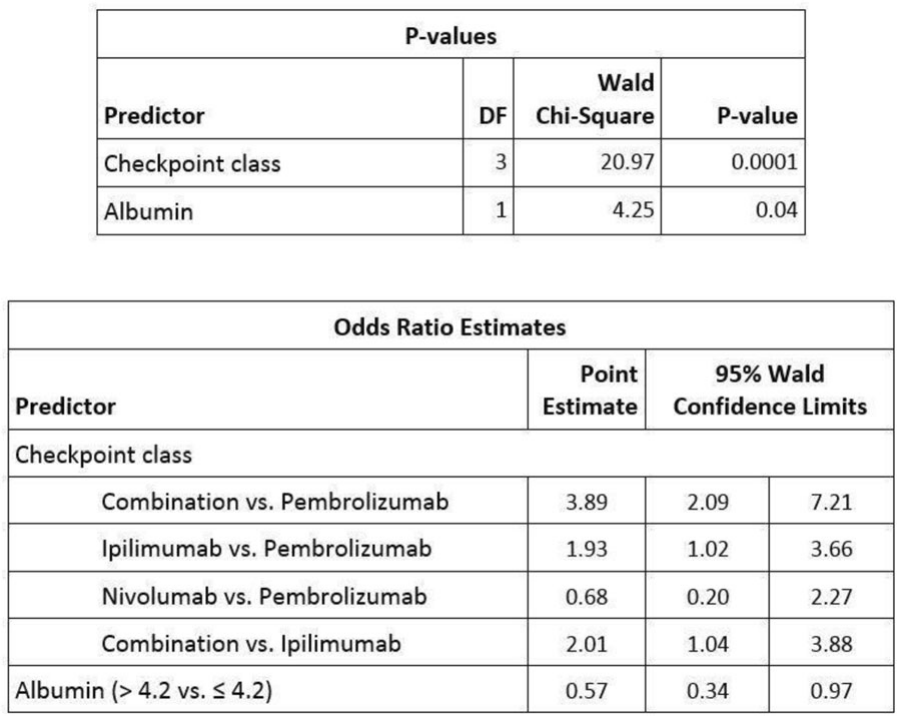
Prediction Model for irAEs

#### P567 A meta-analysis of immune checkpoint inhibitors tumor type and dose-toxicity correlation

##### E Rahma, MD^1^, E Rahma, MD^1^, E Rahma, MD^1^, E Rahma, MD^1^, Joshua Reuss, MD^2^, Ghazaleh Shoja E Razavi, MD^3^, Rawad Elias, MD^4^, Anita Giobbie-Hurder, MS^1^, Samir Khleif, MD^5^

###### ^1^Dana-Farber Cancer Institute, Boston, MA, USA; ^2^Sidney Kimmel Cancer Center, Baltimore, MD, USA; ^3^Tom Baker Cancer Centre, Alberta, Canada; ^4^Boston Medical Center, Hartford, CT, USA; ^5^Georgetown University, Washington, DC, USA

####### **Correspondence:** Samir Khleif (snk48@georgetown.edu)

**Background**

The relationship between ICIs dose-escalation and toxicity has not been established. We performed a meta-analysis of clinical trials investigating ICIs to understand whether there is a correlation between dose or disease type and toxicity.

**Methods**

We searched PubMed and abstracts presented at national and international meetings for trials (T) using FDA-approved ICIs including Ipilimumab, Atezolizumab, Nivolumab, and Pembrolizumab. The rates of treatment-related grade 3-5 adverse events (G3/4 AEs) were collected and the overall incidence rates for each dose cohort (DC) were estimated using exact binomial methods. Generalized linear models with GEE were fit to assess important predictors of G3/4 AEs.

**Results**

A total of 52 T published between January 2010 and December 2017 were reviewed. The overall incidence rate of G3/4 AEs was 34% in melanoma T using Ipilimumab. Patients (Pts) treated at 3 mg/kg q3w (3 T) had 27% reduced risk of G3/4 AEs compared to 10 mg/kg q3w (3 T) (Figure 1, Table 1). There was no difference in the incidence of G3/4 AEs for urothelial cancer (2 T) vs. NSCLC (3 T) using Atezolizumab (1200mg q3w) (Figure 2, Table 2). The investigation of Nivolumab included 39 DC within 24 different T. We compared the following DC: 2mg/kg q3w (2 DC), 3 mg/kg q2w (20), ≤ 1mg/kg q2w (8), ≤ 1mg/kg q3w (2), 10 mg/kg q2w (4), 10 mg/kg q3w (3). The overall incidence rate of G3/4 AEs was 22% which was significantly lower for pts with NSCLC than any of the other tumor types (26-40% reductions). No relationships between dose and incidence of AEs were noted (Figure 3, Table 3). The Pembrolizumab analysis consisted of 23 DC from 17 reported T. Frequencies of DC: 2 mg/kg q3w (3), 200 mg q3w (8), 10 mg/kg q3w (5), 10 mg/kg q2w (7). The incidence of G3/4 AEs was significantly lower in melanoma compared to any of the other tumor types (20% risk reduction). Pts receiving flat dose of 200mg had significantly lower odds of G3/4 AEs compared to 2 mg/kg q3 (P= 0.04) or 10 mg/kg q2 or 3w (P= 0.01) (Figure 4, Table 4).

**Conclusions**

This is the largest meta-analysis to date investigating dose-toxicity relationship of ICIs. There is a clear correlation between increased dose and toxicity using CTLA-4 antibodies (Ipilimumab). However, there is no evidence of dose-toxicity correlation using Nivolumab, while a flat dose of Pembrolizumab was associated with lower toxicity compared to weight-based dose.

**Fig. 1 (abstract P567).**
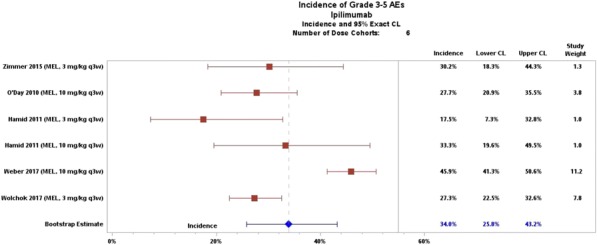
Incidence of Grade 3-5 Adverse Events Ipilimumab

**Fig. 2 (abstract P567).**
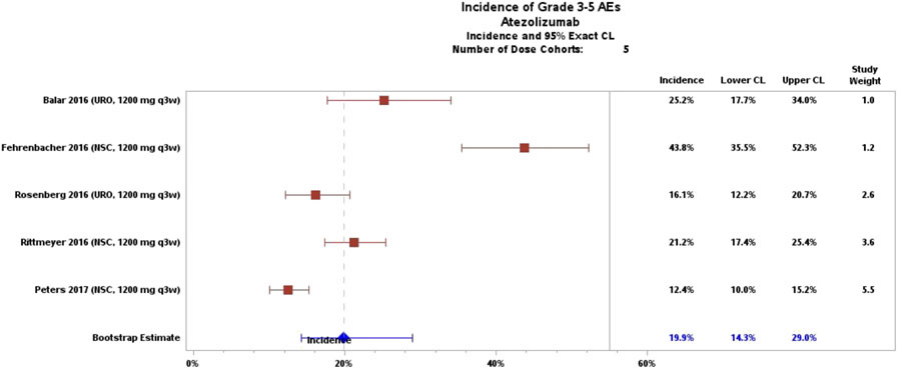
Incidence of Grade 3-5 Adverse Events Atezolizumab

**Fig. 3 (abstract P567).**
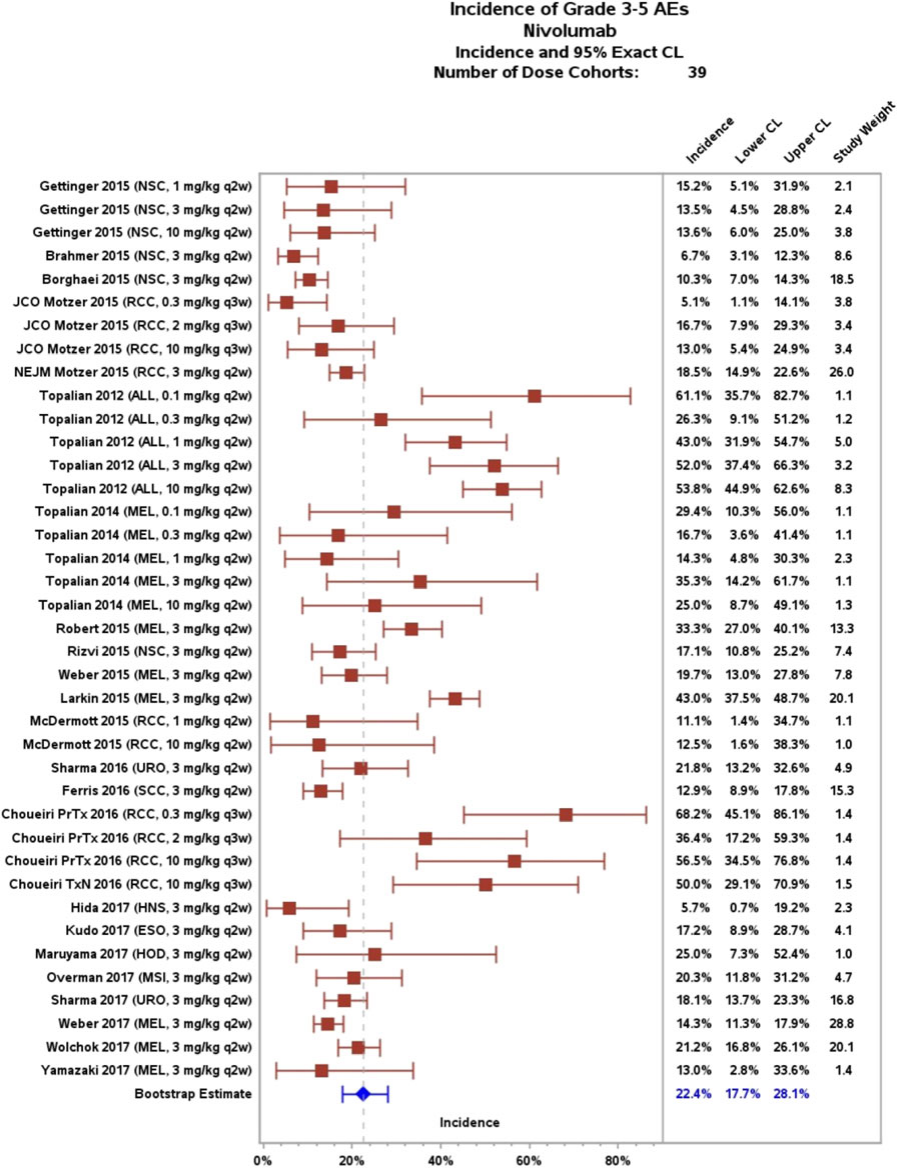
Incidence of Grade 3-5 Adverse Events Nivolumab

**Fig. 4 (abstract P567).**
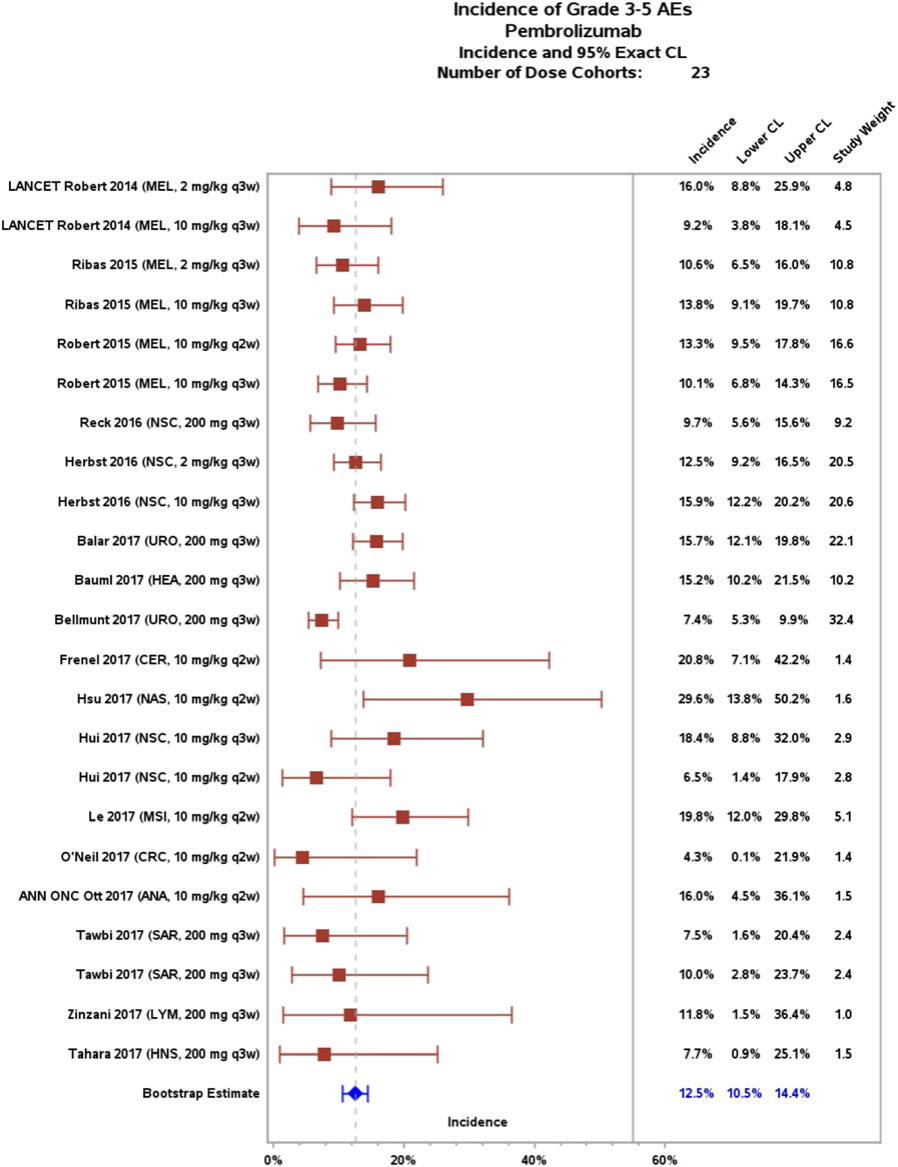
Incidence of Grade 3-5 Adverse Events Pembrolizumab

**Table 1 (abstract P567).**
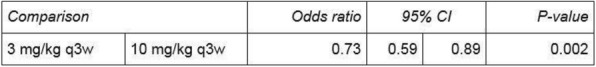
Dose-Toxicity Correlation of Ipilimumab

**Table 2 (abstract P567).**
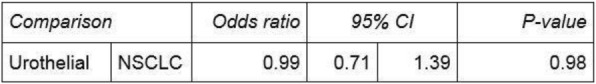
Tumor Type-Toxicity Correlation of Atezolizumab

**Table 3A (abstract P567).**
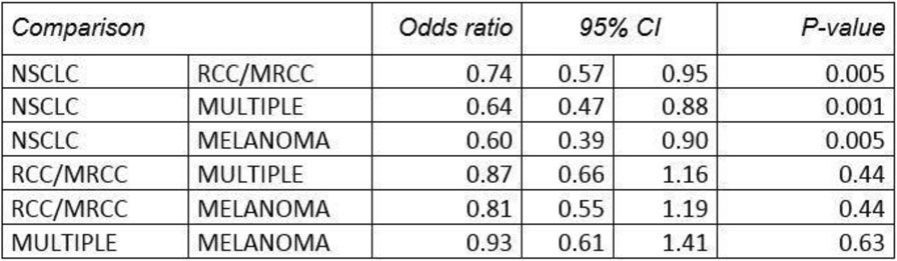
Tumor Type-Toxicity Correlation of Nivolumab

**Table 3B (abstract P567).**
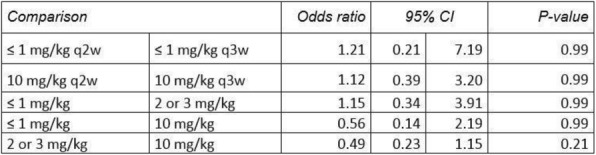
Dose-Toxicity Correlation of Nivolumab

**Table 4A (abstract P567).**
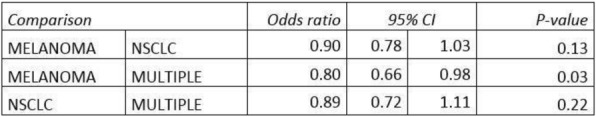
Tumor Type-Toxicity Correlation of Pembrolizumab

**Table 4B (abstract P567).**
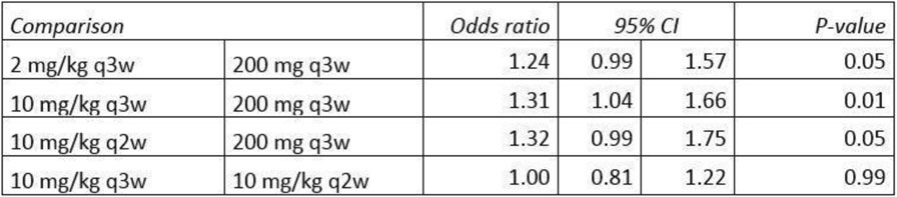
Dose-Toxicity Correlation of Pembrolizumab

#### P568 Pembrolizumab induced multiple immune related adverse events including myasthenia gravis, hepatitis and thyroiditis in a patient with thymoma

##### Kyunghoon Rhee, MD^1^, Taeyeong ko, MD^1^, Sangmin Chang, MD^1^, Ji Hyun Rhee, MD^2^, Lee Chun Park, MD^1^, Young Kwang Chae, MD^1^

###### ^1^Northwestern Univ, Feinberg School Med, Towson, MD, USA; ^2^Greater Baltimore Medical Center, Towson, MD, USA

####### **Correspondence:** Young Kwang Chae (young.chae@northwestern.edu)

**Background**

The rapidly developing field of cancer immunotherapy has shown remarkable progress in its utilization as a treatment option of various tumors in recent years. Although it is currently not recommended as a first line treatment for thymoma, pembrolizumab, which blocks the PD-1 pathway thereby enhancing the immune system, is used as an alternative option in treatment. However, it was shown to also induce rare immune-related adverse events (irAEs) in multiple organs [1, 2], which have been reported in limited cases.

**Methods**

Here we report a case of possible pembrolizumab induced myasthenia gravis (MG), hepatitis and thyroiditis.

**Results**

A 60-year-old female with metastatic thymoma on her second cycle of pembrolizumab presented with worsening SOB for two weeks, left ptosis, limited extra-ocular movement, lower bifacial, upper and lower extremity weakness. She was thought to have either pembrolizumab induced MG or unmasking of occult thymoma related MG, supported by elevated acetylcholine receptor binding antibodies. She was treated with pyridostigmine, IVIG, and plasmapheresis. On labs, TSH was found to be increased and free T4 decreased. Considering her normal thyroid functions before immunotherapy and the rapid development of hyperthyroidism within 2 weeks after the second cycle, pembrolizumab induced thyroiditis was suspected.In addition, she had gradually increasing Alk-P, AST, ALT and total bilirubin. Liver biopsy demonstrated marked portal and lobular T-cell infiltration with bile duct injury, consistent with immune modulator drug effect. She was treated with steroids and Cellcept with improvement in her LFTs. However, she developed septic shock and died.

**Conclusions**

This is a patient with stage IV thymoma on pembrolizumab who developed multiple irAEs. It is important to have a high clinical suspicion of such irAEs, and not only to discontinue the culprit PD-1 inhibitor but also to start early treatment for each involved organ. Since pembrolizumab is not a standard treatment of stage IV thymoma, there are only few reports of irAEs in thymoma. We do not know if pembrolizumab induced a new onset MG or exacerbated underlying MG. It is also unclear if simultaneous development of MG, hepatitis and thyroiditis is only unique in thymoma. Further investigation of irAEs in thymoma patients on pembrolizumab is therefore warranted.

**References**

1. Hofmann L et al. Cutaneous, gastrointestinal, hepatic, endocrine, and renal side-effects of anti-PD-1 therapy. Eur J Cancer. 2016 Jun;60:190-209
2. Zimmer L et al. Neurological, respiratory, musculoskeletal, cardiac and ocular side-effects of anti-PD-1 therapy. Eur J Cancer. 2016 Jun;60:210-25.

**Consent**

Consent top publish was received.

### Microbiome and Anti-Tumor Immunity or I-O Agent Toxicity

#### P569 Novel Pharmacobiotic approach to enhance the tamoxifen efficacy using bacterial extracellular vesicles as the immunotherapy in breast cancer

##### Jeongshin An, MD,PhD^1^, Yeun-yeoul Yang^1^, Won-Hee Lee^2^, Jinho Yang^2^, Jong-kyu Kim^1^, HyunGoo Kim^1^, Se Hyun Paek^1^, Jun Woo Lee^1^, Joohyun Woo^1^, Jong Bin Kim^1^, Hyungju Kwon^1^, Woosung Lim^1^, Nam Sun Paik^1^, Yoon-Keun Kim^2^

###### ^1^Ewha Womans University, Seoul, Korea, Republic of; ^2^MD healthcare company, Seoul, Korea, Republic of

####### **Correspondence:** Jeongshin An (rulru81@hanmail.net)

**Background**

The anti-cancer effect of bacteria has a long history. According to Bierman et al., spontaneous remission of cancer has been observed in patients with severe bacteremia[1]. The reason was not revealed at that time, but we studied that in breast cancer. There are four main ways in which microbiota affects cancer: probiotics, prebiotics, drugs that target microbial enzymes and microbial products that have anticancer properties[2]. Among them, bacterial extracellular vesicles(EVs) are one of microbial products. In this study, we investigated the effects of bacterial EVs on the growth of breast cancer cells and tamoxifen efficacy.

**Methods**

Here, we analized microbiota of urine samples by NGS to select the target EVs that were expected to affect the growth of breast cancer cells. A total of 347 female urine samples – from 127 breast cancer patients (cancer group) and 220 normal individuals (control group) – were collected and analyzed by NGS using a universal bacterial primer of 16S rDNA. Human breast cancer cells were cultured, and the cells were treated with EVs of S. aureus and K.pneumoniae for 72 h. Real-time polymerase chain reaction (PCR) and Western blotting for signalling molecule analysis were performed after treatment of EVs in each breast cancer cell.

**Results**

There was a significant difference in the distribution of bacterial EVs between the urine samples from breast cancer patients and from normal controls. Especially, S.aureus EVs were predominant in the normal group, and K.pneumoniae was abundant in the breast cancer group. Therefore, we selected these two bacterial EVs that may have an effect on breast cancer cell growth. We found that S.aureus and K.pneumoniae EVs down-regulated cell growth in MDA-MB-231 cells. We also found that S.aureus or K.pneumoniae EVs had a synergic effect on growth inhibition of while co-treated with tamoxifen. S.aureus EVs down-regulated mRNA expression of cyclin E2 and up- regulated that of TNF-alpha which was related ERK pathway while co-treated with tamoxifen.

**Conclusions**

The anti-cancer effect of S.aureus and K.pneumoniae was initiated by its bacterial EVs and consequently inhibited the growth of breast cancer cells in triple negative breast cancer cells and improved the efficacy of tamoxifen in ER-positive cells. In the near future, we plan to conduct animal studies which are expected to further clarify the effect of bacterial EV on breast cancer.

**References**

1 Bierman, H. R. et al. Remissions in leukemia of childhood following acute infectious disease. Staphylococcus and streptococcus, varicella, and feline panleukopenias. Cancer 6, 591-605 (1953).2 Zitvogel, L., Daillère, R., Roberti, M. P., Routy, B. & Kroemer, G. Anticancer effects of the microbiome and its products. Nature Reviews Microbiology 15, 465 (2017).

**Ethics Approval**

The study was approved by Ewha Womans University Medical Center‘s Ethics Board.

**Consent**

Written informed consent was obtained from the patient for publication of this abstract and any accompanying images. A copy of the written consent is available for review by the Editor of this journal.

**Fig. 1 (abstract P569).**
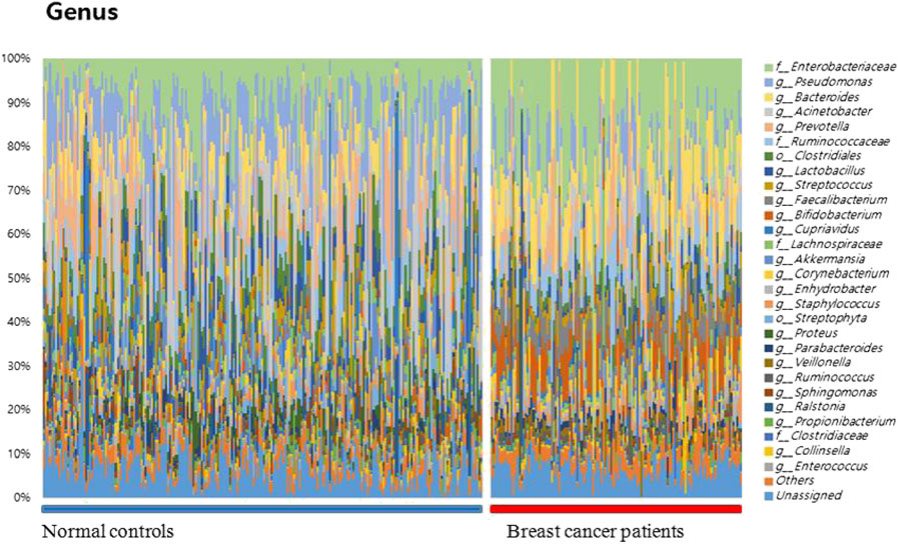
See text for description

**Fig. 2 (abstract P569).**
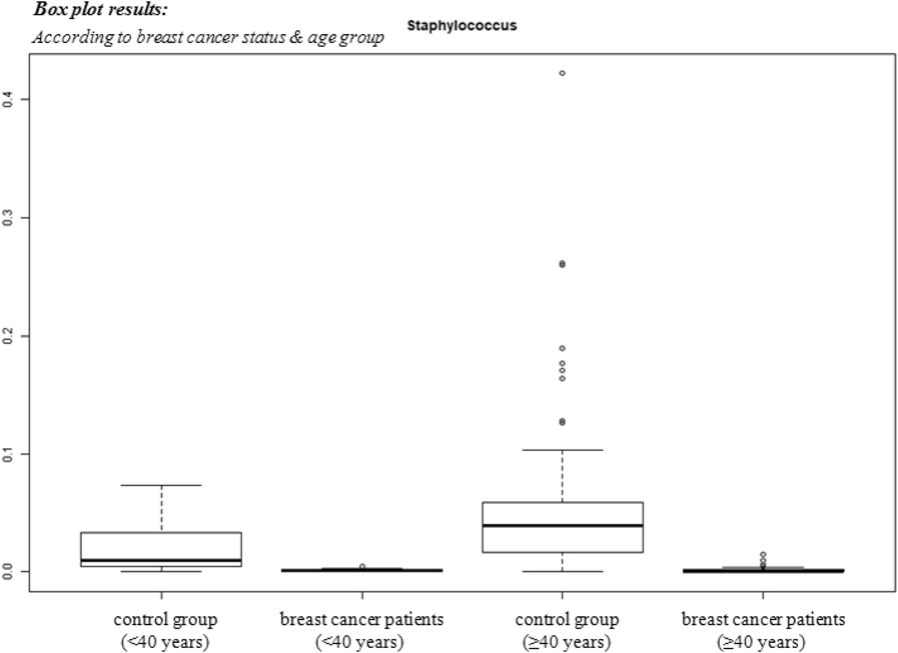
See text for description

**Fig. 3 (abstract P569).**
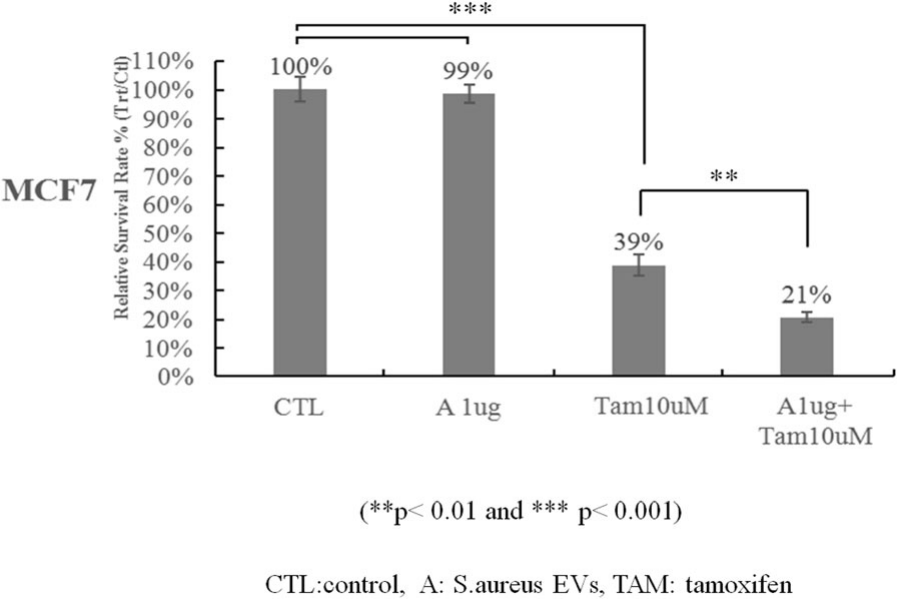
See text for description

**Fig. 4 (abstract P569).**
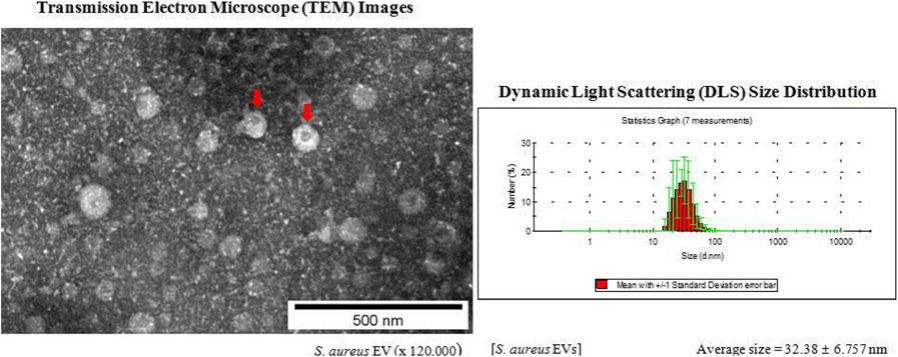
See text for description

**Fig. 5 (abstract P569).**
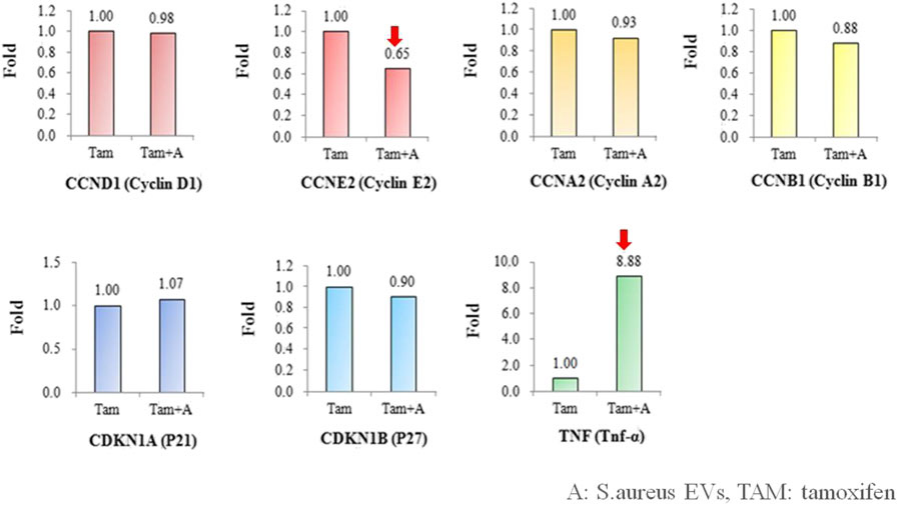
See text for description

**Fig. 6 (abstract P569).**
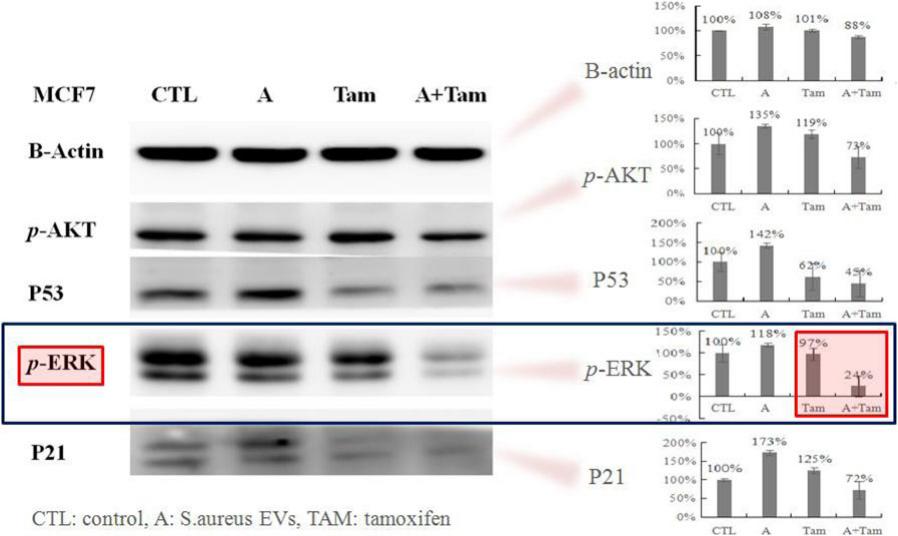
See text for description

**Fig. 7 (abstract P569).**
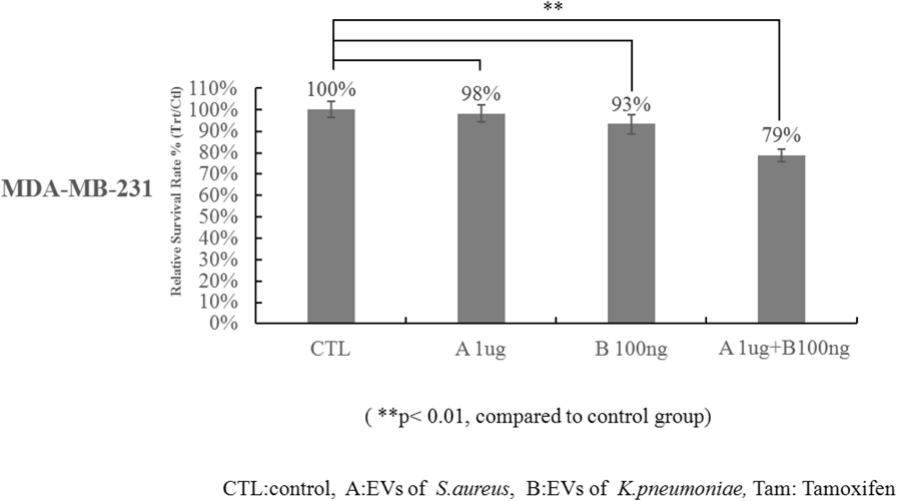
See text for description

#### P570 Commensal bacteria Bifidobacterium stimulates an anti-tumor response via cross-reactivity

##### Catherine Bessell, BA^1^, Catherine Bessell, BA^1^, Ariel Isser^1^, Jonathan Havel, PhD^2^, Sangyun Lee, PhD^3^, Ruhong Zhou, PhD^4^, Jonathan Schneck, MD, PhD^1^, David Bell, PhD^3^

###### ^1^Johns Hopkins University, Baltimore, MD, USA; ^2^Memorial Sloan Kettering Cancer Center, New York, NY, USA; ^3^IBM Thomas J. Watson Research Center, New York, NY, USA; ^4^Columbia University, New York, NY, USA

####### **Correspondence:** Jonathan Schneck (chant@mskcc.org)

**Background**

While recent studies have shown an important role of the microbiome in modulating anti-tumor immune responses, its mechanism remains unclear. One proposed mechanism is due to cross-reactivity between antigens expressed in commensal bacteria and neoepitopes found in tumors. We have identified a cross-reactive antigen expressed in commensal bacteria Bifidobacterium (Bifido) “SVYRYYGL” (henceforth called SVY) and show that it conveys a neoantigen-specific cross-reactivity to the classic neoantigen “SIY.”

**Methods**

The SVY-specific response was analyzed through biophysical experiments and molecular dynamics simulations to determine antigen processing and MHC binding. T cell expansion studies from SIY and SVY T cell populations along with cross specificity studies reveals the cross-reactive T cell populations. B6 mice housed from Jackson, Bifido colonized mice, and Taconic, Bifido lacking, mice were used for examine Bifido colonization on T cell expansion. Sorting cross-reactive T cell populations from Bifido positive or negative mice based on antigen specificity and T cell receptor (TCR) beta sequencing allows to examine the effect of colonization on TCR repertoire composition. Finally the anti-tumor activity of the commensal bacteria population against the cross- reactive tumor antigen was tested by adoptive transfer studies with B16-SIY melanoma model.

**Results**

The SVY-specific response results from SVY peptide binding the H2-Kb MHC and can be processed from whole bacteria. The commensal bacteria SVY-specific T cells population has a cross-reactive SIY-specific T cell response and can recognize tumors expressing the “SIY” antigen. Mice lacking Bifido have a decreased SVY-specific T cell response and an altered (TCR) repertoire compared to Bifido. colonized animals. Bifido. colonization not only shapes the SVY-specific TCR repertoire but selects for clones that are represented in the SIY TCR repertoire. Cross- reactive SVY-specific T cells recognize tumors bearing SIY in vivo in an adoptive T cell transfer model of murine melanoma and leads to decreased tumor growth and extended survival.

**Conclusions**

Our work demonstrates that commensal bacteria can directly stimulate anti-tumor immune responses via T cell cross-reactivity and provides a proof of principle for how bacterial antigens can shape the T-cell landscape.

#### P571 Targeted sequencing of 16s rRNA Gene to understand the diversity and composition of the gut microbiome

##### Rajesh Gottimukkala, MS^1^, Jianping Zheng^2^, Karen Clyde, PhD^2^, Fiona Hyland^2^, Janice Au-Young, PhD^2^

###### ^1^ThermoFisher Scientific, Fremont, CA, USA; ^2^Thermo Fisher Scientific, south san francisco, CA, USA

####### **Correspondence:** Rajesh Gottimukkala (rajesh.gottimukkala@thermofisher.com)

**Background**

Recent studies in humans and experiments in mouse models demonstrated the key role of the gut microbiota in modulating the tumor response to check point blockade immunotherapy. One study showed an association between negative outcome using CTLA-4 blockade therapy and the absence of a specific gut microbiome. So, the gut microbiota has emerged as a promising biomarker to assess the efficacy of immune-modulatory drugs. Next generation sequencing of the 16S rRNA Gene is widely used as standard for understanding the composition of the gut microbiome.

**Methods**

The AmpliSeq pan-Bacterial Research panel that contains 24 primer pairs targeting the 16S rRNA gene provides a cost-effective approach to identify the bacterial species present in the sample. Due to highly homologous nature of 16S sequences, it is challenging to correctly identify organisms at the Genus/Species level using short reads. We have developed a new algorithm that can identify all the organisms in the 16S database at Genus level and a majority at Species level. For every sequence in the database, we construct a coverage pattern using the aligned reads across the multiple amplicons. By matching the observed pattern per sequence with an expected pattern that is pre-computed we can identify the organisms present in the sample. The algorithm reports the identified microbes with Genus/Species level taxonomic classifications and the relative abundance of the organisms in the sample.

**Results**

We sequenced DNA from 12 fecal samples with the assay using Ion GeneStudio S5 System and detected the 25 frequently observed Genera across all the samples including Bifidobacterium, Lactobacillus, Clostridium, Ruminococcus and Bacteroides etc. We sequenced a metagenomics mock community sample comprising of 20 different strains and identified all the 20 species including few organisms relevant to cancer microbiome studies like H.pylori, E.Faecalis, B.vulgatus etc. We did an in-silico analysis using the primers in the assay and demonstrated that using the assay we can identify the frequent bacterial microbes in Gut microbiome resolved to Genus and/or Species level.

**Conclusions**

The AmpliSeq Pan-Bacterial Research panel with the described Bioinformatics pipeline will enable usage of 16s rRNA sequencing to assess the Gut microbiome as a biomarker for immunotherapy.

#### P572 Variation of the gut microbiome of complete responders to immune checkpoint blockade and healthy individuals – implications for clinical trial design

##### Beth Helmink, MD PhD^1^, Vancheswaran Gopalakrishnan, MPH, PhD^1^, Abdul Wadud Khan, MD^1^, Pierre-Olivier Gaudreau^1^, Elizabeth Sirmans^1^, Elizabeth Burton^1^, Vanessa Jensen, DVM^1^, Adrienne Duran, BAS^1^, Linsey Martin^1^, Angela Harris^1^, Miles Andrews, MD, PhD^1^, Jennifer McQuade, MD^1^, Alexandria Cogdill, MEng^1^, Christine Spencer, PhD^1^, Reetakshi Arora^1^, Nadim Ajami, PhD^1^, Joseph Petrosino, PhD^2^, Jamal Mohamed^1^, Sapna Patel, MD^1^, Michael Wong, MD PhD FRCPC^1^, Rodabe Amaria, MD^1^, Jeffrey Gershenwald, MD^1^, Patrick Hwu, MD^1^, Wen-Jen Hwu, MD, PhD^1^, Michael Davies, MD, PhD^1^, Isabella Glitza, MD, PhD^1^, Hussein Tawbi, MD, PhD^1^, George Marnellos^3^, Jaclyn Sceneay^3^, Jennifer Wortman^3^, Lata Jayaraman^3^, David Cook^3^, Theresa LaVallee^4^, Robert Jenq, MD^1^, Timothy Heffernan, PhD^1^, Jennifer Wargo, MD, MMSc^1^

###### ^1^MD Anderson Cancer Center, Houston, TX, USA; ^2^Baylor College of Medicine, Houston, TX, USA; ^3^Seres Therapeutics, Cambridge, MA, USA; ^4^Parker Institute Cancer Immunotherapy, San Francisco, CA, USA

####### **Correspondence:** Jennifer Wargo (JWargo@mdanderson.org)

**Background**

The gut microbiome has been shown to have profound influences on host and anti-tumor immunity, and pre-clinical studies suggest that gut microbiota may be modulated to enhance responses to immune checkpoint blockade [1-4]. Recent studies demonstrate differences in the gut microbiome of responders (Rs) versus non-responders (NRs) to anti-PD-1 therapy in patients [5-8], with identification of a microbiome signature associated with a 100% response rate (Type-1 signature) [5]. Several clinical trials are in development/underway that aim to modulate the microbiome to augment responses to immune checkpoint blockade. These are, in part, based on foundational evidence that treatment with fecal microbiota transplant (FMT) from healthy donors is associated with clinical responses in other diseases (*C. difficile* infection and inflammatory bowel disease, CDI and IBD)[9]; however, the optimal donors for FMT to enhance responses to immune checkpoint blockade remain incompletely understood.

**Methods**

To address this critical question, we performed profiling of the gut microbiota (via 16s and metagenomic sequencing) in a cohort of patients with complete responses (CRs) to anti-PD-1 therapy (n=11) versus healthy controls^10^ (n=116). Importantly, immune profiling was also performed in available baseline tumor biopsies from CRs. Diversity (inverse Simpson) and composition of the gut microbiota was assessed in each of these cohorts, and FMT of selected CR donors versus a known NR (n=3 and 1, respectively) was then performed into gnotobiotic mice and melanoma tumors were implanted. Mice were then treated with immune checkpoint blockade. Tumor outgrowth was assessed and longitudinal microbiome analyses and immune profiling of tumor and the periphery in FMT- treated mice were also performed.

**Results**

Characterization of gut microbiota revealed wide variation in the diversity and composition of the gut microbiota, with preliminary work demonstrating a trend towards higher diversity in CR donors versus healthy controls (p=0.2); validation in a larger cohort of CRs is ongoing. Interestingly, not all CRs demonstrated a Type-1-like signature (with higher relative abundance of Clostridiales versus Bacteroidales) (27%, n=3/11) nor did healthy controls 28% (n=33/116). This has critical implications for FMT donor selection in immune checkpoint blockade trials (versus those for CDI or IBD). Murine studies demonstrated reduced tumor growth in CR-FMT mice vs. NR-FMT mice, with variability noted between donors. Immune profiling in available patient tumor samples and in murine studies and comparisons to gut microbiota are currently being performed.

**Conclusions**

Together, these studies provide important information about potential donor selection in FMT trials in immunotherapy, warranting additional studies and translational research.

**References**

1. Borody TJ, Khoruts A. Fecal microbiota transplantation and emerging applications. Nat Rev Gastroenterol Hepatol. 2011;9:88-96.
2. Frankel AE, Coughlin LA, Kim J, Froehlich TW, Xie Y, Frenkel EP, Koh AY. Metagenomic shotgun sequencing and unbiased metabolomic profiling identify specific human gut microbiota and metabolites associated with immune checkpoint therapy efficacy in melanoma patients(). Neoplasia (New York, NY). 2017;19,848-855.
3. Garrett WS. Cancer and the microbiota. Science. 2015; 348, 80-86.
4. Gopalakrishnan V, Spencer CN, Nezi L, Reuben A, Andrews MC, Karpinets TV, Prieto PA, Vicente D, Hoffman K, Wei SC, et al. Gut microbiome modulates response to anti-PD-1 immunotherapy in melanoma patients. Science. 2017.
5. Matson V, Fessler J, Bao R, Chongsuwat T, Zha Y, Alegre ML, Luke JJ, and Gajewski TF. The commensal microbiome is associated with anti-PD-1 efficacy in metastatic melanoma patients. Science. 2018; 359, 104-108.
6. McDonald D, Hyde E, Debelius JW, Morton JT, Gonzalez A, Ackermann G, Aksenov AA, Behsaz B, Brennan C, Chen Y, et al. American gut: an open platform for citizen science microbiome research. mSystems 3. 2018.
7. Routy B, Le Chatelier E, Derosa L, Duong CPM, Alou MT, Daillere R, Fluckiger A, Messaoudene M, Rauber C, Roberti MP, et al. Gut microbiome influences efficacy of PD-1-based immunotherapy against epithelial tumors. Science. 2017.
8. Segre JA. Microbiome. Microbial growth dynamics and human disease. Science. 2015; 349, 1058-1059.
9. Sivan A, Corrales L, Hubert N, Williams JB, Aquino-Michaels K, Earley ZM, Benyamin FW, Lei YM, Jabri B, Alegre ML, et al. Commensal Bifidobacterium promotes antitumor immunity and facilitates anti-PD-L1 efficacy. Science. 2015; 350, 1084-1089.
10. Vetizou M, Pitt JM, Daillere R, Lepage P, Waldschmitt N, Flament C, Rusakiewicz S, Routy B, Roberti MP, Duong CP, et al. Anticancer immunotherapy by CTLA-4 blockade relies on the gut microbiota. Science. 2015; 350,1079-1084.

**Ethics Approval**

The study was approved by MD Anderson Cancer Center Institutution‘s Ethics Board, approval numbers LAB00-063, PA15-0232, and RN00001344-RN01.

#### P573 Antibiotic use and clinical outcomes of PD-1 antagonists in advanced non-small cell lung cancers

##### Amit Kulkarni, MD, Manish Patel, DO, Ying Wang, MD, PhD, Todd Defor, MS

###### University of Minnesota, Minneapolis, MN, USA

####### **Correspondence:** Manish Patel (patel069@umn.edu)

**Background**

Pre-clinical evidence in mice suggests that antibiotics induced dysbiosis can negatively influence efficacy of immune check-point inhibitors (ICI). The effects of antibiotic use on clinical outcomes of ICI are limited and has yielded inconsistent results. We evaluated whether antibiotic use impacts efficacy of PD-1 inhibitors in patients with advanced non-small-cell lung cancer (NSCLC)

**Methods**

We retrospectively reviewed clinical outcomes of advanced NSCLC patients treated with nivolumab or pembrolizumab at our institution between 5/2015 to 5/2018. Patients who received antibiotics 3 months prior to initiating ICI were considered to be in the Antibiotic exposure (ATB+) group. The remainder of patients were included in antibiotic naïve (ATB-) group. The primary outcome was clinical benefit rate (CBR) defined as proportion of patients with complete response (CR), partial response (PR) or stable disease (SD) per RECIST 1.1 among patients eligible for response assessment. Secondary outcomes of interest included progression-free survival (PFS) and overall survival (OS) across the entire patient population. Logistic regression and Cox proportional hazards model were used to compare outcomes between ATB+ and ATB- groups.

**Results**

111 patients were included in the analysis. The median age was 66 years (range 36-87 years). The majority of patients were female (55%), Caucasian (94%) and had adenocarcinoma (61%) histology. Most patients received Nivolumab (91%) in the second or subsequent line. 30% patients had brain metastasis prior to receiving ICI. 44 (39%) patients received antibiotics up to 3 months prior to ICI initiation, most received fluoroquinolones for respiratory infections. 96 patients were eligible for response assessment (>3 doses before restaging). Antibiotics exposure did not impact CBR, 63% in ATB+ and 53% in ATB- group (OR=1.5; p=0.36). Similarly, no statistically significant difference was seen in median PFS and OS between the two groups (PFS 4.1 months vs 2.8 months, p=0.66 and OS of 12 months vs 10 months, p=0.4 in ATB+ and ATB- group respectively). HR for association of antibiotic use with PFS and OS was 0.8 (p=0.36) and 0.8 (p=0.25) respectively. No significant difference was noted when controlling for age, sex, ECOG status, prior lines of therapy, brain metastasis and steroid use.

**Conclusions**

To our knowledge, this is the largest study showing clinical outcomes are not affected by prior antibiotic use in NSCLC patients receiving ICI. While our study has limitations, more studies are needed to establish an association. Data analysis of more patients is currently underway that will be reported in the final analysis prior to meeting.

#### P574 A rationally-designed consortium of human gut commensals induces CD8 T cells and modulates host anti- cancer immunity

##### Bruce Roberts, PhD^6^, Takeshi Tanoue^1^, Satoru Morita^1^, Koji Atarashi^1^, Wataru Suda^2^, Damian Plichta^3^, Seiko Narushima^4^, Ashwin Skelly^1^, Atsushi Shiota^5^, Jason Norman^6^, Vanni Bucci^7^, Yutaka Kawakami, MD PhD^1^, Masahira Hattori^2^, Ramnik Xavier^3^, Bernat Olle^6^, Bruce Roberts, PhD^6^, Kenya Honda, MD, PhD^8^

###### ^1^Keio University School of Medicine, Tokyo, Japan; ^2^Waseda University, Tokyo, Japan; ^3^Broad Institute of MIT and Harvard, Cambridge, MA, USA; ^4^Riken Center for Integrative Medical Science, Kanagawa, Japan; ^5^JSR-Keio University Innovation Center, Tokyo, Japan; ^6^Vedanta Biosciences, Cambridge, MA, USA; ^7^University of Massachusetts, North Dartmouth, MA, USA; ^8^Keio University School of Medicine and JSR-Keio University Innovation Center, Tokyo, Japan

####### **Correspondence:** Bruce Roberts (broberts@vedantabio.com)

**Background**

Clinical data suggests the gut microbiome influences response to checkpoint inhibitor therapy however the precise identity and mode of action of commensals associated with clinical response has not been elucidated. We report the generation of a consortium of human gut derived commensals capable of inducing CD8 T cells and augmenting anti- cancer immunity.

**Methods**

The microbiota of healthy humans was used to inoculate germ-free mice and assess the level of CD8 T cell induction. Human derived commensals were isolated from inoculated mice exhibiting high levels of CD8 T cell induction and sequenced. Consortia consisting of isolated human commensals were tested for the ability to induce CD8 T cells in germ-free and SPF mice. A minimal consortium capable of inducing CD8 T cells was administered with checkpoint inhibitor antibodies to tumor-bearing mice to assess anti-cancer activity and the level of accumulation of tumor infiltrating lymphocytes.

**Results**

Interferon-gamma producing CD8 T are abundant in the intestines of SPF but not germ-free mice. A consortium of human-derived commensals dubbed VE800 which robustly induces CD8 T cells in germ-free mice was identified.

VE800 administration promotes activation of intestinal dendritic cells and stimulation of interferon-gamma producing CD8 T cells is dependent on the transcription factor BATF3. Comparative gene pathway analysis revealed several of the VE800 strains are related to strains associated with favorable clinical response in metastatic melanoma patients treated with immunotherapy. Administration of the VE800 cocktail with anti-CTLA4 enhanced anti-tumor activity and survival in the MC38 tumor model. VE800 also enhanced the anti-tumor activity of anti-PD1 in the MC38 and B-raf Pten melanoma tumor models. VE800 treatment alone is sufficient to enhance the level of tumor infiltrating CD8 T cells in the MC38 model to a level comparable to anti-PD1 alone however the combination of VE800 and anti-PD1 promoted the highest level of tumor infiltrating CD8 T cells in the MC38 model as well as in the more aggressive B-raf Pten model. VE800 administration promoted enhanced accumulation of interferon- gamma producing CD8 T cells in the spleens of tumor-bearing mice indicating the consortium promotes systemic cellular immune cell activation.

**Conclusions**

A rationally-designed consortium of human gut-derived commensals induces CD8 T cells in vivo and potentiates anti-cancer immunity when administered with checkpoint inhibitors. Given the consortium can be produced via cGMP manufacturing and administered orally on a repeated basis, VE800 constitutes a safe agent for alteration of the microbiome of cancer patients to enhance anti-cancer immunity.

#### P575 Classification of the human gut microbiome using a validated 16S rRNA next generation sequencing method

##### Janet Doolittle-Hall, Melissa Howard, Jennifer Sims, Scott Yourstone, Jason Powers, Patrick Hurban, PhD, Victor Weigman

###### Q2 Lab Solutions, Morrisville, NC, USA

####### **Correspondence:** Janet Doolittle-Hall (Janet.Doolittle-Hall@q2labsolutions.com)

**Background**

Analysis of the gut microbiome composition is becoming increasingly important given its influence on a wide variety of human diseases including cancer. Numerous studies have indicated that the gut microbiome can influence cancer susceptibility, tumorigenesis and cancer progression at least in part through its profound impact on the immune cell function and its inherent metabolic capacity. Emerging evidence suggest that gut microbiome can be manipulated for improving the effects of cancer therapies. Microbiome composition and relative abundance of different microbial taxa can be measured by combining DNA sequencing of hypervariable regions of the 16S ribosomal RNA gene or a whole‐genome shotgun sequencing with computational analysis. The scientific and clinical utility of microbial analysis by NGS strongly depends on the accuracy and precision of identifying and quantitating the microbial taxa. Here we report on the development and validation of a new assay and bioinformatics analysis pipeline for accurate taxonomic classification of complex microbial samples such as stool using 16S rRNA sequencing.

**Methods**

DNA isolation from stool was performed using a validated MoBio Power Soil method. Illumina 16S rRNA targeted sequencing was performed using custom PCR amplification primers for the bacterial 16S V3 and V4 regions and a 2x300 bp paired-end strategy. A bioinformatic sequence alignment and classification pipeline was developed to enable accurate taxonomic identification of constituent bacteria based on genetic differences in the hypervariable regions of the 16S rRNA gene. Output includes taxonomic classification and relative abundance of the identified taxa.

**Results**

Assay analytical performance was determined using admixtures of 4 bacterial strains, at varying levels, into human reference DNA. Correct bacterial species present at or above 0.01% relative abundance were detected with >99.9% accuracy and 100% detection sensitivity. Clinical feasibility with human stool samples is ongoing. In addition, feasibility of recovering microbial communities from formalin-fixed paraffin-embedded (FFPE) tumor tissues was demonstrated using short amplicon/ multiple primer Ion Torrent 16S rRNA sequencing method. Composition and structure of the recovered microbial communities were affected by the FFPE preparation methods, highlighting the need for standardization of the pre-processing procedures.

**Conclusions**

Analytically validated 16S rRNA sequencing assay, with our computational pipeline, offers an option for accurate identification and classification of constituent microbiome components from complex mixtures at a lower coverage and cost option than would be required for shotgun metagenomic approaches. In addition, analysis of microbial communities is feasible from the FFPE tumor tissue.

### Micro-RNA, Epigenetics and Tumor/Immune-cell Signaling Pathways in Anti-Tumor Immunity

#### P576 Evaluating the importance of inhibiting HDAC6 in metastatic breast cancer to enhance the efficacy of immunotherapy

##### Debarati Banik, PhD^2^, Erica Palmer, BS^2^, Melissa Beaty, MS^2^, Satish Noonepalle, PhD^2^, Maria Hernendez, BS^2^, Prathima Vembu, MS^2^, Alejandro Villagra, PhD^2^

###### ^1^George Washington University, Washington DC, USA; ^2^The George Washington University, Washington DC, USA

####### **Correspondence:** Alejandro Villagra (avillagra@email.gwu.edu)

**Background**

Histone deacetylases (HDAC) are recognized to perform diverse functionalities beyond their conventional roles in remodeling the chromatin landscape. These functionalities may range from regulating the outcomes of cellular- health to local or systemic immune-diseases including autoimmunity and cancer, positioning the HDAC inhibitors (HDACi) at a crucial junction of immunotherapy. The excessive toxicity and variability among broad-spectrum HDACi have led to the development of more selective inhibitors, which helped to understand the individual roles of HDACs in shaping anti-tumor immune responses. One such member HDAC6 is reported to promote the pro- tumorigenic STAT3 pathway. By using ultra-selective HDAC6i, the downstream immune-modulatory pathways of STAT3, e.g. co-stimulatory pathways of PD-L1, PD-L2 and B7-H4 could be targeted. HDAC6 has been also involved in a number of structural functions related to cellular motility, shape and intracellular transport through the regulation of the acetylation of numerous targets, including tubulin and cortactin. This function is strongly suggestive of HDAC6 being a key player in metastatic cancer progression. We further hypothesize that by means of modulating PDL1 pathway, the TME and cytoskeletal molecules, HDAC6i may enhance the efficacy of anti-PD1 therapy in Triple Megative Breast Cancer (TNBC).

**Methods**

4T1 was used as a model for murine TNBC implanted orthotopically. Both in vitro and in vivo methods were used to investigate the HDAC6i NexturastatA, with or without combining with anti-PD1 antibody in vivo.

**Results**

NexturastatA was able to reduce primary tumor growth, as well as inhibit tumor invasion and modify the expression of EMT-specific gene signature, even in presence of metastasis-promoting cytokine IL6. Additionally, the size and number of secondary tumor nodules in the lungs were significantly diminished after the HDAC6i treatment. NexturastatA was also able to inhibit anti-PD1 antibody mediated enhancement in PDL1 expression in vitro, suggesting the utility of combining it with checkpoint inhibitor in vivo. While both anti-PD1 and CTLA4 treatments showed certain degrees of success in reducing tumor growth, we demonstrated that in a pre-treatment setting, HDAC6i improves anti-tumor responses when combined with anti-PD1. This was measured in terms of the primary and secondary tumor growth, composition of infiltrating immune cells in the primary tumor, EMT gene signature, and expression of co-stimulatory molecules as well as intra-tumoral interferon gamma expression, indicative of intra-tumoral effector T cell functionality.

**Conclusions**

NexturastatA alone and in combination with anti-PD1 antibody was able to modify some of the critical features of invasion and metastasis as well as properties of tumor microenvironment in TNBC.

**Ethics Approval**

The study containing animals was approved by the IACUC of the George Washington University under protocol number A385.

#### P577 Activation of GSK3-beta in the melanoma tumor microenvironment renders dendritic cells refractory to immune suppression and induces T cell activation and oncolysis

##### Marta Lopez Gonzalez, Msc, Rieneke van de Ven, PhD, Anita Stam, Wen Dong, Victor van Beusechem, Tanja de Gruijl, PhD

###### CCA Amsterdam UMC, Amsterdam, Netherlands

####### **Correspondence:** Marta Lopez Gonzalez (m.lopezgonzalez@vumc.nl)

**Background**

Immune checkpoint blockade results in durable clinical responses in only a fraction of treated patients. There is a growing awareness that for immune checkpoint inhibitors to be effective, sufficient tumor infiltration by effector T cells is an absolute requirement. However, a crippled myeloid compartment in the tumor microenvironment (TME) will stand in the way of T cell activation and recruitment, due to the absence of properly developed and activated dendritic cells (DC), which, as a result, won’t be able to cross-prime antitumor cytotoxic effector T cells and will fail to attract an effector T cell infiltrate through the release of key chemokines. It is therefore vital to develop methodologies to normalize DC differentiation and activation in the melanoma TME.

**Methods**

N/A

**Results**

We have recently uncovered a key role for GSK3β, a known repressor of Wnt signaling, in the control of DC maturation, both at the level of melanoma cells and at the level of DC and their precursors. Employing lysates from IL-10 modulated DC precursors on a peptide kinase substrate microarray, we identified putative signaling networks at play in melanoma-associated DC suppression. GSK3β came out on top of a list of modulated kinases and STRING network analysis revealed links to JAK/STAT, MAPK and Wnt signaling pathways, all previously implicated in cancer-mediated immune suppression. Using melanoma cell line supernatants and co-cultures (employing a set of 5 melanoma cell lines encompassing various oncogenic mutations, including BRAFv600, PTEN, and NRAS), we found that enforced overexpression of constitutively active GKS3β (CA.GSK3β) rendered DC differentiation and maturation refractory to the suppressive effects of melanoma, which appeared to involve both soluble mediators and cell-cell contact. This immune stimulatory effect was accompanied by decreased levels of both phosphorylated and non-phosphorylated β-Catenin in tumor cells, consistent with its reported degradation by activated GSK3β. Of note, virally enforced over-expression of CA.GSK3β in melanoma cells also reduced their DC- suppressive effects and at later time points reduced their proliferative ability and viability. As ex-vivo proof of concept of the therapeutic modulation of GKS3β in the melanoma TME, single-cell suspensions from melanoma metastases (n=4) were transduced with CA.GSK3β. As a result, we observed activation of DC subsets and of infiltrating T cells, reduced IL-10 levels, and specific tumor cell lysis.

**Conclusions**

We conclude that activation of GSK3β in the melanoma TME may simultaneously induce oncolysis and alleviate DC immune suppression, thereby enabling T cell activation and effective immune checkpoint blockade.

#### P578 Application of multiplexed immunofluorescence and multispectral imaging to investigate TGFβ pathway activation of immune cell populations in human lung cancer

##### Sebastian Marwitz^1^, Carmen Ballesteros Merino, PhD^2^, Shawn Jensen, PhD^1^, Bernard Fox, PhD^2^

###### ^1^Robert W Franz Cancer Center, Borstel, Germany; ^2^Robert W Franc Cancer Center, Portland, OR, USA

####### **Correspondence:** Bernard Fox (foxb@foxlab.org)

**Background**

Non-Small Cell Lung Cancer (NSCLC) is the leading cause of cancer-related death worldwide and is usually diagnosed in an already locally or systemically advanced state. Depending on the stage of the tumor, surgical therapy is limited and systemic therapy required. Recent developments with targeted therapies and immune checkpoint blockade resulted in improved survival for a limited number of patients but the magnitude of patients will still progress. The transforming growth factor beta signaling pathway (TGFβ) is frequently activated in lung cancer, involved in malignant progression and a possible target for therapy [1]. TGFβ signaling is known to inhibit immune responses, immune cell proliferation and to dampen effector functions in various immune cells [2]. Therefore we set out to investigate the activation of the TGFβ signaling pathway in different immune cell populations in human lung cancer to better define the cells that are affected by TGFβ.

**Methods**

Multiplexed immuno-fluorescence staining and multi-spectral imaging was used to investigate a cohort of > 200 early stage NSCLC specimens assembled on TMAs for activation of the TGFB signaling pathway by targeting phosphorylated SMAD3 and different immune cell markers. Image analyses were conducted to analyze spatial relationships and local abundances in the tumor as well as stroma in tissue samples from the tumor center or margin

**Results**

Overall, a significantly increased number of CD3, proliferating CD3 (p <0.001) and CD3-phospho SMAD3 (p <0.05) cells were observed in the stroma compared to tumor tissues, however the overall percentage of CD3pS3 remained unaltered between tumor and stroma, suggesting an equal impact on both compartments. In addition, adenocarcinomas exhibited a significantly increased abundance of CD3 in the tumor and stroma in both, the invasive margin or the central region of the tumor compared to squamous cell carcinomas.

**Conclusions**

Using multi-parameter tissue analyses to investigate the abundance of specific immune cell populations and pathway activation in the tumor-microenvironment of lung cancer tissues enables detailed analysis of immune signaling phenotypes. Somewhat surprisingly, these studies suggest that TGFβ impacts an equal percentage of CD3 T cells in both the stroma and tumor center.

**Acknowledgements**

Sebastian Marwitz is funded by the Deutsche Forschungsgemeinschaft (DFG) via MA 7800/1-1.

**References**

1. Marwitz S, Depner S, Dvornikov D, Merkle R, Szczygiel M, Müller-Decker K, et al. Downregulation of the TGF-β pseudoreceptor BAMBI in non-small cell lung cancer enhances TGF-β signaling and invasion. Cancer Research. 10.1158/0008-5472.CAN-15-1326.
2. Li MO, Wan YY, Sanjabi S, Robertson A-KL, Flavell RA. TRANSFORMING GROWTH FACTOR-β REGULATION OF IMMUNE RESPONSES. Annual Review of Immunology. 2006; 24(1): 99–146.

**Ethics Approval**

This study was approved by University of Lübeck´s institutional review board number (Az. 18-026).

#### P579 The leukocyte chemoattractant chemerin modulates PTEN and PD-L1 expression and activity via CMKLR1 in human tumors

##### Keith Rennier, PhD, Gurpal Virdi, BS, Woo Jae Shin, BA, Russell Pachynski, MD

###### Washington University School of Medicine, St. Louis, MO, USA

####### **Correspondence:** Russell Pachynski (rkpachynski@wustl.edu)

**Background**

Chemerin (RARRES2) is an endogenous innate leukocyte chemoattractant previously shown to recruit immune cells via its receptor CMKLR1 into the tumor microenvironment and thereby suppresses tumor growth. RARRES2 has been shown to be downregulated in many tumor types, suggesting a mechanism of immune escape. The loss of the key tumor suppressor PTEN has been shown to promote resistance to T cell-mediated immunotherapy and correlates with decreased T cell tumor infiltration in humans, while restored PTEN functions to repress PD-L1 expression. Importantly, chemerin was recently shown to suppress metastatic hepatocellular carcinoma in a mouse model by upregulating the expression and activity of PTEN. Here, we evaluate the effects of chemerin on human tumor lines in vitro and elucidate its relationship with tumor PD-L1 expression and response to T cell anti-tumor immunity.

**Methods**

We utilized human tumor cell lines in vitro and exposed them to exogenous recombinant human chemerin. PTEN and PD-L1 expression in tumor lines was evaluated by RT-qPCR, flow cytometry, and/or western blot analysis. In vitro matrigel invasion assays were performed to investigate the impact of chemerin exposure on tumor intrinsic activity. T cell-mediated cytotoxicity assays explored the downstream effect of chemerin-mediated PD-L1 modulation. Knockdown of CMKLR1, PTEN or PD-L1 using siRNA was performed to demonstrate their mechanistic role in the chemerin-PTEN-PDL1 pathway.

**Results**

We show that recombinant chemerin significantly upregulates PTEN expression in multiple tumor cell lines. Concomitantly, chemerin exposure significantly decreased PD-L1 expression in DU145 tumor cells (Figure 1). Consistent with these data, chemerin treatment results in attenuated tumor cell migration/invasion and increased T cell mediated cytotoxicity in DU145 cells (Figures 2 and 3, respectively). Using siRNA, we found that CMKLR1 knockdown diminished the modulated PTEN activity and PD-L1 expression levels. Similarly, CMKLR1 knockdown completely mitigated chemerin’s effect on tumor invasion and T cell mediated cytotoxicity, suggesting the functional significance of the chemerin/PTEN/PD-L1 axis in human tumor cells. Importantly, T cell cytotoxicity assays showed chemerin-mediated increases were comparable to direct knockdown of PD-L1 and an anti-PD-L1 antibody (atezolizumab) (Figure 4).

**Conclusions**

Here, we characterize for the first time a novel chemerin-PTEN-PDL1 interaction in human tumor cells. We have shown treatment with exogenous chemerin, through induction of PTEN, significantly reduces tumor migration and increases T cell mediated cytotoxicity via its impact on tumor PD-L1 expression. Ongoing experiments are focused on in vivo mechanisms of action and will serve as the basis for translational studies.

**Fig. 1 (abstract P579).**
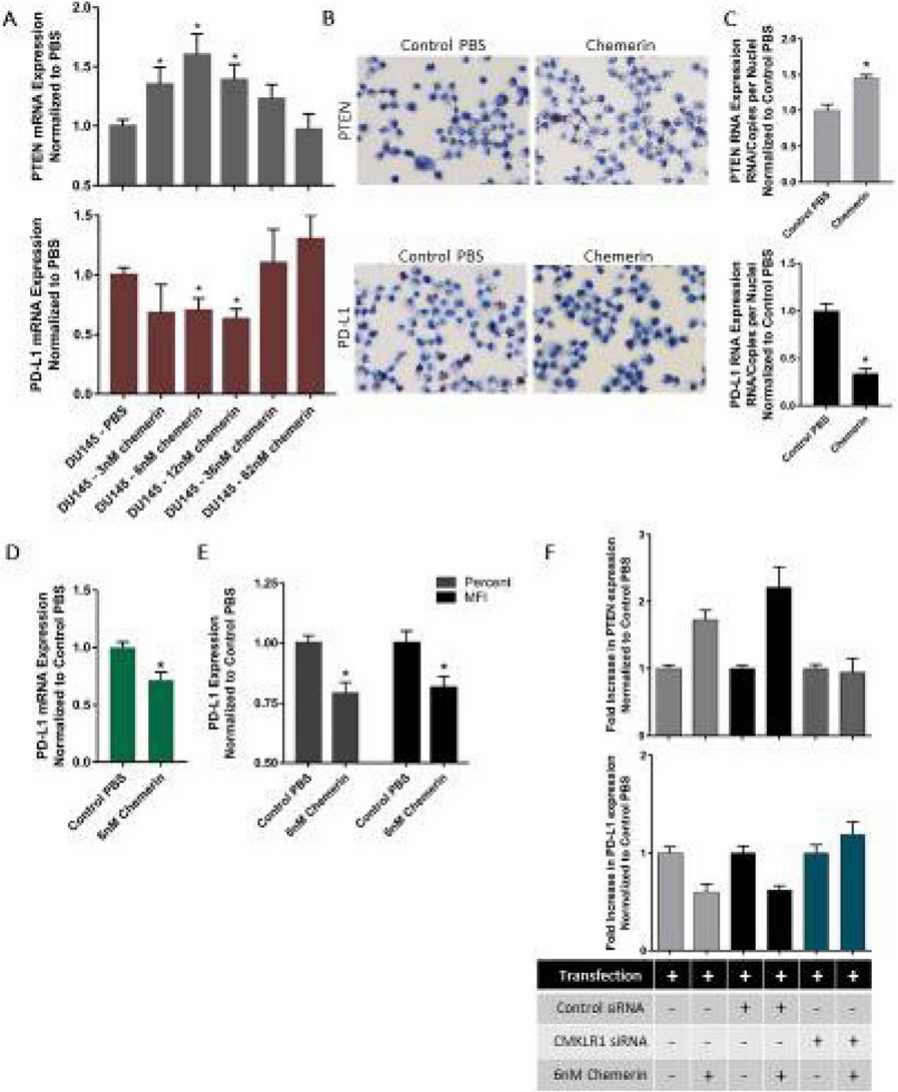
Chemerin upregulates PTEN decreases PD-L1 via CMKLR1

**Fig. 2 (abstract P579).**
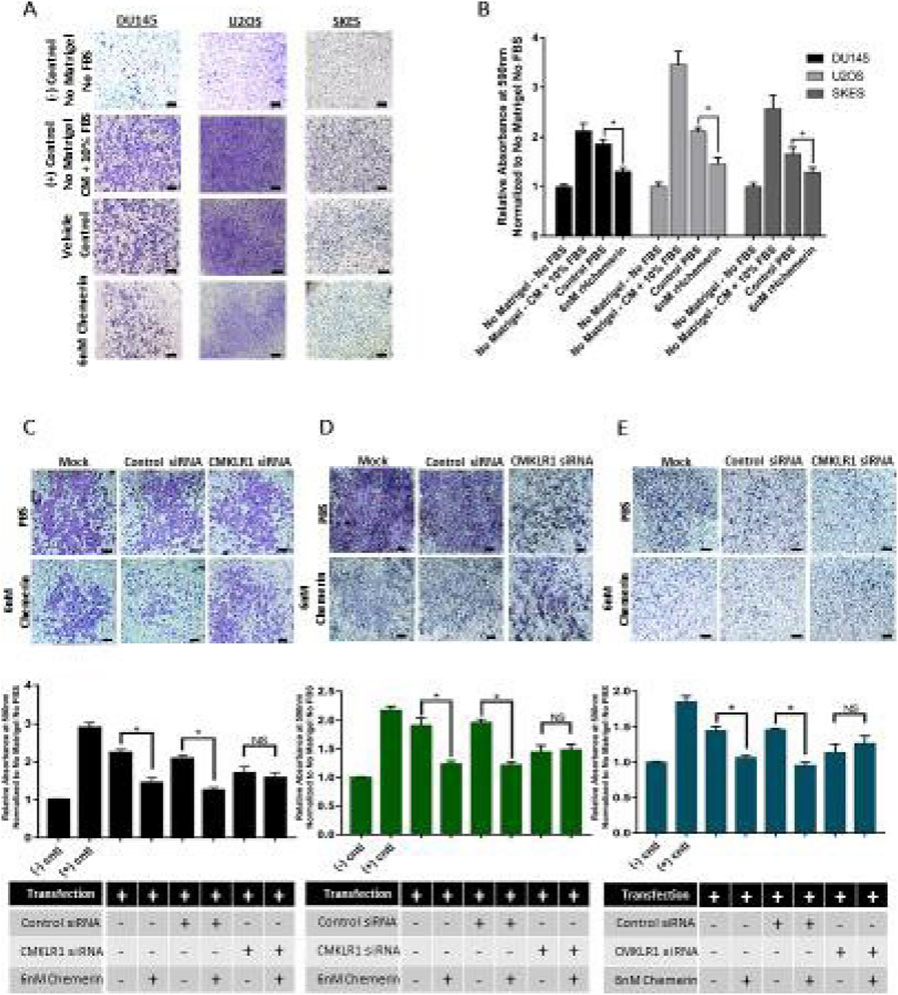
Chemerin diminishes tumor cell invasion via CMKLR1

**Fig. 3 (abstract P579).**
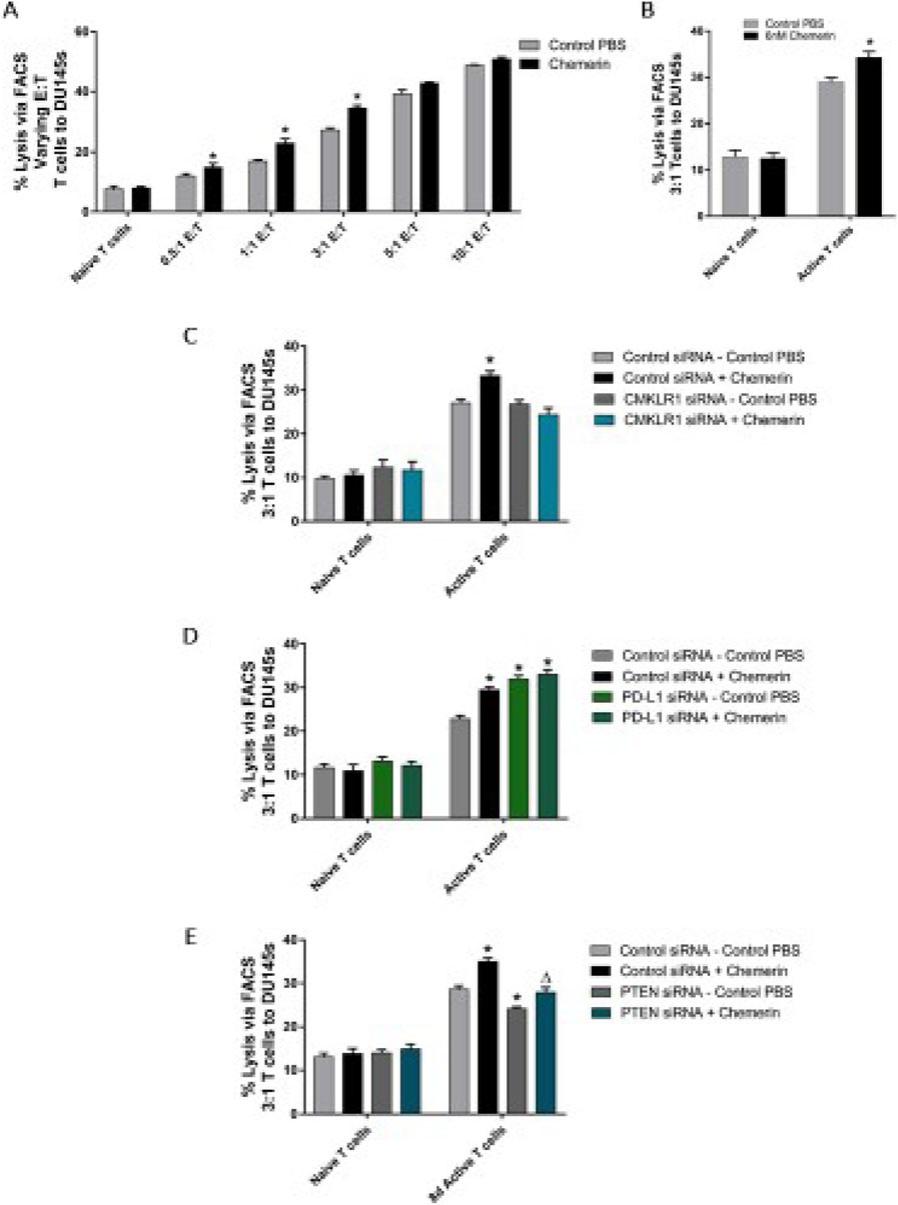
Cytotoxicity increased chemerin-treated tumor cells

**Fig. 4 (abstract P579).**
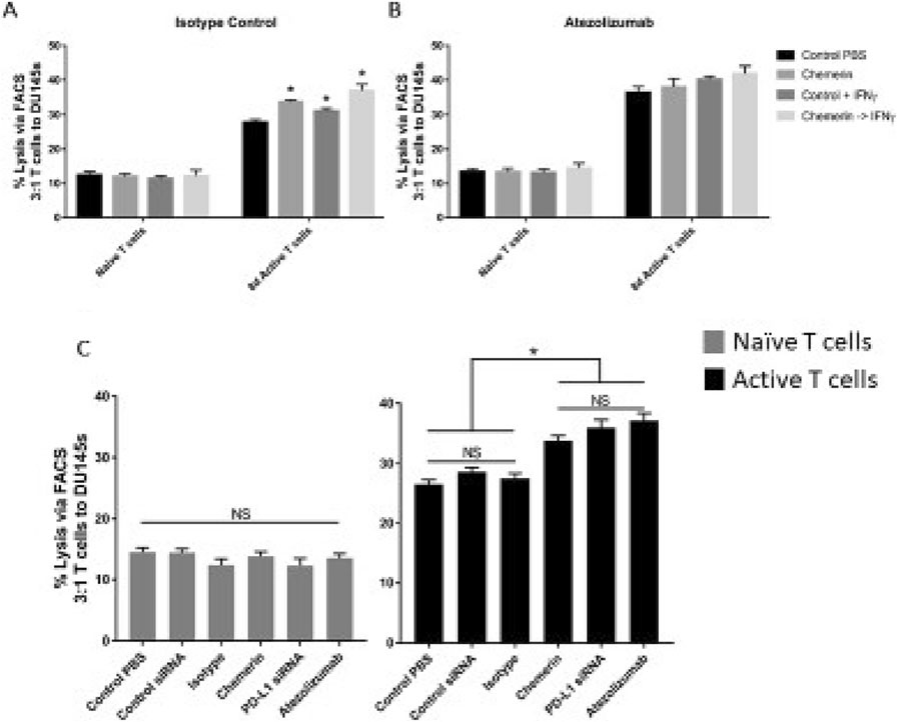
Chemerin treatment comp PD-L1 siRNA & atezolizumab

### Oncogenetics and Immunogenomics

#### P580 Genomic portraits of immune escape mechanism in cold tumours

##### Venkateswar Addala, PhD - Research Student^1^, Futoshi Kawamata^1^, Stephen Kazakoff, PhD^1^, Pamela Mukhopadhyay^1^, Catherine Bond^1^, Katia Nones^1^, Felicity Newell^1^, Jennifer Borowsky^1^, Scott Wood^1^, Conrad Leonard^1^, Qinying Xu^1^, Matthew E Burge^2^, Akinobu Taketomi^3^, Toshiya Kamiyama^1^, Barbara Leggett, MD FRACP^1^, John Pearson^1^, Vicki Whitehall^1^, Ann-Marie Patch^3^, Nic Waddell^3^

###### ^1^QIMR Berghofer Medical Research Institute, Brisbane, QLD, Australia; ^2^Royal Brisbane and Women’s Hospital, Brisbane, Australia; ^3^Hokkaido University, Sapporo, Japan

####### **Correspondence:** Ann-Marie Patch (Ann-Marie.Patch@qimrberghofer.edu.au)

**Background**

Immunotherapy promises to revolutionise cancer treatment. ‘Hot’ tumors are responsive to immune checkpoint blockade that ‘releases the breaks’ of immune system to target tumor cells. High mutation/neoantigen burden with active tumor microenvironment markers predicts response to immunotherapy in tumors associated with UV damage in melanoma and microsatellite instability in colorectal cancer (CRC). However ‘cold’ tumours remain a major research challenge as they exist in an immune suppressed environment. In this study, we investigated the immune escape mechanism of microsatellite stable primary CRC and matched liver metastasis samples.

**Methods**

Whole genome sequencing and RNA sequencing of 15 matched primary CRC and matched liver metastases patients was performed. DNA and RNA Sequence analysis was performed using previously described methods [1] [2]. Four digit HLA class I allele types were determined by Polysolver [3] and Optitype [4] and loss of heterozygosity in the HLA loci was identified from tumor and normal samples using LOHHLA [5]. The pVAC-Seq neoantigen prediction framework [6] was applied to identify the neoantigens using NetMHCpan algorithm. Deconvolution of Immune cells were estimated using CIBERSORT [7] and TCR repertoire diversity predicted through MixCR [8] approach in all paired samples.

**Results**

Frequently shared neoantigens with IC50<500nM were identified in both primary tumor and liver metastasis but did not identify any mutations in antigen processing machinery genes. HLA allele specific loss of heterozygosity occurs in the majority of primary and metastatic samples whereas one metastatic sample showed inconsistency in HLA genotype from germline and primary tumor. The tumor micro-environment is dominated by lymphocytes in the primary tumor and macrophages in liver metastasis. TCR repertoire diversity found to be decreased at metastatic stage in few patients.

**Conclusions**

We found several potential immune escape mechanisms in these cold tumors. This included loss of heterozygosity of the HLA alleles which may lead to a decreased ability to present strong binding neoantigens to the tumor cell surface and so fail to activate immune system. Another mechanism is the loss in TCR diversity in a 10 patients which may result in the T cells being unable to recognise the diverse neoantigens present. In addition the tumor micro-environment in the metastases has become immunologically quiet with enrichment of macrophages and depletion of lymphocytes compared to the primary tumor micro-environment. Our findings highlight the mechanisms that may predict response to immunotherapies and also those that can be targeted in the future in order to convert cold tumours into hot tumours.

**References**

1. Waddell N, Pajic M, Patch AM et al. Whole genomes redefine the mutational landscape of pancreatic cancer. Nature. 2015;518:495.
2. Bailey P, Chang DK, Nones K et al. Genomic analyses identify molecular subtypes of pancreatic cancer. Nature. 2016;531:47.
3. Shukla SA, Rooney MS, Rajasagi M et al. Comprehensive analysis of cancer-associated somatic mutations in class I HLA genes. Nature biotechnology. 2015;33:1152.
4. Szolek A, Schubert B, Mohr C et al. OptiType: precision HLA typing from next-generation sequencing data. Bioinformatics. 2014;30:3310-6.
5. McGranahan N, Rosenthal R, Hiley CT et al. Allele-specific HLA loss and immune escape in lung cancer evolution. Cell. 2017;171:1259-71.
6. Hundal J, Carreno BM, Petti AA et al. pVAC-Seq: A genome-guided in silico approach to identifying tumor neoantigens. Genome medicine. 2016;8:11.
7. Newman AM, Liu CL, Green MR, Gentles AJ et al. Robust enumeration of cell subsets from tissue expression profiles. Nature methods. 2015;12:453.
8. Bolotin DA, Poslavsky S, Mitrophanov I et al. MiXCR: software for comprehensive adaptive immunity profiling. Nature methods. 2015;12:380.

**Ethics Approval**

The study was approved by the QIMR Berghofer Medical Research Institute's Ethis Committee HREC (P2139) and the Hokkaido University Human Research Ethics Committee (HREC) (14-005)

#### P581 Whole-genome sequencing and multi-omic analysis of immuno-oncology biomarkers using formalin-fixed, paraffin-embedded samples

##### Shannon Bailey, PhD, Wanfeng Yu, PhD, Jim Lund, PhD, Richard Williams, Jeffrey Gulcher

###### WuXi NextCODE Genomics, Arlington, MA, USA

####### **Correspondence:** Jeffrey Gulcher (jgulcher@wuxinetcode.com)

**Background**

Next-generation sequencing analysis of archival formalin-fixed, paraffin-embedded (FFPE) tumor samples has the potential to lead to significant insights in immuno-oncology when analyzed with their accompanying rich phenotypic and pathologic data. Analysis of tumor mutation burden (TMB) using FFPE tissues has previously been restricted to estimates from exome or gene panel sequencing approaches, which provide narrow views of mutation burden. Analysis of whole-genome sequencing (WGS) data derived from FFPE samples has been limited due to challenges in isolating quality DNA from these samples and the ability to distinguish true variant calls from artifacts. Despite these challenges, WGS approaches are optimal when applied to quality tumor specimens as they provide whole-genome coverage of all regions including untranslated regions, regulatory regions, human leukocyte antigen (HLA) loci, and microsatellite regions allowing complete microsatellite instability (MSI) analysis, direct TMB calculations, and overall higher quality data for neoantigen prediction.

**Methods**

We have developed an efficient DNA extraction method (SeqPlus) that produces abundant quantities of high-quality DNA and permits robust WGS sequencing of FFPE samples. This method additionally provides improved depth and evenness of coverage for WES sequencing. SeqPlus was used to sequence primary and relapsed tumor FFPE samples from several different cancer types to perform MSI, TMB, and related analyses. Using the same tissue, we also measured mRNA expression by RNA-Seq and DNA methylation by array analysis.

**Results**

Similar to previous studies, we found that, for the majority of samples, low MSI status and low TMB correlate. We also found that, while high MSI and elevated TMB often correlate, samples with high TMB with a low or stable MSI status are more common. A majority of samples without MMR mutations have alterations in one or more genes in other DNA repair pathways. Further analysis will examine correlations between repair gene expression and mutation burden status to investigate discrepancies e.g., samples with elevated TMB and high MSI without MMR mutations. The impact of MSI and the TMB status on DNA methylation will also be examined for key genes in a global measure of genome disruption in samples

**Conclusions**

Our data demonstrate the feasibility of using WGS of FFPE samples to enable patient selection strategies for immune checkpoint inhibitor therapies. Our approach may be useful in standard clinical care or trials in the future and facilitate retrospective analysis of archival FFPE cancer tissues. This method will enhance our understanding of genomic features that respond to immuno-oncology, targeted, or conventional therapies.

#### P582 Implications of ARID1A deficiency on tumor microenvironment and immune landscape in non-small cell lung cancer (NSCLC)

##### Young Kwang Chae, MD^1^, Pedro Viveiros, MD^1^, Bhoomika Sukhadia, MD^1^, Lee Chun Park, MD^1^, Muhammad Mubbashir Sheikh, MBBS / MD^1^, Jeffrey Chuang^2^

###### ^1^Northwestern University Feinberg School of Medicine, Chicago, IL, USA; ^2^The Jackson Laboratory for Genomic Medicine, Farmington, CT, USA

####### **Correspondence:** Young Kwang Chae (young.chae@northwestern.edu)

**Background**

AT-rich interactive domain-containing gene 1A (*ARID1A*) is the most frequently mutated gene in the SWI/SNF chromatin remodeling family [1, 2], involved in transcription regulation and DNA repair. Loss of function of *ARID1A* is associated with disruption of mismatch repair [2] and poor prognosis in many solid tumors, especially gastrointestinal [3,4] and gynecological cancers [5]. Due to its tumor suppressor nature, it was believed to be a poor therapeutic target [2]. Recently, *ARID1A* deficiency was shown to be associated with increased CD8 T-cell infiltration and expression of programmed death-ligand 1 (PD-L1) in ovarian cancer [2], implying the potential of *ARID1A* as a predictor of response to immune checkpoint inhibitors (ICIs). Since the role of *ARID1A* has not been explored in NSCLC, we investigated how *ARID1A* deficiency affected tumor microenvironment and immune landscape in these patients.

**Methods**

We obtained *ARID1A* mRNA levels for NSCLC samples [Adenocarcinoma (ADC), n=517; Squamous cell carcinoma (SqCC), n= 501] from TCGA. The data was arranged into 4 quartiles based on *ARID1A* expression derived from mRNA-seq z-scores, defining the lowest quartile (Q1) as low *ARID1A* and highest quartile (Q4) as high *ARID1A*. We examined how *ARID1A* expression levels correlated with a) PD-L1 expression and b) microsatellite analysis for normal-tumor instability (MANTIS) score [6]. We also evaluated tumor mutational burden (TMB), neoantigen burden and immune landscape [7] among low and high quartiles.

**Results**

In both ADC and SqCC, analysis of immune landscape demonstrated higher infiltration of activated CD8 T-cells in the low quartile (each p<0.01, Figure 1). Lower *ARID1A* expression was associated with higher PD-L1 expression in both ADC and SqCC (each p<0.05, Figure 2A and 2B). There was a significant difference in MANTIS score between the low and high *ARID1A* quartiles in both ADC and SqCC (p<0.05, p<0.01 respectively). One sample in the low quartile of each ADC and SqCC had microsatellite instability (MANTIS>0.4), while the rest of the samples had MANTIS score in the microsatellite stable range. No correlation was found between *ARID1A* expression and TMB or neoantigen burden.

**Conclusions**

In both lung ADC and SqCC, *ARID1A* deficiency appears to influence the tumoral immune landscape. This suggests that *ARID1A* deficiency could be harnessed to select patients who may derive benefit from immunotherapy even in microsatellite stable NSCLC patients.

**References**

1. Trizzino M, Barbieri E, Petracovici A, Wu S, Welsh SA, Owens TA, Licciulli S, Zhang R, Gardini A. The tumor suppressor ARID1A controls global transcription via pausing of RNA polymerase II. Cell Rep. 2018;23(13):3933- 3945.
2. Shen J, Ju Z, Zhao W, Wang L, Peng Y, Ge Z, Nagel ZD, Zou J, Wang C, Kapoor P, Ma X, Ma D, Liang J, Song S, Liu J, Samson LD, Ajani JA, Li GM1, Liang H, Shen X, Mills GB, Peng G. ARID1A deficiency promotes mutability and potentiates therapeutic antitumor immunity unleashed by immune checkpoint blockade. Nat Med. 2018;24(5):556-562.
3. Simbolo M, Vicentini C, Ruzzenente A, Brunelli M, Conci S, Fassan M, Mafficini A, Rusev B, Corbo V, Capelli P, Bria E, Pedron S, Turri G, Lawlor RT, Tortora G, Bassi C, Guglielmi A, Scarpa A. Genetic alterations analysis in prognostic stratified groups identified TP53 and ARID1A as poor clinical performance markers in intrahepatic cholangiocarcinoma. Sci Rep. 2018;8(1):7119.
4. Kim YS, Jeong H, Choi JW, Oh HE, Lee JH. Unique characteristics of ARID1A mutation and protein level in gastric and colorectal cancer: A meta-analysis. Saudi J Gastroenterol. 2017;23(5):268-274.
5. Itamochi H, Oumi N, Oishi T, Shoji T, Fujiwara H, Sugiyama T, Suzuki M, Kigawa J, Harada T. Loss of ARID1A expression is associated with poor prognosis in patients with stage I/II clear cellcarcinoma of the ovary. Int J Clin Oncol. 2015;20(5):967-973.
6. Kautto EA, Bonneville R, Miya J, Yu L, Krook MA, Reeser JW, Roychowdhury S. Performance evaluation for rapid detection of pan-cancer microsatellite instability with MANTIS. Oncotarget. 2017;8(5):7452-7463.
7. Angelova M, Charoentong P, Hackl H, Fischer ML, Snajder R, Krogsdam AM, Waldner MJ, Bindea G, Mlecnik B, Galon J, Trajanoski Z. Characterization of the immunophenotypes and antigenomes of colorectal cancers reveals distinct tumor escape mechanisms and novel targets for immunotherapy. Genome Biology 2015;16(1):64.

**Fig. 1 (abstract P582).**
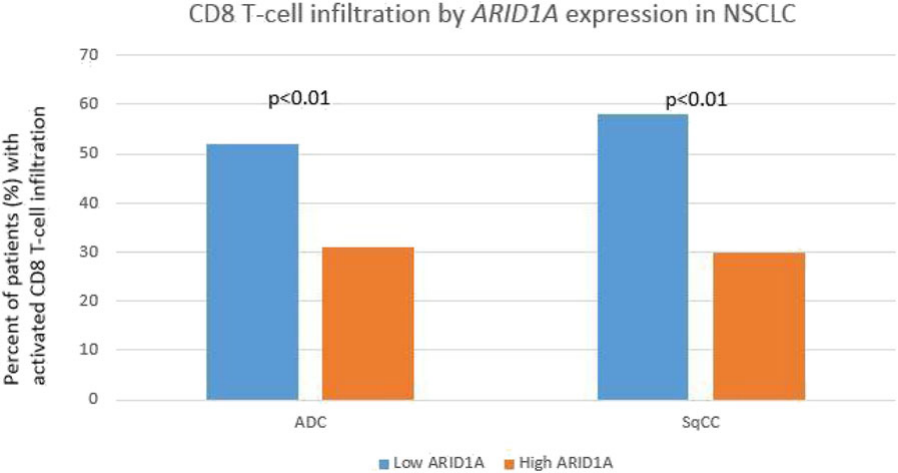
Activated CD8 T-cell infiltration by *ARID1A* expression in NSCLC samples from TCGA^

**Fig. 2 (abstract P582).**
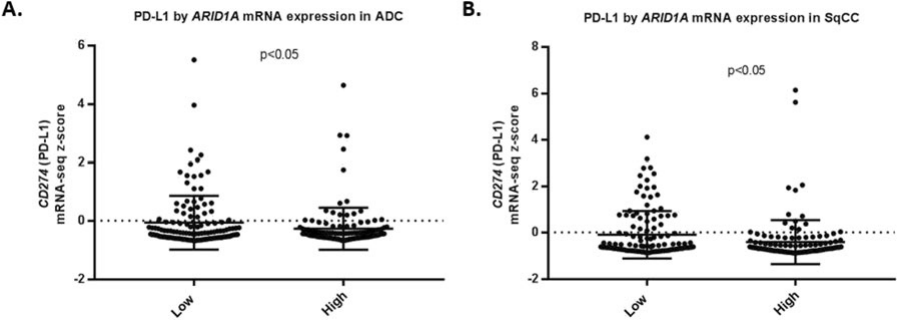
mRNA expression of *CD274* (PD-L1) by *ARID1A* expression in NSCLC samples from TCGA^+^

#### P583 Single cell transcriptional immune landscape of human papilloma virus positive and negative head and neck squamous cell carcinoma

##### Anthony Cillo, PhD, Tullia Bruno, PhD, Tracy Tabib, Zengbiao Qi, Ting Wang, Umamaheswar Duvvuri, Ryan Soose, MD, Wei Chen, Robert Lafyatis, Robert Ferris, Dario Vignali, PhD

###### University of Pittsburgh, Pittsburgh, PA, USA

####### **Correspondence:** Dario Vignali (dvignali@pitt.edu)

**Background**

Head and neck squamous cell carcinoma (HNSCC) develops through either exposure to environmental carcinogens (HPV— HNSCC), or through malignant transformation following infection with human papillomavirus (HPV+ HNSCC) [1]. Patients with HPV+ HNSCC have longer overall survival compared to patients with HPV— HNSCC [2]. We hypothesize that these differences in etiology will contribute to a spectrum of immune transcriptional signatures ranging from similar to highly divergent between these two tumor microenvironments (TMEs).

**Methods**

Paired peripheral blood mononuclear cells (PBMC) and tumor specimens were obtained from immunotherapy treatment naïve HNSCC patients. PBMC and normal tonsils were obtained from healthy donors and patients undergoing tonsillectomy as treatment for sleep apnea. Viable CD45+ cells were isolated by fluorescence based cell sorting from PBMC, tumors, and tonsils. Single-cell RNA sequencing (scRNAseq) libraries were generated using a 3’ droplet-based approach (10X Genomics). Filtered gene/barcode matrices were generated by CellRanger, and analysis was performed using the R packages SCRAN (library size deconvolution), Seurat (clustering and t- distributed stochastic neighbor embedding [tSNE]) and Destiny (diffusion-based pseudotime modeling).

**Results**

Single-cell RNAseq analysis identified a total of 57,891 single cells from 4 healthy donor PBMC, 2 tonsils, 6 paired PBMC and tumor infiltrating leukocytes (TIL) from HPV— HNSCC patients, and 5 paired PBMC/TIL from HPV+ HNSCC patients. Unbiased transcriptional analysis of TIL revealed that B cells and conventional CD4+ T cells (Tconv) had the greatest transcriptional differences between HPV+ and HPV— disease, while CD4+ regulatory T cells (Treg) were the most similar. B cells were more frequently detected in HPV+ versus HPV— disease, and B cells found in HPV+ tumors had transcriptional signatures consistent with germinal center B cells while those from HPV— tumors had memory B cell signatures. Tconv cells from HPV— HNSCC had type 1 helper signatures, while Tconv from HPV+ HNSCC expressed predominantly a T follicular helper cell signature. CD8+ T cells from HPV— HNSCC expressed higher levels of inhibitory receptors and were more terminally differentiated by diffusion pseudotime analysis. Treg cells from TIL expressed a signature associated with effector Treg cells, and this signature was consistent between HPV— and HPV+ HNSCC.

**Conclusions**

The transcriptional landscape of immune cells in HPV— versus HPV+ HNSCC differs by cell type, with B cells and CD4+ Tconv being the most divergent and CD4+ Treg the most consistent. These findings suggest that different immunotherapies may be required to achieve optimal clinical responses in these two types of HNSCC.

**References**

1. Marur S, et al. HPV-associated head and neck cancer: a virus-related cancer epidemic. Lancet Oncology. 2010 Aug;11(8):781-9.2. Fakhry C, et al. Improved survival of patients with human papillomavirus-positive head and neck squamous cell carcinoma in a prospective clinical trial. JNCI: Journal of the National Cancer Institute. 2008 Feb;100(4):261-9.

**Ethics Approval**

This study was approved by the local Institutional Review Board under protocol UPCI 99-069, and patients provided informed consent.

#### P584 High DNA repair activity is associated with immune exclusion in pediatric kidney cancers

##### Emily Higgs, BA, Ami Desai, MD, Riyue Bao, PhD, Thomas Gajewski, MD, PhD

###### University of Chicago, Chicago, IL, USA

####### **Correspondence:** Riyue Bao (rbao@bsd.uchicago.edu)

**Background**

A T-cell rich tumor microenvironment has been associated with improved clinical outcome and better response to immune checkpoint blockade therapies in several adult cancers. Our group and others have discovered mechanisms, such as β-catenin activation and PTEN loss, that drive a lack of T cell infiltration in tumor. However, much less is known about the tumor microenvironment in pediatric cancers, which harbor a lower tumor mutational burden (TMB) than most adult cancers, as well as the molecular mechanisms responsible for driving T cell exclusion in these patients. Thus, we analyzed pediatric kidney cancer data from the Therapeutically Applicable Research to Generate Effective Treatments (TARGET) database.

**Methods**

RNAseq, somatic mutations, and clinical data were obtained for Wilms tumor (WT), rhabdoid tumor (RT), osteosarcoma (OS), and neuroblastoma (NBL) from TARGET, and adult kidney cancers from TCGA. After normalization and log2-transformation, we used a 26-gene activated CD8 T cell signature [1] and identified anti- correlated genes at Pearson’s correlation r<-0.20 and FDR-adjusted P<0.05. Differentially expressed genes were detected by ANOVA at FDR-adjusted P<0.05 and fold change >2.0. Association with progression-free survival (PFS) and overall survival (OS) was assessed using Mantel-Cox test.

**Results**

Among the four pediatric cancers, we observed the lowest activated CD8 scores in WT, only detected in tumor and not in matched normal. We identified 2,128 significant genes negatively correlated with the score, 1,553 genes higher in WT compared to the adult kidney cancers, and 1,952 genes higher in WT than matched normals. There were 502 overlapping genes between these methods. Pathway analysis revealed the most activated pathways involve DNA repair. This was validated in RT. We then calculated a DNA repair expression score consisting of 4 genes (BRCA1, BRCA2, MSH2, MSH6). Within the FHWT histology where > 90% of the patients progressed, higher DNA repair score is associated with worse PFS (P=0.02), but not OS.

**Conclusions**

Our results showed that a higher DNA repair expression score is associated with lower activated T cell gene expression in childhood kidney cancers such as WT and RT, and is associated with worse survival. While loss of DNA repair pathways has previously been associated with increased neoantigens and greater response to checkpoint blockade immunotherapy, our current data suggest that upregulated DNA repair pathways may generate the opposite phenotype. Strategies targeting DNA repair pathways could be considered as new therapeutic interventions to transform non-T cell-inflamed pediatric tumors into clinically favorable tumors despite the low presence of somatic mutations.

**References**

1. Charoentong, Pornpimol, Finotello F, Angelova M, Mayer C, Efremova M, Rieder D, Hackl H, Trajanoski Z. Pan-cancer immunogenomic analyses reveal genotype-immunophenotype relationships and predictors of response to checkpoint blockade. Cell Reports. 2017; 18:248–62.

#### P585 Structured literature review and meta-analyses of the prevalence of microsatellite instability high (MSI-H) and deficient mismatch repair (dMMR) in endometrial and ovarian cancers

##### Maria Lorenzi^1^, Mayur Amonkar, PhD^2^, Jacky Zhang^1^, Shivani Mehta^1^, Kai-Li Liaw^2^

###### ^1^Precision Xtract, Oakland, CA, USA; ^2^Merck and Co., Inc., North Wales, PA, USA

####### **Correspondence:** Maria Lorenzi (maria.lorenzi@precisionxtract.com)

**Background**

Pembrolizumab has been approved in the US for the treatment of patients with unresectable or metastatic MSI- H/dMMR solid tumors that have progressed after prior treatment. There is limited and inconsistent data on the prevalence of MSI-H and dMMR across solid tumors.

**Methods**

A structured literature review covering all solid tumor types identified English language publications that used immunohistochemistry (IHC) for all four MMR proteins or polymerase chain reaction (PCR) techniques using specified NCI or Promega marker panels. MEDLINE, EMBASE, Cochrane databases and key cancer congresses were searched for relevant publications. Data were extracted on the study population, sample size, MSI-H and dMMR prevalence. For this report, we summarized the studies and performed meta-analysis (random effects model) on the prevalence of MSI-H and dMMR among endometrial and ovarian cancers. If sufficient data were available, prevalence estimates were also obtained by geography, disease stage, and histology.

**Results**

Of 1,176 citations retrieved in the larger review across all tumor types, 53 and 23 studies reported prevalence of MSI-H or dMMR in endometrial and ovarian cancers, respectively. Among endometrial cancers, MSI-H pooled prevalence (with 95% CI) from 27 studies (6,813 patents) was estimated at 26% (23-29%) and dMMR pooled prevalence from 26 studies (5,248 patients) was estimated at 25% (22-28%). In ovarian cancers, MSI-H pooled prevalence from 17 studies (4,150 patients) was estimated at 11% (6-18%) and dMMR pooled prevalence from 5 studies (356 patients) was estimated at 8% (6-11%). Based on histology, the highest MSI-H pooled prevalence was observed for endometrioid subtype for each tumor with 30% (25-35%) based on 6 studies (1,204 patients) for endometrial cancers and 17% (25-35%) based on 3 studies (211 patients) for ovarian cancers. In both cancer types, pooled prevalence was further explored by geography and disease stage.

**Conclusions**

This comprehensive literature review provides pooled prevalence estimates of MSI-H and dMMR across two key gynecological tumors based on published data. The pooled prevalence estimates of MSI-H among endometrial and ovarian cancers were similar to the corresponding pooled prevalence estimates for dMMR for these tumors.

#### P586 Frameshift indel selectively correlates with immunotherapy outcome for advanced NSCLC

##### Wungki Park, MD^1^, Lee Chun Park, MD^2^, Vaia Florou, MD^3^, Diana Saravia, MD^3^, Sangmin Chang, MD^2^, Si Wang, MD^2^, Lauren Chiec^2^, Ashkon Rahbari^2^, Pedro Viveiros, MD^2^, Bhoomika Sukhadia, MD^2^, Muhammad Mubbashir Sheikh, MBBS / MD^2^, Nisha Mohindra^2^, Victoria Villaflor, MD^2^, Gilberto Lopes, MD, MBA^3^, Young Kwang Chae, MD^2^, Wungki Park, MD^1^

###### ^1^Memorial Sloan Kettering Cancer Center, New York, NY, USA; ^2^Northwestern University, Chicago, IL, USA; ^3^University of Miami, Miami, FL, USA

####### **Correspondence:** Young Kwang Chae (young.chae@northwestern.edu)

**Background**

Frameshift insertion-deletion (fsindel) was suggested as more immunogenic type of mutation associating with higher tumor-specific neoantigens which also correlated with clinical outcome of immunotherapy in melanoma and renal cell carcinoma patients[1]. Our group recently demonstrated that the presence of fsindel is also relevant in NSCLC patients treated with PD-1/L1 inhibitors-based immune checkpoint inhibitors (ICIs) independently from their Tumor Mutational Burden (TMB) [2]. Also, we showed higher fsindel burden was associated with higher activated CD4 and CD8 T cell RNA signatures and antigen presentation signature from The Cancer Genome Atlas (TCGA) database. Yet, whether this favorable outcome association in fsindel-present NSCLC patients is specifically for immunotherapy only or other treatments is still unknown.

**Methods**

A retrospective analysis was performed from 122 advanced NSCLC patients treated with ICIs from Northwestern University (N=62) and the University of Miami (N=60). The presence or absence of fsindel and tumor mutation burden (TMB) were determined from 324-gene sequencing by FoundationOneTM (F1). Progression free survivals (PFS) of fsindel-present and -absent patients during ICIs and during their first-line chemotherapy (1L Chemo, n=89) were compared.

**Results**

Fsindel-present advanced NSCLC patients treated with ICIs had significantly more favorable outcome with median PFS of 6.2 months vs. 2.7 months (Hazard Ratio [HR], 0.59; 95% Confidence Interval [CI], 0.38 to 0.90 (Figure 1). Importantly, this finding was specific to ICIs and there was no difference observed in 1L Chemo (HR, 1.02; 95% CI, 0.67 to 1.54 (Figure 2).

**Conclusions**

Fsindel may serve as a novel predictive biomarker strategy specifically for immunotherapy independent of TMB, but not for chemotherapy. Future prospective clinical data analysis and immune monitoring assays may validate this hypothesis further. Further exploration on pancancer landscape of TMB and fsindel is underway.

**References**

1. Turajlic S, Litchfield K, Rosenthal R. Insertion-and-deletion-derived tumour-specific neoantigens and the immunogenic phenotype: a pan-cancer analysis. Lancet Oncol, 2017; 8:1009-1021.
2. Hellmann M, Ciuleanu T, Pluzanski A. Nivolumab plus ipilimumab in lung cancer with a high tumor mutational burden. N Engl J Med, 2018; 22: 2093-2104.

**Ethics Approval**

The study was approved by Institution's Ethics Board at University of Miami, approval number ePROST# 20170427.

**Fig. 1 (abstract P586).**
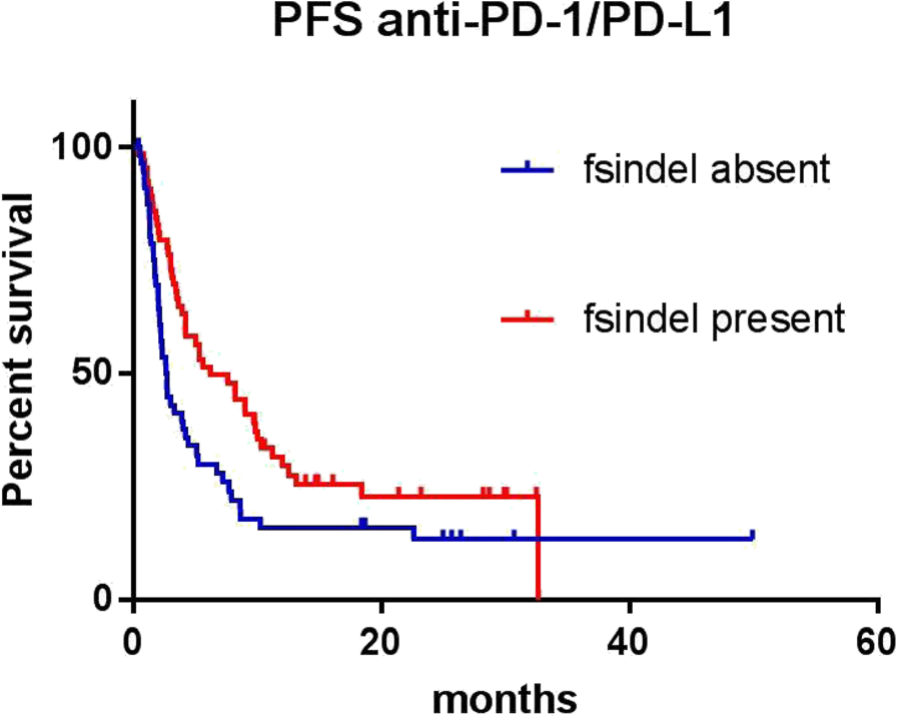
See text for description

**Fig. 2 (abstract P586).**
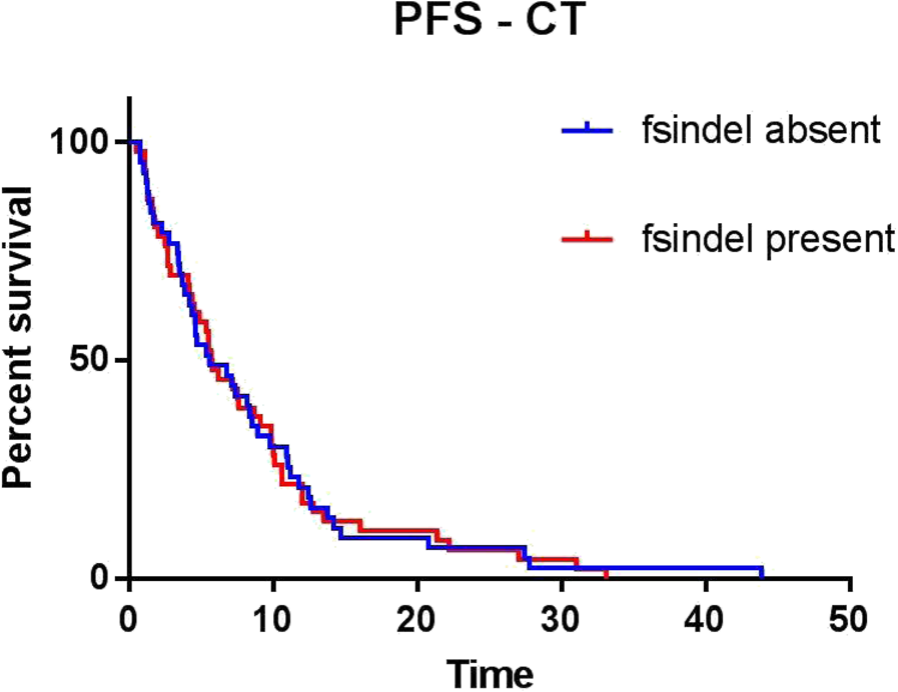
See text for description

#### P587 Conditional activation of immune-related pathways and prognostic significance: a pan cancer analysis

##### Jessica Roelands, Master^1^, Michele Ceccarelli^2^, Darawan Rinchai, PhD^3^, Sara Pai, MD, PhD^4^, Francesco Marincola, MD^5^, Lance Miller, MS, PhD^6^, Peter Kuppen^7^, Davide Bedognetti, MD, PhD^3^, Wouter Hendrickx, PhD^3^

###### ^1^1. Sidra Medicine; 2. Leiden University Medical Center, Doha, Qatar; ^2^AbbVie Inc., Benevento, Italy; ^3^Sidra Medicine, Doha, Qatar; ^4^Massachusetts General Hospital, Boston, MA, USA; ^5^Refuge Biotechnologies, Half Moon Bay, CA, USA; ^6^Wake Forest School of Medicine, Winston-Salem, NC, USA; ^7^Leiden University Medical Center, Leiden, Zuid-Holland, Netherlands

####### **Correspondence:** Davide Bedognetti (dbedognetti@sidra.org)

**Background**

It has been proposed that cancers can be divided in opposite phenotypes according to their immune orientation. This immune orientation influences response to therapy and clinical prognosis. The previously described Immunologic Constant of Rejection (ICR) signature is used here to define two opposite immune phenotypes (i.e., immune-active and immune-silent) across 31 different histologies. The relationship between tumor genetic makeup and immune orientation only recently began to be elucidated. In this pan-cancer study, we systematically analyzed the interconnection between tumoral genetic programs and immune orientation, and the prognostic impact of this interplay.

**Methods**

RNA-seq data of samples from a total of 9282 patients across 31 cancer types were obtained from TCGA. Additionally, RNA and DNA was isolated from fresh frozen tissue samples of an internal cohort of 366 colon cancer patients from LUMC. Exome and RNA sequencing was performed at Sidra. TCGA- and internal RNA-seq (HiSeq4000) data were normalized. Exome sequencing for our internal cohort is currently ongoing. We performed unsupervised consensus clustering for each cancer type separately based on the expression of the ICR gene signature (Figure 1A-E)[2,3]. Oncogenic pathway gene set enrichment and mutational status were analyzed in relation to ICR phenotypes. To explore whether tumor intrinsic attributes associate with the prognostic value of ICR across cancers, we compared mutational load, oncogenic alterations and expression of oncogenic pathways between cancer types.

**Results**

Our analysis identified a distinct prognostic connotation of ICR depending on cancer type. We confirmed a positive impact of ICR gene expression in our colon cancer cohort (Figure 1D). We identified several oncogenic pathways whose enrichment inversely correlated with ICR (Figure 2) in multiple tumor types. Such alterations include novel pathways as well as pathways previously described to influence immune disposition in specific tumor types. In addition, mutations in specific genes were associated with ICR (Figure 3). Interestingly, we found various pathways associated with cancer-cell intrinsic features that were differentially enriched between tumors in which ICR had a prognostic impact versus the ones in which ICR did not bear any prognostic connotation.

**Conclusions**

We identified tumor-intrinsic attributes that correlated with immune phenotypes and potentially influence their development. In addition, a relation was observed between the enrichment of oncogenic pathways and the prognostic significance of the ICR. Such information can be used to prioritize potential candidates for immunogenic conversion and to refine stratification algorithms. A validation of the TCGA results is ongoing through the analysis of the aforementioned internal cohort.

**Acknowledgements**

The authors would like to acknowledge the support from Qatar Foundation, Qatar National Research Fund (grant number: JSREP07-010-3-005).

**References**

1. Turan T, Kannan D, Patel M, Matthew Barnes J, Tanlimco SG, Lu R, et al. Immune oncology, immune responsiveness and the theory of everything. Journal for ImmunoTherapy of Cancer. 2018 Jun 5;6(1):50.
2. Hendrickx W, Simeone I, Anjum S, Mokrab Y, Bertucci F, Finetti P, et al. Identification of genetic determinants of breast cancer immune phenotypes by integrative genome-scale analysis. OncoImmunology. 2017 Feb 6;0.
3. Bedognetti D, Hendrickx W, Ceccarelli M, Miller LD, Seliger B. Disentangling the relationship between tumor genetic programs and immune responsiveness. Current Opinion in Immunology. 2016 Apr;39:150–8.

**Ethics Approval**

Samples were used for research according to the code of good conduct regarding secondary use of human tissue as described in “Human Tissue and Medical Research: Code of Conduct for responsible use (2011)” Drawn up by the FEDERA (Netherlands).

**Fig. 1 (abstract P587).**
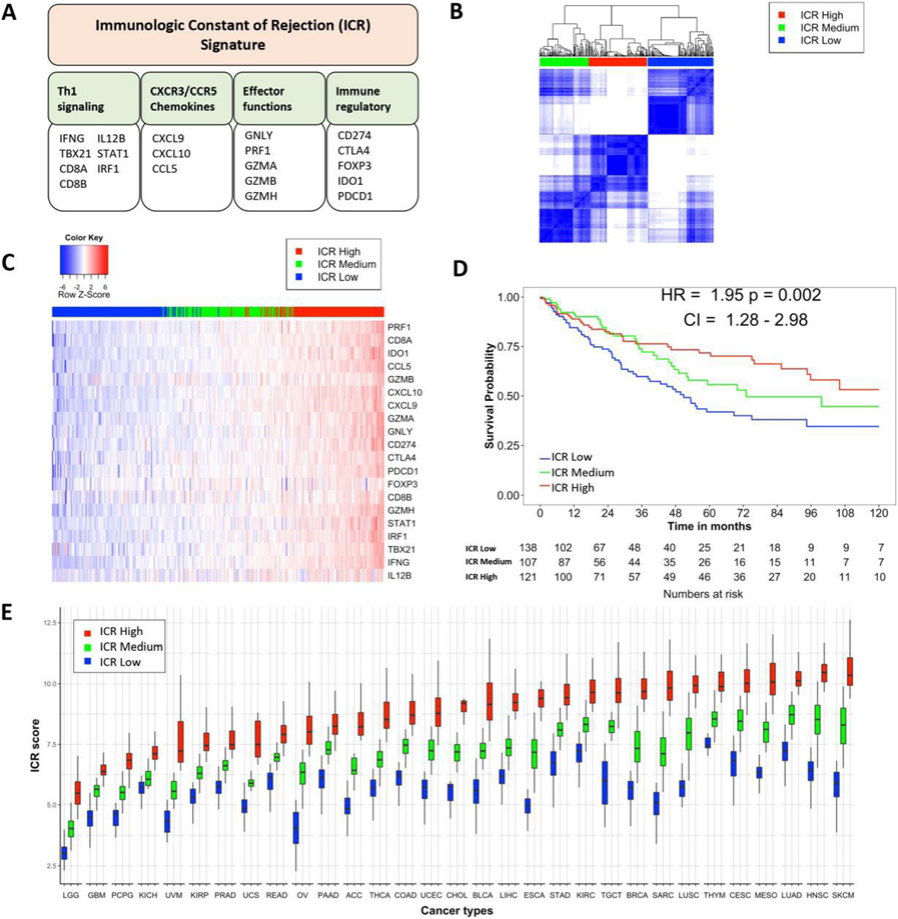
See text for description

**Fig. 2 (abstract P587).**
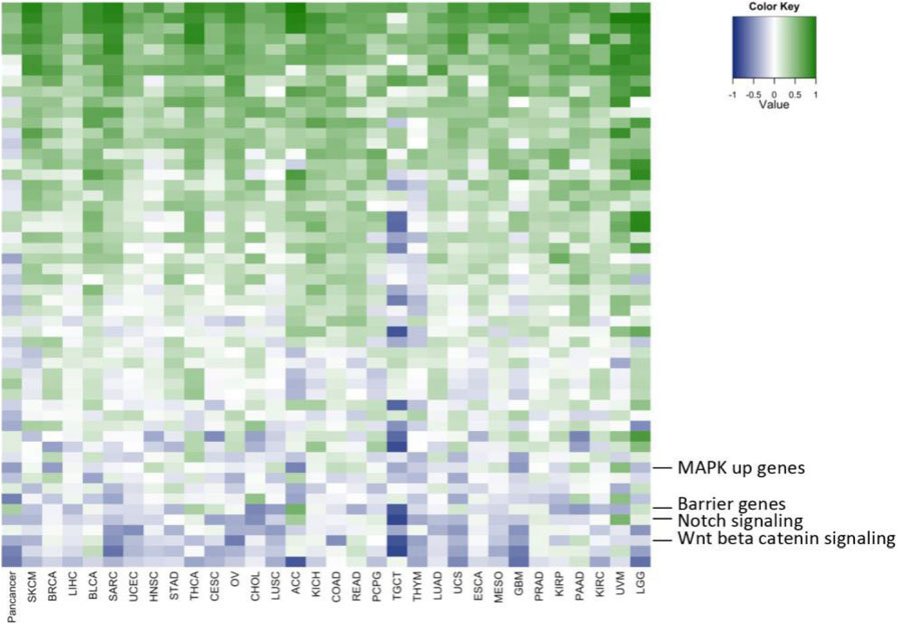
See text for description

**Fig. 3 (abstract P587).**
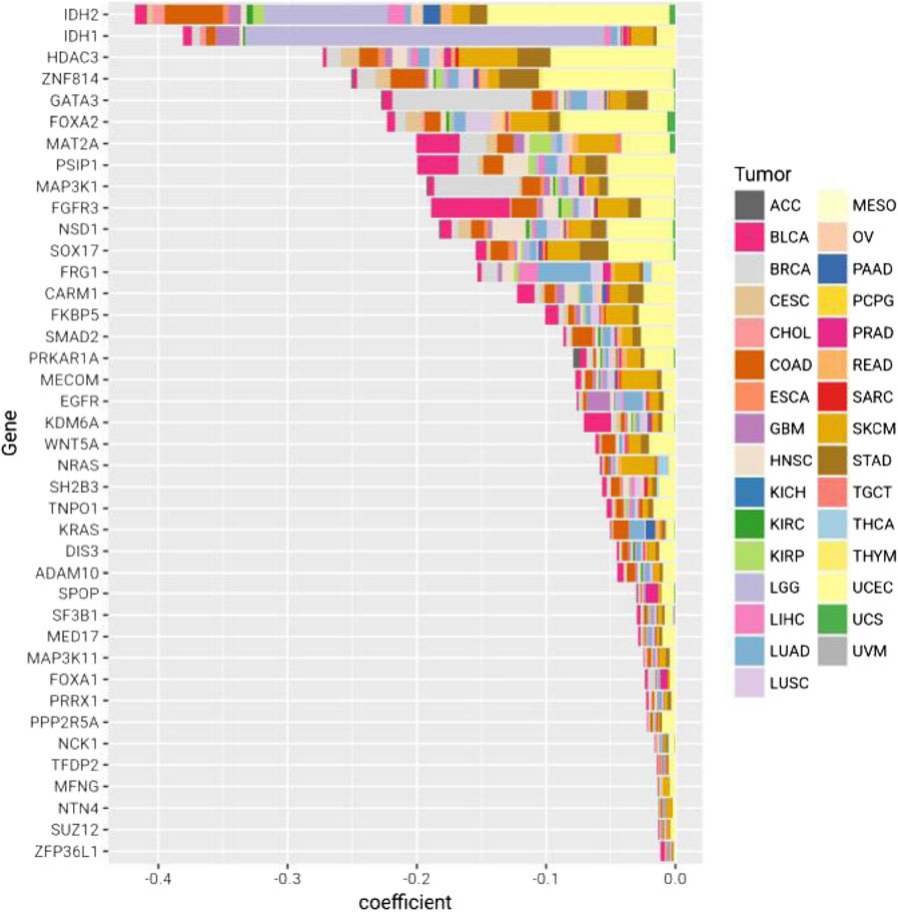
See text for description

#### P588 Deep learning of the immune synapse

##### John-William Sidhom, MSE, Drew Pardoll, MD, PhD, Alexander Baras

###### Johns Hopkins University, Baltimore, MD, USA

####### **Correspondence:** John-William Sidhom (jsidhom1@jhmi.edu)

**Background**

Artificial intelligence is poised to revolutionize every aspect of human life, finding applications in everything from self-driving cars to diagnosing cancer. In fact, almost any task that involves pattern recognition can be formulated in a way that modern AI algorithms can be used to achieve super-human performance. The immune synapse is a highly complex interaction between several proteins and peptides that allows for a constant surveillance of foreign invaders. However, modeling these interactions is extremely difficult as the combinations of interactions is simply intractable. In immuno-oncology, the study of this interaction is crucial as anti-tumor responses rely on sensitive and specific recognition of tumor-specific antigens. Implications of accurately predicting and modeling these interactions in immune-oncology range from improved and potent vaccine design to biomarkers for predicting response to immunotherapy.

**Methods**

Our group has developed a variety of deep learning models to model the signal transmission within the immune synapse. At the core of all architectures designed, convolutional layers, similar to ones used to learn features in images, are used to learn motifs within the sequencing data for a predictive or descriptive purpose.

**Results**

We first present AI-MHC, an applied deep convolutional neural network for class-specific MHC binding algorithm that achieves state-of-the-art performance in both Class and Class II predictions. By incorporating ‘meaning’ of the allele within the network, we are able to model the interaction of allele and peptide within the context of a neural network (Figure 1)[1]. We take these concepts further in the development of DeepMANA, a deep learning framework which combines sequence-specific information about an allele/peptide pairing to not only predict binding affinity for any allele with a known protein sequence but also provide an antigen ‘quality’ score, based on the “non- self/foreign-ness” of a neoantigen. We observe in three previously published immunotherapy clinical trials, these quality neoantigens are enriched in long-term survivors or responders (Figure 2)[2]. Finally, we present DeepTCR, a collection of unsupervised and supervised deep learning algorithms capable of revealing structure in T-cell receptor repertoire. We demonstrate that DeepTCR achieves state-of-the-art performance in clustering antigen-specific TCR’s (Figure 3A). and is capable of learning a predictive signature in TIL repertoire of mice treated with various immunotherapies (Figure 3B)[3,4].

**Conclusions**

These types of AI technologies could yield an entire new area of biomarker discovery as well as improve our understanding of the complex interaction occurring at the immune synapse that is ultimately required for a successful anti-tumor response.

**References**

1. Sidhom, J.-W., Pardoll, D. & Baras, A. AI-MHC: an allele-integrated deep learning framework for improving Class I & Class II HLA-binding predictions. bioRxiv 318881 (2018). doi:10.1101/318881
2. Łuksza, M. et al. A neoantigen fitness model predicts tumour response to checkpoint blockade immunotherapy. Nature (2017). doi:10.1038/nature24473
3. Glanville, J., Huang, H., Nau, A., Hatton, O. & Nature, W. L. Identifying specificity groups in the T cell receptor repertoire. (2017). 4. Rudqvist, N., Pilones, K. & Immunol …, L. C. Radiotherapy and CTLA-4 blockade shape the TCR repertoire of tumor-infiltrating T cells. (2018).

**Fig. 1 (abstract P588).**
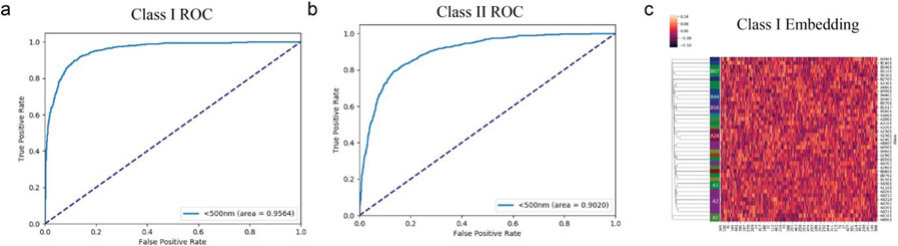
AI-MHC

**Fig. 2 (abstract P588).**
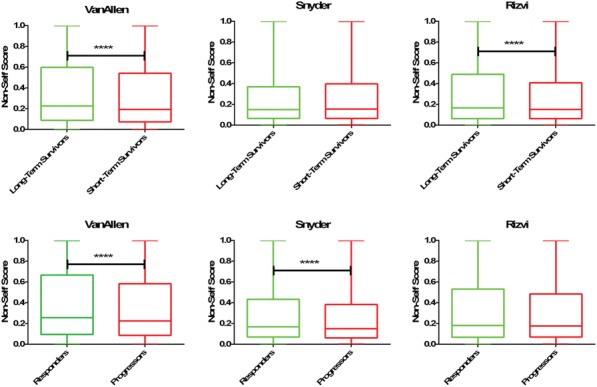
DeepMANA

**Fig. 3 (abstract P588).**
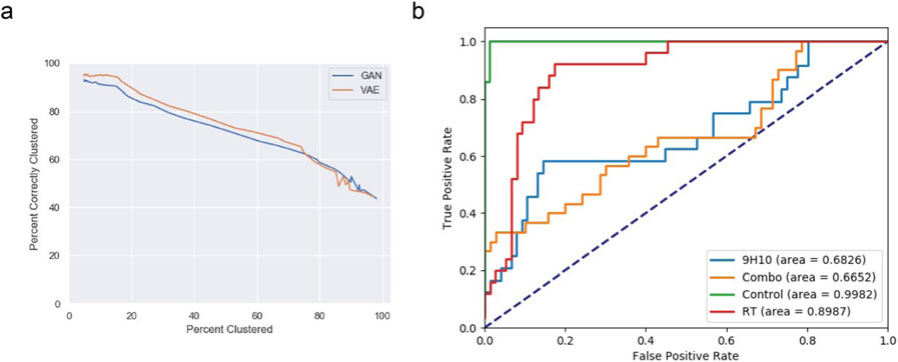
DeepTCR

#### P589 Endogenous oncogene mutation-specific T cell responses in patients with clinical response to PD-1 blockade

##### Kellie Smith, PhD, Nicolas Llosa, MD, Valsamo Anagnostou, MD PhD, Hok Yee Chan, MS, Jiajia Zhang, MD, MPH, Haidan Guo, BS, and BA, Tricia Cottrell, MD, PhD, Jarushka Naidoo, MD, Kristen Marrone, MD, Janis Taube, MD, MSC, Victor Velculescu, MD, PhD, Julie Brahmer, MD, Patrick Forde, MD, Drew Pardoll, MD, PhD, Franck Housseau, PhD

###### Johns Hopkins School of Medicine, Baltimore, MD, USA

####### **Correspondence:** ksmit228@jhmi.edu

**Background**

High tumor mutational burden (TMB) is associated with objective clinical response to checkpoint blockade. We and others have shown that in patients with high TMB, negatively-regulated T cells specific for mutation-associated neoantigens are unleashed upon treatment and may facilitate tumor regression [1-3]. However, some cancers without high mutational burden do respond to anti-PD-1. Understanding the basis for these responses in the absence of high TMB and, consequently, immunogenic neoantigens, will provide potential biomarkers for therapeutic guidance and may provide insights into improving immunotherapy outcomes in patients with low TMB.

**Methods**

Whole exome sequencing was performed on matched tumor/normal tissue from patients receiving checkpoint blockade. The patients spanned multiple tumor types. Putative neoantigens, including those derived from oncogenic driver mutations, were selected for experimental testing based on predicted MHC class I affinity and expression of the mutated gene as reported by TCGA. Neoantigen recognition was evaluated using MANAFEST [4], followed by tracking of neoantigen-specific T-cells in tumor and serial blood using quantitative sequencing of the T cell receptor V-beta CDR3 (TCRseq). When fresh tumor was available, TCRseq was performed on tumor infiltrating lymphocytes pre-sorted based on PD-1 expression.

**Results**

Three patients with low TMB tumors who derived durable clinical benefit demonstrated broad endogenous neoantigen-specific T cell responses. Two of these patients, one with NSCLC and one with a neuroendocrine tumor, exhibited persistent memory T cell responses to neoantigens derived from mutations in the oncogenic BRAF and tumor-suppressor PTCH1 genes, respectively. Additionally, while not considered low TMB, a patient with mismatch repair proficient CRC demonstrated recognition of a neoantigen derived from the hotspot AKT1 E17K mutation.

These neoantigens were validated using in vitro binding and stability assays. Analyses of the peripheral dynamics of T-cell clonotypes sorted by differential PD-1 expression are ongoing.

**Conclusions**

These findings suggest that oncogenic driver mutations are recognized by T-cells more often than previously appreciated. Responses to these neoantigens may be particularly efficacious in low TMB tumors owing to the likelihood that oncogenic mutations are required for tumor survival and are less likely to be eliminated. Sensitive bioassays, such as the MANAFEST assay used here, may identify patients that would not otherwise be predicted to respond to checkpoint blockade based on current biomarkers, such as high mutational burden or mismatch repair status. In patients without endogenous T cell recognition, identifying T cell clonotypes specific for these mutations provides the foundation for vaccines or T cell therapies targeting oncogene mutation-derived neoantigens.

**References**

1. Forde PM, Chaft JE, Smith KN, Anagnostou V, Cottrell TR, Hellmann MD, Zahurak M, Yang SC, Jones DR, Broderick S, Battafarano RJ, Velez MJ, Rekhtman N, Olah Z, Naidoo J, Marrone KA, Verde F, Guo H, Zhang J, Caushi JX, Chan HY, Sidhom J, Scharpf RB, White J, Gabrielson E, Wang H, Rosner GL, Rusch V, Wolchok JD, Merghoub T, Taube JM, Velculescu VE, Topalian SL, Brahmer JR, Pardoll DM. Neoadjuvant PD-1 blockade in resectable lung cancer. NEJM. 2018; 378(21): 1976-1986.
2. Le DT, Durham JN, Smith KN, Wang H, Barlett BR, Aulakh LK, Lu S, Kemberling H, Wilt C, Luber BS, Wong F, Azad NS, Rucki AA, Laheru D, Donehower R, Zaheer A, Fisher GA, Crocenzi TS, Lee JJ, Greten TF, Duffy AG, Ciombor KK, Eyring AD, Lam BH, Joe A, Kang SP, Holdhoff M, Danilova L, Cope L, Meyer C, Zhou S, Goldberg RM, Armstrong DK, Bever KM, Fader AN, Taube J, Housseau F, Spetzler D, Xiao N, Pardoll DM, Papadoploulos N, Kinzler KW, Eshleman JR, Vogelstein B, Anders RA, Diaz LA Jr. Mismatch repair deficiency predicts response of solid tumors to PD-1 blockade. Science. 2017; 357(6349):409-413.
3. Rizvi NA, Hellmann MD, Snyder A, Kvistborg P, Makarov V, Havel JJ, Lee W, Yuan J, Wong P, Ho TS, Miller ML, Rekhtman N, Moreira AL, Ibrahim F, Bruggeman C, Gasmi B, Zappasodi R, Maeda Y, Sander C, Garon EB, Merghoub T, Wolchok JD, Schumacher TN, Chan TA. Mutational landscape determines sensitivity to PD-1 blockade in non-small cell lung cancer. Science. 2015; 348(6230): 124-8.
4. Danilova L, Anagnostou V, Caushi JX, Sidhom J, Guo H, Chan HY, Suri P, Tam A, Zhang J, El Asmar M, Marrone KA, Naidoo J, Brahmer JR, Forde PM, Baras AS, Cope L, Velculescu VE, Pardoll DM, Housseau F, Smith KN. The mutation associated neoantigen functional expansion of specific T cell receptor clonotypes (MANAFEST) assay: a sensitive platform for monitoring anti-tumor immunity. Cancer Immunol Res. 2018; 6(8): 1-12.

**Ethics Approval**

This study was approved by the Johns Hopkins University Institutional Review Board, approval numbers IRB00100653 and NA_00090257.

#### P590 Evaluation of whole exome sequencing for quantitative immune cell type deconvolution in tumor for clinical application in oncology

##### Alex So, PhD, Joyee Yao, BA, Aaron Wise, PhD, Kevin Wu, Kristina Kruglyak, PhD, Sven Biike, Traci Pawlowski, PhD, Shile Zhang, PhD

###### Illumina, San Diego, CA, USA

####### **Correspondence:** Alex So (aso@illumina.com)

**Background**

The rise of cancer immunotherapy has led to increasing interest in detecting the presence of immune cells in the tumor microenvironment. Along with tumor mutation burden (TMB) and microsatellite instability (MSI) status, recent studies have also demonstrated that the level of immune cells in the tumor microenvironment correlates with patient responsiveness to immunotherapy. Here, we present our work demonstrating the feasibility of quantifying immune cells in analytical titration samples and real dissociated tumors from bulk RNA sequencing using an in- house developed immune cell deconvolution algorithm (FRICTION).

**Methods**

Libraries were generated using Illumina’s TruSeq™ RNA Exome. The samples were paired-end sequenced (2x75bp) using the Illumina HiSeq™ 2500 Rapid Run mode. RNA purified from immune cells (CD4+, CD8+, and CD19+ cells) were titrated into RNA from various tissue backgrounds and subjected to RNA sequencing and FRICTION analysis. The fraction of immune cells present in cryopreserved dissociated melanoma tumors were quantified through flow cytometry or RNA sequencing and analyzed with FRICTION.

**Results**

In preliminary testing, immune cell line titration experiments with various percentages of immune cell mixtures added into different tissue backgrounds demonstrated our method’s linearity in quantifying these cells (median R2 > 0.98). The titration experiments were performed by spiking in immune cell RNA into various tissues background at the RNA level. Then, we analyzed cryopreserved dissociated melanoma tumors (n=38) with flow cytometry to determine the percentage of immune cells present and TruSeq™ RNA Exome for immune cell signatures by FRICTION. Our data demonstrated that we can quantify the fractions of CD4+ T cells, CD8+ T cells, and CD19+ B cells presence corresponding to flow cytometry data on each subset of immune cells. Finally, we correlated inflamed tumors, or those with high immune cell content, by FRICTION to CD45+ immune cells from flow cytometry with a linearity of R2 > 0.82.

**Conclusions**

In summary, we demonstrate that the TruSeq™ RNA Exome next-generation sequencing workflow combined with FRICTION, an immune cell deconvolution algorithm, can predict the presence of CD4+ T cells, CD8+ T cells, and CD19+ B cells within tumor microenvironment in a quantitative manner and accurately determine the inflammation of the tumor microenvironment.

#### P591 Somatic alterations in PD-L1 predict response to platinum-based chemotherapy in patients with advanced prostate cancer

##### Panagiotis Vlachostergios, MD, PhD, Aileen Lee, Charlene Thomas, Priyanka Patel, Amy Hackett, Naureen Rashid, Ana Molina, MD, David Nanus, MD, Himisha Beltran, MD, Scott Tawaga, MD

###### Weill Cornell Medicine, New York, NY, USA

####### **Correspondence:** Scott Tawaga (stt2007@med.cornell.edu)

**Background**

The interplay between malignant tumors and the immune system is becoming increasingly understood. Immune checkpoint inhibitors are approved for treatment of several cancer types, with PD-L1 immunohistochemical expression as a companion test in many cases. Immune checkpoint blockade is not an established therapy for advanced prostate cancer (PC) patients (castration-resistant or neuroendocrine PC); however platinum agents are active and in clinical use. This study aimed to assess the impact of somatic alterations in PD-L1 on clinical responses to platinum chemotherapy in patients with advanced PC.

**Methods**

We reviewed records of advanced PC patients enrolled in our Precision Medicine cohort, who received platinum-based chemotherapy with available tumor tissue specimens and clinical information of known prognostic factors. We used whole exome sequencing (WES) to assess for mutations and copy number alterations in the CD274 gene (encoding PD-L1). We used Kaplan Meier curves, univariable and multivariable Cox regression analyses to predict time to PSA progression-free survival (PSA-PFS), radiographic progression-free (rPFS) and overall survival (OS) after initiation of platinum-based chemotherapy.

**Results**

Our cohort included 31 men, median age 69 years (range 50-85). Based on histological features 8 patients (26%) were NEPC. Twenty five patients had bone metastases and 19 had visceral metastases (16 liver, 11 lung, 1 brain).

The majority or patients (26/31) received carboplatin, 8 received cisplatin and 4 received both sequentially, with initial platinum used for data analysis. Most patients received chemotherapy doublets, and platinum was most frequently combined with paclitaxel (N=11) and etoposide (N=12). Somatic alterations (mutations or/and copy number changes) in CD274 (encoding for PD-L1) were associated with a significantly longer rPFS compared to men with wild-type PD-L1 (median rPFS: (median rPFS: 8 versus 4 months, P=0.022). PD-L1 alterations were less frequent observed in men with bone metastases (2/22 vs 4/9, P=0.043). No significant correlations were identified between CD274 status (wild-type versus mutations/copy number alterations) and PSA-PFS or OS. On multivariate analysis (adjusted for Gleason score, PSA, alkaline phosphatase, lactate dehydrogenase, hemoglobin, visceral metastases, performance status, use of opioids), PSA (P=0.049) and the presence of visceral metastases (P=0.048) were independent prognosticators of OS.

**Conclusions**

Our study suggests that somatic alterations in PD-L1 may predict radiologic responses in patients with advanced PC treated with platinum-based chemotherapy. Validation of these findings in larger prospective studies are warranted.

**Ethics Approval**

The patient consented to participate in the Precision Medicine protocol at Weill-Cornell Medicine (WCM). The study was approved by our Institutional Review Board and Ethics Committee (WCM / New York-Presbyterian IRB protocol #: 1305013903).

#### P592 Clonality of tumor infiltrating and peri-tumoral lymphocytes in colorectal cancers with high-microsatellite instability

##### Pamela Ward, PhD, Mihaela Campan, PhD, Katherine Scribner, DO, Ashley Hagiya, MD, Cristina Costales, MD, Michael Bask, BS, Tiffany Long, BS, Afsaneh Barzi, MD, Jonas Pettersson, PhD, Louis Dubeau, MD PhD

###### University of Southern California, Los Angeles, CA, USA

####### **Correspondence:** Pamela Ward (pamela.ward@med.usc.edu)

**Background**

Immune checkpoint inhibitor therapy is approved for colorectal cancers that show high microsatellite instability. These cancers typically show increased mutational burden associated with increased T cell infiltration correlating with higher Immunoscores. Not all these tumors respond to immunotherapy, hence a need for additional predictors of responsiveness. We sought to quantitate tumor infiltrating and peritumoral lymphocytes in patients with colorectal cancers displaying high-microsatellite instability and examine the clonality of their expression profiles based on the hypothesis that these parameters are predictors of such responsiveness.

**Methods**

T cells of interest were identified by immunostains against CD3 and CD8 in 4-micron thick formalin-fixed, paraffin-embedded tissue sections from 4 colorectal cancers with high microsatellite instability including 2 from patients with Lynch syndrome and 2 from sporadic cancers. The average number of immunopositive tumor infiltrating and peritumoral lymphocytes and the ratio of CD8 over CD3 positive cells in 10 high power fields (totaling approximately 3 square millimeters) were scored independently by two pathologists. Areas of at least 5 square millimeters containing representative tumor infiltrating and peritumoral lymphocytes were macrodissected and subjected to RNA extraction using a Promega Maxwell instrument. The Archer Immunoverse NGS TCR assay and an Illumina Miseq instrument were used to sequence 400ng RNA from each sample. Results were analyzed using Archer’s analysis pipeline.

**Results**

All tumors had over 100 tumor infiltrating lymphocytes per high power field, 63% showing CD8 positivity. There was a range of 10-100 peritumoral lymphocytes per high power field, 75% with CD8 positivity. The two cases associated with sporadic cancer had 234 and 338 unique TCR-beta sequences respectively, with a single dominant clone (44% of total sequences) in one case and two dominant clones (15% and 16% of total sequences) in the other. The two Lynch syndrome cases had 665 and 1442 unique receptor sequences respectively in infiltrating lymphocytes, none above background frequency. Peritumoral lymphocytes in 3 cases showed from 1394 to 2986 unique T cell receptor beta sequences, none of which were dominant. The remaining case, from a patient with sporadic cancer, had 438 unique sequences, one representing 21% of all sequences, but the selected region contained a small number of admixed tumor cells.

**Conclusions**

T-cell clonality in tumor infiltrating lymphocytes was detectable and associated with sporadic tumors in this small cohort of colorectal cancers with high microsatellite instability. Whether or not this can predict responsiveness to immunotherapy merits investigation.

**Ethics Approval**

This study was approved by USC Institutional Review Board; approval number HS-18-00285.

#### P593 Robust TMB values calculated from tumor-only material show correlation and precision with paired results

##### Victor Weigman, Natalie Mola, MS

###### EA Genomics, Q Squared Solutions

####### **Correspondence:** Victor Weigman (victor.weigman@q2labsolutions.com)

**Background**

Tumor mutational burden (TMB), a measure of the number of somatic mutations per Mb of the assay target region, is becoming a common biomarker in prediction of immunotherapy response. As the tumor evolves, mutations are accumulated, leading to growth advantages and opportunities for presentation of new neoantigens. Immunotherapies designed to antagonize common checkpoint inhibitors like PD1/PD-L1 and CTLA4 correct for this immune evasion and mutational burden provides a continuous variable that’s prognostic to this attainment. This is also showing promise in 2nd and 3rd line inhibitory compounds (TIM-3, LAG3, IDO1) and co-stimulatory antibodies (OX40, GITR, CD40).Calculation of TMB is dependent on calling tumor-derived (somatic) mutations that are derived from identifying variants in the tumor cells and contrasting them against variants in a matched normal/germline sample. However, this approach increases costs of testing and in many cases, may not be possible if a normal sample cannot be obtained, consented or absent from a clinical trial protocol for TMB testing.

**Methods**

Our tumor-only TMB pipeline uses somatic classifications determined using a random forest model. Utilizing TCGA samples, the model was trained across multiple indications using variant classifications (somatic/germline) produced by our original paired TMB analysis pipeline. The pan-cancer model consists of 236 TCGA samples from 5 separate indications that have been shown to have a range of median TMB values. This model has 17 significant predictors (like variant allele frequency, ExAC frequency, SIFT score, etc) derived from variant call metrics and database annotations pulled from GoldenHelix Varseq.

**Results**

Utilizing data from TCGA samples from indications not included in our model we looked to compare tumor-only TMB using correlation to known values, precision in biological replicates, preservation of dynamic range and linearity. Using indications outside of our original model,, we observed slopes ranging from 0.72-1.1 and R2 values consistently above 0.92 against our paired values. We observed similarly high correlation (slope 0.92/R2 0.87) in paired values generated from TCGA. Utilizing procured case-matched fresh frozen and FFPE samples and showed TMB from FFPE ranging within 30% of the frozen values. When testing replicates for both frozen and FFPE, we consistently observed TMB scores under 10% in frozen and ranging from 1-21% in FFPE.

**Conclusions**

To increase applicability of TMB to the volume of retrospective studies, calculation must be able to be performed by using only the tumor specimen and we feel our approach provides robust calculation across use cases.

#### P594 Dynamic analysis and visualization of the interactions between tumor and the immune cell infiltration by integrating TCGA genomic- and transcriptional- data

##### Mingchao Xie, PhD, Bolan Linghu, Pei Zhang, PhD, Zhongwu Lai, PhD, Jonathan Dry, Benjamin Sidders

###### Astrazeneca, Waltham, MA, USA

####### **Correspondence:** Jonathan Dry (Jonathan.Dry@astrazeneca.com)

**Background**

Investigation of the interactions between the tumor genomic landscape and immune cell infiltration is critical for immune-oncology (IO) projects. Such analysis facilitates the discovery of novel prognostic biomarkers, identification of new drug targets, and understanding of drug resistance mechanisms. However, due to a lack easily available datasets and proper analysis tools, systematically exploring the tumor–immune interaction is a big challenge.

**Methods**

Here, we deconvoluted the immune cell compositions of 9,721 primary tumor samples from 33 TCGA cancer types using transcriptomic data, and developed a web-based application, IO Browser.

**Results**

The browser allows the user to visualize the immune composition of a sample or cohort, and to define disease segments or “immuno-types” based on the presence of single or multiple immune cell types. Users can then perform survival comparisons, explore gene expression of key cancer and IO genes as well as generate oncoprints in the different segments. The browser also provides statistical analysis to identify the gene or mutations enriched in the immuno-typed disease segment, and correlate gene expression or mutations with specific immune cell types in tumor microenvironment (TME).

**Conclusions**

In summary, IO Browser enables comprehensive analysis and visualization of the dynamic interactions between tumor and immune landscape, and will aid our understanding of the interplay between tumor genomics and immune biology to facilitate line of sight and disease segmentation.

#### P595 Smoking and KRAS mutation status in lung adenocarcinomas are associated with distinct favorable immune cell contexture in the tumor microenvironment

##### Yuanquan Yang, MD, PhD, Qiang Hu, MD, PhD, Song Liu, Maya Khalil, MD, Grace Dy

###### Roswell Park Comprehensive Cancer Center, Buffalo, NY, USA

####### **Correspondence:** Grace Dy (Grace.Dy@RoswellPark.org)

**Background**

EGFR mutated- NSCLC or NSCLC among never-smokers (regardless of EGFR or ALK mutation status) have inferior responses to anti-PD-1 antibody therapies compared to NSCLC with KRAS mutation or smoking-associated NSCLC.[1,2] We hypothesized that tumor immune microenvironment (IME) differences between smokers vs non- smokers, KRAS mutants vs EGFR mutants may explain this clinical association.

**Methods**

Using the Cancer Genome Atlas (TCGA) lung adenocarcinoma provisional database, genomic profiles and clinically annotated data were obtained. Patients without RNA-seq data were excluded. 22 different IME cell proportions were estimated using The Cancer Immunome Atlas (TCIA) and CIBERSORT gene expression deconvolutional algorithm LM22.[3,4] Relative IME cell proportions were compared using t-test. Holm-Bonferroni method was applied to control for type I error at alpha=0.05.

**Results**

75 lifelong non-smokers and 426 current/former smokers with lung adenocarcinomas were identified. 58 and 160 patients had putative driver mutations in EGFR and KRAS, respectively. Compared to non-smokers, smokers had significantly higher percentage of activated memory CD4 T cells (5.1% vs 2.6%, p=0.002), and plasma cells (10.1% vs 7.3%, p=0.003). In contrast, non-smokers had higher proportion of resting dendritic cells (9.7% vs 6%, p=0.003) (Figure 1). KRAS mutants had higher fractions of cytotoxic CD8 T cells (1.4% vs 0.5%, p=0.002), activated memory CD4 T cells (4.5% vs 2%, p=0.002) and plasma cells (9.9% vs 6.4%, p=0.003) compared to EGFR mutants (Figure 2). The rest of comparisons did not reach statistical significance.

**Conclusions**

There are distinct phenotypes of immune cell contexture of lung adenocarcinomas in association with smoking status and genotype. Smokers and KRAS mutants have a more favorable immune microenvironment compared to non-smokers and EGFR mutants. It may play a role in determining immunotherapy responsiveness.

**References**

1. Borghaei H, Paz-Ares L, Horn L, et al. Nivolumab versus docetaxel in advanced nonsquamous non–small-cell lung cancer. New England Journal of Medicine 2015;373:1627-39.
2. Reck M, Rodriguez-Abreu D, Robinson AG, et al. Pembrolizumab versus chemotherapy for PD-L1-positive non- small-cell lung cancer. N Engl J Med 2016;375:1823-33.
3. Charoentong P, Finotello F, Angelova M, et al. Pan-cancer immunogenomic analyses reveal genotype- immunophenotype relationships and predictors of response to checkpoint blockade. Cell Reports 2017;18:248-62.
4. Newman AM, Liu CL, Green MR, et al. Robust enumeration of cell subsets from tissue expression profiles. Nature Methods 2015;12:453.

**Fig. 1 (abstract P595).**
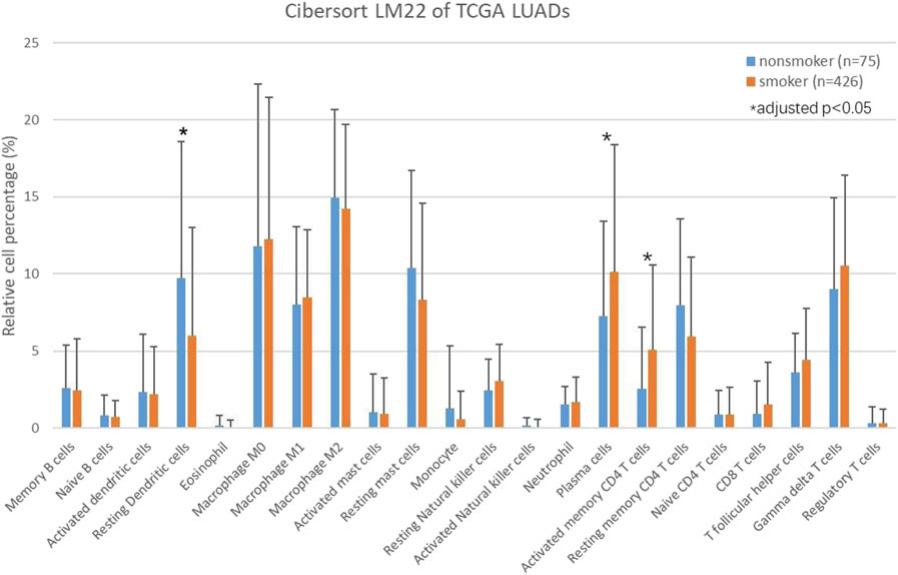
See text for description

**Fig. 2 (abstract P595).**
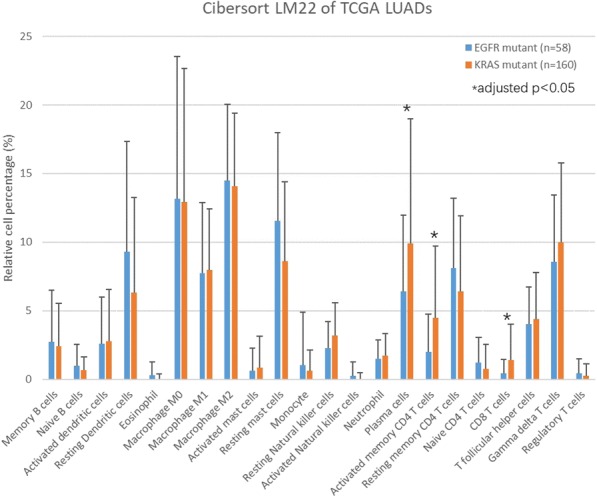
See text for description

#### P596 Immune gene expression characterization of genomic subsets of metastatic non-squamous non-small cell lung cancer

##### Edwin Yau, MD, PhD^1^, Sarabjot Pabla, MSc, PhD, BS^2^, Sean Glenn, PhD^2^, Antonios Papanicolau-Sengos, MD^2^, Jason Zhu, MD^3^, Matthew Labriola, MD^3^, Tian Zhang, MD^3^, Jeffrey Clarke, MD^3^, Carl Morrison, MD, DVM^4^, Grace Dy^1^

###### ^1^Roswell Park Comprehensive Cancer Center, Buffalo, NY, USA; ^2^Omniseq Inc., Buffalo, NY, USA; ^3^Duke Cancer Institute, Durham, NC, USA; ^4^Omniseq Inc, Buffalo, NY, USA

####### **Correspondence:** Edwin Yau (Edwin.Yau@RoswellPark.org)

**Background**

The use of PD-1/PD-L1 checkpoint inhibitors (CPI) has dramatically altered the treatment of metastatic Non-Small Cell Lung Cancer (NSCLC). However, a large proportion of patients with NSCLC do not derive clinical benefit from CPI treatment resulting in efforts aimed at improving patient selection. Molecular profiling of NSCLC has identified genomic subsets of NSCLC such as EGFR mutant NSCLC and KRAS mutant NSCLC with co-occurring mutations in the Nrf2 axis (STK11, KEAP1, and NFE2L2) that appear resistant to CPI monotherapy [1-3]. Using a cohort of patients whose tumors underwent comprehensive genomic and targeted immune transcriptomic analysis, we characterized the immune gene expression profile of EGFR mutant and Nrf2 altered NSCLC tumor samples.

**Methods**

We performed comprehensive analysis of the genomic and immunological landscape of 134 formalin-fixed, paraffin-embedded tumor samples from metastatic non-squamous NSCLC patients at eight institutions using a CLIA-certified laboratory test that included whole-exon DNA-seq and abundance of 394 immune transcripts by RNA-seq. Differential gene expression analyses of immune transcripts for various mutant subtypes was performed using Wilcoxon rank sum test on gene expression ranks (normalized gene expression values ranked against a large tumor reference population). Benjamini-Hochberg adjusted p-values are reported.

**Results**

Among the cohort of 134 cases, 8 were EGFR mutant, 28 were KRAS mutant (6 KRAS/TP53 and 4 KRAS/Nrf2 axis co-mutants), and 11 had Nrf2 axis mutations. In EGFR mutant cases, there was a trend towards enrichment of an IFN-g gene expression signature [4] and significant enrichment for a T-cell exhaustion gene expression signature [5] (p=0.043) (Fig. 1) including elevated expression of PD-L2 (p=0.031) and LAG3 (Fig. 2A). Nrf2 axis altered cases showed decreased PD-L1 expression (p=0.036) (Fig. 3) consistent with previous studies [2-3]. Nrf2 altered cases also demonstrated an increase in suppressive Treg genes and an immunosuppressive environment (Fig. 2B). Potential therapeutic targets of interest that were significantly increased in the Nrf2 altered cohort include NOTCH3 (p=0.048), TBP (p=0.033), HGF (p=0.043), and BCL2L11 (p=0.034). Similar trends were observed comparing the smaller subsets of KRAS and TP53 co-mutants compared to KRAS and Nrf2 altered subsets (Fig. 2C).

**Conclusions**

Using NSCLC patient tumor samples from multiple institutions, we characterized the immune gene expression signatures of CPI resistant EGFR and Nrf2 altered NSCLC genomic subsets and identified different potential mechanisms of CPI resistance in these genomic subsets. Alternative checkpoints resulting in T-cell exhaustion was observed in EGFR mutant NSCLC and an immunosuppressive Treg dominated signature was seen in Nrf2 altered NSCLC.

**References**

1. Lee CK, Man J, Lord S, Links M, Gebski V, Mok T, Yang JC. Checkpoint inhibitors in metastatic EGFR-mutated non-small cell lung cancer – a meta-analysis. J Thorac Oncol. 2017; 12:403-407. 2. Skoulidis F, Goldberg ME, Greenawalt DM, Hellmann MD, Awad MM, et al. STK11/LKB1 mutations and PD-1 inhibitor resistance in KRAS- mutant lung adenocarcinoma. Cancer Discov. 2018; 8:822-835. 3. Arbour KC, Jordan E, Kim HR, Dienstag J, Yu HA, et al. Effects of co-occurring genomic alterations on outcomes in patients with KRAS-mutant non-small cell lung cancer. Clin Cancer Res. 2018; 24:334-340. 4. Ayers M, Lunceford J, Nebozhyn M, Murphy E, Loboda A, et al. IFN-g-related mRNA profile predicts clinical response to PD-1 blockade. J Clin Invest. 2017; 127:2930-2940. 5. Guo X, Zhang Y, Zheng L, Zheng C, Song J, et al. Global characterization of T cells in non-small cell lung cancer by single-cell sequencing. Nat Med. 2018; 24:978-985.

**Ethics Approval**

OmniSeq’s analysis utilized deidentified data that qualified as non-human subject research under IRB-approved protocols, approved by both Roswell Park Comprehensive Cancer Center (Buffalo, NY, BDR #080316) and Duke Cancer Institute (Durham, NC, PRO00088762).

**Fig. 1 (abstract P596).**
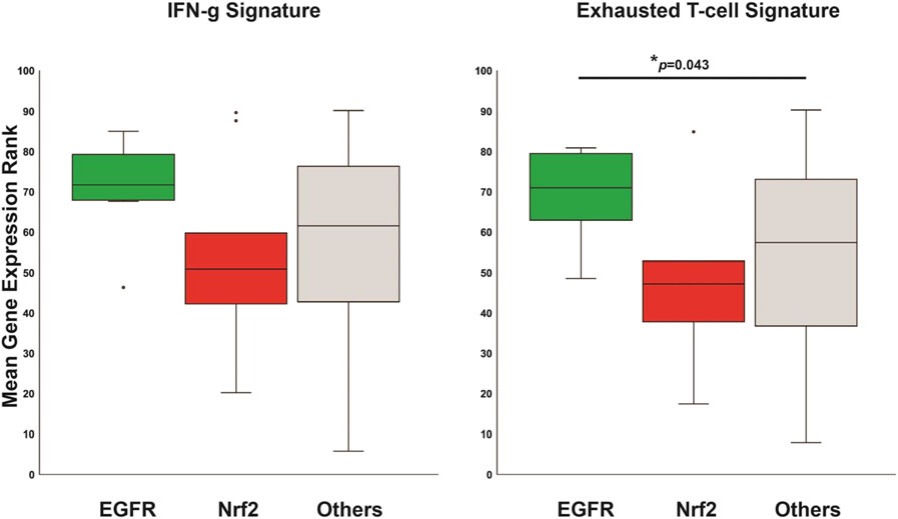
Gene Expression Signature

**Fig. 2 (abstract P596).**
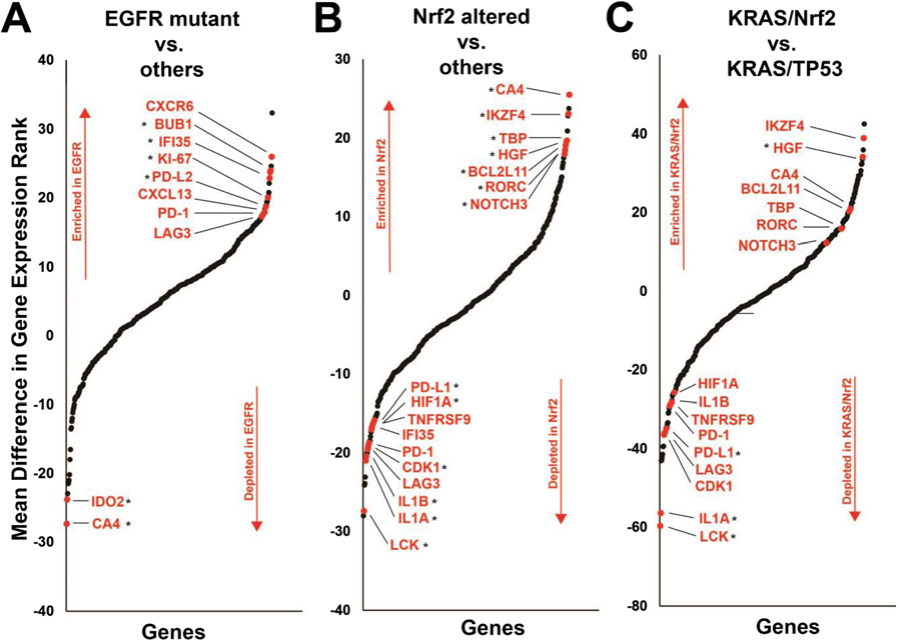
Gene Rank Score comparisons

**Fig. 3 (abstract P596).**
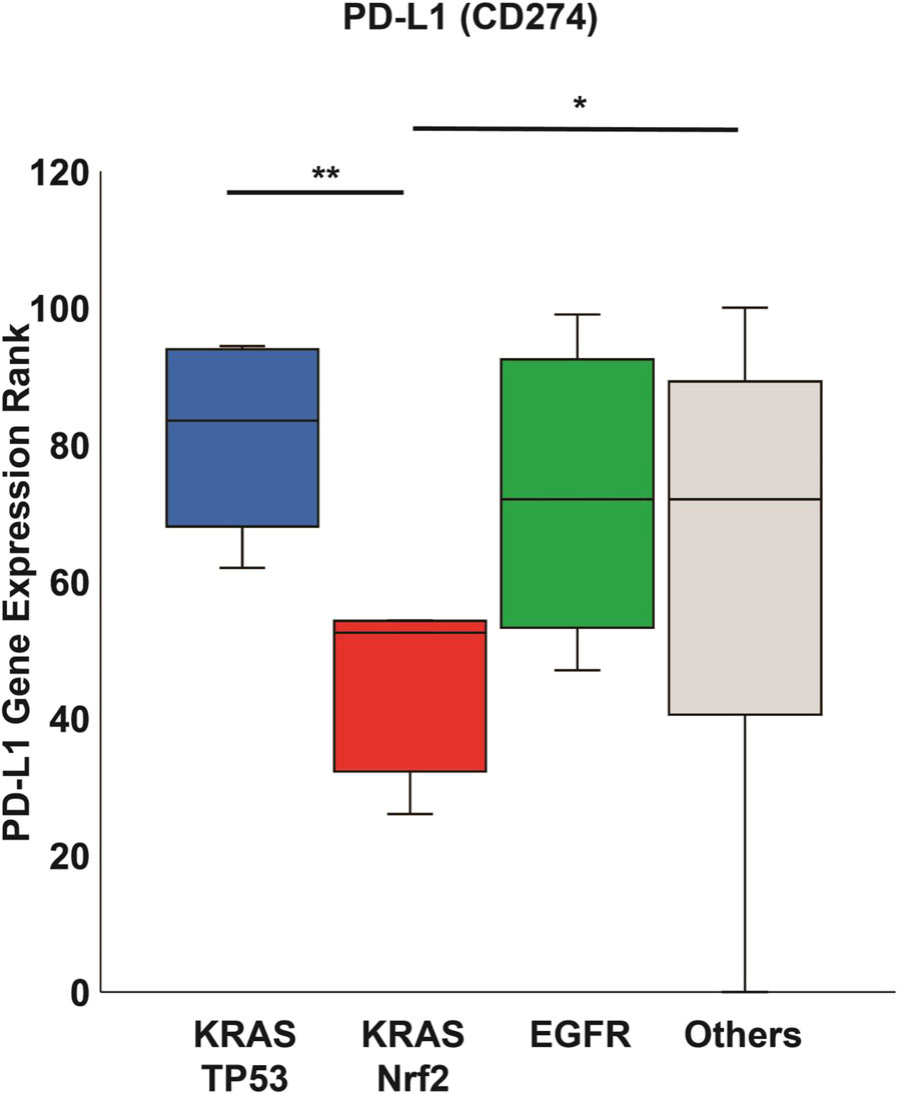
PD-L1 gene expression

### Oncolytic Viruses and Intratumoral Therapies

#### P597 Reovirus infection of prostate cancer induces upregulation of the negative regulators PD-L1 and BTLA

##### Nicola Annels, PhD, Guy Simpson, Hardev Pandha, PhD, FRCP, FRACP, FR, Mehreen Arif, Kevin Harrington, MD, Alan Melcher, Richard Vile, PhD

###### The University of Surrey, Guildford, UK

####### **Correspondence:** Hardev Pandha (h.pandha@surrey.ac.uk)

**Background**

Prostate cancers are generally considered to be a ‘cold’ tumour with this non-inflamed phenotype thought to be largely responsible for the disappointing lack of sensitivity of prostate cancer patients to immune checkpoint blockade (ICB) therapy. However, the use of oncolytic viruses can overcome pre-existing mechanisms of resistance to ICB in prostate cancers by transforming these cold tumours into ‘hot’, immune cell infiltrated, tumours. Such biological therapy can be further enhanced with the use of relevant immune checkpoint blockade that can overcome any constitutive or compensatory inhibitory resistance mechanisms. In this study, we investigated whether the effectiveness of oncolytic viral therapy for prostate cancer could be improved with targeted blockade of PD-1 and/or CD73.

**Methods**

The susceptibility of prostate cancer cell-lines to reovirus infection was tested in-vitro using MTS assays. The immunogenic cell death profile of reovirus-infected TRAMP-C2 cells was determined by analysing the cell-surface expressed ICD determinants, calreticulin and HSP70, by FACS and the secreted determinants by ELISA (HMGB1) or ATP assay. The capacity of reovirus to target TRAMP-C2 tumours in-vivo were evaluated using immunocompetent C57BL/6. Anti-PD-1 and/or anti-CD73 blockade in the TRAMP-C2 prostate cancer murine model was tested as a monotherapy and in combination with reovirus infection. Nanostring’s PanCancer Immune Profiling RNA Panel was used to investigate the impact of reovirus therapy on the tumour microenvironment.

**Results**

Prostate cancer cell-lines were highly susceptible to reovirus infection and displayed significant increases in the immunogenic cell death determinants, calreticulin and HMGB1, post-infection. Using the TRAMP-C2 immunocompetent C57BL/6 murine model, reovirus but not PBS-treated tumours resulted in significant tumour control but did not induce complete regression of tumours. Blockade with either anti-CD73 or anti-PD-1 as a monotherapy did not significantly control tumour growth. However, the addition of these antibodies after reovirus infection significantly controlled tumour growth resulting in complete tumour regressions in some mice who subsequently displayed protective immunity to tumour rechallenge. Whilst Nanostring analysis revealed the expected increase in immune infiltration within the virus-treated TRAMP-C2 tumours, even more interesting was the finding of significant upregulation of the negative regulators, BTLA and PD-L1 in the reovirus-treated TRAMP- C2 tumours compared to untreated tumours.

**Conclusions**

Whilst blockade of the PD-L1 signal with an anti-PD-1 antibody clearly sensitized TRAMP-C2 tumours to cytotoxic T lymphocyte (CTL) killing in the current study, results will be presented to show whether blockade of BTLA can synergise with anti-PD-1 at reversing tumour-specific T cell inhibition.

#### P598 Discovery and preclinical development of E7766, a novel STING agonist for cancer immunotherapy with a superior profile over a leading reference compound

##### Xingfeng Bao, PhD, Kuan-Chun Huang, PhD, Atsushi Endo, Dinesh Chandra, PhD, Jiayi Wu, Dae-Shik Kim, Diana Albu, Karen Tendyke, Kara Loiacono, Thomas Noland, PhD, Christy Ingersoll, David Verbel, MPH, Rongrong Jiang, Donna Kolber-Simonds, Chi Zhang, Muzaffar Akram, MSc, MA, Minghong Hao, HyeongWook Choi, Vaishali Dixit, Janna Hutz, PhD, John Wang, Frank Fang

###### Eisai Inc., Andover, MA, USA

####### **Correspondence:** Xingfeng Bao (xingfeng_bao@eisai.com)

**Background**

Activation of Stimulator of Interferon Genes (STING) in tumor microenvironment is considered to be a novel and promising approach to cancer immunotherapy via turning immune cold tumors into immune hot tumors. Here, we present for the first time E7766, a novel and proprietary STING agonist, and its preclinical profile in comparison with clinical stage reference compound X

**Methods**

E7766 was designed and synthesized to optimize the potency of binding to dimerized STING proteins of different genetic isoforms. Co-crystalization of E7766 with recombinant STING proteins were performed. E7766 was comparatively characterized with 2’3’-cGAMP and compound X in a variety of biochemical, molecular and cell biological, and in vivo pharmacological assays including human primary cells and tumor tissues. ADME and pharmacokinetics were determined.

**Results**

E7766, a representative of a novel class of compounds, was found to be a highly specific agonist. In primary human cells, E7766 was consistently active across all seven tested STING genetic isoforms and one to three orders of magnitude depending on the isoforms more potent than compound X. The broader target specificity and superior potency of E7766 were explained by advantageous molecular interactions in its co-crystal structures with various STING genetic isoforms. E7766 had no direct anti-proliferative activity in vitro, but when E7766 was administrated as single intratumoral (I.T.) injection to twelve subcutaneous syngeneic tumor models, all tumors responded by either complete regression or significant tumor growth delay without serious adverse effects. Single I.T. administration of E7766 eradicated anti-PD1 refractory large CT26 tumors or anti-PD1 resistant MC-38T1 tumors. Furthermore, single administration of E7766 to subcutaneous tumor in mice bearing dual CT26 tumors in liver and under skin cured 90% of animals without recurrence for over 8 months. In contrast, compound X had 30% cure rate in the same treatment condition. The effective abscopal antitumor activity by E7766 was mediated by type I IFN signaling, CD8 T cells, and TNFα, and the tumor free mice completely rejected rechallenges of the same tumor in the absence of CD8 T cells or NK cells. Treatment with E7766 induced expression of signature innate genes, accumulation of active T cells, and tumor cell death. Finally, E7766 was metabolically stable and had a short half- life in plasma with moderate to high plasma clearance through a hepatic transporter-mediated mechanism.

**Conclusions**

We have developed a novel STING agonist E7766 that has an excellent and structurally-supported superior preclinical profile over compound X, supporting further clinical investigation for cancer immunotherapy.

#### P599 OncoVEXmGM-CSF in combination with checkpoint inhibition leads to tumor-specific systemic immunity and increased tumor antigen response in a murine syngeneic melanoma model

##### Keegan Cooke, BS^1^, Juan Estrada^1^, Petia Mitchell^1^, Jinghui Zhan^1^, Jing Qing^1^, Jude Canon^1^, Pedro Beltran^2^

###### ^1^Amgen, MS: 15-2-A, CA, USA; ^2^Unity Biotechnology, Brisbane, CA, USA

####### **Correspondence:** Keegan Cooke (kcooke@amgen.com)

**Background**

Talimogene laherparepvec (T-VEC) is a first-in-class oncolytic immunotherapy derived from herpes simplex virus type 1 (HSV-1). T-VEC was designed to selectively replicate in neoplastic cells, lyse tumor cells, produce GM-CSF and stimulate anti-tumor immune responses. To study T-VEC-induced systemic immunity and T-cell responses against tumor antigens, we developed a mouse model of melanoma. We have previously shown that the combination of OncoVEXmGM-CSF (a virus modified similarly to talimogene laherparepvec, except that it contains mGM-CSF instead of hGM-CSF) and an anti-CTLA-4 antibody results in enhanced efficacy in both primary and metastatic B16F10 tumors. Here we set out to 1) determine whether OncoVEXmGM-CSF and anti-CTLA-4 combination treatment elicits a durable anti-tumor memory response and 2) to identify tumor-specific MHC-I epitopes generated by the treatment.

**Methods**

Experimental lung metastases were established by delivering B16F10-GFP cells intravenously (IV) to C57Bl/6 mice. Subcutaneous (SC) tumors were established by implanting B16F10-mNectin1 cells. When SC tumors reached~100mm3, mice were administered intratumoral injections of OncoVEXmGM-CSF every third day (3 doses). Anti- CTLA-4 antibody was dosed on the same schedule (4 doses). Naïve control mice and treated mice whose SC tumors had resolved were rested for 60 days and then re-challenged with B16F10 tumor cells either on the SC flank or IV. Lungs were collected from IV challenged mice on day 28 for enumeration of lung metastasis. B16F10 tumor antigen epitopes were identified using exome sequencing and MHC/HLA-binding prediction algorithms. Antigen-specific T cell response was measured using an IFN-γ ELISPOT assay.

**Results**

OncoVEXmGM-CSF in combination with an anti-CTLA-4 antibody led to complete SC tumor regression in 60% of mice. These mice rejected a re-challenge with B16F10 either on the SC flank (10/10 tumor-free vs. 0/10 in age- matched naïve mice) or when delivered IV (10/10 tumor-free lungs at day 28 vs. 0/10 in controls). Splenocytes from OncoVEXmGM-CSF treated mice were activated by the tumor antigen-specific peptide P15E and two neoantigen peptides. T cell responses to P15E were associated with efficacy in SC tumors in the combination group, suggesting that it may be a tumor-rejection antigen.

**Conclusions**

The combination of OncoVEXmGM-CSF and anti-CTLA-4 antibody treatment elicited a durable effector memory response against B16F10 tumors. Moreover, OncoVEXmGM-CSF induces both neo-antigen- and tumor antigen- specific T-cell responses. The response to P15E was augmented by anti-CTLA-4 and associated with efficacy of the combination treatment.

**Ethics Approval**

All in vivo work was conducted under an IACUC approved protocol and in an AAALAC accredited facility.

#### P600 IL12/IL15 and PD-L1 blocker co-expressing oncolytic herpes virus VG161 significantly alters tumor microenvironment and eliminates prostate tumors in animal models

##### Zahid Delwar, PhD^1^, Neetu Saxena, PhD^2^, Erica Lee, PhD^3^, Morgan Roberts, PhD^2^, Luke Bu, MSc^3^, Guoyu Liu, MD^3^, Yanal Murad, PhD^4^, Jun Ding, PhD^3^, Dmitry Chouljenko, PhD^3^, Igor Moskalev^2^, Syam Somasekharan, PhD^2^, Ronghua Zhao, MD^3^, Paul Rennie, PhD^1^, William Jia, PhD^3^

###### ^1^University of British Columbia, Vancouver, Canada; ^2^Vancouver Prostate Centre, Vancouver, Canada; ^3^Virogin Biotech Ltd, Vancouver, BC, Canada; ^4^Virogin Biotech Ltd., Vancouver, BC, Canada

####### **Correspondence:** Paul Rennie (prennie@prostatecentre.com)

**Background**

Oncolytic virotherapy has recently emerged as a promising anti-cancer therapeutic approach. A herpes virus-based oncolytic virus (oHSV-1), T-VEC, an oncolytic HSV-1 virus carrying GM-CSF, is the first oncolytic virus (OV) that has been approved by FDA in 2015 for treating melanoma. However, GM-CSF also stimulates myeloid derived suppressive cells (MDSCs) and therefore may not be the best immune stimulating factor to be expressed by OVs. To explore other immune regulatory factors that may better arm oHSV, we developed an oncolytic virus VG161 that expresses IL-12, IL-15 with its receptor alpha subunit and PD-L1 blocking peptide. VG161 has demonstrated to have a very strong antitumor effect in prostate cancer (PCa) mouse models.

**Methods**

Two expression cassettes that express IL-12 and IL-15 with its alpha receptor subunit, and PD-L1 blocking peptide (TF-Fc), respectively, were constructed into an oHSV backbone to generate VG161. Anti-tumor effect of VG161 virus was evaluated using MTT cell viability assay in a panel of prostate cancer cells in vitro. Viral replication in prostate tumor cells were determined by single step viral growth assay. In vivo anti-tumor efficacy was evaluated with a subcutaneously implanted human prostate cancer cell line (LNCaP), as well as the immunocompetent TRAMPC2 murine model of prostate cancer. Immune cell infiltration into the tumor microenvironment was determined by flow cytometry.

**Results**

Cell viability assay demonstrated that viability of PCa cells was reduced following VG161 infection in a dose dependent manner. Additionally, growth assay of VG161 in LNCaP cells showed the replication competency of the virus in PCacells. In vivo treatment of the LNCaP tumours with VG161 led to a dramatic reduction in tumor size compared to treatment with vehicle or replication deficient control virus. Antitumor efficacy by VG161 was also observed in the TRAMPC2 syngeneic prostate cancer model. Our data demonstrated that immunostimulatory transgene expression by VG161 virus, dramatically enhanced NK cell infiltration into LNCaP tumours in Nude mice. Moreover, VG161 intratumoral injections significantly increased cytotoxic CD8+ T cells in TRAMPC2 tumors in both subcutaneous and orthotopic models. More interestingly, immunosuppressive immune cells such as T regulatory cells and MDSC populations were significantly decreased after VG161 treatment.

**Conclusions**

Our study indicates that IL-12/IL-15 and PD-L1 blocker delivering oHSV-1 vector (VG161) alters the prostate tumor microenvironment and thereby significantly inhibits tumor growth.

#### P601 STING signaling can enhance melanoma antigenicity

##### Rana Falahat, PhD^1^, Adam Mailloux, PhD^1^, Patricio Perez-Villarroel^1^, Genyuan Zhu, PhD^1^, Shari Pilon-Thomas, PhD^1^, Glen Barber, PhD^2^, James Mulé, PhD^1^

###### ^1^Moffitt Cancer Center, Tampa, FL, USA; ^2^University of Miami Miller School of Med, Miami, FL, USA

####### **Correspondence:** James Mulé (James.Mule@moffitt.org)

**Background**

Among different gene-targeted mouse models deficient in specific innate immune pathways, only mice lacking STING or its downstream transcription factor IRF3 fail to reject immunogenic tumors [1]. Based on this finding, activation of STING by intratumoral injection of pharmacologic agonists has been investigated as a cancer therapy. While these studies have mainly focused on STING signaling in antigen presenting cells, its functional impact on tumor cells has not been well characterized. Here, we studied the impact of STING signaling activation on antigenicity of human melanoma cell lines following exposure to an agonist.

**Methods**

We examined STING and cGAS expression in a panel of human melanoma cell lines by immunoblot. Functional STING signaling activation was examined in STING-positive melanoma cell lines upon stimulation with the agonist 2’3’-cGAMP by measuring the induction of CXCL-10 and IFN-beta. To determine if hypermethylation was involved in the suppression of STING expression and signaling where gene mutations were absent, we treated melanoma cells lacking STING expression with 5-aza-2’-deoxycytidine (5AZADC). To study the role of STING signaling on antigenicity of melanoma, we co-cultured expanded human tumor infiltrating lymphocytes (TIL) with their HLA-matched melanoma cell lines in the presence or absence of 2’3’-cGAMP agonist. We assessed TIL production of IFN-γ and ^51^Cr release for cytotoxicity.

**Results**

Immunoblot analysis revealed a diverse STING/cGAS expression status in human melanoma cell lines. STING expression was not detected in 11 of 18 of them. Induction of STING expression in 5AZADC-treated melanoma cell lines lacking STING and production of CXCL-10 following their stimulation with the STING agonist suggested DNA hypermethylation involvement in cases where STING gene mutations were absent. Among STING-positive cell lines, two responded strongly to STING signaling activation with 2’3’-cGAMP. Activation of the STING pathway in these cell lines when cultured with their HLA-matched TILs resulted in up to a 15-fold increase in IFN-γ secretion (p < 0.01) as well as augmentation of TIL cytotoxicity by >2-fold (p < 0.05). In addition, STING activation could induce enhanced surface expression of MHC class I in human melanoma cell lines leading to more effective tumor antigen recognition by TIL.

**Conclusions**

Direct activation of the STING pathway in human melanoma cell lines can result in improved antigenicity. Further understanding of the regulation and function of STING in melanomas may lead to the development of new strategies using STING agonists to improve TIL-based immunotherapies.

**Acknowledgements**

Funding: Dr. Miriam and Sheldon G. Adelson Medical Research Foundation.

**References**

1. Woo, Seng-Ryong, et al. STING-dependent cytosolic DNA sensing mediates innate immune recognition of immunogenic tumors. Immunity. 2014;41(5): 830-842.

#### P602 Generation and characterization of a CTLA-4 antibody with improved FcgammaR-dependent Treg deletion for tumor microenvironment-targeted oncolytic virotherapy of cancer

##### Bjorn Frendeus, PhD^1^, Monika Semmrich, PHD^1^, Jean-Baptiste Marchand, pHD^2^, Petra Holmkvist^1^, Linda Mårtensson^1^, Ulla-Carin Tornberg^1^, Ingrid Teige^1^, Andres McAllister^1^, Eric Quemeneur, PharmD, PhD^2^, Nathalie Silvestre^2^

###### ^1^BioInvent, Lund, Sweden; ^2^Transgene S.A., Illkirch Graffenstaden, France

####### **Correspondence:** Bjorn Frendeus (bjorn.frendeus@bioinvent.com)

**Background**

Treatment with checkpoint inhibitor antibodies results in long-lasting antitumor responses in a variety of cancers [1]. However, a great unmet need remains since only a small fraction of patients responds. Reasons for lack of efficacy of checkpoint inhibition are believed to include lack of or inadequate tumor infiltrating immune cells (TIL), a notion supported by improved efficacy observed following combined checkpoint blockade with tumor microenvironment- modulating oncolytic virotherapy [2]. While combination therapy with anti-CTLA-4 and anti-PD-1 antibodies significantly improve efficacy, concerns with tolerability following systemic administration has limited wide-spread clinical use [3]. Here we present a potentially safe and more efficacious strategy to combine anti-CTLA-4 and anti-PD-1/PDL1 checkpoint inhibition in the context of oncolytic virotherapy. A Vaccinia virus (VV)-based oncolytic vector will be armed with herein described full-length human recombinant anti-CTLA-4 antibody, selected and characterized for its improved FcγR-dependent Treg depleting efficacy [4]. FcgammaR-dependent Treg depletion was recently found to underly human anti-CTLA-4 antibody efficacy in vivo, and FcgammaR-polymorphisms were associated with clinical responses to ipilimumab [4]. The Vaccinia virus vector was chosen based on its ability to achieve high intratumoral but low systemic exposure of encoded antibodies following intratumoral injection, and for its ability to induce intratumoral innate immune and effector T cell infiltration in conjunction with checkpoint inhibition in immunocompetent mouse tumor models [5-8].

**Methods**

The n-CoDeR® F.I.R.S.T™ human antibody and target discovery platform [9-12] was screened for antibodies with specificity for CTLA-4. Full-length antibody clones were characterized for efficacy and potency of binding human, cynomolgus and mouse CTLA-4 and for blocking CTLA-4 binding to B7 ligands. Functional activity was assessed in vitro monitoring CTLA-4-mediated T cell suppression and FcγR-dependent T cell depletion. Antibody-mediated deletion of human Treg in vivo was assessed using a human PBMC-based NOG/SCID-transfer model. Ipilimumab was used as benchmarking control.

**Results**

Biopanning, biochemical-, in vitro-, ex vivo- and in vivo-functional characterization of multiple antibody clones identified a lead human anti-CTLA-4 antibody candidate with improved Treg-depleting activity compared with ipilimumab. Its selective improved Treg deleting efficacy contrasted by near identical efficacy and potency compared with ipilimumab in overcoming CTLA-4-mediated effector T cell suppression.

**Conclusions**

A human CTLA-4 antibody optimized for FcgammaR-dependent Treg deletion has been generated for arming of a Vaccinia-based oncolytic viral vector. Oncoviral tumor-localized delivery of this mAb may improve the therapeutic window for CTLA-4 targeted checkpoint intervention, allowing better tolerated and more effective combination therapy with approved antibodies targeting the PD-1/PDL1 axis.

**References**

1. Sharma P, Allison J P. The future of immune checkpoint therapy. Science, 2015; 348(6230): 56-61.
2. Ribas, A., et al., Oncolytic Virotherapy Promotes Intratumoral T Cell Infiltration and Improves Anti-PD-1 Immunotherapy. Cell, 2017; 170(6): 1109-1119 e10.
3. Postow, M.A., et al., Nivolumab and ipilimumab versus ipilimumab in untreated melanoma. N Engl J Med, 2015; 372(21): 2006-17.
4. Arce Vargas F, et al. Fc Effector Function Contributes to the Activity of Human Anti-CTLA-4 Antibodies. Cancer Cell, 2018.
5. Kleinpeter P, et al. Vectorization in an oncolytic vaccinia virus of an antibody, a Fab and a scFv against programmed cell death -1 (PD-1) allows their intratumoral delivery and an improved tumor-growth inhibition. Oncoimmunology, 2016; 5(10): e1220467.
6. Fend, L., et al., Immune Checkpoint Blockade, Immunogenic Chemotherapy or IFN-alpha Blockade Boost the Local and Abscopal Effects of Oncolytic Virotherapy. Cancer Res, 2017; 77(15): 4146-4157.
7. Liu, Z., et al., Rational combination of oncolytic vaccinia virus and PD-L1 blockade works synergistically to enhance therapeutic efficacy. Nat Commun, 2017; 8: 14754.
8. Marchand, J.-B., et al., Antibody-armed oncolytic Vaccinia virus to block immunosuppressive pathways in the tumor microenvironment, in Society of Immunotherapy 33rd Annual meeting. 2018, Journal of Cancer Therapy: National Harbour, MD.
9. Soderlind, E., et al., Recombining germline-derived CDR sequences for creating diverse single-framework antibody libraries. Nat Biotechnol, 2000; 18(8): 852-6.
10. Roghanian A, et al. Antagonistic human FcgammaRIIB (CD32B) antibodies have anti-tumor activity and overcome resistance to antibody therapy in vivo. Cancer Cell, 2015; 27(4): 473-88.
11. Frendeus B., Function-first antibody discovery: Embracing the unpredictable biology of antibodies. Oncoimmunology, 2013; 2(8): e25047.
12. Ljungars, A., et al., A platform for phenotypic discovery of therapeutic antibodies and targets applied on Chronic Lymphocytic Leukemia. Precision Oncology, 2018. In press.

**Ethics Approval**

Ethical approvals for human cells were obtained by the Ethics Committee of Skåne University Hospital and written informed consent was provided in accordance with the declaration of Helsinki. Mouse experiments were performed in agreement with ethical permissions from Malmö Lund Animal Ethics Committee.

**Consent**

Ethical approvals for human cells were obtained by the Ethics Committee of Skåne University Hospital and written informed consent was provided in accordance with the declaration of Helsinki. Mouse experiments were performed in agreement with ethical permissions from Malmö Lund Animal Ethics Committee.

#### P603 Novel treatment strategy for peritoneal carcinomatosis: adoptive cell transfer of tumor-specific lymphocytes after dual therapy with oncolytic virus and PD-1 blockade

##### Esther Giehl, MD^1^, David Bartlett, MD^2^, Zong Sheng Guo, PhD^2^, Mathilde Feist, MD^3^

###### ^1^University of Pittsburgh Medical Center, Pittsburgh, USA; ^2^UPMC, Pittsburgh, PA, USA; ^3^Charite, Berlin, Germany

####### **Correspondence:** David Bartlett (bartlettdl@upmc.edu)

**Background**

Current treatment strategies for peritoneal carcinomatosis (PC), the most frequent metastatic spread of primary gastrointestinal tract cancers, are associated with high recurrence rates and poor long-term survival. Preclinical evidence demonstrates that oncolytic viruses (OV) can be delivered intraperitoneally and augment the immune response within the peritoneal cavity. vvDD and its IL15-IL15-Rα-fusion protein-expressing version (vvDD-IL15- Rα) stimulate a highly CD8 T cell dependent antitumor immunity in colon or ovarian murine cancers in vivo. OV’s ability to induce a T cell population with potent tumor-specific reactivity might present a novel strategy to overcome the immunosuppressive tumor microenvironment of solid organ malignancies and thereby augmenting their response to adoptive cell transfer (ACT).

**Methods**

Female C57BL/6 mice were inoculated intraperitoneally (i.p.) with colon adenocarcinoma cells (MC38). Tumor bearing mice underwent regional i.p. treatment with parental vvDD, vvDD-IL15-Rα or PBS. At different post treatment days, i.p. lavage was performed and T cells were isolated with magnetic beads. Virally induced ascites fluid lymphocytes have been tested for tumor cell recognition ex vivo via coculture assay, ELISA, ELISPOT and flow cytometry and applied for ACT.

**Results**

OV i.p. therapy stimulates a strong immune response with a 100-fold increase of the amount of T cells (p<0.005). Ten days post treatment, T cells isolated from vvDD-IL15-Rα-treated mice exibit a more potent adaptive MC38- specific immunity in comparison to those isolated from control mice as measured by elevated IFNγ release (p<0.005) or 4-1BB expression (p<0.005) on CD8 T cells. CD8 T cells isolated from ascites fluid 10 days after i.p. vvDD-IL15-Rα injection also exert an increased expression of PD-1 (p<0.005) and coexpression of PD-1 and TIGIT (p<0.5). The transfer of these T cells in combination with anti-PD-1 antibody to MC38 tumor bearing mice in the absence of OV therapy cures 80% of animals and extends survival (p<0.005).

**Conclusions**

Regional OV therapy of PC stimulates the i.p. T cell population and induces an adaptiv tumor-specific immune response. When applied in ACT in combination with an anti-PD-1 antibody, these OV induced T cells exert potent therapeutic efficacy as depicted by a high complete response rate and prolonged overall survival. The results demonstrate that the combination of OV and PD-1 blockade possesses great therapeutic potential to enhance efficacy of ACT as treatment strategy of solid cancers as highlighted in this preclinical study.

**Acknowledgements**

I thank Dr. Udai S. Kammula and Dr. Abhishek K. Srivastava for their guidance with regard to their expertise in T cell immunology.

#### P604 DNA-based delivery of immunomodulatory antibodies is effective both in muscle and tumor despite distinct pharmacokinetics

##### Liesl Jacobs, MPharm, Elien De Smidt, Nick Geukens, Paul Declerck, Kevin Hollevoet, PhD

###### KU Leuven - University of Leuven, Leuven, Belgium

####### **Correspondence:** Kevin Hollevoet (kevin.hollevoet@kuleuven.be)

**Background**

Many cancer patients fail to optimally benefit from monoclonal antibody (mAb) immunotherapy because of the high costs, limited single-agent efficacy and toxicity following systemic infusion. DNA-based antibody gene transfer administers the mAb-encoding nucleotides, rather than the protein, allowing the site of delivery to produce the therapeutic in a cost-effective manner[1]. Recently, we achieved preclinical proof of concept for intramuscular gene transfer of tumor-targeting mAbs using plasmid DNA (pDNA) electroporation, a well-established clinical approach[2]. In the current study, we aimed to extend our DNA platform to immunomodulatory mAb combinations and explore the tumor as site of pDNA delivery. We hypothesized that intratumoral mAb production can lead to local and systemic anti-tumor responses, while avoiding high systemic mAb exposure and associated toxicity. We thereto compared the efficacy and pharmacokinetics of intramuscular and intratumoral antibody gene transfer.

**Methods**

Plasmids were designed to express an optimized anti-CTLA-4 mAb (p(aCTLA-4)) or anti-PD-1 mAb (p(aPD-1)), and were evaluated in a subcutaneous MC38 mouse tumor model. An empty plasmid (pNull) served as a control. Intramuscular and intratumoral pDNA electroporation were applied using tweezer electrodes and the NEPA21 Electroporator.

**Results**

Intramuscular p(aCTLA-4) and p(aPD-1) electrotransfer significantly improved survival, with the former resulting in 10% complete responders (CRs). The combination of both DNA-based mAbs further increased response, up to 30% CRs. pNull had no impact on tumor growth. Intratumoral delivery improved the efficacy of p(aPD-1) (33% CRs), while response rates of the other treatment arms were maintained (p(aCTLA-4): 9-17% CRs; combination: 33% CRs). Similar to the literature, intratumoral pNull electroporation resulted in a dose-dependent anti-tumor effect. The survival benefit, however, was less pronounced than when expressing the mAbs. In cured mice rechallenged with MC38 cells, tumor growth was absent or delayed, suggesting long-term anti-tumor immunity. Plasma mAb concentrations resulting from intratumoral mAb expression were up to 20-fold lower and more transient compared to intramuscular pDNA electrotransfer, and correlated with response. For both pDNA delivery sites, plasma mAb levels were similar in the single- and combination-treatment arms, demonstrating our ability to properly express mAb combinations.

**Conclusions**

Intramuscular and intratumoral DNA-based gene transfer of immunomodulatory mAbs enable significant anti-tumor responses, despite the distinct mAb pharmacokinetics. Combining a high efficacy with limited systemic mAb exposure, the tumor emerges as an appealing delivery site for DNA-based therapeutics.

**References**

1. Hollevoet K, Declerck PJ. J Transl Med. 2017; 15:131-150.2. Hollevoet K, et al. Oncotarget. 2018; 9:13623–13636.

**Ethics Approval**

This study was approved by the KU Leuven Animal Ethics Committee, approval number P130/2017.

#### P605 Localized treatment with oncolytic adenovirus Delta-24-RGDOX elicits efficacious abscopal immunity against disseminated melanomas

##### Hong Jiang, PhD^1^, Dong Ho Shin^2^, Caroline Carrillo, BS^2^, Verlene Henry, BS^2^, Teresa Nguyen, BS^2^, Yisel Rivera- Molina, PhD^2^, Frederick Lang, MD^2^, Candelaria Gomez-Manzano, MD^2^, Juan Fueyo^2^

###### ^1^University of Texas MD Anderson Cancer Center, Houston, TX, USA; ^2^MD Anderson Cancer Center, Houston, TX, USA

####### **Correspondence:** Juan Fueyo (jfueyo@mdanderson.org)

**Background**

We have reported that Delta-24-RGDOX, an oncolytic adenovirus expressing immune co-stimulator OX40 ligand (OX40L), induces efficacious anti-glioma immunity in syngeneic intracranial glioma models of immune competent mice. It is unknown if the virus could be used to treat metastatic melanomas.

**Methods**

Flow cytometry was used to determine the expression of OX40L on tumor cells, cell lysis, immunophenotyping of the lymphocyte populations. To study the abscopal immunity against metastatic melanomas induced by intratumoral injection of the virus in primary melanomas, we established subcutaneous/subcutaneous (s.c./s.c.) and subcutaneous/intracranial (s.c./i.c.) melanoma models with B16-Red-FLuc cells, a derivative of B16F10, in C57BL/6 mice to evaluate the effect of Delta-24-RGDOX on tumor-bearing mice survival, and the lymphocyte populations and their distribution in the mice. The tumor growth was monitored with bioluminescence imaging.

**Results**

Delta-24-RGDOX expressed mouse OX40L and induced cell lysis in both human and mouse melanoma cells. It replicated efficiently in human melanoma cells and also showed a moderate replication activity in B16-Red-FLuc cells. In the s.c./s.c. model, compared to treatment with PBS, Delta-24-RGDOX significantly inhibited the growth of both the injected and the untreated distant tumors, resulting in prolonged survival of the mice with 50% long-term survival (P = 0.001). The surviving mice is resistant to rechallenging with the same tumor cells but is susceptible to lung cancer cells, suggesting the development of immune memory specific to the virus-injected tumor type. The virus treatment increased the presence of CD3+ T lymphocytes, CD3+CD4+ helper T cells, CD3+CD8+ cytotoxic T cells and the effector T cell frequency, and decreased the amount of T regulatory cells in the tumor. In addition, analysis of T cells in the blood and spleen demonstrated that the virus mediated an abscopal effect which was indicated by the increment of effector CD4+ and CD8+ T cells frequency, and PD-1 expression on these cells. In the s.c./i.c. model, viral injection into the s.c. tumor induced anti-melanoma activity in the brain, resulting in growth inhibition of both the s.c. and i.c. tumors and an improved survival of the animals. The viral injections in the s.c. tumor increased the presence of T cells and the frequency of the effector cells in the hemispheres with the tumor implant.

**Conclusions**

Localized intratumoral injection of Delta-24-RGDOX induces an in situ antovaccination of the treated melanoma, of which the effect changes the immune landscape of the treated mice, resulting in the immunity against the disseminated s.c. or i.c. tumors.

**Ethics Approval**

The study was approved by the MD Anderson Cancer Center‘s Ethics Board, approval number 00000977-RN01.

#### P606 IFN gamma-independent upregulation of PD-L1 in oHSV-1 infected tumour cells

##### Erica Lee, PhD^1^, Jun Ding, PhD^2^, Sheng Yu^2^, Yanal Murad, PhD^2^, Dmitry Chouljenko, PhD^2^, Guoyu Liu, MSc, MD^2^, Zahid Delwar, PhD^3^, Luke Bu, MSc^2^, Will Liu, PhD^2^, Ronghua Zhao, MD^2^, William Jia, PhD^2^

###### ^1^Virogin Biotech Ltd., Vancouver, BC, Canada; ^2^Virogin Biotech Inc., Vancouver, BC, Canada; ^3^University of British Columbia, Vancouver, Canada

####### **Correspondence:** Erica Lee (elee@virogin.com)

**Background**

Oncolytic viruses (OVs) are capable of direct tumour lysis along with recruitment of anti-tumour immune response. Notably, a systemic effect can be mediated through the induction of systemic anti-tumour immunity, especially when OVs are armed with immunomodulators [1,2]. Recently, an approved oncolytic virus T-VEC shows dramatic improvement in clinical efficacy when combined with PD-1 antibody pembrolizumab [3]. This enhanced efficacy has been implicated through increasing T cell infiltration in tumor microenvironment. In the present study, we attempt to demonstrate that OV/PD-1 antibody combination might be necessary for the anti-tumour function of infiltrating T cells through restricting tumour resistance induced by oncolytic herpes simplex virus-1 (oHSV-1).

**Methods**

We constructed a novel oHSV-1 (VG161) encoding human IL-12 and IL-15/IL-15Ralpha to synergistically stimulate the function of immune cells, and a PD-1 mimic peptide to block PD-1/PD-L1 interaction. Liver and colon cancer cells were infected with VG161 in vitro or intratumorally injected with VG161 in vivo and the PD-L1 expression was evaluated. In experiment investigating which virus-related signaling pathways regulated PD-L1 expression, inhibitors specifically targeting JAK/STAT, STING, or MyD88 pathway were co-treated in VG161- infected cancer cells. To examine the anti-tumour efficacy of oHSV and PD-L1 blockade combination, VG161 was compared with parental oHSV only encoding human IL-12 and IL-15/IL-15Ralpha on stimulation of immune cell functions against cancer cells.

**Results**

Our results demonstrate that VG161 induces PD-L1 expression on liver and colon cancer cell surfaces immediately following infection. In addition, in vivo colon cancer model shows that tumours received VG161 injection intratumorally up-regulate PD-L1 protein level. The upregulation of PD-L1 expression by oHSV infection was mediated by IFN-beta but not IFN-alpha nor IFN-gamma. Furthermore, using inhibitors on different virus-related signaling pathways, we have shown that VG161-induced PD-L1 expression involves activation of STING and STAT that was independent of JAK signal. Finally, we show that an oHSV-1 expressing PD-L1 blocking peptide (VG161) induced stronger anti-tumor effect by immune cells than its parental oHSV-1 without PD-L1 blocker.

**Conclusions**

Our results suggest there is a reciprocal effect between oHSV and PD-1/PD-L1 blocker. oHSV delivery in tumour site increases T cell infiltration, but meanwhile also upregulates PD-L1 expression on tumour, which renders increased resistance to T-cell mediated cytotoxicity. Combing with PD-1/PD-L1 blocker might be necessary for better clinical efficacy of oHSV-1 virotherapy by suppressing virally induced PD-L1 inhibitory effect.

**References**

1. Bartlett DL, Liu Z, Sathaiah M, Ravindranathan R, Guo Z, He Y, Guo ZS. Oncolytic viruses as therapeutic cancer vaccines. Mol. Cancer. 2013; 12:103-118.
2. Lichty BD, Breitbach CJ, Stojdl DF, Bell JC. Going viral with cancer immunotherapy. Nat. Rev. Cancer. 2014; 14:559–567.
3. Ribas A, Dummer R, Puzanov I, VanderWalde A, Andtbacka RHI, Michielin O, Olszanski AJ, Malvehy J, Cebon J, Fernandez E, Kirkwood JM, Gajewski TF, Chen L, Gorski KS, Anderson AA, Diede SJ, Lassman ME, Gansert J, Hodi FS, Long GV. Oncolytic virotherapy promotes intratumoral T cell infiltration and improves anti-PD-1 immunotherapy. Cell. 2017; 170:1109-1119.

#### P607 The TLR4 agonist G100 enhances the efficacy of adoptive T-cell therapy

##### Jardin Leleux, PhD, Tina Albershardt, PhD, Peter Berglund, PhD, Jan Ter Meulen, MD, PhD,

###### Immune Design, Seattle, WA, USA

####### **Correspondence:** Jardin Leleux (jardin.leleux@immunedesign.com)

**Background**

Immunosuppression in the tumor microenvironment (TME) and immune escape mechanisms of tumor cells may impede the effectiveness of adoptive cell therapies (ACT). To this end, strategies to reverse these mechanisms by enhancing T-cell trafficking and immune exposure of the tumor are needed. G100 is a synthetic TLR4 agonist formulated for intratumoral treatment, which has been shown preclinically and clinically to inflame the TME, stimulate local draining lymph nodes, enhance antigen presentation and induce systemic CD4 and CD8 T cell responses that result in antitumor efficacy. G100 also improves infiltration of vaccine-induced T-cells into murine tumors. In this study, we investigated whether the efficacy of ACT is enhanced when combined with intratumoral G100.

**Methods**

Female C57BL/6 recipient mice were inoculated with B16F10 or ovalbumin-expressing-B16F10 (B16-OVA) melanoma tumors. Once tumors were palpable, biweekly treatments of G100 were initiated. Splenic CD8+ T cells from OT-I and pmel mice, which carry a rearranged TCR transgene specific for an OVA or gp100 epitope, respectively, were magnetically isolated and transferred into the tumor-bearing mice. Tumor growth was monitored 2-3 times per week by caliper measurement until tumors either completely regressed or mice were euthanized due to tumor growth. In some experiments, tumors and tumor-draining lymph nodes were isolated from animals and analyzed by flow cytometry for infiltration of transferred CD8+ T cells.

**Results**

Mice that received both ACT and G100 treatments (“transfer-pull”) experienced significantly enhanced tumor protection compared to mice that received ACT or biweekly intratumoral G100 alone. Treatment of B16-OVA tumor-bearing mice using the transfer-pull regimen resulted in complete tumor regression in 70% of the animals, whereas no tumor regression was observed for animals receiving either monotherapy. Consistent with the proposed mechanism of action, actively proliferating transferred T-cells were present in tumors as well as tumor-draining lymph nodes of transfer-pull treated mice. When targeting the less immunogenic gp100 melanoma antigen, median survival was significantly extended and complete regression observed in up to 28% of animals.

**Conclusions**

These data collectively demonstrate that intratumoral G100 can be effectively used in combination with adoptive cell therapy to enhance tumor rejection and survival, warranting further preclinical and clinical evaluation.

#### P608 ProTriTAC: a protease-activatable T cell engager platform that links half-life extension to functional masking and expands therapeutic window to enable targeting of broadly expressed tumor antigens

##### S. Jack Lin, PhD, Maria Rosalyn Dayao, Kendrick Kim, Sony Rocha, Kathryn Kwant, PhD, Timothy Yu, Thomas Evans, Stephen Yu, Michael Cremin, Wade Aaron, BS, Maria Gamez-Guerrero, Evan Callihan, Golzar Hemmati, Kevin Wright, PhD, Yinghua Xiao, Master Degree, Manasi Barath, Che-Leung Law, PhD, Bryan Lemon, PhD, Richard Austin, PhD, Holger Wesche, PhD

###### Harpoon Therapeutics, Inc., South San Francisco, CA, USA

####### **Correspondence:** S. Jack Lin (jlin@harpoontx.com)

**Background**

T cell engagers, such as blinatumomab, have demonstrated clinical activity in several hematological malignancies, but their use in solid tumors is limited by the low number of antigens that are expressed in tumors but not in normal tissues. Conditionally active T cell engagers that function preferentially in the tumor microenvironment offer a path to expanding the therapeutic window by reducing their on-target but off-tumor activity. Here, we describe a prodrug version of our T cell engager platform, termed ProTriTAC, that is activated by proteases in the tumor microenvironment and enables the safe targeting of broadly expressed solid tumor antigens.

**Methods**

ProTriTACs were engineered with three binding domains on a single polypeptide: anti-albumin for half-life extension, anti-CD3e for T cell engagement, and anti-tumor-associated antigen. They have an anti-albumin domain, comprising a masking moiety and a protease-cleavable linker, to keep the molecules inert outside the tumor microenvironment. Activation by tumor-associated proteases removes the anti-albumin domain along with the masking moiety to reveal the active drug. The masking moieties were identified using phage display. Binding studies to recombinant CD3e protein were determined using ELISA assays and to primary T cells using flow cytometry assays. T cell engager function was assessed using T cell-dependent cellular cytotoxicity (TDCC) assays with resting human T cells. In vivo efficacy studies were performed using a subcutaneous tumor xenograft model in immunodeficient NCG mice.

**Results**

Proof-of-concept experiments were carried out in vitro and in vivo. The protease-activated ProTriTAC had markedly increased binding to recombinant CD3e protein and to human primary T cells as well as increased T cell-redirected killing activity in TDCC assays when compared to the prodrug. Consistent with tumor-dependent activation of ProTriTACs in vivo, ProTriTACs have comparable anti-tumor activity to the unmasked molecule but significantly more anti-tumor activity than the masked non-cleavable molecule.

**Conclusions**

ProTriTACs are designed as long-lived inert prodrugs when in circulation and become short-lived active drugs for T cell-redirected tumor killing when activated in the tumor microenvironment. This half-life differential between the prodrug and the active drug is desirable as any aberrant activation of prodrug outside the tumor will be cleared rapidly, thereby further expanding the therapeutic window. This technology enables more T cell engager targets for solid tumors, and we are building a pipeline of ProTriTACs against these targets.

**Ethics Approval**

In vivo studies were reviewed and approved by Harpoon's Institutional Animal Care and Use Committee.

#### P609 First in man study of TK positive oncolytic vaccinia virus delivered by adipose stromal vascular fraction cells

##### Boris Minev, MD^1^, Elliot Lander, MD^3^, John Feller, MD^4^, Mark Berman, MD^5^, Stuart May, MD^4^, Bernadette Greenwood^4^, Ivelina Minev, MSc^6^, Duong Nguyen, PhD^6^, Antonio Santidrian^6^, Dobrin Draganov, PhD^6^, Mehmet Kilinc, PhD^6^, Santosh Kesari, MD, PhD^7^, Edward McClay, MD^8^, Gabriel Carabulea, MD^9^, Aladar Szalay, PhD^6^

###### ^1^Moores University of California San Dieg, San Diego, CA, USA; ^2^Moores UCSD Cancer Center, San Diego, CA, USA; ^3^California Stem Cell Treatment Center, Rancho Mirage, CA, USA; ^4^Desert Medical Imaging, Indian Wells, CA, USA; ^5^Cell Surgical Network, Beverly Hills, CA, USA ^6^Calidi Biotherapeutics, San Diego, CA, USA; ^7^John Wayne Cancer Institute, Santa Monica, CA, USA; ^8^cCare, Encinitas, CA, USA; ^9^Ocean View Hematology and Oncology Group, San Clemente, CA, USA

####### **Correspondence:** Boris Minev (bminev@ucsd.edu)

**Background**

Recent oncolytic virus clinical studies have shown safety and implied anti-tumor activity. However, a major obstacle to this approach has been the rapid oncolytic virus elimination by patient’s immune system. We hypothesized that oncolytic viruses would be protected and delivered efficiently to tumor sites by autologous adipose stromal vascular fraction (SVF) cells. Effective virus protection by adipose derived cells has been confirmed in preclinical studies. Here, we report the results of a first-in-man trial to determine the safety and feasibility of this approach in patients with advanced solid tumors and AML.

**Methods**

In this single-arm, open-label safety study, 24 patients with advanced solid tumors and 2 patients with AML were treated with a single administration of the oncolytic virus ACAM2000 (vaccinia) delivered by SVF cells. Patients received ACAM2000/SVF by intravenous application, or by a combination of intravenous and intratumoral or intra- peritoneal injections. The dose for ACAM2000 was between 1.4 x 106 pfu to 1.8 x 107 pfu incubated with same number of SVF cells. The primary endpoint was safety/tolerability by incidence of dose-limiting toxicity. Secondary endpoints included evaluations of overall survival and induction of anti-tumor and anti-vaccinia immune responses. Blood samples were collected at multiple time points for quantifying vaccinia virus DNA in peripheral blood by qPCR. In addition, levels of 30 plasma cytokines and the effects on activated T cells, Tregs, memory T cells, NK cells, and MDSC were analyzed.

**Results**

No serious toxicities (> grade 2) were reported. Eight of the 26 subjects reported an AE: self-limiting skin lesions, lasting 7 to 18 days – an expected reaction to ACAM2000. No infusion-related AEs were reported. No AEs leading to study discontinuation were reported. Viral DNA was detected in all patients immediately following treatment. Interestingly, in 8 patients viral DNA disappeared 1 day and re-appeared 1 week post treatment, suggesting active viral replication, possibly at tumor sites. This viral DNA reappearance correlated with longer survival of these patients. No major increase in cytokine levels was observed in any of the patients. No correlation between cytokine levels and pox lesions was noted. Flow cytometry showed gradual changes suggesting improved immune cell activation status. Tumor size reduction was documented in several patients.

**Conclusions**

Treatment with ACAM2000/SVF in patients with advanced solid tumors and AML is safe and well tolerated, with clear antitumor effects in several patients. These promising initial clinical results merit further investigation of therapeutic utility.

**Acknowledgements**

Boris Minev, MD and Elliot Lander, MD contributed equally to this work.

**Ethics Approval**

The study was approved by International Cell Surgical Society‘s Ethics Board, approval number ICSS-2017-004

#### P610 Transcriptome analysis of CT26 tumors treated with HSV-1 oncolytic virus expressing multiple immune factors

##### Yanal Murad, PhD^1^, Jun Ding, PhD^1^, Erica Lee, PhD^1^, Dmitry Chouljenko, PhD^1^, Luke Bu, MSc^1^, Guoyu Liu, MSc, MD^1^, Zahid Delwar, PhD^2^, Will Liu, PhD^1^, Ronghua Zhao, MD^1^, William Jia, PhD^2^

###### ^1^Virogin Biotech. Inc., Vancouver, BC, Canada; ^2^University of British Columbia, Vancouver, Canada

####### **Correspondence:** William Jia (w.jia@ubc.ca)

**Background**

Oncolytic HSV-1 (oHSV-1) treatment induces a potent immune response against tumor antigens, which can be augmented once combined with other immune stimulatory factors. Previously, we have generated an oncolytic HSV-1 virus (VG161) which carries 2 immunomodulator cytokines, IL12 and IL15/IL15RA1, along with a PD-L1 mimic peptide capable of blocking PD-1/PD-L1 interaction. These factors work synergistically to trigger and maintain an efficient anti-tumor immune response in the tumor microenvironment. In this work, we demonstrate the superior activity of the VG161 virus, compared to the back-bone virus (with no payloads) using a mouse colon cancer tumor model. We also perform a transcriptome analysis to determine the differential gene expression in the treated tumors and compare the two treatments.

**Methods**

We tested the efficacy of VG161 treatment in CT26 mouse model and examined the differential expression of transcriptome in these tumors collected 5 days post treatment with VG161 or the corresponding backbone virus. After extracting the RNA from the tumor samples, we performed RNA sequencing and analyzed differential expression of genes and compared them between the 2 treatments. qRT-PCR was used to validate targets identified by RNA sequencing.

**Results**

In the CT26 model, tumors regressed to undetectable limits upon intra-tumoral injection with VG161. When the treated mice were challenged with the same tumor, the tumor cells did not grow. Tumor treated with VG161 has demonstrated a higher number of tumor-infiltrating CD8 T cells, which activity against the tumor cells was also demonstrated by ELISpot assay. Differential expression of 24342 genes was performed and 18 differentially expressed genes with q-Value <0.05 were identified. These genes included chemokines and interferon response elements associated with inflammatory response, along with acute phase proteins. Some of the overexpressed genes, especially those related IFN response elements genes were also validated by qRT-PCR.

**Conclusions**

We have demonstrated by transcriptome analysis that VG161, a novel oncolytic virus which can induce a strong anti-tumor immunity and oncolytic activity, can induce multiple genes which result in efficacy against tumors. The efficacy of VG161 can be partially attributed to the immune response generated by the modified virus, which is likely induced by the change in the tumor microenvironment triggered by the VG161 payload. Further work is needed to dissect the function of each of the differentially expressed genes to understand the role they play in the regression of cancer upon VG161 treatment.

#### P611 Withdrawn

#### P612 Overcoming oncolytic poliovirus-mediated adaptive immune resistance by combining with anti-PD1/-PDL1 therapy in cancer

##### Smita Nair, PhD, Eda Holl, PhD, RAC, Michael Brown, PhD, Victoria Frazier, David Boczkowski, BS, MSc, Vidyalakshmi Chandramohan, PhD, Darell Bigner, MD, PhD, Shelley Hwang, Matthias Gromeier, MD

###### Duke University School of Medicine, Durham, NC, USA

####### **Correspondence:** Smita Nair (smita.nair@duke.edu)

**Background**

Oncolytic poliovirus (OncPV) PVSRIPO is a recombinant, non-pathogenic polio:rhinovirus chimera that targets cancer cells via CD155. PVSRIPO also targets antigen-presenting cells (APCs), including dendritic cells (DCs) and macrophages. PVSRIPO infection of APCs induces sustained type I interferon and APC activation (1). In a phase-1 clinical trial of intratumor PVSRIPO in 61 patients with recurrent glioblastoma (GBM), the survival rate at 24- months and 36-months was 21% (2). This study examines the following hypotheses: 1] Intratumor OncPV administration causes oncolysis and inflammation, which stimulates innate and adaptive immunity; 2] Immune cell activation in tumor triggers adaptive immune resistance via the PD1/PDL1 axis; 3] Blocking PD1/PDL1 in conjunction with OncPV will eliminate adaptive resistance and potentiate durable antitumor immunity.

**Methods**

OncPV-mediated immune activation was analyzed in: 1] immunocompetent murine models of orthotopic E0771 breast cancer and subcutaneous B16 melanoma; 2] human tumor cell lines and primary human tumor tissue from patients with breast cancer, melanoma and GBM; 3] human DCs and macrophages. To investigate combination PVSRIPO and PD1/PDL1 blockade, C57BL6-CD155 transgenic mice were orthotopically implanted with E0771- CD155 tumor cells. Mouse PVSRIPO (mRIPO) was injected intratumorally once in 50-100 mm3 tumors. Checkpoint antibodies were injected intraperitoneally 1-day post-mRIPO, 4-6 times every 3 days. Tumor growth was monitored.

**Results**

Intratumor mRIPO induced recruitment of immune cells (Figure 1), a classical acute inflammatory response and systemic antitumor cytotoxic T cell responses (1). Tumor infiltrating CD8/CD4 T cells demonstrated an effector phenotype and expressed PD1 (Figure 2). Infection of primary human tumors with PVSRIPO induced Stat1-p, IFIT1 and PDL1 expression and production of pro-inflammatory cytokines (Figure 2). Infection of human tumor cell lines with PVSRIPO induced PDL1 expression (Figure 2). PVSRIPO-infected primary human DCs and macrophages demonstrated sustained activation and PDL1 expression.Based on these data we investigated combination PVSRIPO with PD1/PDL1 blockade in murine breast cancer model. mRIPO, anti-PD1/-PDL1, and mRIPO+anti-PD1/-PDL1 significantly inhibited tumor growth compared to PBS. There were no significant differences in tumor growth inhibition between mRIPO and anti-PD1/-PDL1 monotherapies. Combination mRIPO+PD1/PDL1 blockade was significantly more effective than the monotherapies alone at controlling tumor growth.

**Conclusions**

We demonstrate that OncPV-mediated adaptive immune resistance involves the PD1/PDL1 axis and is mitigated by combining OncPV with anti-PD1/-PDL1 in murine breast cancer model. We are currently investigating PVSRIPO- mediated local and systemic immune bioactivity in women with triple-negative breast cancer and planning a trial of PVSRIPO with anti-PDL1 in women with breast cancer.

**Acknowledgements**

This study is funded by the Department of Defense Breast Cancer Research Program award (PI, Smita Nair).

**Trial Registration**

ClinicalTrials.gov Identifier: NCT03564782

**References**

1. Brown MC, Holl EK, Boczkowski D, Dobrikova E, Mosaheb M, Chandramohan V, Bigner DD, Gromeier M, Nair SK. Cancer immunotherapy with recombinant poliovirus induces IFN-dominant activation of dendritic cells and tumor antigen-specific CTLs. Sci Transl Med. 2017 Sep 20;9(408).
2. Desjardins A, Gromeier M, Herndon JE 2nd, Beaubier N, Bolognesi DP, Friedman AH, Friedman HS, McSherry F, Muscat AM, Nair S, Peters KB, Randazzo D, Sampson JH, Vlahovic G, Harrison WT, McLendon RE, Ashley D, Bigner DD. Recurrent Glioblastoma Treated with Recombinant Poliovirus. N Engl J Med. 2018; 379(2):150-161.

**Ethics Approval**

All studies are conducted under Duke University IACUC- and IRB-approved protocols. The human samples used in the study were conducted under IRB-exempt protocols.

**Fig. 1 (abstract P612).**
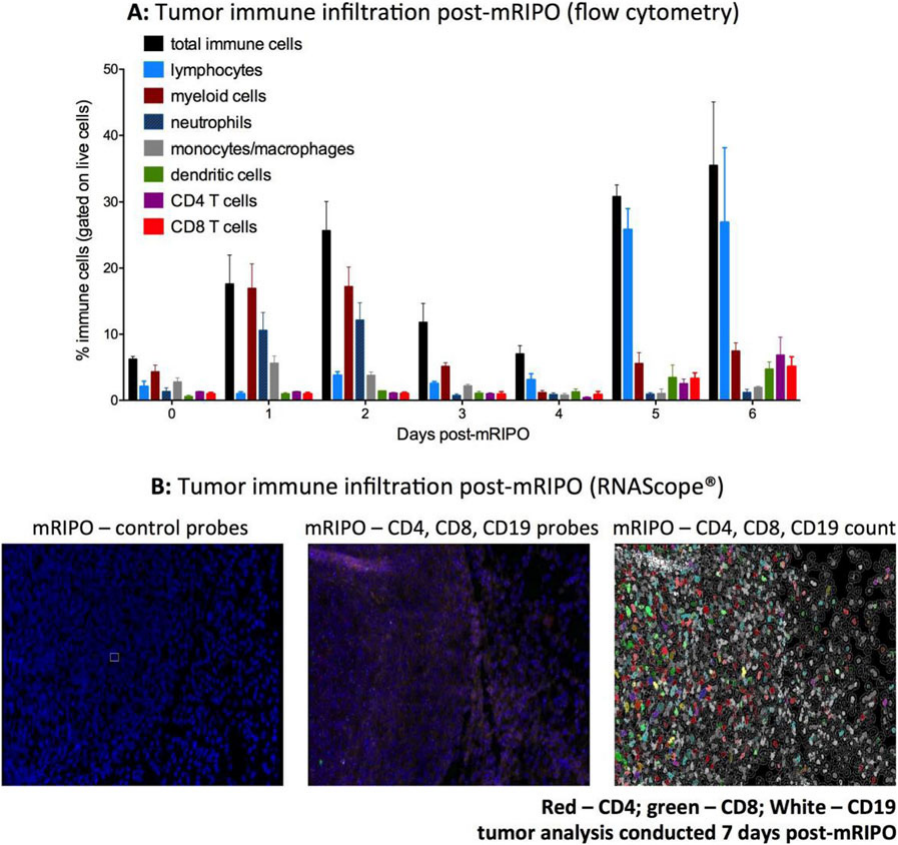
See text for description

**Fig. 2 (abstract P612).**
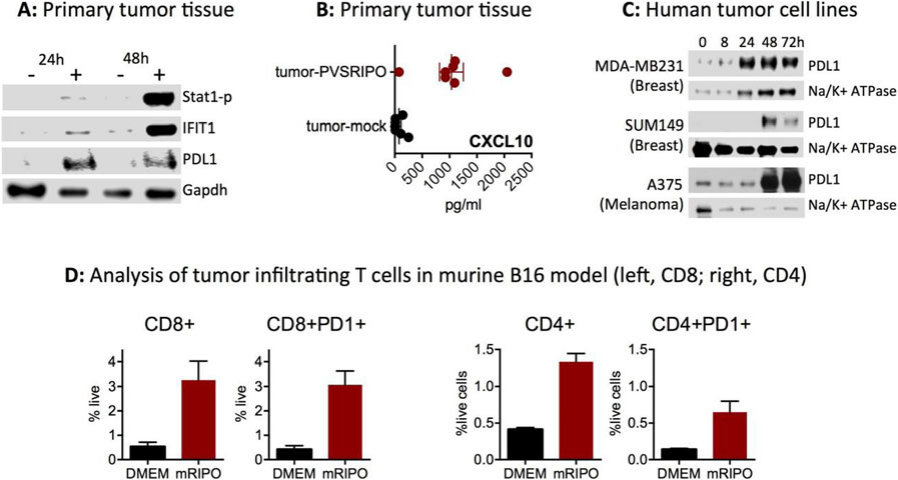
See text for description

#### P613 Non-oncolytic viral infection reduces tumor burden and promotes anti-tumor immunity in synergy with checkpoint blockade

##### Jenna Newman^1^, Charles Chesson, PhD^2^, Andrew Zolza^1^

###### ^1^Rutgers Cancer Institute of New Jersey, Colonia, NJ, USA; ^2^MD Anderson Cancer Center, Houston, TX, USA

####### **Correspondence:** Andrew Zolza (az255@cinj.rutgers.edu)

**Background**

Immunotherapy for cancer has had clinical success in recent years, with adoptive cell therapies and checkpoint blockade inducing long-term regression in an unprecedented subset of patients. Despite success, a significant fraction of patients are resistant to immunotherapy, prompting investigation into alternative strategies to initiate anti- tumor immunity. One approach that has had clinical impact is the intratumoral administration of oncolytic viruses, such as the recently FDA-approved T-VEC. While oncolytic viruses have shown efficacy in promoting cell lysis, tumor cell antigen release, and ultimately initiating an anti-tumor CD8+ T cell response, little is known regarding the impact of non-oncogenic, non-oncolytic viruses on tumor growth and immunity. Therefore, we sought to characterize the impact of influenza, a non-oncolytic virus, on tumor growth and initiation of anti-tumor immune responses in a B16 murine melanoma model.

**Methods**

C57BL/6 mice were challenged with 120,000 cells B16 F10 intravenously to allow for development of melanoma foci in the lungs. Five days following tumor challenge, mice were administered 1 × 106 pfu of A/PR8/H1N1 influenza intranasally. Body weight was recorded every 2-3 days to assess the health of influenza-infected mice. Tumor size was monitored every 2-3 days via caliper measurement. Mice receiving PD-1 blockade were treated with 250 μg α-PD-1, or isotype control antibody, every 3 days. Mice were sacrificed at days 7 and 14 after tumor challenge; tissues were harvested for flow cytometry and LEGENDPlex™ analysis.

**Results**

Mice concomitantly challenged with influenza and melanoma in the lungs exhibited a decrease in the number of melanoma foci in the lungs, relative to that observed in influenza-naïve counterparts (p<0.05). Synergy between influenza infection and checkpoint blockade was observed; influenza-infected tumor-bearing mice treated with PD-1 blockade exhibited the lowest tumor burden of all groups. Influenza-infected, melanoma-bearing mice exhibited a significantly higher proportion of gp100-reactive (anti-tumor) CD8+ T cells in the lungs, than that observed in uninfected controls (p<0.05). PD-1 blockade-treated influenza-infected mice exhibited an elevated proportion of anti-tumor CD8+ T cells relative to counterparts administered an isotype control antibody. Furthermore, LEGENDPlex™ analysis revealed an elevation in the pro-inflammatory cytokine IL-12 (p40) in the lungs of infected mice, compared to that observed in influenza-naïve, tumor-bearing mice, and that of influenza-infected mice without tumors.

**Conclusions**

Our results suggest that a non-oncogenic, non-oncolytic viral infection, influenza, can induce reduction of tumor growth and generation of anti-tumor immunity when administered at the site of a tumor. Further research will address potential therapeutic impact in humans.

#### P614 Nano-Pulse Stimulation™ of murine melanoma and mammary carcinoma is a physical modality that eliminates the treated tumor by regulated cell death and induces innate and adaptive immune responses

##### Richard Nuccitelli, MS, PhD^1^, Amanda McDaniel, BA^2^, Bruce Freimark, PhD^2^, Joel Benjamin, PhD^2^, Jessica Sood, BS^2^, Darrin Uecker, MS^2^

###### ^1^Pulse Biosciences Inc., Hayward, CA, USA; ^2^Pulse Biosciences, Burlingame, CA, USA

####### **Correspondence:** Richard Nuccitelli (rnuccitelli@pulsebiosciences.com)

**Background**

Nano-Pulse Stimulation (NPS™) is a non-thermal, precise, focal tissue treatment technology comprised of nanosecond (billionth of second)-range pulsed electric energy that directly affects the cell membrane and intracellular structures, and initiates regulated cell death in treated cells. NPS has been shown in preclinical models to induce immunogenic cell death (ICD) which exposes the unique antigens of the treated cells to the immune system and recruits immune system cells to mount an adaptive immune response [1, 2, 3].

**Methods**

Two syngeneic murine tumor models (B16-F10 melanoma and 4T1 mammary carcinoma) were used to investigate NPS effects on murine tumors. Tumor cells were injected intradermally on the left flank and when tumors reached 4-5 mm in diameter, we treated with NPS. Tumor volume measurements were conducted twice per week and tumors were excised at predetermined timepoints for immunophenotyping of tumor-infiltrating immune cells by flow cytometry and gene expression profiling using NanoString. Splenocytes were also evaluated for responses to tumor cell stimulation by IFN-gamma ELISpot.

**Results**

NPS-treated tumors undergo regulated cell death and are eliminated within 2 weeks providing complete local control. In addition, ELISpot analysis of splenocytes indicates that NPS initiates an immune response to the treated tumor by 4 weeks after treatment. Gene expression profiling of NPS-treated tumors revealed that transcripts for specific intrinsic apoptotic pathways, damage-associated molecular patterns (DAMPs) and immune mediators increase 24 h after NPS treatment. Splenocytes from tumor-bearing animals treated with NPS have greater IFN- gamma-secreting cells compared to naive or resected animals in response to co-culture with tumor cells. Studies to determine the optimal conditions for NPS treatment are underway.

**Conclusions**

NPS is a physical modality that targets intracellular structures to trigger regulated cell death in the treated tumor cells. Our studies indicate that this treatment initiates endoplasmic reticulum stress and immunogenic cell death (ICD) that leads to a tumor-specific immune response.

**References**

1. Nuccitelli R, Berridge JC, Mallon Z, Kreis M, Athos B, Nuccitelli P. Nanoelectroablation of murine tumors triggers a CD8-dependent inhibition of secondary tumor growth. PLoS ONE. 2015; 10(7): e0134364 1-17.
2. Nuccitelli R, McDaniel A, Anand S, Mallon Z, Berridge JC, Uecker D. Nano-Pulse Stimulation is a physical modality that can trigger immunogenic tumor cell death. J Immunotherapy Cancer. 2017; 5:32 DOI 10.1186/s40425- 017-0234-5.
3. Skeate JG, DaSilva DM, Chavez-Juan E, Anand S, Nuccitelli R, Kast W.M. Nano-Pulse Stimulation induces immunogenic cell death in human papillomavirus-transformed tumors and initiates an adaptive immune response. 2018; PLoS ONE. 13(1): e0191311.

#### P615 Antibody-armed oncolytic Vaccinia virus to block immunosuppressive pathways in the tumor microenvironment

##### Eric Quemeneur, PharmD, PhD, Jean-Baptiste Marchand, pHD

###### Transgene S.A., Illkirch-Graffenstaden, France

####### **Correspondence:** Eric Quemeneur (quemeneur@transgene.fr)

**Background**

Vaccinia virus (VV) has proven to be a powerful oncolytic vector thanks to its large spectrum of tumor cell targets, large genome capacity, good safety properties, and strong immunogenic properties. We have developed an improved VV-based platform in the Copenhagen strain, with a double deletion in the J2R/TK, and I4L/RR genes, that displays a very high therapeutic index (over 104), compatible with the intravenous route. VVTK-RR- can be genetically modified to express full-length monoclonal antibodies, and additional cytokines, at the site of active viral replication and accumulation, i.e. directly in the tumor. Thus, checkpoint blockers can be targeted to boost the immune response locally initiated by the oncolytic activity. Moreover, the benefit of vectorizing a mAb would be to diminish the severe side effects reported for some systemic administration, and to increase the intratumoral antibody concentration to maximize the probability of efficacy. Our first products addressed the two major inhibitory pathways : a VV-anti-PD-1 to restore the activity of infiltrated effector T cells, and a VV-anti-CTLA-4 to selectively deplete intratumoral regulatory T cells via ADCC.

**Methods**

Mice bearing syngeneic tumors were treated with oncolytic VV either alone or in combination with immune checkpoint inhibitors (anti-mCTLA4, and anti-mPD1). In the POC experiment, the anti-mPD1 was vectorized in VVTK-RR- as two independent cassettes at the J2R locus expressing the light and the heavy chains under strong viral promoters. The level of expression of the vectorized mAb was monitored over time after intra-tumoral injection and in blood stream of tumor-bearing mice.

**Results**

Combination experiments in immunocompetent preclinical models demonstrated that oncolytic VV and ICI can have at least additive anti-tumoral activities if administrated sequentially (i.e. VV first and ICI several days later). The vectorization of a mAb allowed its intratumoral expression with a kinetics that mimicked the schedule of administration of the combination (peak of tumor accumulation at days 3-5). The expression lasted for several days after administration of the virus, allowing for a similar anti-tumoral activity as repeated administrations of the reference mAb. Finally, unlike the combination, the vectorization resulted in a minimal systemic exposure to the ICI.

**Conclusions**

Vectorization in an oncolytic VV allows the intratumoral delivery of an active doses of therapeutic antibody. This recombinant platform is particularly relevant for ICIs with unfavorable toxic profiles such as anti-CTLA4 mAb. BioInvent and Transgene are currently developing the next generation of oncolytic viruses by arming VV with Fc−optimized anti-CTLA4 mAbs.

#### P616 Fueling antitumor immunity using oncolytic viruses encoding metabolic modulators

##### Dayana Rivadeneira, PhD, Kristin DePeaux, Nicole Scharping, BS, Padmavathi Sampath, PhD, Saumendra Sarkar, PhD, Stephen Thorne, PhD, Greg Delgoffe, PhD

###### University of Pittsburgh, Pittsburgh, PA, USA

####### **Correspondence:** Greg Delgoffe (delgoffeg@upmc.edu)

**Background**

Immunotherapy has shown impressive clinical responses, but many patients do not respond to single modality immunotherapy due to a number of non-redundant resistance mechanisms. Our lab and others have proposed that tumor cells compromise T cell function by generating a metabolically inhospitable microenvironment, suggesting that immune or tumor metabolism can be differentially modified to improve T cell responses and thus immunotherapy.

**Methods**

We identified the adipokine leptin as a means to remodel the metabolic state of tumor infiltrating T cells. To assess the effects of leptin in the tumor microenvironment, we generated an aggressive PTEN/BRAF melanoma line overexpressing leptin, as well as an oncolytic strain of Vaccinia virus engineered to induce tumor-specific secretion of leptin.

**Results**

Treatment of T cells with leptin in vitro resulted in dramatic metabolic reprogramming. In vivo, intratumoral administration of leptin resulted in enhanced T cell metabolic and effector function. We then engineered melanoma cell lines to locally secrete leptin. While there was no proliferation difference between wild-type and leptin- expressing tumor cells in vitro, these cells are controlled in vivo in a CD8+ T cell specific manner. Leptin overexpressing tumors have increased T cell infiltration compared to control tumors, and these TIL are metabolically and functionally superior. In order to translate out findings to a therapeutic setting we utilized an oncolytic virus model. Oncolytic viruses are an attractive therapeutic modality promoting tumor specific killing as well as inducing an anti-tumor immune response. While wild-type oncolytic Vaccinia resulted in some tumor regression, leptin-engineered Vaccinia had superior therapeutic efficacy inducing complete regressions in 30% of mice. TIL from these tumors have improved T cell infiltration and function. We profiled immune infiltrates by single cell RNAseq and TCR sequencing. Data revealed the influx of new T cells by vaccinia which was characterized by a polyclonal repertoire. On the other hand, T cells from tumors treated with leptin-expressing virus showed a reduced polyclonal phenotype indicative of specific clonal expansion. This clonal expansion is associated with a more memory like state, and indeed leptin-engineered VV induced a greater percentage of CD127hi memory precursors than the oncolytic VV alone.

**Conclusions**

Taken together, these data suggest metabolic modulators like leptin can be therapeutically exploited to bolster intratumoral T cell function using the oncolytic virus platform. Our goal is to further design novel therapeutic strategies using oncolytic viruses.

#### P617 A cell-based platform to protect and enhance oncolytic virus therapies

##### Antonio Santidrian, Dobrin Draganov, PhD, Duong Nguyen, PhD, Okyay Kilinc, Ivelina Minev, MSc, Boris Minev, MD, Aladar Szalay, PhD

###### Calidi Biotherapeutics

####### **Correspondence:** Antonio Santidrian (asantidrian@calidibio.com)

**Background**

Different types of viruses, including vaccinia virus (VACV), can selectively replicate in cancer cells and trigger antitumor immunity. Oncolytic virotherapies, as a monotherapy or in combination with other immunotherapeutics, have shown safety and exciting proof-of-concept results in pre-clinical studies as well as in different clinical trials. The therapeutic potential of oncolytic viruses, however, can be severely restricted by multiple innate and adaptive immune barriers that can be overcome using cell-based delivery approaches. Mesenchymal stem cells are particularly attractive carriers of oncolytic viruses due to their unique immunosuppressive properties allowing protection of the virus from complement/antibodies-mediated neutralization and to overcome anti-viral cellular immunity in both autologous and allogeneic settings

**Methods**

As carriers of oncolytic VACV, we used cells a) freshly isolated from adipose tissue stromal vascular fraction (SVF), and b) SVF-derived cultured Adipose-Derived Mesenchymal Stromal/stem Cells (AD-MSC). We analyzed the ability of those carrier cells to take up, protect, amplify the virus as well as to overcome innate and adaptive immune barriers by flow cytometry, microscopy and virus plaque assays of ex vivo co-cultures of cells infected with VACV in the presence of human serum or peripheral blood mononuclear cells from healthy donors. Comparative analyses were performed to establish statistically significant correlations.

**Results**

We have demonstrated that autologous SVF cells did protect VACV against serum-inactivation. Cell sorting demonstrated that supra adventitial-adipose stromal cells (SA-ASC; CD235a-/CD45-/CD34+/CD146-/CD31-), and pericytes (CD235a-/CD45-/CD34-/CD146+/CD31-) were the two cell populations of SVF cells that were efficient facilitating the delivering of VACV to the tumor cells, validating their clinical use as a tool to potentiate oncolytic virus therapies in autologous settings. We further analyzed the potential of using cultured AD-MSC (derived from CD34+ SA-ASC) as a delivery vehicle in allogeneic settings. AD-MSCs demonstrated ability to protect against serum-inactivation as well as to amplify the virus in the presence of human PBMCs in both autologous and allogeneic settings. This activity can be linked to their intrinsic immunosuppressive properties and the evasion of allogeneic rejection. Moreover, these cells demonstrated ability to provide transient immunosuppression by inhibiting antiviral responses originating from both innate (NK)- and adaptive (T)-immune cells, thus augmenting viral oncolysis and the generation of anti-tumor immunity.

**Conclusions**

Overall, our findings indicate the feasibility to significantly potentiate oncolytic virotherapy by using either a simple autologous or a more scalable off-the-shelf allogeneic cell-based delivery technology allowing rational design of virus-based therapies that are not dramatically eliminated by immune barriers.

**Ethics Approval**

The study was approved by International Cell Surgical Society Ethics Board; IRB# ICSS-2016-024

#### P618 Selective delivery of exosome-mediated STING agonist to antigen presenting cells results in significantly improved potency and reduced toxicity

##### Su Chul Jang, PhD^1^, Raymond Moniz^1^, Christine Sia^1^, Joyoti Dey, PhD, MPH^2^, Rane Harrison^1^, NIKKI ROSS, PhD^1^, Ke Xu^1^, Kevin Dooley^1^, Nuruddeen Lewis^1^, Christine McCoy^1^, Agata Villiger-Oberbek^1^, Scott Estes^1^, Jorge Sanchez-Salazar^1^, Kyriakos Economides^1^, Sriram Sathyanarayanan^1^

###### ^1^Codiak Biosciences, Cambridge, MA, USA; ^2^Presage Biosciences, Seattle, WA, USA; ^3^Codiak, Cambridge, MA, USA

####### **Correspondence:** Sriram Sathyanarayanan (sriram.sathy@codiakbio.com)

**Background**

Emerging research has established the role of exosomes as an efficient natural messenger system to deliver macromolecules between cells. We have leveraged this capacity to develop a novel, engineered exosome therapeutic, to selectively deliver agonists of the Stimulator of Interferon Gene (STING) pathway to tumor resident antigen presenting cells (APC).

**Methods**

ExoSTING is composed of exosomes, which are molecularly engineered to over-express an exosomal membrane glycoprotein, and which are loaded ex vivo with a STING agonist (SA).

**Results**

In vitro assays with human PBMCs showed ExoSTING enhanced the potency of dendritic cell and monocyte activation and IFN_beta production by 100-fold compared to comparable amounts of free SA. Although liposomal formulated SA also improved potency, it resulted in dose-dependent loss of viability in APC. Intra-tumoral micro- dosing of ExoSTING with the CIVO® platform demonstrated selective activation of pTBK1 and pIRF3 in APC resulting in superior IFN_beta production compared to free SA. Anti-tumor activity of Exo-STING and free SA was compared in a checkpoint therapy refractory B16F10 tumor model. Intra-tumoral (IT) administration of ExoSTING resulted in 500-fold enhancement in potency versus free SA, with dose-dependent anti-tumor activity resulting in tumor cures in 50% of the mice in the highest (0.2μg) ExoSTING dose cohort. ExoSTING treated mice were refractory to re-challenge with B16F10 demonstrating the presence of an immune memory response. IT administration of efficacious doses of ExoSTING stimulated robust IFN_gamma but did not result in systemic induction of inflammatory cytokines as seen with an efficacious dose of free SA. ExoSTING IT treatment induced IFNγ regulated genes, PD-L1 and chemokines responsible for T-cell recruitment in the tumor, resulting in significant systemic induction of tumor antigen-specific T cell response.

**Conclusions**

ExoSTING affords selective agonism of the STING pathway in tumor resident APC that results in improved potency, reduced systemic toxicity and enhanced T-cell responses, and highlight the potential of our exosome engineering technology as an impactful therapeutic platform.

#### P619 Image-guided intratumoral delivery of immunotherapeutics: interventional radiology perspective

##### Rahul Sheth, MD, Ravi Murthy, MD, Funda Meric-Bernstam, MD, David Hong, MD, Sapna Patel, MD, Alda Tam

###### MD Anderson Cancer Center, Houston, TX, USA

####### **Correspondence:** Alda Tam (alda.tam@mdanderson.org); Rahul Sheth

**Background**

There has been an exponential proliferation in the direct intratumoral (IT) delivery of a range of immunotherapies (IMT), ranging from cell-based therapies to viruses, bacteria, cytokines, and monoclonal antibodies. In this study, we report our single institutional experience with IT injections in the investigational, off-label, and standard-of-care settings in patients with solid tumors.

**Methods**

All patients who underwent image-guided IT delivery of IMT agents by Interventional Radiology over a 2 year period (Jan 2016 –18) were included in this single institution retrospective analysis. Lesion characteristics (size, location, concomitant biopsy) were recorded. Injection technique and adverse events related to needle insertion vs. drug delivery were identified by chart review.

**Results**

66 patients underwent 429 image-guided IT investigational agent injections; malignancies included melanoma (50%), sarcoma (21%), ovarian cancer (4.5%), breast cancer (3%) and colon cancer (3%), and other (18.5%). Additionally, 18 patients (9 cutaneous melanoma patients as standard-of-care, 9 uveal melanoma patients as off- label use) underwent 113 image-guided IT injections of TVEC. A tracer and fanning methodology was employed to optimize distribution within the lesion when using a single end-hole beveled needle. The median number of encounters per patient was 6 (range: 1-20) in the investigational setting and 5 (range: 1-17) for TVEC. Subcutaneous lesions represented 62% and 100% of injected tumors in the investigational and TVEC patients, respectively. Visceral lesions in deeper locations and solid organs were also injected: pelvic (6.7%), abdominal (6.2%), intramuscular (6.2%), adrenal (4.3%), liver (3.7%), and lung (2.4%) (Figure 1). The median target lesion tumor volume was 6.4cc (range: 0.1 – 984cc) in investigational patients and 3.5cc (range: 0.2 – 250cc) in TVEC patients. There were no adverse events related to needle insertion. However, serious adverse events NCI CTC >3 including dyspnea and rigors developing within 90 minutes of the injection and requiring hospitalization occurred following 2.4% of investigational agent and 3.5% of TVEC injections. NCI CTC AE<3 including allergic reactions that did not require an escalation of care occurred in 3.4% of clinical trial patients and 0.9% of TVEC patients.

**Conclusions**

Initial observations indicate that image guided IT injections of a variety of IMT agents are feasible for both subcutaneous as well as deeper, visceral organ-based lesions. Immediate post delivery anticipated adverse events occur in a small minority of instances. Performing physicians should have the necessary safeguards in place to respond as needed. Moreover, efforts to standardize drug delivery techniques may also be required.

**Ethics Approval**

This study was approved by our institution’s Ethics Board; approval number PA18-0650.

**Fig. 1 (abstract P619).**
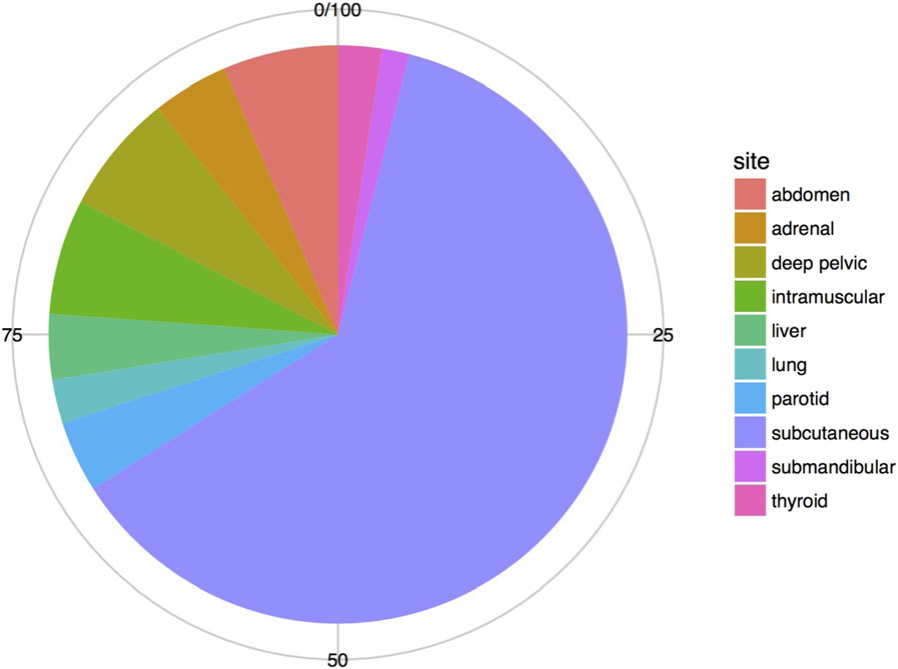
Intratumoral injection site

#### P620 Enhanced efficacy and combinability of low dose toca 511 and 5-FC with metronomic chemotherapy in preclinical models

##### Sophie Viaud, Derek Ostertag

###### Tocagen, San Diego, CA, USA

####### **Correspondence:** Derek Ostertag (dostertag@taocagen.com)

**Background**

Toca 511 (vocimagene amiretrorepvec) is an investigational, conditionally lytic, retroviral replicating vector that selectively infects cancer cells due to specificity for dividing cells combined with the immune-suppressed tumor microenvironment. Toca 511 spreads and stably delivers optimized yeast cytosine deaminase (CD) that converts subsequent Toca FC (an investigational, extended-release version of 5-fluorocytosine [5-FC]) into 5-fluorouracil (5- FU). 5-FU kills infected dividing cancer cells and, in preclinical models, local immunosuppressive myeloid cells leading to therapeutically active anti-tumor immunity. A similarly derived antitumor response may occur in cancer patients, as local injection of recurrent high grade gliomas with Toca 511 followed by treatment with Toca FC has been associated with prolonged survival and durable complete responses (median duration of follow-up for response: 37.4+ months); responses were delayed in onset, consistent with an immunological mechanism. Not all patients responded and, clinically, only portions of the tumors were infected. These data led to the currently enrolling phase III trial (NCT02414165). To model submaximal infections, and look for clinically-compatible synergistic treatments, we implemented a novel preclinical model. We used this model system to test the immuno-stimulatory and antiangiogenic properties of cyclophosphamide following a metronomic/low dose regimen and its previously reported ability to cross the blood-brain-barrier, in a combination therapy.

**Methods**

Naive B6C3F1 mice were implanted in the right flank with a mouse glioma cell line Tu-2449SQ that has been adapted to grow subcutaneously. Tu-2449SQ cells were first 100% infected in vitro with Toca 511 or a sister vector that expresses GFP (Green Fluorescent Protein) instead of CD. In order to control Toca 511 spread, these cells were then admixed at various percentages as Toca 511 does not readily infect cells already infected with the GFP virus. Controlling the percent of Toca 511-infected tumor cells allows the screening of potential drugs combinations for efficacy.

**Results**

We show a significant survival benefit with metronomic cyclophosphamide compared to controls with only 10% of tumor cells infected with Toca 511. This survival benefit is associated with a significant depletion of peripheral Tregs and increase in CD8+ T cells.

**Conclusions**

These data demonstrate that Toca 511 and 5-FC therapy can be combined with metronomic chemotherapeutics like cyclophosphamide to enhance efficacy in preclinical models when the percent of Toca 511-infected tumor cells is manipulated to be submaximal. Data from this study will inform future clinical development of Toca 511 & Toca FC in combination with other therapies.

#### P621 Local immunotherapy with a mixture of mRNAs encoding pro-inflammatory cytokines promotes potent anti- tumor immunity and tumor eradication across multiple preclinical tumor models.

##### Timothy Wagenaar^1^, Christian Hotz^2^, Friederike Gieseke^2^, Hui Cao^1^, Jan Diekmann^2^, Mustafa Diken^2^, Christian Grunwitz^2^, Andrew Hebert^1^, Karl Hsu^1^, Marie Bernardo^1^, Katalin Kariko, PhD^2^, Sebastian Kreiter^2^, Andreas Kuhn^2^, Mikhail Levit^1^, Natalia Malkova^1^, Serena Masciari, MD, MSc^1^, Jack Pollard^1^, Hui Qu^1^, Abderaouf Selmi^3^, Julia Schlereth^2^, Fangxian Sun^1^, Bodo Tillmann^3^, Tatiana Tolstykh^1^, Lena Wicke^2^, Sonja Witzel^3^, Qunyan Yu^1^, Yu-An Zhang^1^, Gang Zheng^1^, Gary Nabel^1^, Joanne Lager^1^, Ugur Sahin, MD^2^, Dmitri Wiederschain

###### ^1^Sanofi, Cambridge, MA, USA; ^2^BioNTech, Mainz, Germany; ^3^TRON, Mainz, Germany

####### **Correspondence:** Ugur Sahin (ugur.sahin@tron-mainz.de)

**Background**

Local administration of immunotherapies to the tumor microenvironment provides the opportunity to stimulate innate and adaptive immune responses against tumors, while avoiding toxicities related to systemic administration of immuno-modulatory therapeutics. Current strategies for tumor-targeted, gene-based delivery of immune therapies face limitations in the clinic due to suboptimal target expression, anti-vector immunity, or the potential for unwanted genomic rearrangements.

**Methods**

We examined the intratumoral administration of synthetic mRNA encoding immunomodulatory cytokines to provide sustained in vivo protein translation localized to the tumor microenvironment and limiting adverse effects associated with systemic administration of recombinant cytokines. Using iterative rounds of in vivo screening in murine syngeneic tumor models, a mixture of four synthetic mRNAs encoding bioactive versions of interleukin-12, interferon alpha, GM-CSF and interleukin-15 was identified which mediated complete tumor regression across multiple different tumor models.

**Results**

Mechanistically, maximal anti-tumor activity of cytokine mRNAs was associated with multiple immune populations including CD4+ and CD8+ T cells as well as NK cells. Localized administration of cytokine mRNA was accompanied by robust intratumoral induction of interferon gamma, systemic expansion of antigen-specific T cells and increased granzyme B positive CD8+ T cell infiltration. Immunological memory to both dominant and subdominant antigens was formed that protected long-term survivors from re-challenge with autologous tumors. Importantly, although cytokine mRNAs were administered locally, anti-tumor activity extended beyond the injected tumor to effectively control the growth of distant tumors in both a dual-tumor model and in an experimental lung metastasis model. Finally, the combination of mRNAs encoding interleukin-12, interferon alpha, GM-CSF and interleukin-15 cytokines with immunomodulatory antibodies enhanced the anti-tumor response in both injected and uninjected tumors leading to improved overall survival and higher incidence of complete tumor regressions across several preclinical models.

**Conclusions**

In summary, the robust and versatile synthetic mRNA platform reported herein was used to identify multi-modal localized cancer immunotherapy with broad anti-tumor activity against treated and untreated tumors.

#### P622 Phase 1/2 evaluation of intratumoral INT230-6 for the treatment of solid tumors

##### Anthony Olszanski, MD, RPh^1^, Nilofer Azad, MD^2^, Lewis Bender, MS, MA, MBA^3^, Ian Walters, MD^3^, Diana Hanna, MD^4^, Jacob Thomas, MD^5^, Lillian Siu, MD^6^, Anthony El-Khoueiry, MD^5^

###### ^1^Fox Chase Cancer Center, Phildelphia, PA, USA; ^2^Johns Hopkins, Chevy Chase, MA, USA; ^3^Intensity Therapeutics, Westport, CT, USA; ^4^University of Southern California, Hoag, Los Angeles, CA, USA; ^5^University of Southern California, Norris, Los Angeles, CA, USA; ^6^Princess Margaret Cancer Center, UHN, Toronto, Canada

####### **Correspondence:** Ian Walters (iwalters@intensitytherapeutics.com)

**Background**

INT230-6 is a novel formulation of cisplatin and vinblastine with an amphiphilic cell penetration enhancer designed specifically for intratumoral administration. It is designed to improve dispersion throughout tumors and facilitate diffusion specifically in cancer cells while sparing healthy cells. In colon 26 animal models, injection into large primary lesions led to substantial tumor necrosis, recruitment of dendritic cells, and engagement of CD4 and CD8 T- cells. Injected tumors experienced high rates of complete response (up to 80%). Importantly, untreated lesions, distal to the injection site, responded and CR animals developed protection from re-challenge. Tumor growth control in injected and bystander tumors and survival improved significantly when INT230-6 was combined with checkpoint inhibitors.

**Methods**

This study is a phase 1/2 evaluation of multiple doses of INT230-6 injected into ≥ 1 superficial or deep tumor(s). The initial cohort treated superficial lesions at low dose once a month x 5. Upon establishing safety, subsequent sequential cohorts opened including a deep tumor cohort of once monthly injections, a superficial cohort of every 2 week dosing, and a cohort treating both deep and superficial tumors every 2 weeks utilizing a higher drug volume and dose load. A multiprong needle was utilized for injecting large tumors. Pharmacokinetic (PK) and pharmacodynamics (PD) samples (for flow cytometry and circulating cytokines) were collected.

**Results**

Fifteen heavily pretreated subjects have been dosed. Median age was 59 (46-72), ECOG 0:1 2:13, 87% Caucasian, 67% Female. 7/15 Subjects had received prior checkpoint antibodies. Cancer types include melanoma, SCC, ovarian, chordoma, cholangiocarcinoma, sarcoma, rectal, thyroid and H&N. Dosing has been into single or multiple lesions. PK analysis reveals negligible amounts of cisplatin and vinblastine in blood, suggesting the majority of INT230-6’s active agents are retained in the tumor (consistent with no observable systemic AE’s). No DLTs or drug-related SAEs were reported. The most frequent adverse events were low grade pain (26% of patients) and swelling/edema (20%). Many injected tumors showed visible necrosis or decreased contrast uptake. Patients dosed every two weeks remained on treatment longer than those treated once a month. 5 out of 11(45%) evaluable patients had increases in circulating CD8+ T-cells, of these 60% also had increases in circulating CD4+ T-cells.

**Conclusions**

INT230-6 is safe when administered intratumorally at doses given to date. Updated results will be presented, including biomarker and response data. Additional planned cohorts include higher drug concentrations and combination with an anti-PD1 antibody.

**Trial Registration**

NCT: 03058289

**Ethics Approval**

The study was approved by Fox Chase Cancer Center, USC, John's Hopkins, and Princess Margaret Cancer Centers Institutution‘s Ethics Boards

**Consent**

Written informed consent was obtained from the patient for publication of this abstract and any accompanying images. A copy of the written consent is available for review by the Editor of this journal.

#### P623 The oncolytic peptide LTX-315 promotes natural killer cell recruitment and reduces lung metastatic burden in combination with local radiation therapy

##### Erik Wennerberg, PhD^1^, Takahiro Yamazaki, PhD^1^, Baldur Sveinbjornsson, PhD^2^, Oystein Rekdal, PhD^2^, Lorenzo Galluzzi, PhD^1^, Sandra Demaria, MD^1^

###### ^1^Weill Cornell Medicine, New York, NY, USA; ^2^Lytix Biopharma, Tromso, Norway

####### **Correspondence:** Erik Wennerberg (erw2010@med.cornell.edu)

**Background**

Cancer patients who lack tumor infiltration by T cells are poorly responsive to immune checkpoint blockade (ICB), prompting efforts to identify immunomodulating strategies to convert T cell-poor into T cell-rich tumors. The oncolytic peptide LTX-315 perturbs the mitochondrial membrane of cancer cells, culminating in the induction of immunogenic cell death (ICD) [1]. Recruitment of XCR1+ dendritic cells (DCs) to the tumor is critical for priming of anti-tumor CD8+ T cells following induction of ICD, and this process can be regulated by natural killer (NK) cells [2]. Here, we tested the interaction of focal radiotherapy (RT) with LTX-315 and their ability to trigger local and systemic anti-tumor immune responses.

**Methods**

BALB/c mice were injected subcutaneously with syngeneic 4T1 tumor cells, a mouse model of triple-negative breast cancer that spontaneously metastasizes to the lung. When average tumor volume reached 70 mm3, mice were randomized and treated on three consecutive days with: [1] LTX-315 (0.3 μg) or [2] saline (both intratumorally), [3] 8 Gy focal RT, [4] LTX-315+RT. Some mice were sacrificed 6 days after treatment for analysis of lymphocytes and DCs in primary tumors and lungs. Remaining mice were followed for tumor growth, and lung metastases were evaluated 23 days after treatment initiation.

**Results**

Monotherapy with LTX-315 significantly inhibited tumor growth (p<0.001) and there was a trend towards improved tumor growth inhibition achieved by RT. Lung metastasis were significantly reduced only in mice treated with the combination of RT+LTX-315 (17±12.8 in mock-treated v. 1.5±1.2 in mice treated with LTX-315+RT, p<0.05). The percentage of XCR1+ DCs was increased in the tumor of mice treated with LTX-315+RT compared to mice treated with saline, LTX-315 alone or RT alone (8.4±2.4% in LTX-315+RT (p<0.05) v. 4.2±0.4% in RT v. 5.7±0.8% in LTX-315 v. 4.1±0.7% in mock-treated). Interestingly, LTX-315 enriched NK cells among leukocytes in both primary tumors and whole lung (tumor: 1.1±0.3% in mock-treated v. 3.5±1.2% in LTX-315 (p<0.01), lung: 2.1±0.3% in mock-treated v. 5.1±0.4% in LTX-315 treated (p<0.0001).

**Conclusions**

In conclusion, our data show that local LTX-315 therapy delays tumor growth and potentiates systemic anti-tumor effects in combination with RT. Importantly, LTX-315 cooperates with RT to promote tumor infiltration by XCR1+ DCs, which are essential for induction of anti-tumor immunity. LTX-315-driven enrichment of NK cells in tumors and lungs from tumor-bearing mice may play an important role in systemic tumor control. LTX-315 represents a promising agent for the treatment of patients with poorly immunogenic and metastatic cancers.

**References**

1. Zhou H, Forveille S, Sauvat A, Yamazaki T, Senovilla L, Ma Y, Liu P, Yang H, Bezu L, Muller K, Zitvogel L, Rekdal O, Kepp O, Kroemer G. The oncolytic peptide LTX-315 triggers immunogenic cell death. Cell Death Dis, 2016; 7: e2134.
2. Barry KC, Hsu J, Broz ML, Cueto FJ, Binnewies M, Combes AJ, Nelson AE, Loo K, Kumar R, Rosenblum MD, Alvarado MD, Wolf DM, Bogunovic D, Bhardwaj N, Daud AL, Ha PK, Ryan WR, Pollack JL, Samad B, Asthana S, Chan V, Krummel MF. A natural killer-dendritic cell axis defines checkpoint therapy-responsive tumor microenvironments. Nat Med. 2018; [Epub ahead of print]

#### P624 Development of a STING agonist-producing synthetic Biotic™ medicine to activate innate and adaptive immunity and drive antitumor immune responses

##### Kip West, PhD, Kip West, PhD, DANIEL LEVENTHAL, PHD, Ning Li, PhD, Christopher Plescia, MS, Starsha Kolodziej, BS, Rudy Christmas, BS, Carey Gallant, BS, Michael James, BS, Adam Fisher, PhD, Anna Sokolovska, PhD, Paul Miller, PhD, Jose Lora, PhD

###### Synlogic, Cambridge, MA, USA

####### **Correspondence:** Jose Lora (jose@synlogictx.com)

**Background**

Engagement of both the innate and adaptive arms of the immune system has been shown to be critical in generating an efficacious anti-tumor immune response. Recent studies demonstrate that activation of the stimulator of interferon genes (STING) pathway plays an essential role in initiating anti-tumor immunity through activation of antigen presenting cells (APCs), production of type I interferon and subsequent T cell priming and tumor-specific T- cell-responses. Bacteria may provide an ideal mechanism for STING activation as they can be deployed within the tumor microenvironment (TME), are engulfed by APCs and activate parallel pathways of innate immunity that may potentiate the interferon response.

**Methods**

Using synthetic biology we introduced an anaerobically inducible di-nucleotide cyclase gene into our probiotic chassis, E. coli Nissle (EcN), to generate a bacterial strain capable of efficient production of the STING agonist cyclic-di-AMP (CDA) in response to the hypoxic TME, which we refer to as SYN-STING. We then employed a suite of cell-based assays and mouse tumor models to evaluate the activity of SYN-STING in vitro and in vivo.

**Results**

In in vitro assays, SYN-STING generated high levels of CDA and triggered expression of IFN-beta when co-cultured with both mouse and human APCs. In syngeneic tumor-bearing mice, intra-tumoral administration of SYN- STING resulted in an early rise of innate cytokines which later shifted towards molecules indicative of an effector- T-cell response. These pharmacodynamic changes correlated with increased immune infiltrate, robust anti-tumor responses and complete tumor regressions. We also observed increased tumoral innate cytokine levels and anti- tumor activity in response to treatment with the un-engineered EcN, supporting that our bacterial chassis itself is able to stimulate innate immunity in the TME, and this feature is further potentiated by arming it with STING agonist. Additionally, mice that exhibited complete regressions in response to SYN-STING treatment demonstrated long-term immunological memory when re-challenged with tumor cells >40 days post tumor eradication. Finally, administration of SYN-STING, singly or in combination with antibodies targeting co-stimulatory molecules, led to abscopal effects with significant anti-tumor activity observed in both injected and non-injected lesions.

**Conclusions**

Taken together, these results demonstrate that a Synthetic Biotic medicine designed to specifically deliver STING agonist locally within the TME leads to significant anti-tumor activity, systemic immunity and long-term immunological memory in mouse tumor models. Moreover, the ability of our platform to engage multiple innate immune pathways simultaneously further supports the development of Synthetic Biotic medicines for cancer- immunotherapy in humans.

### Other

#### P625 Outcomes with first-line PD-1/PD-L1 inhibition in advanced urothelial cancer (UC): A single institution experience

##### Arjun Balar, MD^1^, Miles Hsu, BS^2^, Yuhe Xia^3^, Andrea Troxel, PhD^3^, Daniela Delbeau, RN^3^, Kaitlyn Francese, RN^3^, Dayna Leis, RN^3^, Deneuve Shepherd^3^

###### ^1^Perlmutter Cancer Center - New York Univ, New York, NY, USA; ^2^NYU School of Medicine, New York, NY, USA; ^3^NYU Langone Health, New York, NY, USA

####### **Correspondence:** Arjun Balar (arjun.balar@nyumc.org)

**Background**

First-line PD-1 checkpoint inhibition (CI) in cisplatin-ineligible advanced UC represents a new treatment standard based on single arm trials [1,2], leaving uncertainty regarding role of chemotherapy. Describing utilization and corresponding outcomes with second-line treatment will provide guidance in this new sequence. We present the outcomes of these patients treated at our institution.

**Methods**

43 patients with advanced UC received 1st-line CI from 6/2014 – 6/2018 on or off protocol. Clinical, laboratory and imaging data within 30 days of 1st-line initiation were gathered and clinical outcomes were analyzed including response by RECIST v1.1 and survival (OS). Disposition and treatment post- CI were also analyzed. Clinical outcomes were analyzed for the entire study population as well as known prognostic subgroups. A multivariable analysis was used to determine the prognostic value of baseline factors, and a log rank test was used to compare outcomes in prognostic subgroups.

**Results**

43 patients were treated with 1st-line CI (atezolizumab or pembrolizumab) from 6/2014 until 6/2018. Median age was 77 (range 34 - 89), (74% male) (26% prior BCG), (60% visceral metastases, 19% liver), (reason for cisplatin ineligibility: ECOG PS =2 30%, Impaired renal function 44%, both 21%). ORR to first-line CI was 30.2% (95% CI 28% – 32%), CR 14%. Median OS and PFS was 11.7 mos (95% CI 7.6 – 19.8) and 3.0 mos (95% CI 2 - 11.2), respectively (median follow up 11.7 mos). OS was negatively correlated with visceral metastases at baseline. Of 29 patients who progressed, 17 received 2nd-line treatment (71% chemotherapy (most commonly Gem/Carbo (10 pts)) or 29% immunotherapy). Patients on chemotherapy had an RR of 38.46% while those on immunotherapy had an RR of 20.0%. Combined, 2nd-line treatment resulted in a median OS of 6.2 months (95% CI of 2.9-12.66), an ORR of 11.1% (95% CI of 8.0% to 15.0%) and an RR of 33.3% (95% CI of 28% - 39%).

**Conclusions**

In our single institution experience, OS and ORR with 1st-line CI are similar to outcomes reported in single arm trials. RR to 2nd-line chemotherapy is comparable to historical rates with gem/carbo in the first-line, however 12 of 29 progressing patients did not receive 2nd-line treatment, highlighting the importance of patient selection for first- line CI. Outcomes by PD-L1 status will be presented.

**References**

1. Balar AV, Galsky MD, Rosenberg JE, Powles T, Petrylak DP, Bellmunt J, et al. Atezolizumab as first-line treatment in cisplatin-ineligible patients with locally advanced and metastatic urothelial carcinoma: a single-arm, multicentre, phase 2 trial. Lancet. 2017 Jan 7;389(10064):67-76. doi: 10.1016/S0140-6736(16)32455-2. PMID: 27939400
2. Balar AV, Castellano D, O’Donnell PH, Grivas P, Vuky J, Powles T, Plimack ER, Hahn NM, de Wit R, Pang L, Savage MJ, Perini RF, Keefe SM, Bajorin DF, Bellmunt J. First-line Pembrolizumab in cisplatin-ineligible advanced urothelial cancer: results of the Phase 2 KEYNOTE-052 study. Lancet Oncol. 2017 Nov; 18(11):1483-1492. doi: 10.1016/S1470-2045(17)30616-2. Epub 2017 Sep 26.

#### P626 Overcoming target-driven fratricide for CAR-T cell therapy

##### Eytan Breman, MSc, Benjamin Demoulin, Sophie AGAUGUE, PhD, Sebastien Mauen, PhD, Alexandre Michaux, PhD, Lorraine Springuel, Fanny Huberty, Céline Jacques-Hespel, Celine Marchand, Laboratory Technician, Jérôme Marijsse, master, Peter DeWaele, David Gilham, PhD, Valérie Steenwinckel, PhD

###### Celyad, Mont-St-Guibert, Belgium

####### **Correspondence:** Eytan Breman (ebreman@celyad.com)

**Background**

Chimeric Antigen Receptor (CAR) T cells expressing the fusion of the NKG2D protein with CD3ζ (termed CYAD-0 cells) acquire a specificity for eight ligands, the UL16 Binding Protein 1-6 (ULBP1-6) and MHC class I chain- related A and B (MICA and MICB). These stress-induced ligands are expressed on multiple cancers, while they are absent from most normal tissues, rendering them an interesting target for cancer therapy. However, these stress ligands are also transiently expressed by activated T cells, implying that CYAD-01 cells may undergo self-killing (fratricide) during production. Since NKG2D potentially targets eight individual ligands, genetic editing to avoid T cell fratricide is currently challenging. Therefore, alternative strategies are required to enable the production of CYAD-01 CAR-T cells.

**Methods**

CYAD-01 cells were manufactured by isolating PBMCs from healthy donors and activating the T cells with IL-2 and OKT3 for 2 days. Cells were then transduced with the CAR construct (or control truncated CD19) for two days and expanded for an additional 4-6 days. The broad Phosphoinositol-3-Kinase inhibitor (LY294002) was added following the two-day transduction. Alternatively, or in combination, blocking anti-CD314 antibody was added at the expansion phase.

**Results**

The first approach to inhibit NKG2D-mediated fratricide focused upon the inclusion of LY294002 into the production process. A second strategy involved the inclusion of antibody blockade of NKG2D itself. Both processes impacted T cell fratricide, albeit at different levels. The antibody process was the most effective in terms of cell yield, with a T cell phenotype closely resembling the control condition, while the LY294002 process impacted both T cell proliferation and T cell phenotype. Nonetheless, both approaches generated highly potent CYAD-01 cells. Interestingly, the differing T cell populations could be tweaked through the phased combination of LY294002 and blocking antibody into a single process. The combined approach led to a high yield of potent CYAD-01 CAR T cells. CYAD-01 CAR T cells are currently part of the phase I THINK clinical trial (NCT03018405) where clinical objective responses have been observed.

**Conclusions**

These results indicate that target-driven fratricide can be overcome using clinically relevant approaches where technologies such as gene-editing may be more challenging.

#### P627 From staining to analysis: fully automated workflow for multiplexed immuno-profiling in FFPE tumor samples using UltiMapper™ reagent kits

##### Amanda Bares, PhD, Michael Murphy, Heike Boisvert, PhD, Katir Patel, PhD, Bonnie Phillips, PhD, Sean Downing, PhD, Mael Manesse, PhD

###### Ultivue, Cambridge, MA, USA

####### **Correspondence:** Sean Downing (sean.downing@ultivue.com)

**Background**

The success of personalized medicine hinges on translating the biological complexity of a disease into actionable information. To achieve this goal, high-content data generation and analysis is required, covering both the detection of biomarkers as well as understanding their spatial interaction. In the case of oncology, understanding the protein expression profile in the tumor microenvironment is the basis for the development of new and improved translational research and future companion diagnostic tools. To enable this effort, multiplex immunohistochemistry (mIHC) methods have been established to provide insights into a wide number of markers of interest and their spatial context in a single sample. We have developed a fully automated approach to enable high-multiplexing detection of protein markers in tumor samples using an integrated workflow compatible with a range of autostainers and fluorescence whole slide scanners.

**Methods**

UltiMapper reagent kits were used to stain formalin-fixed, paraffin-embedded (FFPE) samples from human tonsil and primary tumor biopsies on automated staining platforms such as the Leica BondRX autostainer. The kits contain a cocktail of primary antibodies of interest, modified with unique, addressable DNA barcodes, applied to tissue samples in a single staining step. A cocktail of fluorescently labeled oligonucleotides is then used to tag the targets of interest for multiplexed imaging. Images were acquired using commercially available fluorescence whole slide scanners and analyzed using the Indica Labs HALO software.

**Results**

The UltiMapper kits alleviate lengthy assay development efforts by offering a convenient, optimized assay kit with a simplified workflow. The staining workflow includes a single staining step for all targets which eliminates the need to optimize staining order and solves issues of tissue damage during cyclical staining steps. Fully automated staining protocols were developed on autostainers to enable high throughput and highly reproducible results. The optimized protocols were used to stain a series of FFPE tumor sections and perform multiplexed quantification analysis of immune cell phenotypes in tumor samples.

**Conclusions**

The workflow of the InSituPlex technology enables multiplexed immune-profiling in tumor FFPE sections though a highly streamlined, automated workflow with existing high-throughput staining and imaging instrumentation.

#### P628 High dimensional immune cell profiling with data-independent acquisition mass spectrometry

##### Jakob Vowinckel, PhD, Tobias Treiber, Nicholas Dupuis, PhD, Kristina Beeler, Claudia Escher

###### Biognosys, Zurich, Switzerland

####### **Correspondence:** Nicholas Dupuis (nicholas.dupuis@biognosys.com)

**Background**

Recent successes with therapies directed at the immune system have demonstrated the utility of targeting the immune response for control of multiple cancers. These successes have also spurred interest in characterizing immune cell sub-populations to understand mechanisms of activation and suppression, and their relationship to therapeutic response. Currently, antibody-based approaches are commonly used to characterize immune cells, however these methods are limited to 30-40 markers from previous hypotheses, limiting new discovery. In this context, determination of the surface and cellular proteome of responsive populations will provide a powerful tool for insight into response mechanisms, so far hampered by low sample availability and limited sensitivity of proteomic methodology. Here, we demonstrate how data-independent acquisition mass spectrometry can be used for high-dimensional characterization of immune cell sub-populations, even with limited cell numbers. These studies open the way for further understanding sub-populations contained within those defined by classical cell markers.

**Methods**

Primary human Cytotoxic CD8+ T cells, CD4+ T cells, CD14+ monocytes and natural killer (NK) cells, isolated from peripheral blood mononuclear cells, were prepared for mass spectrometry using standard sample preparation workflows. All samples were analyzed using 4 hour gradients on a C18 column coupled to a Thermo Scientific Q Exactive HF mass spectrometer in data-independent acquisition (DIA-MS) mode. DIA data was extracted using Spectronaut Pulsar X (Biognosys) with directDIA data searching.

**Results**

Cytotoxic CD8+ T cells were evaluated using 100,000 cells of input material, which resulted in >3500 proteins quantified in the primary cells. Serial dilutions revealed that >2750 proteins (~73% of all proteins detected) were quantified even with lower numbers of cells (30k). In the current experimental setup, 30k cells also represents the lower limit of detection of CD8A and CD8B. Among other previously characterized proteins associated with CD8+ T cells, KLRG1, CCL5, TBX21, GZMH, PRF1, GNLY, CST7 were all detected at 30k cell input, except KLRG1 which was detected at 50k cell input. Additionally, Granzyme A and B were also quantified which have classically been used, along with PRF1, as markers of lymphocyte infiltration. Data will be presented for the additional cell types (CD4+ T cells, CD14+ monocytes, and NK cells) to further map the immune cell phenotypic landscapes.

**Conclusions**

Our DIA-MS platform enables deep proteomic phenotyping of sorted immune cell samples, even with limited numbers of cells. These new data sets make available broad and un-constrained biomarker investigation for deconvolution of the processes driving immune cell activation and suppression.

#### P629 Addressing immunotherapy educational needs: esults from an educational program on immunotherapy for cancer patients and caregivers

##### Maria Gonzalo^1^, Liliana Zigo, BA^1^, Claire Saxton, MBA^1^, Heather Hollen, BS, MS^1^, Julie Olson, PhD^2^, Kevin Stein, PhD, FAPOS^2^

###### ^1^Cancer Support Community, Washington, DC, USA; ^2^Cancer Support Community, Philadelphia, PA, USA

####### **Correspondence:** Maria Gonzalo (mgonzalo@cancersupportcommunity.org)

**Background**

As cancer treatment becomes more personalized and the use of immunotherapy expands, it’s important that patients and caregivers have access to educational tools to make informed decisions, reduce cancer-related distress, and get optimal benefit across the continuum of care. This analysis explores participants’ experiences with the Cancer Support Community’s national evidence-based educational program, Frankly Speaking about Cancer: Immunotherapy. This comprehensive psychosocial program was created for people diagnosed with cancer and their families to provide information about immunotherapy as a treatment option, including how immunotherapy works, how to cope with the delayed response to treatment that is characteristic of some immunotherapy drugs, and the different side effects that people encounter while taking these treatments.

**Methods**

593 patients and 213 caregivers attending in-person Frankly Speaking about Cancer: Immunotherapy workshops across the country between 2014 and 2017 completed post-workshop evaluations. Survey questions focused on how cancer patients and their caregivers met their informational and assistance needs in regard to immunotherapy and whether participating in the workshop was associated with positive gains. Descriptive analyses and ANOVAs were used to assess workshop outcomes.

**Results**

73% of participants were cancer patients and post-treatment survivors; the remainder were caregivers who included spouses/partners (14%), family members (7%), and friends (6%). The average age of participants was 62 years old (s.d.= 11.4 years). While 39% of patients listed their primary diagnosis as ‘breast cancer,’ 31 other cancer diagnoses were represented in the survey. At the time of the workshops, 49% of patients had received the diagnosis within the last two years and 10% reported to have received immunotherapy treatment. Comparing retrospective pre-post workshop self-assessment means, participating in the workshops was associated with positive gains in knowledge about immunotherapy (F=265.8, p<.05). At the same time, 85% of patients and 67% of caregivers indicated that after the workshop they felt more confident to talk to their healthcare team about immunotherapy. The majority (78% of patients and 94% of caregivers) reported feeling better prepared to ask questions about side effects of immunotherapy. Finally, 95% of all participants said that they would recommend the program to their loved ones.

**Conclusions**

Results suggest that the program successfully improves self-rated knowledge about immunotherapy and boosts confidence in discussing this treatment modality and its side effects with healthcare providers. Furthermore, we found this program to be effective with both patients and caregivers, as over two-thirds in both groups indicated gaining confidence in talking to their providers.

#### P630 Patient-reported toxicities in lung cancer patients receiving immune checkpoint blockade

##### Heather Jim, PhD^1^, Sandra Shaw, BS, MBA^2^, Sarah Eisel, PhD^1^, Aasha Hoogland, PhD^1^, David LeDuc, CFRE^2^, Adam Dicker, MD, PhD^3^

###### ^1^Moffitt Cancer Center, Tampa, FL, USA; ^2^Addario Lung Cancer Foundation, Harleysville, PA, USA; ^3^Thomas Jefferson University, Philadelphia, PA, USA

####### **Correspondence:** Heather Jim (heather.jim@moffitt.org)

**Background**

Background: There is increasing clinical and research interest in patient-reported outcomes (PROs) which provide unique and complementary information to provider-rated adverse events. While several studies have been published of PROs in lung cancer patients receiving immune checkpoint blockade, to our knowledge all have focused on PROs collected as part of a clinical trial. Clinical trial participants tend to be younger and healthier than patients who receive standard of care. The goal of the current observational study is to describe PROs outside the context of a clinical trial in lung cancer patients treated with immune checkpoint inhibitors.

**Methods**

Method: The Addario Lung Cancer Foundation (ALCF) international patient registry was used to collect patient-reported clinical information. English-speaking patients who reported current or past treatment with an FDA- approved immune checkpoint inhibitor (i.e., atezolizumab, durvalumab, ipilumumab, nivolumab, pembrolizumab) were asked to complete a second survey of symptomatic toxicities of these therapies. Patients rated 40 symptoms (e.g., fatigue, rash) on a five-point scale (0=none, 4=very much). The Charlson Comorbidity Index and Functional Assessment of Cancer Therapy General (FACT-G) were administered to assess comorbidities and quality of life, respectively.

**Results**

Results: A total of 90 patients (mean age 62, 72% female, 87% White, 92% from the USA) who reported treatment with nivolumab (48%) or pembrolizumab (52%) were included in analyses. Patients reported 3 comorbidities on average (range 2-17). The majority of patients (53%) had been treated for 6 months or less. The most commonly reported symptoms were fatigue (89%), aching joints (72%), itching (65%), aching muscles (63%) skin dryness (62%), and insomnia (62%). A total of 25% of patients had experienced a treatment delay, 10% had been to the emergency room, and 8% had been hospitalized due to toxicity. Participants reported a mean score of 76.65 (SD=18.30) on the FACT-G, significantly lower than previously-published normative data for cancer patients [1].

**Conclusions**

Conclusions: This study is among the first to our knowledge to evaluate symptomatic toxicities of immune checkpoint inhibitors outside the context of a clinical trial. Results indicate that symptomatic toxicities of immune checkpoint inhibitors are common in lung cancer patients. Additional research is needed to better understand the longitudinal course of symptomatic toxicities and evaluate whether supportive care interventions to ameliorate symptoms are efficacious.

**Acknowledgements**

Addario Lung Cancer Foundation, Society for Immunotherapy in Cancer

**References**

1. Pearman T, Yanez B, Peipert J, Wortman K, Beaumont J, Cella D. Ambulatory cancer and US general population reference values and cutoff scores for the functional assessment of cancer therapy. Cancer. 2014; 120: 2902-2909.

**Ethics Approval**

This study was determined to be non-human subjects research because it uses anonymous data.

#### P631 A novel CD137/PD-L1 bispecific antibody modulates the tumour microenvironment by activating CD8^+^ T cells and results in tumour growth inhibition

##### Matthew Lakins, PhD, Jose Munoz-Olaya, Daniel Jones, Raffaella Giambalvo, Clinton Hall, Anne Knudsen, Neus Masque Soler, Dr, Sarka Pechouckova, Emma Goodman, BSc, Cristian Gradinaru, Alexander Koers, PhD, Sylwia Marshall, Mateusz Wydro, PhD, Francisca Wollerton, Sarah Batey, Dan Gliddon, Jacqueline Doody, PhD, Michael Davies, Michelle Morrow, PhD, Mihriban Tuna, PhD, Neil Brewis, PhD

###### F-star Biotechnology Ltd, Cambridge, UK

####### **Correspondence:** Matthew Lakins (matthew.lakins@f-star.com)

**Background**

Blockade of the PD-1/L1 axis has shown durable responses and extended overall survival in multiple cancer types. However, there is still a significant unmet need for patients who relapse and activation of the Tumour Necrosis Factor Receptor (TNFR) superfamily embodies the next stage of cancer immunotherapy. CD137-mediated costimulation directs the fate of antigen-stimulated T and NK cells. Upon interaction with its ligand, CD137 signalling supports cell activation, survival and proliferation. Current agonist therapeutic interventions aimed at CD137 hold great promise for cancer immunotherapy with the caveat of being associated with safety concerns. Here, we show how a novel bispecific antibody (mAb² ™) directed to CD137 and PD-L1 induces potent in vitro T cell activity in a PD-L1-dependent manner, and results in significant tumour control across three syngeneic tumour models without toxicity.

**Methods**

An anti-CD137/PD-L1 mAb² was generated by introducing a CD137-binding specificity into the Fc-region of a human IgG1 targeting PD-L1. FcγR binding was abrogated by introducing the LALA mutation. Binding characterisation was carried out via SPR and in vitro activity was assessed using functional assays employing engineered overexpressing T cell lines, and a mouse primary OT-1 CD8^+^ T cell assay. The anti-tumour activity of the anti-CD137/PD-L1 mAb² was tested in CT26, MC38 and B16-F10 tumour-bearing mice.

**Results**

An anti-CD137/PD-L1 bispecific antibody was developed, which binds mouse PD-L1 and which, upon binding to mouse CD137, elicits potent T cell stimulation in vitro (EC50: 3 pM in primary antigen-specific OT-1 assay). The mAb² significantly reduced tumour growth in MC38 and CT26 syngeneic colon carcinoma models, as well as in the B16-F10 syngeneic melanoma model. In CT26 this activity was dose-dependent resulting in a significant survival benefit at concentrations of 0.3 mg/kg or above and liver pharmacology was minimal as defined by histopathology.

**Conclusions**

We report potent in vitro CD137-mediated activation only upon engagement of PD-L1 using the anti-CD137/PD-L1 bispecific mAb² which outperforms monospecific antibodies on their own and in combination in multiple syngeneic mouse tumour models in a dose-dependent manner. None of the liver pharmacology and resultant toxicity reported with other CD137 agonist mAbs are observed with the anti-CD137/PD-L1 mAb². This warrants the development of a first-in-class anti-human CD137/PD-L1 bispecific antibody with a novel mode of action and improved therapeutic index for the treatment of human cancer.

**Ethics Approval**

The murine syngeneic tumour studies were approved by the Home Office, project license number 70/7991.

#### P632 A multi-omics approach to understanding the tumor microenvironment

##### Deepali Malhotra, PhD, Gordon Moody, Michael Surace, PhD, Jaime Rodriguez-Canales, MD, Ronald Herbst, PhD, John Mumm, PhD

###### MedImmune, Gaithersburg, MD, USA

####### **Correspondence:** Deepali Malhotra (malhotrad@medimmune.com)

**Background**

Human tumors contain highly variable immune and stromal cell infiltrates. Tumor heterogeneity, classically defined by tumor intrinsic variation in morphology and metabolism, is also observed at the level of infiltrating immune cell localization and activation within these tissues. Understanding the composition and functional consequences of different tumor microenvironments (TMEs) is critical to identifying correlates that inform our development and use of IO therapies in patients. Therefore, it is essential that tumor-targeted IO therapies be tested within the relevant context of the human TME. To this end, we developed a multi-omics work flow to characterize primary human tumors and to assess the functional consequences of intervention with immunotherapeutics that target different cell types and pathways within the TME.

**Methods**

Within 24 hours of surgical resection, tumors are enzymatically processed using a protocol optimized for cellular viability and maximal cellular yield. Additional tumor tissue is processed for FFPE to enable multi-parameter IHC analysis of the composition and structure of the TME. Flow cytometry panels were developed to define the immune, tumor, and stromal cell makeup of each disaggregated tumor and to provide insight into the nature and activation state of infiltrating lymphocytes and myeloid cells. Disaggregated tumors were treated in vitro with IO therapeutics that target different pathways and cell types, such as: co-stimulation, checkpoint blockade, and myeloid activation. Tumor disaggregates and supernatants are collected at various timepoints to assess the ability of these compounds to modulate transcriptional activity and cytokine outputs.

**Results**

Tumors representative of colon, lung, renal, and pancreatic cancers were assessed using the aforementioned workflow. The TMEs defined by each of these metrics highlighted the diversity of these samples, some samples were enriched for lymphocytes, other myeloid cells, while some had similar contributions of lymphocytes and myeloid cells. Infiltrating T cells also demonstrated various degrees of activation and exhaustion, likely affecting the ability of these disaggregates to respond to different classes of IO therapies.

**Conclusions**

We have developed a multi-omics workflow to allow evaluation of how the TME influences responses to different preclinical IO assets. This project represents an ongoing functional and phenotypic characterization effort to enable the development and implementation of novel tumor-targeted IO therapies. Preliminary results suggest that multi- omics approaches are required to understand the IO context within the TME. Further refinements of this methodology may include alternative approaches that better reproduce more complex aspects of the TME such as structure and localization of immune cells within the tumor.

**Ethics Approval**

All tumor samples were received in agreement with the IRB of the University of Maryland and processed in agreement with the ethical guidelines of MedImmune.

#### P633 Generation of a modular landing pad cell line for T cell receptor exchange and screening

##### Ethan Patterson, Stacey Ward, PhD, Jason Gustin

###### Milliporesigma, St. Louis, MO, USA

####### **Correspondence:** Ethan Patterson (ethan.patterson@sial.com)

**Background**

T cell biology is integral to the study of normal immune regulation as well as cancer biology, CAR-T cells, epitope specificity and antigen presentation. However, primary T cells can be difficult to propagate in culture for the length of time necessary for functional assays. In addition, populations of primary T cells express variant T cell receptor (TCR) heterodimers that can be challenging to identify and may not be optimal for downstream studies.

**Methods**

We sought to simplify this system using transformed T cells which can be grown in culture for extended periods of time. We engineered a floxed landing pad sequence into the safe harbor AAVS1 locus using CompoZr® zinc finger nucleases. Both the promoter and landing pad expression cassette are flanked by unique lox sites, allowing swapping of the promoter and/or expression cassette as needed. We ensured that only one copy of this sequence was found within the genome to avoid any complications associated with random insertion events.

**Results**

We also generated a landing pad cell line null for the endogenous TCR using targeted nucleases. Both the TCR alpha and beta loci were rendered null due to non-homologous end joining and the presence of insertions and deletions culminating in premature stop codons were genotyped using next generation sequencing. The absence of a functional TCR was validated using flow cytometry staining for surface TCR and CD3. This cell line was then used to generate a knock-in of the desired exogenous TCR heterodimer to the landing pad locus, verified using flow cytometry staining.

**Conclusions**

These lines will be very useful for a multitude of studies where a researcher needs to express a gene of interest in a discrete genetic locus or wants to generate a panel of TCR expressing cell lines.

#### P634 Precision genome editing in macrophage and CD8+ human primary T cells for immuno-therapeutics applications

##### Laura Daley, Ethan Patterson

###### Milliporesigma, St. Louis, MO, USA

####### **Correspondence:** Ethan Patterson (ethan.patterson@sial.com)

**Background**

Innate immune cells play a critical role in cell-mediated immunity and have the potential to serve as cell-based therapies to treat a broad spectrum of immune diseases such as cancer and autoimmune disorders. Modified immune cells, such as genetically engineered CAR-T cells, have proven to be critical in developing new cell-based therapies for these diseases. However, immune cell biology creates challenges during the gene-editing process that lead to hyper-regulated RNA and DNA sensing pathways and enhanced cell death upon introduction of exogenous ribonucleotides. Further, engineering in primary immune cells is often restricted due to their limited expansion capacity.

**Methods**

Genetic engineering in immune cells has traditionally relied on random integration of gene-editing components using viral delivery systems. In contrast, genome editing mediated by nucleases, such as CRISPR/Cas9-single guide RNA ribonucleoproteins (RNPs), provide a platform for precision editing, and alleviate the potential side effects caused by randomly integrated viral DNA. While RNP gene editing in immune cells is just beginning to be considered by the immune-therapeutics field, our recent advances demonstrate that this approach can be used to create targeted modifications in two key cell types, the macrophage and the CD8+ primary T-cell.

**Results**

In an effort to circumvent challenges with the finite lifespan of primary T-cells, we targeted genes to edit that rendered this cell type “pseudo-immortalized”, thus allowing additional passages for further downstream genome editing and propagation. In addition, we demonstrated that precision editing can be used to introduce disease relevant single-nucleotide-polymorphisms (SNPs) into the macrophage genome, which resist introduction of exogenous ribonucleotides due to the induction of apoptotic pathways.

**Conclusions**

Advances such as these overcome many of the obstacles currently faced with immune cell editing and offer improved gene stability and expression in immune cells and, in doing so, will transform the Immuno-Oncology and Gene Therapy fields.

#### P635 Effectiveness and tolerance of immune checkpoint blockade in a real-world lung cancer patient population

##### Daniel Pease, MD^1^, Michael Shyne^2^, Shilvi Joshi^2^, Allison Lee^2^, Manish Patel, DO^2^

###### ^1^Hennepin Healthcare, Minneapolis, MN, USA; ^2^University of Minnesota, Minneapolis, MN, USA

####### **Correspondence:** Daniel Pease (peas0044@umn.edu)

**Background**

The PD-1 inhibitors nivolumab and pembrolizumab are approved for treatment of non-small cell lung cancer in the second line setting, based on superior outcomes compared to chemotherapy. The study populations generally were composed of younger patients with excellent performance status (ECOG 0-1) and a minimal number of prior lines of therapy. In our study we evaluated outcomes with these agents in older, less fit, and more heavily pretreated lung cancer patients that are reflective of real-word practice.

**Methods**

A single-institution retrospective analysis of all lung cancer patients treated with nivolumab or pembrolizumab at the University of Minnesota from 2015 to 2016. Outcomes were compared by age, performance status (PS), prior lines of therapy, presence of brain metastases, history of autoimmune disease, and immune-related adverse events (irAE). Overall survival (OS) data was calculated using two-sample log-rank testing.

**Results**

111 patients received at least two doses of nivolumab or pembrolizumab, with median age 65 years. Overall the complete response rate was 2.7%, partial response 27.9%, and stable disease 19.8%, for a clinical benefit rate of 50.4%. The median duration of response was 12.5 months and median OS 11 months. The clinical benefit rate was not significantly different by age (52.8% for ≤65 versus 59.6% for >65 and 55.4% for ≤75 versus 58.8% for >75), PS (55.1% for ECOG 0-1 versus 62.5% for >1), or prior lines of therapy (53.6% for 0-1 versus 59.1% for >1). Median OS was 5 months for patients with ECOG PS >1 compared to 13 months for PS 0-1 (p-value 0.00042). Survival by age and prior lines of therapy was not significantly different. The incidence of irAE requiring steroids was 19.8%, with no treatment-related deaths. The median OS for patients experiencing an irAE was 23.9 months versus 9 months for patients without an irAE. Of 9 patients with a history of autoimmune disease, only 1 experienced disease flair. In patients with no history of brain metastases, only 5 (6.3%) developed CNS progression. Of those with previously treated brain metastases, 29.6% had CNS progression.

**Conclusions**

The clinical benefit of immune checkpoint blockade persists in older or heavily pretreated patients. Survival for patients with PS >1 is very limited, suggesting these agents should be used judiciously in this group. Therapy was well tolerated, with a low risk for flare of previous autoimmune disease, and appears to be effective for CNS disease. Incident irAE predicted for improved OS.

**Ethics Approval**

This study was approved by the University of Minnesota’s institutional review board; approval number 1606M88925.

#### P636 Evaluating the occurrence of early tumor progression (ETP) in patients with gastric cancer treated with nivolumab versus placebo

##### Yan Feng^1^, Paul Nghiem, MD, PhD^2^, Ricardo Zwirtes, MD^1^, Dan Reshef^1^, Greg Plautz^1^, Narikazu Boku^3^, Li-Tzong Chen^4^, Yoon-Koo Kang^5^, Akintunde Bello, PhD^1^, Amit Roy^1^, Jennifer Sheng^1^

###### ^1^Bristol-Myers Squibb, Princeton, NJ, USA; ^2^University of Washington, Seattle, WA, USA; ^3^National Cancer Center Hospital, Tokyo, Japan; ^4^National Institute of Cancer Research, Tainan, Taiwan, Province of China; ^5^Asan Medical Center, Seoul, Korea, Republic of

####### **Correspondence:** Jennifer Sheng (Jennifer.Sheng@bms.com)

**Background**

Early tumor growth has been documented as a feature of natural disease progression in some patients [1,2]. However, without consideration of the natural history of disease progression, there have been recent reports of “hyperprogression” (using various definitions of the term) to suggest accelerated tumor growth in some patients receiving anti–programmed death 1/programmed death ligand 1 therapies [3-5]. Additionally, these reports do not account for the necessity for randomization and suitable control groups [5]. The reported phenomenon of “hyperprogression” was assessed with data from a randomized controlled phase 3 trial of nivolumab versus placebo (as a surrogate for the natural history of disease progression) in patients with unresectable, advanced, or recurrent gastric cancer who had received at least 2 prior lines of treatment (NCT02267343, ATTRACTION-2). Tumor growth of all patients was retrospectively evaluated at the first on-treatment scan, relative to baseline, using a tumor growth dynamics (TGD) model, with a focus on patients experiencing ETP at the first tumor assessment.

**Methods**

Patients from Japan, Korea, and Taiwan with unresectable, advanced, or recurrent gastric cancer, refractory or intolerant to standard therapy, were randomized 2:1 to nivolumab or placebo (N = 493). A TGD model was developed to characterize change of tumor size using longitudinal data from 358 patients who had baseline and at least 1 post treatment tumor measurement available. An increase of ≥20% in the sum of longest diameter (SLD) of target lesions at 8 weeks post baseline was considered ETP.

**Results**

A high variability of change in tumor size was observed among both placebo- and nivolumab-treated patients, consistent with the variable nature of cancer progression and response. The percentage of ETP was lower in patients receiving nivolumab compared with placebo, across cutoffs up to 100% increase in SLD relative to baseline (Table 1). Pseudoprogression was reported in 2 patients receiving nivolumab (~1%) and in none of the patients receiving placebo. Furthermore, the maximum observed SLD increases in nivolumab and placebo arms were 260% and 130%, respectively. A small fraction of patients in both nivolumab and placebo arms (~1%) experienced ETP >100% of observed SLD at the first assessment.

**Conclusions**

This study does not support any association between nivolumab therapy and early tumor progression. However, this study focused on patients with gastric cancer; the potential of hyperprogression following immuno-oncology therapy should be investigated in other randomized trials with a placebo arm, focusing on different tumor types.

**Acknowledgements**

Medical writing assistance was provided by Katerina Pipili, PhD, of Spark Medica Inc. (US), funded by Bristol-Myers Squibb. This study was funded by Bristol-Myers Squibb. Mitsunobo Tanimoto contributed as the NCT02267343 Study Director.

**Trial Registration**

ClinicalTrials.gov: NCT02267343

**References**

1. Detterbeck FC, Gibson CJ. Turning gray: the natural history of lung cancer over time. J Thorac Oncol. 2008;3:781-792.
2. Wang J, Mahasittiwat P, Wong KK, Quint LE, Kong FM. Natural growth and disease progression of non-small cell lung cancer evaluated with 18F-fluorodeoxyglucose PET/CT. Lung Cancer. 2012;78:51-56.
3. Champiat S, Dercle L, Ammari S, Massard C, Hollebecque A, Postel-Vinay S, Chaput N, Eggermont A, Marabelle A, Soria JC, Ferté C. Hyperprogressive disease is a new pattern of progression in cancer patients treated by anti-PD-1/PD-L1. Clin Cancer Res. 2017;23:1920 1928.
4. Kato S, Goodman A, Walavalkar V, Barkauskas DA, Sharabi A, Kurzrock R. Hyperprogressors after immunotherapy: analysis of genomic alterations associated with accelerated growth rate. Clin Cancer Res. 2017;23:4242-4250.
5. Sharon E. Can an immune checkpoint inhibitor (sometimes) make things worse? Clin Cancer Res. 2017;23:1879- 1881.

**Ethics Approval**

The protocol and all amendments were approved by the institutional review board or independent ethics committee for each study center.

**Table 1 (abstract P636).**
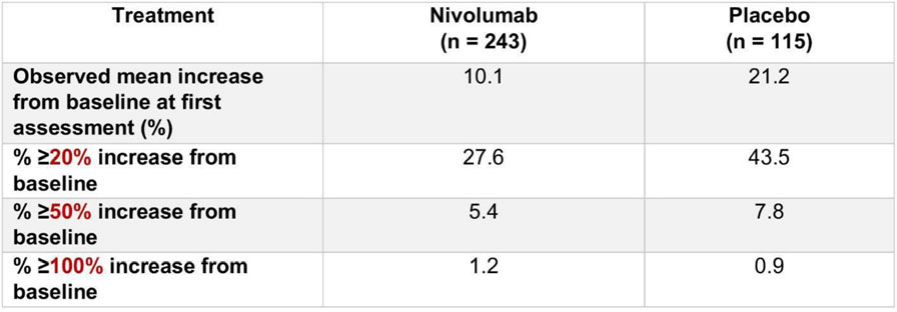
See text for description

#### P637 Fibroblast activation protein (FAP)-selective delivery of CD40 agonistic DARPin® molecule for tumor- restricted immune activation

##### Nicolo Rigamonti, PhD, Anja Schlegel, Sophie Barsin, Master of Science, Jonas Schwestermann, Jennifer Krieg, Susanne Mangold, Maria Paladino, Valérie Calabro, PhD, Victor Levitsky, MD PhD, Simon Plyte, PhD, Michael Stumpp, PhD, Clara Metz, PhD

###### Molecular Partners AG, Schlieren - Zurich, Switzerland

####### **Correspondence:** Nicolo Rigamonti (nicolo.rigamonti@molecularpartners.com)

**Background**

CD40 is a co-stimulatory molecule belonging to the tumor necrosis factor receptor superfamily which can activate both innate and adaptive immune system, making it an interesting target for tumor immunotherapy. Systemic activation of CD40 receptor, induced by administration of agonistic CD40 antibodies, has shown signs of activity in cancer patients, but dose-limiting toxicities have impaired the efficacy. New therapeutic approaches are therefore needed to increase the therapeutic index of CD40-targeting molecules and achieve better clinical outcomes. Here, we report a novel approach based on systemic administration of a tumor-restricted CD40 agonistic DARPin® molecule targeting human CD40 and fibroblast activation protein (FAP) alpha, a tumor antigen (TA) abundantly expressed in many solid tumors, enabling CD40 pathway activation exclusively in the presence of TA-expressing cells.

**Methods**

Using ribosome display technology, we generated a panel of bispecific CD40/FAP DARPin® molecules able to trigger specifically CD40 receptor and activate the NF-κB pathway in a reporter cell assay, in the presence of FAP- transfected Chinese hamster ovary (CHO) cells, but not in the presence of parental CHO cells. Selected bispecific DARPin® clones were then tested in more meaningful cell assays using human 1) primary B cells, 2) monocyte- derived dendritic cells and 3) monocyte-derived macrophages from whole blood, in the presence of FAP-positive or FAP-negative cells.

**Results**

In these immune cell populations, bispecific CD40/FAP DARPin® molecules confirmed a FAP-dependent activation of CD40 pathway inducing an upregulation of costimulatory molecules and proinflammatory cytokines, such as CD86 and IL12, only in the presence of FAP-expressing cells. In order to properly address the in vivo activity, a surrogate mouse-specific CD40/FAP DARPin® molecule was also generated and tested in different in vitro assays showing a FAP-dependent activation and similar results as the human counterpart. Experiments are ongoing to assess the efficacy and mechanism of action of this tumor-restricted CD40 agonistic DARPin® molecule.

**Conclusions**

In conclusion, we have generated bispecific agonist CD40/FAP DARPin® molecules able to activate the CD40 pathway in cellular assays in a targeting-dependent manner, supporting the hypothesis that these DARPin® molecules could lead to a tumor-localized immune activation in vivo. Data of the ongoing in vivo experiments in mouse tumor models to test this hypothesis will be shown at the meeting.

**Ethics Approval**

In vivo studies were approved by Veterinary Authorities of the Canton Zurich, approval number ZH102/16.

#### P638 Comparison of immunoprofiling from whole tumor versus pathologist-selected regions of interest in tissues: a study using multiplex immunofluorescence and multispectral analysis on lung cancer

##### Michael Surace, PhD^1^, Matthew Gates^1^, Clifford Hoyt, MS^2^, Jennifer Cann, PhD^1^, Jaime Rodriguez-Canales, MD^1^

###### ^1^MedImmune, Gaithersburg, MD, USA; ^2^PerkinElmer, Hopkinton, MA, USA

####### **Correspondence:** Jaime Rodriguez-Canales (rodriguezcanalesj@MedImmune.com)

**Background**

Multiplex immunofluorescence (mIF) and multispectral analysis has become an important tool for cancer immunoprofiling. An investigator, usually pathologist, selects regions of interest (ROI) within the tumor area for multispectral scanning and immunophenotyping analysis. However, tumor heterogeneity and human sample bias may affect the representation of the immunoprofiling in the whole tumor area. Our goal was to compare the tumor immunoprofiling data using ROI analysis versus whole tumor region scanned in multispectral mode.

**Methods**

20 non-small cell lung carcinomas (9 adenocarcinomas (ADC), 11 squamous cell carcinomas (SCC) were stained with a 6-marker mIF panel (PD-L1, CD8, Ki-67, CD68, pancytokeratins, and PD-1) and imaged using a multispectral scanner. Two pathologists (A and B) independently selected 5 ROI (0.64 mm2 each) within the tumor area for each case. Additionally, to assess the human sampling bias, two sets of 5 ROI where generated randomly within the tumor by a computer (C and D). The immunoprofiling data was generated using HALO software from all ROI sets (A-D) and independently compared to the whole tumor region.

**Results**

Cell densities and percentages of 8 cell subpopulations [CD8+; CD68+; PD-1+; (PD-1+/CD8+); (CK+/Ki67+); (CK+/PDL1+); (CD68+/PD-L1+), and (CD8+/Ki67+)] were evaluated in tumor epithelium and stroma compartments. These data were assessed in each set of 5 ROI (A-D) and compared with the data from the whole tumor. Pearson’s correlation coefficients (PCC) between the whole tumor and ROI data ranged from 0.65 to 0.99. The endpoints for which ROI were least representative of whole tumor were percent of CD8+/Ki67+ cells in the stroma (PCC 0.65), and PD-1+/CD8+ cell density in the epithelial compartment (PCC 0.70). The endpoints for which ROI were most representative of whole tumor were PD-1+ cell density in tumor stroma (0.98) and percent CD68+ macrophages in stroma (0.99). The average PCC for randomly-assigned ROI (computer) was 0.96 vs 0.90 for pathologist-assigned ROI. ADC were more robustly assessed by ROI (PCC 0.97) as compared to SCC (0.88). As compared to the whole tumor, and across all reportable metrics, pathologist-placed ROI undercounted by an average of 18.36% and computer-placed ROI undercounted by 7.02%.

**Conclusions**

Overall, our results suggests that immunoprofiling using 5 ROI is comparable to the data from whole tumor region. However, the assessment of cell densities in ROI for certain populations showed a lower correlation, suggesting an advantage of the analysis of either more than five ROI or the whole tumor region for the evaluation of cell densities.

**Ethics Approval**

Tissues were commercially available and obtained according the Declaration of Helsinki

#### P639 Tumor-associated B cells promote melanoma cell dedifferentiation and invasiveness

##### Anastasia Samarkina, MSc, Rajasekharan Somasundaram, PhD, Meenhard Herlyn, DVM PhD

###### The Wistar Institute, Philadelphia, PA, USA

####### **Correspondence:** Meenhard Herlyn (herlynm@wistar.org)

**Background**

Tumor heterogeneity and dedifferentiation of cancer cells have been shown to hamper the effective response to the targeted- and immune-therapies. This ultimately leaves two-thirds of metastatic melanoma patients with a limited number of treatment options. The role of the tumor microenvironment and specifically tumor-associated stromal cells on the induction of dedifferentiation and on the presence of heterogenous tumor subpopulations is not fully understood.

**Methods**

In the present work, we have dissected the functional interplay between tumor-associated B cells (TABs) and melanoma cells through the integration of a humanized mouse model, computational transcriptome analysis, and organotypic 3D culture assays.

**Results**

We first found that immunotherapy-resistant tumors from humanized mice showed a dramatic influx of CD19+ B cells and exhibited the focal loss of melanoma-specific antigens intratumorally. Of note, both of these trends are not seen in immunotherapy-responding tumors. We then performed a gene microarray and detected that in a stark contrast to the normal B cells (NBs) derived from healthy donors, TABs’ transcriptome had enriched signature of inflammatory and angiogenic factors, as well as numerous molecules involved in invasion and matrix degradation. In concordance with our mouse studies, we also found that TABs induced a robust upregulation of migration- associated, neural crest and early neural progenitor markers across different melanoma cell lines as determined by microarray and RT-qPCR. The enhanced invasive phenotype was further functionally validated in a 3D organotypic culture, where melanoma spheroids cultured in the presence of TABs showed an average two-fold increase in invasive potential when compared to spheroids co-cultured with NBs.

**Conclusions**

Ultimately, our results reveal a novel role of TABs in mediating the invasiveness and dedifferentiation of human melanoma cells.

**Ethics Approval**

All mouse studies were performed at the Wistar Institute (AAALAC accredited) and approved by the Institutional Animal Care and Use Committee.

#### P640 hMENA is a key regulator of tumor cell-cancer associated fibroblasts dialogue via Gas6/Axl paracrine axis

##### sheila spada, PhD^1^, Roberta Melchionna^2^, Francesca Di Modugno, PhD^2^, Mariangela Panetta^2^, Anna Di Carlo^2^, Anna Maria Mileo^2^, Isabella Sperduti^2^, Barbara Antoniani^2^, Rita Teresa Lawlor^3^, Lorenzo Piemonti^4^, Maria Grazia Diodoro^2^, Daniel D'Andrea, PhD^5^, Paolo Visca^2^, Michele Milella^2^, Gian Luca Grazi^2^, Francesco Facciolo^2^, Emily Chen^6^, Aldo Scarpa^3^, Paola Nistico’, MD^7^

###### ^1^Weill Cornell Medicine, new york, NY, USA; ^2^IRCCS - Regina Elena National Cancer Institute, Rome, Italy; ^3^ARC-NET Research Centre, Univ. of Verona, Verona, Italy; ^4^IRCCS - San Raffaele Scientific Inst., Verona, Italy; ^5^Centre for Cell Signaling and Inflammation, Imperial College, London, UK; ^6^Herbert Irving Comprehensive Cancer Center, Columbia University Medical Centre, New York, USA; ^7^Regina Elena National Cancer Institute, Rome, Italy

####### **Correspondence:** Paola Nistico’ (paola.nistico@ifo.gov.it)

**Background**

Cancer-associated fibroblasts (CAFs) play a critical role in the complex process of tumor-stroma interaction, and may either favor or hinder tumor initiation, progression and drug resistance.The identification of phenotypical and functional subtypes is of great relevance for the development of microenvironment-related anti-tumor treatment.Pancreatic ductal adenocarcinoma (PDAC) and non-small cell lung cancer (NSCLC) are tumours with an abundant fibrotic stroma, and we have demonstrated that the tissue-specific alternative splicing of the actin regulator hMENA, generates hMENA11a and hMENAΔv6 isoforms that represent powerful diagnostic and prognostic factors in early stage NSCLC and PDAC [1-3]. hMENA/hMENAΔv6 influence intracellular signaling pathways involved in invasion and epithelial mesenchymal transition (EMT)[4-6] and [3].Here we aimed at investigating the role of hMENA isoforms in CAF functions and cancer cell-CAF crosstalk.

**Methods**

We have analyzed the expression of hMENA isoforms in normal fibroblasts and in CAFs isolated from pancreatic and lung cancer tissues, by IF and WB. The role of hMENA isoforms in CAF activity were analyzed by loss and gain of function experiments. hMENAΔv6-driven secreted factors were identified by LC-MS/MS proteomic analysis.

**Results**

CAFs express hMENA and hMENAΔv6 isoforms but not hMENA11a. hMENAΔv6 is overexpressed in CAFs compared to normal pancreatic fibroblasts and lung fibroblasts isolated from tissue distant from the tumor. The downregulation of hMENA/hMENAΔv6 isoforms, by siRNA, reduced the contractile activity of CAFs and MMP2 activity. Conversely, hMENAΔv6 overexpression in CAFs promoted their ability to invade, to activate the MMP2 and increase CAF-mediated cancer cell invasiveness. Notably, tumor cells over-expressing hMENA/hMENAΔv6 secrete factors essential for hMENAΔv6 overexpression and CAF activation, indicating hMena as crucial in tumor/CAF co-evolution. LC-MS/MS analysis revealed that CAFs over-expressing hMENAΔv6 secret the Axl ligand Gas6, favoring the invasiveness of Axl-expressing NSCLC and PDAC cells. Of relevance we demonstrated that hMENA regulate Axl expression in tumor cells and sustain the paracrine Gas6-Axl axis. Clinically, we found that a high hMENA/Gas6/Axl gene expression signature is associated with a poor prognosis in PDAC patients.

**Conclusions**

We demonstrated that hMENA/hMENAΔv6 identify a subset of CAFs with pro-tumoral functions and defined a novel function of hMENA in regulating tumour/stroma cross-talk via paracrine Gas6-Axl signaling, described as crucial in EMT, drug resistance and immune evasion [7]. We indicate that the network-based on hMENA/Gas6/Axl expression may represent novel prognostic and therapeutic targets and the pattern of hMENA isoform expression in both tumor cells and CAFs may reveal tumor mesenchymal traits identifying cancer subtypes for tailored therapies.

**Acknowledgements**

* S.S. and R.M. contributed equallyThe project is supported by the Italian Association for Cancer Research AIRC: 5×1000, 12182, and IG 15224.

**References**

1. Bria E, Di Modugno F, Sperduti I, Iapicca P, Visca P, Alessandrini G, Antoniani B, Pilotto S, Ludovini V, Vannucci J, Bellezza G, Sidoni A, Tortora G, Radisky DC, Crinò L, Cognetti F, Facciolo F, Mottolese M, Milella M, Nisticò P. Prognostic impact of alternative splicing-derived hMENA isoforms in resected, node-negative, non- small-cell lung cancer. Oncotarget. 2014; 30;5(22):11054-63.
2. Di Modugno F, Spada S, Palermo B, Visca P, Iapicca P, Di Carlo A, Antoniani B, Sperduti I, Di Benedetto A, Terrenato I, Mottolese M, Gandolfi F, Facciolo F, Chen EI, Schwartz MA, Santoni A, Bissell MJ, Nisticò P. hMENA isoforms impact NSCLC patient outcome through fibronectin/β1 integrin axis. Oncogene. 2018 Jun 15.
3. Melchionna R, Iapicca P, Di Modugno F, Trono P, Sperduti I, Fassan M, Cataldo I, Rusev BC, Lawlor RT, Diodoro MG, Milella M, Grazi GL, Bissell MJ, Scarpa A, Nisticò P. The pattern of hMENA isoforms is regulated by TGF-β1 in pancreatic cancer and may predict patient outcome. Oncoimmunology. 2016 ; 12;5(12).
4. Di Modugno F, Iapicca P, Boudreau A, Mottolese M, Terrenato I, Perracchio L, Carstens RP, Santoni A, Bissell MJ, Nisticò P. Splicing program of human MENA produces a previously undescribed isoform associated with invasive, mesenchymal-like breast tumors. Proc Natl Acad Sci U S A. 2012 Nov 20; 19280-5.
5. Di Modugno F, Caprara V, Chellini L, Tocci P, Spadaro F, Ferrandina G, Sacconi A, Blandino G, Nisticò P, Bagnato A, Rosanò L. hMENA is a key regulator in endothelin-1/β-arrestin1-induced invadopodial function and metastatic process. Proc Natl Acad Sci U S A. 2018 20; 3132-3137.
6. Sistigu A, Di Modugno F, Manic G, Nisticò P. Deciphering the loop of epithelial-mesenchymal transition, inflammatory cytokines and cancer immunoediting. Cytokine Growth Factor Rev. 2017 Aug;36:67-77.
7. Aguilera TA, Giaccia AJ. Molecular pathways: oncologic pathways and their role in T-cell exclusion and immune evasion-a new role for the AXL receptor tyrosine kinase. Clin Cancer Res. 2017;15;23(12):2928-2933.

**Ethics Approval**

The Study was approved by Regina Elena National Cancer Institute's Ethics Board, approval number 1008/17

#### P641 Development of automated protocols for OPAL, SMIFT and ESIFT 4-color lymphocyte kits

##### Joe Vargas, Julio Masabanda, PhD, David Tacha, PhD

###### Biocare Medical, Martinez, CA, USA

####### **Correspondence:** David Tacha (dtacha@biocare.net); Joe Vargas

**Background**

We have compared the staining of the OPAL 4-color lymphocyte kit for manual use with the staining of the same kit adapted for the ONCORE automatic stainer. This kit is composed of primary antibodies against CD4, CD8 and CD20, and they are detected using a TSA-based fluorescence reaction. Additionally, we had developed in-house two additional kits with the same components as the OPAL 4 color kit, e.g. containing CD4, CD8 and CD20 antibodies. These kits we designated as SMIFT- (Sequential Multiplex Immunofluorescence Technology) and ESIFT- (Enhanced Sequential Immunofluorescence Technology) - 4-color lymphocyte kits, respectively. The goal was to develop an effective fluorescence-based assay that can be fully automated and used to assess the numbers of immune cells within the tumor microenvironment.

**Methods**

SMIFT is based on the indirect detection of antibody binding to hapten using secondary antibodies labeled with fluorescence. ESIFT is based on the use of primary antibodies directly labeled with HRP and detection with fluorescence. Additionally, we had compared the staining of the manual protocols for OPAL with SMIFT 4-color lymphocyte kits.

**Results**

Microscope observations show that the staining intensities are comparable for both OPAL and ESIFT on the ONCORE. The intensity of the staining for SMIFT 4-color kit is the lowest of the compared systems. This is expected because SMIFT is not a TSA-based detection technology. Noteworthy, the fluorescence from the 3 antibodies of all kits systems is clearly visible directly under the fluorescence microscope and this is also valid for the SMIFT system.On the tissues in general the fluorescence from all the kits components was brighter for the Melanoma tissue compared to the tonsil control. For running the OPAL kit on the ONCORE, the manufacturer suggested protocol had to be adjusted in order to get a satisfactory staining on the tested tissues. The SMIFT protocol compared to OPAL required less optimization during the conversion of the manual to the ONCORE version. The ESIFT kit required the least efforts for protocol optimization for the ONCORE. Protocol run time on the ONCORE was the shortest for ESIFT (about 5.5 hours) and for SMIFT and OPAL the run times on the ONCORE were comparable (about 7 hours).

**Conclusions**

The manual performance is more labor intensive for the OPAL compared to the SMIFT kit. Obviously, the availability of automatic stainers has enabled the consistent performance without major human intervention of involved technologies such as OPAL 4-color kit.

#### P642 Withdrawn

### Patient Experience

#### P643 Oncology Nursing Society’s patient immunotherapy wallet cards improve communication and immune- related adverse event management

##### Kathleen Wiley, RN, MSN, AOCNS, Michele Galioto, RN, MSN, Kathleen Wiley, RN, MSN, AOCNS, Nicole Lininger, Lisa Sheldon, PhD, ANP-BC, PhD APRN

###### Oncology Nursing Society, Woolwich, NJ, USA

####### **Correspondence:** Michele Galioto (mgalioto@ons.org)

**Background**

As the number of patients receiving immunotherapy agents continue to grow, so too does the potential for non-oncology clinicians to need to manage immune related toxicities. The similarities in the side effect profile of chemotherapy and immunotherapy agents are striking, yet their mechanism of action and evidence-based management strategies differ greatly. Oncology Nursing Society (ONS) members recognized the need to facilitate communication between oncology and non-oncology providers to improve outcomes of patients receiving immunotherapy.

**Methods**

ONS developed immunotherapy wallet cards to improve communication between oncology providers and non-oncology healthcare teams. The card enables patients to carry information about their treatment and side effects with them to appointments with non-oncology providers. The goal is to help inform providers who may not be involved with a patient’s cancer treatment that he/she is receiving immunotherapy, and this will greatly impact their care. On the card, the oncology provider indicates whether the patient is receiving checkpoint inhibitors, monoclonal antibodies, adoptive cell therapy, vaccines or oncolytic viral therapy. Wallet cards include information about expected side effects associated with these agents and cautions them to be aware that these side effects, while they may mimic those of chemotherapy, require vastly different management. The cards include a prompt to contact the oncology care team before altering immunotherapy treatment regimens.

**Results**

Immunotherapy wallet cards were finalized and marketed to the public starting in January 2018. Since that time, 90,000 wallet cards have been ordered by oncology providers, nurses, pharmacists and patients and families, both within the USA and internationally. Those who have ordered cards from ONS have been contacted for feedback on utilization and outcomes observed as a result of using wallet cards.

**Conclusions**

Immunotherapy as a form of cancer treatment is growing at an exponential rate. This increase in treatment options is met with many questions about adverse event management, and the speed at which approvals are seen mean clinicians outside the immediate oncology arena may not be fully aware of the intricacies of immunotherapy that set it apart from other antineoplastic strategies. The ONS Immunotherapy Wallet Card offers a strategy to help mitigate the unanticipated effects of such rapid growth of this class of treatment, and ensure patients have a way to communicate their treatment status and side effects likely to affect this patient population.

### T-cell Checkpoints and Checkpoint Inhibitors

#### P644 Checkpoint inhibitor therapy in solid organ and allogeneic stem cell transplantation: data mining of The Truven Health Marketscan Research Database

##### Noha Abdel-Wahab, MD, PhD^1^, Ala Abudayyeh^1^, Xiudong Lei^1^, Gheath Alatrash, DO, PhD^1^, Hui Zhao^1^, Sharon Giordano^1^, Houssein Safa, MD^1^, Daniel Johnson, MD^1^, Van Trinh, DPharm^1^, Maen Abdelrahim, MD, PhD^2^, Ahmed Gaber, MD^2^, Maria Suarez-Almazor, MD, PhD^1^, Adi Diab, MD^1^

###### ^1^UT MD Anderson Cancer Center, Houston, TX, USA; ^2^Houston Methodist, Houston, TX, USA

####### **Correspondence:** Adi Diab (adiab@mdanderson.org)

**Background**

There are paucity of data on the safety of checkpoint-inhibitors (CPI) in patients (pts) with solid-organ transplant (SOT) and allogeneic stem-cell transplant (ASCT), primarily from case reports and small series. As the use of CPI expands to many new indications, stratifying the risk-benefit ratio in this population is needed. To estimate the magnitude of this oncological challenge, we used a health insurance-based database to identify the rate of rejection among transplant recipients treated with CPI.

**Methods**

We identified cancer pts with SOT/ASCT receiving CPI from the Truven Health MarketScan Research Database between 2011 and 2016. Only pts who had CPI claims post-transplant were considered. Validated ICD9&10 diagnostic codes were used to identify definite graft rejection or graft versus host disease (GVHD) occurred at any time after the first CPI.

**Results**

A total of 50 pts were identified. The SOT cohort included 22 pts; median age was 53 (12-70) years; 73% were male; 40.9% had melanoma. Most received PD1 inhibitors (59.1%). Kidney transplant was reported in 77.3%. Other SOT included liver, lung, heart, and pancreas. Median time from SOT till CPI initiation was 3.7 (0.2-11.6) years. Median duration of follow-up since CPI initiation was 3.2 (0.1-17.8) months. Four pts (36.4%) had graft rejection after a median of 0.7 (0-3) months post CPI initiation (3 kidney, and 1 heart); 3 after receiving ipilimumab. Seven pts (63.6%) with at least four months follow-up had no rejection. Additionally, 11 pts had shorter follow-up (0.4-3.8 months) and no rejection at last claim. The ASCT cohort included 28 pts; median age was 46 (14-64) years; 53.6% were male; 46.4% had lymphoma. Most received PD1 inhibitors (75.0%). Median time from ASCT till CPI initiation was 3.6 (1.1 -8.6) years. Median duration of follow-up since CPI initiation was 6.5 (0.1-24.3) months. Nine pts (52.9%) had GVHD after a median of 1.7 (0-6.6) months post CPI initiation; 8 after receiving PD1 inhibitors. Eight pts (47.1%) with at least six months follow-up had no GVHD. Additionally, 11 pts had shorter follow-up (0.1-5.2 months) and no GVHD at last claim.

**Conclusions**

Acute rejection and GVHD were reported as a claim in over a third and over a half of SOT and ASCT recipients, respectively, after CPI initiation. Organ rejection tend to occur earlier than GVHD. Pre-clinical studies, and multi- institutional prospective studies in transplant recipients are needed to evaluate potential risk factors and management strategies that maintain graft tolerance without compromising antitumor benefits.

#### P645 Clinical characteristics and outcomes of immune checkpoint inhibitor-induced pancreatic injury

##### Hamzah Abu-Sbeih, MD, Tenglong Tang, MD, Yinghong Wang, MD, PhD

###### MD Anderson Cancer Center, Houston, TX, USA

####### **Correspondence:** Yinghong Wang (ywang59@mdanderson.org)

**Background**

Lipase elevation is one of the reported immune checkpoint inhibitor (ICI)-induced adverse events.[1-3] The incidence and clinical characteristics of immune-mediated pancreatitis with clinical symptoms and suggestive imaging findings are not well studied in the literature. Therefore, we aimed to describe the clinical characteristics and outcomes of patients who developed immune mediated pancreatic injury (IMPI).

**Methods**

We studied consecutive patients that received ICI and developed IMPI from 1/2010 through 3/2018. IMPI was defined as CTCAE grade ≥3 lipase elevation. Comprehensive chart review was then conducted to confirm the diagnosis of IMPI, based on the clinical judgement of the treating oncologist or gastroenterologist, and to exclude other etiologies. Chi-square test, Fisher exact test, and Wilcoxon rank-sum test were used to compare clinical characteristics. Kaplan-Meier curves and log-rank test were used to estimate and compare unadjusted survival time distributions.

**Results**

Eighty-two (3.5%) out of the 2279 patients who received ICI and had lipase follow-up values had IMPI; most were white (77%), males (66%) with a mean age of 57 years (Table 1). Seven patients (9%) had a history of pancreatitis, and 11 (13%) had pancreatic metastasis at the time of ICI initiation. Overall, 65% of patients received inhibitors of programmed death protein-1 or its ligand-1. The median duration from ICI initiation to IMPI onset was 5 months (SD, 7 months), and it was the shortest for patients who received cytotoxic T-lymphocyte-associated protein-4 (P=0.033; Table 2). Patients who had clinical symptoms of pancreatitis (n=24 [1%]) had higher levels of lipase (P=0.006) and amylase (P=0.029), more frequent imaging findings of pancreatitis (P=0.030), more frequent hospitalization (P<0.001) with treatment with intravenous fluids (P=0.004) and steroids (P=0.016; Table 3). We observed no differences of clinical characteristics between patients that had pancreatic metastasis and those that did not. Patients who developed long-term consequences of IMPI (chronic pancreatitis on imaging studies (n=3), recurrence of IMPI (n=3), and subsequent diabetes (n=6)) received ICI treatment for longer duration (P=0.006), had more frequently past history of smoking (P=0.048) or hyperlipidemia (P=0.021), received less intravenous fluids treatment (P=0.031), and resumed ICI treatment more frequently (P=0.017; Table 4). Patients who resumed ICI treatment had a trend for better survival rates (P=0.0559; Figure 1).

**Conclusions**

IMPI can present as a typical clinical scenario of acute pancreatitis, with the risk of pseudocyst, diabetes and chronic pancreatitis. ICI resumption in patients who had IMPI resulted in more long-term IMPI consequences, however, was associated with longer survival durations.

**References**

1. Cramer P, Bresalier R.S. Gastrointestinal and hepatic complications of immune checkpoint inhibitors. Curr Gastroenterol Rep, 2017. 19(1):3.2. 2. Wolchok, J.D., et al. Nivolumab plus ipilimumab in advanced melanoma. New England Journal of Medicine, 2013. 369(2):122-33.3. 3. Hofmann L, et al. Cutaneous, gastrointestinal, hepatic, endocrine, and renal side-effects of anti-PD-1 therapy. European Journal of Cancer, 2016. 60: 190-209.

**Ethics Approval**

This retrospective, single-center study was approved by the Institutional Review Board at The University of Texas MD Anderson Cancer Center (IRB No. PA18-0472).

**Consent**

This study was granted waiver for consent.

**Table 1 (abstract P645).**
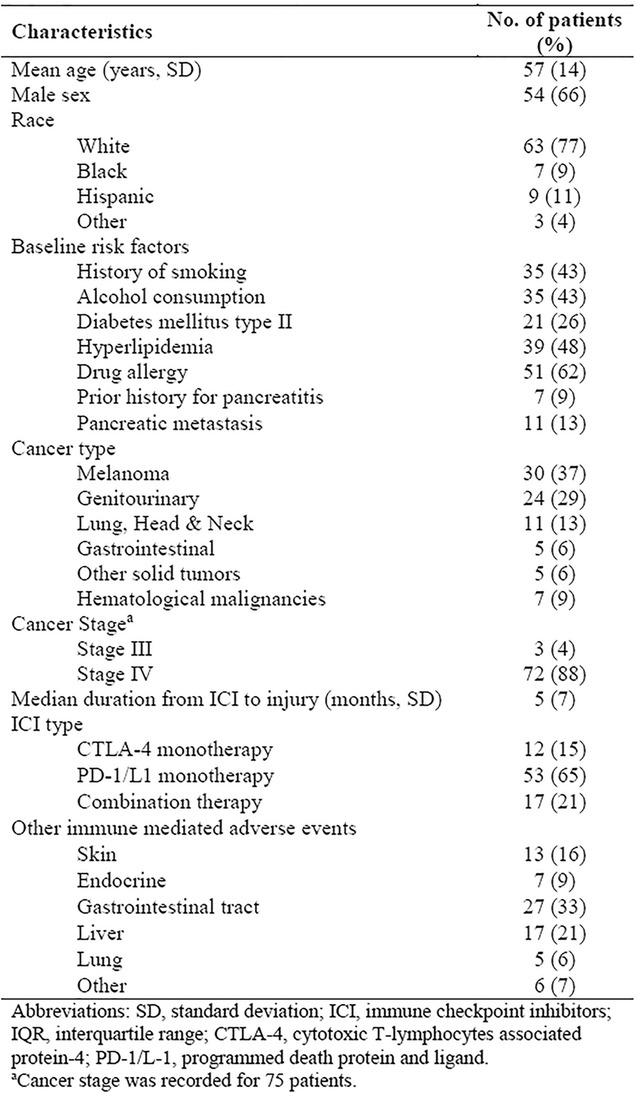
Baseline clinical characteristics

**Table 2 (abstract P645).**
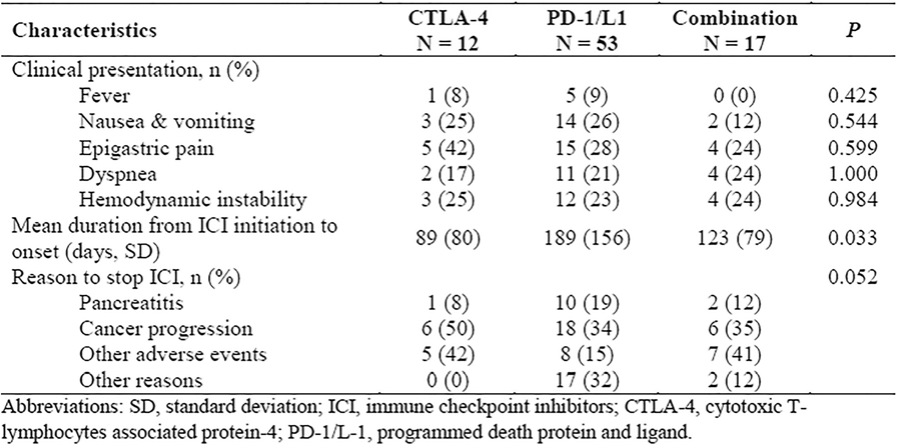
Clinical characteristics stratified by ICI regimen

**Table 3 (abstract P645).**
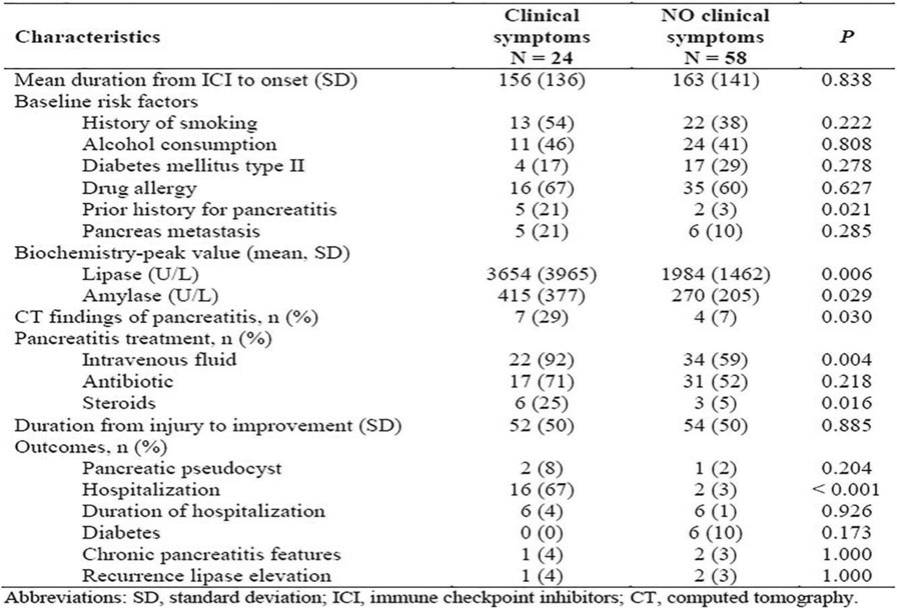
Characteristics of patients who had clinical symptoms diagnostic of pancreatitis

**Table 4 (abstract P645).**
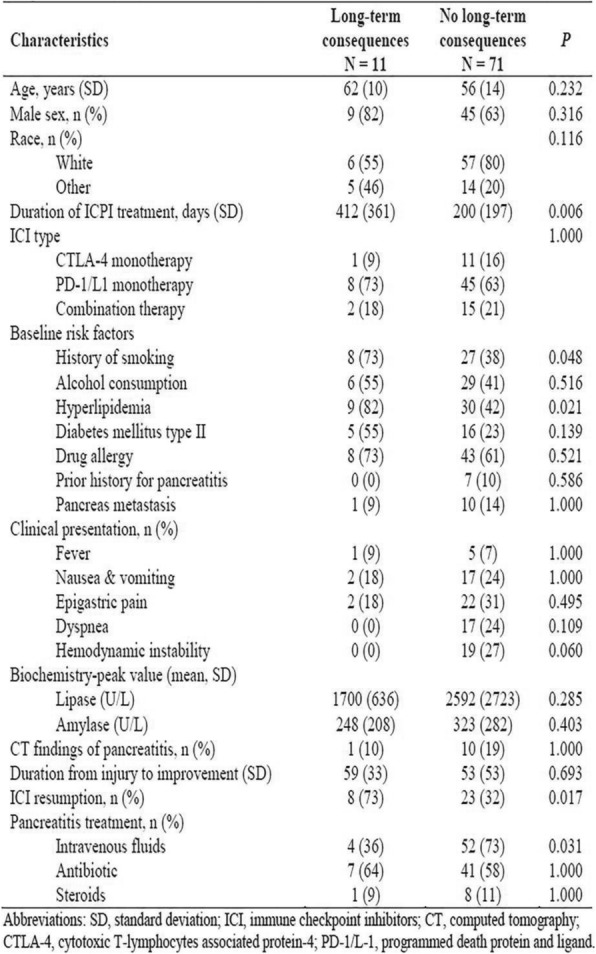
Clinical characteristics of patients who had long-term consequences of pancreatitis (chronic pancreatitis, diabetes, and recurrence)

#### P646 Discovery and characterization of therapeutic human monoclonal antibodies against 17 immuno-oncology targets using microfluidics and molecular genomics

##### Adam Adler, PhD, Rena Mizrahi, PhD, Carter Keller, MBA, David Johnson, PhD

###### GigaGen, South San Francisco, CA, USA

####### **Correspondence:** David Johnson (djohnson@gigagen.com)

**Background**

Monoclonal antibodies (mAbs) have proven to be extremely effective immuno-oncology therapeutics that lead to previously unseen durable responses in melanoma, bladder cancer, RCC, lung cancer, and other malignancies. Most mAb therapeutics, including current anti-PD-1 drugs, were discovered by mouse immunization followed by hybridoma isolation, a highly inefficient process that leads to few high affinity, developable antibodies. We recently developed a novel method for human mAb discovery that leverages humanized mice, microfluidics, multiplex PCR, yeast single chain variable fragment (scFv) display, and fluorescence activated cell sorting (FACS). Using this technology we have discovered thousands of high affinity antibodies against 17 immuno-oncology targets in less than 6 months.

**Methods**

We embarked on a discovery campaign for 17 immuno-oncology targets, including the antagonist targets PD-1, PD- L1, CTLA-4, LAG-3, TIM-3, B7-H3, B7-H4, TIGIT, CSF1R, VISTA, & CD47, and the agonist targets OX40, GITR, CD27, ICOS, TLR2, and TLR4. We immunized chimeric humanized mice (Trianni) with antigen twice weekly for four weeks. B cells were isolated from lymph nodes, spleen, and bone marrow, and >4 million single cells per target were processed through our microfluidic platform, generating DNA libraries that maintain proper heavy:light chain pairing. We built yeast scFv libraries for each of the 17 antigen-specific B cell repertoires and performed multiple rounds of FACS sorting to enrich for antigen-specific binders.

**Results**

We deep sequenced the libraries of enriched, high-affinity scFvs, which yielded the sequences for >2,800 candidate scFv binders. More than 400 of these candidate scFvs were engineered into full-length mAb constructs and expressed transiently in Chinese hamster ovary (CHO) cells. The affinity of individual mAbs ranged from 26 pM to 530 nM, with a median KD of 12.3 nM and ~10% with KD <1 nM. More than 55% of full-length antibodies were validated as specific binders to cell surface expressed target protein, and cell-based reporter assays for T cell activation revealed that ~60% of verified binders were functional. Epitope cross binning revealed extensive epitope diversity among the binders.

**Conclusions**

Candidates mAbs that are high affinity, bind cell surface antigen, and induce immune cell activation are now being tested in combinations or bispecific molecules for in vitro and in vivo efficacy. Combinations or bispecifics that show improved in vitro and in vivo efficacy over currently available checkpoint inhibitors will be moved forward into clinical studies.

#### P647 DuoBody-PD-L1x4-1BB combines checkpoint blockade and 4-1BB co-stimulation to promote antigen-specific T-cell stimulation and proliferation

##### Isil Altintas, PhD^1^, Alexander Muik, PhD^2^, Friederike Gieseke^2^, Theodora Salcedo^1^, Saskia Burm^1^, Mustafa Diken, PhD^2^, Christian Grunwitz^2^, Sebastian Kreiter, MD^2^, David Satijn, PhD^1^, Danita Schuurhuis^1^, Ozlem Tureci^2^, Esther Breij, PhD^1^, Ugur Sahin, MD^2^

###### ^1^Genmab, Utrecht, Netherlands; ^2^BioNTech, Mainz, Germany

####### **Correspondence:** Isil Altintas (ial@genmab.com)

**Background**

Currently pursued approaches in immuno-oncology involve either inhibition of immune checkpoints or activation of T cell co-stimulatory pathways. The concept of combining both modes-of-action within a single molecule, however, is so far not being clinically explored. Here, we introduce DuoBody-PD-L1x4-1BB (GEN1046), a first-in-class bispecific antibody for conditional engagement of T-cell co-stimulatory receptor 4-1BB in concert with PD-L1 checkpoint blockade.

**Methods**

DuoBody-PD-L1x4-1BB is an Fc-silenced IgG1 bispecific antibody that was obtained by controlled Fab-arm exchange of human(ized) PD-L1 and 4-1BB monoclonal antibodies. The binding characteristics of DuoBody-PD- L1-x4-1BB were assessed by flow cytometry, and functional characterization was performed using reporter assays and primary human T-cell assays. The effect on tumor-infiltrating lymphocytes (TIL) and their TCR repertoire was analyzed ex vivo in human primary tumor tissue cultures. In vivo activity of a surrogate mouse-reactive bispecific antibody was evaluated in both an OT-1 adoptive-cell-transfer model with ovalbumin vaccination and a CT26 mouse tumor model. The non-clinical safety profile of DuoBody-PD-L1x4-1BB was assessed in cynomolgus macaques.

**Results**

DuoBody-PD-L1x4-1BB showed dose-dependent binding to 4-1BB and PD-L1, with the PD-L1-specific arm being able to effectively antagonize PD-1:PD-L1 interactions. In T-cell stimulation assays with and without an active PD- 1:PD-L1 axis, DuoBody-PD-L1x4-1BB enhanced T cell proliferation and pro-inflammatory cytokine secretion. DuoBody-PD-L1x4-1BB showed superior potency when compared to PD-1:PD-L1 checkpoint blockade alone. The 4-1BB agonist activity of DuoBody-PD-L1x4-1BB was shown to be strictly conditional, depending on simultaneous binding of both targets. In fresh human tumor tissue cultures, DuoBody-PD-L1x4-1BB increased TIL expansion ex vivo. In vivo, the mouse surrogate PD-L1x4-1BB bispecific antibody, led to massive expansion of adoptively transferred ovalbumin specific T-cells upon ovalbumin vaccination. In a subcutaneous CT26 tumor model >80% of tumor-bearing mice experienced long-term tumor remission when treated with the surrogate bispecific antibody. Finally, DuoBody-PD-L1x4-1BB showed a favorable safety profile in cynomolgus macaque at doses up to 30 mg/kg.

**Conclusions**

This data supports that DuoBody-PD-L1x4-1BB induces conditional stimulation of T cells by blocking the inhibitory PD-1:PD-L1 axis and simultaneously inducing a co-stimulatory signal via 4-1BB. This unique and distinctive mechanism of action may result in potent anti-tumor immunity with an improved safety profile compared to using single PD-1/PD-L1 and 4-1BB antibodies as a combination. The clinical safety of DuoBody-PD-L1x4-1BB in patients with solid cancers will be assessed in a first-in-human clinical trial.

**Ethics Approval**

Cynomolgus macaque studies were performed at Charles River Laboratories, Tranent, UK, in accordance with the EU legislations described in Directive 2010/63/EU. This study was licensed by UK Home Office under the Animals (Scientific Procedures) Act 1986 (approval number PBAD559F8, Toxicology of Pharmaceuticals, Protocol 15) and overseen by Genmab B.V. The use of tumor tissue resections was approved by BioNTech AG’s Ethics Board, approval number 837.309.12 (8410-F).

#### P648 Different exhausted t-cell subsets exhibit different degrees of dysfunction in follicular lymphoma

##### Theodora Anagnostou, MD, Zhi Zhang Yang, MD, Hyo Jin Kim, PhD, Jose Villasboas, MD, Tammy Price- Troska, Shahrzad Jalali, PhD, Anne Novak, PhD, Stephen M. Ansell, MD, PhD

###### Mayo Clinic, Rochester, MN, USA

####### **Correspondence:** Stephen M. Ansell (ansell.stephen@mayo.edu)

**Background**

Although PD1 inhibitors represent a breakthrough in classical Hodgkin lymphoma, responses are substantially lower in follicular lymphoma (FL) (1). T-cell exhaustion plays an important role in the tumor microenvironment (TME) of FL and expression of exhaustion markers, such as PD1, TIM3 and LAG3, correlates with poor outcomes (2, 3). The reactivation potentials of different exhausted T-cell subsets in FL remain unknown. In this study we sought to determine different degrees of dysfunction in FL.

**Methods**

We developed a model to induce T-cell exhaustion in healthy donor T-cells isolated from peripheral blood and activated using IL-12, which allows us to manipulate the exhaustion phenotypes over time and mimics the exhaustion state present in FL. We assessed expression of exhaustion markers, cytokines and proliferation by flow cytometry. We compared these results to 6 samples from FL lymph nodes. To assess different degrees of dysfunction, we blocked PD1, TIM3 and LAG3 signaling alone and in combination.

**Results**

The ability of T-cells activated and treated with IL-12 to proliferate and produce perforin was lower compared to that of cells not treated with IL-12, confirming that IL-12 induces exhaustion (Figure 1a). IL-12 led to expression of PD1 ligands on the majority of PD1-expressing cells whereas TIM3 ligands were predominantly expressed on TIM3- cells. Of note, activation did not lead to co-expression of PD1/PD-L1 suggesting a possible autocrine mechanism of exhaustion (Figure 1b). Prolonged exposure to IL-12 led to increased expression of TIM3 over time, whereas PD1 expression occurred early and plateaued after day 6 of treatment (Figure 1c). LAG3 was mainly expressed on PD1+ cells and most were also TIM3+ (Figure 1d). Cells expressing PD1 had a higher degree of dysfunction compared to PD1-TIM3+ cells (Figure 2a) and TIM3 blockade led to better reversal of function compared to PD1 or LAG3 blockade (Figure 2b). Since PD1-TIM3+ cells appeared more functional, we tested the effect of blocking antibodies on them. TIM3 or LAG3 blockade were able to restore both proliferation and IFN-g production, but dual TIM3/LAG3 blockade did not have better effects than either blockade alone (Figure 3a). PD1- TIM3+ cells had the highest reactivation potential amongst other subsets (Figure 3b).

**Conclusions**

Different exhausted T-cell subsets exhibit different degrees of dysfunction in FL. Future studies should focus on identifying targets for immunotherapy on T-cell subsets with high reactivation potential.

**References**

1. Ding W, et al. PD-1 blockade with Pembrolizumab in relapsed low grade non-hodgkin lymphoma, blood. 2017; 130: 4055-55.
2. Yang, ZZ,et al. PD-1 expression defines two distinct T-cell sub-populations in follicular lymphoma that differentially impact patient survival., Blood Cancer J. 2015 5:e281.
3. Yang, ZZ, et al. 2017. Expression of LAG-3 defines exhaustion of intratumoral PD-1(+) T cells and correlates with poor outcome in follicular lymphoma., Oncotarget. 2015; 8:61425-39.

**Fig. 1 (abstract P648).**
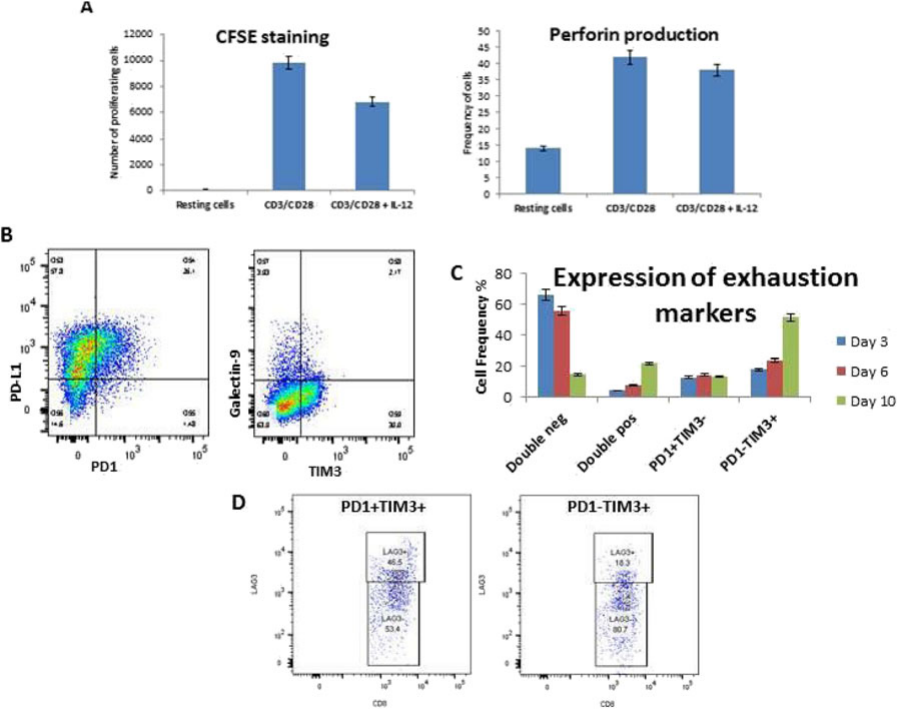
Effects of IL-12 on healthy T-cells

**Fig. 2 (abstract P648).**
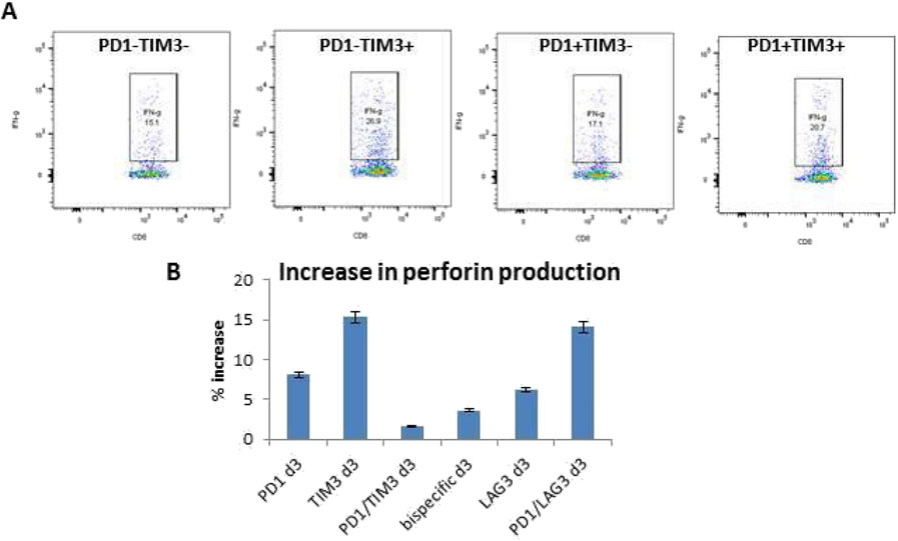
Different subsets of CD8+ cells classified

**Fig. 3 (abstract P648).**
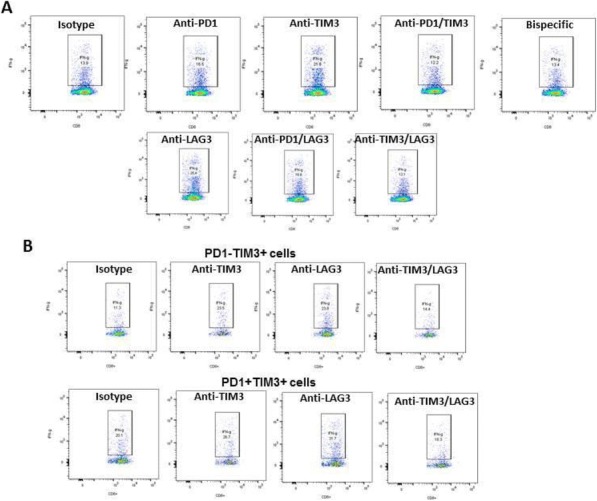
Effects of antibodies blocking different inhibitor

#### P649 Resistance to PD1 blockade in the absence of metalloprotease-mediated LAG3 shedding

##### Lawrence Andrews, PhD^1^, Ashwin Somasundaram, MD^1^, Jessica Moskovitz, MD^1^, Andrea Szymczak-workman^1^, Chang Liu, PhD^1^, Anthony Cillo, PhD^1^, Brenda Kurland^1^, Huang Lin^1^, Kelly Moynihan, PhD^2^, Darrell Irvine, PhD^2^, Robert Ferris, MD, PhD^1^, Tullia Bruno, PhD^1^, Creg Workman, PhD^1^, Dario Vignali, PhD^1^

###### ^1^University of Pittsurgh, Pittsburgh, PA, USA; ^2^Massachusetts Institute of Technology, Cambridge, MA, USA

####### **Correspondence:** Dario Vignali (dvignali@pitt.edu)

**Background**

Inhibitory receptors (IRs) prevent exacerbated T cell activation, yet can be hijacked by tumors for protection from immune attack. LAG3 co-expression with PD1 defines functionally exhausted T cells, with combinatorial blockade against these IRs in clinical trial. However, LAG3 expression and function is itself regulated by ADAM10/17- mediated cell surface cleavage at the connecting peptide. Our previous studies have shown that non-cleavable LAG3 (LAG3.NC) mutant T cells exhibit reduced cytokine production and proliferation in vitro, prompting us to examine whether LAG3 shedding from the surface of T cells is necessary to mount an effective antitumor immune response in vivo [1].

**Methods**

A conditional knock-in mouse was generated to restrict LAG3.NC to all T cells (CD4Cre) or specifically CD8+(E8ICre.GFP), CD4+ (ThPOKCreERT2) or CD4+Foxp3+ populations (Foxp3CreERT2), by Cre-LoxP-mediated replacement of the connecting peptide with a mutant form. Moreover, LAG3 and ADAM10 expression was assessed to understand the translational relevance of LAG3 shedding in a cohort of patients with head and neck squamous cell carcinoma (HNSCC).

**Results**

LAG3.NC-CD4Cre and LAG3.NC-ThPOKCreERT2 mice resist anti-PD1 therapy and succumb to MC38 tumor growth, compared to ~40% of controls and LAG3.NC-E8ICre.GFP mice becoming tumor-free. This phenotype is effector CD4+ T cell-mediated, and not Treg-mediated, as LAG3.NC-Foxp3CreERT2 mice clear tumors. Intrinsically, CD4+Foxp3– TIL isolated from LAG3.NC-CD4Cre mice show reduced proliferation and IFN-g/TNF- a compared with controls. CD8+ TIL also show reduced cytokine release, extrinsically affecting CD8+ pmel-1 T cells in an adoptive transfer model into B16-gp100-bearing LAG3.NC-CD4Cre hosts. Selective ADAM10 inhibition in vivo, also reduces IFN-g/TNF-a proposing that metalloproteinase-mediated LAG3 cleavage limits an antitumor immune response. This is clinically relevant as HNSCC patients show reciprocal expression of LAG3 and ADAM10 that is particularly evident on CD4+ T cells. This inverse correlation shown in patients with high LAG3 and low ADAM10 expression on CD4+Foxp3– T cells in peripheral blood is associated with higher HNSCC disease stage and reduced patient survival.

**Conclusions**

Overall, these data suggest that failure of LAG3.NC-CD4Cre/ThPOKCreERT2, but not LAG3.NC-E8ICre/Foxp3CreERT2, mice to clear MC38 tumors suggests that LAG3 shedding on CD4+Foxp3– T cells is necessary for CD8+ TIL to mediate an effective antitumor immune response. Indeed, HNSCC patients with higher LAG3 expression on CD4+Foxp3– T cells, due to lower ADAM10 expression, have poorer prognosis. Understanding the role of LAG3 shedding on T cells is thus clinically relevant, and may be useful as a potential biomarker for patient responsiveness to anti-PD1 immunotherapy.

**References**

1. Li N, Wang Y, Forbes K, Vignali KM, Heale BS, Saftig P, Hartmann D, Black RA, Rossi JJ, Blobel CP, Dempsey PJ, Workman CJ, Vignali DAA. Metalloproteases regulate T-cell proliferation and effector function via LAG-3. EMBO J. 2007; 26, 494-504..

#### P650 Selective CD47 immune checkpoint targeting on tumor cells modulates the tumor microenvironment to enhance macrophage tumoricidal function

##### Vanessa Buatois, PhD, Xavier Chauchet, PhD, Laura Cons, Laurence Chatel, Limin Shang, PhD, Marie Kosco- Vilbois, Krzysztof Masternak, PhD, Nicolas Fischer, PhD, Walter Ferlin, PhD

###### Novimmune SA, Geneva, Switzerland

####### **Correspondence:** Vanessa Buatois (vbuatois@novimmune.com)

**Background**

CD47 is a membrane protein overexpressed on tumor cells and considered as an innate immune checkpoint. CD47 interacts with SIRPα on myeloid cells and induces a “don’t eat me” signal that allows healthy cells to limit elimination by immune cells, in particular macrophages. CD47 upregulation in solid and hematological cancers is correlated with poor clinical prognosis, almost certainly by allowing tumor cells to escape immune surveillance. Blocking the CD47-SIRPα axis is thus an attractive approach to foster tumor cell killing by tumor-associated macrophages (TAMs) and boost cross-priming of anti-tumor T cells by dendritic cells (DC). However, CD47 targeting is hindered by haematological toxicity due to its ubiquitous expression. To focus CD47 blockade on tumor cells, we generated two bispecific antibodies (biAbs) pairing a high affinity arm targeting a tumor associated antigen (TAA), i.e., CD19 or mesothelin (MSLN), to an optimized lower affinity arm targeting CD47 and investigated their efficacy in xenograft tumor models.

**Methods**

BiAbs targeting CD47xCD19 or CD47xMSLN were tested in immunodeficient mice implanted with the following human tumor cell lines: Raji (CD19+, lymphoma), OVCAR-3 (MSLN+, ovarian) or MSLN-transfected HepG2 (hepatic). Analysis by flow cytometry and Nanostring technology was used to evaluate the impact of the biAbs on the tumor microenvironment. The contribution of macrophages to tumor growth inhibition was addressed by clodronate-mediated depletion. Finally, biAbs-stimulated cross-priming of anti-tumor T-cells by DC was assessed in vitro.

**Results**

BiAbs controlled the growth of CD19+ lymphomas and MSLN+ tumors. In the Raji xenograft model, treatment with the CD47xCD19 biAb did not increase the number of TAMs, but enhanced their tumoricidal activity (i.e., more macrophages engulfing tumor cells). The treatment also reduced the proportion of CD11b+Ly6g+ granulocytic myeloid-derived suppressor cells. In contrast, in the MSLN-transfected HepG2 xenograft model, treatment with the CD47xMSLN biAb induced a significant increase of macrophages in the tumor. Depletion of phagocytic cells with clodronate impaired the anti-tumor activity of the biAbs, highlighting the role of macrophages. Finally, the biAbs promoted cross-priming of tumor antigen-specific T-cells, suggesting the potential of CD47xTAA biAbs to enhance anti-tumor T cell responses.

**Conclusions**

CD47 targeting biAbs, tethered to cancer cells by their TAA binding arm, remodel the tumor microenvironment enhancing macrophage tumoricidal function. Furthermore, the ability for the biAbs to amplify DC-induced cross-priming of tumor antigen to T-cells further augments the antitumor efficacy potential of such molecules.

#### P651 First-in-human study of FAZ053, an anti-PD-L1 mAb, alone and in combination with spartalizumab, an anti- PD-1 mAb, in patients with advanced malignancies

##### Janis Callister^1^, Filip Janku, MD, PhD^2^, David Tan^3^, Juan Martin-Liberal^4^, Shunji Takahashi^5^, Ravit Geva, MD^6^, Ayca Gucalp^7^, Xueying Chen^8^, Kulandayan Subramanian^9^, Jennifer Mataraza^9^, Jennifer Wheler^9^, Philippe Bedard, MD^10^

###### ^1^Articulate Science, Manchester, UK; ^2^MD Anderson Cancer Center, Houston, TX, USA; ^3^National University Cancer Institute, Singapore, Singapore ^4^Vall d’Hebron Institute of Oncology, Barcelona, Spain; ^5^The Cancer Institute Hospital of JFCR, Tokyo, Japan; ^6^Tel Aviv Sourasky Medical Center, Tel-Aviv, Israel; ^7^Memorial Sloan Kettering Cancer Center, New York, NY, USA ^8^Novartis Pharmaceuticals Corporation, East Hanover, NJ, USA ^9^Novartis Institutes for BioMedical Resea, Cambridge, MA, USA; ^10^Princess Margaret Cancer Centre, Toronto, ON, Canada

####### **Correspondence:** Filip Janku (fjanku@mdanderson.org)

**Background**

FAZ053 and spartalizumab are humanized immunoglobulin G4 monoclonal antibodies (mAbs) that bind anti-programmed death ligand-1 (PD-L1) and programmed death-1 (PD-1), respectively. We report the dose-escalation results from an ongoing Phase I study of FAZ053 ± spartalizumab in patients with advanced malignancies, enriched for patients with chordoma, a rare subtype of sarcoma.

**Methods**

Patients received escalating doses of single-agent (SA) FAZ053 intravenously once every 3 weeks (Q3W) or 6 weeks (Q6W), or FAZ053 + spartalizumab Q3W. The primary objective was to assess the safety and tolerability of FAZ053 ± spartalizumab, and determine recommended doses for expansion (RDEs). Dose escalation was guided by an adaptive Bayesian logistic regression model following the escalation with overdose control principle.

**Results**

=As of the data cutoff of March 30, 2018, 61 patients received SA FAZ053 at doses 80–1600 mg Q3W or 800–1600 mg Q6W. Most patients (n=54; 89%) received prior treatment; 1 (2%) received prior anti-PD-1. FAZ053 exposure was generally dose proportional, with terminal half-life of ~16–18 days. A dose-limiting toxicity occurred in 1 patient (Grade 4 renal failure; FAZ053 1600 mg Q6W). RDE was determined to be 1200 mg Q3W or 1600 mg Q4W. Adverse events (AEs) of all grades assessed as possibly related to treatment were reported for 33 patients (54%); most commonly (≥10%) fatigue (n=11; 18%) and pruritus (n=8; 13%); 4 patients (7%) had Grade 3/4 treatment-related AEs, including elevated amylase (3%), renal failure, elevated lipase, elevated AST, and elevated blood CPK (each 2%). For these patients treated with SA FAZ053, partial responses (PRs) were demonstrated in 4 patients (7%) with chordoma, alveolar soft part sarcoma (ASPS), poorly differentiated carcinoma of scalp, and penile squamous cell carcinoma (duration of treatment 5.5–12.5 months; all with treatment ongoing). Among 5 patients with chordoma treated with SA FAZ053, 1 patient has a PR, treatment ongoing >12 months, and 4 patients have stable disease ongoing (+4% to –29%). Data for 57 patients treated with combination FAZ053 (20–1200 mg) + spartalizumab 300 mg Q3W are preliminary. Updated results and biomarker data for patients receiving SA and combination treatment, including additional patients with chordoma, will be presented.

**Conclusions**

SA FAZ053 was well tolerated and the RDE was determined to be 1200 mg Q3W. Clinical activity was observed in a range of indications including chordoma, a rare tumor without standard therapy options.

**Trial Registration**

www.clinicaltrials.gov; NCT02936102

**Ethics Approval**

This study was approved by an independent ethics committee or institutional review board at each site.

#### P652 Cytokine-mediated induction of PD-L1 expression on tumor and immune cells

##### Shuming Chen, PhD, George Crabill, MS, Theresa Pritchard, MS, Tracee McMiller, MS, Drew Pardoll, MD, PhD, Fan Pan, Suzanne Topalian, MD

###### Johns Hopkins University School of Medicine, Sidney Kimmel Comprehensive Cancer Center, and Bloomberg~Kimmel Institute for Cancer Immunotherapy, Baltimore, MD, Canada

####### **Correspondence:** Shuming Chen (schen72@jhmi.edu)

**Background**

The PD-1/PD-L1 checkpoint is a central mediator of immunosuppression in the tumor immune microenvironment (TME). To characterize factors regulating PD-L1 expression on tumor and/or immune cells, we investigated the effects of TME-resident cytokines and the role of transcription factors in constitutive and cytokine-induced PD-L1 expression.

**Methods**

Thirty-four cultured human tumor lines derived from 18 melanomas (MEL), 12 renal cell carcinomas (RCC), and 4 squamous cell carcinomas of the head and neck (SCCHN), and normal donor peripheral blood monocytes (Monos), were treated with cytokines that we found expressed in the PD-L1+ TME by gene expression profiling (GEP), including IFN-g, IL-1a, IL-10, IL-27 and IL-32g. PD-L1 cell surface protein expression was detected by flow cytometry, and mRNA by quantitative RT-PCR. Total and phosphorylated STAT1, STAT3, p65 and c-jun proteins were detected by Western blotting. STAT1, STAT3, p65 and c-jun were knocked down with siRNAs. The proximal promoter region of CD274 (PD-L1) was sequenced in all cultured tumor lines.

**Results**

PD-L1 protein was not constitutively expressed on cultured MELs, but was found on some RCCs (n=6) and SCCHNs (n=3). Brief IFN-g exposure induced PD-L1 on MELs, and induced or upregulated PD-L1 on RCCs and SCCHNs. Among other TME-resident cytokines detected in human PD-L1+ tumor biopsies by GEP, IL-27 and IL- 1a increased PD-L1 expression on cultured tumor lines, alone or in combination with IFN-g. In short-term cultured Monos, PD-L1 expression was induced by these and additional cytokines, including IL-10 and IL-32g. Changes in PD-L1 protein expression (FACS) correlated with mRNA levels, suggesting cytokine regulation primarily via mRNA transcription and not via protein translocation. siRNA knockdown of STAT1 but not STAT3 reduced IFN-g- and IL-27-induced PD-L1 protein expression on tumor lines and Monos. In contrast, STAT3 knockdown in Monos reduced IL-10-induced PD-L1 protein expression, and p65 knockdown in tumor lines reduced IL-1a-induced PD-L1 expression. Notably, constitutive PD-L1 expression was not affected by knocking down STAT1, STAT3, p65 or c- jun. Differential effects of IFN-g, IL-1a, and IL-27 were not due to CD274 promoter polymorphisms or mutations.

**Conclusions**

Multiple cytokines play important and cell type-selective roles in promoting PD-L1 expression in the TME.

Activation of STAT1, STAT3 or p65 was associated with PD-L1 expression in response to distinct cytokines. Factors driving constitutive tumor cell PD-L1 expression were not identified in this study. However, in many cancers, adaptive expression of PD-L1 on tumor and/or stromal cells reflects an immune-reactive TME that can be unleashed by anti-PD-1/PD-L1 therapy.

**Acknowledgements**

Supported by NCI R01 CA142779 and the Johns Hopkins Bloomberg~Kimmel Institute for Cancer Immunotherapy.

#### P653 Discovery of a novel TIGIT therapeutic antibody with strong efficacy in tumor xenografts as monotherapy

##### Feifei Cui, PhD, Lei Fang, Zhengyi Wang, Taylor Guo, Jingwu Zhang, Feifei Cui, PhD

###### I-Mab Biopharma, Shanghai, China

####### **Correspondence:** Jingwu Zhang (jingwu.zang@i-mabbiopharma.com); Feifei Cui

**Background**

The immune checkpoint co-inhibitory receptor TIGIT (T cell immunoglobulin and immunoreceptor tyrosine-based inhibitory motif) is expressed on activated CD4+ T, CD8+ T and NK cells and on regulatory T cells (Tregs). Blocking TIGIT interaction with its ligand CD155 can re-energize tumor antigen-specific CD8+ T cells, unleash NK cells and inhibit Treg-mediated immunosuppression in the tumor microenvironment. Previous reports showed strong efficacy of anti-TIGIT antibodies in combination with anti-PD(L)1 agents in multiple tumor models, indicating a potential for TIGIT blocking antibodies as a combination partner for cancer treatment. Here we report discovery of a novel, humanized, Fc-enabled, subnanomolar anti-TIGIT antibody with significantly enhanced tumor inhibition as a monotherapy, thus differentiating it from current anti-TIGIT molecules.

**Methods**

TJT6 was generated by traditional hybridoma technology using extracellular domain (ECD) of human TIGIT as immunogen and then humanized through complementarity determining region (CDR) grafting. The TIGIT antigen binding and CD155 receptor blocking activities of TJT6 were evaluated for through both protein-based and cell- based assays. The immunological function of TJT6 was further studied in a TIGIT- and its counterreceptor CD226- overexpressed Jurkat cell activation assay. The in vivo efficacy of TJT6 was investigated in MC38 tumor model using human TIGIT knock-in mice. Percentages of tumor infiltrating CD4+ T and CD8+ T cells and the production of interferon gamma (IFN-gamma) were analyzed by flow cytometer.

**Results**

TJT6, an Fc-enabled human IgG1 antibody, binds human and cynomolgus TIGIT with similar sub-nanomolar binding. It blocked the interaction of TIGIT to its ligand CD155, leading to increased production of interleukin 2 (IL-2) in a Jurkat cell line which overexpressed TIGIT and CD226. Mono-treatment of TJT6 significantly suppressed tumor growth in a dose-dependent manner in a syngeneic MC38 tumor model compared to vehicle, achieving maximal 80% tumor growth inhibition at 10 mg/kg. Percentages of tumor infiltrating CD4+ and CD8+ T cells and the production of IFN-gamma in CD8+ T cells were significantly enhanced after anti-TIGIT antibody treatment.

**Conclusions**

We have successfully discovered a TIGIT-blocking therapeutic antibody with strong efficacy in the inhibition of tumor growth and reinvigoration of tumor-infiltrating CD8+ T cells. TJT6 represents potentially a best-in-class molecule with superior potency as a single agent and is undergoing preclinical development with an aim to enter clinical studies in 2019.

#### P654 T-cell profiling in cancer tissue with multiplexed IHC using UltiMapper™ I/O assays

##### Alexis Wong, PhD, Alexis Wong, PhD, Amy Zhang, MS, Max Rubinstein, Laura Sciarra, PhD, Chakib Boussahmain, BS, Bonnie Phillips, PhD, Katir Patel, PhD, Sean Downing, PhD, Stephanie Hennek, PhD

###### Ultivue, Cambridge, MA, USA

####### **Correspondence:** Sean Downing (sean.downing@ultivue.com)

**Background**

With high spatial heterogeneity and biological complexity, resected tumor samples present a significant characterization challenge, particularly when samples are scarce. Multiplexing in a single, intact tissue sample is required to identify cell types, understand biological pathways, and map cellular interactions. Ultivue has developed a portfolio of multiplexed immunofluorescence tissue imaging assays (UltiMapper assays) that simultaneously enable whole-slide imaging and integrate seamlessly with existing instrumentation to address key questions in immuno-oncology. This work highlights the application of UltiMapper assays in human tumor samples to profile and phenotype T-cells – including regulatory T-cells, activated T-cells, exhausted T-cells, and memory T-cells. Each T-cell subtype plays an important role in the immune response to cancer and determining the presence and interactions of each T-cell population drives a deeper understanding of the biological mechanisms at work.

**Methods**

UltiMapper assays were used to perform multiplexed IHC on deidentified FFPE tissue samples using control tissues (tonsil) and on multiple tumor types (lung, melanoma, colon, or breast). Three separate panels were run on serial sections. The UltiMapper I/O T-Reg panel included the markers CD4, CD25, and FoxP3, as well as a tumor markers pan-cytokeratin (CK) for carcinomas and Sox10 for melanomas. The UltiMapper I/O T-Act panel included markers CD3, Granzyme B, Ki67, CK, and SOX10. The UltiMapper I/O PD-1 panel included markers CD3, CD45RO, PD- 1, CK, and SOX10. Staining was performed manually or using the Leica Bond Rx™ autostainer. Imaging was performed on various tissue scanners including the Zeiss Axio Scan.Z1, and image analysis was performed using HALO from Indica Labs.

**Results**

Each marker in the UltiMapper panels was individually compared at the singleplex level to reference IHC assays to confirm staining specificity. Serial tissue sections stained with the UltiMapper panels were found to have good reproducibility. In tumor samples, a range of abundance and expression of infiltrating T-cells was observed. Cell counts for all possible phenotypes were obtained for each panel to identify regulatory T-cells, activated T-cells, exhausted T-cells, and memory T-cells. Spatial analysis was employed to map the degree of T-cell infiltration between tissue samples.

**Conclusions**

Target specificity was confirmed for the UltiMapper I/O T-Reg, T-Act, and PD-1 panels. UltiMapper assays are proficient in maintaining tissue integrity and identifying several subsets of T-cells within and surrounding tumor sections. The UltiMapper assay can be implemented by any lab possessing a standard fluorescent microscope and does not rely on the purchase of, nor access to, more expensive platforms.

#### P655 Withdrawn

#### P656 ATOR-1017, a tumor directed Fcγ-receptor cross linking dependent 4-1BB agonistic antibody

##### Karin Enell Smith, PhD, Anna Rosén, Karin Barchan, Ida Aberg, Doreen Werchau, BS, Mia Thagesson, Anna Dahlman, Niina Veitonmaki, Christina Furebring, PhD, Peter Ellmark, PhD

###### Alligator Bioscience, Lund, Sweden

####### **Correspondence:** Karin Enell Smith (kae@alligatorbioscience.com)

**Background**

4-1BB is an inducible costimulatory receptor expressed on activated T and natural killer (NK) cells. Activation of 4-1BB results in improved survival, proliferation and enhanced effector functions of the T and NK cells. Treatment with 4-1BB antibodies in preclinical mouse tumor models leads to tumor eradication and induction of long-term immunity. 4-1BB is a promising candidate for immunotherapy, however 4-1BB antibodies in clinical development, have either been suffering from poor efficacy or toxicity. ATOR-1017 is a 4-1BB ligand-blocking IgG4 antibody, designed for improved safety and enhanced efficacy. The functional activity is dependent on engagement with Fcγ receptors (FcγRs), directing the immune activation to the tumor area where 4-1BB as well as certain FcγRs are highly expressed.

**Methods**

ATOR-1017 was characterized using human primary cells isolated from leukocyte concentrates from healthy donors or 4-1BB reporter assays co-cultured with FcγR-expressing cells. The in vitro effects were measured using either ELISA or FACS. Co-expression of 4-1BB, FcγRI and FcγRIIb was assessed on human tumor tissue samples by immunohistochemistry (IHC).

**Results**

ATOR-1017 blocks the 4-1BBL binding to the 4-1BB receptor and binds to a unique epitope compared to the 4-1BB antibodies currently in clinical development. Co-stimulation of human primary CD8+ T cells with ATOR-1017 induces a potent CD8+ T cell activation only after cross-linking by FcγRI or FcγRIIb. Similarly, ATOR-1017 induces an FcγR-dependent cytotoxic phenotype in NK cells. The importance of the improved cytotoxic profile of NK cells is further supported by combining ATOR-1017 with a tumor targeting antibody, which synergistically enhances the induction of antibody-dependent cell-mediated cytotoxicity (ADCC).Co-expression of FcγRs and 4- 1BB is required for induction of a tumor-directed immune response. This was demonstrated in solid tumors as well as lymphomas, using IHC multiplex staining of 4-1BB, FcγRI and FcγRIIb. Tumor tissue from several tumor indications demonstrated a high and co-localized expression of the targets, supporting a tumor-directed immune response of ATOR-1017.

**Conclusions**

ATOR-1017 is a FcγR crosslinking dependent 4-1BB agonistic antibody with an activation profile that minimize the risk for inducing systemic immune activation and toxicity. ATOR-1017 was designed for an optimal efficacy and improved safety, by combining the IgG4-format that mediates a potent FcγR cross-linking with a unique binding epitope on 4-1BB. The immune activation will be directed to tumors co-expressing both specific FcγRs and 4-1BB, which are potential biomarkers for patients as well as tumor indication selection. ATOR-1017 is currently in preclinical development phase and clinical studies are planned for 2019

**Ethics Approval**

This study was approved by Lund/Malmö Ethics Board approval number M142-15.

#### P657 Agonist redirected checkpoint platform (ARC), engineering bi-functional fusion proteins (PD1-Fc-OX40L), for cancer immunotherapy

##### George Fromm, PhD^1^, Suresh de Silva, PhD^2^, Taylor Schreiber, MD, PhD^2^

###### ^1^Shattuck Labs, Inc, Apex, NC, USA; ^2^Shattuck Labs, Inc., Durham, NC, USA

####### **Correspondence:** George Fromm (gfromm@shattucklabs.com)

**Background**

Current combination immunotherapy with bispecific antibodies, linked scFv's or T cell engagers have not been able to both block checkpoints and agonize TNF receptors; likely because these molecules lose target avidity when engineered to bind multiple targets with monovalent antigen binding arms. Fusion proteins incorporating the extracellular domain (ECD) of type I membrane proteins (e.g., Enbrel, Orencia) or type II membrane proteins (e.g., OX40L-Fc, GITRL-Fc), linked to the hinge-CH2-CH3 domain of antibodies are both functional, despite the fact that their ECDs are in opposite orientations. Here we report the generation of a two-sided fusion protein platform linking type I ECDs to type II ECDs by a central domain (Agonist Redirected Checkpoint, ARC), and present preclinical findings on one of the PD1-containing molecules in our pipeline; PD1-Fc-OX40L.

**Methods**

Human and mouse PD1-Fc-OX40L were produced and characterized using a range of biochemical assays to determine molecular weight, subunit composition & binding affinity; molecular assays to characterize in vitro/ex vivo binding & functional activity; and anti-tumor efficacy in multiple syngeneic tumor models. Human PD1-Fc- OX40L completed GMP manufacturing and GLP NHP toxicity studies, with anticipated IND filing in Q4 2018.

**Results**

The PD-1 end of the ARC binds immobilized PD-L1 and PD-L2 at 2.08 and 1.76 nM affinity, respectively, and binds PD-L1 on human tumor cells in vitro and in vivo. The OX40L end of the ARC binds immobilized OX40 at 246 pM affinity and binds OX40 on primary T cells. High binding affinity on both sides of the construct translated to potent stimulation of OX40 signaling and PD1:PD-L1/L2 blockade, in multiple in vitro assays, including improved potency as compared to pembrolizumab, nivolumab, tavolixizumab and combinations of those antibodies. Furthermore, when activated human T cells were co-cultured with PD-L1 positive human tumor cells, PD1-Fc- OX40L was observed to concentrate within the immune synapse, which enhanced proliferation of T cells and production of IL-2, IFNγ and TNFα; leading to efficient killing of tumor cells. The therapeutic activity of PD1-Fc- OX40L in established murine tumors was superior to PD1 blocking, OX40 agonist, or combination antibody therapy. Importantly, all agonist functions of PD1-Fc-OX40L are independent of Fc receptor cross-linking.

**Conclusions**

These data demonstrate feasibility and function of a novel chimeric fusion protein platform, providing checkpoint blockade and TNF superfamily costimulation in a single molecule, which is uniquely advantageous because the construct links those two signals in the same microenvironment, at the time in which T cells are engaging cognate tumor antigens.

#### P658 Agonist redirected checkpoint platform (ARC), engineering bi-functional fusion proteins (TIM3-Fc-OX40L and TIM3-Fc-CD40L), for cancer immunotherapy

##### George Fromm, PhD^1^, Suresh de Silva, PhD^2^, Taylor Schreiber, MD, PhD^2^

###### ^1^Shattuck Labs, Inc, Apex, NC, USA; ^2^Shattuck Labs, Inc., Durham, NC, USA

####### **Correspondence:** George Fromm (gfromm@shattucklabs.com)

**Background**

Current combination immunotherapies with bispecific antibodies, linked scFv's or T cell engagers have not been able to both block checkpoints and agonize TNF receptors. This is likely because these molecules lose target avidity when engineered to bind multiple targets with monovalent antigen binding arms. Fusion proteins incorporating the extracellular domain (ECD) of type I membrane proteins (e.g., Enbrel, Orencia) or type II membrane proteins (e.g., OX40L-Fc, GITRL-Fc), linked to the hinge-CH2-CH3 domain of antibodies are both functional, despite the fact that their ECDs are in opposite orientations. Here we report the generation of a two-sided fusion protein platform that links type I ECDs to type II ECDs by a central domain (Agonist Redirected Checkpoint, ARC), and present preclinical findings on two molecules in our pipeline; TIM3-Fc-OX40L and TIM3-Fc-CD40L.

**Methods**

Shattuck synthesizes both murine and human versions of ARCs, and assesses them using biochemical assays to determine molecular weight, subunit composition & binding affinity; molecular assays to characterize in vitro/ex vivo binding, in vitro functional activity; and anti-tumor efficacy in multiple syngeneic tumor model systems. TIM3- Fc-OX40L & TIM3-Fc-CD40L have advanced into cell line development and early manufacturing.

**Results**

The TIM3 end of the fusion protein binds GAL9 and phosphtidylserine (PS) on human tumor cells. The OX40L/CD40L ends bind OX40 and CD40, respectively, on the surface of primary PBMCs. Both TIM3-Fc-OX40L & TIM3-Fc-CD40L activate NF B signaling in cells engineered to overexpress OX40 or CD40 and an NF B- luciferase reporter, and also in NIK signaling reporter cells. Each TIM3-containing ARC induces a unique cytokine signature in PBMCs incubated with the super-antigen Staphylococcal enterotoxin B, or when cultured in AIMV media. In vivo, the therapeutic activity of TIM3-Fc-OX40L and TIM3-Fc-CD40L in established murine MC38 and CT26 tumors was superior to TIM3-blocking antibody, OX40-/CD40-agonist antibody monotherapies, and the respective correlating combination antibody therapy. Importantly, a pharmacodynamic biomarker of tumor rejection was identified by coordinated elevations in serum IFNγ, IL-2, IL-4, IL-5, IL-6 and IL-17A. Interestingly, therapeutic activity (anti-tumor in mice or human cytokine secretion in the SEB assay) was enhanced when ARCs were combined with antibody blockade of PD1.

**Conclusions**

These data demonstrate feasibility and functional activity of a novel chimeric fusion protein platform, providing checkpoint blockade and TNF superfamily costimulation in a single molecule, which is uniquely advantageous because the construct links those two signals in the same microenvironment, at the time in which T cells are engaging cognate tumor antigens.

#### P659 AGEN2373 is a conditionally-active agonist antibody targeting the co-stimulatory receptor CD137 for the treatment of human malignancies

##### Claire Galand, PhD, David Savitsky, Vignesh Venkatraman, Min Lim, Rebecca Ward, MSc, Nicholas Wilson, Christina Riordan, Matthew Costa, Randi Gombos, PhD, Benjamin Morin, PhD, Dhan Chand, PhD, Claire Galand, PhD

###### Agenus, Lexington, MA, USA

####### **Correspondence:** David Savitsky (david.savitsky@agenusbio.com)

**Background**

CD137 is a co-stimulatory member of the tumor necrosis factor receptor superfamily (TNFRSF) that is expressed by discrete populations of T and natural killer (NK) cells. Agonist antibodies targeting CD137 are potent inducers of tumor-reactive T cell proliferation, cytokine production and cytotoxicity, and have been explored clinically for their ability to enhance immune cell-mediated destruction of tumor cells. Despite early signs of clinical activity, the development of first generation anti-CD137 antibody has been hampered by on-target dose-limiting hepatotoxicity. Next-generation CD137 therapies have therefore sought to localize CD137 agonism to the tumor microenvironment (TME) to potentially improve tolerability in patients. However, these approaches may also limit the potential benefit of CD137 signaling outside the TME that contributes to anti-tumor immunity, such as T cell priming in tumor draining lymph nodes. Here we describe the pharmacologic and non-clinical safety profile of AGEN2373, a novel anti-CD137 antibody designed to provide potent CD137 co-stimulation in the presence of CD137 ligand and Fc gamma receptor-expressing antigen presenting cells. Importantly, AGEN2373 was well-tolerated in non-human primates, therefore supporting the potential for a therapeutic window in patients either as a monotherapy, or in combination with immune checkpoint blockade.

**Methods**

Binding assay; Pharmacologic and non-clinical safety study in non human primates; Signaling reporter assay; Cytokine secretion assay

**Ethics Approval**

Non human primate and rodent studies were approved by Agenus Institutional Animal Care and Use Committee (IACUC).

#### P660 A novel bispecific agent targeting PD-L1 and IL-15/IL-15Ralpha promotes potent anti-tumor efficacy in multiple models of human solid carcinomas

##### Karin Knudson, PhD^1^, Kristin Hicks, PhD^1^, Yohei Ozawa, MD, PhD^1^, Jeffrey Schlom, PhD^1^, John Lee, MD^2^, Shahrooz Rabizadeh, PhD^3^, Patrick Soon-Shiong, MD, FRCS, FACS^4^, Sofia Gameiro, PharmD, PhD^1^

###### ^1^National Cancer Institute, NIH, Bethesda, MD; ^2^NantKwest LLC, Culver City, CA; ^3^NantOmics, LLC, Culver City, CA; ^4^NantWorks, LLC, Culver City, CA

####### **Correspondence:** Jeffrey Schlom (js141c@nih.gov)

**Background**

The PD-1/PD-L1 checkpoint axis is a negative regulator of CD8+ T and NK cell function. Immunotherapy targeting this axis has failed to provide significant and durable clinical benefit in the majority of patients with solid carcinomas. In a recent clinical study, patients with NSCLC refractory to PD-1 blockade showed signs of clinical responses to the PD-1 targeting antibody nivolumab when given in combination with the IL-15 superagonist N-IL15 (previous known as ALT803). N-IL15 has been shown to promote significant expansion of circulating NK and CD8+ T cells in cancer patients and has displayed anti-tumor efficacy in murine models of solid carcinomas. Current studies are investigating the combination of N-IL15 with other immunotherapies, especially those that decrease immune suppression in the tumor microenvironment (TME). Thus, it may be possible to improve anti- tumor efficacy through the generation of a molecule that targets IL-15 activity to the TME and potentially reduces immunosuppression and immune-related adverse events.

**Methods**

Using multiple murine models of human solid carcinomas, we examined the immunomodulatory effects and anti-tumor efficacy of N-IL15/PDL1, a novel bifunctional immune-oncology agent comprising N-IL15 fused to two single chain anti-PD-L1 variable region domains.

**Results**

Similar to N-IL15, N-IL15/PDL1 expanded splenic NK and CD8+ T cells. N-IL15/PDL1 promoted a 3.5- and 3.8-fold increase in NK and CD8+ T cell numbers, respectively, as compared to PBS treatment. Both NK and CD8+ T cell populations displayed a more active phenotype, characterized by significant upregulation of numerous activation- and effector function-associated proteins including NKG2D, Ki67, and Granzyme B. Additionally, N- IL15/PDL1 was able to partially block PD-L1 expression in the spleen and tumor. Importantly, N-IL15/PDL1 promoted significant reduction in the growth of MC38-CEA+ colon carcinoma tumors, with 70% of mice undergoing complete tumor rejection. In addition, N-IL15/PDL1 was able to drastically reduce spontaneous metastasis of 4T1 triple-negative breast tumors to the lung by 82% as compared to PBS treatment. In both murine carcinoma models, N-IL15/PDL1 was well-tolerated and displayed comparable or improved anti-tumor efficacy versus previous studies with N-IL15 or PD-L1 monotherapy or N-IL15 plus PD-L1 combination therapy.

**Conclusions**

Together, these findings offer a preclinical proof of concept for the clinical use of the novel bifunctional immune-oncology agent N-IL15/PDL1 to promote tumor control given either as a monotherapy or in combination with additional immunotherapies or standards of care.

#### P661 Quantitative cell-based reporter gene bioassays to advance individual or combination cancer immunotherapy

##### Jamison Grailer, PhD, Jun Wang, Julia Gilden, PhD, Pete Stecha, Denise Garvin, Michael Beck, Jim Hartnett, Frank Fan, PhD, Mei Cong, PhD, Zhi-jie Cheng, PhD

###### Promega, Madison, WI, USA

####### **Correspondence:** Zhi-jie Cheng (jey.cheng@promega.com)

**Background**

Targeting immune checkpoint receptors (e.g., PD-1, CTLA-4) has emerged as a promising new approach to enhance anti-tumor immune responses. While cancer immunotherapies directed against PD-1 and CTLA-4 are showing unprecedented efficacy, some patients and tumor types remain refractory to these therapies. This has resulted in a broadening of immunotherapy research and development to include additional inhibitory (e.g., LAG-3, TIGIT) or stimulatory (e.g., 4-1BB, ICOS) co-receptors targeted individually or in combination with other immune co- receptors.A major challenge in the development of biologics is access to quantitative and reproducible functional bioassays. Existing methods rely on primary cells and measurement of complex functional endpoints. These assays are cumbersome, highly variable, and fail to yield the data quality required for drug development. To address this need, we have developed a suite of cell-based reporter gene bioassays for drug candidates of immune checkpoint targets (PD-1 CTLA-4, LAG-3, TIGIT, BLTA), drug candidates of immune co-stimulatory targets (ICOS, 4-1BB, OX40, GITR, CD27, HVEM (LIGHT), CD40), as well as bispecific antibody drugs targeting two immune checkpoint receptors simultaneously (PD-1+LAG-3, PD-1+TIGIT, PD-1+CTLA-4, PD-1+4-1BB).

**Methods**

These reporter-based bioassays were rationally designed to reflect the mechanism of action (MOA) of drug candidates targeting immune receptors. For each assay, an engineered cell line in an immune cell background was generated which stably expresses an immune target receptor and a luciferase reporter driven by a promoter responding to TCR signaling or activation of a specific immune receptor. These cell lines were further developed into a Thaw-and-Use format that can be used in assays without the need for cell culture.

**Results**

These cell-based bioassays reflect the MOA of each drug target and demonstrate specificity for research-grade antibodies as well as FDA-approved drugs (e.g., nivolumab, ipilimumab). The assay signals are robust and highly reproducible. The assays have been pre-qualified according to ICH guidelines and demonstrate the performance required for use in antibody screening, QC lot release and stability studies.

**Conclusions**

We have developed a suite of MOA-based bioassays for immune checkpoint and co-stimulatory T cell receptors. These assays are easy to use, demonstrate high assay specificity, sensitivity and reproducibility, and are suitable for drug development in a quality-controlled environment.

#### P662 A phase 1a/b study investigating novel anti-PD-L1 antibody (LY3300054): interim safety and clinical activity in patients with advanced cancers

##### Antoine Hollebecque, MD^2^, Hyun Cheol Chung, MD, PhD^3^, Marcus Butler, MD^4^, Antoine Italiano, MD^5^, Chia-Chi Lin, MD, PhD^6^, Jean-Pascal Machiels, MD, PhD^7^, Wu-Chou Su, MD^8^, Marc Peeters, MD, PhD^9^, Leijun Hu, PhD^1^, Anna Szpurka, PhD^1^, Danni Yu, PhD^1^, Anindya Chatterjee, PhD^1^, Burkhard Vangerow, MD^10^, Shivani Nanda, MS^1^, Yumin Zhao, PhD^1^, Mythili Koneru, MD^1^, Yung-Jue Bang, MD PhD^11^, Antoine Hollebecque, MD^2^

###### ^1^Eli Lilly and Company, Indianapolis, IN, USA; ^2^Institut de Cancerologie Gustave Roussy, Villejuif, France; ^3^Yonsei University College of Medicine, Seoul, Korea, Republic of; ^4^UHN/Princess Margaret Cancer Centre, Toronto, ON, Canada; ^5^Institut Bergonie, Bordeaux, France; ^6^National Taiwan University Hospital, Taipei, Taiwan, Province of China; ^7^Cliniques Universitaires Sain-Luc, Brussels, Belgium; ^8^National Cheng Kung University, Tainan, Taiwan, Province of China; ^9^Antwerp University, Antwerp, Belgium; ^10^Lilly Deutschland GmbH, Bad Homburg, Germany; ^11^Seoul National University Hospital, Seoul, Korea

####### **Correspondence:** Antoine Hollebecque (antoine.hollebecque@gustaveroussy.fr)

**Background**

LY3300054 is a novel human monoclonal antibody (IgG1, lambda, Fc-null) targeting PD-L1 expressed on tumor cells and tumor-infiltrating immune cells, preventing binding to its T-cell receptors (PD-1 and CD-80). Here we report phase 1b safety and preliminary efficacy results from LY3300054 monotherapy in patients (pts) with MSI- high (MSI-H) solid tumors and metastatic melanoma (M).

**Methods**

This ongoing, phase 1a/b, multicenter, dose escalation and expansion study enrolled patients with histologically or cytologically confirmed advanced cancer having ECOG PS 0-1, measurable disease (assessable by RECIST v1.1), and no prior treatment with PD-1/PD-L1 agents. The primary objectives for this study were to assess safety and tolerability, secondary objectives included efficacy and pharmacokinetics, and exploratory objectives were biomarker analysis and overall survival. All patients received intravenous infusions of LY3300054 Q2W at 700 mg. Adverse events (AEs) were assessed per NCI CTCAE v4.0; tumor assessment per RECIST v1.1. Recruitment in the M cohort was closed early as eligible melanoma patients were not able to be identified.

**Results**

As of 8 December 2017, 30 patients (MSI-H: n=22, M: n=8) were treated. There were no deaths due to adverse events. Two patients in MSI-H cohort experienced grade 3 treatment-related AEs (TRAEs): diarrhea (n=1, 4.5%), blood creatinine phosphokinase increased (n=1, 4.5%), and hyponatraemia (n=1, 4.5%). No grade 3 events were reported in M cohort, and no grade 4/5 TRAEs were reported in either cohorts. There were no TRAEs leading to discontinuation of study treatment. Preliminary efficacy data in MSI-H cohort showed ORR of 36% [CR in 1 pt (5%) (ovarian), PR in 7 pts (32%) (small intestine adenocarcinoma [1 pt], endometrial [3 pts], colon [3 pts])], DCR in 64% [SD in 6 pts (27%)]; mPFS was 7.39 months (95% CI 1.7, NR). In the M cohort, DCR was 63% [PR in 1 pt (13%), SD in 4 pts (50%)]. As of data cut-off, 16 pts (53%) remain on treatment. Preliminary biomarker analysis, including but not limited to, PD-L1 and CD8 expression and circulating markers will be presented.

**Conclusions**

LY3300054 was well-tolerated and demonstrated antitumor activity in patients with MSI-H solid tumors; combination expansions are ongoing.

**Trial Registration**

NCT02791334

**Ethics Approval**

The study included multiple investigator sites, and some of these had approval dates instead of approval numbers.

The following are listed showing approving committee followed by approval date or approval # (if available): IntegReview, 15Jun2016; IntegReview, 21June2016; MD Anderson Office of Protocol Approval IRB, 26May2016; Princess Margaret Cancer Centre, University Health Network Research Ethics Board, 16-5824 (initial approval date 01Feb2017); Comite de protection des Personnes (CPP), EudraCT number 2016-000440-33

#### P663 Successful monitoring of checkpoint inhibitors by flow cytometry

##### Laila-Aicha Hanafi, Valerie Hebert, Delphine Labit, Vicky Sgouroudis, PhD, Jean-Francpos Poulin, PhD, MBA, Philippe Pouliot, PhD

###### Caprion Bioscienes, Montreal, Canada

####### **Correspondence:** Philippe Pouliot (ppouliot@caprion.com)

**Background**

Cancer immunotherapies represent more than half of treatments approved for oncology, as well as those currently in development. Among the key targets of these therapies are immune-checkpoint molecules (i.e. PD-1, LAG3, PD-L1, CTLA-4). Novel immunotherapies targeting these molecules are designed to impact tumor microenvironment immune-suppression. The characterization and monitoring of circulating and tumor infiltrating immune cells by flow cytometry is a critical aspect of drug development. In order to generated robust data to support drug development, flow cytometric methods must be fully optimized. This poster will discuss the critical aspects of assay optimization for flow cytometric methods with an emphasis on screening monoclonal antibody clones for PD-1, PD- L1, and LAG-3. The importance of the data is illustrated in the differences in assay performance when the correct clones are identified.

**Methods**

Four different anti-PD-1 obtained from different providers were screened on activated PBMC. Additionally, four PD-L1 and five LAG-3 monoclonal antibody clones were screened on activated or rested PBMC spiked in whole blood. All antibodies were all titrated to obtain saturating concentration and optimal titers were compared.

**Results**

On CD4+ T cells from thawed cryopreserved peripheral blood mononuclear cells (PBMC), the best performance was observed with EH12.2H7 or EH12.1 clones which identified 30-33% of CD4+ T cells as PD-1 positive and up to 55-60% of CD8+ T cells as PD-1 positive. Clone eBioJ105 staining of the same samples on the same day identified 14% PD-1 positive in CD4+ T cell and around 40% PD-1 positive in CD8+ T cell, while clone MIH4 staining revealed low level of PD-1 expression on CD4+ T cells and CD8+ T cells. After ex vivo stimulation with staphylococcus enterotoxin B (SEB), EH12.2H7, EH12.1 and eBioJ105 clones all yielded similar results with 76- 85% PD-1 positive on both CD4+ T cells and CD8+ T cells but fewer positive cells were identified with MIH4 clone. Out of the four PD-L1 antibodies tested, only two were able to distinguish a clear PD-L1 population on PBMC rested for 4 days and spiked in whole blood used as positive control for PD-L1 induction. For LAG-3 antibodies, all five were able to reveal the induction of this checkpoint molecule on cells activated by SEB and spiked in whole blood with % of LAG3+ cells in lymphocytes ranging from 13% to 25%.

**Conclusions**

These results illustrate the importance of thorough reagent evaluation in order to achieve a sensitivity coherent with the information-rich promises of flow cytometry.

#### P664 Simultaneous checkpoint - checkpoint or checkpoint - costimulatory receptor targeting with bispecific antibodies promotes enhanced human T cell activation

##### Michael Hedvat, PhD, Gregory Moore, PhD, Matthew Bernett, PhD, Christine Bonzon, PhD, Kendra Avery, PhD, Rumana Rashid, PhD, Alex Nisthal, PhD, Umesh Muchhal, PhD, John Desjarlais PhD

###### Xencor, Inc., Monrovia, CA, USA

####### **Correspondence:** John Desjarlais (jrd@xencor.com)

**Background**

Combination checkpoint blockade promotes productive anti-tumor clinical responses that are often associated with an increase in immune-related adverse events. Because tumor infiltrating lymphocytes (TILs) typically express multiple immune checkpoints and costimulatory receptors [1, 2, 3], we hypothesized that bispecific antibodies will enable targeting of tumor-reactive TILs, leading to safer and more cost-effective combination checkpoint blockade.

**Methods**

We developed optimized bispecific antibody candidates that simultaneously engage PD1 and CTLA4 (XmAb20717), CTLA4 and LAG3 (XmAb22841), and PD1 and ICOS (XmAb23104). These antibodies contain substitutions in the Fc domain to eliminate effector function. These candidate bispecific antibodies preferentially bind cells co-expressing the targeted receptors using transfected cell lines. The in vitro activity of these antibodies were evaluated by measuring cytokine release from Staphylococcal Enterotoxin B (SEB) stimulated PBMC or mixed lymphocyte reactions (MLR). The in vivo activity of the candidate antibodies were evaluated using a model in which human PBMC are engrafted into NSG mice (huPBMC-NSG). Anti-tumor activity was assessed in huPBMC-NSG mice engrafted with established human cancer cell lines.

**Results**

XmAb20717 selectively binds cells expressing both PD1 and CTLA4. Treatment with XmAb20717 enhanced engraftment of hCD45+ cells and the release of IFNγ in NSG mice to levels equivalent to a combination of anti-CTLA4 and anti-PD1 antibodies. XmAb20717 enhanced allogeneic anti-tumor activity of huPBMCs against KG1a cancer cells in vivo. XmAb22841 selectively binds cells expressing both CTLA4 and LAG3. XmAb22841 combined productively with an anti-PD1 antibody enhancing the engraftment of hCD45+ cells and the release of IFNγ in NSG mice via triple-checkpoint blockade. XmAb22841 combined productively with an anti-PD1 antibody, enhancing allogeneic anti-tumor activity of huPBMCs against established MCF7 solid tumors.A bispecific antibody pairing PD1 blockade with engagement of the T cell co-stimulatory receptor ICOS resulted in superior IL-2 release from SEB-stimulated PBMC, warranting preclinical development of XmAb23104. XmAb23104 promoted significant IFNγ release in vitro from a MLR in contrast to a combination of anti-PD1 and anti-ICOS antibodies that lacked activity, illustrating the unique biological properties of XmAb23104. XmAb23104 enhanced engraftment of hCD45+ cells and IFNγ release in NSG mice to levels higher than anti-PD1 antibody alone. XmAb23104 enhanced allogeneic anti-tumor activity of huPBMCs against established MCF-7 tumors in vivo.

**Conclusions**

Compelling ex vivo and in vivo data support the development of XmAb20717, XmAb23104 and XmAb22841 for the treatment of human malignancies. XmAb20717 is currently in a phase 1 study, and IND filings for XmAb23104 and XmAb22841 are anticipated in 2018.

**References**

1. Fourcade J, Sun Z, Pagliano O, et al. CD8(+) T cells specific for tumor antigens can be rendered dysfunctional by the tumor microenvironment through upregulation of the inhibitory receptors BTLA and PD-1. Cancer Res. 2012;72(4):887-96
2. Gross A, Robbins PF, Yao X, et al. PD-1 identifies the patient-specific CD8+ tumor-reactive repertoire infiltrating human tumors. J Clin Invest. 2014;124(5):2246-2259.
3. Matsuzaki J, Gnjatic S, Mhawech-Fauceglia P. Tumor-infiltrating NY-ESO-1–specific CD8+ T cells are negatively regulated by LAG-3 and PD-1 in human ovarian cancer. PNAS. 2010;107(17):7875-7880

#### P665 Anti-tumor synergy evaluation of an AZD4635/ anti-PD-L1 combination therapy using a quantitative systems model

##### Veronika Voronova, MSc^2^, Lulu Chu, PhD^1^, Yuri Kosinsky, PhD^2^, Alexandra Borodovsky, PhD^1^, Richard Woessner, PhD^1^, Kris Sachsenmeier, PhD^1^, Ganesh Mugundu, PhD^1^, Melinda Merchant, MD, PhD^1^, Wenlin Shao, PhD^1^, Kirill Peskov, PhD^2^, Gabriel Helmlinger, PhD^1^

###### ^1^AstraZeneca, Waltham, MA, USA; ^2^M&S-Decisions LLC, Moscow, Russian Federation

####### **Correspondence:** Gabriel Helmlinger (gabriel.helmlinger@astrazeneca.com)

**Background**

Adenosine accumulation in the tumor microenvironment (TME) has been shown to limit anti-tumor immune responses [1]. We developed a model which provides a mechanistic understanding of interactions between the A2AR inhibitor AZD4635 and an anti-PD-L1 antibody (Ab), as observed in four studies in CT26, MC38 and MCA205 syngeneic mice. The model also helped identify factors underlying inter-individual and inter-study variabilities (IIV and ISV), through non-linear mixed effects (NLME) modeling.

**Methods**

A system of differential equations was used to describe antigen presenting cell (APC)-dependent T cell influx into the tumor, T cell proliferation and activation into cytotoxic lymphocytes (CTL), tumor cell death, as well as corresponding PD-L1 expression and related immune response modulation. APC and CTL activation are both regulated by adenosine-dependent A2AR occupancy. Mechanisms of AZD4635 and anti PD-L1 Ab were characterized in the model: while AZD4635 prevents adenosine binding to A2AR, diminishing its immunosuppressive effects, anti-PD-L1 Ab blocks the negative PD-L1 effects on CTL (Fig 1A). Model parameters were taken from literature and estimated using NLME modeling of individual longitudinal tumor size data pooled from the studies. The following treatment regimens were used for calibration: (1) vehicle; (2) AZD4635 10-50 mg/kg BID; (3) anti-PD-L1 Ab 10 mg/kg BIW; (4) combination of (2) and (3). Treatments were initiated when tumors reached a volume of 50-90mm2.

**Results**

The model adequately described individual and population tumor size patterns, for all treatment regimens and studies. IIV and ISV were explained by differences in T cell infiltration and adenosine effect on antigen presentation efficiency. Model simulations showed that AZD4635 increased T cell influx and anti-tumor cytotoxic ability in a dose-dependent manner, by stimulation of antigen presentation efficacy and a direct effect on T cell activation in the TME. However, monotherapy efficacy with AZD4635 was limited by PD-L1 negative feedback, providing a mechanistic explanation of synergistic effect observed in combination treatment. The model also revealed that animals with stabilized tumor size dynamics (“non-progressors”) are characterized by more efficient antigen presentation, thereby maintaining high CTL infiltration during treatment (Fig 1B).

**Conclusions**

A quantitative model was developed to provide mechanistic insights into potentially synergistic effects between AZD4635 and anti-PD-L1. Model-based simulations showed that inhibition of intra-tumor adenosine effects can lead to an increase in antigen presentation efficiency and CTL infiltration – important considerations for further dose and sequencing studies of combination therapies.

**References**

1. Borodovsky A, et al. Inhibition of A2AR by AZD4635 induces anti-tumor immunity alone and in combination with anti-PD-L1 in preclinical models. AACR annual meeting 2018.

**Fig. 1 (abstract P665).**
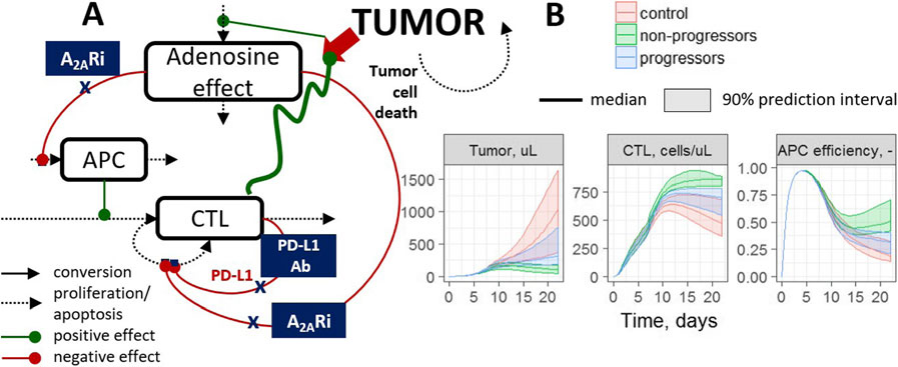
See text for description

#### P666 Antitumor efficacy of anti-TIGIT antagonist antibody EOS884448 is mediated by a dual mechanism of action involving restoration of T cell effector functions and preferential depletion of Tregs

##### Chaterine Hoofd, Julia Cuende, PhD, Virginie Rabolli, PhD, Julie Preillon, MSc, Noemie Wald, PhD, Lucile Garnero, Florence Lambolez, PhD, Marjorie Mercier, Shruthi Prasad, PhD, Florence Nyawame, Sofie Denies, PhD, Margreet Brouwer, MSc, Erica Houthuys, PhD, Veronique Bodo, PhD, Xavier Leroy, PhD, Scott Chappel, Michel Detheux, PhD, Gregory Driessens, PhD, Chaterine Hoofd, Olivier de Henau

###### iTeos Therapeutics, Gosselies, Belgium

####### **Correspondence:** Gregory Driessens (gregory.driessens@iteostherapeutics.com)

**Background**

T cell Immunoreceptor with Ig and ITIM domains (TIGIT) is a co-inhibitory receptor expressed by lymphocytes, preferentially CD8+ T cells, NK, as well as by regulatory T cells (Tregs). TIGIT ligands belong to the PVR/nectin family, among which PVR (CD155) shows the highest affinity and is commonly expressed on myeloid and upregulated on tumor cells. TIGIT costimulatory counterreceptor CD226 (DNAM-1) competes with TIGIT for PVR binding but with a lower affinity. Co-expression of TIGIT and CD226 receptors on T and NK effector cells suggests a role of these molecules in the fine control of cell activation. TIGIT expression is upregulated in cancer patients, especially on Tregs within the tumor microenvironment that are almost all positive for TIGIT and are described to be highly suppressive 1 [1]. Frequent co-expression with exhaustion markers such as PD-1 is an extra argument to develop anti-TIGIT blocking antibodies as a therapeutic approach to reverse T or NK cell dysfunction associated with tumor immune escape.

**Methods**

Antagonist anti-TIGIT antibodies were selected using a synthetic yeast display library of fully human antibodies and characterized for their binding and antitumor properties.

**Results**

EOS884448 anti-TIGIT mAb displays a strong affinity for recombinant and native human TIGIT. It competes with PVR for binding to TIGIT and the blockade of the TIGIT/PVR axis restores cytokine production in human primary T cells. To further explore its potency, EOS884448 was produced in a mammalian system with different human isotypes that exhibit different Fc effector functions. When PBMC from healthy volunteers were incubated with its hIgG1 format, EOS884448 demonstrated preferential depletion of Treg cells in vitro. In vivo, two different isotypes of a surrogate mouse anti-TIGIT mAb were used to evaluate the anti-tumor efficacy in the colon carcinoma CT26 syngeneic murine model. Interestingly, only the ADCC enabling mIgG2a isotype was able to induce strong antitumor efficacy in monotherapy and in combination with an anti-PD1, resulting in a complete regression of pre- established tumors in the majority of animals. Antitumor efficacy was associated with an increased activity of conventional T cells but also with Treg depletion within the tumor microenvironment, reaffirming the in vitro data generated on human PBMCs.

**Conclusions**

In summary, in vitro and in vivo data demonstrate the potential for anti-TIGIT mAb EOS884448 to promote antitumor immunity by preferential depletion of Treg cells and activation of conventional T cells, which supports the rationale for its clinical evaluation.

**References**

1. Joller et al., Treg cells expressing the coinhibitory molecule TIGIT selectively inhibit proinflammatory Th1 and Th17 cell responses. Immunity. 2014 Apr 17;40(4):569-81.

#### P667 EOS100850, an insurmountable and non-brain penetrant A2A receptor antagonist, inhibits adenosine- mediated T cell suppression, demonstrates anti-tumor activity and shows best-in class characteristics

##### Erica Houthuys, PhD, Stefano Crosignani, PhD, Reece Marillier, PhD, Theo Deregnaucourt, MSc, Margreet Brouwer, MSc, Romain Pirson, MSc, Joao Marchante, MSc, Annelise Hermant, MSc, Florence Nyawouame, MSc, Julie Preillon, MSc, Kim Frederix, PhD, Anne-Catherine Michaux, MSc, Jakub Swiercz, PhD, Noemie Wald, PhD, Chiara Martinoli, PhD, Veronique Bodo, PhD, Michel Detheux, PhD, Xavier Leroy, PhD

###### iTeos Therapeutics, Gosselies, Belgium

####### **Correspondence:** Stefano Crosignani (stefano.crosignani@iteostherapeutics.com); Erica Houthuys

**Background**

High levels of extracellular adenosine in the tumor microenvironment promote tumor immune evasion. We and others have shown that adenosine, predominantly through the A2A receptor (A2AR), suppresses the Th1 cytokine production of T cells and monocytes and cytolytic activity of T cells.

**Methods**

Human CD3 T cells were isolated from healthy donor PBMC using Dynabeads. T cells in whole blood or isolated T cells were activated using aCD3/aCD28 stimulation. pCREB assays were performed using freshly collected mouse whole blood.

**Results**

We demonstrated that A2AR antagonists initially designed for Parkinson’s disease but repurposed for immuno-oncology dramatically lost potency in a high adenosine environment. We therefore developed EOS100850, a novel, non-brain penetrant and highly selective inhibitor of A2AR with sub-nanomolar Ki. Using experimental conditions that mimic tumor environment, we have shown that EOS100850 potently inhibited A2AR signalling in human T lymphocytes independently of adenosine concentrations, and rescued cytokine production, even in the presence of high concentrations of A2AR agonists. iTeos A2AR antagonist potently rescued Th1 cytokine production in human whole blood treated by A2AR agonists, and increased CD8+ T cell cytotoxicity in a co-culture assay of effector CD8+ T cells and target cancer cells. An in vivo pharmacodynamic assay based on phosphorylation of CREB (pCREB) in mouse peripheral CD8+ and CD4+ T cells was developed and validated as a readout for A2AR activation. EOS100850, 30 minutes after oral gavage at doses ranging from 0,03 to 1mg/kg demonstrated 80 to 100% inhibition of pCREB induced by the ex vivo addition of A2AR agonist. Remarkably, 12 hours after gavage at 1 and 3mg/kg, when the EOS100850 antagonist was no longer detectable in the plasma, more than 50% of inhibition of pCREB was still observed. These results demonstrate that EOS100850 has a PD activity that extends well beyond its PK based on a long residence time. iTeos’s A2AR antagonist, uniquely designed to address the challenge of counteracting elevated adenosine concentrations in tumors, was tested for the first time in a mouse A20 lymphoma model. iTeos’s A2AR antagonist in combination with anti-PD-L1 demonstrated significant tumor growth suppression compared with anti-PD-L1 alone, with a decrease in tumor volume compared to anti-PD-L1 alone.

**Conclusions**

EOS100850 represents a novel, potent, insurmountable and best-in-class A2AR antagonist, specifically optimized for immuno-oncology indications, that deserves studies in Human.

#### P668 Selection of optimized drug candidates, dosing regimen, pharmacodynamic endpoints, tumor types, and biomarkers for translating inhibition of the adenosine pathway into effective anti-tumor activity

##### Juan Jaen, PhD, Jay Powers, PhD, Ulrike Schindler, PhD, Steve Young, PhD, Matthew Walters, PhD, Joanne Tan, PhD, Lisa Seitz, MSc

###### Arcus Biosciences, Inc., Hayward, CA, USA

####### **Correspondence:** Juan Jaen (jjaen@arcusbio.com)

**Background**

High intra-tumoral adenosine concentrations are prevalent and highly suppressive of the ability to mount an effective anti-tumor immune response. This presents multiple therapeutic opportunities, either by preventing extracellular adenosine generation from nucleotide precursors or by blocking adenosine receptor activation. Translating these therapeutic hypotheses into clinical benefit requires careful selection of tumor types and individual patients most likely to respond to a particular mechanism of action. Equally important is identification of drug candidates with optimal activity profiles and dosing regimens that allow for maximal interference with the selected targets.

**Methods**

The ability of small-molecule adenosine receptor antagonists AB928 – dual A2aR/A2bR – and AB745 (selective A2aR) and CD73 inhibitors (AB680) to inhibit their respective targets was evaluated using recombinant biochemical/cell-based assays, human blood T cells, and immune function assays (e.g., CD3/CD8; MLR). Potency under physiological conditions was assessed using blood or serum-based assays. Blood-based pharmacodynamic assays were used to bridge plasma levels effective in mouse tumor models with those expected to achieve maximal biological effects in humans. Gene expression data from TCGA were used to select tumor types most likely to respond to adenosine receptor or CD73 inhibition. Immunohistochemistry was used to confirm this selection. Various tumor and blood-based biomarkers were developed to assess levels of adenosine-producing enzymes in individual patients receiving one of these drug candidates.

**Results**

AB928: hA2aR KB: 1.5 nM (buffer) & hA2bR KB: 2.0 nM (buffer); hA2aR KD: 1.9 nM (20% serum); reversal of adenosine-mediated inhibition of polyclonal T-cell activation, IC50: 1-3 nM; inhibition of pCREB activation (human whole blood), IC50: 88 nM; healthy volunteer receptor coverage (blood CD8 T cells), 90% inhibition at trough, 150 mg QD. Tumor types selected: NSCLC, CRC, GE, RCC, TNBC, Ovarian.AB680: hCD73 IC50 (CD8 T cells): 0.008 nM; Ki (soluble CD73): 0.005 nM; reversal of AMP-mediated inhibition of T-cell activation in MLR, IC50 ~ 3 nM; inhibition of soluble CD73 (human serum), IC50 ~16 nM; projected human half-life: ≥ 4 days; projected clinical dosing to maintain >90% CD73 inhibition: ≤ 15 mg q2w. Tumor types selected: CRC, NSCLC, GE, SCCHN.

**Conclusions**

The totality of the data for AB928 and AB680 (both of which are in clinical development) indicate that 100-150 mg once-daily oral doses of AB928 and 10-20 mg intravenous AB680 every ~2 weeks should be explored in tumor types that either rely on multiple pathways for adenosine generation (AB928) or those that primarily utilize CD73 for that purpose (AB680).

#### P669 First-in-human Phase 1 study of INCMGA00012 in patients with advanced solid tumors: Interim results of the cohort expansion phase

##### Janice Mehnert, MD^1^, Anthony Joshua, MD^2^, Nehal Lakhani, MD, PhD^3^, Udai Banerji^4^, Drew Rasco^5^, Iwona Lugowska^6^, Monika Tomaszewska-Kiecana^7^, Elena Garralda^8^, Deanna Kornacki, PhD^9^, Bradley Sumrow^10^, Mark Cornfeld, MD, MPH^9^, Chuan Tian^9^, John Powderly, MD, CPI^11^

###### ^1^Rutgers Cancer Institute of New Jersey, New Brunswick, NJ, USA; ^2^Kinghorn Cancer Centre, St Vincents Hospital, Syndey, Australia; ^3^START Midwest – South Texas Accelerated, Grand Rapids, MI, USA; ^4^The Royal Marsden, Sutton, UK; ^5^START – South Texas Accelerated Research, San Antonio, TX, USA; ^6^Maria Sklodowska-Curie Institute – Oncol, Warsaw, Poland; ^7^BioVirtus Research Site, Otwock, Poland; ^8^Vall D’Hebron Institute of Oncology (VHIO), Barcelona, Spain; ^9^Incyte Corporation, Wilmington, DE, USA; ^10^MacroGenics, Inc, Rockville, MD, USA; ^11^Carolina BioOncology Institute, Huntersville, NC, USA

####### **Correspondence:** Janice Mehnert (mehnerja@cinj.rutgers.edu)

**Background**

INCMGA00012 (or MGA012) is a humanized, hinge-stabilized IgG4 monoclonal antibody that binds to PD-1, inhibits the interaction of PD-1 with PD-L1/PD-L2, and disrupts the negative signaling axis to restore T-cell function. At doses up to 10 mg/kg twice weekly (Q2W), INCMGA00012 has an acceptable tolerability profile with no DLTs or MTD. A dose of 3 mg/kg Q2W was selected as the recommended phase 2 dose (RP2D) for the tumor- specific cohorts.

**Methods**

The expansion contained 4 tumor-specific cohorts (endometrial, cervical, soft tissue sarcoma, and non-small cell lung [NSCLC]) and 2 tumor-agnostic flat dose cohorts (500 and 750 mg Q4W). All patients were ≥18 years old; and had disease progression during or following 1–5 prior treatments, measurable disease per RECIST v1.1, and no prior immune checkpoint inhibitors. The primary endpoint was safety. Adverse events were graded via CTCAE v4.03. Response was evaluated by RECIST v1.1 with treatment past progression allowed at the discretion of the investigator.

**Results**

As of July 10, 2018, 127 patients have been treated with INCMGA00012 in the tumor-specific cohorts (29 endometrial, 33 cervical, 32 sarcoma, 33 NSCLC), and 15 each in the tumor-agnostic flat-dose cohorts. Median (range) age was 59 (18–86) years. The majority of patients were female (66.9%), white (85.4%), and had ECOG performance status of 1 (67.5%). Of the 127 patients treated at the RP2D, 59 (46.5%) had treatment-related adverse events (TRAEs), with rash occurring most frequently (n=7 [5.5%]). Eleven patients (8.7%) had immune-related AEs; the only immune-related AE occurring in >1 patient was colitis (n=3). Five patients discontinued treatment due to TRAEs (colitis [n=3], transaminases increased, and iritis [n=1 each]). There were no treatment-related deaths. RECIST responses (confirmed and unconfirmed) were observed in all 4 tumor types (6/25 evaluable patients with cervical cancer, 6/26 with NSCLC, 3/29 with endometrial cancer, and 1/22 with sarcoma). Pharmacokinetics, receptor occupancy, and safety for the 500 mg Q4W flat dose compared favorably to weight-based dosing at 3 mg/kg Q2W.

**Conclusions**

In the cohort expansion portion of this Phase 1 study, INCMGA00012 has been generally well tolerated with evidence of antitumor activity, particularly in the refractory cervical cancer and NSCLC cohorts. Pharmacologic properties with flat dosing are favorable for further development. This study will be further expanded to evaluate the 500-mg Q4W dose in a larger cohort of MSI-h or dMMR endometrial cancer patients as well as a Q3W flat dosing regimen in a tumor-agnostic population.

**Trial Registration**

NCT03059823

**Ethics Approval**

The study was approved by institutional review boards or independent ethics committees of participating institutions.

#### P670 Withdrawn

#### P671 Discovery of a novel anti-LAG3 antagonist antibody

##### Wenqing Jiang, PhD, Lei Fang, Zhengyi Wang, Taylor Guo, Jingwu Zang

###### I-Mab Biopharma, Shanghai, China

####### **Correspondence:** Jingwu Zang (jingwu.zang@i-mabbiopharma.com)

**Background**

LAG3 (CD223) is an inhibitory checkpoint expressed on activated T cells (CD4 and CD8), NK cells and Treg cells. Persistent T-cell activation in a chronic inflammatory environment, such as in a tumor, results in sustained LAG3 expression, contributing to a state of exhaustion manifest in impaired proliferation and cytokine production. In addition, LAG3 is also found to be expressed on Treg cells and play a role in Treg mediated immune suppression. Co-expression of LAG3 and PD-1 on tumor-infiltrating T cells is found in many human cancers including ovarian cancer, melanoma, hepatocellular carcinoma (HCC) and colon cancer. Dual blockade of LAG3 and PD-1 pathway demonstrated significant therapeutic synergy for the treatment of melanoma patients from the latest clinical studies. Here we report the discovery of a novel LAG3 antibody, which showed a strong potency in the T cell activation and tumor growth inhibition in combination with anti-PD-L1 antibodies, indicating a potential therapeutic value of this antibody as a combination partner with PD-1 or PD-L1 agent.

**Methods**

A mouse lead clone, termed mu147H was generated by traditional hybridoma library using human LAG3-extracellular domain as antigen. After humanization and affinity maturation, the candidate antibody (TJA3) was selected based on a series of in vitro assays, including affinity measurement, receptor blocking and T cell activation assay. In vivo efficacy of TJA3 was evaluated in combination with anti-PD-L1 antibody (Tecentriq) using a syngeneic tumor model.

**Results**

TJA3 is an antagonist antibody against LAG3 with sub-nanomolar affinity. Upon binding, it blocks the interaction of LAG3 to its receptor MHCII, leading to the increased production of IL-2 in Jurkat cells overexpressing LAG3 and in activated human primary T cells. Under the same T cell activation system, TJA3 showed strong synergistic effects on the T cell activation in combo with anti-PDL1 antibody. Consistently, when combined with PD-L1 antibody, TJA3 can significantly inhibit tumor growth in a syngeneic MC38 tumor model using LAG3-humanized mice. Developability assessment of the TJA3 sequence indicates that it is a reasonably developable molecule to be taken forward. CMC-wise, a cell line with decent titer have been developed.

**Conclusions**

We have successfully discovered a LAG3-blocking therapeutic antibody with high binding affinity and strong potency in the T cell re-invigoration and anti-tumor efficacy. The observed synergy of our LAG3 antibody with anti- PD-L1 agent indicated a great potential of this asset in the treatment of PD-1/PD-L1 refractory or resistant cancer patients.

#### P672 Discovery and development of a humanized monoclonal antibody targeting the CD73 immune checkpoint for cancer immunotherapy

##### H. Toni Jun, PhD, Fen Pei, PhD, Hui Zou, PhD, Ming Wang, PhD

###### Phanes Therapeutics, Inc., San Diego, CA, USA

####### **Correspondence:** Hui Zou (hzou@phanesthera.com)

**Background**

Extracellular adenosine in the tumor microenvironment can create an immunosuppressive milieu that can act on multiple immune cell types, including effector T cells [1]. CD73 plays a key role in the generation of extracellular adenosine. It is highly overexpressed on multiple tumor types and is expressed on endothelial cells as well as lymphoid and myeloid subsets [reviewed in 2]. CD73 is normally tethered to the cell surface via a GPI linkage but can also be shed from the surface of cells [3] which may result in the presence of a soluble CD73 enzyme in the tumor microenvironment. Combined, soluble and cell expressed CD73 may generate high adenosine levels in the tumor microenvironment. Therefore, inhibition of CD73 should reduce adenosine levels in the tumor environment, thereby relieving immune suppression and preventing tumor evasion from the host immune system. This inhibition, either alone or in combination with immune checkpoint inhibitors such as anti-PD-1 or anti-CTLA4, may consequently be an attractive therapeutic strategy.

**Methods**

Mice were immunized with soluble CD73 to generate a panel of monoclonal antibodies. Antibodies were selected for ability to bind cellular CD73 and inhibit the enzymatic activity of CD73. Select candidates showing desirable activity profiles were humanized prior to further characterization.

**Results**

We have discovered PT199, a humanized antibody that can potently inhibit CD73 catalytic activity in both soluble and cell based enzymatic assays. When compared to other anti-CD73 benchmark molecules, we demonstrate equivalent or improved activity, and we do not observe a “hook” effect common to other anti-CD73 antibodies. This is likely due to the distinct binding epitope of our antibody. Interestingly, PT199 binding to cellular CD73 is affected by the addition of AMPCP, suggesting that the CD73 catalytic site is important to the PT199/CD73 interaction. We are currently characterizing the antibody in a PBMC T-cell suppression assay and the mixed lymphocyte reaction (MLR) assay to confirm activity in cell based functional assays as well as investigating the in vivo activity of PT199 in a mouse A375 xenograft model. These data will be presented.

**Conclusions**

We have generated a potent inhibitory antibody to CD73 that demonstrates activity against cell surface and soluble forms of the enzyme. Based on its expression on tumor and stromal cells as well as its role in immunosuppression, this antibody may represent an important tool as an immunooncology therapy.

**References**

1. Ohta A, Gorelik E, Prasad SJ, Ronchese F, Lukashev D, Wong MK, Huang X, Caldwell S, Liu K, Smith P, et al. 2006. A2A adenosine receptor protects tumors from antitumor T cells. Proc. Natl. Acad. Sci. USA 103: 13132– 13137.
2. Ghiringhelli F, Bruchard M, Chalmin F, Reb´e C. Production of adenosine by ectonucleotidases: a key factor in tumor immunoescape. Journal of Biomedicine and Biotechnology. 2012; 473712-47321.
3. Airas L, Niemelä J, Salmi M, Puurunen T, Smith DJ, Jalkanen S. Differential regulation and function of CD73, a glycosyl-phosphatidylinositol–linked 70-kD adhesion molecule, on lymphocytes and endothelial cells. JCB 1997; 136 (2): 421–431.

#### P673 Preliminary results from an ongoing phase 1 study of AB122, an anti-programmed cell death-1 (PD-1) monoclonal antibody, in patients with advanced solid tumors

##### Paul de Souza, MBBS, MPH, PHD, FRACP^1^, Chee Khoon Lee^2^, Katrin Sjoquist^2^, Shu Pan^2^, Amanda Idan^2^, Aimee Rieger, BS^3^, Wade Berry, BA^3^, Lixia Jin^3^, Lisa Seitz, MSc^3^, Devika Ashok, PhD^3^, Matthew Walters, PhD^3^, Dana Piovesan, MSc^3^, Joanne Tan, PhD^3^, Susan Lee, PhD^3^, Adam Park, BS^3^, Daniel DiRenzo, PhD^3^, Joyson Karakunnel, MD, MSc^3^

###### ^1^University of Western Sydney, Sydney, Australia; ^2^St George Public Hospital, Kogarah, Australia; ^3^Arcus Biosciences, Inc., Hayward, CA, USA

####### **Correspondence:** Joyson Karakunnel (jkarakunnel@gmail.com)

**Background**

AB122, a fully human monoclonal antibody targeting PD-1, has shown preclinically to be similar to currently approved anti-PD-1/programmed cell death ligand 1 agents, but it may offer some unique characteristics. Based on preclinical models, the binding affinity of AB122 for PD-1 appears to be greater than that of nivolumab, which may be due to a difference in epitopes for AB122 versus nivolumab, allowing for tighter target engagement.

**Methods**

We present preliminary data from a phase 1, open-label, dose-escalation (3+3 design) study evaluating AB122 monotherapy in select advanced solid tumors. AB122 is administered intravenously every 2 weeks (Q2W) at escalating doses (80, 240, 720 mg). Intermediate Q2W doses and other schedules are also being evaluated. Treatment continues until progressive disease, unacceptable toxicity, withdrawal of consent, or other reasons for study drug discontinuation occurs. The primary endpoint is safety/tolerability, and secondary endpoints are immunogenicity, pharmacokinetics, pharmacodynamics, and clinical activity.

**Results**

As of 15Jun2018, 15 patients have been treated: 3 at 80 mg, 6 at 240 mg, and 1 at 360 mg Q2W, 2 at 360 mg every 3 weeks, and 3 at 480 mg every 4 weeks. The number of doses received ranged from 1 to 14. The most common adverse events (AEs; regardless of grade or relationship to study drug) were fatigue (40%), diarrhea (27%), nausea (20%), and constipation, insomnia, anemia, and pain (13% each). Five patients (2 each in 80 and 240 mg Q2W cohorts and 1 in 360 mg Q2W cohort) had treatment-related AEs (fatigue in 2 patients, vision blurred and tremor in 1 patient, rash in 1 patient, and rash macular in 1 patient); all were Grade 1. There were no treatment-related grade ≥3 AEs, dose limiting toxicities, serious AEs, or discontinuations due to AEs. Data from patients in the 80 and 240 mg Q2W cohorts showed that AB122 serum concentrations ≥1.5 μg/mL (equivalent to 10 nM) are associated with full receptor occupancy. In 9 of 15 efficacy-evaluable patients, the best overall response was stable disease in 4 patients (44%) and progressive disease in 5 patients (56%). For those with stable disease, tumor sites were colorectal (n=2), ovarian (n=1), and head and neck (n=1), and time on study ranged from 1.9 to 5.8 months. The patients with head and neck and ovarian cancer each experienced a reduction in tumor lesion size.

**Conclusions**

AB122 demonstrated a manageable safety profile and evidence of clinical activity in several solid tumor types.

**Ethics Approval**

The study was approved by Bellberry Human Research Ethics committee (HREC), 129 Glen Osmond Road Eastwood South Australia 5063; Institutional Review Board approval number 2017-10-748.

#### P674 Defining Tim-3 signaling and localization during T cell activation

##### Shunsuke Kataoka, PhD, Lawrence Kane, PhD

###### University of Pittsburgh, Pittsburgh, PA, USA

####### **Correspondence:** Lawrence Kane (lkane@pitt.edu)

**Background**

The transmembrane protein Tim-3 is thought to have inhibitory effects on immune responses and its expression is increased on exhausted T cells during chronic infection or in tumors. Despite containing tyrosine residues, the C- terminal cytoplasmic domain of Tim-3 does not have any known inhibitory motifs. On the other hand, Tim-3 has been shown to have stimulatory effects under acute stimulation conditions. We previously found that the Tim-3 has an ability to enhance TCR signaling, as read-out by phosphorylation of S6 (pS6) and other markers of T cell activation. Other studies suggest that extension of acute T cell activation may contribute to T cell exhaustion. However, it is still unclear how Tim-3 expression enhances TCR signaling and where Tim-3 is localized relative to the immune synapse.

**Methods**

Jurkat T cells transfected with WT or truncated Tim-3, or naïve T cells from CD8-specific Tim-3-induced mice, were pretreated with mitogen-activated protein kinase kinase (MEK) or/and protein kinase B (AKT) inhibitors before being stimulated through the TCR; pS6 was then assessed by flow cytometry. Jurkat cells were also conjugated with superantigen-pulsed antigen presenting cells (APCs) to track the localization of Tim-3 during immunological synapse (IS) formation. These samples were analyzed by ImageStream.

**Results**

As we reported previously, Tim-3 expression enhanced TCR-induced pS6 in both Jurkat and primary cells, and either MEK or AKT inhibitors significantly inhibited pS6 induction. Furthermore, combined treatment with both inhibitors reduced pS6 more than either inhibitor alone and blocked the enhancement of pS6 by Tim-3 expression. We also found that Tim-3 was recruited to the IS by conjugation with superantigen-pulsed APCs from early time points, although the extent of polarization of Tim-3 into the synapse was not as robust as that of CD3. Using various deletion mutants of Tim-3, we observed that neither the extracellular IgV domain nor the intracellular cytoplasmic tail of Tim-3 was required for recruitment to the IS.

**Conclusions**

These results suggest that the MEK/ERK MAPK and PI3K-AKT pathways both play key roles in the enhancement of TCR signaling by Tim-3. In addition, Tim-3 may interact with TCR signaling components by its recruitment proximal to the TCRCD3 complex, although our results suggest that such recruitment is not sufficient to co- stimulate T cells. Nonetheless, this localization may help promote more sustained T cell activation. These findings could aid further the study of Tim-3 effects on immune regulation.

#### P675 Mechanisms of primary resistance to immune checkpoint blockade

##### Duane Moogk, PhD^2^, Michelle Krogsgaard, PhD^2^, Kaitao Li, PhD^3^, Zhou Yuan, PhD^3^, Shi Zhong, PhD^2^, Zhiya Yu^4^, Ivan Liadi^5^, William Rittase, PhD^3^, Victoria Fang, BS^2^, Janna Dougherty, BS^2^, Arianne Perez-Garcia, PhD^2^, Iman Osman^2^, Jeffrey Weber, MD, PhD^2^, Navin Varadarajan, PhD^5^, Nicholas Restifo, MD^4^, Alan Frey, PhD^2^, Cheng Zhu, PhD^3^

###### ^1^New York University School of Medicine, New York, NY, USA; ^2^NYU School of Medicne, New York, NY, USA; ^3^Georgia Institute of Technology, Atlanta, GA, USA; ^4^NCI, NIH, Bethesda, MD; ^5^University of Houston, Houston, TX

####### **Correspondence:** Michelle Krogsgaard (krogsm01@nyumc.org)

**Background**

Although much clinical progress has been made in harnessing the immune system to recognize and target cancer, there is still a significant lack of an understanding of how tumors evade immune recognition and the mechanisms that drive tumor resistance to both T cell and checkpoint blockade immunotherapy. Our objective is to understand how tumor-mediated signaling through inhibitory receptors, including PD-1, combine to affect the process of T cell recognition of tumor antigen and activation signaling. This with the goal of understanding the basis of resistance to PD-1 blockade and potentially identify new molecular targets to enable T cells to overcome dysfunction mediated by multiple inhibitory receptors.

**Methods**

Methods and results are combined.

**Results**

We show that Lck activity affects T cell sensitivity and influences the probability of inducing effector function[1]. Further, we showed that Shp-1 directly influences Lck activity under non-activating conditions, as inhibition of Shp- 1 leads to increased Lck activity. Importantly, inhibition of Shp-1/2, a major mediator of PD-1 signaling, targeting CD28 and Lck[2], prior to activation leads to increased T cell cytotoxic effector function. Our proteomics-based analysis of patient T cells identified additional mediators of PD-1 signaling and signaling components and pathways associated with blockade resistance. It has generally been thought that TCR and CD8 binding depend mainly on their ectodomain interactions with pMHC. We have shown, however, that Lck-CD8 binding[3] and Lck activity[4] are required for upregulated CD8 binding to pre-bound TCR-pMHC complex. Therefore, the cytoplasmic associations of Lck with CD8 and Zap-70, as well as CD3 with Zap-70 may influence formation and stability of the TCR-pMHC-CD8 complex. To determine the mechanistic basis of PD-1 inhibition of TCR-pMHC-CD8 binding we utilized 2D affinity combined with Biomembrane Force Probe (BFP) measurements[5, 6] and showed that PD-1 directly suppresses TCR-pMHC-CD8 binding. Our data also revealed that TCR-pMHC binding was independent of PD-1-PD-L1, but TCR-pMHC-CD8 binding was suppressed by PD-1-PD-L1 binding demonstrating negative cooperativity, as fewer bonds formed than the sum of bonds formed by each interaction alone.

**Conclusions**

Together, our results show that the activities of TCR-proximal signaling components affect T cell mechanosensing and sensitivity at the earliest stages of antigen recognition and are influenced by PD-1. Targeting these interactions may enhance tumor-specific T cell sensitivity and provide an understanding the basis of resistance to PD-1 blockade to potentially allow identification of new molecular targets to enable T cells to overcome dysfunction mediated by multiple inhibitory receptor.

**Acknowledgements**

This work was supported by research grants from National Institute of Health U01 CA214354 (to M.K. and C.Z.) and Merck OTSP grant 58166 (to M.K.) and 57570 (to M.K.).

**References**

1. D. Moogk et al. Constitutive Lck activity drives sensitivity differences between CD8+ memory T cell subsets. J. Immunol.. July 2016; 15; 197(2):644-54.
2. Hui E, et al. T cell costimulatory receptor CD28 is a primary target for PD-1-mediated inhibition. Science. Mar 31 2017; 355(6332):1428-1433.
3. Casas J, et al. Ligand-engaged TCR is triggered by Lck not associated with CD8 coreceptor. Nat Commun. 2014; 5:5624.
4. Jiang N, et al. Two-stage cooperative T cell receptor-peptide major histocompatibility complex-CD8 trimolecular interactions amplify antigen discrimination. Immunity. 2011; 34(1):13-23.
5. Evans E, Ritchie K, Merkel R. Sensitive force technique to probe molecular adhesion and structural linkages at biological interfaces. Biophys J.Jun 1995; 68(6):2580-7.
6. Huang J, et al. The kinetics of two-dimensional TCR and pMHC interactions determine T-cell responsiveness (in eng). Nature. Apr 8 2010; 464(7290):932-6.

**Ethics Approval**

The study was approved NYU School of Medicine Institutions Ethics Board, approval numbers 170302-02 (IACUC) and i10362 (IRB).

#### P676 Targeting the sialoglycan/Siglec pathway in combination with checkpoint inhibitors for cancer immunotherapy

##### Michal Stanczak^1^, Natalia Rodrigues Mantuano^1^, Adam Petrone^2^, Melissa Anne Gray^3^, Carolyn Bertozzi^3^, Li Peng, PhD^2^, Alfred Zippelius, MD^4^, Heinz Läubli, MD PhD^4^

###### ^1^University of Basel, Switzerland, Switzerland; ^2^Palleon Pharmaceuticals, Waltham, MA, USA; ^3^Stanford University, Stanford, USA; ^4^University Hospital Basel, Basel, Switzerland

####### **Correspondence:** Heinz Läubli (heinz.laeubli@unibas.ch)

**Background**

Immunotherapy with immune checkpoint inhibitors (ICI) targeting PD-(L)1 and CTLA-4 has been successfully introduced into routine oncological practice. Despite the success of ICI, most patients derive no benefit from ICI therapy and new approaches are needed including combinations of ICI with new immunotherapeutics. Emerging evidence demonstrated a critical role for the sialoglycan/Siglec axis in cancer immune escape.

**Methods**

In order to further investigate the potential of targeting the sialoglycan/Siglex axis, we used genetic mouse models, tumor cell lines deficient for sialylation and tumor-targeted sialidases. Tumor growth in preclinical mouse models was analysed after targeted desialylation of tumors combined with checkpoint inhibitors including anti-PD-1 and anti-CTLA-4 antibodies.

**Results**

We discovered that hypersialylation on tumor cells and the extracellular tumor matrix results in an immunesuppressive tumor microenvironment through engagement of sialic acid-binding Siglec receptors on immune cells including CD8 T cells. We also found that various Siglecs were upregulated on tumor-infiltrating T cells from cancer patients, and such expression in NSCLC patients correlated with a reduced survival. Siglec- expressing T cells co-expressed several inhibitory receptors including PD-1, suggesting potential for combination therapies by targeting the sialoglycan/Siglec pathway and PD-1 or CTLA-4 inhibition. Here, our work shows that the combination therapy targeting the sialoglycan/Siglec pathway and PD-1 or CTLA-4 inhibition induces tumor control in different preclinical mouse models and increases T cell activation in primary patient tumor samples. Therapeutic intervention of the pathway using EAGLE-301, a Herceptin-sialidase antibody-enzyme conjugate let to targeted desialylation of the tumor microenvironment and resulted in intratumoral T cell activation and T cell- dependent tumor rejection, which was non-redundant to PD-1 or CTLA-4 inhibition. The efficacy was dependent on inhibitory Siglecs. Accordingly, ICI therapies in Siglec-deficient animals were more effective and priming of T cells Siglec-dependent.

**Conclusions**

We demonstrate that targeting the sialoglycan/Siglec pathway is a new immunotherapeutic strategy and can be combined with PD-(L)1 and/or CTLA-4 inhibition for further clinical development.

**Ethics Approval**

Mouse experiments were approved by the local committee of Basel Stadt, Switzerland (approval number 2747).

#### P677 The antitumor efficacy of TIM-3 blockade in a murine model of sarcoma

##### Kristen McEachern, PhD, Geeta Sharma, Srimoyee Ghosh, PhD, Sridhar Ramaswamy, MD, David Jenkins, PhD

###### TESARO, Inc., Waltham, MA, USA

####### **Correspondence:** Kristen McEachern (kmceachern@tesarobio.com)

**Background**

TSR-022 is an anti-TIM-3 antibody that is currently undergoing clinical development in combination with anti-PD-1 (NCT02817633). TIM-3 is an immune checkpoint receptor that negatively regulates T-cell activity and is implicated in resistance to PD-1 blockade. In addition, TIM-3 is also expressed on myeloid cells and has been shown to regulate dendritic cell activity. We previously reported a case study of a patient with leiomyosarcoma who had a partial response to TSR-022 monotherapy. Here we explore the mechanism of the antitumor effect of TIM-3 blockade in a preclinical mouse model of fibrosarcoma.

**Methods**

A/JCr mice were inoculated with murine fibrosarcoma SaL/N cells. Mice were randomized into 4 groups of 8 mice each at tumor volumes of 80-120 mm^3^ and treated with isotype control, anti-mouse PD-1, anti-mouse TIM-3, or a combination of both. A total of 5 doses were administered in a twice-weekly schedule. Mice with complete tumor regression were re-challenged with SaL/N cells along with a fresh cohort of 5 mice to serve as control group. The mice were monitored for tumor regrowth until all the mice in the control group were euthanized due to tumor burden. In a follow-up study to understand the mechanism of efficacy, the mice were randomized at tumor volumes of 200-300 mm^3^, treatment initiated, and tumors collected for immunoprofiling using flow cytometry.

**Results**

Both anti-PD-1 and anti-TIM-3 treatment resulted in antitumor efficacy with 73% and 53% tumor growth inhibition, respectively, which improved to 98% in the combination group. In addition, 2 mice in the anti-PD-1 group and 6 in the combination group showed complete tumor regression. The mice with complete regression were re-challenged and monitored for tumor growth. The tumors in the fresh cohort of mice grew normally; however, no tumor growth was observed in the re-challenged mice, consistent with the induction of immune memory. Tumor immune contexture correlated with the antitumor efficacy seen.

**Conclusions**

In the preclinical setting, TIM-3 blockade alone and in combination with PD-1 resulted in a robust antitumor response associated with durable immunologic memory, further supporting the development of anti-TIM-3 agents, such as TSR-022, in the clinic.

#### P678 Targeting Siglec-15 with NC318, a novel therapeutic antibody to enhance anti-tumor immunity

##### Linda Liu, PhD^1^, Jun Wang, PhD^2^, Jingwei Sun, PhD^2^, Dallas Flies, PhD^1^, Chang Song, PhD^1^, Melissa Zarr, PhD^1^, Kristina Archer, PhD^1^, Alison McGuire, BS^1^, Tom O'Neill, MS^1^, Karla Maloveste, MS^1^, Xinxin Nie, PhD^2^, Agedi Boto, MD/PhD^2^, Ron Copeland, PhD^1^, Sathya Janardhanan, MS^1^, Tete Obot, BS^1^, Jim Bingham, PhD^1^, Kevin Heller, MD^1^, Sol Langermann, PhD^1^, Lieping Chen, MD, PhD^2^

###### ^1^NextCure, Inc., Beltsville, MD, USA; ^2^Yale School of Medicine, New Haven, CT, USA

####### **Correspondence:** Lieping Chen (lieping.chen@yale.edu)

**Background**

Siglec-15 (S15), a member of sialic acid-binding immunoglobulin-type lectins, is a highly conserved Type I cell surface protein, which was previously reported to play a role in osteoclast differentiation and bone remodeling [1,2]. Here we describe S15 as a novel co-inhibitory ligand expressed on tumors and myeloid cells that suppresses T cell function and promotes cancer growth. Blocking S15 by antibody enhances anti-tumor immunity in preclinical models.

**Methods**

S15 KO mice were generated and challenged with syngeneic tumors to study its role in tumor immunology. S15 specific hybridomas were generated by immunizing the KO mice with human S15 fusion protein. Recombinant S15 antibodies that cross react with murine S15 were generated and assessed for their functionality in vitro and in vivo. Top candidate was humanized and characterized.

**Results**

Mice with S15 KO or a conditional KO in myeloid cells have decreased tumor growth and enhanced T cell responses. Additional studies revealed that S15 expression was limited in normal tissues but expressed in tumor cells and tumor-associated myeloid cells. S15 was inducible on myeloid cells by M-CSF and downregulated by IFN-γ, demonstrating that S15 expression was associated with suppressive macrophages. Anti-S15 clone 5G12, bound to S15 with sub-nanomolar affinity, cross reacted with murine and cynomolgus monkey S15, and demonstrated an ability to block S15 Fc fusion protein mediated suppression of human and mouse T cells in vitro. This immunomodulatory property was further demonstrated in several in vivo tumor models, where 5G12 showed monotherapy activity. An increase of tumor antigen-specific effector CD8+ T cell and reduced myeloid cell infiltration were demonstrated within the tumors of 5G12 treated mice. 5G12 also demonstrated anti-tumor synergy with PD-1 blockade. Based on these data, clone 5G12 was humanized and the selected variant, which maintained high affinity and activity, was named NC318. In follow up experiments, NC318 increased IL-2 production in human PBMC assays in a dose dependent manner.

**Conclusions**

S15 immunosuppressive properties in the TME make it a rational target for immunotherapy. NC318 is a high affinity humanized IgG1 mAb specific for S15 developed to reverse tumor immune suppression and promote an effective anti-tumor immune response. NextCure has completed IND-enabling studies and is planning to begin evaluations of NC318 in patients with advanced malignancies in Q4/2018.

**References**

1. Macauley MS, Crocker PR, Paulson JC. Siglec-mediated regulation of immune cell function in disease. Nat Rev Immunol. 2014 Oct;14(10):653-66.
2. Hiruma Y, Tsuda E, Maeda N, Okada A, Kabasawa N, Miyamoto M, Hattori H, Fukuda C. Impaired osteoclast differentiation and function and mild osteopetrosis development in Siglec-15-deficient mice Bone. 2013 Mar;53(1):87-93.

#### P679 Neuropilin-1 is a T cell memory checkpoint limiting long-term tumor immunity

##### Chang Liu, PhD^1^, Ashwin Somasundaram, MD^2^, Tullia Bruno, PhD^2^, Creg Workman, PhD^1^, Dario Vagnali^1^

###### ^1^University of Pittsburgh, Pittsburgh, PA, USA; ^2^UPMC Hillman Cancer Center, Pittsburgh, PA, USA

####### **Correspondence:** Dario Vagnali (dvignali@pitt.edu)

**Background**

Memory CD8+ T cells are pivotal for conferring long-term protective immunity against tumors, and the promotion of their generation and survival is key to achieving effective immunotherapy. At the conclusion of a primary response, a long-lived memory T cell pool is generated from a small fraction of effector CD8+ T cells deviated from terminal differentiation, a process which is thought to be corrupted in tumor-bearing patients and mouse models, resulting in diminished long-term tumor immune surveillance [1]. However, the cell-intrinsic mechanisms limiting this process remain unclear. We found that Neuropilin-1 (NRP1), a receptor constitutively expressed by thymically- derived regulatory T cells (Tregs) and crucial for their suppression of anti-tumor immunity [2], is transiently induced in effector CD8+ T cells in mouse tumor models and in patients with advanced head and neck cancer.We are interested in understanding the impact of CD8+ T cell-expressing NRP1 on both the short- and long-term tumor surveillance and the implication in cancer immunotherapy.

**Methods**

The B16F10 tumor growth in the CD8-restrictive Nrp1-deficient mice (Nrp1L/LE8ICre) was assessed upon initial inoculation, as well as re-challenge after surgical removal of the primary tumors. In a competitive setting, we investigated the long-term persistence and recall activity of Nrp1–/– and Nrp1+/+ pMel-T cells (transgenic CD8+ T cells expressing TCR specific for gp100 melanoma antigen) in response to gp100-B16 tumors, after co-transferred into the same host. Transcriptome analysis was performed in these pMel-T cells (Nrp1–/– vs. Nrp1+/+) recovered from both effector and memory phase to gain insight into the downstream pathways that are modulated by NRP1.

**Results**

Surprisingly, CD8+ T cell-restricted Nrp1-deficient mice showed significantly enhanced protection following B16.F10 tumor challenge, despite unchanged primary tumor growth prior to resection. Constitutive NRP1 expression by CD8+ T cells is sufficient to drive resistance to tumor vaccine-induced secondary tumor protection. Moreover, Nrp1–/– gp100-specific pMel-T cells outcompeted their wildtype counterparts to persist longer in vivo.

**Conclusions**

These data revealed NRP1 as a unique “immune checkpoint” limiting the memory maturation of tumor-reactive CD8+ T cells in a cell-intrinsic manner, which is distinct from the mechanism of action of well-known immune checkpoints (PD1, CTLA4, LAG3) that primarily suppress effector CD8+ T cell function. Importantly, blockade of checkpoint inhibitors of T cell memory may be necessary to achieve durable anti-tumor immunity.

**References**

1. Klebanoff CA, Gattinoni L, Restifo NP. CD8+ T-cell memory in tumor immunology and immunotherapy. Immunol Rev. 2006;211:214-24. Epub 2006/07/11. doi: 10.1111/j.0105-2896.2006.00391.x. PubMed PMID: 16824130; PubMed Central PMCID: PMCPMC1501075.
2. Delgoffe GM, Woo SR, Turnis ME, Gravano DM, Guy C, Overacre AE, et al. Stability and function of regulatory T cells is maintained by a neuropilin-1-semaphorin-4a axis. Nature. 2013;501(7466):252-6. Epub 2013/08/06. doi: 10.1038/nature12428. PubMed PMID: 23913274; PubMed Central PMCID: PMCPMC3867145.

#### P680 Withdrawn

#### P681 A translational platform using primary human immune cells in vitro, syngeneic and humanized models in vivo to support and advance immune-oncology drug discovery

##### Shilina Roman, Gary Salmon, Julie Hawkins, Jonathan White, PhD, Julia Lloyd, Ria Goodwin, Arunima Ganguli, PhD, Omar Aziz, Julia Schueler, Martin O'Rourke, Ian Waddell, Edgar Wood, PhD

###### Charles River, Cambridge, UK

####### **Correspondence:** Ian Waddell (Ian.Waddell@crl.com)

**Background**

Charles River Laboratories (CRL) are establishing a powerful translational immuno-oncology platform with the capability of progressing biologics or small molecule modulators of immune response from in vitro to in vivo assays using human and mouse variants of current check-point inhibitors and small molecules.The platform is supported by an internal blood donor panel which ensures highly reproducible data and high-quality immune cells which are prepared immediately once sampled.

**Methods**

Our in vitro platform includes primary human immune cell assays which profile T cell activation, cytokine release, T cell mediated cancer cell kill, expansion of T cell populations, T cell invasion and macrophage mediated T cell phagocytosis.The platform is currently being expanded to determine the effect of activated immune cell populations on tumour cell spheroid cultures. We are in the process of developing a range of GFP expressing cell lines which will be used to support co-culture experiments. The platform has been validated with standard of care chemotherapeutics, including anti-CTLA4, anti-PD1 and a selection of small molecule inhibitors of targets known to modulate immune responses including IDO inhibitors. Ex-vivo analysis of activated mouse splenocytes response to check-point inhibitors measured as cytokine release and modulation of immune cell populations, as measured by flow cytometry supports the translation of important compounds from the bench to pre-clinical models. Syngeneic mouse tumour models have frequently been used to profile immune responses in tumours, CRL have optimized and profiled existing check-point inhibitors to support immuno-oncology drug discovery using mouse and rat antibody variants of anti-CTLA4 and anti-PD1. To confirm the translational development of our platform CRL have developed and optimized humanized mouse models using sub-cutaneous implanted patient derived xenografts (PDX) with human engraftment via CD34+ haematopoeitic stem cells in NOG mice which were treated with anti- CTLA4 and anti-PD1. Infiltration of human immune cells and PDL-1 expression was detected by flow cytometry (FC) and immunohistochemistry (IHC) in hematopoietic organs and tumor tissue, supporting the initial in vitro response in primary immune cells.

**Results**

We present a screening platform which will support translation of compounds from in vitro primary immune cell assays, to modulation of mouse immune cell population in spleen and tumours, resulting in efficacy and tumour immune cell activation in humanized mouse models.

**Conclusions**

N/A

#### P682 Treatment-naïve HPV+ head and neck cancers display a T-cell-inflamed phenotype distinct from their HPV- counterparts that has implications for immunotherapy

##### Saman Maleki Vareki, Steven Gameiro, Farhad Ghasemi, John Barrett, James Koropatnick, Anthony Nichols, Joe Mymryk

###### University of Western Ontario, London, Canada

####### **Correspondence:** Saman Maleki Vareki (smvsaman@gmail.com)

**Background**

Head and neck squamous cell carcinomas (HNSCC) are often characterized by aggressive local invasion and overall poor prognosis. HPV status is a strong predictor of positive clinical outcome in HNSCC. Expression of viral antigens by HPV+, but not HPV-, HNSCC allows direct comparison of the immune status (immune cell presence and characteristics) between these two otherwise anatomically-similar tumors. Currently, the FDA has approved two anti-programmed death 1 antibodies for the treatment of HNSCC, regardless of the HPV status of the disease. A detailed comparison of the immunological differences between HPV+ and HPV- HNSCC provides an opportunity to identify immunological determinants that contribute to successful treatment in HNSCC that may be broadly applicable to cancer treatment in general.

**Methods**

Patient data from The Cancer Genome Atlas, including Merged Clinical data and Level 3 RSEM normalized Illumina HiSeq RNA expression data for the HNSCC cohort, was downloaded from the Broad Genome Data Analysis Centers Firehose server. RSEM-normalized expression data was extracted and HPV status was manually curated. Primary patient samples with known HPV status were grouped as HPV+, HPV-, or normal. Patient samples with unknown HPV status were omitted from our calculations. This resulted in 73 HPV+, 442 HPV-, and 43 normal control samples with data available for the HNSCC gene expression analysis. Boxplot comparison of gene expression was performed using GraphPad Prism v7.0.

**Results**

We determined that HPV+ HNSCC tumors exhibit a strong Th1 response, characterized by increased infiltration with dendritic cells, CD4+ and CD8+ T-cells, and increased expression of interferon-γ, but not tumor necrosis factor-α. HPV+ HNSCC also expressed higher levels of multiple T-cell exhaustion markers compared to HPV-HNSCC. This gene expression profile is consistent with a “T-cell-inflamed” phenotype, one that is dominated by T- cell markers and chemokines associated with effector T-cell recruitment. Importantly, higher expression of these T- cell inhibitory genes correlated with markedly improved patient survival in HPV+, but not HPV-, HNSCC.

**Conclusions**

The presence of high expression levels of multiple immune inhibitory genes in HPV+ HNSCC suggests that these patients have an existing antitumor immunity and may exhibit strong beneficial responses to immunotherapy, providing a strong rationale for using ICIs as single or combination therapies in first-line treatment of HPV+ HNSCC. This would save patients from disfiguring surgery, or the toxicities associated with conventional chemotherapy or radiation treatment. Finally, expression of immune checkpoint molecules could serve as a predictive biomarker of patient outcome in HPV+ HNSCC.

#### P683 The CTLA-4 x OX40 bispecific antibody ATOR-1015 induces anti-tumor effects through tumor-directed immune activation

##### Anne Mansson Kvarnhammar, PhD, Niina Veitonmäki, Karin Hagerbrand, PhD, Mia Thagesson, Doreen Werchau, BS, Kristine Smedenfors, Anna Dahlman, Anna Rosen, MSc, Maria Johansson, Ida Åberg, Per Norlen, MD, PhD, Christina Furebring, PhD, Peter Ellmark, PhD

###### Alligator Bioscience, Lund, Sweden

####### **Correspondence:** Anne Mansson Kvarnhammar (amk@alligatorbioscience.com)

**Background**

ATOR-1015 is a human IgG1 CTLA-4 x OX40 bispecific antibody, designed as a next generation CTLA-4 antibody with improved benefit-risk profile. Dual targeting of CTLA-4 and OX40, both overexpressed on tumor-infiltrating regulatory T cells (Tregs), directs the effect to the tumor area. This allows ATOR-1015 to induce enhanced anti-tumor effects with lower systemic toxicity compared to CTLA-4 monotherapy. Mode of action is a combination of effector T-cell (Teff) activation and Treg depletion.

**Methods**

Human cells were isolated from leukocyte concentrates from healthy donors. T-cell activation induced by ATOR-1015 upon CTLA-4 or Fcy receptor crosslinking was measured as IL-2 and IFN-y release. Treg depletion was studied using FcyRIIa (H131) and FcyRIIIa (V158) reporter assays and ADCC assays with NK cells measuring LDH release. The effect of ATOR-1015 on Treg function was studied in terms of suppressive activity and IL-10 release.Human OX40 transgenic (knock-in) mice (hOX40KI) with syngeneic tumors were used, enabling studies of both targets as ATOR-1015 binds to murine CTLA-4. Tumor growth and survival were studied after administration of ATOR-1015, monotargeting antibodies to CTLA-4, OX40 and/or PD-1. Tumors and spleens from treated mice were analyzed for Treg and Teff numbers and activation markers by flow cytometry. Tumor localization was assessed by flow cytometry and near infrared (NIR) live imaging of tumor-bearing mice.

**Results**

ATOR-1015 binds CTLA-4 and OX40 simultaneously, promoting cell-cell interactions for enhanced immune stimulating effects. In vitro, ATOR-1015 induces a superior T-cell activation and depletion of Tregs compared to the combination of monotargeting antibodies to CTLA-4 and OX40. ATOR-1015 slightly inhibits the suppressive effect of Tregs and reduces their IL-10 production.ATOR-1015 administered to hOX40KI mice with colon cancer localizes specifically to the tumor via binding to OX40. Similarly, ATOR-1015 increases the intratumoral CD8+ Teff/Treg ratio by depleting Tregs and increasing the infiltration/expansion and cytotoxic activity of Teffs without affecting systemic T cells.Treatment with ATOR-1015 reduces tumor growth and prolongs survival in several tumor models. Re-exposure of mice that responded to ATOR-1015 treatment with the same and an irrelevant tumor, demonstrates that the response is tumor-specific and induces long-lasting immunological memory. Lastly, ATOR- 1015 improves the anti-tumor responses of anti-PD-1 treatment.

**Conclusions**

ATOR-1015 is a next generation CTLA-4 antibody with tumor-directed activity for enhanced efficacy and reduced toxicity. ATOR-1015 increases the anti-tumor responses of anti-PD-1 treatment in mice, supporting the combination with PD-1/PD-L1 in the clinic. ATOR-1015 is planned to enter clinical phase I in H2 2018.

**Ethics Approval**

The studies were approved by Malmö/Lund Ethics Board approval number M142-15.

#### P684 Preclinical studies of TIM-3 blockade supporting clinical development of BMS-986258, an anti–TIM-3 monoclonal antibody

##### Xiao Min Schebye, PhD^1^, Minhua Han^1^, Hong-An Truong^1^, Andy Deng^1^, Alan Korman, PhD^1^, Mark Selby, PhD^1^, Christine Bee^2^

###### ^1^Bristol-Myers Squibb, Redwood City, CA, USA; ^2^Bristol Myers Squibb, x, USA

####### **Correspondence:** Xiao Min Schebye (xiaomin.schebye@bms.com)

**Background**

T-cell immunoglobulin- and mucin-domain-containing-3 (TIM-3) is among the next generation of checkpoint inhibitors whose role in human cancer therapy is being explored. TIM-3 is often co-expressed with PD-1 in CD8^+^ T cells and is a marker of CD8^+^ T-cell dysfunction or exhaustion in several cancers [1-3]. TIM-3 is also expressed on natural killer cells, regulatory T cells, and antigen-presenting cells (APCs), where its role in mediating tumor immunity is not well understood. To this end, we have developed a TIM-3 antagonist for evaluation in human trials. Here we describe the preclinical characterization of BMS-986258, a fully human immunoglobulin (Ig)G1 anti–TIM- 3 monoclonal antibody that was engineered to eliminate Fc-gamma receptor binding.

**Methods**

BMS-986258 binding activity was evaluated by fluorescence-activated cell sorting (FACS) and Scatchard analysis. An artificial APC:Th1 co-culture assay, in which CHO cells expressing a single-chain anti-CD3 (CHO-OKT3) were irradiated, was developed to assess BMS-986258 functions. In addition, CHO-OKT3 cells expressing PD-L1 were cultured with Th1 cells to evaluate co-blockade of TIM-3 and PD-1. Single-cell suspension of human tumor- infiltrating leukocytes (TIL) derived from surgically resected tumor tissues of a variety of tumor types was co- cultured with the irradiated CHO-OKT3 cells. Interferon (IFN)-gamma production was evaluated by enzyme-linked immunosorbent assay (ELISA), and the percentage of IFN-gamma^+^CD8^+^ T cells was determined using FACS.

**Results**

BMS-986258 bound with high affinity and selectivity to TIM-3 expressed on activated human T cells (EC_50_: 0.1 nM; K_D_: 0.16 nM). The antibody bound to the phosphatidylserine (PS) binding site of TIM-3 as revealed by a crystal structure of TIM-3 IgV domain complexed with the Fab of BMS-986258 resolved to 1.5Å, consistent with the observation that BMS-986258 blocks TIM-3 from binding to PS in an in vitro TIM-3:PS blocking assay. BMS- 986258 and its monovalent Fab fragments promoted Th1-cell proliferation in a concentration-dependent manner, indicating that BMS-986258 acts as an antagonistic antibody in promoting T-cell function. When using the irradiated CHO-OKT3 cells expressing PD-L1, BMS-986258 and nivolumab (anti–PD-1) promoted Th1-cell proliferation as single agents, and co-blockade of TIM-3 and PD-1 showed an additive effect. In the human TIL assay, BMS-986258 increased both IFN-gamma production and IFN-gamma+CD8+ T cells. The EC_50_ value for a human tumor TIL assay was 1.2 nM.

**Conclusions**

These results support the notion that TIM-3 blockade promotes CD8^+^ T-cell functions in tumors. Preclinical characterization of BMS-986258 supports its evaluation alone and in combination with nivolumab in patients with advanced cancers (NCT03446040).

**References**

1. Fourcade J, Sun Z, Benallaoua M, et al. Upregulation of Tim-3 and PD-1 expression is associated with tumor antigen-specific CD8+ T cell dysfunction in melanoma patients. *J Exp Med*. 2010;207:2175-2186.
2. Gao X, Zhu Y, Li G, et al. TIM-3 expression characterizes regulatory T cells in tumor tissues and is associated with lung cancer progression. *PLoS One*. 2012;7:e30676.
3. Yang ZZ, Grote DM, Ziesmer SC, et al. IL-12 upregulates TIM-3 expression and induces T cell exhaustion in patients with follicular B cell non-Hodgkin lymphoma. *J Clin Invest*. 2012;122:1271-1282.

**Ethics Approval**

This preclinical study was conducted in accordance with ethical principles and local laws/regulations. The use of samples were reviewed and approved by an institutional review board or independent ethics committee.

#### P685 CD226 impact on TIGIT blockade in T-cell rejuvenation

##### Katherine (Kate) MacDonald, PhD, Yoon Park, Padma Perkins, Eric Boyer, Salvatore Santino, Michael Hamilton, Ishita Barman, Pavel Strop, Alan Korman, PhD, Bryan Barnhart, PhD

###### Bristol-Myers Squibb, Redwood City, CA, USA

####### **Correspondence:** Katherine (Kate) MacDonald (Kate.MacDonald@bms.com)

**Background**

T-cell immunoreceptor with Ig and ITIM domains (TIGIT) is a checkpoint molecule that interacts with poliovirus receptor (PVR) and plays a key role in maintaining immune homeostasis. TIGIT counterbalances CD226-mediated T-cell activation by competing with CD226 for binding to PVR, a receptor that is expressed in multiple tumor types. Signaling through TIGIT contributes to T-cell exhaustion, resulting in inhibition of antitumor T-cell responses. Importantly, antibody blockade of TIGIT has shown antitumor activity in preclinical mouse models, highlighting the potential utility of this pathway for tumor immunotherapy. Previous work has demonstrated an interdependence between TIGIT and CD226 for maximal activity of TIGIT blockade; however, the precise mechanism of T-cell rejuvenation by TIGIT blockade remains elusive. Here we present data describing the relationship between TIGIT and CD226 in T-cell activation.

**Methods**

Tumor-infiltrating lymphocytes (TILs) from a variety of human tumors, including non-small cell lung cancer, renal cell carcinoma, and colorectal cancer, were analyzed for coexpression of TIGIT and CD226. *In vitro* functional assays were established to test the impact of TIGIT blockade on antigen-specific T cells from healthy donors, exploring the relationship between CD226 expression and its impact on TIGIT-mediated T-cell suppression. This relationship was further analyzed in various subsets of CD8^+^ T cells, focusing on those with phenotypic characteristics representative of exhausted T cells.

**Results**

CD8^+^ TILs expressed higher levels of TIGIT together with other checkpoint receptors, including PD-1, but lower levels of CD226 than T cells from patient-matched peripheral blood. Stimulation of TILs with anti-CD3 restored CD226 expression in some samples but correlated with an increase in expression of TIGIT and other exhaustion markers. In analyses of healthy donor peripheral blood, CD226^low^ CD8^+^ T cells displayed phenotypic characteristics of exhausted T cells and impaired effector function compared with CD226^high^ CD8^+^ T cells. In contrast to CD226^low^ CD8^+^ T cells, the antigen-specific T-cell response of CD226^high^ CD8^+^ T cells was greatly enhanced by TIGIT blockade alone or combined TIGIT and PD-1 blockade.

**Conclusions**

These findings highlight the importance of the relative expression of CD226 and TIGIT required for T-cell function and suggest that these pathways play a role in governing optimal T-cell activity. Together, these observations suggest that TIGIT and CD226 potentially regulate antitumor T-cell responses and warrant further investigation of these molecules in human cancers.

**Ethics Approval**

This preclinical study was conducted in accordance with ethical principles and local laws/regulations. The use of samples were reviewed and approved by an institutional review board or independent ethics committee.

Tumor-infiltrating lymphocytes (TILs) from a variety of human tumors, including non-small cell lung cancer, renal cell carcinoma, and colorectal cancer, were analyzed for coexpression of TIGIT and CD226. *In vitro* functional assays were established to test the impact of TIGIT blockade on antigen-specific T cells from healthy donors, exploring the relationship between CD226 expression and its impact on TIGIT-mediated T-cell suppression. This relationship was further analyzed in various subsets of CD8^+^ T cells, focusing on those with phenotypic characteristics representative of exhausted T cells.

**Results**

CD8^+^ TILs expressed higher levels of TIGIT together with other checkpoint receptors, including PD-1, but lower levels of CD226 than T cells from patient-matched peripheral blood. Stimulation of TILs with anti-CD3 restored CD226 expression in some samples but correlated with an increase in expression of TIGIT and other exhaustion markers. In analyses of healthy donor peripheral blood, CD226^low^ CD8^+^ T cells displayed phenotypic characteristics of exhausted T cells and impaired effector function compared with CD226^high^ CD8^+^ T cells. In contrast to CD226^low^ CD8^+^ T cells, the antigen-specific T-cell response of CD226^high^ CD8^+^ T cells was greatly enhanced by TIGIT blockade alone or combined TIGIT and PD-1 blockade.

**Conclusions**

These findings highlight the importance of the relative expression of CD226 and TIGIT required for T-cell function and suggest that these pathways play a role in governing optimal T-cell activity. Together, these observations suggest that TIGIT and CD226 potentially regulate antitumor T-cell responses and warrant further investigation of these molecules in human cancers.

**Ethics Approval**

This preclinical study was conducted in accordance with ethical principles and local laws/regulations. The use of samples were reviewed and approved by an institutional review board or independent ethics committee.

#### P686 The number of metastatic compartments involved at immunotherapy initiation may influence survival in stage-IV non-small cell lung cancer

##### Abdul Rafeh Naqash, MD, Mahvish Muzaffar, MD, Paul Walker, MD, Li Yang, PhD

###### East Carolina University, Greenville, NC, USA

####### **Correspondence:** Abdul Rafeh Naqash (Naqasha16@ecu.edu)

**Background**

In recent years immune checkpoint blockade (ICB) has revolutionized the management of non-small cell lung cancer (NSCLC). However, outside of clinical trials, the lack of uniform responses suggests clinical heterogeneity. Often patients with high tumor burden tend to do poorly with ICB. Even in non-bulky disease, due to organ-specific tumor microenvironments (TME), metastasis to >1 organ may suggest distinct tumor biology that may in turn influence outcomes. Hence, we sought to investigate if the number of metastatic compartments involved at ICB initiation could impact survival.

**Methods**

We retrospectively identified 100 stage-IV NSCLC patients treated with ICB from April 2015 to February 2018. Follow up cutoff for survival analysis was set on July 1, 2018. A single metastasis to >1 organ was categorized as >1 metastatic compartment involvement (MCI) which was independent of the number of lesions in the organ or total tumor bulk. Overall survival after immunotherapy (OSI) was defined as the time from ICB initiation to last follow up or death. Cox regression was used to assess survival correlations.

**Results**

The median age was 63 years with predominant histology being adenocarcinoma (65.0 %). A majority of patients were of Caucasian ethnicity (66.0 %) and male gender (59.0 %). Nivolumab (72.0%), Carboplatin/Alimta/Pembrolizumab (17.0%), Pembrolizumab (6.0%), and Atezolizumab (5.0%) were used for ICB. The median number of cycles for ICB was 4.0. Skeletal involvement (45.0 %), brain (35.0 %) and liver (20.0 %) were the most common metastatic compartments. 47.0 % of patients had > 1 MCI at ICB initiation. No differences in baseline medians for C-reactive protein, albumin or neutrophil-lymphocyte ratio were seen based on MCI. The median OSI for our NSCLC cohort was 7.5 months. Brain or liver involvement did not show inferior OSI. >1 MCI at ICB initiation was noted to be independently associated with inferior OSI both in the univariate [p= 0.02, HR 1.68, CI(1.06-2.65)] and multivariate Cox regression [p=0.02, HR 1.85, CI(1.09-3.13)].

**Conclusions**

Involvement of >1 metastatic compartment which may or may not be synonymous with the total tumor bulk, appears to be an important factor in survival stratification of NSCLC treated with ICB. This suggests that patients with >1 MCI may have baseline aggressive tumor biology that adapts to different TME and does not respond well to ICB. Hence improving outcomes in such subgroups will require exploring strategies that involve combining different immunotherapies or other novel agents with ICB to overcome this resistant biology.

**Ethics Approval**

Study was approved by ECU UMCIRB 15-001400

#### P687 Single-cell analysis of human T cells in the bladder tumor microenvironment reveals novel cytotoxic CD4s that are modulated by anti-PD-L1 therapy

##### David Oh, MD, PhD^1^, Serena Kwek^1^, Siddharth Raju, BS^1^, Tony Li, BS^1^, Eric Chow, PhD^1^, Arun Burra, BS^1^, Chien-Chun Pai, PhD^1^, Chiara Rancan, PhD^1^, Yang Sun, PhD^1^, Jacky Li, BS^1^, Dvir Aran, PhD^1^, Matthew Spitzer, PhD^1^, Serghei Mangul, PhD^2^, Sima Porten, MD^1^, Maxwell Meng, MD^1^, Terence Friedlander, MD^1^, Chun Jimmie Ye, PhD^1^, Lawrence Fong, MD^1^

###### ^1^University of California, San Francisco, San Francisco, CA, USA; ^2^University of California, Los Angeles, Los Angeles, CA, USA

####### **Correspondence:** Lawrence Fong (lfong@medicine.ucsf.edu)

**Background**

Bladder cancer can be responsive to immunotherapies such as PD-1 checkpoint inhibitors, but overall response rates are low. While tumor-resident T cells may demonstrate considerable heterogeneity in their antigenic repertoire and functional phenotype, the genotypic and phenotypic features of T cells in the bladder tumor environment and how both are modulated by systemic therapies remains unclear.

**Methods**

We performed droplet single-cell RNA and paired T cell receptor (TCR) sequencing of CD4+ and CD8+ T cells isolated from localized bladder tumors and paired adjacent non-malignant tissue including patients who received anti-PD-L1 antibody prior to surgery.

**Results**

Bladder tumors possess both known and novel CD4+ T cell populations, including multiple populations of regulatory (CD4reg) and central memory (CD4cm) cells, as well as novel populations of cytotoxic (CD4cyto) CD4+ T cells expressing cytolytic effectors and granule-associated proteins. Moreover, CD4cyto cells are functionally competent and capable of killing autologous tumor cell. While CD4reg populations are consistently enriched in tumor compared to adjacent non-malignant tissue, anti-PD-L1 therapy results in enrichment of a specific CD4cyto population in treated tumor compared to treated non-malignant tissue, which is not enriched in untreated bladder tumors. Anti-PD-L1 therapy also elicits a more oligoclonal TCR repertoire in intratumoral CD4cyto1 as well as CD4reg populations compared to non-malignant tissue. Individual CD8+ populations are not detectably enriched in tumor across all samples, and anti-PD-L1 therapy does not specifically enrich CD8+ populations or restrict their repertoire in tumors.

**Conclusions**

These findings reveal the importance of CD4+ T cell heterogeneity in the bladder tumor environment, and underscores that cancer immunotherapies may elicit both quantitative enrichment and focusing of the antigenic repertoire of novel intratumoral cytotoxic CD4+ populations in lieu of effects on cytotoxic CD8+ cells.

**Ethics Approval**

The study was approved by the Institutional Review Board of the University of California, San Francisco (approval # 14-15423).

#### P688 Peripheral blood profiling to identify predictors of sensitivity to anti–PD-1 blockade in non-small cell lung cancer

##### Akio Osa, Student^1^, Yujiro Naito^1^, Takeshi Uenami^2^, Masahide Mori^2^, Atsushi Kumanogoh^1^

###### ^1^Osaka University Graduate School of Medicine, Suita city, Japan; ^2^National Hospital Organization Toneyama National Hospital, Suita City, Japan

####### **Correspondence:** Atsushi Kumanogoh (koyama@imed3.med.osaka-u.ac.jp)

**Background**

Recent clinical trials have demonstrated that a specific subset of patients with non–small cell lung cancer (NSCLC) exhibits a clear response to PD-1 blockade. Although selecting for patients with high PD-L1 expression enriches the response to anti–PD-1 therapy, more than 50% of selected patients do not demonstrate a durable response to PD-1 blockade. Several studies have reported that the neutrophil-to-lymphocyte ratio (NLR) at the initiation of anti–PD-1 blockade is potentially a marker of therapeutic outcomes in cancer patients; however, a definitive predictive biomarker in peripheral blood has not been found. Therefore, we performed immune cell analysis on peripheral blood from patients with NSCLC receiving nivolumab.

**Methods**

This study enrolled 96 patients with NSCLC who began nivolumab as second-line or further-line therapy at Osaka University Hospital and Toneyama Hospital between January 2016 and May 2018. After excluding patients who were driver mutation positive (n=15), discontinued treatment due to immune-related adverse events (n=11), or recently received treatment for another malignancy (n=2), 68 patients were in the analysis. The distribution of immune cell populations in freshly collected blood samples before nivolumab administration were examined using flow cytometry including differentiation and proliferation markers. We also examined plasma humoral factors and serum interferon-inducing activity using THP-1-ISG reporter cells.

**Results**

We divided patients into two groups: the durable response (DR) group, who received 6 or more doses of nivolumab (n=38), and the early progression (EP) group, who received less than 6 doses (n=30). The percentage of neutrophils among all CD45-positive cells was significantly lower in the DR group, whereas the percentages of CD56+ T cells and CD141+ dendritic cells were significantly higher in the DR group. Regarding differentiation and proliferation markers, the percentage of effector memory T cells and Ki-67–positive T cells were significantly higher in the DR group. We will present humoral factor and interferon-inducing activity findings at the presentation.

**Conclusions**

Immune analysis using fresh peripheral whole blood might be useful in identifying NSCLC patients who are nivolumab sensitive or resistant. Profiling results suggest that neutrophil-rich status is possibly linked to suppression of effector T cell count and function. Future studies will focus on the detailed mechanisms in the immunosuppressive role of neutrophils in patients with NSCLC resistant to anti PD-1 therapy.

**Ethics Approval**

The study was approved by Osaka University and Toneyama National Hospital Institutution‘s Ethics Board, approval number 15383 and 1545.

**Consent**

Written informed consent was obtained from the patient for publication of this abstract and any accompanying images. A copy of the written consent is available for review by the Editor of this journal.

#### P689 Withdrawn

#### P690 Pharmacokinetics (PK) and dosing regimen selection of the PD-1 Inhibitor ABBV-181 in patients with advanced solid tumors: Preliminary Phase 1 results from study M15-891

##### Apurvasena Parikh, PhD, Sreeneeranj Kasichayanula, PHD, Rajeev Menon, Daniel Afar, PhD, Stacie Lambert, Benjamin Engelhardt, Sven Mensing

###### AbbVie Inc., Redwood City, CA, USA

####### **Correspondence:** Apurvasena Parikh (apurvasena.parikh@abbvie.com)

**Background**

ABBV-181 is a humanized, recombinant, IgG1 monoclonal antibody targeting programmed cell death 1 (PD-1). ABBV-181 is currently being evaluated in a phase 1 clinical trial (NCT03000257) in patients with solid tumors. Preliminary ABBV-181 pharmacokinetic (PK) data, population PK modeling and PK-pharmacodynamic (PD) simulations supporting a flat dose and alternate regimens are described herein.

**Methods**

Patients with previously treated advanced solid tumors received ABBV-181 at 1, 3, or 10 mg/kg IV Q2W in dose escalation. The ongoing expansion cohorts include patients with multi-histology, non-small cell lung cancer and head and neck squamous cell cancer. Intensive PK samples for ABBV-181 were collected in Cycle 1 and Cycle 3, along with pre-dose concentrations for other Cycles, for all subjects. PD assessments included circulating T-cell PD- 1 receptor saturation by flow cytometry. Serum concentration-time data were summarized using non-compartmental PK analysis (Phoenix WinNonlin 7.0) and modeled using nonlinear mixed effects modeling (NONMEM 7.3) using a two compartment PK model. PK/PK-PD simulations for flat doses and varying regimens were conducted utilizing a distribution of wide range of body-weight values (47-128 kg) to compare exposures and corresponding PD between weight-based, and flat dosing.

**Results**

Preliminary PK data were available for 62 patients (N=24 for 1-10 mg/kg Q2W in dose escalation and N=38 for 250 mg Q2W in dose expansion). ABBV-181 PK were approximately dose-proportional across the dose range studied with Cmax values ranging from 19.5-277 μg/mL (%CV 26-43%), AUCinf values of 214-3129 μg*day/mL (%CV 31-59%), and 2-3 fold accumulation with Q2W dosing in Cycle 3 compared to Cycle 1. Population PK modeling and simulation analyses indicated that a 250 mg fixed dose would result in similar overall exposures achieved with a 3 mg/kg dose, which was consistent with the observed data. PK predictions indicated the exposures achieved with alternate dosing regimens, i.e., 375 mg Q3W and 500 mg Q4W will result in PD-1 positive CD4 T central memory cell saturation and significant PD-1 blockade, with likely no impact on safety based on the available safety data.

**Conclusions**

The 3 mg/kg and 250 mg Q2W doses were safe, well-tolerated and indicated complete receptor saturation at clinical concentrations. Flat alternate dosing regimens of 375 mg Q3W and 500 mg Q4W were predicted to achieve comparable efficacious exposures as the 250 mg Q2W regimen. PK data and simulations support flat dosing and less frequent dosing regimen for ABBV-181 in expansion, with likely no impact on safety events.

**Acknowledgements**

AbbVie and the authors thank the patients participating in this clinical trial and all study investigators for their contributions

**Ethics Approval**

This study was approved by an institutional review board or ethics committee at each participating center.

#### P691 Correlating skin toxicity and steroid treatment with outcomes of Anti-PD-1 therapy

##### Henry Quach, BS^1^, Anna Dewan, MD^2^, Douglas Johnson, MD, MSCI^2^

###### ^1^Vanderbilt University School of Medicine, Nashville, TN, USA; ^2^Vanderbilt University Medical Center, Nashville, TN, USA

####### **Correspondence:** Douglas Johnson (douglas.b.johnson@vumc.org)

**Background**

Immune checkpoint inhibitors (ICI) such as anti-programmed death protein 1 (anti-PD-1) antibodies produce durable responses in a subset of cancer patients. ICI can produce immune-related adverse events (irAEs). Among the earliest and most common irAEs are skin toxicities [1]. Several studies have associated the development of irAEs with increased treatment efficacy, though it remains unclear whether steroid treatment for irAEs interferes with the antitumor effects of ICI [2-4]. We sought to evaluate the effect of cutaneous irAEs on treatment outcomes.

**Methods**

We retrospectively assessed whether skin toxicity with anti-PD-1 correlated with clinical response in patients with metastatic melanoma. Skin toxicity was defined as new rash or pruritus arising on therapy. Subjects at one center that received treatment with anti-PD-1 therapy were included (n = 318). Presence and timing of skin toxicity, and steroid treatment (topical vs. systemic) were correlated with response rate (RR), progression-free survival (PFS), and overall survival (OS).

**Results**

38% of patients on anti-PD-1 therapy (n = 121) developed skin toxicity. Skin toxicity was correlated with higher RR (60.3% vs. 28.4%, p<0.0001), clinical benefit rate (response + stable disease; 73.6% vs. 41.6%, p<0.0001), median PFS (743 days vs. 112 days, p<0.0001), and median OS (1691 days vs. 517 days, p<0.0001). Late skin toxicity (after 3 months on therapy) was associated with superior clinical outcomes than early skin toxicity. Not developing cutaneous toxicity was associated with the worst outcomes both in terms of median PFS (not reached vs. 383 days vs. 112 days, p<0.0001 for late, early, and no skin toxicity, respectively) and OS (median not reached vs. 1065 days vs. 526 days p<0.0001). There was no difference in PFS or OS for patients who received topical steroids, systemic steroids, or no steroids. Interestingly, patients who experienced pruritus without rash had inferior PFS compared with other skin toxicities (p=0.0008), which was comparable to patients without skin toxicity.

**Conclusions**

The development of cutaneous toxicity is correlated with clinical response to anti-PD-1 therapy. Late toxicity is associated most strongly with response, and illustrates that time on therapy can confound toxicity-efficacy correlations. The development of pruritis alone does not correlate with beneficial outcomes, suggesting that distinct mechanisms may be responsible for pruritus. The use of steroids to treat these skin toxicities did not affect treatment outcomes.

**Acknowledgements**

We would like to acknowledge the Vanderbilt Medical Scholars Program and UL1 RR 024975 (NIH CTSA grant).

**References**

1. Postow M, Sidlow R, Hellmann M. Immune-Related Adverse Events Associated with Immune Checkpoint Blockade. N Engl J Med. 2018; 378(2):158-168.
2. Hua C, Boussemart L, Mateus C, Routier E, Boutros C, Cazenave H, Viollet R, Thomas M, Roy S, Benannoune N, Tomasic G, Soria JC, Champiat S, Texier M, Lanoy E, Robert C. Association of Vitiligo With Tumor Response in Patients With Metastatic Melanoma Treated With Pembrolizumab. JAMA Dermatol. 2016; 152(1):45-51.
3. Teulings H, Limpens J, Jansen S, Zwinderman A, Reitsma J, Spuls P, Luiten R. Vitiligo-like depigmentation in patients with stage III-IV melanoma receiving immunotherapy and its association with survival: a systematic review and meta-analysis. J Clin Oncol. 2015; 33(7):773-81.
4. Sanlorenzo M, Vujic I, Daud A, Algazi A, Gubens M, Luna SA, Lin K, Quaglino P, Rappersberger K, Ortiz-Urda S. Pembrolizumab Cutaneous Adverse Events and Their Association With Disease Progression. JAMA Dermatol. 2015; 151(11):1206-1212.

**Ethics Approval**

This study was approved by Vanderbilt University Medical Center’s Ethics Board; approval number 150625.

#### P692 Effect of steroids in metastatic non-small cell lung cancer (mNSCLC) patients treated with nivolumab after initial platinum therapy in community practices in the USA

##### Jenine Sanzari, PhD^1^, Jay Rathi, Marc Monté^2^, David Smith^3^, Mark Danese^4^, Michelle Gleeson^4^, Deborah Lubeck^4^, James White^1^, Pranav Abraham^1^, Beata Korytowsky^1^, Brian Ulrich^5^

###### ^1^Bristol-Myers Squibb, Lawrence, NJ, USA; ^2^St. Bernards Medical Group, Jonesboro, AR, USA; ^3^Compass Oncology, Vancouver, WA, USA; ^4^Outcomes Insights, Inc, Westlake Village, CA, USA; ^5^Texas Oncology, Houston, TX, USA

####### **Correspondence:** Jenine Sanzari (Jenine.Sanzari@bms.com)

**Background**

Limited data are available on mNSCLC patients treated with nivolumab and steroids. Recent presentations suggest that mNSCLC patients who received concomitant steroids and anti–programmed death-1 (PD-1) receptor or anti– PD-ligand 1 (PD-L1) treatment have worse survival when compared to patients who received anti–PD(L)1 alone [1,2]. However, these analyses were not adjusted to account for residual confounding by variables associated with mortality. Here, we present analyses exploring the effect of confounding variables on the efficacy of concomitant steroid and nivolumab treatment in mNSCLC patients in a real-world setting, using data from CA209-118, a prospective observational study of 70 community practices in the USA.

**Methods**

Only mNSCLC patients who received initial platinum-based therapy and were enrolled before initiating second-line treatment between April 2014 and January 2018 were included in this analysis. Patients were followed until death, initiation of subsequent immunotherapy, end of follow-up, or study withdrawal. Study sites reported therapy start and end dates, sites of metastases, and outcomes. Inverse probability of treatment weighting was used to account for possible residual confounding between factors associated with using steroids and mortality [3].

**Results**

174 patients received nivolumab after initial platinum therapy. Of these, 24 (14%) received systemic steroids at a dose of ≥10 mg, and 17 (10%), 30 (17%), 68 (39%), 28 (16%), and 24 (14%) had liver, brain, lymph node, adrenal, and pleural effusion metastases, respectively.In this analysis, confounding variables included age, sex, ECOG score, duration of first-line therapy, and liver, brain, lymph node, adrenal and pleural effusion metastases. Taking into account the confounding variables, the adjusted Kaplan-Meier curve suggests that concomitant steroid and nivolumab treatment does not negatively impact overall survival (Figure 1).

**Conclusions**

These analyses demonstrate that variables that could result in residual confounding should be considered when evaluating the effects of concomitant steroid and nivolumab treatment. Additional validation in larger cohorts of patients receiving immune checkpoint inhibitors is warranted.

**References**

1. Scott and Pennell. Poster presented at: International Association for the Study of Lung Cancer (IASLC) 2017. Poster number: PS02.09.
2. Arbour KC, Mezquita L, Long N, et al. Poster presented at: 2018 American Society of Clinical Oncology (ASCO) Annual Meeting. Abstract number: 9003.
3. Brookhart MA, Stürmer T, RJ Glynn, et al. Confounding control in healthcare database research: challenges and potential approaches. Med Care. 2010;48(6 Suppl):S114-20.

**Ethics Approval**

This multi-site study was approved by the Western Institutional Review Board (IRB) (December 2013), approval number 20132144; US Oncology IRB (January 2014), approval number 13172; Cone Health IRB (March 2014), approval number 1790; St. Charles Health System IRB (May 2014), approval number not applicable; and the UCLA IRB (May 2015), approval number 14-000865.

**Fig. 1 (abstract P692).**
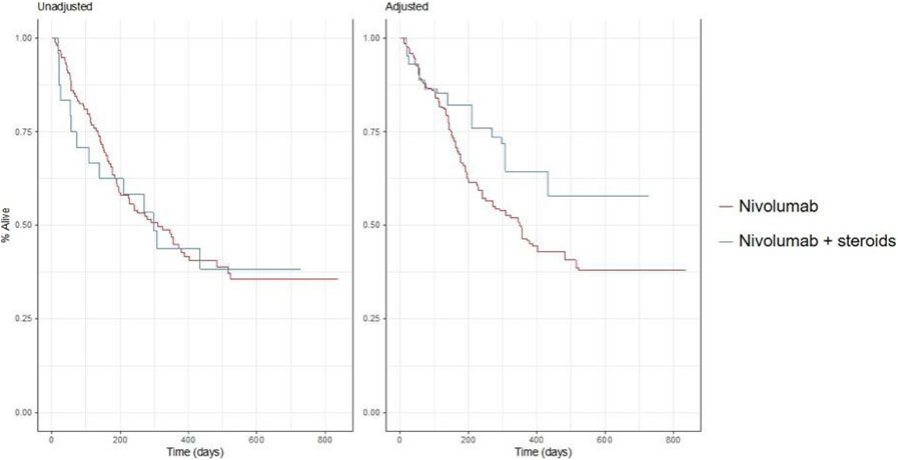
See text for description

#### P693 Efficacy of ex-vivo PD-1 blockade in cervical tumor draining lymph nodes is related to a CD8+FoxP3+ T-cell subset with high levels of multiple immune checkpoints and superior effector functions

##### Jossie Rotman, MD^1^, Marijne Heeren, MSc^2^, Anita Stam^1^, Noëlle Pocorni, MsC^1^, Awa Gassama^1^, Sanne Samuels, MD, PhD^2^, Maaike Bleeker^1^, Stijn Mom, MD PhD^2^, Henry Zijlmans, MD PhD^3^, Gemma Kenter, MD,PhD^2^, Ekaterina Jordanova, PhD^1^, Tanja de Gruijl, PhD^1^

###### ^1^Amsterdam UMC, Cancer Center Amsterdam, Amsterdam, Netherlands; ^2^Amsterdam UMC, Amsterdam, Netherlands; ^3^Netherlands Cancer Institute, Amsterdam, Netherlands

####### **Correspondence:** Jossie Rotman (j.rotman@vumc.nl)

**Background**

An important prognostic factor in cervical cancer (CxCa) is lymph node metastasis. Our previous findings of PD-L1 expression in primary tumors and high and interrelated rates of Tregs and PD-L1-positive macrophages in metastatic tumor-draining lymph nodes (TDLN) point to the possible applicability of PD-(L)1 blockade to halt metastatic spread (Heeren et al, 2015). Here, through extensive flowcytometric profiling and ex-vivo functional analyses, we confirm the validity of PD-1 blockade in early-stage CxCa and relate its efficacy to the presence of a specific CD8+ effector T-cell subset.

**Methods**

Multicolor flow cytometric analysis of T-cells in TDLN (n=23) and PT (n=10) was performed. In addition, the effect of PD-1 blockade on T-cell reactivity against the HPV16 E6 oncoprotein in TDLN (n=12) and PT (n=7) single cell suspensions was assessed by IFNγ Elispot read-out after 10 days in-vitro culture. Cytokine and Granzyme-B production was analyzed after anti-CD3 stimulation of metastatic TDLN (n=4) and PT samples (n=3). Moreover, multicolor immunofluorescence histochemistry was performed on FFPE sections from metastatic TDLN (n=4) and PT (n=4) to study T-cell localization.

**Results**

Extensive flow cytometric analysis revealed progressively elevated levels of activated regulatory T-cells (aTregs) and central and effector memory T-cells, from tumor negative to tumor positive TDLN, to PT. Similarly, significantly and progressively increasing levels of multiple immune checkpoints were observed on both CD4+ and CD8+ T-cells. High levels of PD-1 supported further exploration of PD-1 blockade. Ex-vivo PD-1 blockade consistently enhanced measurable T-cell responses to HPV16 E6 in TDLN with HPV-16+ metastases (4/4), but, remarkably, only in 1/4 HPV16+ PT. Whereas activated Treg (aTreg) rates were significantly higher in PD-1 non- responders, in responders elevated levels of CD8+ CD25+ FoxP3+ T-cells were observed, which correlated significantly with the efficacy of PD-1 blockade (p=0.018). This subset, mainly found in the peritumoral compartment in the tumor microenvironment, was characterized by an activated effector phenotype with elevated expression levels of PD-1, CTLA-4, Tim-3 and Lag-3 checkpoints, but, rather than exhausted, was shown upon ex- vivo polyclonal activation to express higher levels of Granzyme B and effector cytokines as compared to its CD8+FoxP3- counterparts.

**Conclusions**

These data support earlier reports of a “poised” HPV-specific T-cell repertoire in metastatic TDLN and PT and show it to be an actionable target for PD-1 blockade, which may benefit from additional depletion of aTregs. Moreover, they specifically point to a CD8+CD25+FoxP3+ T-cell subset as likely therapeutic target for PD-1 blockade.

**References**

1. Heeren AM, Koster BD, Samuels S, Ferns DM, Chondronasiou D, Kenter GG, et al. High and interrelated rates of PD-L1+CD14+ antigen-presenting cells and regulatory T cells mark the microenvironment of metastatic lymph nodes from patients with cervical cancer. Cancer Immunol Res. 2015;3:48–58.

**Ethics Approval**

The study was approved by the local Institutional Review Boards of the Antoni van Leeuwenhoek (AVL) Hospital and the Academic Medical Center (AMC), both in Amsterdam.

#### P694 An orally bioavailable small molecule dual antagonist of TIGIT and PD-L1 pathways shows immune- mediated anti-tumor activity

##### Murali Ramachandra, PhD^2^, Pottayil Sasikumar, PhD^1^, Sudarshan Naremaddepalli, PhD^2^, Sandeep Patil, PhD^2^, Chennakrishnareddy Gundala^2^, Raghuveer Ramachandra, PhD^2^, Nagesh Gowda, PhD^2^, Saikrishna Tangela^2^, Sreenivas Adurthi, PhD^2^, Amit Dhudashia, MSc^2^, Dodheri Samiulla, PhD^2^, Nagaraj Gowda, PhD^2^, Kavitha Nellore^2^, Murali Ramachandra, PhD^2^

###### ^1^Aurigene Discovery Technologies, Bangalore, India; ^2^Aurigene Discovery Technologies Limited, Bangalore, India

####### **Correspondence:** Murali Ramachandra (murali_r@aurigene.com)

**Background**

Immune checkpoint inhibition using antibodies targeting CTLA4 and PD-1/PD-L1 is considered as a major breakthrough in cancer therapy in recent years. Apart from PD-1 and CTLA-4, there are several other checkpoint proteins in tumor microenvironment that play a role in dampening the anti-tumor immune response. Interestingly, these immune checkpoint pathways are non-redundant thus providing an opportunity for simultaneous targeting more than one checkpoint protein to overcome the immune tolerance in the tumor. T cell Ig and ITIM domain (TIGIT) is a recently identified co-inhibitory receptor expressed by activated T cells, Tregs, and NK cells. TIGIT binds two ligands, CD112 (PVRL2, nectin-2) and CD155 (PVR), and these ligands are expressed by T cells, APCs, and tumor cells. TIGIT is upregulated on tumor antigen-specific CD8^+^ T cells and CD8^+^ tumor-infiltrating lymphocytes in various cancer types and TIGIT receptor/poliovirus receptor (PVR) ligand interaction signaling inhibits cytotoxicity mediated by NK and CD8+ T cells. Interestingly, TIGIT-expressing CD8^+^ T cells often co-express the inhibitory receptor PD-1 providing a strong rationale for simultaneously targeting TIGIT and PD-L1 for optimal anti-tumor activity.

**Methods**

We sought to discover and develop small molecule immune checkpoint antagonists capable of simultaneously targeting TIGIT and PD-L1 pathways. We reasoned that such agents will be amenable for oral dosing, likely show greater response rate due to dual antagonism and allow better management of irAEs due to shorter pharmacokinetic profile. In order to develop an oral agent, we took the approach of identifying a minimal pharmacophore from the interface of TIGIT/PVR interactions via truncating the interfacial sequences. Considering the pockets of sequence similarity of PDL1 and TIGIT proteins a focused library of small molecule compounds, based on minimal pharmacophore, mimicking the interaction of checkpoint proteins was designed and synthesized to achieve compounds exhibiting dual antagonism towards TIGIT and PD-1 pathways.

**Results**

The dual antagonism of these agents have been inferred through potent rescue of PVR-mediated inhibition of IL-2 production from T cells and PD-L1 mediated IFN-γ production. The SAR optimized lead compounds exhibits desirable invitro ADME and DMPK profile including oral bioavailability and better tumor distribution. The lead compounds exhibit significant anti-tumor activity in a syngeneic tumor model and demonstrated profound immune PD in vivo on both T and NK cells.

**Conclusions**

Preclinical data demonstrate a proof-of-concept showing that a dual antagonism of oral small molecules antagonizing TIGIT and PD-L1 pathways significantly enhance anti-tumor efficacy. Efficacy studies and biomarker characterization in additional tumor models are ongoing.

#### P695 Differential expression of microRNAs in immune cell subpopulations during checkpoint inhibitor treatment

##### Barbara Seliger, MD, PhD^1^, Rolf Kiessling, MD, PhD^1^

###### ^1^Martin Luther University Halle-Wittenber, Halle, Germany; ^2^Karolinska Institutet, Stockholm, Sweden

####### **Correspondence:** Rolf Kiessling (rolf.kiessling@ki.se)

**Background**

Checkpoint blockade has revolutionized the treatment of metastatic melanoma, with significant increases in overall survival, and a dramatic improvement in patient quality of life. Ipilimumab, a fully human antibody that blocks CTLA-4, was the first checkpoint inhibitor that received FDA approval in 2011. Despite the success of this therapeutic approach, the number of responding patients is limited and there is a need for predictive, prognostic and pharmacodynamic biomarkers. Non-coding RNAs have recently been shown to have important clinical implications in cancer therapeutics and could be used as targets or as the aforementioned biomarkers for diagnosis, prognosis and prediction of responses to ipilimumab treatment. This study aims (i) to determine the expression of miR-155, known to be a potent regulator of immune cell activity, in untreated and ipilimumab treated melanoma patients and (ii) to correlate miR-155 expression pattern with clinical response to treatment.

**Methods**

30 metastatic melanoma receiving ipilimumab treatment at Karolinska Hospital were enrolled in this study. 6/30 patients had a long-term survival of > 182 weeks. 2/6 responders were Braf V600E mutated, 4/6 expressed wt Braf. CD4+, CD8+ as well as CD14+ cells were sorted from PBMCs purified before, during and at the end of treatment. Expression of miR-155 in each of the purified cellular populations was measured by qPCR.

**Results**

Interestingly, data show a constitutive and stable expression of miR-155 in CD14+ monocytes prior and during the whole course of ipilimumab treatment. In contrast, an induction of miR-155 was found in the different T cell subpopulations. In 7/30 patients an upregulation of miR-155 expression in CD8+ T cells was found with a frequency ranging between 3.6 to 70.3 fold, while in 9/30 patients miR-155 was upregulated in CD4+ T cells in a range between 3.5 to 96 fold. A coordinated upregulation of miR-155 in both T cell subpopulations was only found in 2 patients demonstrating distinct effects of ipilimumab on miR-155 expression in the distinct immune cell subpopulations. In order to determine whether the distinct miR-155 expression pattern had clinical relevance, the miR-155 expression pattern was correlated to the response to ipilimumab. 1/6 responders showed a dramatic 96-fold increase of miR-155 expression in CD4+ T cells over time. In contrast, an upregulation of miR-155 expression was only seen in non-responders.

**Conclusions**

Currently, samples are analyzed for general miR expression pattern in order to identify other differentially expressed miRs, which might serve as markers for monitoring of therapy response and resistance.

#### P696 Combined anti-PD-1 and anti-LAG-3 checkpoint blockade enhances CD8+ TIL effector function while reducing Tregs leading to reduced immune suppression and improved overall survival

##### Elizabeth Sturgill, PhD^1^, Courtney Mick, BS^1^, David Jenkins, PhD^2^, Johanna Kaufmann, PhD, MSc^2^, William Redmond, PhD^1^

###### ^1^Earle A. Chiles Research Institute, Portland, OR, USA; ^2^Tesaro, Waltham, MA, USA

####### **Correspondence:** William Redmond (william.redmond@providence.org)

**Background**

Checkpoint inhibition is a potent strategy to reinvigorate T cells. However, aCTLA-4 or aPD-1 monotherapy has not been effective for the majority of patients, resulting in the exploration of combinatorial approaches to improve treatment efficacy. One such target is LAG-3, which is upregulated on T cells that have experienced repeated antigen exposure, such as in the tumor microenvironment (TME), and is associated with reduced T cell effector function. In addition, high LAG-3 expression on regulatory T cells (Tregs) has been reported for patients with varying cancer types, providing an additional rationale for targeting LAG-3 with the aim of reducing immune suppression within the TME. We hypothesized that the combination of aPD-1 and aLAG-3 would synergize to promote tumor regression and increase survival via a reduction in tumor-induced immune suppression and enhanced CD8+ T cell effector function.

**Methods**

CT26 (colon carcinoma) tumor-bearing BALB/c mice received aPD-1 and/or aLAG-3 (200 μg/dose; ip) 3x/week on days 7, 10, and 13 post-tumor implant. Tumor growth (area) was assessed 2-3x/week and mice were sacrificed when tumors exceeded 150 mm2. In additional cohorts, tumors were harvested 7 days post-treatment (d17) and tumor- infiltrating lymphocytes (TIL) were analyzed by flow cytometry. Responders to combined aPD-1/aLAG3 therapy were designated as those exhibiting decreased tumor size on the day of harvest (d17) compared to maximum tumor growth post-implant.

**Results**

Combined aPD-1/aLAG-3 immunotherapy significantly improved the survival of CT26 tumor-bearing mice compared to monotherapy (p<0.05). Further analysis revealed that aPD-1/aLAG-3 therapy significantly increased the percentage of CD8+ TIL compared to aPD-1 (p<0.01) or aLAG-3 (p<0.05) alone. Additionally, we observed increased effector function in CD8+ TIL from aPD-1/aLAG-3-treated mice, as evidenced by increased cytotoxicity (granzyme A; p<0.05) and cytokine production (TNF-a; p<0.05 and IFN-g; p<0.01). Interestingly, responders to aPD-1/aLAG-3 therapy were enriched for CD8+ TIL with higher cytolytic activity and effector cytokine production, which correlated with a reduction in PD-1 MFI amongst the PD-1+/CD8+ TIL. Lastly, aPD-1/aLAG-3 treatment significantly increased the frequency of effector CD4+ T cells (Teff) compared to FoxP3+CD4+ Tregs (p<0.05).

**Conclusions**

In summary, these data suggest that aPD-1/aLAG-3 immunotherapy increased recruitment of CD8+ TIL exhibiting enhanced effector function, increased CD4+ Teff/Treg ratios, which likely mitigated Treg-mediated immune suppression. Together, these positive immunological changes led to a more immune stimulatory TME capable of supporting tumor regression and significantly improved tumor-free survival.

#### P697 Preclinical characterization of AB154, a fully humanized anti-TIGIT antibody, for use in combination therapies

##### Joanne Tan, PhD^1^, Amy Anderson, PhD^1^, Daniel DiRenzo, PhD^1^, Annette Becker, PhD^1^, Susan Lee, PhD^1^, Lisa Seitz, MSc^1^, Rick Stanton, MSEE^2^, Hema Singh^1^, Sharon Zhao^1^, Nigel Walker, PhD^1^, Matthew Walters, PhD^1^

###### ^1^Arcus Biosciences, Inc., Hayward, CA, USA; ^2^Stanton Biosciences, Newbury Park, CA, USA

####### **Correspondence:** Matthew Walters (mwalters@arcusbio.com)

**Background**

TIGIT (T-cell immunoreceptor with Ig and ITIM domains) is an inhibitory receptor expressed on natural killer (NK) cells, CD8+ T cells, CD4+ T cells and regulatory T cells (Treg). CD226 (DNAX Accessory Molecule-) is an activating receptor found on NK cells, monocytes and a subset of T cells. TIGIT and CD226 are paired receptors that compete for shared ligands CD155 (PVR) and CD112 (Nectin-2) expressed by cancer and antigen-presenting cells. TIGIT binding to CD155 results in immune suppression, whereas binding of CD226 to the same ligand promotes immune activation. AB154 is a fully humanized antibody that binds and blocks human TIGIT with sub- nanomolar affinity.

**Methods**

TIGIT and CD226 expression in healthy and cancer patient PBMCs were assessed by flow cytometry. TIGIT and CD226 expression in various tumor types and normal tissues was derived from TCGA (The Cancer Genome Atlas), GTEX (Genotype-Tissue Expression Project), and immunohistochemistry. Binding affinity of AB154 was determined using CHO.hTIGIT and human T cells. Functional consequences of TIGIT blockade were determined using a TIGIT-expressing reporter gene cell line and a mixed lymphocyte reaction assay. Receptor occupancy (RO) analyses were quantified using a competing anti-TIGIT antibody.

**Results**

Data assembled from TCGA identified tumor types in which expression of TIGIT is greater than PD-1, equivalent to PD-1, or expressed at low levels. TIGIT and PD-1 marked primary tumor samples containing T cells. In these tumors, TIGIT and PD-1 expression were higher compared to normal adjacent tissue. Immunophenotyping performed on dissociated human tumor cells demonstrated strong correlation between TIGIT and PD-1 expression on immune cells. The intensity of TIGIT staining was lowest on conventional CD4+ T cells while its intensity in Treg and CD8+ T cells was 1.5 to 3-fold higher on average. Consistent with a high degree of PD-1 and TIGIT co- expression, combination of AB154 with anti-PD-1 (AB122) significantly increased IFN-gamma secretion relative to anti-PD-1 alone. Using flow cytometry, we demonstrated target engagement by AB154 in T cells and NK cells in the low nanomolar range in both healthy and cancer patient whole blood.

**Conclusions**

Blockade of multiple immune checkpoint proteins can confer effective and durable responses in the treatment of cancer. The data presented here provide: 1) selection of tumor types based on TIGIT RNA and protein expression profile, 2) rationale for combining AB154 with AB122 in upcoming clinical trials, and 3) methodology to evaluate TIGIT receptor occupancy in AB154 Phase 1 studies.

#### P698 Outcomes of advanced triple negative breast cancer patients enrolled in immune oncology clinical trials

##### Tira Tan, MBBS, Lisa Wang, David Cescon, Eitan Amir, David Warr, Christine Elser, Marcus Butler, MD, Albiruni Razak, Aaron Hansen, Anna Spreafico, MD PhD, Lillian Siu, MD, Philippe Bedard, MD

###### Princess Margaret Cancer Centre, Toronto, Canada

####### **Correspondence:** Philippe Bedard (Philippe.Bedard@uhn.ca)

**Background**

Advanced triple negative breast cancer (aTNBC) is an aggressive disease with poor prognosis. Immune-oncology (IO) agents are under investigation for this disease. We evaluated the outcomes of aTNBC patients (pts) enrolled on IO clinical trials at a large academic medical center and explored factors associated with IO treatment outcomes.

**Methods**

We retrospectively reviewed the medical records of aTNBC patients who consented for IO monotherapy or combination clinical trials at Princess Margaret Cancer Centre between June 2013 and June 2018. Demographics data, medical history, details of trial enrolment and response to study treatment according to RECIST 1.1 were recorded. Univariable logistic regression was used to identify factors associated with poor outcomes defined as screen failure due to rapidly progressive disease (PD) or central nervous system metastases (CNS) and/or duration of treatment of 21 days or less.

**Results**

A total of 99 pts with aTNBC consented for 15 IO clinical trials, 60% IO monotherapy, 22% chemotherapy-IO combination and 18% IO combinations. Median age at time of trial enrolment was 52 (range 25-78) and median number of lines of prior systemic therapy for advanced disease was 1 (range 0-8). ECOG performance status, was 0 (39%), 1(58%) and 2/unknown (3%). 15% had de-novo metastatic disease, 58% recurred after a distant disease free interval (DDFI) of less than 3 years and 25% after a DDFI of more than 3 years. 61% had fewer than 3 metastatic disease sites, and 71% had metastases involving the viscera. Of patients consented, 67% started trial treatment and 33% were screen failures, including 19 due to rapid PD and/or CNS metastases. Median progression free survival (mPFS) and overall survival (mOS) in all treated pts were 1.9 months (95% CI 1.7-3.4) and 10.6 months (95% CI 7.7-17.2). In pts who achieved partial response (PR), the mPFS was 7.6 months [3.75-not reached (NR)] and mOS NR. 32% of pts had poor outcomes. In univariate analysis, higher Royal Marsden Index (RMI) (p=0.01), higher Princess Margaret IO prognostic index (PM-IPI) (p=0.01), elevated LDH (p=0.002), higher number of metastatic sites (p=0.03) and presence of visceral metastases (p=0.01) were associated with disease related screen failures and/or duration on IO trial treatment of 21 days or less. (Table 1, 2)

**Conclusions**

The overall prognosis of aTNBC pts enrolled in IO clinical trials is poor with heterogeneous treatment outcomes. Pts with poor prognostic indices, elevated LDH, higher number of metastatic sites and visceral metastases should be reconsidered for IO trials.

**Ethics Approval**

The study was approved by UHN Research Ethics Board, approval number 15-9269.

**Table 1 (abstract P698).**
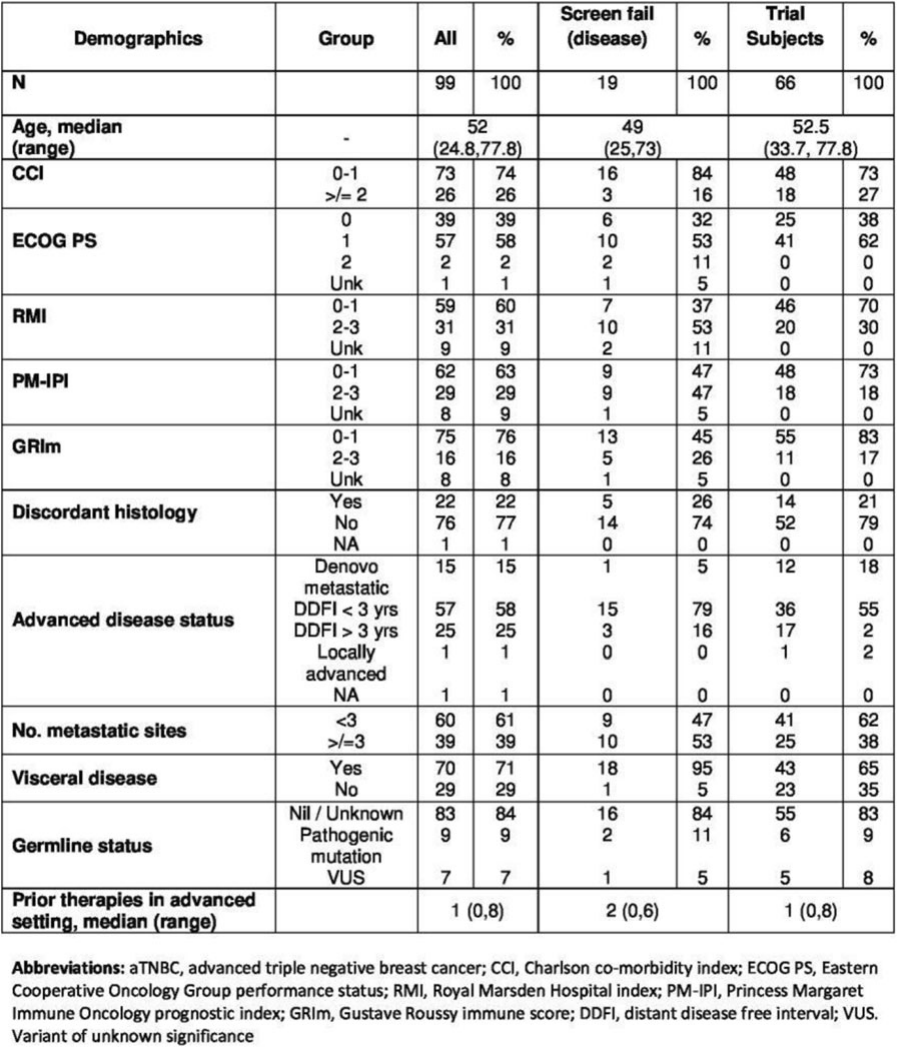
See text for description

**Table 2 (abstract P698).**
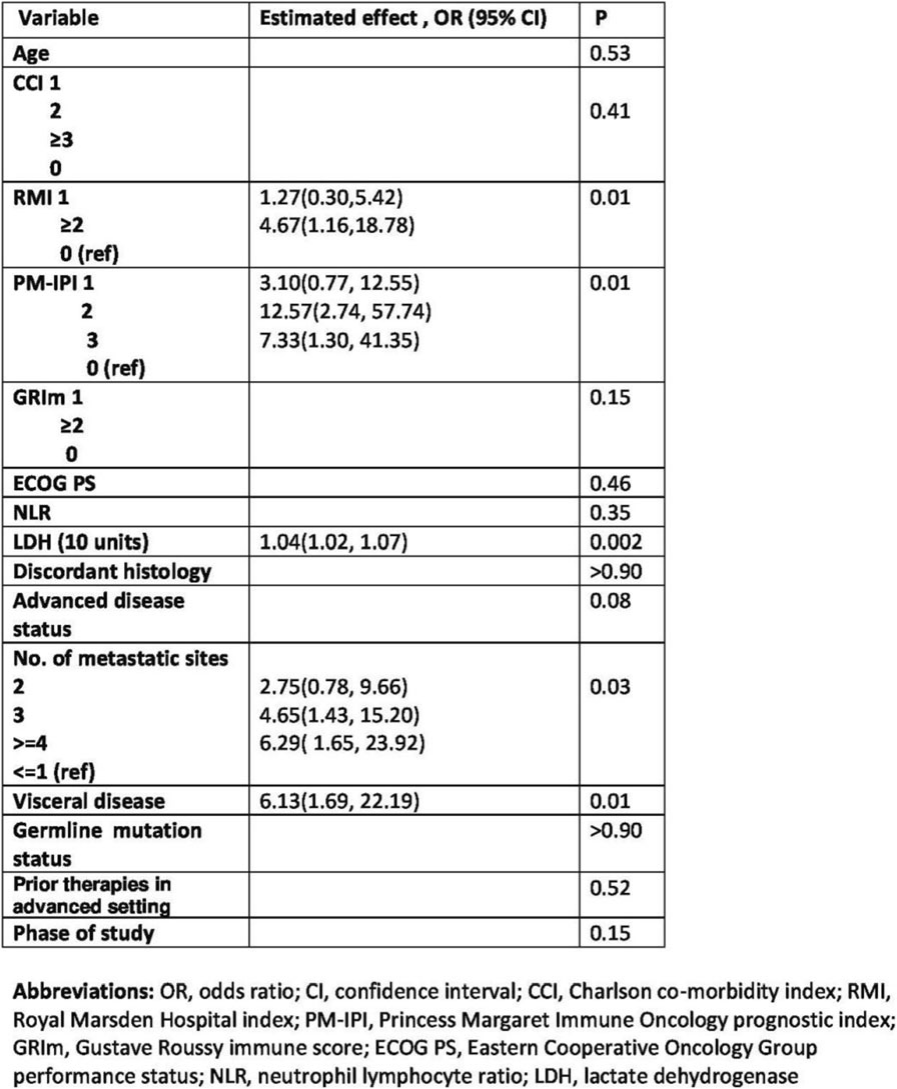
See text for description

#### P699 MEDI0562, a humanized OX40 agonist monoclonal antibody (mAb), increases T cell effector function and depletes regulatory T cells in blood and tumor

##### Katie Streicher, PhD^2*^, Roger Wild^1^, Keith Steele, DVM, PhD^2^, Steven Eck, PhD^2^, Yanan Zheng, PhD^2^, Farzad Sekhavati, PhD^3^, Han Si^2^, Fernanda Pilataxi^2^, Song Wu, PhD^2^, Brandon Higgs, PhD^2^, Danielle Townsley^2^, Rakesh Kumar, PhD^2^, Mike Sheehan, PhD^2^, Scott Hammond, PhD^2^, Matthew Gribbin^2^, Victoria Chiou, MD^2^, Maria Jure- Kunkel^2^, Sandip Patel, MD^4^, John Powderly, MD, CPI^5^, Bonnie Glisson, MD^6^, Koustubh Ranade, PhD^2^

###### ^1^Ashfield Healthcare; ^2^MedImmune, Gaithersburg, MD, USA; ^3^Definiens, Munich, Germany; ^4^University of California San Diego, La Jolla, CA, USA; ^5^Carolina BioOncology Institute, Huntersville, NC, USA; ^6^MD Anderson Cancer Center, Houston, TX, USA

####### **Correspondence:** Katie Streicher (streicherk@medimmune.com)

**Background**

In preclinical models, OX40 agonists display a dual mechanism of action (MOA) that leads to antitumor activity: stimulating effector/memory T cell function and depleting regulatory T cells. To determine if these pharmacodynamic changes are measurable in patients with advanced solid tumors, we evaluated blood and tumor samples pre/post treatment in a Phase 1 trial of MEDI0562, a humanized OX40 agonist mAb (NCT02318394).

**Methods**

Patients received 1 of 6 escalating doses of MEDI0562 (0.03, 0.1, 0.3, 1.0, 3.0, and 10 mg/kg) Q2W until confirmed disease progression or unacceptable toxicity. Tumor response was assessed using irRECIST and RECIST. Selected patients with head and neck squamous cell carcinoma, bladder or cervical cancer had mandatory pre- and on- treatment tumor biopsies to evaluate pharmacodynamic changes. Tumor samples were evaluated pretreatment and at day 29 from a subset of 14 patients by quantitative digital analysis of immunohistochemistry images and gene expression. Peripheral blood from all patients with evaluable samples (n = 36) was monitored using gene expression and flow cytometry.

**Results**

A total of 55 patients received MEDI0562 across 6 dose cohorts where 10 mg/kg Q2W was the maximum administered dose. Serum exposure increased approximately dose proportionally. Post-treatment antidrug antibodies (ADAs) were detected in 51% of patients, with an impact on MEDI0562 PK at all doses below 3 mg/kg. The activity of MEDI0562 was evaluated in both blood and tumor. In blood, a 1.5- to 3.0-fold increase in mean maximum percentage of Ki67+CD4+ and Ki67+CD8+ memory T cells was observed across doses. Ratios of T effector/T regulatory gene expression signatures increased 3.5- to 8-fold in blood (p < 0.05) at all doses at 2 days post-treatment. In tumor, paired biopsies showed ≥ 2-fold increased expression of PD-L1 and/or CD8+ T cell infiltration in 7/14 patients, with a 60% median reduction in OX40+FOXP3+ cells at doses of 1 and 3 mg/kg compared with doses <1 mg/kg. Ratio of T effector/T regulatory gene expression signatures increased intratumorally as seen peripherally. Intratumoral pharmacodynamic changes were more prominent in patients with high OX40 expression (> median) regardless of MEDI0562 dose or presence of ADAs.

**Conclusions**

MEDI0562-treated patients exhibited increased Ki67+CD4+ and CD8+ memory T cells in the periphery and decreased intratumoral OX40+FOXP3+ cells, consistent with the hypothesized dual MOA of this agonist mAb. The enhancement of these pharmacodynamic effects in patients with high OX40 levels at baseline may help identify patients or indications where MEDI0562 could provide clinical benefit.

**Trial Registration**

ClinicalTrials.gov [NCT02318394]

**Ethics Approval**

Multicenter study conducted at 3 sites:(1) MD Anderson Cancer Center, 7007 Bertner Avenue, Unit 1637, Houston, TX 77030, USA [PI: Bonnie Glisson; IRB Registration No. IRB00000121](2) University of California San Diego Moores Cancer Center, 9444 Medical Center Drive, 3rd Floor, Room 3-030, La Jolla, CA 92093, USA [PI: Sandip Patel; IRB Registration No. Committee O: 00009940](3) Carolina BioOncology Institute, PLLC, 1019 39th Avenue South East, Suite 120, Puyallup, WA 98374-2115, USA [PI: John Powderly II; IRB Registration No. IRB00000533]

#### P700 Immune checkpoint inhibitors induce differential anti-tumor response and immune cell infiltration across syngeneic models

##### Brandy Wilkinson, PhD^2^, Patrick Allison, PhD^2^, Hua-Chen Chang^2^, Lauren Ursic, BA^2^, Paul Trampont, PhD^2^, Lindsey Standarski^2^, Jennifer Rusk, NA^2^

###### ^1^Integrated Oncology & Covance (LabCorp), Greenfield, IN, USA; ^2^Covance, Greenfield, IN, USA

####### **Correspondence:** Brandy Wilkinson (Brandy.Wilkinson@covance.com)

**Background**

Clinical success of immune checkpoint inhibitors has accelerated the evaluation of immune-related targets for novel anti-cancer therapies. Preclinical testing of immune-targeted oncology agents requires preclinical models with functional immune systems. Utilization of murine syngeneic tumor models provides a robust system to evaluate both anti-tumor activity and mechanism of action of novel therapeutics. This study evaluates the efficacy and anti-tumor immune response of different IO therapies across a panel of syngeneic murine models.

**Methods**

Cohorts of mice were inoculated with six different murine derived cancer cell lines (MC38, CT-26, LL/2, EMT-6, 4T1, B16F10). Tumor bearing mice were administered anti-CTLA-4, anti-PD-1, anti-PD-L1, anti-OX40 or anti- LAG3 twice weekly for 3 weeks. Tumor volume was used to assess anti-tumor activity. Subsets of tumors from treated mice were analyzed two weeks following dosing initiation for tumor-infiltrating lymphocytes (TILs) and myeloid cell populations.

**Results**

Following 3 weeks of dose administration: anti-CTLA4, anti-PD-1, anti-PD-L1, anti-OX40, and anti-LAG3 conferred significant antitumor activity on some of the xenograft models surveyed, while other models were resistant. Phenotypes of TILs resident in tumors were profiled across the xenograft models. CD45+ lymphocytes were analyzed for populations of cytotoxic lymphocytes (CTLs, CD8+ cells), T-helper cells (CD4+ cells), regulatory T-cells (T-regs, CD4+/CD25+/FoxP3+ cells), and myeloid populations including B-Cells, neutrophils and monocytes. The immune cell response to checkpoint inhibitors were distinct, but varied across xenograft models.

**Conclusions**

In this study, we demonstrate differential responses between immune-checkpoint inhibitors across several syngeneic xenograft models with respect to anti-tumor activity and lymphocyte tumor infiltration responses. These models constitute a highly-relevant tool to evaluate efficacy and mechanism of action for novel immune-targeted therapies for oncology.

#### P701 Off-label use of immunotherapy in advanced malignancies

##### David Xu, Kathryn Gold, MD, Lyudmila Bazhenova, MD, Sandip Patel, MD

###### UCSD Moores Cancer Center, San Diego, CA, USA

####### **Correspondence:** Kathryn Gold (kagold@ucsd.edu)

**Background**

Immunotherapy with checkpoint inhibitors (CI) has become a standard treatment in an increasing number of cancers. Off-label use of these agents is common but their efficacy is not known.

**Methods**

We performed a retrospective review of 98 consecutive patients treated at a single institution using CI through a patient assistance program with start dates between 5/2015 to 11/2016. Patients were excluded from further analysis if they received treatment for an FDA-approved indication.

**Results**

Sixty-one patients were included in our analysis: 41% male; 75% white, 3% black, 7% Asian, 11% Hispanic, and 3% other. Median age at diagnosis was 59; median age at start of CI was 62. Median time between diagnosis and start of CI was 23 months. Most patients had good performance status at the time of CI initiation (69% ECOG 0 or 1). Most patients had metastatic disease (87%) at the start of CI. All but 3 patients had received prior chemotherapy; most had prior surgery (70%) and prior radiation (69%). Twenty patients received nivolumab (33%), and 41 patients received pembrolizumab (67%). Two patients had received prior immunotherapy (anti-CD40, tremilumumab). Most common tumor types were breast (11), sarcoma (7), thyroid (5), glioblastoma, hepatocellular carcinoma, and ovarian cancer (four each). Time on therapy ranged between 0 and 21+ months (median 2 months). Eight patients remained on therapy at the time of analysis (9.6 to 21.6 months). Ten patients were on therapy for 1 year or more (esophageal SCC, triple negative breast cancer [2], nasopharyngeal carcinoma, pleomorphic sarcoma, adrenal cortical carcinoma, cutaneous SCC, uterine sarcoma, basal cell carcinoma). Median number of cycles of CI was 5. 20% of the patients had some evidence of response, 21% stable disease, 7% mixed response, and 38% progression of disease as their best treatment response per clinician assessment. Restaging was not available for 15% of patients. Median PFS defined as time from first dose of CI to date of progression was 2 months. Median overall survival was 9 months.

**Conclusions**

In this study, we observed activity in a wide variety of malignancies with off label use of CIs. Several patients with uncommon cancers have remained on therapy for an extended period of time. For rare cancers for which clinical trials are not feasible, retrospective analyses such as this can suggest some evidence of efficacy.

#### P702 Acute liver injury in the context of immune checkpoint inhibitor-related colitis treated with infliximab

##### Hao Chi Zhang, MD^1^, Wenyi Luo, MD^2^, Yinghong Wang, MD, PhD^2^

###### ^1^UT Health Science Center at Houston, Houston, TX, USA; ^2^University of Texas MD Anderson Cancer Center, Houston, TX, USA

####### **Correspondence:** Yinghong Wang (ywang59@mdanderson.org)

**Background**

Immune checkpoint inhibitors (ICPI) are commonly used in the treatment of several advanced cancers. ICPI can also be associated with immune-related adverse events (irAEs) including enterocolitis and hepatitis. Infliximab has been used successfully in treating steroid-refractory gastrointestinal irAE [1], but it carries the risk for liver injury. We describe a challenging case of a patient with a new diagnosis of acute hepatitis after infliximab treatment for gastrointestinal irAE.

**Methods**

A 79-year-old man with a history of metastatic prostate adenocarcinoma, treated with ipilimumab and nivolumab, was evaluated for new elevation in liver enzymes and bilirubin. He had no known risk factors for acute or chronic liver disease. After three cycles of ipilimumab and nivolumab, he had developed grade 3 diarrhea/colitis, and ICPI was discontinued. His diarrhea was steroid-refractory and required a one-time treatment with infliximab (5 mg/kg) which led to prompt resolution of diarrhea. Three weeks later, the patient developed jaundice with elevated liver enzymes and total bilirubin (Figures 1-2). Physical examination did not reveal stigmata of advanced liver disease. A liver biopsy showed cholestatic hepatitis with predominantly microvesicular steatosis, and mild portal and peri- portal fibrosis (Figure 3). A short course of steroid treatment did not improve the hepatitis condition. By exclusion of other etiologies, acute drug-induced liver injury secondary to infliximab was deemed to be the most likely cause of his liver condition, rather than an adverse effect of ICPI. By fourteen weeks post-infliximab administration, liver enzymes and total bilirubin eventually returned close to normal baseline levels (Figures 1-2).

**Results**

N/A

**Conclusions**

Ipilimumab- and nivolumab-related irAEs have been reported; high-grade liver irAE in particular occur with an incidence of <10% [2-5]. In the context of gastrointestinal irAE management, concern was raised about liver toxicity from infliximab, a previously described effective rescue therapy. In our patient’s case, the clinical history and data supported infliximab-associated hepatotoxicity, rather than an irAE. Lack of pathognomonic histologic features from hepatic irAE [6-7] limited the value of liver biopsy in this situation. With the increasing application of ICPI for different cancers and the understanding of potential risks for irAE, liver function and liver biochemical tests should be closely monitored during treatment using ICPI and during treatment using anti-TNF-α agents in this patient population.

**References**

1. Pagès C, Gornet JM, Monsel G, et al. Ipilimumab-induced acute severe colitis treated by infliximab. Melanoma Res. 2013;23(3):227-30.
2. National Institutes of Health. LiverTox: Clinical and Research Information on Drug-Induced Liver Injury. Ipilimumab. [online] Available at: https://livertox.nlm.nih.gov/Ipilimumab.htm [Accessed 28 Jul 2018].
3. National Institutes of Health. LiverTox: Clinical and Research Information on Drug-Induced Liver Injury. Nivolumab. [online] Available at: https://livertox.nlm.nih.gov/Nivolumab.htm [Accessed 28 Jul 2018].
4. DeSouza K, Savva C. Management of Immunotherapy Related Adverse Effects. J Cancer Prev Curr Res. 2016;6(1):00187.
5. Ibrahim RA, Berman DM, DePril V, et al. Ipilimumab safety profile: Summary of findings from completed trials in advanced melanoma. J Clin Oncol 2011;29(15):8583-8583.
6. Kleiner DE, Berman D. Pathologic changes in ipilimumab-related hepatitis in patients with metastatic melanoma. Dig Dis Sci. 2012;57(8):2233-40.
7. Zen Y, Yeh MM. Hepatotoxicity of immune checkpoint inhibitors: a histology study of seven cases in comparison with autoimmune hepatitis and idiosyncratic drug-induced liver injury. Mod Pathol. 2018;31(6):965-973.

**Fig. 1 (abstract P702).**
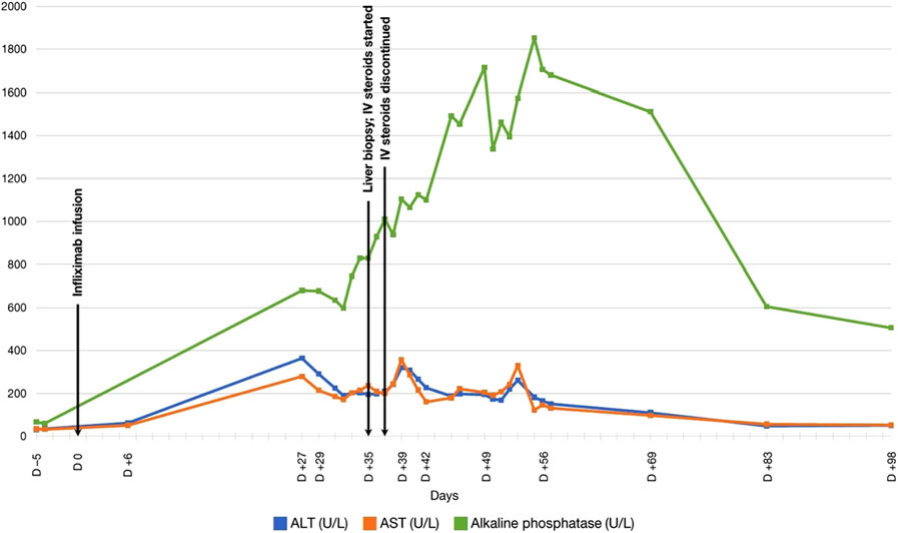
Trends in liver enzymes

**Fig. 2 (abstract P702).**
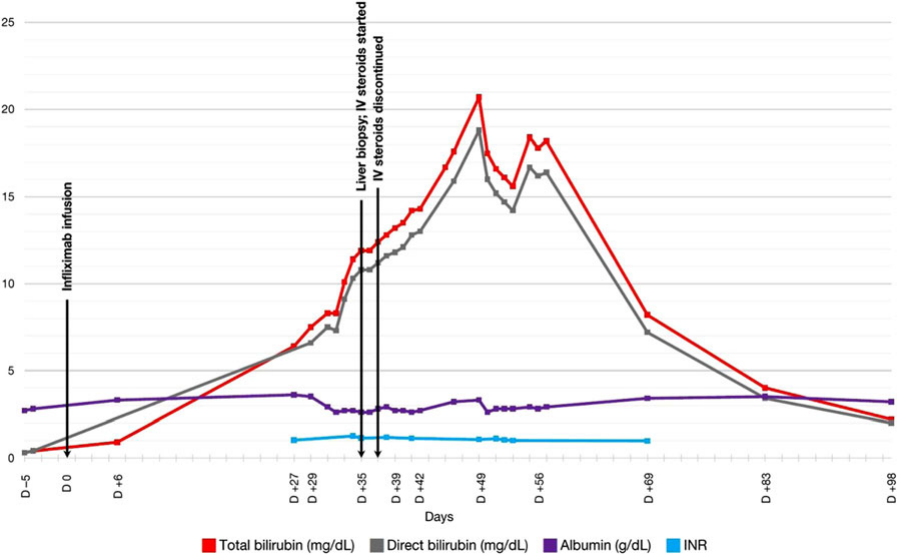
Trends in additional liver biochemical tests

**Fig. 3 (abstract P702).**
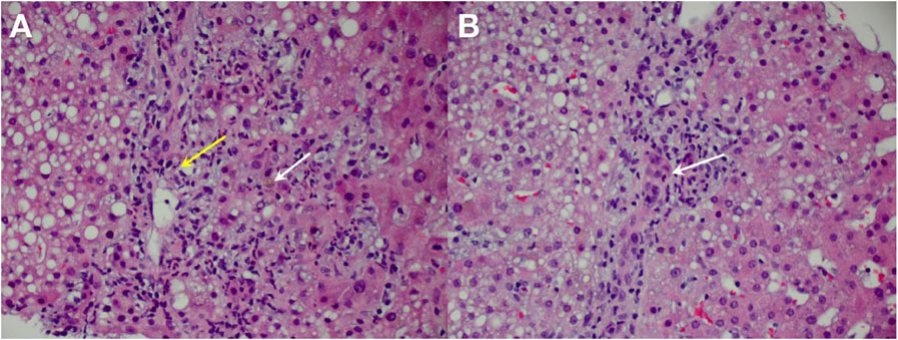
Liver biopsy histology

#### P703 Exploration of PD-1/PD-L1 spatial interaction and T cells functionality to predict anti-PD-1 treatment outcome in GI tract tumors using automated quantitative fluorescence multiplexed IHC

##### Xiangxue Wang, BS^1^, Shizen Moh, BS^1^, José L. Muñoz-Rodríguez, PhD^1^, Antony Hubbard, BS^1^, Mehrnoush Khojasteh, PhD^1^, Qingfeng Zhu, PhD^2^, Robert A. Anders, MD, PhD^2^, Luis A. Diaz, MD^2^, Lidija Pestic-Dragovich^1^, Lei Tang, PhD^1^, Wenjun Zhang, MD, PhD^1^

###### ^1^Roche Tissue Diagnostics, Tucson, AZ, USA; ^2^John Hopkins Univeristy Hospital, Balitmore, MD, USA

####### **Correspondence:** Wenjun Zhang (wenjun.zhang@roche.com)

**Background**

Blockade of the PD-1/L1 axis is the effective immunotherapy in a proportion of cancer patients. Identification of predictive biomarkers for patient selection represents a major challenge. The predictive value of PD-L1 IHC, tumor mutational load and mismatch repair (MMR) status are limited for the variable strength of association among studies and tumor types. This study aims to quantitatively assess the content and spatial interaction of key immune suppression components (e.g. PD-1 & LAG3 for T cell exhaustion, PD-1/L1 interaction) in tumor micro- environment, and their predictive values to anti-PD-1 treatment.

**Methods**

Multiplex IHC for PD-L1, PD-1, CD8, LAG3, and pan-cytokeratin (panCK) stained 50 pre-pembrolizumab treatment patient specimens including pancreatic, colorectal and cholangio carcinoma, among which 17 are MMR proficient (13PD, 3SD, and 1CR) and 33 deficient (3PD, 12SD, 3NE, 4CR, and 11PR). Whole slides were scanned with ZEISS Axio Scan.Z1 scanner, on which pathologists annotated tumor area. HALO® Hi-Plex software was used for image analysis. Epithelial tumor (panCK+), stroma (panCK-), artifacts, and no-tissue areas were annotated with Halo’s random forest classifiers. X/y coordinates, number, intensity and density of PD-1+, PD-L1+, LAG3+, and CD8+ cells were identified. CD8+ cells co-expressing PD-1, PD-L1, and/or LAG3 phenotypes and their fraction over CD8+ cells were extracted. Spatial relationships including the number of PD-1+ cells within 10/20um of PD- L1+ cells and the average distance between them were computed. MATLAB® was used for feature selection, reduction, ranking and prediction of the responses to anti-PD-1 treatment.

**Results**

Responders versus non-responders classes were defined as PR and CR versus SD, PD, and NE. From ~190 features analyzed, both Relieff and random forest rank the following feature in the top two features in predicting response to treatment: “Maximum number of CD8+/low-intensity-PD-1+ cells within 20um of PD-L1+ cells in epithelial tumor”. Quadrant Discriminant Analysis (QDA) with five-fold cross validation yields a prediction accuracy of 85%. When combined with the “average number of PD-1+ cells within 20um radius of PD-L1+” and the "max value of CD8+ cell Lag3+ intensity", the accuracy reaches 90.2%, regardless of the MMR status. Other features have lower accuracy (e.g. 60-70%). Independent cross-validation was not performed due to small sample size.

**Conclusions**

Deep immune characterization of tumor microenvironment on high dimensional image features from multiplexed IHC staining may provide insightful directions on finding and validating predictive markers for PD-1/PD-L1 blockage and other immunotherapies.

**Acknowledgements**

We acknowledge Sriravali Kamthamraju for her technical assistance on image analysis, John Hurley, Jorge Lozano and Nick Cummins for their technical assistance on assay development.

#### P704 Batf3 dendritic cells within the tumor microenvironment are necessary during the effector phase of the immune response for anti-PD-L1 efficacy through a mechanism involving the 4-1BB/4-1BB ligand axis

##### Andrea Ziblat, PhD^1^, Brendan L. Horton^2^, Philip C. He, BA^1^, Thomas F. Gajewski, MD, PhD^1^

###### ^1^University of Chicago, Chicago, IL, USA; ^2^MIT, Boston, MA, USA

####### **Correspondence:** Thomas F. Gajewski (tgajewsk@medicine.bsd.uchicago.edu)

**Background**

Spontaneous CD8+ T cell responses against tumor antigens can be detected in both cancer patients and in murine models. However, immunosuppressive mechanisms arise within the tumor microenvironment that blunt T cell functions and enable tumor escape. Immunotherapy that targets the interaction between the T cell inhibitory receptor PD-1 and its ligand PD-L1 can generate durable tumor regressions that translate into clinical benefit across many cancer types. However, many patients fail to respond to immune checkpoint blockade and many others acquire resistance. Therefore, understanding the mechanisms involved in anti-PD-L1 immunotherapy efficacy may enable new strategies for improving efficacy. We and others previously demonstrated that Batf3-lineage dendritic cells (DCs), which express the markers CD8α or CD103, act in at least two steps in anti-tumor immunity: 1) spontaneous T cell priming in the tumor-draining lymph node, and 2) recruitment of effector CD8+ T cells to the tumor [1-3]. In the current work, we examined whether Batf3+ DCs are also required at a third level, during the effector phase of the anti-tumor immune response upon treatment with PD-1/PD-L1 blockade.

**Methods**

We utilized the B16-SIY melanoma model, CD11c-DTR-GFP bone marrow chimeras and CD11c-DTR-GFP/Batf3 KO mixed bone marrow chimeras to study the role Batf3-DCs play during anti-PD-L1 immunotherapy. To focus on the effector phase of the immune response we depleted CD11c+ cells with diphtheria toxin from day seven of tumor injection while simultaneously blocking T cell entry into the tumor with FTY720. In other experiments anti-4-1BBL blocking antibody was administered alone or in combination with anti-PD-L1 simultaneously with FTY720. Also, wild type or 4-1BB-deficient splenocytes were transferred into Rag KO mice and anti-PD-L1 efficacy was evaluated. Tumor growth and phenotypic analysis of the tumor infiltrate was evaluated in all of the experiments.

**Results**

Interestingly, we found that CD11c+ cells, and specifically Batf3-CD11c+ cells, must be present in the tumor before the beginning of anti-PD-L1 treatment for therapeutic efficacy of anti-PD-L1. Phenotypic analysis of the tumor infiltrate showed high expression of 4-1BBL on CD11c+CD103+ DCs compared to other antigen-presenting cell subsets. Unexpectedly, when anti-4-1BBL blocking antibody was co-administered with anti-PD-L1, therapeutic efficacy was lost. In addition, transfer of 4-1BB-deficient splenocytes into Rag KO hosts revealed a requirement of 4-1BB expression on T cells for anti-PD-L1 efficacy.

**Conclusions**

Overall, these results suggest that Batf3-DCs are necessary during the effector phase of the immune response for anti-PD-L1 efficacy, at least in part through the co-stimulation of 4-1BB signaling on CD8+ TILs.

**References**

1. Fuertes MB, Kacha AK, Kline J, Woo SR, Kranz DM, Murphy KM, Gajewski TF. Host type I IFN signals are required for antitumor CD8+ T cell responses through CD8α+ dendritic cells. J Exp Med. 2011; 208(10):2005-2016.
2. Roberts EW, Broz ML, Binnewies M, Headley MB, Nelson AE, Wolf DM, Kaisho T, Bogunovic D, Bhardwaj N, Krummel MF. Critical role for CD103(+)/CD141(+) dendritic cells bearing CCR7 for tumor antigen trafficking and priming of T Cell immunity in melanoma. Cancer Cell. 2016; 30(2): 324-336.
3. Spranger S, Dai D, Horton B, Gajewski TF. Tumor-residing Batf3 dendritic cells are required for effector T cell trafficking and adoptive T cell therapy. Cancer Cell. 2017; 31(5):711-723.

#### P705 Time to GoInVivo(TM), validated checkpoint functional antibodies for cancer research

##### Miguel Tam, PhD, Josh Croteau

###### BioLegend

####### **Correspondence:** Miguel Tam (mtam@biolegend.com)

**Background**

Immune checkpoint molecules control the fate of an immune response. Well- studied combinations of molecules include PD-1/PD-L1, CTLA-4/CD80 and CD86, LAG-3/MHC II, and Tim-3/Galectin 9. Our GoInVivo™ antibodies, against some of these immune checkpoint molecules, offer several advantages. They have been tested by flow cytometry and in vitro bioassays, are pathogen-free as tested by qPCR, and have excellent price for large sizes, among others. Here we present our results on the use of anti-mouse PD-L1 in the treatment of a mouse cancer model, melanoma. We characterize the phenotype and localization of T cells, in the tumor microenvironment and draining lymph nodes. We also study the cytokine profile in serum, as well as the antigen-specific T cell response.

**Methods**

Balb/cJ or C57BL/6J mice were injected with 106 B16F10 melanoma cells in the flank and treated with 100 or 200 μg of anti-PD-L1 at the time points indicated. Tissue samples and serum were collected and analyzed by flow cytometry, microscopy, or screened for cytokine content with LEGENDplex™.

**Results**

CD4 and CD8 T cells redistribute in the spleen (Figure 1). Animals were implanted for 14 days with B16 melanoma cells, after receiving 3 doses of anti-PD-L1 the CD8/CD4 T cell ratio increases. Splenic CD4 T cells show an activated phenotype (Figure 2). After tumor implant and antibody treatment, CD4 T cells show increased expression of CD25, CD69, CD278, and CD279. Similar results were observed in CD8 T cells. CD8+ cells infiltrate the tumor and co-localize with CD11c+ cells (Figure 3). After tumor implant and antibody treatment, the tumors were excised and analyzed by fluorescent microscopy. Pictures were taken with a 40X objective. Cytokine and chemokine profile in serum. After tumor implant and antibody treatment, the analysis of the samples with LEGENDplex™ show A) Increased Th Cytokines. B) Decreased pro-inflammatory cytokines. C) Increased chemokine production. (Figure 4)A) Tumor-specific infiltrating CD8+ T cells increase 24 days after treatment. Animals were implanted with SIYRYYGL-expressing B16 cells and 24 days after treatment the tumor was collected and analyzed. B) Anti-PD-L1 treatment reduces tumor growth. (Figure 5)

**Conclusions**

Injection of anti-mouse PD-L1 in mice implanted with B16 melanoma cells:1. Redistributes CD4 and CD8 T cell content in spleen and tumor, and induce activation of T cells2. Stimulates production of Th cytokines and chemokines, while suppressing pro-inflammatory cytokine production 3. Increases tumor-specific T cells, as well as IFN-g-producing cells (not shown) 4. Reduces tumor growth

**Fig. 1 (abstract P705).**
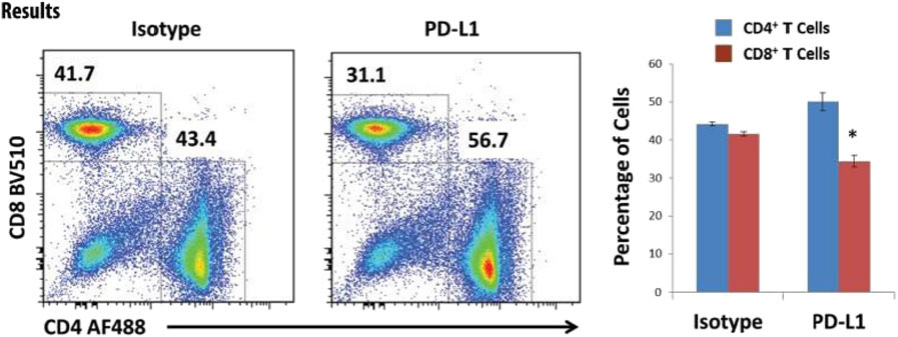
CD4 and CD8 T cells redistribute in the spleen. Animals were implanted for 14 days with B16 melanoma cells, after receiving 3 doses of anti-PD-L1 the CD8/CD4 T cell ratio increases

**Fig. 2 (abstract P705).**
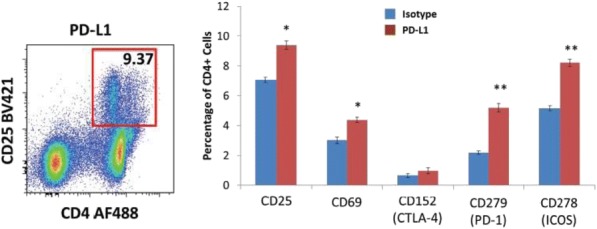
Splenic CD4 T cells show an activated phenotype. After tumor implant and antibody treatment, CD4 T cells show increased expression of CD25, CD69, CD278, and CD279. Similar results were observed in CD8 T cells (not shown)

**Fig. 3 (abstract P705).**
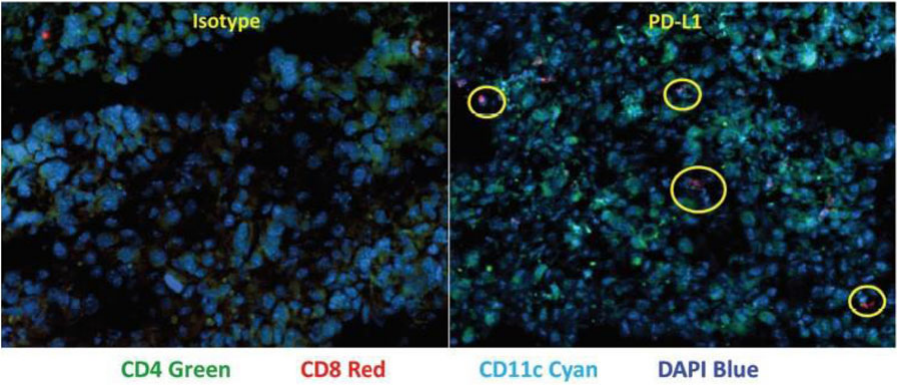
CD8^+^ cells infiltrate the tumor and co-localize with CD11c^*^ cells. After tumor implant and antibody treatment, the tumors were excised and analyzed by fluorescent microscopy. Pictures were taken with a 40X objective

**Fig. 4 (abstract P705).**
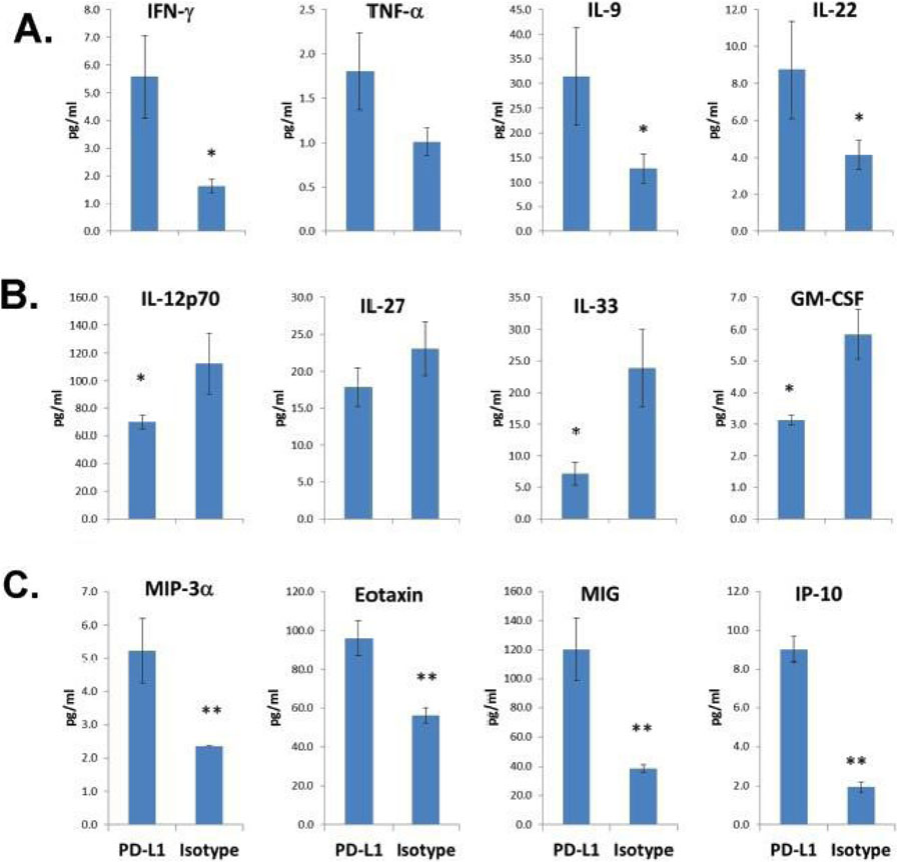
Cytokine and chemokine profile in serum. After tumor implant and antibody treatment, the analysis of the samples with LEGENDplex^TM^ show A) Increased Th Cytokines. B) Decreased pro-inflammatory cytokines. C) Increased chemokine production

**Fig. 5 (abstract P705).**
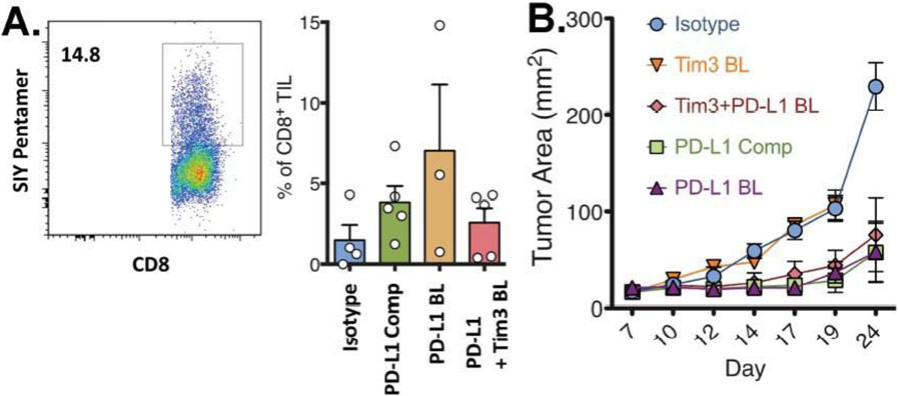
A) Tumor-specific infiltrating CD8^+^ T cells increase 24 days after treatment. Animals were implanted with SIYRYYGL-expressing B16 cells and 24 days after treatment the tumor was collected and analyzed. B) Anti-PD-L1 treatment reduces tumor growth

## Oral Presentations

### Biomarkers and Immune Monitoring

#### O1 Identification and profiling of neoantigen-specific T cells in NSCL cancer patients treated with atezolizumab

##### Michael Fehlings^1^, Suchit Jhunjhunwala, BS, PhD^2^, Marcin Kowanetz, PhD^2^, Bill O'Gorman^2^, Priti Hegde, PhD^2^, Jessica Li, Master of Science^2^, Hermi Sumatoh^1^, Boon Heng Lee^1^, Alessandra Nardin, DVM^1^, Mahesh Yadav, PhD^2^, Leesun Kim^2^, Susan Flynn^2^, Marcus Ballinger, PhD^2^, Evan Newell, PhD^3^

###### ^1^immunoSCAPE, Singapore, Singapore; ^2^Genentech, South San Francisco, CA, USA; ^3^SiGN, Singapore, Singapore

####### **Correspondence:** Mahesh Yadav (yadav.mahesh@gene.com)

**Background**

There is strong evidence that immunotherapy-mediated tumor rejection is associated with the reinvigoration of tumor-specific CD8+ T cells most likely recognizing neoantigens derived from tumor somatic mutations. However, despite a substantial number of mutations present in some tumors, only a small fraction of neoantigens have been shown to be immunogenic, partly due to the challenge in identifying rare neoantigen-specific CD8+ T cells in tumor-bearing individuals.

**Methods**

We employed mass cytometry and highly-multiplexed combinatorial tetramer staining together with cellular barcoding and high-dimensional phenotypic characterization to longitudinally monitor neoantigen-specific CD8+ T cells in PBMC from 14 NSCL cancer patients treated with atezolizumab. Close to 800 candidate tumor neoantigens and 73 viral-derived control peptides were screened across all patient samples; T cells were simultaneously profiled using 30 or more markers.

**Results**

Virus-specific T cells were detected in most patient samples at frequencies as low as 0.004% of total CD8+ T cells. T cells reactive for 13 different neoantigens were also identified with a medium to high confidence across all patients and time points. Interestingly, the majority of medium-to-high confidence hits (9/13) were detected among the 8 patients who presented an objective response to treatment, with only 4 of the 13 hits detected in the 6 patients with progressive disease. The neoantigen-specific cells differed phenotypically from bulk CD8+ T cells in the peripheral blood and displayed a diverse phenotype.

**Conclusions**

This study demonstrates the utility of the use of mass cytometry together with a combinatorial tetramer staining for the ex vivo identification, characterization, and longitudinal follow-up of rare tumor-specific T cells. Importantly, it suggests that the detection of neoantigen-specific T cells may be used as a predictor of response to checkpoint blockade and supports further research into this.

**Trial Registration**

NCT01903993

**References**

1. Atezolizumab versus docetaxel for patients with previously treated non-small-cell lung cancer (POPLAR): a multicentre, open-label, phase 2 randomised controlled trial The Lancet. Volume 387, No. 10030, p1837–1846, 30 April 2016

**Ethics Approval**

This study was performed on de-identified samples from POPLAR trial, a multicentre, randomized, open-label, allcomer, multicenter phase 2 trial. The POPLAR trial was performed in full accordance with the guidelines for GCP and the Declaration of Helsinki. Protocol; approval was obtained from an independent ethics committee for each trial site.

**Consent**

All patients gave appropriate ethical approval for this analysis. Statements confirming compliance with ethical regulations, the committees that approved the POPLAR study protocol, and confirmation of informed consent from all study participants are included in the previous publications describing the POPLAR trial [1].

#### O2 Using artificial intelligence to distinguish subjects with prostate cancer (PCa) from benign prostate hyperplasia (BPH) through immunophenotyping of MDSCs and lymphocyte cell populations

##### John Roop^1^, Alex Polo^1^, Anthony Campisi, BS^1^, Dmitry Gabrilovich, MD, PhD^2^, Amit Kumar, PhD^1^, George Dominguez, PhD^1^

###### ^1^Anixa Biosciences, San Jose, CA, USA; ^2^The Wistar Institute, Philadelphia, PA, USA

####### **Correspondence:** George Dominguez (george@ituscorp.com)

**Background**

Myeloid-derived suppressor cells (MDSCs) are key contributors in supporting tumor progression and tumor escape through their ability to suppress anti-tumor responses mediated through T cell and natural killer (NK) cell activity [1,2]. Several studies have quantified MDSCs to detect tumor development, monitor progression, and/or predict therapeutic responses [3, 4]. The objective of this study was to determine if flow cytometry data analysis of MDSCs and other leukocytes could be incorporated with a supervised machine learning classifier to identify individuals presenting either with a prostate malignancy (PCa) or benign condition (such as benign prostate hyperplasia or BPH).

**Methods**

We used standard multiparametric flow cytometry techniques to immunophenotype MDSCs and other leukocytes found in the peripheral blood of 73 PCa, 48 BPH, and 73 control subjects; all prostate pathologies were confirmed with a transrectal ultrasound guided prostate (TRUSP) biopsy. Subjects were excluded if they had a previous history of cancer (not including subjects under active surveillance), had a medical intervention for prostate cancer, or were receiving a dihydrotestosterone (DHT) or alpha-1 blocker for active treatment of BPH. Next, a series of neural networks were created with inputs consisting of the numerical event counts from the flow cytometry FCS file (fluorescent or scatter intensity values). Three datasets were constructed: the training dataset – to ‘teach’ two output categories through backpropagation and parameter fitting; the validation dataset – to evaluate the fit to minimize overfitting; and the test dataset – to rank the trained networks against each other and estimate the classification performance. Finally, a naïve testing set (i.e. never seen by the network) was used to determine the overall performance of the top-ranking networks after voting.

**Results**

With this approach, we were able to distinguish PCa subjects (both high and low grade) from control subjects with 91.7% accuracy (AUROC = 0.929; 95% CI: 0.8418 to 1.016). Using the same approach, we can further identify PCa from BPH subjects with 87.5% accuracy (AUROC = 0.869; 95% CI: 0.7938 to 0.9442).

**Conclusions**

By pairing supervised machine learning with the immunophenotyping of MDSCs and other leukocytes using flow cytometry, we have developed a novel method for distinguishing PCa from control or BPH subjects with high levels of accuracy. We believe this methodology could be used to predict patient responses to immunotherapies and/or to monitor tumor recurrence.

**References**

1. Kumar V, Patel S, Tcyganov E, Gabrilovich D. The nature of myeloid-derived suppressor cells in the tumor microenvironment. Trends Immunol. 2016; 37:208-220.

2. Marvel D, Gabrilovich D. Myeloid-derived suppressor cells in the tumor microenvironment: expect the unexpected. J Clin Invest. 2015; 125:3356-3364.

3. Elliott L, Doherty G, Sheahan K, Ryan E. Human tumor-infiltrating myeloid cells: phenotypic and functional diversity. Front Immunol. 2017; 8:86.

4. Okla K, Wertel I, Wawruszak A, Bobinski M, Kotarski J. Blood-based analyses of cancer: circulating myeloid-derived suppressor cells – is a new era coming? Crit Rev Clin Lab Sci. 2018.

**Ethics Approval**

The study was approved by The Wistar Institute's Institutional Review Board, protocol number 21802305.

#### O3 Anti-tumor immune responses in metastatic breast cancer exceptional responder patients

##### Alusha Mamchak, PhD^1^, Ngan Nguyen, PhD^1^, Danhui Zhang, MD PhD^1^, Felix Chu, MS^1^, Mike Harbell, BS^1^, Beatriz Millare, BS^1^, Kevin Williamson^1^, Xiaomu Chen^1^, Xiaobin Tang^1^, Shuwei Jiang^1^, Dongkyoon Kim, BS PhD^1^, Nicole Hasser^1^, Sarah Hippely^2^, Maren Levin^2^, Amy Manning-Bog, PhD^1^, Jeff DeFalco^1^, william Robinson, MD^1^, Daniel Emerling, PhD^1^, Norman Greenberg, PhD^1^, Guy Cavet, PhD^1^, Joyce O’Shaughnessy, MD^2^

###### ^1^Atreca Inc, Redwood City, CA, USA; ^2^Baylor University Medical Center, Dallas, TX, USA

####### **Correspondence:** Alusha Mamchak (amamchak@atreca.com)

**Background**

Analyzing anti-cancer immune responses can offer insights into the mechanisms that underlie successful cancer therapies. While the role of T cell immunity in anti-cancer responses has been well characterized, the role of the humoral immune response to cancer remains less clear. Thus, we set out to identify anti-tumor antibodies from 11 metastatic breast cancer (MBC) patients who had exceptional responses to systemic therapy with multi-year benefit, all of whom were disease-free or long-term non-progressors. The patients’ breast cancers were diverse with respect to hormone receptor status, HER2 status, and treatment history.

**Methods**

We used flow cytometry to isolate plasmablasts, which are antibody secreting cells produced in lymphoid tissues through activation, affinity maturation, and differentiation of antigen-specific naive and memory B cells. Natively paired IgG sequences were generated from individual cells using Immune Repertoire Capture® (IRC™) technology. Similar immunoglobulin sequences were grouped into putative antibody lineages based on germline gene usage and CDR3 sequence features.

**Results**

A total of 9160 native pairs of expression-ready, heavy and light chain immunoglobulin sequences were generated. The antibody sequences were grouped into putative clonal lineages, with 931 of these lineages being expressed by two or more plasmablasts, providing evidence of selection and expansion. Comparison of the immunoglobulin repertoires across patients revealed several cases in which similar families of antibodies were identified in more than one patient, suggesting convergent selection. Antibodies in the putative convergent families were predominantly IgG2 (86%) which was significantly higher than the frequency of IgG2 in non-convergent lineages.By computationally selecting immunoglobulin sequences and expressing them as recombinant proteins, patient-derived antibodies were identified that bind to human and mouse cancer cell lines, including human breast and lung cancer, and tissues derived from xenogeneic murine models of human breast and prostate cancer. In addition, several antibodies were shown by immunohistochemistry to bind specifically to non-autologous human breast cancer tissue but not to adjacent breast tissue.

**Conclusions**

Through the use of IRC™ technology and antibody binding assays, the humoral immune response in MCB exceptional responder patients has been quantified. Of particular interest, this study has identified patient derived-antibodies that bind specifically to non-autologous breast cancer tissue and have potential to form the basis of new cancer therapeutics.

#### O4 Immune monitoring after NKTR-214 plus nivolumab (PIVOT-02) in previously untreated patients with metastatic Stage IV melanoma

##### Adi Diab, MD^1^, Scott Tykodi^2^, Brendan Curti, MD^3^, Daniel Cho, MD^4^, Michael Wong, MD PhD FRCPC^1^, Igor Puzanov, MD, MSCI, FACP^5^, Karl Lewis, MD^6^, Michele Maio, MD, PhD^7^, Gregory Daniels, MD, PhD^8^, Alexander Spira, MD, PhD, FACP^9^, Mary Tagliaferri, MD^10^, Alison Hannah, MD^10^, Wendy Clemens, PhD^10^, Michael Imperiale^10^, Chantale Bernatchez^1^, Cara Haymaker, PhD^1^, Salah Eddine Bentebibel^11^, Jonathan Zalevsky, PhD^10^, Ute Hoch, PhD^10^, Christie Fanton, PhD^10^, Ahsan Rizwan, MPharm, PhD^10^, Sandra Aung, PhD^10^, Fiore Cattaruzza^10^, Ernesto Iaccucci^10^, Dariusz Sawka^12^, Mehmet Bilen, MD^13^, Paul Lorigan^14^, Giovanni Grignani^15^, James Larkin, MD^16^, Sekwon Jang, MD^17^, Ewa Kalinka-Warzocha, PhD, MD^18^, Mario Sznol, MD^19^, Michael Hurwitz, MD, PhD^19^

###### ^1^The University of Texas MD Anderson Cancer Center, Houston, TX, USA; ^2^University of Washington and Fred Hutchinson Cancer Research Center, Seattle, WA, USA; ^3^Providence Cancer Institute and Earle A. Chiles Research Institute, Portland, OR, USA; ^4^NYU Medical Oncology Associates, New York, NY, USA; ^5^Roswell Park Cancer Institute, Buffalo, NY, USA; ^6^University of Colorado Denver, Aurora, CO; ^7^Azienda Ospedaliera Universitaria Senese, Siena, Italy; ^8^Moores Cancer Center, University of California San Diego, La Jolla, CA, USA; ^9^Virginia Cancer Specialists, PC, Fairfax, VA, USA; ^10^Nektar Therapeutics, San Anselmo, CA, USA; ^11^The University of Texas MD Anderson Canc, Houston, TX, USA; ^12^Szpital Specjalistyczny w Brzozowie Podkarpacki Osrodek Onkologiczny, Brzozów, Poland; ^13^Emory University Hospital (Winship Cancer Institute), Atlanta, GA, USA; ^14^The Christie NHS Foundation Trust, Manchester, UK; ^15^Institute for Cancer Research and Treatment (IRCC), Candiolo, Italy; ^16^The Royal Marsden, London, UK; ^17^Inova Schar Cancer Institute, Fairfax, VA, USA; ^18^Instytut Medyczny Santa Familia, Lodz, Poland; ^19^Yale School of Medicine, New Haven, CT, USA

####### **Correspondence:** Adi Diab (adiab@mdanderson.org)

**Background**

In patients with melanoma, low levels of tumor-infiltrating lymphocytes and low/absent PD-L1 expression are associated with limited response to anti-PD-1/anti-PD-L1 therapies. NKTR-214 (IL-2Rβγ-biased cytokine) monotherapy stimulates proliferation and activation of lymphocytes in blood and tumor and increases PD-1/PD-L1 expression. The impact of NKTR-214 and nivolumab on the systemic immune system and local tumor microenvironment is presented.

**Methods**

The melanoma cohort is closed; 41 patients were enrolled with 38 evaluable for efficacy (≥1 follow-up scan). Tumor biopsies were analyzed using multispectral IHC, gene expression, and TCR sequencing. Flow cytometry and hematology were used to evaluate blood cells. PD-L1 expression was evaluated using DAKO, 28-8 PharmDx Assay.

**Results**

Immune monitoring of blood revealed clear activation of the IL-2 pathway following administration of NKTR-214 plus nivolumab. Lymphocyte numbers increased 9x (N=41) from nadir reaching their peak 7 days post dose and maintained that magnitude of increase after each cycle. The proportion of proliferating (Ki67+, n=12) CD4+, CD8+, and NK cells increased 13x, 20x, and 6x over baseline, respectively. Similar immune activation was reported with NKTR-214 monotherapy (8x, 8x, and 7x over baseline, respectively). Immune cells demonstrated an antigen-experienced phenotype with an increased proportion of HLA-DR expression on CD4+, CD8+, and NK cells 3x, 2x, and 6x over baseline, respectively. ICOS levels increased 2x on CD8+ T cells. Baseline and week 3 biopsies (n=12, evaluable) showed local effects on the tumor microenvironment including elevated expression of PD-L1 on the tumor (patients converted from PD-L1 negative to positive), increased total numbers of CD8 infiltrate, and increased proportion of proliferating cells all ranging from 6-17x over baseline. Following treatment, intratumoral gene expression analyses showed elevations in networks associated with the NKTR-214 mechanism of action, including induction of an interferon-gamma gene signature. The investigator-assessed objective response rate as of 12 July 2018 was 50% (N=38), and no responder has relapsed. Deepening of response was observed over time and was associated with immune activation, consistent with the MOA of NKTR-214 plus nivolumab. The median duration of response has not been reached.

**Conclusions**

NKTR-214 is a robust agonist of the IL-2 pathway and together with nivolumab promotes immune activation in the periphery and tumor microenvironment for significant clinical activity. A global phase 3 trial in treatment-naïve advanced melanoma patients of NKTR-214 plus nivolumab versus nivolumab (1:1) will be open for enrollment in 2018.

**Trial Registration**

Clinicaltrials.gov NCT02983045 (PIVOT-02)

#### O5 B-cells and tertiary lymphoid structures (TLS) predict response to immune checkpoint blockade (ICB)

##### Sangeetha Reddy, MD, MSci^1^, MD, MSci^1^, Beth Helmink, MD PhD^1^, Jianjun Gao, MD PhD^1^, Shaojun Zhang, PhD^1^, Keren Yizhak^2^, Moshe Sade-Feldman^3^, Jorge Blando, DVM^1^, Guangchun Han^1^, Vancheswaran Gopalakrishnan, MPH, PhD^1^, Hao Zhao^1^, Wenbin Liu^1^, Hussein Tawbi, MD, PhD^1^, Rodabe Amaria, MD^1^, Michael Davies, MD, PhD^1^, Patrick Hwu, MD^1^, Jeffrey Lee, MD^1^, Jeffrey Gershenwald, MD^1^, Scott Woodman^1^, Elizabeth Burton^1^, Lauren Haydu, MS, BChe, MIPH^1^, Alexander Lazar, MD, PhD^1^, Courtney Hudgens, MS^1^, Alexandria Cogdill, MEng^1^, Oscar Krijgsman, PhD^4^, Elisa Rozeman, MD^4^, Daniel Peeper, PhD^4^, Christian Blank, MD^4^, Ton Schumacher, PhD^4^, Emily Keung^1^, Pierre-Olivier Gaudreau^1^, Alexandre Reuben^1^, Christine Spencer, PhD^1^, Lisa Butterfield, PhD^5^, James Allison, PhD^1^, Michael Tetzlaff, MD PhD^1^, Florent Petitprez, MS^6^, Wolf Herman Fridman, MD, PhD^6^, Catherine Sautes-Fridman, PhD^6^, Nir Hacohen, PhD^2^, Padmanee Sharma, MD, PhD^1^, Linghua Wang, PhD^1^, Jennifer Wargo, MD, MMSc^1^

###### ^1^MD Anderson Cancer Center, Houston, TX, USA; ^2^Broad Institute, Cambridge, MA, USA; ^3^Massachusetts General Hospital, Boston, MA, USA; ^4^The Netherlands Cancer Institute, Netherlands, Netherlands; ^5^University of Pittsburgh Medical Center, Pittsburgh, PA, USA; ^6^Centre de Recherche des Cordeliers, Paris, France

####### **Correspondence:** Jennifer Wargo (JWargo@mdanderson.org)

**Background**

Mutational load, cytotoxic T-cell markers, and PD-L1 have been identified as biomarkers of response to ICB. However, there is a growing understanding of the contribution of B-cells in shaping response to ICB. We conducted a neoadjuvant ICB trial in patients with high-risk resectable melanoma ((NCT02519322), and identified B-cell signatures in responders by protein expression profiling.

**Methods**

To further investigate this, we performed transcriptomic profiling of longitudinal specimens in this melanoma cohort. Differentially expressed genes (DEG) were assessed in baseline samples with adequate tumor purity. Targeted immune profiling was further performed using immune deconvolution tool MCP-counter in all baseline and on-treatment samples, with additional validation from a metastatic renal cell carcinoma (RCC) trial of ICB (NCT02210117) and the melanoma TCGA dataset. Cases were dichotomized by CD8 T-cell scores to study interaction between B- and T-cells. Singlet and multiplex immunohistochemistry assessed spatial organization of the tumor infiltrating B-cells. Single cell RNA sequencing was performed in an independent cohort of metastatic melanoma patients treated with ICB.

**Results**

The most DEG at baseline in melanoma responders to ICB were B-cell related genes such as MZB1, BTLA, and IGLL5 (NR) (p<0.0001 for all). B-cell signatures were confirmed by a targeted immune gene assessment using MCP-counter, showing higher B lineage signatures among responders at baseline (p=0.036) and on-treatment (p=0.038). Among baseline cases, B-cells were more strongly predictive among CD8 T-cell low subsets (p=0.085) compared to T cell high (p=0.833). The applicability of these findings to other tumors was demonstrated in an RCC cohort, in which B lineage scores was predictive of response (p=2.6e-03), and differential effects were again seen between CD8 T-cell low (p=0.008) and high cases (p=0.564). In the melanoma TCGA, B-cell lineage score was correlated with improved survival (p<0.0001 for overall and disease-specific survival), in particular in CD8 T-cell low cases including after multivariable adjustment (p=0.001 for overall survival and 0.006 for disease-specific survival). Single cell sequencing in an independent melanoma cohort identified DEG within B-cells by response, providing insights into B-cell phenotypes associated with outcomes. Assessment of tissue sections from tumor samples in the neoadjuvant melanoma ICB cohort demonstrated co-localization of the B cells in TLS with CD8 and CD4 T-cells and CD21 follicular dendritic cells. The ratio of tumor area occupied by TLS was higher in responders (p=0.037 at baseline and 0.002 on-treatment).

**Conclusions**

Together, these results highlight the potential significance for B-cell signatures as prognostic and predictive factors for response to ICB.

#### O6 Comparison of biomarker assay modalities in anti-PD-(L)1 monotherapy: a meta-analysis

##### Steve Lu, BS^1^, Steve Lu, BS^1^, Ludmila Danilova, PhD^2^, David Rimm, MD, PhD^3^, Clifford Hoyt, MS^4^, Matthew Hellmann, MD^5^, Janis Taube, MD^1^

###### ^1^Johns Hopkins University School of Medicine, Baltimore, MD, USA; ^2^Johns Hopkins Medical Institutions, Baltimore, MD, USA; ^3^Yale University School of Medicine, New Haven, CT, USA; ^4^PerkinElmer, Hopkinton, MA, USA; ^5^Memorial Sloan Kettering Cancer Center, New York, NY, USA

####### **Correspondence:** Janis Taube (jtaube1@jhmi.edu)

**Background**

Substantial effort is ongoing to identify predictors of response to immunotherapy. Numerous PD-L1 immunohistochemistry (IHC) assays are now FDA-approved. More recent biomarker approaches include the assessment of tumor mutational burden (TMB), gene expression profiling (GEP) and quantitative and/or spatial assessment of multiple proteins by multiplex IHC/immunofluorescence (mIHC/IF). The purpose of this project was to determine the relative sensitivity and specificity of these modalities.

**Methods**

We performed a meta-analysis of the association between overall response rate to anti-PD(L)1 monotherapy and PD-L1 IHC, TMB, GEP, or mIHC/IF. For PD-L1 IHC, only clinical trials that resulted in FDA-approved indications for anti-PD-(L)1 monotherapy were included. Due to the earlier development phase of TMB, GEP, and mIHC/IF, all identified publications or meeting abstracts using these modalities were included. Results were filtered to ensure that each study/data set was represented only once. Studies focused on MSI-high tumors were not included in the TMB category. For each individual study, the specificity, sensitivity, negative and positive predictive values (NPV and PPV) were determined according to each individual study’s scoring algorithm of a positive vs. negative test. Summary receiver-operating characteristic (sROC) curves corresponding to each of the modalities were generated with each study 1) weighted equally (i.e. unweighted) and 2) weighted by patient specimen number tested.

**Results**

7454 patient specimens representing 10 different solid tumor types were assayed, and the results were correlated with anti-PD-(L)1 response. This data was derived from n=25 reports that tested PD-L1 IHC, n=11 for TMB, n=6 for GEP, and n=5 for mIHC/IF. When each modality was evaluated with sROC curves, PD-L1 had the lowest predictiveness (unweighted AUC 0.664, weighted AUC 0.659) followed by TMB (unweighted AUC 0.732, weighted AUC 0.708, Figure 1A, 1B). Although GEP and mIF/IHC had the least data available, they had the highest AUCs (unweighted AUC 0.859 and 0.821, weighted AUC 0.877 and 0.785, respectively, Figure 1A, 1B). Most modalities provide relatively high NPV, but mIHC/IF also demonstrates high PPV.

**Conclusions**

Several distinct biomarkers have predictive value in identifying patients most likely to respond to PD-(L)1 therapy. TMB has modestly better performance relative to PD-L1 IHC, and newer approaches such as GEP and mIHC/IF may have improved sensitivity and specificity. Further studies are needed to determine the most predictive analytes and scoring algorithms, and to assess whether biomarker performance varies by tumor type. Composite approaches including more than one of these modalities may perform better than any single modality alone.

**Ethics Approval**

The study was approved by the Johns Hopkins University Institutional Review Board.

**Fig. 1 (abstract O6).**
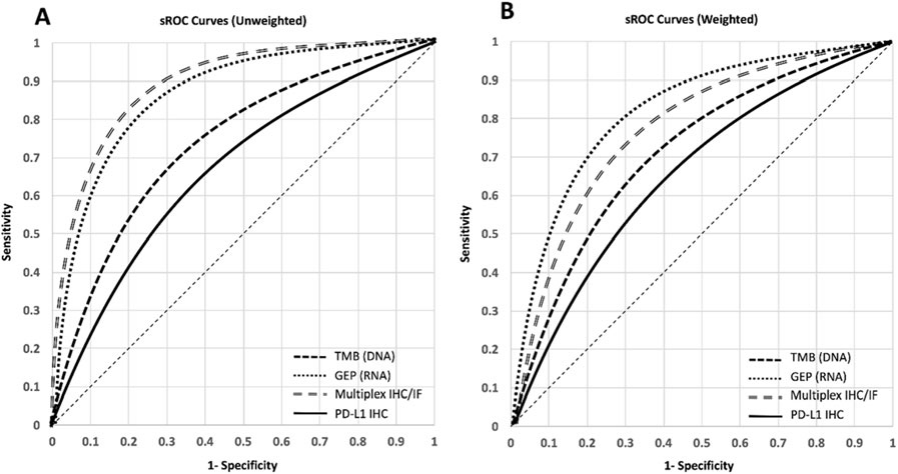
See text for description

### Cancer Vaccines, Personal Vaccines and Tech

#### O7 The personal vaccine, NEO-PV-01 with anti-PD1, induces neoantigen-specific de novo tumor-related immunity in patients with advanced cancer

##### Siwen Hu-Lieskovan, MD, PhD^2^, Ramaswamy Govindan, MD^3^, Aung Naing, MD, FACP^4^, Terence Friedlander, MD^5^, Kim Margolin, MD^6^, Jessica Lin, MD^7^, Nina Bhardwaj, MD, PhD^8^, Matthew Hellmann, MD^9^, Lakshmi Srinivasan^1^, Joel Greshock^1^, Melissa Moles^1^, Richard Gaynor, MD^1^, Matthew Goldstein, MD, PhD^1^, Patrick Ott, MD, PhD^10^

###### ^1^Neon Therapeutics, Inc, Cambridge, MA, USA; ^2^Ronald Reagan UCLA Medical Center, Los Angeles, CA, USA; ^3^Washington University Medical School, Saint Louis, MO, USA; ^4^MD Anderson Cancer Center, Houston, TX, USA; ^5^Division of Hematology/Oncology, UCSF, San Francisco, CA; ^6^City of Hope, Duarte, CA, USA; ^7^Massachusetts General Hospital, Boston, MA, USA; ^8^Icahn School of Medicine at Mount Sinai, New York, NY, USA; ^9^Memorial Sloan Kettering Cancer Center, New York, NY; ^10^Dana Farber Cancer Institute, Boston, MA, USA

####### **Correspondence:** Joel Greshock (jgreshock@neontherapeutics.com)

**Background**

Neoantigens arise from DNA mutations in cancer cells and are important targets for T cell mediated anti-tumor immunity. NEO-PV-01 is a personal neoantigen vaccine of up to 20 peptides designed based on a patient’s neoantigen and HLA profile that is directed at inducing tumor-specific T cell responses to neoantigens. We report clinical and immune data from NT-001, a phase 1b study of NEO-PV-01 + adjuvant in combination with nivolumab in metastatic melanoma, NSCLC and bladder cancer (ClinicalTrials.gov: NCT02897765).

**Methods**

After 12 weeks of nivolumab treatment, patients received NEO-PV-01 vaccine plus adjuvant poly-ICLC in a prime-boost format spanning 12 weeks. The primary endpoint is safety; secondary endpoints include overall response rate (ORR) and the rate of post-vaccination responses. Comprehensive genomic and immune assessments were performed with serial biopsies and apheresis before and after vaccination to characterize treatment-related immune response in each patient.

**Results**

In 31 patients across tumor cohorts, all vaccine-related AE’s were grade 1/2, with the most frequent being injection site reactions and fatigue. There were no vaccine-related SAEs. Among the 13 melanoma patients who received the full vaccine course at the time of data cut, the ORR was 62% (8/13), of which two responses were seen post-vaccination. A total of 11/13 (85%) vaccinated melanoma patients remain on treatment with a median duration of 41.3 weeks (range = 29.6-62.0 weeks). ORR’s were 33% (1/3) for both the NSCLC and bladder cohorts with a median time on treatment of 39.0 and 31.9 weeks respectively. Notably, one NSCLC patient who had stable disease on nivolumab alone had a partial response post-vaccination. Serial immune analysis completed on 10 melanoma patients demonstrated CD4 and CD8 T cell responses against 58% of vaccine peptides as measured by via IFNγ ELISpot. T cell responses were neoantigen-specific for 86% (12/14) of peptides tested. Most T cell responses were polyfunctional, producing multiple cytokines and were of a memory phenotype. Epitope spreading, defined by post-vaccination T cell responses to neoantigens not included in the vaccine, was observed in 4/6 (67%) melanoma patients analyzed. Multi-platform assessments of immune response, including serial exome sequencing and TCR repertoire analysis of tumor biopsies will be presented.

**Conclusions**

Treatment with NEO-PV-01 + adjuvant and nivolumab was well tolerated and demonstrated evidence of clinical activity. In addition, this combination induced broad de novo neoantigen-specific immune responses in metastatic cancers.

#### O8 The GAPVAC approach of actively personalized peptide vaccination for patients with newly diagnosed glioblastoma

##### Norbert Hilf, PhD^1^, Sabrina Kuttruff-Coqui, PhD^1^, Katrin Frenzel^2^, Valesca Bukur^2^, Stefan Stevanovic^3^, Cecile Gouttefangeas, PhD^3^, Michael Platten, MD^4^, Ghazaleh Tabatabai^5^, Valérie Dutoit^6^, Sjoerd van der Burg, PhD^7^, Per Thor Straten^8^, Francisco Martínez-Ricarte, MD^9^, Berta Ponsati^10^, Hideho Okada, MD, PhD^11^, Ulrik Lassen^12^, Arie Admon^13^, Christian Ottensmeier, MD PhD FRCP^14^, Alexander Ulges^1^, Sebastian Kreiter, MD^2^, Andreas von Deimling^4^, Marco Skardelly^5^, Denis Migliorini^6^, Judith Kroep, MD, PhD^7^, Manja Idorn, PhD^8^, Jordi Rodon^15^, Jordi Piro^10^, Hans Poulsen^12^, Bracha Shraibman^13^, Katy McCann^14^, Regina Mendrzyk^1^, Martin Löwer^2^, Monika Stieglbauer^3^, Cedrik Britten, MD^16^, David Capper^17^, Marij Welters^7^, Juan Sahuquillo^9^, Marie Stockhausen^12^, Katharina Kiesel^1^, Evelyna Derhovanessian^2^, Elisa Rusch^3^, Colette Song^1^, Sandra Heesch^2^, Claudia Wagner^1^, Alexandra Kemmer-Brueck^2^, Jörg Ludwig^1^, John Castle^18^, Oliver Schoor, PhD^1^, Jens Fritsche, PhD^1^, Miriam Meyer^1^, Nina Pawlowski, PhD^1^, Sonja Dorner^1^, Franziska Hoffgaard^1^, Bernhard Rössler, Dipl INg (FH), MBA^19^, Dominik Maurer, PhD^1^, Toni Weinschenk, PhD^1^, Carsten Reinhardt, MD, PhD^1^, Christoph Huber, MD^2^, Hans-Georg Rammensee, PhD^3^, Harpreet Singh, PhD^1^, Ugur Sahin, MD^2^, Pierre-Yves Dietrich^6^, Wolfgang Wick, MD^4^

###### ^1^Immatics Biotechnologies GmbH, Tuebingen, Germany; ^2^BioNTech AG, Mainz, Germany; ^3^Eberhard Karls Universität Tuebingen, Tuebeingen, Germany; ^4^University Hospital Heidelberg, Heidelberg, Germany; ^5^University Hospital Tuebingen, Tuebingen, Germany; ^6^Geneva University Hospital, Geneva, Switzerland; ^7^Leiden University Medical Center, Leiden, Netherlands; ^8^CCIT, University Hospital Herlev, Copenhagen, Denmark; ^9^Vall d’Hebron University Hospital, Barcelona, Spain; ^10^BCN Peptides S.A., Barcelona, Spain; ^11^UCSF and Parker Institute, San Francisco, CA, USA; ^12^Ringhospitalet, Copenhagen, Denmark; ^13^Technion - Israel Institute of Technology, Haifa, Israel; ^14^University of Southampton, Southampton, UK; ^15^Vall d’Hebron University Hospital (present address: MDACC, Houston), Barcelona, Spain; ^16^CIMT, BioNTech AG (present address: GSK, UK), Stevenage, UK; ^17^University Hospital Heidelberg (present address: Charité Berlin), Heidelberg, Germany; ^18^BioNTech AG (present address: Agenus Inc., USA), Mainz, Germany; ^19^Immatics biotechnologies GmbH (present address: Rigontec GmbH, Munich), Planegg, Germany

####### **Correspondence:** Wolfgang Wick (wolfgang.wick@med.uni-heidelberg.de)

**Background**

The need for treatment personalization in cancer therapy is evident as every tumor is molecularly unique. Especially immunotherapy should be customized to the highly individual antigenic landscape of every tumor for optimal efficacy. Glioblastoma are immunologically regarded as resistant and “cold” with an average of 30-50 mutations per tumor resulting in very few targetable neoantigens. To fully exploit all available antigens in glioblastoma, mutation-derived neoantigens as well as non-mutated antigens that are over-presented in the individual tumor should be addressed.

**Methods**

The GAPVAC consortium realized an immunotherapy, in which patients with newly diagnosed glioblastoma were offered two peptide-based actively personalized vaccines (APVAC) in addition to standard chemotherapy. Personalization was based on the mutational landscape, transcriptome and immunopeptidome of the individual tumors. Analysis of the patients’ immune repertoire before treatment completed the dataset. GAPVAC-101 (NCT02149225) enrolled 16 patients in a European phase I feasibility, safety and immunogenicity trial integrated into standard of care. For APVAC1, up to 7 peptides were selected from a trial-specific warehouse based on individual biomarker data. Vaccination (i.d.) with GM-CSF (i.d.) and poly-ICLC (s.c.) started with the 1st adjuvant cycle of temozolomide. For APVAC2, analyses revealed between 19 and 84 somatic, non-synonymous mutations in the patients’ tumors. From the 4th TMZ cycle onwards, 11 patients received APVAC2 with usually 2 *de novo* antigens per patient (preferentially neoantigens).

**Results**

Personalized APVAC vaccines could be designed and manufactured for all patients demonstrating the feasibility of the complex personalization approach. Adverse events were largely reversible injection site reactions but also 2 anaphylactic reactions and one increase in cerebral edema. All immune evaluable patients developed at least one APVAC-specific immune response. Short, non-mutated APVAC1 antigens induced sustained CD8+ T-cell responses, in most cases with induction of a memory phenotype. 51% of vaccinated APVAC1 class I peptides were immunogenic (*ex vivo* readout). 84.7% of the mutated APVAC2 peptides induced predominantly multi-functional CD4+ T-cell responses of favorable TH1 type; 45% of APVAC2 peptides induced also CD8+ T-cell responses against nested HLA class I neoantigens. Median OS was 29 months from diagnosis in patients that received APVAC vaccination (N = 15). For one patient, a broad APVAC-specific immune response in periphery and tumor was paralleled with a favorable clinical course.

**Conclusions**

Overall, the GAPVAC approach displayed expected safety profiles and high biological activity warranting further development. This concept may be extrapolated to future developments of personalized medicines.

**Trial Registration**

NCT02149225

**Ethics Approval**

The GAPVAC-101 trial was approved by ethics committees in the following countries:- Germany: Ethikkommission der Medizinischen Fakultät Heidelberg; Zeichen: AFmo-126/2014- Netherlands: Centrale Commissie Mensgebonden Onderzoek; EC No: NL51127.000.14- Denmark: De Videnskabsetiske Komiteer Region Hovedstaden; Protocol-Nr: H-4-2014-121- Spain: Comité Ético de Investigación Clínica del Hospital Universitari Vall d’Hebron (CEIC)- Switzerland: Commission cantonale d’ethique de la recherche (CCER); No. CER 14-189

### Cellular Metabolism and Antitumor Immunity

#### O9 Endoplasmic reticulum stress-induced transcription factor C/EBP homologous protein (Chop) thwarts effector T cell activity in tumors through repression of T-bet

##### Yu Cao, PhD^1^, Yu Cao, PhD^1^, Rosa Sierra^1^, Jimena Trillo-Tinoco^1^, Carmen Anadon^1^, Wenjie Dai^1^, Eslam Mohamed^1^, Richard Klar, PhD^2^, Sven Michel^2^, Frank Jaschinski, PhD^2^, Shikhar Mehrotra, PhD^3^, Juan Cubillos-Ruiz^4^, David Munn, MD^5^, Jose Conejo-Garcia, MD, PhD^1^, Paulo Rodriguez^1^

###### ^1^H. Lee Moffitt Cancer Center & Research Institute, TAMPA, FL, USA; ^2^Secarna Pharmaceuticals GmbH & Co. KG, Planegg/Martinsried, Germany; ^3^Medical University of South Carolina, Charleston, SC, USA; ^4^Weill Cornell Medicine, New York, NY, USA; ^5^Augusta University, Augusta, GA, USA

####### **Correspondence:** Paulo Rodriguez (Paulo.Rodriguez@moffitt.org)

**Background**

The inhibited T cell function present in most patients and experimental animals with cancer represents a key obstacle in the development of promising immunotherapies. Several suppressive mechanisms including starvation of nutrients, exposure to high levels of reactive species, and acidosis impair T cell responses and are characterized by the induction of cellular stress pathways. However, the molecular targets that render tumor-infiltrating T cells dysfunctional remain practically unknown. Here, we aimed to determine the role of the C/EBP-homologous protein (Chop, encoded by Ddit3 gene), a downstream sensor of severe endoplasmic reticulum (ER) stress, in the functional regulation of tumor-exposed T cells.

**Methods**

For in vitro analysis, tumor-exposed activated T cells were collected for real-time PCR, Western Blot, Fluorescence-Activated Cell Sorting (FACS), RNA-seq, and Chromatin Immunoprecipitation assays. For T cell adoptive transfer, CD8+ T cells were isolated from control or Chop (Ddit3) null Pmel mice, stimulated with gp100 peptide, and transferred into mice bearing established B16 melanoma tumors. Next, transferred T cells were detected by FACS and the production of IFN-gamma examined through ELISpot and FACS. For in vivo tumor growth, T cell-conditional Chop null (Ddit3 T cell-KO) mice were generated and injected with s.c. tumors. In some experiments, CD8+ T cells were eliminated from tumor-bearing mice after treatment i.p. with a depleting anti-CD8 antibody every 3 days.

**Results**

Our results show that Chop is upregulated in tumor-infiltrating CD8+ T cells from tumor-bearing mice and patients with advanced ovarian carcinoma. Chop upregulation in tumor-infiltrating CD8+ T cells correlated with poor clinical outcome. The induction of Chop in tumor-exposed T cells was mediated by an increase in reactive oxygen species and a subsequent activation of the ER stress-associated kinase Perk. Deletion of Chop in CD8+ T cells enhanced effector/cytotoxic pathways, promoted significant anti-tumor effects, and overcame tumor-induced T cell tolerance. Mechanistically, Chop intrinsically repressed the transcription of the master regulator of effector T cell function, T-bet, and therefore enhanced anti-tumor effector mediators. Moreover, therapeutic inhibition of Chop in CD8+ T cells, through specific anti-sense oligonucleotides, dramatically augmented the anti-tumor effectiveness of T cell-based adoptive transfer therapies.

**Conclusions**

Our study reveals for the first time the significant regulatory role of the ER stress-driven Chop in the T cell dysfunction occurring in tumors and suggests the therapeutic potential of inhibiting Chop in T cells as a strategy to overcome tumor-induced T cell tolerance and enhance the effect of T cell-based immunotherapy.

#### O10 Lipid accumulation in the pancreatic tumor microenvironment drives metabolic exhaustion of CD8+ T cells

##### Teresa Manzo, PhD (teresa.manzo@ieo.it)

###### IEO, Milan, Italy

**Background**

The advent of immunotherapy has revolutionised cancer treatment by inducing, providing, and/or reactivating anti-tumuor T cells. Complete and durable clinical responses have been achieved in patients whose cancers were resistant to available standard treatments. Yet it has still met with limited success in most patients with solid tumours, including pancreatic ductal adenocarcinoma (PDAC). Several studies both in murine models of pancreatic cancer and PDAC patients have demonstrated that CD8+ T cells were often scarce and, if present, dysfunctional. However, our knowledge of the mechanisms that regulate their function in the context of the tumour microenvironment (TME) is still limited. We designed a study to fill this gap, aiming to enhance the clinical efficacy of immunotherapy.

**Methods**

To identify the sequence of events leading to CD8+ T cell dysfunction in the context of PDAC, first we conducted a longitudinal analysis of the T cell infiltration in a mouse model that recapitulates the progression of the human disease. Second, we employed MALDI-FT-ICR imaging mass spectrometry (IMS) to reveal the compositional changes in the regions of the murine and human TMEs infiltrated by CD8+ T cells. Thus, we profiled metabolically and transcriptionally both murine flow-sorted and patient-derived intra-pancreatic CD8+ T cells to gain mechanistic insights on their dysfunction. Finally, we performed adoptive T cell therapy in an engineered mouse model of PDAC to provide preclinical evidence that metabolic reprogramming of tumour-specific T cells might represent an effective strategy to enhance outcomes of immunotherapy in PDAC.

**Results**

We describe high levels of lipid accumulation in the TME areas of PDAC populated by infiltrating CD8+ T cells. In this lipid-rich TME, transcriptional deregulation in CD8+ T cells of pathways involved in lipid metabolism prevented engagement of fatty acid catabolism, leading to mitochondrial dysfunction and ultimately impairing their effector functions during PDAC progression. Intra-pancreatic CD8+ T cells from both murine and human tumours showed down-regulation of the very-long-chain acyl-CoA dehydrogenase (ACADVL) enzyme. Metabolic reprogramming of tumour-specific T cells through enforced expression of ACADVL enabled enhanced intra-tumoral T cell persistence in an engineered mouse model of PDAC.

**Conclusions**

CD8+ T cells penetrate into PDAC TME and persist until late stages, but eventually become functionally impaired. Our comprehensive understanding of the metabolic state of PDAC TME offers novel insights in the dynamic of PDAC immunity and we harnessed this information to generate an innovative immunotherapy strategy based on metabolic reprogramming of tumour-specific T cells.

#### O11 Tumor cell oxidative metabolism as a barrier to PD-1 blockade immunotherapy in melanoma

##### Ashley Menk, BS^1^, Yana Najjar, MD^2^, John Kirkwood, MD^2^, Cindy Sander, BS^2^, Greg Delgoffe, PhD^1^

###### ^1^University of Pittsburgh, Pittsburgh, PA, USA; ^2^UPMC Hillman Cancer Center, Pittsburgh, PA, USA

####### **Correspondence:** Greg Delgoffe (delgoffeg@upmc.edu)

**Background**

PD-1 blockade therapy has been paradigm-shifting for melanoma, but durable responses only occur in a subset of patients. While we have previously shown that tumor-infiltrating T cells have repressed metabolic machinery, the environment itself is nutrient poor due to the deregulated metabolism of tumor cells. While recent studies have suggested that T cells compete with tumor cells for glucose, our studies suggest it is the oxidative metabolism that may be limiting in tumor immunity. We hypothesize resistance to immunotherapy may be due to deficiencies in the metabolic makeup of the tumor microenvironment, and that we can infer that environment’s metabolism in patients by metabolically profiling tumor cells.

**Methods**

Melanoma patient samples were profiled by Seahorse analysis in parallel to flow cytometric analysis of tumor infiltrating lymphocytes (TIL). Murine melanoma cells were generated including RNAi constructs to specific metabolic pathways. Response to PD-1 blockade immunotherapy was monitored and lymph node or TIL T cells were tested for effector function, metabolism, and localization by flow cytometry and immunofluorescence.

**Results**

Analysis of melanoma patient biopsies showed striking heterogeneity in the metabolism of tumor cells. Patients with more metabolically active tumors have more dysfunctional TIL, while tumors that were metabolically quiescent contained T cells with superior effector function. Profiling of patient tumor cells prior to PD-1 blockade therapy revealed that patients with metabolically oxidative tumors had poorer responses while patients with quiescent tumors experienced long-term response. To confirm the role of oxidative metabolism, we generated murine melanoma lines in which glucose or oxidative metabolism was inhibited. Tumor cells in which oxidative (but not glucose) metabolism was inhibited created a less hypoxic microenvironment, had improved T cell function, and an increased response to PD-1 blockade immunotherapy.

**Conclusions**

Our data suggest that the degree of tumor hypoxia, driven through deregulated oxidative metabolism of the tumor cell, determines whether T cells have a permissive microenvironment for effective immunotherapy, and that inhibiting tumor cell oxidative metabolism may be an attractive strategy to improve the efficacy of immunotherapy.

#### O12 Chronic endoplasmic reticulum stress drives mitochondrial exhaustion of CD8 TILs

##### Jessica Thaxton, PhD, MSCR, Kiley Lawrence, Katie Hurst, Lee Leddy, Matthew Essman

###### Medical University of South Carolina, Charleston, SC, USA

####### **Correspondence:** Jessica Thaxton (Thaxton@musc.edu)

**Background**

Tumor antigen-specific T cells rapidly lose energy and effector function in tumors. The cellular mechanisms by which energy loss and inhibition of effector function occur in tumor infiltrating lymphocytes (TILs) are ill-defined. Processes upstream of the mitochondria guide cell-intrinsic energy depletion. We hypothesized that a mechanism of T cell-intrinsic energy consumption that may affect exhausted CD8+ TILs was the process of oxidative protein folding that takes place in the endoplasmic reticulum (ER) guided by protein kinase R-like endoplasmic reticulum kinase (PERK) and its downstream target ER oxidoreductase ERO1a.

**Methods**

To test our hypothesis, we created TCR transgenic mice with a T cell-specific PERK gene deletion (OT-1-Lckcre-PERKf/f, PERK KO) to determine how the PERK axis shapes T cell energetics and oxidative stress. We used proteomics, small molecule inhibitors, and quantification of PERK axis gene activation in CD8 TILs to measure how this axis impacts T cell bioenergetics.

**Results**

We found that, through PERK, ERO1a drives energy consumption and oxidative stress in T cells. Proteomics analysis revealed that ERO1a induced a protein profile in T cells associated with increased translation, energy production and consumption, and extraction of misfolded proteins. We identified a biomarker of PERK-ERO1a-mediated metabolic exhaustion and oxidative stress in T cells as mitochondrial reactive oxygen species (mtROS), and we found that PD-1+ CD8+ TILs express mtROS and high levels of ERO1a. In vivo treatment with a PERK inhibitor abrogated mtROS and boosted viability and effector function of PD-1+ CD8 TILs. Combination therapy was effective compared to control conditions in a sarcoma mouse model.

**Conclusions**

Our data identify the ER as a regulator of T cell energetics and indicate that ER elements are effective targets to improve cancer immunotherapy.

#### O13 Lactic acid metabolically supports the high suppressive function of tumor infiltrating regulatory T cells

##### McLane Watson, BS^1^, McLane Watson, BS^1^, McLane Watson, BS^1^, Paolo Vignali, BA^1^, Ryan Whetstone, MS, PhD^1^, Ronal Peralta^1^, Rahul Deshpande, PhD^1^, Ashley Menk, BS^1^, Nicole Scharping, BS^1^, Brett Morrison, MD, PhD^2^, Stacy Wendell, PhD^1^, Greg Delgoffe, PhD^1^

###### ^1^University Of Pittsburgh, Pittsburgh, PA, USA; ^2^Johns Hopkins, Baltimore, MD

####### **Correspondence:** Greg Delgoffe (delgoffeg@upmc.edu)

**Background**

Cancer immunotherapy fails for a majority of patients due a number of resistance mechanisms including the recruitment, activation, and differentiation of regulatory T cells (Treg). While metabolically harsh conditions in the tumor microenvironment (TME) starve infiltrating effector T cells, it has been shown that Treg cells have a distinct metabolic profile potentially providing them metabolic flexibility to utilize metabolites rich in the TME. Therefore, we hypothesized that the TME is metabolically supporting Treg cells.

**Methods**

Methods: B16, a mouse model of melanoma, was used. From tumor bearing mice conventional effector T cells and Treg cells were transcriptionally and metabolically profiled using flow cytometry, proliferation assays, suppression assays, and isotopic flux analysis. A mouse with a Treg specific deletion of MCT1, a predominant lactate transporter, was generated. A pharmacological inhibitor of MCT1 was used to prevent lactate uptake.

**Results**

Results: In vitro, Treg cells conditioned in no or low glucose media were superior suppressors to those conditioned in high glucose. Similarly, sorting Treg cells based on glucose uptake revealed low glucose Treg cells to be superior suppressors. Transcriptional and metabolic profiling revealed intratumoral Treg cells upregulate a distinct metabolic profile utilizing the glycolytic end-product, lactic acid. Lactic acid has been known to be immunosuppressive, and indeed, it curbed the function of conventional effector cells in vitro. However, lactic acid had no effect on Treg cell function. Isotopic flux analysis revealed lactate is utilized by Treg cells to generate glycolytic intermediates, in part through gluconeogenic pathways. Inhibition of gluconeogenesis in vivo resulted in decreased proliferation of tumor infiltrating Treg cells. Preventing lactate transport in Treg cells through a conditional knockout of MCT1 resulted in mice with normal immune homeostasis, but superior anti-tumor immunity when implanted with melanoma. This coincided with increased IFN-γ production by intratumoral CD8, Treg, and Tconv cells leading to dramatically slowed tumor growth.

**Conclusions**

Conclusions: These data suggest that lactic acid supports Treg cells in the TME and that targeting lactate metabolism to weaken Treg cells may increase efficacy of cancer immunotherapy.

#### O14 Reinvigorating TILs by hyper-oxygenation

##### Mateusz Rytelewski, PhD^2^, Karine Haryutyunan^2^, Felix Nwajei, PhD^2^, Sergei Vinogradov, PhD^3^, Marina Konopleva, MD, PhD^2^, Tomasz Zal, PhD^2^, Anna Zal^2^

###### ^1^University of Texas MD Anderson Cancer C, Houston, TX, USA; ^2^MD Anderson Cancer Center, Houston, TX, USA; ^3^University of Pennsylvania, Philadelphia, PA, USA

####### **Correspondence:** Tomasz Zal (tzal@mdanderson.org)

**Background**

Active migration of lymphocytes within tumors is pre-requisite to immune therapies. Both solid tumors as well as bone marrow malignancies develop regions of low oxygen partial pressures, i.e. below 5 mmHg, known as tumor hypoxia. Tumor hypoxia plays multiple roles in tumor immune suppression including tissue expression of checkpoint molecules, decreased antigen presentation and deactivation of various tumor-infiltrating lymphocytes (TILs). However, the relationship of intratumoral oxygen distribution to the motility of TILs remains undefined, largely owing to the lack of a suitable method for contextual imaging of oxygen gradients and cell dynamics in vivo.

**Methods**

Using PtP-C343 oxygen probe, we developed a regimen of intravital 2-photon microscopy that combines TIL motility recording with oxygen imaging based on phosphorescence lifetimes. We applied this method, termed 2-photon pre-pulse phosphorescence lifetime imaging microscopy (2PreP-PLIM) to relate the dynamic behavior of T cells to the local oxygen gradients that develop inside solid lung tumors and leukemic bone marrow in pre-clinical mouse models.

**Results**

We found that tumor infiltrating T-lymphocytes traversed regions of varying oxygen concentrations, including regions of hypoxia that developed within the solid tumor cores and in bone marrow with advanced-stage B-cell acute lymphocytic leukemia (B-ALL). T cell motilities were markedly decreased in hypoxic regions compared to the neighboring normoxia, and many of the TILs experiencing hypoxia appeared stalled. Remarkably, breathing 100% oxygen, which alleviated hypoxia inside solid lung tumors, rapidly increased the migratory behavior of otherwise stalled T cells.

**Conclusions**

The devised technique for intravital oxygen and cell dynamics co-imaging reveals a role of oxygen supply in promoting intratumoral T cell migration. Our results reveal reinvigoration of TIL motility as a new mechanistic benefit of oxygen and, possibly, other hypoxia countermeasures in counteracting tumor immune suppression.

**Acknowledgements**

This work is supported by grants NCI R01CA155056 and CPRIT MIRA 160683 to TZ and MK and grants EB018464 and NS092986 from the National Institutes of Health (USA) to SV. MR is the recipient of a post-doctoral fellowship from the Canadian Institutes of Health Research (CIHR).

**Ethics Approval**

The study was approved by the University of Texas MD Anderson Cancer Center Institutional Animal Care and Use Committee under protocol number 00000878-RN01.

### Cellular Therapy Approaches

#### O15 Engineering adoptive T cell therapy to co-opt Fas ligand-mediated death signaling in solid tumors

##### Kristin Anderson, PhD^1^, Shannon Oda^1^, Breanna Bates, BS^1^, Madison Burnett^1^, Edison Chiu, BS^2^, Magdalia Suarez Gutierrez^1^, Nicolas Garcia, BS^1^, Andrew Daman^1^, Philip Greenberg, MD^1^

###### ^1^Fred Hutchinson Cancer Research Center, Seattle, WA, USA; ^2^University of Washington, Seattle, WA, USA

####### **Correspondence:** Philip Greenberg (pgreenberg@fredhutch.org)

**Background**

Over 20,000 women are diagnosed with ovarian cancer annually, and over half will die within 5 years. This rate has changed little in the last 20 years, highlighting the need for therapy innovation [1]. One especially promising new strategy employs immune T cells engineered to target proteins uniquely overexpressed in tumors, with the potential to limit tumor growth without toxicity to healthy tissues. Mesothelin (Msln) is a rational target for ovarian cancer immunotherapy [2] - it contributes to the malignant and invasive phenotype in these tumors and has limited expression in healthy cells [3,4,5].

**Methods**

Deep transcriptome profiling of whole tumor tissue was used to confirm the expression of similar gene signatures in human cancers and in the preclinical ID8 mouse model, including comparable expression of immunosuppressive pathways. For example, RNA sequencing, flow cytometry and immunohistochemistry analysis revealed consistently high expression of the immunomodulatory protein Fas ligand (FasL). Human/mouse T cells were engineered to express a human/mouse Msln-specific high-affinity T cell receptor (TCR-Msln)[6] and tested for cytotoxic activity against human patient-derived or ID8 mouse ovarian cancer cell lines *in vitro* and *in vivo*.

**Results**

In a disseminated ID8 tumor model, adoptively transferred TCR-Msln T cells preferentially accumulated within established tumors, delayed ovarian tumor growth, and significantly prolonged mouse survival. However, our data also revealed that elements in the tumor microenvironment (TME) limit engineered T cell persistence and anti-cancer activity. We and others previously detected FasL in the tumor vasculature [7] and TME of human and murine ovarian cancers. FasL can induce apoptosis in infiltrating lymphocytes expressing Fas receptor (Fas)[8]. To overcome this potential T cell evasion mechanism, we generated a panel of immunomodulatory fusion proteins (IFP) containing the Fas extracellular binding domain fused to a CD28 or 4-1BB co-stimulatory domain, rather than the natural death domain. Relative to T cells modified with only TCR-Msln, T cells engineered to express both TCR-Msln and a Fas IFP preferentially infiltrate tumors, expand/persist and retain function in the TME of tumor-bearing mice. Moreover, adoptive immunotherapy with IFP+ T cells significantly prolonged survival in tumor-bearing mice, relative to TCR-Msln T cells lacking an IFP.

**Conclusions**

Fas/FasL signaling can mediate T cell death, including activation-induced cell death, an apoptotic mechanism responsible for regulating T cell expansion. Thus, tumor cells may upregulate FasL for protection from tumor-infiltrating lymphocytes. As many solid tumors overexpress FasL, IFPs may provide an opportunity to enhance engineered adoptive T cell therapy against many malignancies.

**Acknowledgements**

The authors thank Matthias Stephan for the ID8-VEGF cell line, Sunni Farley, Robert Pierce, Savanh Chanthaphavong, Brian Johnson and Megan Larmore for histopathology assistance, Ingunn Stromnes and Tom Schmitt for MSLN TCR development, and the Fred Hutchinson Cancer Research Center Flow Cytometry Core for technical support.

**References**

1. Vaughan S, Coward JI, Bast RC, Berchuck A, Berek JS, Brenton JD, Coukos G, Crum CC, Drapkin R, Etemadmoghadam D, Friedlander M, Gabra H, Kaye SB, Lord CJ, Lengyel E, Levine DA, McNeish IA, Menon U, Mills GB, Nephew KP, Oza AM, Sood AK, Stronach EA, Walczak H, Bowtell DD, Balkwill FR. Rethinking ovarian cancer: recommendations for improving outcomes. Nat. Rev. Cancer. 2011;11(10):719–725.

2. Cheever MA, Allison JP, Ferris AS, Finn OJ, Hastings BM, Hecht TT, Mellman I, Prindiville SA, Viner JL, Weiner LM, Matrisian LM. The Prioritization of Cancer Antigens: A National Cancer Institute Pilot Project for the Acceleration of Translational Research. Clin. Cancer Res. 2009;15:5323–5337.

3. Chang K, Pastan I, and Willingham MC. Isolation and characterization of a monoclonal antibody, K1, reactive with ovarian cancers and normal mesothelium. Int. J. Cancer. 1992;50: 373–381.

4. Chang K, and Pastan I. Molecular cloning of mesothelin, a differentiation antigen present on mesothelium, mesotheliomas, and ovarian cancers. Proc. Natl. Acad. Sci. USA. 1996;93:136–140.

5. Gubbels JAA, Belisle J, Onda M, Rancourt C, Migneault M, Ho M, Bera TK, Connor J, Sathyanarayana BK, Lee B, Pastan I, Patankar MS. Mesothelin-MUC16 binding is a high affinity, N-glycan dependent interaction that facilitates peritoneal metastasis of ovarian tumors. Mol. Cancer. 2006;5:50.

6. Stromnes IM, Schmitt TM, Hulbert A, Brockenbrough JS, Nguyen HN, Cuevas C, Dotson AM, Tan X, Hotes JL, Greenberg PD, Hingorani SR. T Cells Engineered against a Native Antigen Can Surmount Immunologic and Physical Barriers to Treat Pancreatic Ductal Adenocarcinoma. Cancer Cell. 2015;28:638–652.

7. Motz GT, Santoro SP, Wang LP, Garrabrant T, Lastra RR, Hagemann IS, Lal P, Feldman MD, Benencia F, Coukos G. Tumor endothelium FasL establishes a selective immune barrier promoting tolerance in tumors. Nat. Med. 2014;20(6):607-615.

8. Waring P, and Mullbacher A. Cell death induced by the Fas/Fas ligand pathway and its role in pathology. Immunology and Cell Biology. 1999;77:312-317.

**Ethics Approval**

The Institutional Animal Care and Use Committees of the University of Washington and the Fred Hutchinson Cancer Research Center approved all animal studies.

#### O16 Tscm-like CD8+ T-cells are associated with adoptive TIL therapy response and survival

##### Matthew Beatty, PhD, Benjamin Schachner, Autumn Joerger, Ellen Scott, Patrick Verdugo, John Mullinax, MD, Amod Sarnaik, MD, Shari Pilon-Thomas, PhD

###### Moffitt Cancer Center, Tampa, FL, USA

####### **Correspondence:** Shari Pilon-Thomas (shari.pilon-thomas@moffitt.org)

**Background**

Adoptive Cell Transfer (ACT) using Tumor Infiltrating Lymphocytes (TIL) for unresectable metastatic melanoma results in a median progression free survival of 6 months and median overall survival of 33 months at our institution. The purpose of this study is to analyze the phenotype and function of patient infused TIL and identify TIL phenotypes associated with response and progression free survival.

**Methods**

Patient-derived and infused TIL samples were phenotyped for composition of CD4+ and CD8+ T-cells, expression of co-stimulatory and co-inhibitory markers (4-1BB, PD-1, OX-40, BTLA, LAG3, TIM3, TIGIT), and Tregs (CD4+ CD25hi CD127-). Additionally, T-cell memory markers were used to analyze the proportion of naïve (CD45RA+ CCR7+ CD62L+ CD95-), T stem cell memory (Tscm) (CD45RA+ CCR7+ CD62L+ CD95+), central memory (CD45RA- CCR7+ CD62L+ CD95+), effector memory (CD45RA- CCR7- CCR7- CD62L- CD95+), and terminal effector (CD45RA+ CCR7- CD62L- CD95+). Samples was co-cultured with HLA-matched melanoma cell lines and analyzed for IFNγ production.

**Results**

Forty one infused TIL samples were analyzed. We observed an increased CD8+:CD4+ T-cell ratio in patients who were partial or complete responders (responders) compared to stable disease and progressive disease (non-responders) by RECIST criteria. No differences in expression of co-stimulatory or co-inhibitory markers on CD8+ or CD4+ T-cells were found. An overall increase in Treg percentage was found in non-responders, though there was no increase in Tregs as a percentage of CD4+ T-cells. Among memory T-cell populations, responders had enrichment for CD8+ Tscm cells as a percentage of total TIL compared to non-responders. Additionally, patients with greater than 12% CD8+ Tscm in the TIL product have not met their median 5 year progression free survival compared to 6 months for patients with 12% of less CD8+ Tscm. Of 37 samples, 20 were reactive in an MHC class I dependent manner against at least one HLA-matched melanoma cell line. Additionally, reactivity was associated with an increased progression free survival of 35 months compared to 5 months for non-reactive samples.

**Conclusions**

In this study we show that CD8+ Tscm T-cells within the ACT TIL therapy product as well as reactivity to HLA-matched cell lines is associated with patient response to therapy as well as progression free survival. Tscm cells have been of great interest in cellular therapies due to their increased persistence. These data form the rationale for enriching CD8+ Tscm cells in TIL ACT products and could lead to better clinical outcomes.

#### O17 Infusion of TGFβ-resistant EBV-specific T-cells post cytoreductive chemotherapy is safe and associated with clinical benefit in patients with recurrent/metastatic NPC

##### Christopher DeRenzo, MD^1^, Mamta Kalra^2^, Carlos Ramos^2^, Catherine Robertson^2^, Huimin Zhang^2^, Claudia Gerken^2^, Olga Dakhova^2^, Melinda Mata^2^, Meng-Fen Wu^2^, Hao Liu^2^, Catherine Bollard^2^, Zhuyong Mei^2^, Adrian Gee^2^, Bambi Grilley^2^, Gianpietro Dotti, MD^2^, Helen Heslop, MD^2^, Malcolm Brenner^2^, Cliona Rooney^2^, Stephen Gottschalk^1^

###### ^1^Center for Cell and Gene Therapy (Houston, TX); St. Jude Children's Research Hospital (Memphis, TN), Memphis, TN, USA; ^2^Center for Cell and Gene Therapy (Houston, TX), Houston, TX, USA

####### **Correspondence:** Stephen Gottschalk (Stephen.Gottschalk@STJUDE.ORG)

**Background**

Outcomes for patients with recurrent/metastatic Epstein-Barr virus positive (EBV+) nasopharyngeal carcinoma (NPC) are poor and treatment options are limited. Previously, systemic administration of up to 3x10^8/m2 autologous EBV-specific T-cells was safe, however antitumor activity was limited. Lack of efficacy was likely due to i) limited specificity of the infused T-cell products to EBV antigens expressed in NPC, ii) lack of T-cell expansion, and iii) the immunosuppressive tumor microenvironment. To address these limitations, we developed a product enriched in LMP1-, LMP2-, EBNA1-, and BARF1-specific T-cells. T-cells were also modified to resist TGFβ mediated immunosuppression by introducing a dominant-negative TGFβ receptor (DNR.NPC T-cells). We evaluated the safety and antitumor activity of these DNR.NPC T-cells in patients with recurrent/metastatic NPC.

**Methods**

In a phase 1 trial, NCT02065362, we administered 0.4-1x10^8/m2 autologous DNR.NPC T-cells to patients with recurrent/metastatic EBV+ NPC with or without cytoreductive chemotherapy. Post-infusion we evaluated safety, T-cell expansion, and antitumor activity.

**Results**

Four patients received two doses of 2x10^7/m2 DNR.NPC T-cells 14 days apart. Without cytoreduction, T-cell expansion was limited as judged by DNR-qPCR (mean peak: 80.2 copies/μg DNA; range: 10.4-133.6 copies/μg DNA). The protocol was therefore modified to include cytoreductive chemotherapy with cyclophosphamide (Cy; 500mg/m2/day) and fludarabine (Flu; 30mg/m2/day) on days -4 to -2 prior to T-cell infusion. Cy/Flu and T-cell infusions were well tolerated without dose limiting toxicities in 4 patients who received either 4x10^7 cells/m2 (n=2) or 1x10^8 cells/m2 (n=2). There was a 128-fold greater DNR.NPC T-cell expansion (p<0.05) in patients who received cytoreduction compared to those who did not. T-cell expansion occurred at a median 7 days post infusion (range: 4-7 days) with a peak DNR-qPCR level of 10,305 copies/μg DNA (range: 1,016-31,765 copies/μg DNA). Antitumor activity was assessed 6 weeks post T-cell infusion. Without cytoreduction, 2/4 patients had progressive disease, 1 had stable disease (SD), and 1 was not evaluable. Of the 4 patients who received cytoreduction followed by T-cells, 3/4 had SD, and 1, who had imaging findings of unknown significance (recovering marrow vs metastatic disease), had a complete response. With a median follow up of 19.5 months (range: 18.2-27.7 months), 3/3 patients who received T-cells only died from disease progression. In contrast, 4/4 patients who received T-cells preceded by cytoreduction are alive; 2 with SD, and 2 in remission.

**Conclusions**

Infusion of DNR.NPC-specific T-cells after cytoreductive chemotherapy is safe, results in robust T-cell expansion, and is associated with objective clinical benefit in patients with recurrent/metastatic NPC.

**Ethics Approval**

This study (ClinicalTrials.gov identifier: NCT02065362) was approved by the institutional review board at Baylor College of Medicine (Houston, TX) and by the US Food and Drug Administration.

#### O18 Preliminary clinical data from a pilot study of NY-ESO-1c259T-cells in advanced myxoid/round cell liposarcoma

##### Sandra D'Angelo^2^, Justina Stadanlick^1^, Mihaela Druta^3^, David Liebner, MD^4^, Scott Schuetze^5^, Brian Van Tine^6^, William Tap^2^, Cedrik Britten, MD^7^, Karen Chagin, MD^1^, Aisha Hasan, MBBS MD^7^, Elliot Norry, MD^1^, Trupti Trivedi, MS^1^

###### ^1^Adaptimmune, Philadelphia, PA, USA; ^2^Memorial Sloan Kettering Cancer Center &, New York, NY, USA; ^3^Moffit Cancer Center, Tampa, FL, USA; ^4^Ohio State University Medical Center, Columbus, OH, USA; ^5^University of Michigan, Ann Abror, USA; ^6^Washington University St.Louis, St. Louis, USA; ^7^GlaxoSmithKline, Stevenage, UK

####### **Correspondence:** Sandra D'Angelo (rafael.amado@adaptimmune.com)

**Background**

Metastatic myxoid/round cell liposarcoma (MRCLS) has a poor prognosis. NY-ESO-1 is expressed in 80-90% of MRCLS tumors, making it an attractive target for adoptive T-cell immunotherapy. This study evaluates affinity enhanced autologous NY-ESO-1c259T-cells (SPEAR T-cells) recognizing an NY-ESO-1-derived peptide complexed with HLA-A*02 in MRCLS (NCT02992743).

**Methods**

This open label phase I/II single arm pilot study evaluates efficacy, safety, and translational research endpoints. The protocol was approved by each center’s Institutional Review Board, and all patients signed informed consent forms. Eligible patients are ≥ 18 years old; HLA-A*02:01+, *02:05+ or *02:06+; have advanced MRCLS expressing NY-ESO-1 at 2+/3+ intensity in ≥30% of tumor cells by immunohistochemistry; have measurable disease; received anthracycline therapy; ECOG status 0 or 1; and have adequate organ function. Following apheresis, T-cells are isolated, expanded, transduced with a lentiviral vector containing the NY-ESO-1c259TCR, and 1– 8 × 109 transduced T-cells are infused on day 1 after lymphodepletion with fludarabine 30 μg/m2/d and cyclophosphamide 600 μg/m2/d on d -7 to -5. Response is assessed at 4, 8, 12, and 24 weeks and then every 3 months until disease progression.

**Results**

Thirty-nine patients were screened: 20 had the requisite HLAs, and 17 of these had NY-ESO-1 present at required levels. Thirteen patients were enrolled, and 10 received the TCR therapy between 04 October 2017 and 27 June 2018 (as of 12 July 2018). The patients received 1.0-5.7 × 109 transduced T-cells. Two patients were recently treated, and tumor lesion assessment at week 8 is still pending. There are post-infusion tumor lesion assessments for 8 patients: 3 females, 5 males, with a median age of 48, and median 4 lines of prior systemic therapies. At the time of the data cut-off, 4 of the 8 patients (50%) have achieved a confirmed partial response (PR) and 50% have stable disease (SD) as the best overall response. The duration of responses varies from 4 weeks to greater than 5 months; 2 of the patients have ongoing PRs as of 12 July 2018. AEs ≥ grade 3 in these 8 patients include lymphopenia (6), neutropenia (5), leukopenia (5), thrombocytopenia (3), hypophosphatemia (2), anemia (1), cytokine release syndrome (1; SAE), pyrexia (1) and leukocytosis (1). None required study discontinuation.

**Conclusions**

These preliminary data indicate that treatment with NY-ESO-1c259T-cells in MRCLS appears to have an acceptable safety profile consistent with other NY-ESO-1c259T-cell studies, with potential for anti-tumor effects. We will report updated results.

**Trial Registration**

NCT02992743

### Clinical Trials (Completed)

#### O19 Phase 1 study using mogamulizumab (KW-0761) to deplete regulatory T cells in combination with checkpoint inhibitors durvalumab (MEDI4736) or tremelimumab in subjects with advanced solid tumors

##### Dmitriy Zamarin, MD, PhD^2^, Omid Hamid, MD^3^, Asha Nayak, MD^4^, Solmaz Sahebjam, MD^5^, Mario Sznol, MD^6^, Agron Collaku, PhD^1^, Floyd Fox, PhD^1^, Margaret Marshall, MD^1^, David Hong, MD^7^

###### ^1^Kyowa Kirin Pharmaceutical Development, Inc., Princeton, NJ, USA; ^2^Memorial Sloan-Kettering Cancer Center, New York, NY, USA; ^3^The Angeles Clinic and Research Institute, Los Angeles, CA, USA; ^4^Georgia Cancer Center, MARTINEZ, GA, USA; ^5^H. Lee Moffitt Cancer Center, Tampa, FL, USA; ^6^Yale Cancer Center, New Haven, CT, USA; ^7^MD Anderson Cancer Center, Houston, TX, USA

####### **Correspondence:** Dmitriy Zamarin (zamarind@mskcc.org)

**Background**

Regulatory T cells (Tregs) play a pivotal role in maintaining immunological tolerance, which can inhibit antitumor immune responses and may mediate resistance to immunomodulatory therapy targeting CTLA-4 or PD-1/PD-L1. Mogamulizumab (Moga) is a humanized IgG1 monoclonal antibody (mAb) targeting anti-CC-chemokine receptor 4 (CCR4), which is highly expressed on Tregs. This study sought to evaluate whether Treg depletion by Moga enhanced antitumor response in combination with PD L1 blockade by Durvalumab (Durva) or CTLA-4 blockade by Tremelimumab (Treme).

**Methods**

Study 0761 012 was a multicenter, Phase 1, open-label, dose escalation/cohort expansion study of Moga in combination with either Durva (Treatment A; TrA) or Treme (Treatment B; TrB) in adult subjects with advanced solid tumors. Dose escalation (Part 1) included 4 dose cohorts for each combination (Table 1). There was one tumor-specific expansion cohort (Part 2). The primary objective was to assess safety and tolerability; the secondary objectives were to evaluate antitumor effect (RECIST 1.1 and irRECIST 1.1), pharmacokinetics, and immunogenicity. Exploratory objectives were to examine biomarkers/pharmacodynamics.

**Results**

A total of 64 subjects were enrolled and treated: n=40 in Part 1 (Table 1) and n=24 in Part 2. Dose escalations were completed in Part 1 without any dose-limiting toxicities, and combinations of 1 mg/kg Moga with 10 mg/kg of either Durva (TrA) or Treme (TrB) were used to treat an expansion cohort with pancreatic cancer in Part 2. Treatment-emergent adverse event (TEAE) rates and most common TEAEs for each treatment combination are shown in Table 2 (Table 2). Moga showed pharmacologic activity by reduction in number of peripheral blood CCR4+ effector Tregs (eTregs) in both TrA and TrB; however, other important effector cell types were also depleted. One TrA subject with ASPS and one TrB subject with prostate cancer had partial responses, with a duration of response of 10.6 months and 3.7 months, respectively. Five (26.3%) TrA subjects and 7 (36.8%) TrB subjects showed disease stability. No subjects with pancreatic cancer responded. There was no correlation between the degree of peripheral blood eTreg depletion and response.

**Conclusions**

Combining Moga with either Durva or Treme in solid tumors was tolerable and effectively decreased eTregs in peripheral blood. There was no efficacy beyond what was anticipated from the individual monotherapy with Durva or Treme. Concomitant depletion of non eTreg effector T cell populations may have been responsible for the apparent lack of therapeutic enhancement.

**Trial Registration**

NCT02301130

**Ethics Approval**

This study was approved by an Institutional Review Board at each investigative site.

**Table 1 (abstract O19).**
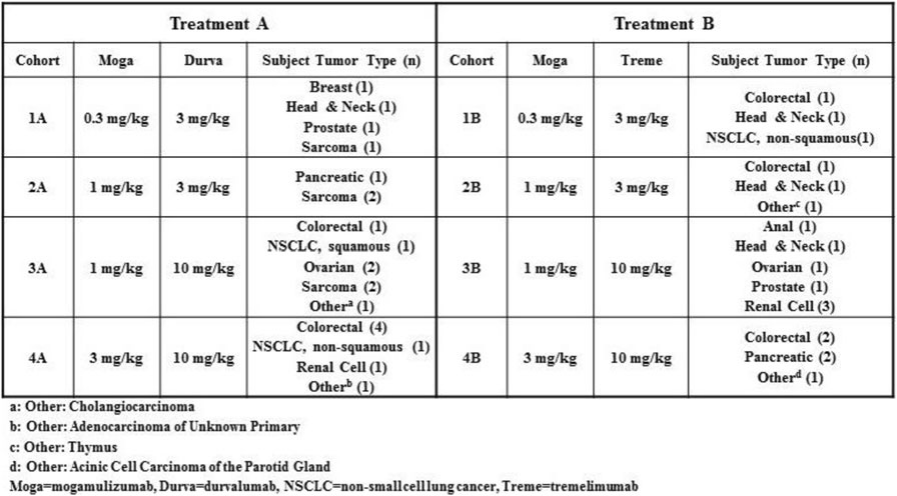
See text for description

**Table 2 (abstract O19).**
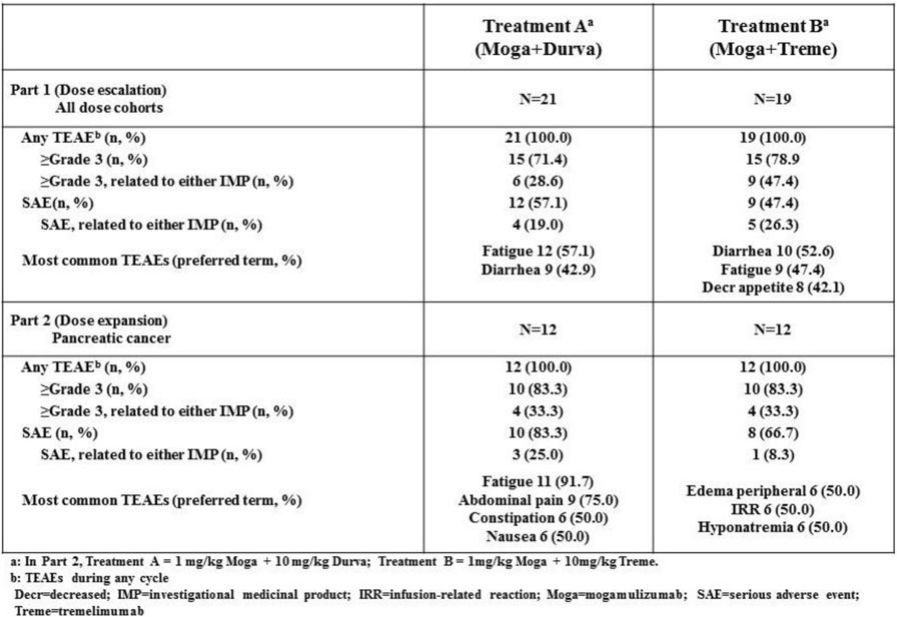
See text for description

### Clinical Trials (In Progress)

#### O20 Reprogramming suppressive myeloid cells in tumor microenvironment with pepinemab, first-in-class Semaphorin 4D Mab, enhances combination immunotherapy

##### Terrence Fisher, PhD^2^, John Leonard, MD PhD^1^, Crystal Mallow, BS^1^, Holm Bussler, PhD^1^, Sebold Torno, BS^1^, Maria Scrivens^2^, Alan Howell, MS^2^, Leslie Balch, BS^2^, Clint Allen, MD^3^, Paul Clavijo, PhD^3^, Gregory Lesinski, PhD, MPH^4^, Christina Wu, MD^4^, Brian Olson, PhD^4^, Siwen Hu-Lieskovan, MD, PhD^5^, Antoni Ribas, MD, PhD^5^, Emily Greengard, MD^6^, Ernest Smith, PhD^2^, Maurice Zauderer, PhD^2^, Elizabeth Evans, PhD^1^, Elizabeth Evans, PhD^1^

###### ^1^Vaccinex, Rochester, NY, USA; ^2^Vaccinex Inc, Rochester, NY, USA; ^3^NIH/NIDCD Head and Neck Surgery Branch, Bethesda, MD, USA; ^4^Winship Cancer Institute of Emory Univer, Atlanta, GA, USA; ^5^David Geffen School of Medicine at UCLA, Los Angeles, CA, USA; ^6^University of Minnesota Masonic Children, Minneapolis, MN, USA

####### **Correspondence:** Elizabeth Evans (eevans@vaccinex.com)

**Background**

Tumor growth inhibition by anti-semaphorin 4D (SEMA4D, CD100) blocking antibody is enhanced when combined with various immunotherapies in preclinical animal models. Immune checkpoint combinations with pepinemab (VX15/2503), humanized anti-SEMA4D antibody, are currently being evaluated in several clinical trials.

**Methods**

Mechanistic studies in syngeneic preclinical models investigated the effects of SEMA4D blockade on anti-tumor activity and immune responses, both as a single agent and in combination with various immunotherapy agents. Pepinemab (VX15/2503) is currently being evaluated as single agent or in combination with other immunotherapies in four clinical trials: (i) a Phase 1b/2a combination trial of pepinemab with avelumab in NSCLC (CLASSICAL-Lung) (NCT03268057); (ii) a phase 1 combination trial of pepinemab with nivolumab or ipilimumab in melanoma patients who have progressed on any anti-PD-1/PD-L1 (NCT03425461); (iii) a neoadjuvant integrated biomarker trial in patients with metastatic colorectal and pancreatic cancers treated with pepinemab in combination with nivolumab or ipilimumab (NCT03373188); and (iv) a Phase 1/2 trial of pepinemab in children with solid tumors and children and young adults with osteosarcoma (NCT03320330).

**Results**

SEMA4D exerts multi-faceted effects within the tumor microenvironment by creating a barrier at the tumor-stroma margin to restrict immune cell infiltration and promoting immunosuppressive activity of myeloid-derived cells. Blocking antibody to SEMA4D directly enhanced M1/M2 ratio, and both reduced expression of chemokines that recruit MDSC and the ability of MDSC to suppress T cell proliferation. In preclinical models, anti-SEMA4D reduced the function of MDSC and Treg in the TME while simultaneously restoring the ability of dendritic cells and cytotoxic T cells to migrate into the tumor. Importantly, anti-SEMA4D MAb enhanced the activity of co-administered immunotherapies in murine colon, head and neck (HNSCC), and melanoma models. For example, anti-SEMA4D plus anti-CTLA-4 resulted in 90% complete tumor rejection (CR) (p<0.0001) in an HNSCC model representative of a T cell inflamed tumor with high MDSC suppression. Combination treatment of anti-SEMA4D with anti-LAG3 or epigenetic modulator entinostat resulted in maximal tumor growth delay and 90% CR (p<0.0001).Pepinemab treatment was well tolerated in a Phase I trial in patients with advanced refractory solid tumors (NCT01313065). Several clinical trials are in progress to evaluate safety, tolerability, efficacy, and biological endpoints, including immunophenotyping tumors and blood of patients treated with pepinemab in combination with immunomodulatory agents. Trial design will be reported and enrollment updates are expected.

**Conclusions**

SEMA4D blockade represents a novel approach to promote functional immune infiltration into the tumor, reduce mesenchymal suppression, and enhance immunotherapy effects.

**Trial Registration**

NCT01313065NCT03268057NCT03425461NCT03373188NCT03320330

**References**

1. Evans, EE et al. Cancer Immunol Res. 2015 Jun;3(6):689-701.

2. Patnaik A et al. Clin Cancer Res. 2016 Feb 15;22(4):827-363. Adusumilli PS et al. J Immunother Cancer. 2016 Jun 20; 5:50.

#### O21 A phase 1 study of TSR-022, an anti-TIM-3 monoclonal antibody, in combination with TSR-042 (anti-PD-1) in patients with colorectal cancer and post-PD-1 NSCLC and melanoma

##### Diwakar Davar, MD^1^, Peter Boasberg, MD, FACP^2^, Zeynep Eroglu, MD^3^, Gerald Falchook, MD^4^, Justin Gainor, MD^5^, Erika Hamilton, MD^6^, J. Randolph Hecht, MD^7^, Jason Luke, MD, FACP^8^, Michael Pishvaian^9^, Antoni Ribas, MD, PhD^7^, Judy Wang, MD^10^, Kristen McEachern, PhD^11^, Angela Waszak^11^, Sharon Lu^11^, Yong Li^11^, Ying Wang^11^, Patricia LoRusso, DO^12^

###### ^1^University of Pittsburgh Department of Medicine, Pittsburgh, PA, USA; ^2^The Angeles Clinic and Research Institute, Santa Monica, CA, USA; ^3^Moffitt Cancer Center, Tampa, FL, USA; ^4^Sarah Cannon Research Institute at HealthONE, Denver, CO, USA; ^5^Massachusetts General Hospital, Boston, MA, USA; ^6^Sarah Cannon Research Institute at Tennessee Oncology, Nashville, TN, USA; ^7^Ronald Reagan UCLA Medical Center, Los Angeles, CA, USA; ^8^The University of Chicago Medicine, Chicago, IL, USA; ^9^MedStar Georgetown University Hospital, Washington, DC, USA; ^10^Sarah Cannon Research Institute, Sarasota, FL, USA; ^11^TESARO, Inc., Waltham, MA, USA; ^12^Yale Cancer Center, New Haven, CT, USA

####### **Correspondence:** Diwakar Davar (davard@upmc.edu)

**Background**

T cell immunoglobulin and mucin-domain containing-3 (TIM-3) is a key immune checkpoint that is often co-expressed with programmed cell death protein (PD)-1 and has been implicated in both effector T cell exhaustion and immune suppression mediated by myeloid cells. TIM-3 expression on effector T cells is associated with reduced cell proliferation and cytokine production, while TIM-3 expression on dendritic and other myeloid cells may prevent recruitment and priming of T cells. In combination with PD-1 blockade, anti-TIM-3 enhances the activation of T cells and demonstrates greater anti-tumor activity than anti-PD-1 alone in preclinical models. TSR-022 is a potent, selective anti-TIM-3 antibody that is being developed in combination with PD-1 blockade.

**Methods**

TSR-022 is being investigated in a multicenter, open-label, first-in-human phase 1 trial that is enrolling patients with advanced or metastatic solid tumors that have progressed after treatment with available therapies or are intolerant to standard treatment. Part 1 includes an evaluation of dose escalation of TSR-022 as a monotherapy, as well as in combination with TSR-042, an anti-PD-1 antibody, also including immuno-oncology–naïve patients. Patients received IV infusion of TSR-022 alone and in combination with 500 mg TSR-042 in escalating, fixed doses. The primary objective of the part 1 study is to determine the recommended phase 2 dose (RP2D) and to evaluate the safety and tolerability of TSR-022 alone and in combination with TSR-042. In part 2 of the study, the anti-tumor activity of TSR-022 in combination with TSR-042 is being evaluated in colorectal cancer, post-PD-1 melanoma, and post-PD-1 NSCLC.

**Results**

As of July 13, 2018, 98 patients have been treated in part 1 of this study and 104 patients have been treated in part 2. Of these 202 patients, 52 received TSR-022 monotherapy and 150 received combination therapy of TSR-022 and TSR-042. The incidence of grade ≥3 treatment-related adverse events in combination therapy was 6.7%. The grade ≥3 treatment-related adverse events in combination therapy that occurred in >1.0% of patients were lipase increased (1.3%) and rash maculo-papular (1.3%). There was a dose-proportional increase in TSR-022 exposure and receptor occupancy. Objective responses were observed in patients with post-PD1 NSCLC and melanoma and will be reported.

**Conclusions**

TSR-022 in combination with TSR-042 was well tolerated across multiple dose levels. Adverse events were manageable and consistent with the safety profiles of other checkpoint inhibitors. Clinical activity was observed and warrants continued exploration.

**Trial Registration**

clinicaltrials.gov NCT02817633

#### O22 Safety and efficacy of cryopreserved autologous tumor infiltrating lymphocyte therapy (LN-144, lifileucel) in advanced metastatic melanoma patients following progression on checkpoint inhibitors

##### Amod Sarnaik, MD^1^, Sajeve Thomas^2^, Diwakar Davar, MD^3^, John Kirkwood, MD^4^, Harriet Kluger, MD^5^, Jose Lutzky, MD, FACP^6^, Melissa Wilson^7^, Anna Pavlick, MD, MBA^8^, Brendan Curti, MD^9^, Eric Whitman, MD, FACS^10^, Giao Phan, MD^11^, Marc Ernstoff, MD^12^, Jason Chesney, MD^13^, Toshimi Takamura, BS^14^, Debora Barton, MD^14^, Sam Suzuki, MS^14^, Lavakumar Karyampudi^14^, Nancy Samberg, PhD^14^, Maria Fardis, PhD, MBA^14^

###### ^1^H. Lee Moffitt Cancer Center, Tampa, FL, USA; ^2^Univ. of Florida Health Cancer Center, Orlando, FL, USA; ^3^Univ. Pittsburgh Hillman Cancer Center, Pittsburgh, PA, USA; ^4^Univ Pittsburgh Hillman Cancer Center, Pittsburgh, PA, USA; ^5^Yale Cancer Center, New Haven, CT, USA; ^6^Mount Sinai Comprehensive Cancer Center, Miami Beach, FL, USA; ^7^Thomas Jefferson Kimmel Cancer Center, Philadelphia, PA, USA; ^8^NYU Perlmutter Cancer Center - Langone, New York, NY, USA; ^9^Chiles Research Inst. Providence Cancer, Portland, OR, USA; ^10^Atlantic Health System Cancer Care, Morristown, NJ, USA; ^11^Virginia Commonwealth University, Richmond, VA, USA; ^12^Roswell Park Comprehensive Cancer Center, Buffalo, NY, USA; ^13^James Graham Brown Cancer Center, Louisville, KY, USA; ^14^Iovance Biotherapeutics, Inc., San Carlos, CA, USA

####### **Correspondence:** Nancy Samberg (nancy.samberg@iovance.com)

**Background**

While immunotherapy including checkpoint inhibitors and targeted therapies (BRAF/MEK inhibitors) are options for patients with metastatic melanoma, many patients experience progression. Patients progressed after multiple checkpoints and targeted therapy have few treatment options available including high dose IL-2 and chemotherapy. Response rates of 4-10% have been reported for investigator’s choice of these second line agents. Adoptive cell therapy utilizing tumor-infiltrating lymphocytes (TIL) is recognized as an effective treatment in metastatic melanoma and other solid tumors eliciting durable and complete responses, even in heavily pretreated patients. We provide preliminary data of responses to lifileucel and biomarkers in heavily pre-treated metastatic melanoma patients who have progressed on multiple checkpoint and BRAF/MEK inhibitors (if BRAF mutated).

**Methods**

C-144-01 is a global phase 2, open-label, multicenter study of efficacy and safety of lifileucel in patients with unresectable metastatic melanoma. We report on Cohort 2 (N=30) patients who received cryopreserved Gen-2 lifileucel. Tumors resected at local institutions are processed at central GMP facilities in a 22-day manufacturing process. The final product is cryopreserved and shipped to the sites. Patients receive one week of a preconditioning cyclophosphamide/fludarabine lymphodepletion regimen, followed by a single infusion of lifileucel, plus up to 6 doses of intravenous IL-2 (600,000 IU/kg).

**Results**

Cohort 2 patients with Stage IIIC/IV melanoma had a median of 3 prior therapies. Concomitantly administered treatment regimens containing multiple agents, such as ipilimumab and nivolumab, were counted as a single prior therapy if the therapies were started within 28 days of one another. Preliminary results from this ongoing study indicate efficacy in Cohort 2: ORR=33% (1 uCR, 7 PR, 2 uPR), DCR=73%, median follow-up of all patients was 6 months, median time to initial response 1.7 months (range: 1.6-4.4 months), and median DOR not reached (8 ongoing responders out of 10). Median follow up for all responders was 4.5 months. A longer follow-up led to improving responses in some patients including the CR. Per investigator assessment, none of the grade 5 SAEs were due to any of the study treatment. Lifileucel-induced in vitro IFNγ response to antibody coated beads (anti-CD3, anti-CD28, anti-CD137) and serum IP-10 levels post-treatment, correlates with reduction in tumor (Sum of Diameter of target lesions) and/or overall response.

**Conclusions**

These preliminary data support TIL therapy with lifileucel as an efficacious and well tolerated therapeutic option for patient with metastatic melanoma who have failed multiple lines of prior therapies including checkpoint inhibitors and BRAF/MEK inhibitors (if BRAF mutated).

**Trial Registration**

NCT02360579

**Ethics Approval**

The C-144-01 study was approved by Western Institutional Review Board, approval number 20160198, along with additional approvals by applicable institutional Ethics/Review Boards.

**Table 1 (abstract O22).**
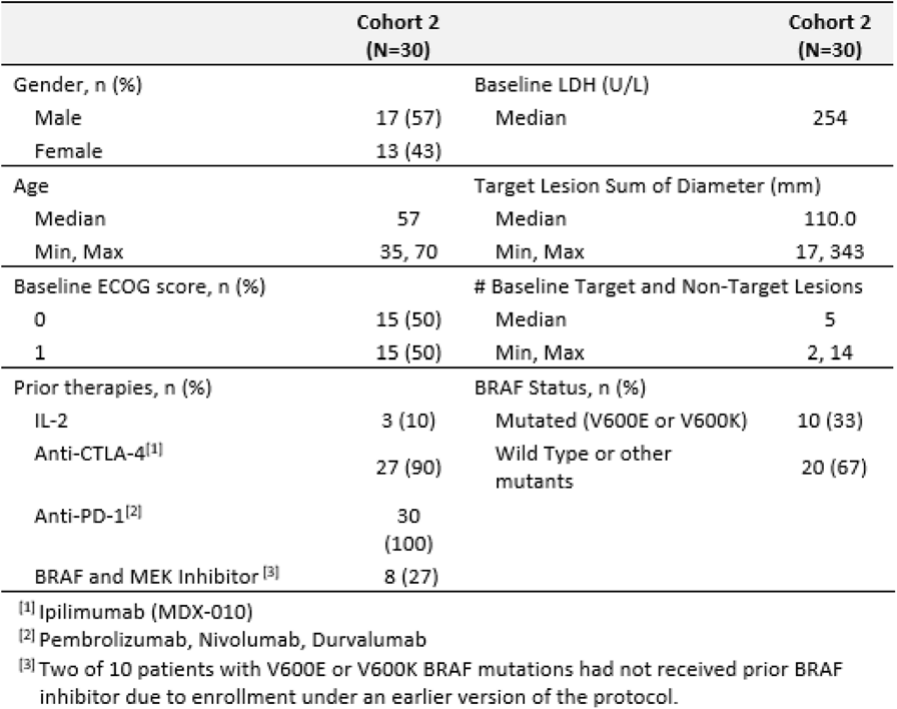
Demographics & Baseline Characteristics

#### O23 Combination of subcutaneous selicrelumab (CD40 agonist) and vanucizumab (anti-Ang2/VEGF) in patients with solid tumors demonstrates early clinical activity and a favorable safety profile

##### Emiliano Calvo, MD PhD^1^, Jan Schellens^2^, Ignacio Matos^3^, Elena Garralda^3^, Morten Mau-Soerensen^4^, Aaron Hansen^5^, Maria Martinez-Garcia^6^, Martijn Lolkema^7^, Jehad Charo, PhD^8^, Chiara Lambertini^8^, Christoph Mancao^8^, Katrijn Bogman^8^, Cristiano Ferlini, MD^8^, Martin Sern, MD^9^, Willeke Ros, MSc^2^

###### ^1^START Madrid, Madrid, Spain; ^2^The Netherlands Cancer Institute, Amsterdam, Netherlands; ^3^Vall d'Hebron University Hospital, Barcelona, Spain; ^4^Rigshospitalet, Copenhagen, Denmark; ^5^Princess Margaret Hospital, Toronto, Canada; ^6^Hospital del Mar, Barcelona, Spain; ^7^Erasmus Medical Center, Rotterdam, Netherlands; ^8^Roche pRED, Schlieren, Switzerland; ^9^Roche Innovation Center Zurich, Schlieren, Switzerland

####### **Correspondence:** Martin Sern (martin.stern@roche.com)

**Background**

Selicrelumab is a fully human agonistic IgG2 monoclonal antibody to CD40, a member of the TNF-receptor superfamily expressed on antigen-presenting cells (APC), endothelial cells and some tumors. CD40 activation of APCs primes T-cells, whereas vanucizumab (anti-Ang2/VEGF bispecific antibody) - on top of anti-angiogenic effects - supports dendritic cell maturation and inhibits immunosuppressive signals. Here, results of the dose escalation study exploring selicrelumab administered subcutaneously (SC) combined with vanucizumab are reported.

**Methods**

This is an ongoing study in patients with advanced/metastatic solid tumors not amenable to standard therapy (NCT02665416) assessing safety, PK/PD, and antitumor activity by RECIST and irRC.

**Results**

Fifty-nine patients received intravenous vanucizumab (2g; q2w) followed by SC selicrelumab at doses of 1-72mg (q4w). Selicrelumab serum concentrations were below the limit of quantification at doses up to 14mg, higher doses resulted in measurable serum levels with high inter-subject variability. Dose-limiting toxicity occurred in 1 patient with injection site reaction (ISR) G3 in the 8mg selicrelumab cohort. The most frequent observed AEs were ISR (91.5%), pyrexia (54.2%), nausea (49.2%), fatigue and hypertension (44.1% each). No selicrelumab-related G4/G5 AEs were reported, vanucizumab toxicity was as expected for a VEGF inhibitor. Best response by RECIST in 54 efficacy-evaluable pts was 1 CR (bladder cancer, 32mg) + 1 PR (ovarian cancer, 24mg), and 29 SD. SD patients included 3 patients with unconfirmed PR and one irPR (medullary thyroid, head and neck, esophageal, and adrenal carcinoma; at 8, 12, 14, and 14mg, respectively); two of these patients stopped treatment due to wound healing complications after rapid and deep response.Exploratory biomarker analyses showed selicrelumab-dose dependent peripheral B-cell depletion, despite non-detectable serum drug levels, followed by activation/proliferation of CD8+ T-cells - most prominently observed at intermediate selicrelumab dose levels (8-32mg). Furthermore, tumor infiltration of activated/proliferating T-cells was generally higher at these dose levels. Baseline B-cell counts in tumor and periphery and post-treatment activation of peripheral CD8 T-cells potentially correlate with radiological response. Immune modulation is being investigated by comparing gene expression patterns of paired baseline vs. on-treatment biopsies.

**Conclusions**

Selicrelumab with vanucizumab shows pharmacodynamic and early clinical activity in patients with advanced solid tumors. Correlative analyses suggest a contribution of CD40 agonism to the activity observed. The combination demonstrated a favorable safety profile with no evidence for systemic autoimmune toxicity. The results triggered an expansion of the study in selected tumor indications with 16mg SC selicrelumab in combination with bevacizumab (anti-VEGF).

**Trial Registration**

This study is registered on Clinicaltrials.gov: NCT02665416

**Ethics Approval**

This study was approved by the local IRB at each participating study site.

### Combination Therapy

#### O24 A phase 1, open-label, dose-escalation study of enoblituzumab in combination with pembrolizumab in patients with select solid tumors

##### Charu Aggarwal, MD MPH^1^, Anthony Joshua, MD^2^, Robert Ferris, MD, PhD^3^, Scott Antonia, MD, PhD^4^, E E. Rahma, MD^5^, Anthony Tolcher, MD, FRCP(C)^6^, Roger Cohen^1^, Yanyan Lou, MD^7^, Ralph Hauke, MD^8^, Nicholas Vogelzang, MD^9^, Dan Zandberg, MD^10^, Arash Rezazadeh Kalebasty, MD^11^, Victoria Atkinson, MD^12^, Alex Adjei, MD PhD^7^, Mahesh Seetharam^7^, Ariel Birnbaum, MD^13^, Andrew Weickhardt, MBBS, FRACP, DMedSc^14^, Vinod Ganju, MBBS, FRACP^15^, Riva Bondarenko, PhD^16^, Linda Peng, MS^16^, Tony Wu, PhD^16^, Scott Currence^16^, Jan Baughman, MPH^16^, Ezio Bonvini^16^, Stacie Goldberg, MD^16^, Jon Wigginton, MD^16^, Nehal Lakhani, MD, PhD^17^

###### ^1^University of Pennsylvania, Philadelphia, PA, USA; ^2^St. Vincent's Hospital, Syndey, Australia; ^3^University of Pittsburgh, Pittsburgh, PA, USA; ^4^Moffitt Cancer Center, Tampa, FL, USA; ^5^Dana Farber Cancer Institute, Boston, MA, USA; ^6^START, San Antonio, TX, USA; ^7^Mayo Clinic, Jacksonville, FL, USA; ^8^Nebraska Cancer Specialists, Omaha, NE, USA; ^9^Comprehensive Cancer Centers of Nevada, Las Vegas, NV, USA; ^10^University of Maryland, Baltimore, MD, USA; ^11^Norton Cancer Institute, Louisville, KY, USA; ^12^University of Queensland, Woolloongabba, Australia; ^13^Rhode Island Hospital, Providence, RI, USA; ^14^Olivia Newton-John Cancer Research Inst., Melbourne, VT, Australia; ^15^Peninsula and Southeast Oncology, Frankston, Australia; ^16^MacroGenics, Inc., Rockville, MD, USA; ^17^Start Midwest, Grand Rapids, MI, USA

####### **Correspondence:** Stacie Goldberg (goldbergs@macrogenics.com)

**Background**

Enoblituzumab is a humanized IgG1 monoclonal antibody (mAb) targeting B7-H3 (CD276), and is Fc-engineered to enhance antibody-dependent cell-mediated cytotoxicity. B7-H3 has limited normal tissue expression, but is highly expressed in many solid tumors. Enoblituzumab monotherapy has demonstrated antitumor activity with an acceptable safety profile in patients with selected solid tumors. Pembrolizumab is a humanized IgG4 anti-PD-1 mAb and is FDA approved for multiple solid tumor indications. It is hypothesized that coordinate engagement of both innate and adaptive immunity via targeting of two distinct members of the B7 family, the combination of enoblituzumab and pembrolizumab could achieve greater antitumor activity than either agent alone.

**Methods**

Patients with advanced/metastatic solid tumors received weekly (qwk) enoblituzumab (doses 3-15 mg/kg) IV plus pembrolizumab (2 mg/kg) IV q3wk during dose-escalation (3+3 design) and cohort expansion. Tumor B7-H3 and PD-L1 expression was assessed via immunohistochemical staining. Disease assessment occurred after 6 weeks, then q9wks thereafter. Expansion cohorts included non-small cell lung cancer (NSCLC; checkpoint-inhibitor naïve, PD-L1 <1%) NSCLC (post checkpoint inhibitor), squamous cell carcinoma of the head and neck (SCCHN; checkpoint-inhibitor naïve), SCCHN (post checkpoint inhibitor), and urothelial cancer (UC) and melanoma (post checkpoint inhibitor).

**Results**

The combination demonstrated acceptable tolerability in the overall population (N=133) at doses up to enoblituzumab 15 mg/kg+pembrolizumab (2 mg/kg), with no maximum tolerated dose defined. Treatment-related adverse events (AE, all grade) occurred in 85% of patients, with > G3 in 28%. Infusion-related reaction and elevated lipase (6%) were the only Grade > 3 AE occurring in > 5% of patients. One treatment-related death due to pneumonitis occurred. The combination demonstrated antitumor activity in checkpoint-inhibitor-naïve SCCHN patients, and also induced objective responses in patients anticipated to have relatively limited responsiveness to pembrolizumab alone, including NSCLC with tumor PD-L1 expression <1% and checkpoint-inhibitor-refractory UC. To date, objective responses have occurred in 6/18 (33%) response-evaluable, checkpoint-inhibitor-naïve SCCHN patients, including 4 confirmed and 2 unconfirmed PR, with stable disease in 6/18 (33%). Among SCCHN patients with tumor B7-H3 expression >10%, the ORR was 40% (6/15). In NSCLC patients (PD-1 naïve, tumor PD-L1 <1%), there were 4/14 PR (29%; 2 confirmed and 2 unconfirmed) and 9 SD (64%). Two of 16 post-checkpoint-inhibitor UC patients achieved a PR and unconfirmed CR, respectively.

**Conclusions**

The enoblituzumab+pembrolizumab combination demonstrated an acceptable safety profile, encouraging initial antitumor activity in patients with checkpoint-inhibitor-naïve SCCHN, and the ability to induce partial responses in patients anticipated to be poorly responsive to checkpoint inhibitor alone.

**Trial Registration**

NCT02475213

**Ethics Approval**

This study was approved by each Institution's Ethics Board prior to enrolling.

#### O25 Phase 1 dose-finding study of the anti–TIGIT antibody MK-7684 as monotherapy and in combination with pembrolizumab in patients with advanced solid tumors

##### Talia Golan^1^, Todd Bauer, MD^2^, Antonio Jimeno, MD, PhD^3^, Ruth Perets^4^, Jiaxin Niu^5^, James Lee, MD, PhD^6^, Mallika Lala^7^, Jennifer Garrus^7^, Zhen Zeng^7^, Elliot Chartash^7^, Jane Healy^7^, Drew Rasco, MD^8^

###### ^1^Sheba Medical Center, Ramat Gan, Israel; ^2^Sarah Cannon Research Institute/TN Oncol, Nashville, TN, USA; ^3^University of Colorado, Denver, CO, USA; ^4^Rambam Medical Center, Haifa, Israel; ^5^Banner MD Anderson Cancer Center, Gilbert, AZ, USA; ^6^University of Pittsburgh Medical Center, Pittsburgh, PA, USA; ^7^Merck & Co., Inc., Kenilworth, NJ, USA; ^8^START, San Antonio, TX, USA

####### **Correspondence:** Talia Golan (Talia.Golan@sheba.health.gov.il)

**Background**

TIGIT (T-cell immunoreceptor with Ig and ITIM domains) is an immunomodulatory receptor that functions as a negative immune checkpoint. In preclinical models, TIGIT inhibition provided antitumor activity, with an enhanced effect observed when combined with PD-1 inhibition. MK-7684 is a humanized, IgG1 monoclonal antibody that binds TIGIT and blocks its interaction with its ligands, CD112 and CD155. We present the dose escalation portion of the first-in-human study of MK-7684 as monotherapy or in combination with pembrolizumab in patients with advanced solid tumors.

**Methods**

Eligible patients had metastatic solid tumors that failed standard treatment options, measurable disease, and ECOG PS 0-1. MK-7684 dose escalation followed a modified toxicity probability interval design with a target DLT rate during cycle 1 of ~30% and planned doses of 2.1, 7, 21, 70, and 210 mg Q3W. MK-7684 and pembrolizumab 200 mg Q3W were given for up to 35 cycles or until progression, intolerable toxicity, or investigator or patient decision. Study objectives included evaluation of safety and tolerability, pharmacokinetics, and ORR of MK-7684 monotherapy and MK-7684 plus pembrolizumab. Data cutoff date was July 16, 2018.

**Results**

68 patients were treated. 34 patients received MK-7684 monotherapy; median age was 67.5 years, 50% had ECOG PS 1, and 50% received ≥3 prior therapies. 34 patients received MK-7684 plus pembrolizumab; median age was 62.5 years, 65% had ECOG PS 1, and 32% received ≥3 prior therapies. There were no DLTs. Across doses, treatment-related AEs occurred in 53% of monotherapy and 65% of combination therapy recipients (grade 3-5, 6% and 12%); no patients died or discontinued because of treatment-related AEs. The most common treatment-related AEs were fatigue (15%) and pruritus (12%) with MK-7684 and pruritus (21%) and rash (15%) with MK-7684 plus pembrolizumab. The pharmacokinetic profile of MK-7684 was generally consistent with that of a typical monoclonal antibody. Exposure increased with increasing dose, and target-mediated drug disposition was observed at low doses. Clearance was linear at a dose of 210 mg. ORR (confirmed+unconfirmed) was 3% with monotherapy (1 PR) and 18% with combination therapy (6 PRs). DCR was 35% and 48%, respectively.

**Conclusions**

MK-7684 as monotherapy and in combination with pembrolizumab 200 mg Q3W was well tolerated and had an acceptable safety profile across all dose levels. Promising antitumor activity was seen, especially for the combination. Dose confirmation and evaluation of efficacy for monotherapy and combination therapy is ongoing in patients with select advanced solid tumors.

**Trial Registration**

ClinicalTrials.gov, NCT02964013

#### O26 The anti–LAG-3 antibody MK-4280 as monotherapy and in combination with pembrolizumab for advanced solid tumors: first-in-human phase 1 dose-finding study

##### Nehal Lakhani, MD, PhD^1^, Todd Bauer, MD^2^, Anson Abraham, PhD^3^, John Luddy^3^, John Palcza, MS^3^, Elliot Chartash^3^, Jane Healy^3^, Amita Patnaik, MD FRCP(C)^4^

###### ^1^START-Midwest, Grand Rapids, MI, USA; ^2^Sarah Cannon Research Institute/TN Oncol, Nashville, TN, USA; ^3^Merck & Co., Inc., Kenilworth, NJ, USA; ^4^START, San Antonio, TX, USA

####### **Correspondence:** Nehal Lakhani (nehal.lakhani@startmidwest.com)

**Background**

LAG-3 (lymphocyte-activation gene 3) is an immunomodulatory receptor that regulates T_eff_ homeostasis, proliferation, and activation and has a role in T_reg_ suppressor activity. Preclinical data suggest that dual LAG-3/PD-1 blockade synergistically reverses tumor-specific anergy. MK-4280 is a humanized, IgG4, anti–LAG-3 monoclonal antibody that prevents LAG-3 from binding its ligand, MHC class II. We present dose-escalation results of the first-in-human study of MK-4280 as monotherapy or in combination with pembrolizumab for patients with advanced solid tumors.

**Methods**

Adults with metastatic solid tumors without clinically effective treatment, measurable disease, and ECOG PS of 0-1 were eligible. Dose finding for monotherapy and combination therapy followed standard 3+3 dose escalation using MK-4280 doses of 7, 21, 70, 210, and 700 mg Q3W. MK-4280 and pembrolizumab 200 mg Q3W were given for 35 cycles or until progression, intolerable toxicity, or investigator or patient decision. The DLT evaluation period was cycle 1. Study objectives included evaluation of the safety and tolerability, pharmacokinetics, and ORR of MK-4280 as monotherapy and in combination with pembrolizumab. Data cutoff date was June 12, 2018.

**Results**

18 patients received MK-4280 monotherapy; 15 received MK-4280 plus pembrolizumab. Median age was 60 years, 67% of patients had ECOG PS 1, and 48% received ≥3 prior therapies. MK-4280 dose escalation proceeded to 700 mg Q3W for both monotherapy and combination therapy without any DLTs. Treatment-related AEs occurred in 61% of monotherapy and 53% of combination therapy recipients, were of grade 3-4 toxicity in 6% and 20%, and led to discontinuation in 6% and 13%. There were no treatment-related deaths. Treatment-related AEs that occurred in ≥10% of patients were fatigue (17%) and arthralgia (11%) for monotherapy and fatigue (20%), pyrexia (20%), pruritus (13%), and maculopapular rash (13%) for combination therapy. The pharmacokinetic profile was generally consistent with that of a typical monoclonal antibody. Exposure increased with increasing dose, and target-mediated drug disposition was observed at low doses. Clearance was linear at doses of 210 mg and 700 mg. ORR was 6% with monotherapy (1 PR) and 27% with combination therapy (4 PRs). DCR was 17% and 40%, respectively.

**Conclusions**

MK-4280 as monotherapy and in combination with pembrolizumab was well tolerated and had an acceptable safety profile across all dose levels. Promising antitumor activity was observed, particularly for the combination. Dose confirmation and efficacy evaluation of MK-4280 alone and in combination with pembrolizumab is ongoing in patients with select advanced solid tumors.

**Trial Registration**

ClinicalTrials.gov, NCT02720068

#### O27 Phase 2 trial of mocetinostat in combination with durvalumab in NSCLC patients with progression on prior checkpoint inhibitor therapy

##### Melissa L. Johnson, MD^1^, Keith Eaton, MD^2^, Balazs Halmos, MD^3^, Edward Garon, MD^4^, Thomas Hensing, MD^5^, Nisha Mohindra, MD^6^, James Strauss, MD^7^, Timothy McCarthy^8^, Rami Owera^9^, Isan Chen, MD^10^, Peter Olson^10^, Demiana Faltaos, PharmD, PhD^10^, James Christensen^10^, Diane Potvin^10^, Tavette Neskorik^10^, Adam Pavlicek^11^, Manish Patel, DO^12^

###### ^1^Sarah Cannon Research Institute, Nashville, TN, USA; ^2^Seattle Cancer Care Alliance, Seattle, WA, USA; ^3^Montefiore Medical Center, White Plains, NY, USA; ^4^University of California-Los Angeles, Santa Monica, CA, USA; ^5^NorthShore University Health System, Evanston, IL, USA; ^6^Northwestern University, Chicago, IL,USA; ^7^Mary Crowley Cancer Research Center, Dallas, TX, USA; ^8^Virginia Cancer Specialists, Fairfax, VA, USA; ^9^Woodlands Medical Specialists, Pensacola, FL, USA; ^10^Mirati Therapeutics, San Diego, CA, USA; ^11^Monoceros Biosystems Inc., San Diego, CA, USA; ^12^University of Minnesota Masonic Cancer, Minneapolis, MN, USA

####### **Correspondence:** Melissa L. Johnson (mjohnson@tnonc.com)

**Background**

Mocetinostat, a spectrum-selective class I histone deacetylase inhibitor, has multiple potential immunomodulatory features including: 1) induction of major histocompatibility complex Class I and Class II expression on tumor cells, 2) enhanced function of T effector cells, and 3) decreased function of immunosuppressive cell subsets including regulatory T cells and myeloid derived suppressor cells. Given these pleiotropic immune activating effects, the combination of mocetinostat and the PD-L1 blocking mAb durvalumab was tested in NSCLC patients (pts) with checkpoint inhibitor therapy (CIT) naïve disease or had progressive disease after prior CIT.

**Methods**

Phase 1 of the study explored increased doses of mocetinostat administered orally (50, 70, 90 mg three times weekly [TIW]) in combination with durvalumab in patients with advanced solid tumors. Review of all safety data supported a recommended Phase 2 dose of mocetinostat 70 mg TIW with durvalumab (1500 mg) on day 1 of each 28-day cycle. Study objectives include evaluation of safety and Objective Response Rate (ORR) in pts with NSCLC who have progression of disease (PD) on or after treatment with CIT or are CIT-naïve. Two CIT-experienced cohorts enrolled pts based on prior clinical benefit with CIT. A predictive probability design is used for assessment of enrollment expansion in each stage and treatment arm. Other objectives include tolerability, pharmacokinetics, incidence of anti-drug antibody and multiple correlative endpoints.

**Results**

Enrollment in Stage 1 of the CIT-experienced cohorts is complete. As of July 13, 2018, 37 patients who received at least one dose of mocetinostat and durvalumab were included in the assessment. Six of the 37 pts achieved a partial response (PR); 4 confirmed and 2 unconfirmed (2/24 PRs in Prior Clinical Benefit; 2 PRs/2 uPRs/13 in No Prior Clinical Benefit cohorts, respectively) and 11/37 pts demonstrated tumor reductions; with the longest treatment duration exceeding 55 weeks. Treatment-related AEs (>10% of pts; all grades) included fatigue, nausea, diarrhea, vomiting, and decreased appetite. Updated safety, efficacy, and correlative science data will be presented.

**Conclusions**

Based on preliminary data, the combination of mocetinostat with durvalumab is clinically active with manageable side effects.

**Trial Registration**

Clinical Trial Information: NCT02805660

**Ethics Approval**

This study was approved by Copernicus Group Independent Review Board; approval tracking PRA0-16-027.

### Co-Stimulatory Ligand-Receptor Interactions

#### O28 Results from a Phase I dose escalation trial (TACTI-mel) with the soluble LAG-3 protein (IMP321, eftilagimod alpha) together with pembrolizumab in unresectable or metastatic melanoma

##### Frederic Triebel, MD, PhD^1,2^, Christian Mueller^2^, Chrystelle Brignone^2^, Victoria Atkinson, MD^3^, Melissa Eastgate, MD^4^, Amitesh Roy, MD^5^, Adnan Khattak, MD^6^, Andrew Haydon, MBBS PhD^7^

###### ^1^Prima Biomed Ltd, Orsay, France; ^2^Immutep, Orsay, France; ^3^Princess Alexandra Hospital, Woolloongabba, Australia; ^4^Royal Brisbane Womens Hospital, Herston, Australia; ^5^Flinders Centre for Innovation in Cancer, Bedfork Park, Australia; ^6^Fiona Stanley Hospital, Murdoch, Australia; ^7^Alfred Hospital, Melbourne, Australia

####### **Correspondence:** Frederic Triebel (ftriebel@immutep.com)

**Background**

Eftilagimod alpha (efti, IMP321) is a recombinant soluble LAG-3Ig fusion protein binding to MHC class II molecules and mediating antigen presenting cell (APC) activation followed by CD8 T-cell activation. The activation of the dendritic cell network and the subsequent T cell recruitment at the tumor site with efti may lead to stronger anti-tumor CD8 T cell responses. Combining an APC activator with an immune checkpoint inhibitor (ICI) aims to increase efficacy without additional toxicity. We report results of the dose escalation phase I trial (NCT02676869) with pembrolizumab and efti.

**Methods**

Melanoma patients (pts) on pembrolizumab (2 mg/kg i.v.) with progressive disease (irPD), stable disease (irSD) or partial response (irPR) acc. to irRC after 3 cycles received 1 mg (n=6), 6 mg (n=6) or 30 mg (n=6) s.c. injections of efti (every 2 weeks for 6 months) from cycle 5 of pembrolizumab onwards. Blood samples for pharmacokinetics were taken in cycle 1 and 9.

**Results**

In stage A, 18 pts (17 male, 1 female) with a median age of 66 years (range 48-85) were enrolled. Fifteen (83 %) and seven (39 %) pts had stage M1C disease and elevated LDH, respectively. Seven (39 %) pts completed the 6 months combination treatment. Reasons for discontinuation were irPD (n=7), withdrew consent (n=2) and death not related to efti or pembrolizumab (n2). The most common AEs were fatigue (44 %), rash (33 %), diarrhea (28 %), nausea (28 %), arthralgia (17 %) and colitis (11 %). One patient experienced intracranial hemorrhage grade 4, another had a colitis grade 4, both not related to efti or pembrolizumab. No dose limiting toxicity has been reported. A dose-dependent increase in serum IMP321 concentration was observed among the three dose levels with a Cmax between 4 and 24 hours. Objective response rate (ORR) taking cycle 5 of pembrolizumab as baseline was 33 % (irRC) including one patient with a confirmed irCR after progress on pembrolizumab monotherapy.

**Conclusions**

Up to 30 mg efti in combination with pembrolizumab are safe and well tolerated. The responses observed in pts with suboptimal response to pembrolizumab alone may point to a benefit of adding a systemic APC activator to an ICI.

**Trial Registration**

NCT02676869

**Ethics Approval**

The study was approved by Metro South Human Research Ethics Commitee (Australia), approval number HREC/15/QPAH/726

**Consent**

Written informed consent was obtained from the patient for publication of this abstract and any accompanying images. A copy of the written consent is available for review by the Editor of this journal.

### Co-Stimulatory Ligand-Receptor Interactions

#### O29 Immuno-oncology tHERApy with HERA-GITRL: the novel hexavalent human GITR agonist activates T cells and promotes anti-tumor efficacy independent of Fc-functionality

##### Viola Marschall, PhD, Meinolf Thiemann, Jaromir Sykora, David Richards, Christian Merz, Julian Sefrin, Karl Heinonen, Matthias Schroeder, Mauricio Redondo Müller, Christian Gieffers, Oliver Hill

###### Apogenix AG, Heidelberg, Germany

####### **Correspondence:** Christian Gieffers (christian.gieffers@apogenix.com)

**Background**

Glucocorticoid-induced TNFR-related protein (GITR, TNFRSF18, CD357), a TNFR-SF member, is a co-stimulatory receptor that increases anti-tumor T cell activation. Based on Apogenix hexavalent TNFR-SF agonist (HERA-ligand) technology platform, we created a fully human hexavalent GITR ligand fusion protein HERA-GITRL intended for T cell costimulatory approaches in immuno-oncology (IO) therapy. HERA-GITRL is composed of a trivalent single chain GITRL-receptor-binding-domain fused to an IgG1-derived silenced Fc-domain serving as dimerization scaffold. Because of the unique design of the silenced Fc-domain, HERA-GITRL allows the study of pure GITR agonism in contrast to Fc-mediated mixed modes of action. Here we report in vitro and in vivo properties of our novel HERA-GITRL construct.

**Methods**

N/A

**Results**

For functional characterization of HERA-GITRL in vitro, human immune cells isolated from healthy-donor blood were profiled by multicolor flow cytometry and real-time cell analysis (RTCA). Stimulation of unfractionated human T cells or purified naïve CD4+ T cells by anti-CD3 antibody was further augmented by HERA-GITRL. This effect was accompanied by increased proliferation, differentiation and elevated levels of TNF-α and IFN-γ. HERA-GITRL enables effector T cells proliferation even in the presence of regulatory T cells. In line with these findings, the murine surrogate mmHERA-GITRL enhanced antigen-specific clonal expansion of both CD4+ (OT-II) and CD8+ (OT-I) T cells in vivo. In a direct in vitro comparison of the anti-human GITR monoclonal antibody TRX518, trivalent GITRL and hexavalent HERA-GITRL, only HERA-GITRL showed full biological activity independent of additional crosslinking. Importantly, HERA-GITRL mediated T cell activation increases tumor cell killing by PBMCs in vitro. Finally, mmHERA-GITRL showed in vivo anti-tumor efficacy as a single agent in a subcutaneous syngeneic colon cancer model (CT26wt) in mice. The anti-tumor efficacy of mmHERA-GITRL is independent of its Fc functionality, as both mmHERA-GITRL with a functional Fc- and a silenced Fc-domain show a similar tumor growth inhibition.

**Conclusions**

By clustering six receptor chains in a spatially well-defined manner, HERA-GITRL induces potent agonistic activity without being dependent on additional Fc-mediated crosslinking. The anti-tumor effect of most anti-GITR antibodies is dependent on a fully functional Fc domain and is generally mediated by depletion of Treg cells. HERA-GITRL boosts antigen-specific T cell activity and shows anti-tumor efficacy while having no effect on Treg cells. This property of HERA-GITRL makes it particularly suitable for IO-combination therapies. The HERA-ligand concept has also been successfully translated to HERA-TRAIL (now in Phase I), -CD40L, -CD27L, -OX40L, -HVEML (LIGHT) and -4-1BBL.

**Ethics Approval**

The experimental protocols were registered by the regional board in Freiburg, Germany (Regierungspräsidium Freiburg; approval number G-15/41).

### Cytokines in Anti-Tumor Immunity

#### O30 First-in-human phase 1 dose-escalation trial of the potent and selective next generation transforming growth factor-β receptor type 1 (TGF-βR1) inhibitor LY3200882 in patients with advanced cancers

##### Timothy Yap, MD^2^, Capucine Baldini, MD^3^, Christophe Massard, MD, PhD^3^, Ivelina Gueorguieva, PhD^1^, Yumin Zhao, PhD^1^, Shelly Schmidt^1^, Michael Man, PhD^1^, Shawn Estrem, PhD^1^, Karim Benhadji, MD^1^, Maria Vieito, MD^4^

###### ^1^Eli Lilly and Company, Indianapolis, IN, USA; ^2^Univ. of Texas MD Anderson Cancer Center, Houston, TX, USA; ^3^Gustave Roussy Cancer Campus, Villejuif, France; ^4^Vall d’Hebron University Hospital & VHIO, Barcelona, Spain

####### **Correspondence:** Timothy Yap (tyap@mdanderson.org)

**Background**

The TGF-β pathway is commonly deregulated in cancer and plays a pivotal role during tumorigenesis. LY3200882 is a potent and selective TGF-βR1 inhibitor. This multicenter, nonrandomized, open-label, phase 1 study assessed the recommended phase 2 dose of LY3200882 as monotherapy in patients (pts) with advanced cancers.

**Methods**

Pts with refractory advanced or metastatic cancer were enrolled and were required to have adequate organ function as well as ECOG PS of 0 or 1. LY3200882 tosylate salt (hereafter referred to as LY3200882) was given twice daily (BID) to 5 cohorts of patients at increasing doses (5 to 50 mg) for 14 days in a 28 day cycle. Primary endpoint was to determine the recommended phase 2 dose. Secondary objectives included safety, response assessments and pharmacokinetics (PK), and exploratory objectives included pharmacodynamics (PD).

**Results**

30 pts were enrolled (19 males and 11 females); median age 47 y (32 y-74 y). The most common tumors were gliomas (15 pts), of which 5 were glioblastomas. No dose-limiting toxicities were observed. All treatment-related adverse events were grade 1-2 and most frequently included thrombocytopenia (2 pts), acneiform dermatitis (2 pts), rash (2 pts, 1 maculopapular rash and 1 pustular rash), and constipation (2 pts). LY3200882 was absorbed within 2-6 hours (h) and mostly eliminated within 48 h, with mean terminal half-life of 7.4 h (*n*=15). Drug exposures increased with escalating dose levels, up to the highest planned dose in the targeted therapeutic range of 50 mg BID. Plasma exposures in patients receiving the 50 mg BID dose were comparable to efficacious exposure levels from preclinical *in vivo* target inhibition and efficacy models. Detailed PD biomarker studies are ongoing and data will be presented. A confirmed partial response per Response Assessment in Neuro-Oncology (RANO) of a 85.7% decrease was observed in a glioblastoma pt (*EGFR* mutation; *CDK4* amplification; *IDH1/2* wildtype, *MGMT* methylated) treated at 50 mg BID who remains on LY3200882 for >11 months. Tumor regression (1.3%-85.7%) was observed in 5 pts (oligoastrocytoma, glioma (2 pts), astrocytoma, and glioblastoma).

**Conclusions**

LY3200882 monotherapy is well tolerated, with appropriate plasma exposures observed at the recommended phase 2 dose of 50 mg BID for 14 days in 28 days cycle, where preliminary antitumor activity has been observed. An expansion cohort assessing pts with glioblastoma is ongoing.

**Trial Registration**

ClinicalTrials.gov NCT02937272

**Ethics Approval**

MD Anderson Office of Protocol Review IRB approved protocol number 2016-0582 on 25-Aug-2016; Gustave Roussy Ethic Committee (Comite de Protection des Personnes Ile de France 3) approved protocol on 15-Nov-2016 and Competent Authority (Agence Nationale de Sécurité des Médicaments et de produits de santé) approved on 27-Oct-2016; VHIO Ethic Committee (Dictamen del comite de etica de la investigacion con medicamentos) approved protocol on 30-Sep-2016 and Competent Authority (Agencia espanola de medicamentos y productos sanitarios) approved on 03-Nov-2016; the EudraCT number 2016-001431-12 is the file number indicated on all approvals in Europe

#### O31 Tumor infiltrating lymphocyte recruitment after peri-lymphatic IRX-2 cytokine immunotherapy in resectable breast cancer and head and neck carcinoma

##### Joanna Pucilowska, PhD^2^, Venkatesh Rajamanickam^2^, Nicole Moxon, RN^2^, Katherine Sanchez, MD^1^, Monil Shah, PharmD^3^, James Imatani, MD^1^, Shaghayegh Aliabadi-Wahle, MD^1^, Maritza Martel, MD^2^, Alison Conlin, MD^2^, James Egan, PhD^3^, David Page, MD^2^

###### ^1^Providence Portland Cancer Center, Portland, OR, USA; ^2^Providence Portland Medical Center, Portland, OR, USA; ^3^IRX Therapeutics, Skillman, NJ, USA

####### **Correspondence:** David Page (david.page2@providence.org)

**Background**

The IRX-2 biologic is a subcutaneous injectable immunotherapy composed of IL-2 and other cytokines derived from stimulated lymphocytes. Preclinically, IRX-2 activates T cells, natural killer cells, macrophages, and dendritic cells, and facilitates maturation of antigen-presenting cells. Tumor-infiltrating lymphocytes (TILs) are associated with improved outcomes in many cancers including early stage breast cancer (ESBC) and head and neck squamous cell carcinoma (HNSCC). We report data on TIL recruitment associated with pre-operative IRX-2 in a phase Ib ESBC trial, as well as phase Ib and IIa HNSCC trials.

**Methods**

The pre-operative IRX-2 regimen was evaluated in both ESBC and HNSCC trials for safety and immunologic activity. Beginning 21 days prior to surgical resection, enrolled operable patients with resectable stage I-III ESBC and stage II-IVA HNSCC received single low-dose intravenous cyclophosphamide (300 mg/m2 to facilitate T-regulatory cell depletion), followed by 10 days of subcutaneous injections of IRX-2 (1mL × 2 directed to regional peri-lymphatic space, 230IU/day). Endpoints included feasibility, TIL count by H&E blinded pathology review, and Nanostring RNA analysis.

**Results**

In the ESBC trial, 16 patients were enrolled and evaluable for TIL analysis, whereas in the HNSCC trials, 40 patients were enrolled and 36 patients were evaluable. In both trials, all patients received all planned injections with no treatment-related surgical delays, complications, or treatment-related grade III/IV toxicities. Treatment was associated with a mean 116% relative increase in TILs (range –36% to +1275%, p = 0.02) in ESBC and a mean 58% relative increase (range -57 to +452%, p=0.01) in HNSCC. Treatment was associated with PD-L1 RNA upregulation in EBSC (mean +54%, range –53% to +185%, p=0.04) but not HNSCC, however PD-L1 was higher at baseline in HNSCC. RNA analysis in ESBC and HNSCC revealed concordant increases in cytokine gene expression, including CXCL2, CCL4, CXCR4, and CXCL12 as well as transcription factors including FOS, ETS1, NFKB, EGR1/2 which are involved in T-cell activation and differentiation. We also note augmentation of ITGAE (CD103), a known marker of memory T-cell activation in EBSC cohort.

**Conclusions**

Pre-operative IRX-2 was well tolerated in both tumor histologies with statistically significant TIL recruitment, as well as PD-L1 upregulation in ESBC. Future directions include an evaluation of neoadjuvant IRX-2 with anti-PD-1 and chemotherapy in stage II-III TNBC, ongoing follow-up of a randomized phase IIb trial of neoadjuvant IRX-2 regimen in HNSCC to ascertain clinical benefit, and trials evaluating efficacy of IRX-2/anti-PD-1 combination across various metastatic cancers.

**Trial Registration**

NCT02950259, NCT02609386

**Ethics Approval**

The study was approved by Providence Portland Medical Canter Institutution‘s Ethics Board, approval number 132055.

#### O32 Decoy-resistant Interleukin-18 overcomes the soluble immune checkpoint IL-18BP to unlock a potent immunotherapeutic cytokine pathway

##### Ting Zhou, William Damsky, MD, PhD, Karen Hartmann, MS, Suzanne Fischer, BS, Marcus Bosenberg, MD, PhD, Aaron Ring

###### Yale University School of Medicine, New Haven, CT, USA

####### **Correspondence:** Aaron Ring (Aaron.Ring@yale.edu)

**Background**

Decades before the advent of immune checkpoint inhibitors (ICIs), recombinant cytokine treatments established that the immunotherapeutic paradigm could produce durable cures in patients [1]. From single-cell RNA expression analyses of tumor-infiltrating lymphocytes [2], we found that the Interleukin-18 (IL-18) pathway is markedly upregulated in activated and dysfunctional CD8 T cells, suggesting its promise as an immunotherapeutic target. However, previous clinical trials found that IL-18 therapy is ineffective, with no objective responses seen in a phase 2 study of melanoma patients [3]. We hypothesized that the endogenous, high-affinity IL-18 antagonist, IL-18BP, acts as a “soluble immune checkpoint” that restricts the effectiveness of IL-18 immunotherapy.

**Methods**

Using directed evolution with yeast-surface display, we engineered a “decoy-resistant” IL-18 variant (DR-18) that is impervious to neutralization by IL-18BP. We measured DR-18’s affinity for IL-18Ralpha and IL-18BP using surface plasmon resonance and its ability to stimulate downstream signaling with phosphoflow cytometry of primary lymphocytes. We assessed the anti-tumor efficacy of DR-18 in immunogenic tumor models and ICI-resistant models that lack MHC class I surface expression.

**Results**

DR-18 exhibits >10,000,000-fold reduced affinity for IL-18BP compared to WT IL-18 and can potently elicit MyD88 signaling despite concentrations of IL-18BP sufficient to completely neutralize WT IL-18. Similar to the clinical experience with recombinant IL-18, WT IL-18 produced no benefit alone or in combination with anti-PD-1 in the treatment of MC38, YUMMER1.7, or CT26 tumors. By contrast, DR-18 produced monotherapeutic efficacy commensurate with anti-PD-1, with robust synergism observed in combination therapy. Antibody-mediated depletion studies indicated that DR-18’s efficacy in these models required CD8 and CD4 cells, but not NK cells. To determine whether DR-18 was effective in the setting of ICI-resistance, we utilized three tumor models with impaired MHC class I surface expression (RMA/S and Beta-2 microglobulin-deficient MC38 and YUMMER1.7). In these models, DR-18 treatment exhibited strong NK-cell dependent efficacy, whereas no response was observed with combined blockade of CTLA4 and PD-1.

**Conclusions**

We established that IL-18BP is the key barrier to IL-18 immunotherapy using an engineered cytokine variant, DR-18, that is impervious to the decoy receptor. DR-18 is a promising candidate immunotherapeutic that can augment conventional ICIs and treat ICI-refractory tumors that have lost antigen presentation through MHC class I, one of the most common mechanisms of ICI resistance [4]. These results highlight the IL-18 pathway as a powerful target for immunotherapeutic intervention and establish the value of precisely-engineered protein therapeutics to dissect complex immunoregulatory pathways.

**References**

1. Atkins MB, Kunkel L, Sznol M, Rosenberg SA. High-dose recombinant interleukin-2 therapy in patients with metastatic melanoma: long-term survival update. Cancer J Sci Am. 2000;6 Suppl 1:S11-4.

2. Singer M, Wang C, Cong L, Marjanovic ND, Kowalczyk MS, Zhang H, Nyman J, Sakuishi K, Kurtulus S, Gennert D, Xia J, Kwon JY, Nevin J, Herbst RH, Yanai I, Rozenblatt-Rosen O, Kuchroo VK, Regev A, Anderson AC. A Distinct Gene Module for Dysfunction Uncoupled from Activation in Tumor-Infiltrating T Cells. Cell. 2016;166(6):1500-11 e9.

3. Tarhini AA, Millward M, Mainwaring P, Kefford R, Logan T, Pavlick A, Kathman SJ, Laubscher KH, Dar MM, Kirkwood JM. A phase 2, randomized study of SB-485232, rhIL-18, in patients with previously untreated metastatic melanoma. Cancer. 2009;115(4):859-68.

4. Zaretsky JM, Garcia-Diaz A, Shin DS, Escuin-Ordinas H, Hugo W, Hu-Lieskovan S, Torrejon DY, Abril-Rodriguez G, Sandoval S, Barthly L, Saco J, Homet Moreno B, Mezzadra R, Chmielowski B, Ruchalski K, Shintaku IP, Sanchez PJ, Puig-Saus C, Cherry G, Seja E, Kong X, Pang J, Berent-Maoz B, Comin-Anduix B, Graeber TG, Tumeh PC, Schumacher TN, Lo RS, Ribas A. Mutations Associated with Acquired Resistance to PD-1 Blockade in Melanoma. N Engl J Med. 2016;375(9):819-29.

**Ethics Approval**

These studies were approved by the Yale Institutional Animal Care & Use Committee (IACUC), approval number 2016-20117.

#### O33 Adaptive plasticity of IL10+ and IL35+ regulatory T cells and their cooperative regulation of anti-tumor immunity

##### Hiroshi Yano, BS, Deepali Sawant, PhD, Maria Chikina, PhD, Qianxia Zhang, PhD, Zhe Sun, Tao Sun, Wei Chen, Creg Workman, PhD, Dario A. Vignali, PhD

###### University of Pittsburgh, Pittsburgh, PA, USA

####### **Correspondence:** Dario A. Vignali (dvignali@pitt.edu)

**Background**

Regulatory T cells (Tregs), while contributing to the maintenance of self-tolerance, are a barrier to effective cancer immunotherapy [1]. Previous studies have shown that tumor-infiltrating (TIL) Tregs substantially upregulate the expression of inhibitory cytokines, such as IL10 [2] and IL35 [3]. Although we have reported that IL35+ Tregs support tumor growth by promoting inhibitory receptor (IR) expression in CD8+ TILs [3], the underlying mechanism of IL35-mediated IR-upregulation and whether and how IL35+ Tregs and IL10+ Tregs cooperatively regulate anti-tumor immunity remain elusive. Improving our understanding of Treg functions in the tumor microenvironment (TME) will help us identify new targets and develop novel immunotherapy with greater efficacy.

**Methods**

To address the question, we generated Ebi3tdTomato.Il10GFP.Foxp3Cre-YFP reporter mice to assess whether there are phenotypic, functional, and transcriptomic differences between Treg subpopulations. We also utilized Foxp3Cre-YFP, Ebi3L/L-tdTom.Foxp3Cre-YFP, Il10L/L.Foxp3Cre-YFP, and Ebi3L/L.tdTom.Il10L/L.Foxp3Cre-YFP conditional knockout mice to examine the impact of inhibitory cytokine-deficient Tregs on CD8+ TILs via multi-parameter flow cytometry and global transcriptomic analyses.

**Results**

IL10 and IL35 were reciprocally expressed by Tregs, while inhibitory cytokine-producing Tregs were significantly enriched in the TME. However, single cell RNAseq analysis and a significant clonal overlap among TIL Tregs demonstrated by TCRseq revealed Tregs in transitional-states between IL10+, IL35+, and IL10+IL35+ Treg populations. Our observations suggest a strong developmental relationship between IL10+ and IL35+ Tregs.Interestingly, despite the distinct signaling pathways utilized by IL10 and IL35 receptors, both IL10- and IL35-deficient Treg mice presented a similar reduction of IR expression on CD8+ TILs and tumor growth, while double-deficient Treg mice did not show a notable additive phenotype. Our subsequent analyses demonstrated that Blimp1 is a common target of IL10 and IL35 signaling in CD8+ T cells, and its expression was significantly downregulated in CD8+ TILs from all the cytokine-deficient Treg mice.

**Conclusions**

Blimp1 has been reported as one of the key transcription factors that drive exhaustion of CD8+ T cells [4], but upstream external stimuli in the TME that trigger Blimp1 activities have not been fully understood. Here, we report the direct link between Treg-derived inhibitory cytokines, IL10 and IL35, and Blimp1-mediated CD8+ T cell exhaustion. These observations suggest that inflammatory cues in the TME induce the plasticity of IL10 and IL35 expression in Tregs that may further differentiate into functionally distinct subtypes of effector Tregs to cooperatively modulate the balance between exhausted and memory CD8+ TIL compartments.

**References**

1. Tanaka A, Sakaguchi S. Regulatory T cells in cancer immunotherapy. Cell Research. 2016; 27:109. 2. Stewart, CA, et al. Interferon-dependent IL-10 production by Tregs limits tumor Th17 inflammation. J Clin Invest. 2013; 123(11):4859-74. 3. Turnis, ME, et al. Interleukin-35 limits anti-tumor immunity. Immunity. 2016; 44(2):316-29. 4. Shin, H, et al. A role for the transcriptional repressor Blimp-1 in CD8(+) T cell exhaustion during chronic viral infection. Immunity. 2009; 31(2):309-20.

#### O34 Next-generation retroviral vector with membrane-anchored IL-12 to improve adoptive T cell immunotherapy and enhance its safety

##### John Davies, Carylinda Serna, Zhiya Yu, Nicholas Restifo, MD, Steven Rosenberg, MD, PhD, Christian Hinrichs, MD, Ling Zhang, PhD

###### National Institutes of Health, Bethesda, MD, USA

####### **Correspondence:** Christian Hinrichs (hinrichs@nih.gov)

**Background**

IL-12 is an important regulator of cell-mediated immunity. Though antitumor activity of IL-12 has been demonstrated in mouse tumor models, its clinical application was limited by systemic toxicity. Previously, we developed an IL-12 vector with a promoter containing binding motifs for Nuclear Factor of Activated T cell (NFAT-IL12) to secret IL-12 upon T cell activation. In a phase I/II clinical study using autologous tumor infiltrating lymphocytes (TIL) engineered with NFAT-IL12 in patients with metastatic melanoma, 62.5% of patients receiving a dose above 3X108 NFAT-IL12 TILs exhibited objective responses. However, increasing cell doses resulted in high serum levels of IL-12 and IFNg and clinical toxicities, including fever, liver dysfunction and hemodynamic instability. Therefore, a safer delivery of IL-12 is needed

**Methods**

To avoid detrimental toxicity of secreted IL-12, we designed a next-generation retroviral vector in which IL-12 is anchored on the cell surface by B7 transmembrane domain and cytoplasmic tail (LTR-mIL12.mB7). This vector was evaluated in two different murine tumor treatment models. The Pmel or OTI-T cells were transduced with membrane-anchored IL12 (LTR-mIL12.mB7) or secreting IL-12 (NFAT.mIL12) and were used to treat mice bearing B16 melanoma or B16-OVA melanoma. To further guide IL-12 expression at the tumor sites, we constructed NFAT inducible membrane-anchored IL12 vectors (NFAT-mIL12.mB7 and NFAT-hIL12.hB7). The treatment efficacy and safety profile of these vectors were evaluated in vitro and in vivo.

**Results**

Our results showed that T cells engineered with LTR-mIL12.mB7 could effectively control tumor burden, and importantly with an improved safety profile compared with T secreting IL-12. No IL-12 was detected in the serum and no body weight loss was observed from mice who were treated with membrane-anchored IL-12 T cells. In contrast, a transient body weight loss and increase serum level of IL-12 and IFNg were found in mice receiving T cells secreting IL-12. Furthermore, T cells modified with inducible membrane-anchored IL-12 vector could efficiently express IL-12 on cell surface with minimal IL-12 secretion upon T cell activation. Adoptive transfer of OT-I T cells modified by NFAT-mIL12.mB7 significantly enhanced tumor regression in a dose dependent manner without toxicity. In an NSG mice bearing human HPV positive epithelia tumor, T cells co-expression HPV16-E7 TCR and NFAT-hIL12.hB7 improved treatment efficacy without detection of IL-12 in serum.

**Conclusions**

Taken together, the results support a clinical application of this novel IL-12 vector for cell-based immunotherapy with controlled toxicity

### Immunosuppressive Cells in the Tumor Microenvironment

#### O35 Suppression of myeloid cell arginase activity leads to therapeutic response in a NSCLC mouse model by activating anti-tumor immunity

##### Esra Akbay, PhD^7^, Juan Miret^2^, Paul Kirschmeier^2^, Shohei Koyama^3^, Yvonne Li^2^, Glenn Dranoff, MD^4^, Peter Hammerman^4^, Chad Pecot^5^, Kwok-Kin Wong, MD, PhD^6^

###### ^1^University of Texas Southwestern Medical Center, Dallas, TX, USA; ^2^Dana-Farber Cancer Institute, Boston, USA; ^3^Osaka University, Osaka, Japan; ^4^Novartis, Cambridge, MA, USA; ^5^University of North Carolina, Chapel Hill, USA; ^6^New York University, New York, NY,USA; ^7^University of Texas Southwestern Medical, Dallas, TX, USA

####### **Correspondence:** Esra Akbay (esra.akbay@utsouthwestern.edu)

**Background**

Tumor orchestrated metabolic changes in the microenvironment limit generation of anti-tumor immune responses. Availability of arginine, a semi-essential amino acid, is critical for lymphocyte proliferation and function. Levels of arginine are regulated by the enzymes arginase 1,2 and nitric oxide synthase (NOS). However, the role of arginase activity in lung tumor maintenance has not been investigated in clinically relevant orthotopic tumor models.

**Methods**

RNA sequencing (RNA-seq) of sorted cell populations from mouse lung adenocarcinomas derived from immunocompetent genetically engineered mouse models (GEMM)s was performed. To complement mouse studies, a patient tissue microarray consisting of 150 lung adenocarcinomas, 103 squamous, and 54 matched normal tissue were stained for arginase, CD3, and Cd66b by multiplex immunohistochemistry. Efficacy of a novel arginase inhibitor compound 9 in reversing arginase mediated T cell suppression was determined in splenocyte ex vivo assays. Additionally, the anti-tumor activity of this compound was determined in vitro and in an autochthonous immunocompetent KrasG12D GEMM of lung adenocarcinoma model.

**Results**

Analysis of RNA-seq of sorted myeloid cells suggested that arginase expression is elevated in myeloid cells in the tumor as compared to the normal lung tissue. Accordingly, in the patient samples arginase 1 expression was mainly localized in the granulocytic myeloid cells and significantly elevated in both lung adenocarcinoma and squamous tumors as compared to the controls. Our ex vivo analysis demonstrated that myeloid derived suppressor cell (MDSC)s cause T cell suppression by arginine depletion, and suppression of arginase activity by a novel Arg1/2 inhibitor, compound 9, led to restoration of T cell function by increasing arginine. Treatment of KrasG12D GEMM of lung cancer model with compound 9 led to a significant tumor regression associated with increased T cell number and function, while it had no activity across several murine and human non-small cell (NSCLC) lung cancer lines in vitro.

**Conclusions**

We show that arginase expression is elevated in mouse and patient lung tumors. In a KRASG12D GEMM arginase inhibition diminished growth of established tumors. Our data suggest arginase as an immunomodulatory target that should further be investigated in lung or other tumors with high arginase activity.

**Ethics Approval**

Human tissue specimen investigations were performed after approval by an institutional review board at University of North Carolina Chapel Hill (IRB # 14-1755). Written informed consent was obtained from all patients.

#### O36 Neuropilin-1 stabilizes human Tregs in cancer patients leading to more potent suppressive function

##### Chuckran Chuckran, BS, Anthony Cillo, PhD, Jessica Moskovitz, MD, Ashwin Somasundaram, MD, John Kirkwood, MD, Francesmary Modugno, PhD, Robert Edwards, MD, Robert Schoen, MD, Robert Ferris, MD, PhD, Tullia Bruno, PhD, Dario A. Vignali, PhD

###### University of Pittsburgh School of Medicine, Pittsburgh, PA, USA

####### **Correspondence:** Dario A. Vignali (dvignali@pitt.edu)

**Background**

Regulatory T cells (Tregs) maintain peripheral tolerance;[1] however, in cancer, Tregs dampen anti-tumor immunity, contributing to disease progression.[2,3] Neuropilin-1 (NRP1) is required for intratumoral Treg stability as Treg-specific knockout of NRP1 leads to reduced tumor growth.[4]Genome-wide transcriptional analyses in mice revealed that intratumoral NRP1-deficient Foxp3+ Tregs develop an effector phenotype, characterized by interferon-gamma (IFNγ) production and decreased suppression of conventional T cells.[5] Whereas NRP1 is constitutively expressed on mouse Tregs, expression on human Tregs is activation-driven and thus may be modulated by immune processes. Furthermore, NRP1’s role in maintaining human Treg stability amidst proinflammatory signals is not known. We hypothesize that (1) surface NRP1 expression marks highly suppressive human Tregs, (2) NRP1 expression is driven by proinflammatory signals in the tumor microenvironment, and (3) NRP1 binding to its cognate ligand, Semaphorin 4A, is required for maximal suppressive function.

**Methods**

Phenotypic profiling of peripheral blood (PBL) and tumor infiltrating lymphocytes (TILs) from head and neck squamous cell carcinoma (HNSCC), melanoma, non-small cell lung, ovarian, and colorectal cancer patients was conducted by flow cytometry. Treg function was evaluated in vitro by a micro-scale suppression assay, which measures the ability of Tregs to suppress CD8+ T cell proliferation. Paired with our phenotyping, we cultured Tregs under various stimulatory conditions to query drivers of NRP1 expression. These included numerous cytokine conditions, T cell receptor stimulation, co-culture with antigen presenting cells, as well as blockade of specific costimulation/inhibitory receptors.

**Results**

NRP1+ Tregs are greatly enriched in cancer patient PBL and TIL across numerous malignancies, and high NRP1 expression on intratumoral Tregs negatively correlates with disease-free survival in HNSCC. NRP1+ Tregs upregulate several inhibitory receptors commonly found on Tregs, including TIGIT, ICOS, and TNFR2, as well as markers of proliferation and survival. NRP1+ Tregs also suppressed cytotoxic T cell proliferation to a greater degree than their NRP1- counterparts. NRP1+ Tregs were enriched in vitro in low interleukin-2 (IL-2) conditions as well as upon T cell activation. Proinflammatory cytokines, such as IFNγ, also drove increased NRP1 expression in a subset of cancer patient samples.

**Conclusions**

NRP1+ Tregs constitute a more suppressive human Treg subset based on their enhanced regulatory phenotype and function. Given the increased expression of proliferation and survival markers, especially under destabilizing conditions, NRP1 confers a survival advantage to these Tregs, allowing them to persist and function amidst such signals. Therefore, destabilizing intratumoral Tregs with NRP1 blockade may compliment other T cell therapies such as anti-PD1 blockade.

**References**

1. Vignali DAA, Collison LW, Workman CJ. How regulatory T cells work. Nat. Rev. Immunol. 2008; 8: 523–532.

2. Tanaka A, Sakaguchi S. Regulatory T cells in cancer immunotherapy. Cell Res. 2017: 27: 109–118.

3. Liu C, Workman CJ, Vignali DAA. Targeting regulatory T cells in tumors. FEBS J. 2016; 283:2731–2748.

4. Delgoffe GM, Woo SR., Turnis ME, Gravano DM, Guy C, Overacre AE, Bettini ML, Vogel P, Finkelstein D, Bonnevier J, Workman CJ Vignali DAA. Stability and function of regulatory T cells is maintained by a neuropilin-1–semaphorin-4a axis. Nature.. 2013; 501:252–256.

5. Overacre-Delgoffe AE, Chikina M, Dadey RE, Yano H, Brunazzi EA, Shayan G, Horne W, Moskovitz JM, Kolls JK, Sander C, Shuai Y, Normolle DP, Kirkwood JM, Ferris RL, Delgoffe GM, Bruno TC, Workman CJ, Vignali DAA. Interferon-γ Drives T reg Fragility to Promote Anti-tumor Immunity. Cell. 2017; 169:1130–1141.e11.

**Ethics Approval**

This study was approved by the local Institutional Review Board under protocol UPCI 99-069, and patients provided informed consent.

### Innate Anti-Tumor Immunity

#### O37 A new immunomodulatory strategy of inhibiting the glyco-immune checkpoint axis with EAGLE technology to treat cancer

##### Lizhi Cao^2^, Adam Petrone^2^, Lihui Xu, BS^1^, Wayne Galtlin, MS^1^, Michele Mayo^1^, Michal Stanczak^3^, Carolyn Bertozzi^4^, Karl Normington, PhD, MBA^1^, Jeff Brown^1^, Heinz Laubli^3^, Jim Broderick, MD^1^, Li Peng, PhD^2^

###### ^1^Palleon Pharmaceuticals, Waltham, MA, USA; ^2^Palleon Pharma, Waltham, MA, USA; ^3^University Hospital Basel, Switzerland, Switzerland; ^4^Stanford University, Stanford, CA, USA

####### **Correspondence:** Li Peng (lpeng@palleonpharma.com)

**Background**

Cancer therapy has been revolutionized by the recent developments of immune-checkpoint inhibitors to harness the power of the immune system in fighting cancer. However, the majority of patients fail to have durable responses or become resistant to immuno-oncology drugs, highlighting the need to identify new mechanisms of immune evasion in cancer and to develop new therapeutic modalities. Recently, the glyco-immune checkpoint axis (sialoglycan/Siglec pathway) has emerged as a novel mechanism of immune regulation involving both innate and adaptive immunity and an important mechanism of cancer immune escape. Upon ligation of sialylated glycans to ITIM-containing Siglecs on immune cells, this pathway plays a previously unrecognized role in regulating functions of NK cell, macrophages, dendritic cells, monocytes and T-cells in the tumor microenvironment. It suppresses multiple facets of anti-cancer immunity, including cancer antigen release, cancer antigen presentation, priming and activation of anti-cancer T-cell immunity, which may represent a novel mechanism of resistance to current immunotherapy.

**Methods**

Here, we described a novel therapeutic modality, a multi-functional antibody-like molecule named EAGLE (Enzyme-Antibody Glyco-Ligand Editing), to inhibit the glyco-immune checkpoint axis in the tumor microenvironment by selectively removing the terminal sialic acids of sialoglycans on tumor cells.

**Results**

We demonstrated that EAGLE decreased sialic acid levels on tumor cells and led to increased immune cell infiltration and activation in vivo in syngeneic mouse tumor models. EAGLE treatment achieved 50% complete regressions in well-established tumor models as a monotherapy with no reductions in body weight, and remarkably 100% cures in combination with anti-PD1 mAb. Furthermore, re-challenge experiments in cured mice from the EAGLE monotherapy treatment group resulted in a complete rejection of tumor cells, demonstrating that EAGLE induced anti-tumor immunological memory.

**Conclusions**

In summary, we demonstrated EAGLE as a novel and promising immunomodulatory therapeutic modality and the great potential of inhibiting the glyco-immune checkpoints for overcoming resistance to current immunotherapies.

#### O38 Remodeling the tumor microenvironment – Targeting scavenger receptors

##### Dhifaf Sarhan, PhD, Caroline Driescher, MS, Silke Sohn, MS, Salvatore Nania, Rainer Heuchel, Matthias Löhr, Mikael Karlsson

###### Karolinska Institutet, Solna, Sweden

####### **Correspondence:** Dhifaf Sarhan (dhifaf.sarhan@ki.se)

**Background**

Immunotherapy for cancer has revolutionized clinical practice and enabled cures for previously lethal cancers. However, the clinical responses are variable and highly influenced by immune regulatory compartments in the tumor microenvironment (TME). These include, Myeloid-derived suppressor cells (MDSC), and Tumor-associated macrophages (TAMs). We have previously shown that antibodies targeting scavenger receptors (SR) expressed on TAMs, reduces tumor growth and metastasis in murine melanoma and breast cancer models. Thus, we hypothesized that targeting these receptors will remodel the suppressive environment and relive the anti-tumor responses to increase the efficacy of immunotherapy.

**Methods**

To test our hypothesis, analysis of SR gene expression data from the Human Protein Atlas (HPA) project was performed investigating pancreatic tumors (n=176), as these consist of up to 80% stroma, compared with healthy pancreatic tissues (n=171). In vitro, we first cytokine-polarized macrophages from healthy blood donors towards M1 anti-tumor and M2 pro-tumor phenotype. Alternatively, macrophages were cultured with tumor cell lines under hypoxia and normoxia conditions. M1, M2, or tumor-conditioned macrophages were co-cultured with cytotoxic cells to mimic their interaction in the TME. Later, macrophages were treated with anti-SR Abs and their phenotype, metabolism, and cytokine profile were examined prior and following interaction with immune effector cells. Subsequently, T and NK cell activation was measured by cytokine production, proliferation, degranulation, and capacity to kill tumor cells.

**Results**

We found a 30-fold increase in SR expression in pancreatic tumors compared to healthy tissues. Also, a significant (p=0.03) correlation between high expression and decreased survival was noted in pancreatic cancer patients. Furthermore, pancreatic cancer cell lines induced SR expression on macrophages and dedifferentiated them towards MDSC. This effect was amplified by hypoxic condition. Notably, SR+ MDSC in contrast to control monocytes and M1-macrophages, suppressed cytotoxic cell anti-tumor activities, which was reversed by treatment with anti-SR Abs. In addition, targeting M2-macrophages with anti-SR Abs, abolished their anti-inflammatory phenotype and normalized their metabolism towards M1.

**Conclusions**

Our findings demonstrate a novel approach to specifically target M2-like TAMs and MDSC for treatment of pancreatic cancer.

### Mechanisms of Resistance to Immunotherapy

#### O39 PAK4 inhibition reverses immune cell exclusion and overcomes resistance to checkpoint blockade therapy

##### Gabriel Abril-Rodriguez, MS, Antoni Ribas, MD, PhD, Davis Torrejon, MD, Jesse Zaretsky, Theodore Nowicki, MD, PhD, Siwen Hu-Lieskovan, MD, PhD, Beata Berent-maoz, Begonya Comin-Anduix, PhD, Catherine GrassO

###### UCLA, Los Angeles, CA, USA

####### **Correspondence:** Antoni Ribas (aribas@mednet.ucla.edu)

**Background**

Lack of immune cell infiltration is the main mechanism of primary resistance to PD-1 blockade therapies for cancer.

**Methods**

Here, we performed a transcriptomic analysis of metastatic melanoma biopsies taken from patients treated with anti-PD-1 (n= 41) with biopsies pre- (n=27) and during treatment (n=33), and investigated cancer cell intrinsic mechanisms of immune evasion.

**Results**

We first performed RNAseq deconvolution to estimate the relative levels of the different immune cell populations and confirmed that on-treatment responding biopsies had increased levels of T-cells (p-value = 1.21e-05) and dendritic cells (p-value = 1.9e-05) compared to non-responding tumor biopsies. To elucidate potential mechanisms of immune cell exclusion, we performed differential gene expression analysis between infiltrated and non-infiltrated tumor biopsies. P21 (RAC1) Activated Kinase 4 (PAK4) was consistently enriched in tumor biopsies with low T-cell (q-value = 2.75e-07) and dendritic cell (q-value = 1.9e-05) infiltration and was validated using an independent cohort of 99 metastatic melanoma biopsies treated with anti-PD-1 published by Riaz et al. Cell 2017 (q-value= 1.59e-11). PAK4 is a kinase known to be involved in tumorigenesis that directly binds and phosphorylates β-catenin to activate Wnt signaling. In our series, PAK4 expression negatively correlated with several immune cell populations including T-cells (pcc = -0.21, p-value = 1.04e-07) and dendritic cells (pcc= -0.49, p-value = 6.60e-05). Furthermore, tumor biopsies from patients without a response to PD-1 blockade showed increased levels of PAK4 expression (p-value = 0.004). We performed a pan-cancer correlation analysis between PAK4 expression and T-cell infiltration using TCGA expression data from 32 cancer types, identifying a negative correlation in melanoma (pcc = -0.31 and p-value= 7.4e-12), prostate cancer (pcc = -0.26 and p-value= 7.9e-09) and pancreatic cancer (pcc = -0.41 and p-value= 1.6e-08) among 20 other cancer types with similar negative correlation. To study the anti-tumor efficacy of PD-1 blockade in the context of PAK4 deletion, we treated syngeneic C57BL/6 mice (n=7 each group) bearing either B16 PAK4 KO or B16 WT tumors with anti-PD-1. We observed anti-tumor efficacy in the B16 PAK4 KO anti-PD-1 treated group (p-value = 0.0006) while no significant difference was found in the B16 WT treated group (p-value = 0.11). Depletion of CD8 T-cells abrogated the anti-tumor efficacy observed in the B16 PAK4 KO group.

**Conclusions**

In summary, high PAK4 expression is correlated with non-responding tumor biopsies with low T and dendritic cell infiltration, and inhibition of PAK4 overcomes resistance to PD-1 blockade in a CD8 dependent manner.

**References**

1. Riaz, N., et al., Tumor and Microenvironment Evolution during Immunotherapy with Nivolumab. Cell, 2017. 171(4): p. 934-949 e15.

**Ethics Approval**

The study was approved by UCLA IRBs 11-001918 and 11-003066.Mice were used under the UCLA Animal Research Committee protocol #2004-159-23.

#### O40 Anti-CTLA4 activation of intratumoral NK cells may contribute to intratumoral Treg depletion

##### Erin O'Brien, MS^1^, Emilio Sanseviero^1^, Jenna Karras, PhD^1^, Tamer Shabaneh^2^, Bulent Aksoy, PhD^3^, Wei Xu^4^, Xiaowei Xu^4^, Giorgos Karakousis^4^, Ravi Amaravadi, MD^5^, Mark Rubinstein, PhD^3^, Mary Jo Turk^2^, Jeffrey Hammerbacher, BA^3^, Lynn Schuchter, MD^4^, Tara Mitchell^4^, Qin Liu^1^, Erica Stone, PhD^1^

###### ^1^The Wistar Institute, Philadelphia, PA, USA; ^2^Norris Cotton Cancer Center, Dartmouth, Lebanon, NH, USA; ^3^MUSC, Charleston, SC, USA; ^4^University of Pennsylvania, Philadelphia, PA, USA; ^5^The University of Pennsylvania, Philadelphia, PA, USA

####### **Correspondence:** Erica Stone (estone@wistar.org)

**Background**

Antibodies targeting CTLA4 induce durable responses in some patients with melanoma and are being tested in a variety of human cancers. However, a majority of patients across tumor types fail to respond. Further understanding the mechanisms of action of these therapies will enable the development of novel strategies to overcome resistance and biomarkers to identify patients most likely to respond. In murine models anti-CTLA4 efficacy depends on interactions between the Fc region of anti-CTLA4 antibodies and Fc receptors (FcRs). Anti-CTLA4 binding to FcRs has been linked to depletion of intratumoral Tregs by myeloid cells including non-classical monocytes. However, FcR engagement can lead to Natural Killer (NK) cell activation and NK cells can mediate antibody-dependent cell mediated cytotoxicity (ADCC).

**Methods**

Flow cytometry was used to investigate surface CTLA4 expression on murine and patient T cell subsets, including tumor-infiltrating lymphocytes and T cells from matched patient blood. Using murine tumor models we assessed the effect of anti-CTLA4 administration on intratumoral and peripheral immune cells including NK cells. A previously published RNA-seq data set was used to investigate intratumoral CD56 expression in cutaneous melanoma patients who benefited from Ipilimumab treatment compared to those who did not.

**Results**

In agreement with previous studies, we found that in murine models intratumoral Tregs have the highest expression of surface CTLA4 (sCTLA4) and anti-CTLA4 leads to Fc/FcR-dependent depletion of these Tregs. Similarly, analysis of T cells infiltrating patient-derived tumor tissue showed that Tregs have the highest sCTLA4 expression, and intratumoral Tregs express significantly more sCTLA4 than circulating Tregs. Interestingly, cutaneous melanoma patients who benefited from Ipilimumab treatment had higher intratumoral expression of the NK cell marker CD56. Using murine tumor models, we found that anti-CTLA4-induced Treg depletion coincided with activation and degranulation of intratumoral NK cells.

**Conclusions**

Taken together, our data suggest that anti-CTLA4-induced Treg depletion may be mediated in part by NK cells. These results suggest new strategies to overcome resistance to anti-CTLA4 therapies.

**Ethics Approval**

Patient-derived tumor tissue specimens were collected in accordance to the Institutional Review Boards at HFGCC of Christiana Health Care System or The University ofPennsylvania.

#### O41 Withdrawn

### Micro-RNA, Epigenetics and Tumor/Immune-cell Signaling Pathways in Anti-Tumor Immunity

#### O42 LSD1 inhibition promotes CD141Hi dendritic cell differentiation in myelodysplastic syndromes

##### Pragya Srivastava, PhD, Pragya Srivastava, PhD, Kyle Wiatrowski, BS, Prashant Singh, PhD, Eduardo Cortes Gomez, MS, Jianmin Wang, PhD, Miranda Lynch, PhD, Sheila Sait, PhD, Laurie Ann Ford, BS, Brandon Martens, BS, Linda Lutgen-Dunckley, BS, Elizabeth Griffiths, MD, Michael Nemeth, PhD, Stephanie Tzetzo, MA, Scott Abrams, PhD

###### Roswell Park Comprehensive Cancer Center, Buffalo, NY, USA

####### **Correspondence:** Michael Nemeth (Michael.Nemeth@RoswellPark.org); Scott Abrams

**Background**

Immunotherapeutic approaches for patients with the myelodysplastic syndrome (MDS) have shown promise. Progress is limited by incomplete understanding of the immunologic milieu in MDS. We performed a phase I study of vaccination against the tumor antigen NY-ESO-1 in combination with decitabine in newly diagnosed patients with MDS (1). Response to vaccination was associated with the presence of CD141Hi conventional dendritic cells (cDCs). This population of cells is critical to effective anti-tumor immune responses in solid tumors. We confirmed a deficiency of CD141Hi cDCs in MDS patients and proceeded to identify translatable regulatory mechanisms to enhance their development.

**Methods**

We quantified DC populations in bone marrow from MDS patients (n = 61) using flow cytometry. RNA-seq analysis was used to assess gene expression in MDS and healthy CD34+ progenitors. We assessed CD141Hi differentiation of MDS and healthy CD34+ progenitors using an in vitro differentiation model.

**Results**

We binarized patients into cohorts based on detectable (n = 36) versus undetectable (i.e. below the limit of detection; n = 25) CD141Hi cDC populations. Patients with detectable numbers of CD141Hi cDCs had longer median progression-free and overall survival compared to patients with undetectable CD141Hi cDCs (416 versus 216 days and 650 versus 304 days respectively). The transcription factor IRF8 is a master regulator of CD141Hi cDC differentiation. Expression of IRF8 in MDS progenitors (as measured by RNA-seq) was associated with detectable numbers of CD141Hi cDCs. This result suggests that increased IRF8 expression drives enhanced CD141Hi cDC differentiation. We hypothesized that inhibition of lysine-specific histone demethylase 1A (LSD1), known to enhance IRF8 expression, would induce CD141Hi cDC differentiation (2). We showed that pharmacologic LSD1 inhibition (GSK2879552; GSK) enhances IRF8 expression in KG1 AML cells. Treatment of MDS CD34+ progenitors with GSK increased the number of CD141Hi cDCs in 2/4 patients samples compared to untreated controls (by 2.7 and 25.7-fold increase respectively). Cultured cells maintained MDS associated cytogenetic abnormalities (del 5q and -7), indicating their derivation from the malignant population. LSD1 inhibition also enhanced differentiation of healthy CD34+ cells to CD141Hi cDCs. This approach can therefore drive differentiation of CD141Hi cDCs which can arise from both malignant and healthy CD34+ cells.

**Conclusions**

These data highlight an unrecognized and potentially reversible immune defect in patients with MDS. Since immunotherapeutic strategies, including checkpoint inhibitors and vaccinations, require functional CD141Hi cDCs, strategies to enhance this population are critical for effective immune therapy for MDS patients.

**References**

1. Griffiths EA et al. Clin Cancer Res. 2018;24:1019-1029.2. Maiques-Diaz A et al. Cell Rep. 2018;22:3641-3659.

**Ethics Approval**

The study was approved by the Roswell Park Institututional Review Board (BDR 079816 and NCT01834248).

### Oncolytic Viruses and Intratumoral Therapies

#### O43 Characterization of anti-tumor immune responses and Effects on Survival of Neoadjuvant Oncolytic Virotherapy in spontaneous Osteosarcoma

##### Shruthi Naik, PhD^1^, Kelly Makielski, DVM, MS^2^, Michael Henson, DVM, PhD^2^, Kathleen Stuebner^2^, Alexander-Flaviu Tabaran, DVM, MSc, PhD^2^, Ingrid Cornax, PhD^2^, Gerard O'Sullivan, MVB, PhD^2^, Andrea Eckert^2^, Lauren Mills, PhD^2^, Milcah Scott, BS^2^, Aaron Sarver, PhD^2^, Michael Farrar^2^, Stephen Russell, MD, PhD^1^, Jaime Modiano, VMD, PhD^2^

###### ^1^Mayo Clinic, Rochester, MN, USA; ^2^University of Minnesota, Minneapolis, MN, USA

####### **Correspondence:** Jaime Modiano (modiano@umn.edu)

**Background**

Development of effective therapies for osteosarcoma, an infrequent disease that primarily affects adolescents and young adults, is a critical unmet medical need. The rarity and heterogeneity of these cancers have hindered research in this field. Comparative oncology studies in naturally occurring osteosarcoma in companion dogs provide opportunities to advance development in a clinically realistic setting where the tumors resemble their human counterparts but where the barriers of rarity and cost are lowered [1]. Vesicular stomatitis virus (VSV) is a rapidly replicating, robustly immunogenic oncolytic virus platform with demonstrated cytotoxicity in canine osteosarcoma cell lines. VSV-IFNβ-NIS, a recombinant oncolytic VSV, can be safely administered intravenously (IV) to reach sites of metastatic disease and has been shown selectively replicate in and destroy tumor cells and activate an anti-tumor immune response [2]. The aim of this study is to evaluate neoadjuvant VSV-IFNβ-NIS therapy for osteosarcoma using a comparative oncology approach and characterize anti-tumor immune responses in spontaneous canine osteosarcoma.

**Methods**

A veterinary clinical study was initiated. Dogs with osteosarcoma are randomized to receive neoadjuvant oncolytic VSV-IFNβ-NIS (109 TICD50/kg) or placebo followed by amputation and carboplatin chemotherapy. Pre- and post-treatment tumor biopsies and serial peripheral blood mononuclear cells are obtained to assess anti-tumor immunity by histopathology, RNA and DNA sequencing, and lymphocyte effector functions.

**Results**

To date 24 dogs have been enrolled, of 30 dogs planned. The VSV safety profile is excellent, with mild, transient changes in body temperature and evidence of acute cytokine responses. Preliminary analyses show survival outcomes exceed the expectation for standard-of-care alone. Focal tumor necrosis that is potentially treatment-related has been observed in osteosarcoma lesions in resected tissue. Assessment of naïve and treatment-associated gene cluster expression summary scores from RNA sequencing is ongoing, as is massive parallel sequencing of lymphocyte antigen receptors to describe clonal expansion and attrition.

**Conclusions**

Neoadjuvant VSV treatment is well-tolerated, and shows preliminary evidence of biological activity and clinical efficacy. Updated results describing anti-tumor immunity that is attributable to oncolytic VSV, and its effects on patient outcomes will be presented. These data will indicate if intravenous neoadjuvant VSV-IFNβ-NIS therapy improves clinical outcomes in canine osteosarcoma and inform clinical studies to evaluate this therapeutic approach as an addition to current chemotherapy protocols for osteosarcoma patients.

**Consent**

1. Scott, M.C., et al., Molecular subtypes of osteosarcoma identified by reducing tumor heterogeneity through an interspecies comparative approach. Bone, 2011. 49(3): p. 356-67.

2. Naik, S., et al., Curative one-shot systemic virotherapy in murine myeloma. Leukemia, 2012. 26(8): p. 1870-8.

### T-cell Checkpoints and Checkpoint Inhibitors

#### O44 FS120 mAb^2^, a dual agonist bispecific antibody targeting OX40 and CD137, activates T cells *in vitro* and induces potent, FcγR-independent anti-tumour activity

##### Miguel Gaspar, PhD, Cyril Privezentzev, PhD, Katy Everett, PhD, Sandra Uhlenbroich, John Pravin, Leonor Rodrigues, MSc, Delphine Buffet, Marine Houee, Francisca Wollerton, Melanie Medcalf, Edouard Souteyrand, Alexander Koers, PhD, Emma McConnell, Master in Phyhsiology and Pharmacology for Researc, Martyn Rhoades, miat, Mateusz Wydro, PhD, Maud Berthelot, MSc, Sarah Batey, Michael Davies, Jacqueline Doody, PhD, Michelle Morrow, PhD, Mihriban Tuna, PhD, Neil Brewis, PhD

###### F-star Biotechnology ltd, Cambridge, UK

####### **Correspondence:** Miguel Gaspar (Miguel.Gaspar@f-star.com)

**Background**

Following the success of checkpoint blockade, activation of the co-stimulatory Tumour Necrosis Factor Receptor (TNFR) superfamily receptors represents the next stage of cancer immunotherapy with clinical trials underway for antibodies stimulating T cell co-stimulatory pathways. Targeting OX40 and CD137 has the potential to strongly activate the immune system due to their broad expression across CD4+ and CD8+ T cells and NK cells. However, FcγR-mediated crosslinking is often required for the activity of monoclonal antibodies, and this likely limits clinical activity, due to the inherently low affinity of Fc:FcγR interactions, as well as due to FcγR-mediated depletion of T cells through ADCC. FS120 is a novel, dual agonist bispecific antibody that does not bind to FcγR, but instead crosslinks OX40 and CD137 resulting in potent activation of both CD4+ and CD8+ T cells, independent of FcγR binding.

**Methods**

FS120 was generated by introducing an OX40-binding specificity into the Fc-region of a human IgG1 targeting CD137. FcγR binding was significantly decreased using the LALA mutation. A parallel approach was taken to generate a murine surrogate molecule to test *in vivo*.

**Results**

FS120 binds simultaneously to OX40 and CD137 with subnanomolar affinity and has strong activity in PBMC and T cell stimulation assays. Conventional OX40 and CD137 agonist antibodies require crosslinking, e.g. via anti-Fc secondary antibodies for their activity. OX40 agonist antibodies activate CD4+ T cells, but not CD8+ T cells and CD137 agonist antibodies activate CD8+ T cells but not CD4+ T cells. FS120 has subnanomolar dual agonist activity on both CD4+ and CD8+ T cells, which is independent of additional crosslinking. This activity is dependent on concurrent binding to the two receptors. An anti-mouse OX40/CD137 bispecific antibody showed greater anti-tumour activity than a combination of OX40 and CD137 agonists in a CT26 mouse tumour model, which was associated with peripheral T cell activation and proliferation. The anti-tumour activity was independent of T regulatory cell (Treg) depletion, as evidenced by similar levels of tumour infiltrating Treg cells in mice treated with the anti-mouse OX40/CD137 mAb² and isotype control. Anti-tumour activity was also demonstrated in a B16-F10 syngeneic model.

**Conclusions**

An OX40/CD137-specific mAb^2^ can autonomously stimulate both CD4+ and CD8+ T cells *in vitro* independent of FcγR binding and mediate potent anti-tumour activity *in vivo* via an FcγR-independent mechanism of action. These data support initiation of clinical development of FS120, a first-in-class dual agonist bispecific antibody for the treatment of human cancer.

**Acknowledgements**

We thank the following people for their technical assistance:*In vivo* team for animal studies, Protein sciences team for protein production

**Ethics Approval**

Murine studies were conducted under a U.K. Home Office License in accordance with the U.K. Animal (Scientific Procedures) Act 1986 and EU Directive EU 2010/63.

#### O45 Refractory renal cell cancer (RCC) exhibits high adenosine A2A receptor (A2AR) expression and prolonged survival following treatment with the A2AR antagonist, CPI-444

##### Lawrence Fong, MD^1^, Lawrence Fong, MD^1^, John Powderly, MD, CPI^2^, Jason Luke, MD, FACP^3^, Drew Hotson, PhD^4^, Mario Sznol, MD^5^, Saby George, MD, FACP^6^, Toni Choueiri^7^, Brian Rini, MD^8^, Matthew Hellmann, MD^9^, Shivaani Kummar, MD^10^, Leonel Hernandez-Aya, MD PhD^11^, Daruka Mahadevan, MD, PhD^12^, Brett Hughes, MD^13^, Ben Markman^14^, Matthew Riese, MD, PhD^15^, Joshua Brody, MD^16^, Daniel Renouf, MD^17^, Rebecca Heist, MD^18^, Rachel Goodwin, MD^19^, Amy Weise, DO^20^, Leisha Emens, MD, PhD^21^, Stephen Willingham, PhD^4^, Long Kwei, PhD^4^, Ginna Laport, MD^4^, Richard Miller, MD^4^

###### ^1^University of California, San Francisco, CA, USA; ^2^Carolina BioOncology Institute, Huntersville, NC, USA; ^3^University of Chicago, Chicago, MA, USA; ^4^Corvus Pharmaceuticals, Burlingame, CA, USA; ^5^Yale University, New Haven, CT, USA; ^6^Roswell Park Cancer Institute, Buffalo, NY, USA; ^7^Dana Farber Cancer Institute, Boston, MA, USA; ^8^Cleveland Clinic Foundation, Cleveland, OH, USA; ^9^Memorial Sloan Kettering Cancer Center, New York, NY, USA; ^10^Stanford University School of Medicine, Stanford, CA, USA; ^11^Washington University, Saint Louis, MO, USA; ^12^University of Arizona, Tucson, AZ, USA; ^13^Royal Brisbane Hospital, Herston, Australia; ^14^Monash Health and Monash University, Melbourne, VIC, Australia; ^15^Medical College of Wisconsin, Milwaukee, WI, USA; ^16^Icahn School of Medicine at Mt Sinai, New York, NY, USA; ^17^British Columbia Cancer Agency, Vancouver, Canada; ^18^Massachusetts General Hospital, Boston, MA, USA; ^19^Ottawa Hospital Cancer Center, Ottawa, Canada; ^20^Karmanos Cancer Institute, Detroit, MI, USA; ^21^Johns Hopkins University, Pittsburgh, PA, USA

####### **Correspondence:** Richard Miller (rmiller@corvuspharma.com)

**Background**

Adenosine plays a role in blocking the function of immune cells through binding to the A2AR. CPI-444 is an oral A2AR antagonist that has been evaluated in Phase 1 trials in advanced cancer.

**Methods**

This Phase 1b trial was conducted at 30 centers. RCC patients (pts) with progressive disease were randomized to receive either CPI-444, 100 mg po bid monotherapy or CPI-444 in combination with atezolizumab 840 mg IV every 2 weeks and treated until progression or unacceptable toxicity. Endpoints included safety, tumor response (RR), progression free survival and overall survival (OS). Peripheral blood and tumor biopsies were obtained at baseline and on-treatment and evaluated for lymphocyte subsets and gene expression of A2AR using Nanostring. A2AR signaling was assessed by measurement of inhibition of pCREB.

**Results**

Results: 68 pts enrolled (N=33 monotherapy, N=35 combination); median prior therapies was 3 (range 1-5) including TKI, 84%; anti-PD(L)1 (IO), 72%; median time since last IO was 3.1 and 1.7 months for monotherapy and combination, respectively. Median treatment duration was 4.6 months. CPI-444 was well-tolerated with no Gr3/4 toxicity in monotherapy; 7 pts had reversible Gr3 toxicity in combination. CPI-444 blocked A2AR signaling based on inhibition of phosphorylation of CREB in blood lymphocytes. A2AR mRNA was measured in 31 pre-treatment RCC tumor biopsies and compared to other cancers (36 lung; 12 bladder; 5 colon; 8 prostate, 9 melanoma and 20 triple negative breast). RCC showed increase in A2AR expression compared to lung (p<0.01) and the other tumors (p<0.001). Objective clinical responses were observed in 8% of pts; another 21% had reduction of tumor not meeting criteria for response. Responses were seen in both monotherapy and combination arms and in pts who failed prior IO agents. 40% and 58% of pts experienced disease control (DC) for > 3 months (confirmed scans) in monotherapy and combination, respectively, including in pts resistant to prior IO. The OS for both monotherapy and combination exceeded 80% at 16 months. Treatment increased CD8+ T cells in tumors in pts with DC>6mo compared to DC<6mo (p<0.02).

**Conclusions**

CPI-444 is active in RCC and is associated with prolonged survival and DC in treatment refractory pts, including those who have failed prior IO. Prolonged DC was associated with treatment-induced increase in tumor infiltrating CD8+ cells. Our results compare favorably to those reported with atezolizumab demonstrating 1 yr OS of 81% and RR of 15% in IO naïve RCC with median 2 prior therapies[1].

**Acknowledgements**

not applicable

**Trial Registration**

NCT02655822

**References**

1. McDermott D, Sosman J, Sznol M, Massard C, Gordon M, Hamid O, Powderly J, Infante J, Fasso M, Wang Y, Zou W, Hegde P, Fine G, Powles T. Atezolizumab, an anti-programmed death- ligand 1 antibody, in metastatic renal cell carcinoma: long-term safety, clinical activity, and immune correlates from a Phase 1a study. J Clin Oncol. 2016;34:1-10.

**Ethics Approval**

The protocol was approved by the institutional review board or ethics committee at each participating center.

#### O46 Increased tumor-resident memory T cells in breast cancer is associated with improved prognosis

##### Peter Savas^1^, Balaji Virassamy^1^, Chengzhong Ye^2^, Agus Salim^3^, Chris Mintoff^1^, Franco Caramia^1^, Roberto Salgado^4^, Zhi Ling Teo^1^, Sathana Dushyanthen^1^, Ann Byrne^1^, Stephen Luen^1^, Paul Beavis, PhD^1^, Stephen Fox^1^, Phillip Darcy^1^, Terence Speed^2^, Laura Mackay^5^, Paul Neeson, PhD^6^, Sherene Loi, MD, PhD^6^

###### ^1^Peter MacCallum Cancer Center, Melbourne, Australia; ^2^WEHI, Melbourne, Australia; ^3^La Trobe University, Melbourne, Australia; ^4^GZA Ziekenhuizen, Antwerp, Belgium; ^5^University of Melbourne, Melbourne, Australia; ^6^Peter MacCallum Cancer Centre, Melbourne, Australia; ^7^Peter Mac Callum Cancer Center, Victoria, Australia

####### **Correspondence:** Paul Neeson (paul.neeson@petermac.org)

**Background**

The level of tumor infiltrating lymphocytes (TIL) is a prognostic factor for improved patient survival in triple negative and HER2-overexpressing breast cancer (BC) subtypes. T cells are the main immune subset in BC tumors with a high TIL content, TIL(hi); however the influence of the T cell qualitative response on patient prognosis is unknown.

**Methods**

To address this issue, TILs were isolated from 129 primary and metastatic BC samples, T cells were sorted and single-cell RNA sequencing (scRNA-seq) performed to reveal the BC TIL T cell clusters present. BC TIL scRNAseq data was confirmed by multi-parameter flow cytometry (FACS). We also performed bulk RNA seq on FACS-sorted T cell populations, and interrogated BC TIL T cell receptor (TCR) sequences to compare the difference in TCR usage between clusters. We explored BC TIL functional responses (cytokines, effector proteins) following co-culture with autologous BC cells. Finally we compared the clinical outcome data for triple negative BC patients who expressed high vs low levels of the signature clusters in BC TILs.

**Results**

Our study showed BC T cells were heterogeneous in sub-type and functional polarization. In particular, BC TIL(hi) cases contained increased CD8+ tumor-resident memory T (TRM) cells. These cells expressed CD103 and high levels of immune checkpoint molecules (PD1, CTLA-4, TIM-3, Lag-3) and T cell effector proteins (perforin and granzyme B). Further analysis of BC TILs by multi-parameter FACS confirmed the presence of increased CD8+ TRM in TIL(hi) BC tumors, these CD8+ TRM also released granzyme B on co-culture with autologous BC cells. In two primary tumors, the BC TRM had different TCR usage to TEM suggesting these are clonally distinct populations. Using the scRNAseq data, we developed a CD8+ TRM gene signature that was associated with improved patient disease free survival (DFS), (n=329, log-rank p=0.003) in early-stage triple negative breast cancers (TNBC) from the METABRIC data set. The CD8+ TRM gene signature also stratified patients with high vs low CD8A expression for DFS (log-rank p = 0.03).

**Conclusions**

In conclusion, patients with BC TIL(hi) tumours have a qualitatively different T cell response which includes CD8+ TRM differentiation. These cells express high levels of immune checkpoint molecules, plus T cell effector proteins suggesting they could be the key responders to immune checkpoint inhibitor therapy. Further studies exploring BC-associated CD8+ TRM cell development and homeostasis will be critical for creating new opportunities for BC immunotherapy.

**Acknowledgements**

We wish to thank H Thorne, E Niedermayr, all the kConFab research nurses and staff, the heads and staff of the Family Cancer Clinics, and the Clinical Follow Up Study (which has received funding from the NHMRC, NBCF, Cancer Australia, and the National Institute of Health (USA)) for their contributions to this resource, and the many families who contribute to kConFab. kConFab is supported by a grant from NBCF, and previously NHMRC, the Queensland Cancer Fund, the Cancer Councils of New South Wales, Victoria, Tasmania and South Australia, and the Cancer Foundation of Western Australia. We wish to thank the FACS core facility staff R Rossi, V Milovac and S Curcio, and T Tan and P Petrone for additional FACS assistance. We also thank S Ellis for assistance with confocal imaging, G Mir Arnau for facilitating RNASeq, and the Anatomical Pathology staff at the Peter MacCallum Cancer Centre. Special thanks also to J Jabbari and the Australian Genome Research Facility for making the single cell sequencing possible.

**Ethics Approval**

This project was approved by the Human Research Ethics Committee of the Peter MacCallum Cancer Centre (project approval number “SEGMENT” 13/123, Kathleen Cuningham Foundation Consortium for Research into Familial Breast Cancer (kConFab) project approval numbers #129 and #150).

**Consent**

All participating patients provided written informed consent.There is no sensitive or identifiable information in the abstract.

#### O47 Peripheral T cell dynamics in resectable NSCLC patients treated with neoadjuvant PD-1 blockade

##### Jiajia Zhang, MD, MPH^1^, Zhicheng Ji^1^, Margueritta El Asmar, MD^1^, Justina Caushi^1^, Valsamo Anagnostou, MD PhD^1^, Hok Yee Chan, MS^1^, Prerna Suri, MS^1^, Haidan Guo, BS, and BA^1^, Kristen Marrone, MD^1^, Jarushka Naidoo, MD^1^, Taha Merghoub, PhD^2^, Jamie Chaft, MD^2^, Matthew Hellmann, MD^2^, Janis Taube, MD, MSC^1^, Julie Brahmer, MD^1^, Victor Velculescu, MD, PhD^1^, Ni Zhao^1^, Patrick Forde, MD^1^, Drew Pardoll, MD, PhD^1^, Hongkai Ji^1^, Kellie Smith, PhD^1^

###### ^1^Johns Hopkins University, Baltimore, MD, USA; ^2^Memorial Sloan Kettering Cancer Center, New York, NY, USA

####### **Correspondence:** Kellie Smith (ksmit228@jhmi.edu)

**Background**

Neoadjuvant PD-1 blockade has recently been shown to induce major pathologic responses (MPRs) and delay time to relapse in patients with resectable non-small cell lung cancer (NSCLC)[1]. While the role of tumor-specific T cells in facilitating tumor regression has been demonstrated in advanced NSCLC, it is unknown how neoadjuvant PD-1 blockade affects the anti-tumor T cell repertoire and how these factors correlate with clinical outcome. The neoadjuvant setting provides a unique opportunity to evaluate T cell mobility into the tumor and other tissues after checkpoint blockade, which is challenging in studies of patients with metastatic disease.

**Methods**

Patients with resectable NSCLC were treated with neoadjuvant PD-1 blockade (NCT02259621). T cell receptor (TCR) sequencing was performed on pre- and post-treatment tissues, bulk serial peripheral blood samples, and pre-treatment blood sorted for PD-1 expression. Whole exome sequencing and neoantigen prediction was performed on pre-treatment tumor biopsies and matched normal lung tissue. Peripheral dynamics of intratumoral T cell clonotypes, as well as enrichment of TCR motifs were evaluated. The associations of T cell dynamics and neoantigen recognition with MPRs and recurrence-free survival were assessed.

**Results**

Substantial alterations in the T cell repertoire and influx of peripheral T cell clonotypes into tumor tissue, normal lung, and lymph nodes were observed following PD-1 treatment. Peripheral TCR responses were independent of MPR status. T cell clonality in the resected tumorpost-treatment tissue positively correlated with pre-treatment tumor mutational burden and negatively correlated with percent residual tumor. Tumor infiltrating clonotypes underwent dynamic peripheral remodeling on-treatment; the magnitude of clonal reshaping was significantly higher than clonotypes not found in the tumor. Tumor-infiltrating clonotypes that were also detected in the peripheral blood were of significantly higher frequency than those only present in the tumor. Notably, an anergic/monoclonal anti-tumor TCR repertoire was observed in a patient with KRAS/STK11 co-mutations and an early relapse after PD-1 blockade. Analyses of the dynamics of T cell clonotypes with differential PD-1 expression is ongoing.

**Conclusions**

Significant and systemic alterations exist in the peripheral anti-tumor T cell repertoire in NSCLC patients treated with neoadjuvant PD-1 blockade. The periphery represents a vital biological compartment for the anti-tumor immune response.

**Trial Registration**

www.clinicaltrials.gov (NCT02259621)

**References**

1. Forde PM, Chaft JE, Smith KN, et al. Neoadjuvant PD-1 Blockade in Resectable Lung Cancer. N Engl J Med 2018; 378: 1976–86.

**Ethics Approval**

The study was approved by Johns Hopkins Institutution‘s Ethics Board, approval number CIR00038778

**Consent**

Written informed consent was obtained from the patient for publication of this abstract and any accompanying images. A copy of the written consent is available for review by the Editor of this journal.

